# Inhibition of *miR338* rescues cleidocranial dysplasia in *Runx2* mutant mice partially via the *Hif1a*-*Vegfa* axis

**DOI:** 10.1038/s12276-022-00914-w

**Published:** 2023-01-04

**Authors:** Runze Jin, Hanshu Zhang, Chujiao Lin, Jinqiang Guo, Weiguo Zou, Zhi Chen, Huan Liu

**Affiliations:** 1 The State Key Laboratory Breeding Base of Basic Science of Stomatology & Key Laboratory for Oral Biomedicine of Ministry of Education, School and Hospital of Stomatology, Wuhan University, 237 Luoyu Road, Wuhan, 430079 China; 2 Division of Rheumatology, Department of Medicine, University of Massachusetts Medical School, Worcester, MA 01605 USA; 3 State Key Laboratory of Cell Biology, Shanghai Institute of Biochemistry and Cell Biology, CAS Center for Excellence in Molecular Cell Sciences, Chinese Academy of Sciences, University of Chinese Academy of Sciences, Shanghai, China; 4 Department of Periodontology, School of Stomatology, Wuhan University, Wuhan, 430079 China; 5 Taikang Center for Life and Medical Sciences, Wuhan University, Wuhan, China

**Keywords:** Bone development, Differentiation, miRNAs

## Abstract

Haploinsufficiency of Runt-related transcription factor-2 (*RUNX2*) is responsible for cleidocranial dysplasia (CCD), a rare hereditary disease with a range of defects, including delayed closure of the cranial sutures and short stature. Symptom-based treatments, such as a combined surgical-orthodontic approach, are commonly used to treat CCD patients. However, there have been few reports of treatments based on *Runx2*-specific regulation targeting dwarfism symptoms. Previously, we found that the *miR338* cluster, a potential diagnostic and therapeutic target for postmenopausal osteoporosis, could directly target *Runx2* during osteoblast differentiation in vitro. Here, we generated *miR338*^−/−^;*Runx2*^+/−^ mice to investigate whether inhibition of *miR338* could rescue CCD defects caused by Runx2 mutation in vivo. We found that the dwarfism phenotype caused by *Runx2* haploinsufficiency was recovered in *miR338*^−/−^;*Runx2*^+/−^ mice, with complete bone density restoration and quicker closure of fontanels. Single-cell RNA-seq analysis revealed that knockout of *miR338* specifically rescued the osteoblast lineage priming ability of bone marrow stromal cells in *Runx2*^+/−^ femurs, which was further confirmed by *Osterix*-specific conditional knockout of *miR338 in Runx2*^+/−^ mice (*OsxCre; miR338* ^*fl/fl*^*;Runx2*^*+/−*^*)*. Mechanistically, ablation of the *miR338* cluster in *Runx2*^+/−^ femurs directly rescued the *Hif1a*-*Vegfa* pathway in *Runx2*^+/−^ osteoblasts, as proven by gene expression profiles and ChIP and Re-ChIP assays. Collectively, our data revealed the genetic interaction between *Runx2* and the *miR338* cluster during osteoblast differentiation and implied that the *miR338* cluster could be a potential therapeutic target for CCD.

## Bone development: microRNA in a congenital skeletal disorder

A tiny RNA with a role in adult osteoporosis may also offer a useful therapeutic target for a category of congenital disorders that impair bone development throughout life. Children born with cleidocranial dysplasia (CCD) exhibit osteoporosis and other skeletal abnormalities. CCD is specifically linked to mutations in a gene called *RUNX2*. Zhi Chen and Huan Liu of Wuhan University in China have now shown that *RUNX2* is negatively regulated by *miR338* cluster, a pair of short, non-protein-coding RNAs associated with postmenopausal osteoporosis. *RUNX2-*mutant mice exhibit dwarfism and other developmental issues but these effects could be countered by further genetically modifying these animals so that they also lacked *miR338*. These results suggest that interventions targeting the *miR338* pathway could reduce at least some of the developmental defects observed in CCD patients.

## Introduction

Genetic disorders primarily affecting the skeletal system represent a considerable portion of the recognized rare diseases, and there are over 400 different forms of skeletal dysplasia^1^. Among all genetic metabolic bone diseases with “altered osteoclast, osteoblast or osteocyte activity”, CCD is a relatively well-characterized skeletal dysplasia with low bone formation. The spectrum of CCD disease phenotypes is diverse. The most classic features of CCD patients are open fontanels, clavicle hypoplasia, short stature, or other abnormal bone development. Some patients may even have severe generalized osteoporosis^2^. Recent studies have shown that heterozygous mutations in *RUNX2*, a master transcription regulator for skeletogenesis, are mostly related to CCD pathogenesis^2,3^. Mice with germline deletion of *Runx2* (*Runx2*^−/−^) exhibit a complete absence of mineralized bone in the calvaria or long bones, indicating that RUNX2 is required for intramembranous and endochondral bone formation^3,4^. The heterozygous *Runx2* mutation in mice causes phenotypes resembling human CCD, including clavicle hypoplasia, delayed development, ossification of cranial bones, short stature, hypomineralization of the hyoid bone, and sternum^3,5,6^. Currently, patients with CCD are often treated with combined surgical-orthodontic treatment^7^. However, there is no effective *Runx2*-targeted treatment for short-stature defects. A previous report suggested that regulating the molecular metabolism associated with the causative mRNA or protein can overcome a single gene disorder^8^. Thus, we hypothesized that if the *Runx2* mutation cannot be easily corrected during embryonic development, post-transcriptional or post-translational regulation of *Runx2* could be a viable alternative. Several lines of evidence suggest the regulation of *Runx2* mRNA or protein activity and stability via noncoding RNA or m6A modification^9,10^, phosphorylation^11^, acetylation^12^, or ubiquitination. However, it is unclear how these mechanisms, especially miRNAs, govern *Runx2* after its initial commitment to the osteoblast lineage from bone marrow stromal cells and how it is then sustained to drive osteoblast differentiation in vivo.

miRNAs have been recognized as diagnostic and therapeutic biomarkers in multiple bone metabolic diseases, such as postmenopausal osteoporosis and osteoarthritis, due to their effectiveness in various physiological and pathological processes and their regulatory role in the occurrence of diseases^13–15^. Many miRNAs have been reported to directly target *Runx2* during osteoblast differentiation in vitro^16–18^. The m*iR204/211*-*Runx2* axis has also been demonstrated to be critical in maintaining joint tissue homeostasis, while disruption of this axis results in osteoarthritis^15^. Similarly, in vivo, we previously identified the *miR338* cluster as a potential diagnostic and therapeutic target for postmenopausal osteoporosis, and *miR338*^−/−^ mice were less susceptible to osteoporosis following ovariectomy. In the presence of estrogen, the *miR338* cluster and *Runx2* form a positive feedback loop during osteoblast differentiation in vitro^14^. However, the genetic interaction between these two regulators has yet to be confirmed in vivo.

In the present study, we generated *miR338*^−/−^; *Runx2*^+/−^ mice and found that the skeletal defect induced by *Runx2* haploinsufficiency can be rescued to varying degrees depending on the ossification pattern, which is consistent with our previous in vitro findings. Single-cell transcriptomic analysis revealed that the ablation of *miR338* can restore the osteoblast lineage priming ability of bone marrow stromal cells, which was further confirmed by the conditional knockdown of the *miR338* cluster in the preosteoblast lineage. Furthermore, we found that the *Hif1a*-*Vegfa* pathway can be directly rescued with ablation of the *miR338* cluster in *Runx2*^+/−^ femurs. These results collectively emphasize the role of *miR338* during osteoblast differentiation and suggest that the *miR338* cluster could be a potential therapeutic target for CCD.

## Materials and methods

### Generation and maintenance of transgenic mice

*miR338* conditional knockout (*miR338*^*fl/fl*^, this study) mice and *miR338* knockout (*miR338* ^*−/−*^)^14^ mice were generated in the Laboratory Animal Center, Hangzhou Normal University. Briefly, for *miR338*^*fl/fl*^ mice, using the CRISPR‒Cas9-mediated knock-in approach, donor DNA with the *miR338* precursor flanked by two LoxP sequences and two gRNAs^14^ + Cas9 was used for microinjection (Supplementary Fig. 18b). Heterozygous *miR338* ^fl/+^ mice were outcrossed with the wild-type C57BL/6 mice for three generations to reduce the off-target effects caused by CRISPR. B6.Cg-Tg(Sp7-tTA, tetO-EGFP/cre)1Amc/J (hereafter OsxCre) mice were purchased from Jackson Laboratory. *Runx2*^*+/−*^ mice were provided by Professor Laurie Glimcher’s laboratory. C57BL/6 mice employed for the BMSC in vitro study were purchased from Shulaibao (Wuhan) Biotechnology Co., Ltd. Genotyping were performed using the primers listed in Supplementary Table 7. All mice used in the present study were maintained under specific pathogen-free conditions in the animal center attached to Wuhan University School and Hospital of Stomatology under the guidelines and approval of the Institutional Animal Care and Use Committees at the School and Hospital of Stomatology attached to Wuhan University (protocol no. S07921060I).

### ^18^F-NaF Micro-PET/CT analysis

The bone metabolic rate of 4-week-old female mice was determined by ^18^F-NaF micro positron emission computed tomography (PET/CT), with at least three mice in each group. Before PET imaging, the mice were anesthetized with 2% isoflurane, and then, ~250 ± 10 μCi ^18^F-Na was injected by intravenous injection. After 90 min of ^18^F-Na uptake, mice were anesthetized with 2% isoflurane and placed on a scanning bed. PET/CT images were obtained with the static mode for 10 min followed by a CT scan of normal mode by the TransPET Discoverist 180 system (Raycan Technology Co., Ltd., Suzhou, China). The PET images were reconstructed using the three-dimensional (3D) ordered subset expectation maximization (OSEM) method with a voxel size of 0.5 × 0.5 × 0.5 mm^3^. CT images were reconstructed using the FDK algorithm with a 1024 × 1024 × 1024 matrix. Images were displayed with Carimas software (Turku PET Center, Turku, Finland). The mean standardized uptake value (SUV) was calculated using the following formula: mean pixel value with the decay-corrected region-of-interest activity (μCi/kg)/(injected dose [μCi]/weight [kg]). The SUVs of each mouse’s left and right distal femurs were used to compare their bone metabolic differences.

### Single-cell RNA sequencing (scRNA-seq) and analysis

One-month-old wild-type, *miR338*^*−/−*^*;Runx2*^*+/−*^ and *Runx2*^*+/−*^ mice from the same batch were sacrificed by CO_2_ asphyxiation. The femur was dissected from each mouse, and bone marrow cells were flushed according to a previously reported protocol^19^. After being treated with eBioscience™ 1× Red Blood Cell Lysis Buffer (Life Technologies, Carlsbad, CA, USA), the remaining cells were cultured in medium with α-MEM with 20% FBS at 37 °C in a 5% CO_2_ incubator. Nonadherent cells were removed and washed once with prewarmed phosphate-buffered saline (PBS; Gibco-Life Technologies, Grand Island, NY, USA) after three days. After seven days, the cells were washed three times with PBS, treated with 0.25% trypsin (Life Technologies, Carlsbad, CA, USA), and then resuspended in PBS with 20% FBS. Cell suspensions were then strained through a 70-μm Falcon® filter (BD, Franklin Lakes, NJ, USA) and prepared at a concentration of 700–1200 cells/μL using PBS with 20% FBS. Approximately 10,000 cells from each genotype were subjected to downstream scRNA-seq library preparation. Single-cell RNA-seq was performed on the 10× Chromium platform using Chromium Single-Cell 3’ Library and Gel Bead Kit v3 (10x Genomics; Annoroad Genomics, China). Following the quality check, the DNA library was sequenced on the Illumina Novaseq 6000 (Illumina, Annoroad Genomics, China) for 250 Gb sequencing depth. Filtered reads were mapped to the mm10 transcriptome using Cell Ranger v3.0 (10x Genomics)^20^. Both the Seurat package (v3.0)^21^ and scVelo^22^ (v 0.2.4) were employed in R (v 4.0.3) or Python (v 3.7) for downstream analysis. Briefly, after doublet removal, raw count matrices were filtered to remove barcodes with less than 500 genes expressed, more than 8000 genes expressed, and a high percentage of UMIs from mitochondria (>10%). To eliminate the nonstromal cell type, we filtered out the cells with any expression (count>0) of *Pecam1*, *Ngp*, *Cd19*, *Ptprc*, *Car1*, and *Hbb-bs*^23^. To compare our scRNA-seq profile from monolayer expansion with published results from sorted mouse bone marrow mesenchymal stromal cells (BMSCs)^23^, after the above filtering process, we integrated our profile with other filtered data using Harmony^24^ followed by principal component analysis embedded in the Seurat package. After confirming the similarity between the scRNA-seq in this study and published one, we integrated counts from the remaining cells of different genotypes using the Harmony approach^24^, normalized, and scaled using the SCTransform function^25^. Dimension reduction and clustering were performed and visualized using uniform manifold approximation and projection plots. Marker genes for different clusters were identified using the embedded approach in Seurat. Cell numbers in each cluster were calculated and normalized to the total number of filtered cells in different genotypes. For RNA velocity analysis and comparison, we generated loom files for the spliced and unspliced counts using the outputs from Cell Ranger with scVelo. We then filtered the cells using the cell-count matrix generated from Seurat. We employed the dynamical model in scVelo to solve the full transcriptional dynamics in BMSCs from wild-type, *Runx2*^*+/−*^ and *miR338*^*−/−*^*; Runx2*^*+/−*^ mice and identified the putative driver genes in different clusters imputed from Seurat results. RNA velocity plots of specific genes were also visualized using scVelo.

### RNA sequencing (RNA-seq) and analysis

Femurs from two batches of wild-type, *Runx2*^*+/−*^ and *miR338*^*−/−*^*; Runx2*^*+/−*^ mice were used for BMSC isolation. BMSCs from different individual mice served as biological replicates. BMCSs were induced with or without osteogenic medium for nine days (D0 and D9). RNA extraction was performed using an RNeasy mini kit (Qiagen, Valencia, CA, USA). Genomic DNA was digested using Turbo-DNase I (Promega, San Luis Obispo, CA, USA). Three micrograms of RNA from different biological replicates was used for RNA-seq library preparation. An NEBNext Ultra RNA Library Prep Kit (New England Biolabs, Ipswich, MA, USA) was used to generate and index RNA-seq libraries. The library quality was assessed using a DNA 1000 kit (5067–1504; Agilent) on a Bioanalyzer 2100 (Agilent, Santa Clara, CA, USA). Then, 150-bp paired-end sequencing was performed using a HiSeq X Ten sequencer (Illumina, San Diego, CA, USA, provided by Annoroad Genomics Company (China)). Then, sequencing reads were pseudoaligned to the mm10 mouse genome using Kallisto (v 0.46.2). Sequencing data were pseudoaligned to the mm9 genome and quantified using Kallisto (v.0.43.0)^26^, followed by significantly changed gene identification using the Sleuth R package^27^ with a cutoff of *p* < 0.05. The relative gene expression level for the most abundant transcript of each gene is presented in TPM (transcript per kilobase million). Hierarchical clustering was performed with significantly changed genes on D0 or D9 concatenated to visualize the pattern of differentially expressed genes among the three different genotypes. For a specific cluster of genes, GO enrichment assays were performed using Metascape (http://metascape.org)^28^. We also employed a gene set enrichment assay (GSEA) (v 4.0)^29^ to compare the transcriptome of induced BMSCs from the *Runx2*^*+/−*^ and *miR338*^*−/−*^*; Runx2*^*+/−*^ groups using the driver genes in Cluster 7 from wild-type BMSC scRNA-seq as a gene set. The GSEA result was visualized in R with a custom script.

### Statistical analysis

All data in this study are presented as the mean ± SEM. Differences between individual groups were analyzed by unpaired *t* test in R (v. 4.0.4). The sample size in different experiments is indicated in the text.

## Results

### Global knockout of the *miR338* cluster completely rescued the bone defect in the femur caused by *Runx2* haploinsufficiency

**Fig. 1:**
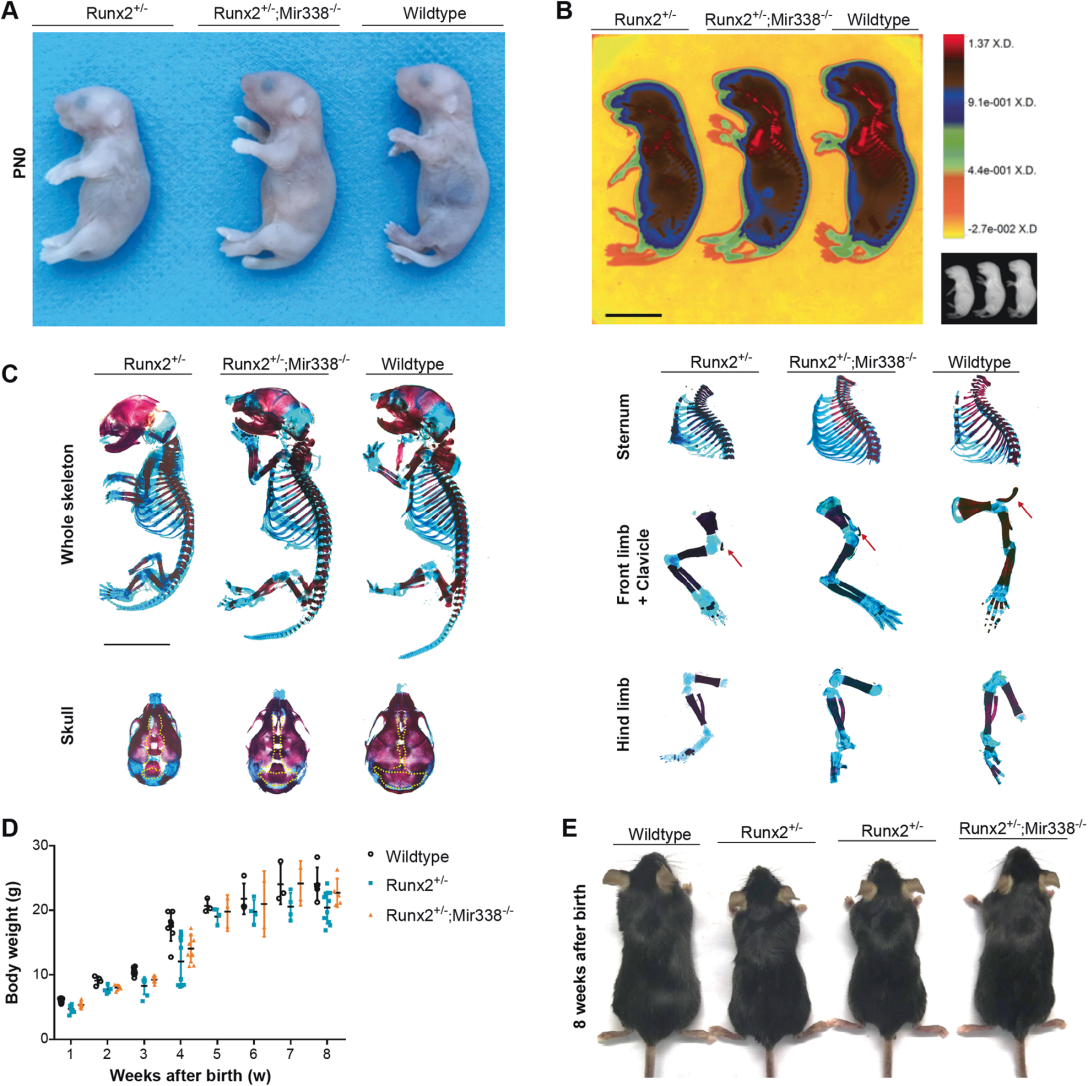
Knockout of miR338 rescued the body length induced by *Runx2* haploinsufficiency. **A** Representative gross images of postnatal Day 0 (PN0) *Runx2*^*+/−*^, *Runx2*^+/−^; *miR338*^−/−^ and wild-type mice from the same batch. **B** Heatmap of X-ray analysis for mice in a, with scale = 1 cm. **C** Whole-mount Alizarin red/Alcian blue staining of the whole skeleton, skull, sternum, front limb+clavicle, and hind limb dissected from *Runx2*^*+/−*^, *Runx2*^+/−^; *miR338*^−/−^ and wild-type mice at PN0. **D** Bodyweight of *Runx2*^*+/−*^, *Runx2*^+/−^; *miR338*^−/−^ and wild-type mice from 1 to 8 weeks after birth. Each dot indicates one individual mouse. **E** Representative gross images of female *Runx2*^*+/−*^, *Runx2*^+/−^; *miR338*^−/−^ and wild-type mice eight weeks after birth.

In our previous study, we found that both *miR-3065-5p* and *miR-338-3p*, members of the *miR338* cluster, may suppress *Runx2* expression in BMSCs^18^ and odontoblasts^30^. However, in the presence of estrogen, *RUNX2* can repress both of their transcriptional levels, promoting osteoblastic differentiation of BMSCs in vitro^14^. However, little is known about their interaction in vivo. Thus, we first confirmed that the half-life or *Runx2* mRNA in the BMSCs isolated from *miR338*^−/−^ (*miR338*-KO) femurs was longer than those from wild-type and *Runx2*^+/−^ (*Runx2*-Het) femurs (Supplementary Fig. 1a). In the limbs of the *Runx2*^+/−^, *Runx2*^−/−^ mice and their wild-type littermates, we compared the expression levels of the two members in the *miR338* cluster. When the *Runx2*^−/−^ limb was compared to the *Runx2*^+/−^ and wild-type limbs, qRT‒PCR analysis showed a significant increase in the *miR338* cluster, while the latter two showed no notable difference (Supplementary Fig. 1b). These results prompted us to hypothesize that knockout of the *miR338* cluster can restore *Runx2* expression levels and rescue the cleidocranial dysplasia induced by *Runx2* haploinsufficiency. Thus, we bred *miR338*-KO mice with *Runx2*-Het mice. Consistent with our previous findings, newborn, 4-week-old, and 8-week-old *miR338*-KO mice exhibited the same body length as the wild-type controls (Supplementary Fig. 2a). The *miR338*^−/−^; *Runx2*^+/−^ (DoubleMutant) mice had almost equal body size to their wild-type littermates, but the *Runx2*-Het mice were significantly smaller (Fig. 1A, and Supplementary Fig. 2a). X-ray (Fig. 1B and Supplementary Fig. 3) and skeletal staining (Fig. 1C) confirmed that the global knockout of *miR338* completely restored the bone size and mineralization in the femur, tibia, limbs, and skull. However, dysplasia of the clavicle was not fully rescued in the *Runx2*-Het mice (Fig. 1C, Supplementary Fig. 2c and Supplementary Fig. 3). Given the different ossification processes in clavicles and femurs, these results indicated that *miR338* ablation could probably rescue endochondral ossification but had less of an effect on the *Runx2*-Het mouse defects in intramembranous ossification. We also observed that for the DoubleMutant cross, no mice with the *Runx2*^−/−^ genotype were observed, indicating that global knockout of the *miR338* cluster did not rescue the lethality phenotype of *Runx2* homozygotes. Thus, we chose the DoubleMutant mice for further phenotypic characterization.

To further confirm whether the knockout of the *miR338* cluster can rescue the postnatal development defect of *Runx2*-Het, we tracked the bodyweight of the DoubleMutant and *Runx2*-Het mice and their wild-type littermates. The results showed no significant difference between the DoubleMutant mice and their wild-type littermates; however, the *Runx2*-Het mice were significantly lighter than the mice of the other two genotypes, as we expected (Fig. 1D). Body lengths showed the same difference (Fig. 1E). Previously, we found that *Runx2* and *miR338* cluster epistasis was estrogen-dependent in vitro, but we found no difference in the rescue effect between male and female mice (Supplementary Fig. 2b and Supplementary Fig. 4).

**Fig. 2:**
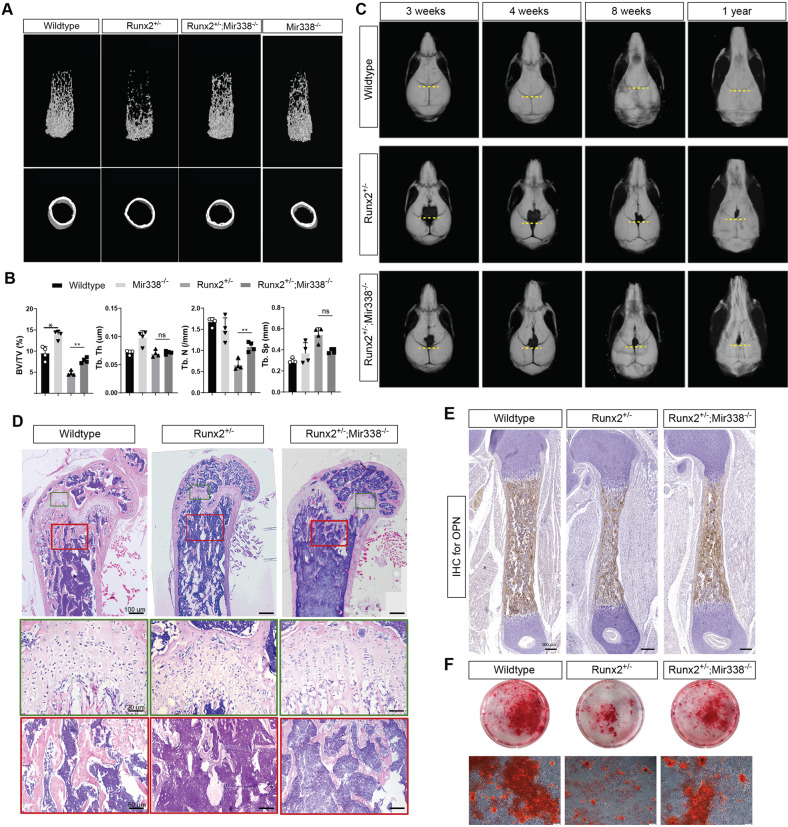
Knockout of miR-338 fully rescued the bone defect caused by Runx2 haploinsufficiency. **A** Micro-CT scans and quantification of 8-week-old *Runx2*^*+/−*^, *miR338*^−/−^, *Runx2*^+/−^; *miR338*^−/−^ and wild-type mouse femurs. **B** Quantification data indicate bone volume/tissue volume (BV/TV), trabecular number (Tb.N), trabecular thickness (Tb.Th) and trabecular separation (Tb.Sp). Each dot indicates one individual mouse tested. An unpaired *t* test was performed. *P < 0.05, **P < 0.01, ***P < 0.001. **C** Micro-CT scans and quantification of 3-week-old, 4-week-old, 8-week-old, and 1-year-old *Runx2*^*+/−*^, *Runx2*^+/−^; *miR338*^−/−^ and wild-type femurs. The yellow dashed line indicates the separation from sagittal (SAG) and posterior frontal (PF) sutures on the skull. **D** H&E staining of femurs from 8-week-old *Runx2*^*+/−*^, *Runx2*^+/−^; *miR338*^−/−^ and wild-type mice. Scale = 200 µm in the upper panel, 20 µm in the middle, and 50 µm in the lower panel. **E** Immunohistochemistry staining for osteopontin (OPN) in sections of femurs from PN0 *Runx2*^*+/−*^, *Runx2*^+/−^; *miR338*^−/−^ and wild-type mice. Scale = 200 µm. **F** BMSCs from 8-week-old *Runx2*^*+/−*^, *Runx2*^+/−^; *miR338*^−/−^ and wild-type mice were subjected to Alizarin Red staining on Day 14 (*n* = 3).

Given that the developmental defects in CCD patients are largely due to the critical biological role of *Runx2* in bone development^3^, we characterized the femurs and skulls of the *Runx2*-Het and DoubleMutant mice. To determine the microarchitecture of the skeleton and the cortical and trabecular bone parameters of the femurs, we used microcomputed tomography (μCT) (Fig. 2A). We found that cortical bone thickness was significantly lower in the *Runx2*-Het mice, but there was no significant difference between the DoubleMutant mice and their wild-type siblings. Similar rescue effects were also observed in total tissue volume (BV/TV), trabecular bone number (Tb.N), and trabecular separation (Tb. Sp) (Fig. 2B). However, there was no significant difference in trabecular thickness among the three genotypes (Fig. 2B). Interestingly, we found that knockout of the *miR338* cluster partially but remarkably rescued the suture closure defect induced by *Runx2* haploinsufficiency. The sagittal (SAG) sutures in the DoubleMutant and wild-type littermates were “closed” three weeks after birth, whereas the *Runx2*-Het mice had an apparent delayed closure defect for SAG. However, the closure defect in the posterior frontal (PF) suture closure defect remained in both the DoubleMutant and *Runx2*-Het mice, although the defect was substantially smaller in the DoubleMutant mice than the *Runx2*-Het mice before eight weeks after birth. The closure defect was still visible even in the one-year-old Runx2-Het and DoubleMutant mice (Fig. 2C and Supplementary Fig. 5). Previously, *Runx2* was reported to regulate cranial suture closure partially by regulating the FGF pathway^31^, and *Fgfr2* is a direct target of the *miR338* cluster^18^. Thus, we assessed the expression levels of *Fgfr2* and *Fgfr3* in PF and SAG sutures on PN7, when FGF signaling is activated. Consistent with previous work, *Fgfr2* and *Fgfr3* in both PF and SAG sutures were significantly inhibited in the *Runx2*-Het mice. Ablation of the *miR338* cluster significantly rescued *Fgfr2* and *Fgfr3* in the SAG suture (Supplementary Fig. 6a) but not in the PF suture (Supplementary Fig. 6a). Moreover, we found that ablation of the *miR338* cluster itself resulted in delayed development in the PF suture compared with that of the wild-type siblings on PN7, with fewer condensed chondrocytes (Supplementary Fig. 6b). Considering the different embryonic lineages contributing to PF and SAG suture, this result reflects a heterogeneous contribution of the *miR338* cluster during suture closure.

We then focused on fully rescuing the femur defect with further detailed analysis. H&E staining confirmed that the growth plate thickness, trabecular bone number, and width of cortical bone were rescued in the DoubleMutant mice (Fig. 2D). Immunohistochemistry staining revealed that osteopontin expression was significantly restored in the DoubleMutant mice compared with their *Runx2*-Het littermates (Fig. 2E and Supplementary Fig. 7). We then isolated BMSCs from the femurs of 8-week-old wild-type, *Runx2*-Het, and DoubleMutant mice, which had been subjected to nine days of osteoblastic induction. Consistent with the in vivo results, Alizarin red staining showed that the BMSCs from the wild-type and DoubleMutant mice generated significantly more mineralization nodules than those of the *Runx2*-Het mice (Fig. 2F). On Day 9 post-induction, qRT‒PCR analysis revealed that the downregulation of *miR-338-3p* in the DoubleMutant BMSCs almost completely rescued *Ocn*, *Alp*, *Runx2*, *Osx*, and *Opn* expression (Supplementary Fig. 8). In vivo, we did not find any significant changes of SOX2 and POSTN in the femurs from Runx2-Het and DoubleMutant (Supplementary Fig. 10). Taken together, these results suggest that global knockout of the *miR338* cluster may restore pre- and postnatal developmental defects in *Runx2* heterozygotes by a complete rescue of the endochondral ossification defect and a partial rescue of the intramembranous ossification defect.

### In *Runx2* mutant mice, ablation of the *miR338* cluster rescued the bone defect without altering osteoclast activity

The balance between osteoblast and osteoclast differentiation is required for bone formation and remodeling^32^. Despite the fact that *Runx2* is not produced in osteoclasts, it is essential for the differentiation of both osteoblasts^2,3^ and osteoclasts^33,34^. However, we observed that loss of the *miR338* cluster resulted in a mild increase in osteoblast differentiation and a significant decrease in osteoclast differentiation in vivo^14^. We wanted to determine whether knockout of the *miR-338* cluster could rescue bone defects made by both osteoclasts and osteoblasts. For this reason, we performed tartrate-resistant acid phosphatase staining of femur sections from 4-week-old *Mir338*-KO, *Runx2*-Het, DoubleMutant, and wild-type littermates. Statistical analysis revealed that the number of active osteoclasts in the femurs of the *Runx2*-Het and DoubleMutant mice did not differ significantly (Supplementary Fig. 9).

### Knockout of *miR338* restored osteoblastic lineage priming of BMSCs in *Runx2* heterozygous mice partially by directly upregulating *Hif1a*

**Fig. 3:**
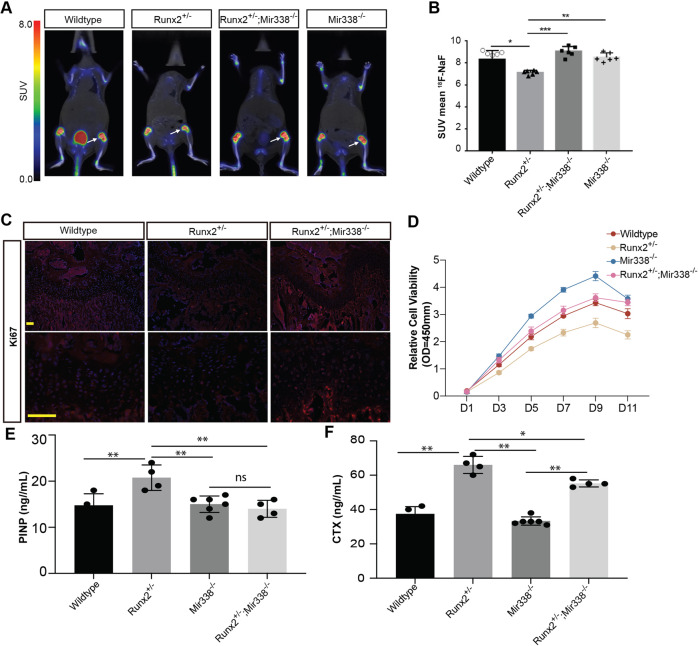
Knockout of miR338 restored the proliferative ability of BMSCs in Runx2^+/−^ femurs. **A** Representative 18F-NaF micro-PET/CT images of femurs from 8-week-old *miR338*^−/−^, *Runx2*^*+/−*^, *Runx2*^+/−^; *miR338*^−/−^ and wild-type mice and quantification **B**. Each dot indicates one femur analyzed (six femurs from three independent mice with the same genotype). An unpaired *t* test was performed. *P < 0.05, **P < 0.01, ***P < 0.001. **C** Ki67 staining of femurs from 8-week-old *Runx2*^*+/−*^, *Runx2*^+/−^; *miR338*^−/−^ and wild-type mice. DAPI staining indicated the nuclei. **D** CCK-8 assay comparing the viability of BMSCs isolated from *miR338*^−/−^, *Runx2*^*+/−*^, *Runx2*^+/−^; *miR338*^−/−^ and wild-type mice. *n* = 3, unpaired *t* test was performed, **P < 0.01, ***P < 0.001. **E** Measurement of serum PINP from 4-week-old female mice. **F** Measurement of serum CTX from 4-week-old female mice. *P < 0.05, **P < 0.01, ns: not significant.

Based on the above results, we hypothesized that the knockout of *miR338* restores bone defects without affecting osteoclast activity. We then assumed that this was due to cell proliferation in the femur. We first scanned 4-week-old *Runx2*-Het, DoubleMutant, *Mir338*-KO, and wild-type littermates employing 18F-NaF microPET/CT (Fig. 3A), which is a marker for new bone formation^35^. The results revealed that the new bone formation in the *Runx2*-Het femur was the lowest, but the formation in the DoubleMutant femur was comparable to that of the wild-type littermates (Fig. 3B). MCM2, a cell proliferation marker, was substantially higher in the DoubleMutant femurs than in the *Runx2*-Het femurs (Supplementary Fig. 10). Ki67 staining in the growth plate region showed that the overall cell proliferation rate near the Runx2-Het growth plate was the lowest among the three genotypes (Fig. 3C). In vitro, CCK-8 analysis showed that BMSCs isolated from the *miR338*-KO mice had the greatest potential to proliferate among the four genotypes. BMSCs from the *Runx2*-Het femurs had the lowest proliferation, while BMSCs from wild-type and DoubleMutant femurs had similar proliferation levels (Fig. 3D). These results suggest that depletion of *miR338* could rescue the proliferation of cells in femurs.

We then analyzed the concentration of serum markers for bone turnover. In 4-week-old mice, circulating levels of the N-terminal propeptide of type I procollagen, a marker of bone formation^36^, were significantly elevated in the Runx2-Het mice but downregulated in DoubleMutant mice (Fig. 3E). However, the serum levels of the C-terminal telopeptide of type I collagen (CTX), a marker of bone resorption activity^36^, were elevated in both the Runx2-Het and DoubleMutant mice compared with their wild-type siblings. We also found that CTX in the DoubleMutant serum was still lower than that in the Runx2-Het mice (Fig. 3F). All the above results showed that ablation of the *miR338* cluster rescued bone formation defects caused by Runx2 haploinsufficiency.

**Fig. 4:**
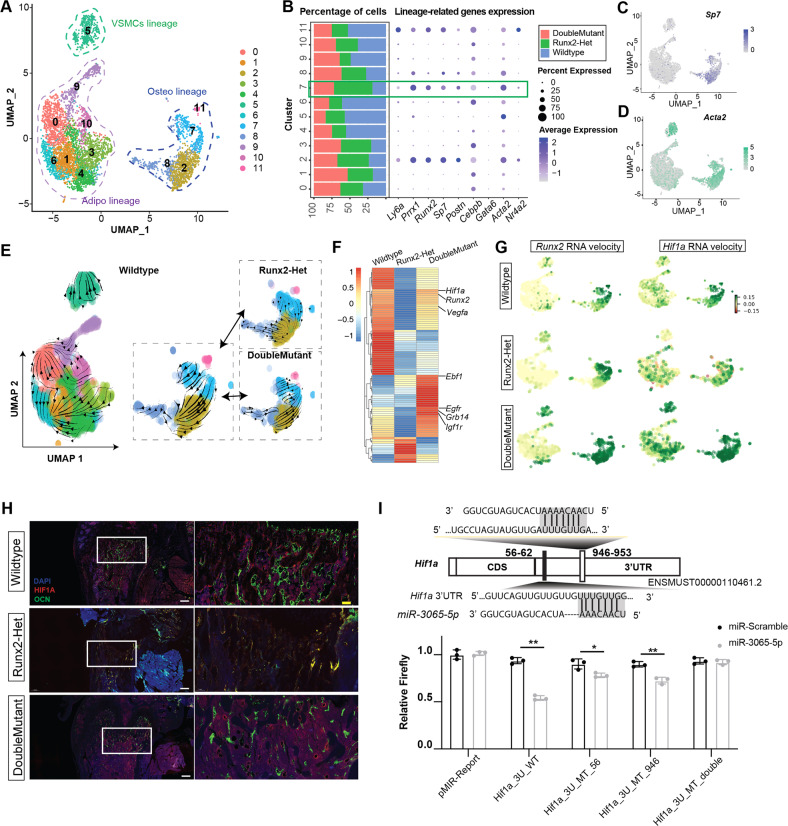
Single-cell RNA-seq of BMSCs revealed that knockout of *miR338* restored osteoblast lineage priming ability by directly upregulating HIF1A expression. **A** A uniform manifold approximation and projection (UMAP) plot depicting the clustering of single-cell transcriptional profiles for 5562 filtered BMSCs isolated from 4-week-old *Runx2*^*+/−*^ (Runx2-Het), *Runx2*^+/−^; *miR338*^−/−^ (DoubleMutant) and wild-type mouse femurs. Partition mainly related to VSMCs, adipocyte (Adipo) or osteoblast (Osteo) lineage was outlined with a dashed line with different colors. **B** Dot plot showing the expression and enrichment of selected top genes identified in each cluster. The size of the dot indicates the percentage of cells per cluster. Bar charts show the percentage of cell numbers in different clusters normalized to the total number of BMSCs from different genotypes. The green box indicates that there are more cells enriched in Cluster 7 from Runx2-Het BMSCs compared with other genotypes. **C** Feature plot for *Sp7* as a marker gene for (pre)osteoblasts in all filtered cells. **D** Feature plot for *Acta2* as a marker for (pre)VSMCs in all filtered cells. **E** The RNA velocity plot in UMAP indicates the dynamic transcriptome changes in all filtered BMSCs from wild-type cells and cells from Runx2-Het and DoubleMutant in Clusters 2, 7, 8, and 11. The direction of RNA velocity in Clusters 7 to 2 of Runx2-Het was visually different compared with the other two genotypes. **F** Heatmap showing the average expression of the top driver genes in wild-type Cluster 7 in bulk RNA-seq for D0 BMSCs from wild-type, Runx2-Het, and DoubleMutant femurs. Of the indicated genes in the heatmap, *Hif1a*, *Runx2*, *Egfr*, and *Igf1r* were predicted to be the direct targets of the *miR338* cluster by TargetScan (v. 7.0). **G** RNA velocity portrait for *Runx2* and *Hif1a* in the scRNA-seq profiles for BMSCs from wild-type, Runx2-Het, and DoubleMutant mice. Positive velocity (in green) indicates that the gene is upregulated, with a higher abundance of unspliced mRNA for that gene than expected. Negative velocity (in red) indicates that the gene is downregulated. **H** Immunofluorescence for HIF1A (in red) and OCN (in green) in 8-week-old mouse femurs with different genotypes. **i** Dual-luciferase assay validating the putative binding site of miR-3065-5p in the 3′UTR of *Hif1a* (ENST00000323441.6). Each dot indicates a different replicate. An unpaired t test was performed. *P < 0.05, **P < 0.01.

Therefore, since BMSCs are the main source for bone formation, we employed scRNA-seq to profile the transcriptome hierarchies of BMSCs with different genotypes. We isolated BMSCs using a short-term in vitro culture (seven days)^19^, and 10,000 cells from each genotype were subjected to scRNA-seq. After filtering of doublets and cells with low-quality gene expression or high mitochondrial genes, the wild-type, *Runx2*-Het and DoubleMutant groups were left with 8999, 5282, and 4740 cells, respectively. To avoid possible contamination of the nonstromal cell type, we removed cells with expression (count >0) of *Pecam* (for endothelial lineage), *Ptprc* (for leukocytes), *Ly76* (for lymphocytes), *Ngp* (for granulocytes), *Cd19* (for lymphoid lineage), *Car1* (for early erythoid lineage) or *Hbb-bs* (for late erythoid lineage)^23^. After two rounds of filtering, wild-type, *Runx2*-Het, and DoubleMutant BMSCs were left with 2640, 1301, and 1621 cells, respectively. To strengthen our confidence, we compared our profiles with previously reported scRNA-seq profiles from FACS-isolated BMSCs. Principal component analysis with downstream clustering revealed that our approach’s profile was similar to the published profile (Supplementary Fig. 11). Integrated profiles of BMSCs with three genotypes showed that most of the BMSCs expressed more than one lineage priming marker, which is consistent with a previous report^37^, although there were still three significant partitions with several cluster depending on signature genes (Fig. 4A, Supplementary Fig. 12 and Supplementary Table 1). For example, cells in Clusters 2, 7, 8, and 11 primarily expressed *Sp7* (Fig. 4C), *Runx2*, and *Postn*, which are markers for the “osteoblast/chondrocyte lineage”. Cells in Clusters 0, 1, 3, 4, 6, 9, and 10 expressed *Cebpb* and *Ccl7*, which are markers for the adipocyte lineage. Cells in Cluster 5 expressed *Acta2*, a marker for vascular smooth muscle cells (VSMCs) (Fig. 4D). We also observed that cells from the osteoblast/chondrocyte lineage expressed *Acta2* and *Prrx1*, which are markers for mesenchymal stem cells. *Postn* and *Runx2* were also expressed by some cells in the “adipocyte lineage” (Supplementary Fig. 13b, c).

We then analyzed the subclusters in each lineage. Overall, there were unique subclusters of cycling cells in each lineage expressing both *Mki67* and lineage marker genes. Additionally, we found that genes that promote lineage differentiation were widely expressed within each lineage, but markers for mature osteoblasts, adipocytes or VSMCs were barely detected. *Sp7* was widely expressed in all subclusters of the osteo lineage (Supplementary Fig. 14a–d), but *Ocn*, a marker for mature osteoblasts, could be detected only in a small proportion of cells (Supplementary Fig. 14d). Similarly, *Plin2* and *Adipor1* were widely expressed in the adipogenic lineage, but *Ucp1*, a gene for thermogenesis in adipocytes, was barely detected (Supplementary Fig. 16). *Acta2*, *Cald1,* and *Col1a2*, markers for contractile and synthetic VSMCs, were widely expressed in the VSMC lineage (Supplementary Fig. 17). We then focused our assays on the osteo lineage. Marker gene identification (Supplementary Fig. 15a–c) along with pseudotime trajectory analysis (Supplementary Fig. 15d, e) for the osteo lineage indicated that (1) O3, with enrichment of *Mki67* and *Top2a*, was a cycling cell population; (2) O1, with enrichment of *Runx2*+, *Sp7*+, and *Ocn*+ cells, was an osteoblast population; and (3) O2, with specific enrichment of *Mgp*^38^, *Rspo2*^39^, and *Id4*^40^, which have been shown to exert a positive effect on osteoblast differentiation, was an intermediate population from cycling cells toward osteoblasts.

Overall, the (sub)clustering patterns of the wild-type, *Runx2*-Het, and DoubleMutant BMSCs were quite comparable (Supplementary Fig. 13a). However, when we normalized the total number of cells in each genotype, we observed that Cluster 7 had more *Runx2*-Het cells than wild-type and DoubleMutant cells (Fig. 4B). Interestingly, RNA velocity analysis comparing spliced and unspliced forms of transcripts revealed that the transcriptional dynamics differed among the three genotypes. In the wild-type (wt-cluster-7) and DoubleMutant (DM-cluster-7) profiles, we observed a continuous transcriptomic dynamic transition from Cluster 7 toward Cluster 2, but this dynamic was “stopped” in Cluster 7 in the *Runx2*-Het profile (Het-cluster-7) (Fig. 4E). Similarly, in the detailed analysis of the RNA velocity of the osteo lineage, the trajectory from O3 toward the O2 and O1 clusters of the Runx2-Het group was reversed compared with that of the wild-type or DoubleMutant group (Supplementary Fig. 14e). These cumulative results indicated that Runx2 haploinsufficiency led to incomplete osteoblast lineage priming but could be recovered by the ablation of the miR338 cluster. We subsequently used bulk RNA-seq for BMSCs from wild-type, *Runx2*-Het, and DoubleMutant femurs to verify the average expression of the driver genes for wt-cluster-7 (Supplementary Tables 2–4). The RNA-seq results showed that in the DoubleMut mutant, the driver genes in wt-cluster-7, such as *Hif1a*, *Runx2*, and *Igfr1*, were significantly rescued (Fig. 4F). Most of these genes were found only in the driver gene lists of wt-cluster-7 and DM-cluster-7 (Supplementary Tables 2–4) and are related to osteoblast differentiation, indicating that knockout of *miR338* restores the osteoblast lineage priming ability of BMSCs. RNA velocity portraits showed that the transcriptional dynamics of *Runx2* and Hif1a in Cluster 7 cells of the wild-type and DoubleMutant groups were higher than those in *Runx2*-Het (Fig. 4G). Similarly, the RNA velocity for Hif1a was rescued in the O1 subcluster in the osteoblastic lineage (Supplementary Fig. 14f). Among these driver genes, *Hif1a* was predicted as a target for *miR-3065-5p* by TargetScan Mouse (v.7.1) (http://www.targetscan.org/mmu_71/). We further validated that the expression of HIF1A in the DoubleMutant and wild-type femurs was comparable and much higher than that in the *Runx2*-Het femur (Fig. 4H). Furthermore, we predicted two potential direct binding sites for *miR-3065-5p* in the 3′UTR of Hif1a using TargetScan. We further confirmed that these are essential for maintaining the transcriptional stability of *Hif1a* (ENSMUST00000110461.2) (Fig. 4I). Overall, we identified that knockout of *miR338* could partially rescue the osteoblast lineage priming ability of BMSCs by directly upregulating *Hif1a* expression using RNA velocity analysis comparing scRNA-seq profiles from *Runx2*-Het, DoubleMutant and wild-type femurs.

### *miR338* deficiency in preosteoblasts (*Osx* + lineage) partially rescued *Runx2* haploinsufficiency-induced bone defects

**Fig. 5:**
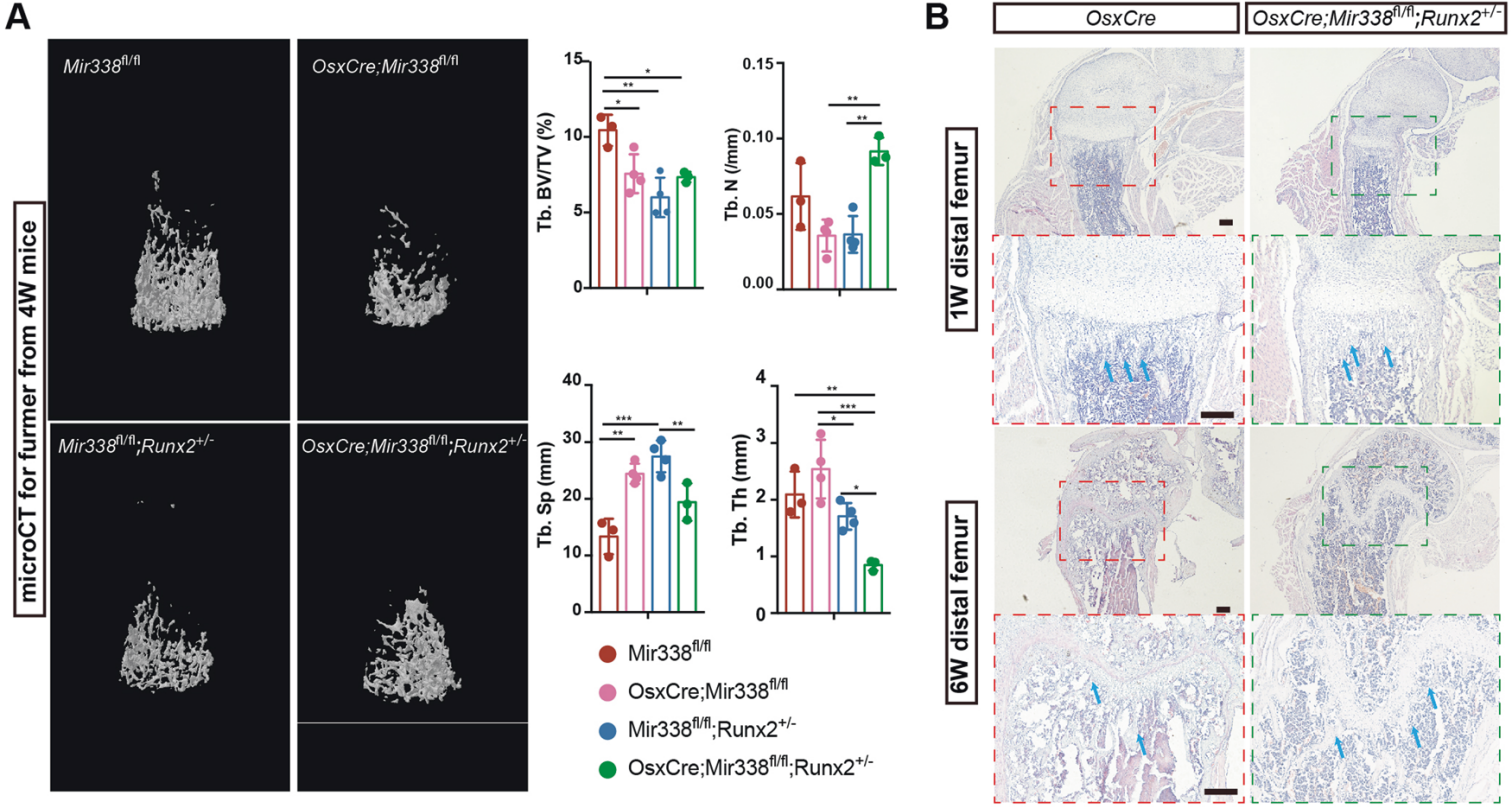
Specific ablation of the *miR338* osteoblast lineage (*Osx* + lineage) partially rescued bone defects caused by the OsxCre transgene and *Runx2* haploinsufficiency. **A** Representative image for micro-CT scans and quantification of 4-week-old *miR338*^*fl/fl*^, *OsxCre; miR338*^*fl/fl*^, *miR338*^*fl/fl*^*; Runx2*^*+/−*^, and *OsxCre; miR338*^*fl/fl*^*; Runx2*^*+/−*^ mouse femurs, and quantification data indicate bone volume/tissue volume (BV/TV). Each dot represents one individual. An unpaired *t* test was performed. Data are presented as the means ± SEMs. *P < 0.05, **P < 0.01, ***P < 0.001NS: not significant. **B** H&E staining of a section of femurs from 1-week-old and 6-week-old OsxCre and *OsxCre; miR338*^*fl/fl*^*; Runx2*^*+/−* mice^ from the same batch. Blue arrows indicate the trabecular bone. Scale = 200 µm.

Based on the abovementioned results, we concluded that knockout of the *miR338* cluster mainly rescued the Runx2-Het BMSC osteoblastic lineage priming ability. We then investigated whether specific ablation of miR338 in the preosteoblast lineage could rescue the bone defect. Thus, we first generated a mouse model with conditional knockout (cKO) alleles for the *miR338* cluster using the CRISPR‒Cas9-mediated knock-in technique (Supplementary Fig. 18a–c). Although the LoxP sites flanking the *miR338* cluster resided in the promoter regions of the *miR338* cluster, they had no effect on the expression levels of *miR-338-3p*, *miR-3065-5p*, and *Aatk* in the brain (Supplementary Fig. 18d), where *miR338* had the highest expression^14^. We then generated OsxCre; *miR338*^fl/fl^ mice by breeding *miR338*-cKO mice with OsxCre transgenic mice. We isolated BMSCs from OsxCre; *miR338*^fl/fl^ mice and OsxCre; *miR338*^fl/-^ littermates and used osteoblastic medium to induce expression. We found considerable downregulation of miR-338-3p and miR-3065-5p on day five after induction compared to the control (Supplementary Fig. 18e). Finally, we devised two breeding strategies to obtain OsxCre; *miR338*^fl/fl^; *Runx2*^+/−^ mice (Supplementary Fig. 11a, b). Unexpectedly, we failed to obtain OsxCre; *Runx2*^+/−^ mice, which were meant to be the best control for phenotypic analysis of OsxCre; *miR338*^fl/fl^; *Runx2*^+/−^ mice in more than 1000 newborn mice. Based on the recent genotyping data (Supplementary Fig. 19a, b), we found that mice carrying either the *OsxCre* or *Runx2*^-^ allele had a lower birth ratio than expected in both breeding strategies (Supplementary Fig. 19a, b). Mice having both these alleles had an even lower birth ratio, with no OsxCre; Runx2^+/−^ mice being born. We sacrificed some of the pregnant mice and found that most of the dead embryos had the OsxCre; *Runx2*^+/−^ allele (example in Supplementary Fig. 19c, d). However, some OsxCre; *miR338*^fl/fl^; *Runx2*^+/−^ mice were viable and could survive up to a month after birth. We performed micro-CT analysis to examine the femurs of one-month-old *miR338*^fl/fl^, *miR338*^fl/fl^; *Runx2*^+/−^, OsxCre; *miR338*^fl/fl^, and OsxCre; *miR338*^fl/fl^; *Runx2*^+/−^ mice and found that more but thinner trabecular bone was formed in the OsxCre; *miR338*^fl/fl^; *Runx2*^+/−^ mouse distal femurs than in *Mir338*^fl/fl^; *Runx2*^+/−^ and OsxCre; *miR338*^fl/fl^ femurs (Fig. 5A), which was confirmed by H&E staining of 2-week-old mouse femurs (Fig. 5B). Consistent with previous results, we observed that mice with the OsxCre or *Runx2*^-^ allele had lower bone volume (Tb.BV/TV) than the controls (*miR338*^fl/fl^). However, the bone volume in OsxCre; *miR338*^fl/fl^; *Runx2*^+/−^ mouse femurs was rescued. Of note, OsxCre; *miR338*^fl/fl^ mice exhibited thick growth plates and more trabecular bone than OsxCre mice at 1 week and 6 weeks, as shown in the section from distal femurs (Supplementary Fig. 20). All these phenotypic analyses confirmed that specific ablation of *miR338* in the preosteoblast lineage could promote bone formation and rescue bone defects caused by *Runx2* haploinsufficiency.

### Despite *Runx2* haploinsufficiency, *miR338* ablation was restored during osteoblastic differentiation by the Hif1a-Vegfa axis

**Fig. 6:**
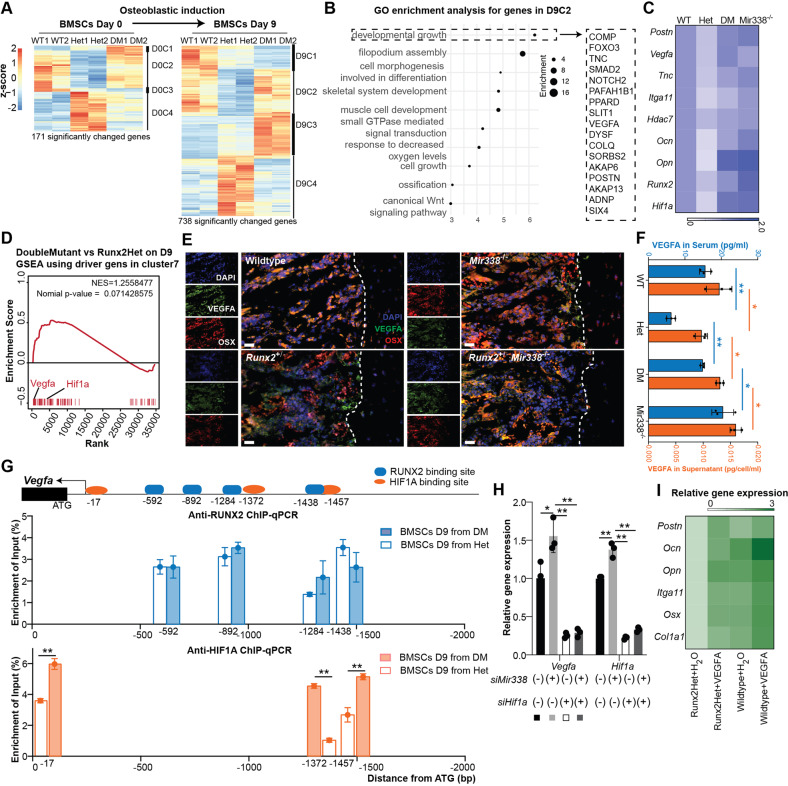
Knockout of miR338 restored VEGFA expression through HIF1A despite Runx2 haploinsufficiency. **A** Hierarchical clustering heatmap for all significantly differentially expressed genes identified by bulk RNA-seq using BMSCs from wild-type (WT), Runx2^+/−^ (Het), and *miR338*^−/−^; Runx2^+/−^ (DM) mice before (Day 0) and after (Day 9). **B** Dot plot for GO enrichment analysis for genes in the D9C2 cluster. Genes in D9C2 also listed in the GO term “developmental growth” are listed on the right. **C** Heatmap showing the qRT‒PCR for selected osteoblast-related genes using BMSCs from different genotypes after osteoblastic induction. The color key indicates the fold change compared with expression in WT. **D** GSEA plot ranking genes between Het and DM on Day 9 using the wild-type Cluster 7 driver gene as a gene set. *Vegfa* ranked at the edge. **E** Immunofluorescence for VEGFA (green) and OSX (red) near the cortical bone region (dashed line) of femurs from mice with the indicated genotype. **F** ELISA showing the secretion of VEGFA from cultured BMSCs with different genotypes or circulating VEGFA collected from serum with different genotypes. Data are presented as the means ± SEMs. An unpaired *t* test within the same sample type (serum in blue and supernatant in orange) was performed. *P < 0.05, **P < 0.01, *n* = 3. **G** ChIP‒qPCR assay of HIF1A and RUNX2 binding sites 2000 upstream of the translational start site (ATG) of *Vegfa*. Data are presented as the means ± SEMs. An unpaired t test was performed to compare the DM and Het groups. *P < 0.05, **P < 0.01. **H** qRT‒PCR validated the epistasis among *miR338*-Hif1a-Vegfa in the MC3T3-E1 cell line using a combination of *miR338* inhibitor and siHif1a under hypoxic conditions (1% O_2_, 5% CO_2_, and 94% nitrogen). **I** Heatmap showing qRT‒PCR validation of osteoblast-related gene expression in BMSCs with different genotypes cultured in osteoblastic induction medium with VEGFA. The color key indicates the fold change compared with BMSCs from Runx2-Het without VEGFA supplementation.

To further examine this result and explore more potential therapeutic targets for CCD, we investigated its underlying molecular mechanisms. First, we performed bulk RNA-seq using BMSCs from three genotypes cultured in osteoblastic induction medium (D9) or regular medium (D0), with two biological replicates for each genotype. The results revealed that 171 genes were significantly changed among the wild-type, Runx2-Het, and DoubleMutant BMSC D0 groups (Supplementary Table 5), and 738 genes were substantially modified in the BMSC D9 group (Supplementary Table 6). Here, we focused on the genes that varied in the D9 group since this in vitro condition was similar to osteoblastic differentiation. Hierarchical clustering revealed that a cluster of genes (D9C2) was rescued in the DoubleMutant group (Fig. 6A). Gene Ontology (GO) enrichment analysis showed that the genes in D9C2 were highly associated with developmental growth, response to decreased oxygen levels, and ossification (Fig. 6B). Then, we chose some of these genes to verify their expression using qRT‒PCR in the BMSC D9 group (Fig. 6C and Supplementary Fig. 21). *Vegfa* and *Hif1a* ranked near the edge of the GSEA ranking genes expressed in both DoubleMutant D9 vs. *Runx2*-Het D9 using the wt-cluster-7 driver genes as a gene set (Fig. 6d). We confirmed that VEGFA was downregulated in *Runx2*-Het cells using immunofluorescence (Fig. 6E). In addition, when BMSCs or serum from *Runx2*-Het mice were compared to other genotypes, VEGFA secretion was reduced (Fig. 6F). In addition to Vegfa, we observed that the DoubleMutant BMSC D9 group had higher expression of *Igf1* and *Vegfc* than the wild-type and *Runx2*-Het groups. The proliferation markers *Mcm2*, *Mcm9* and *Mcm10* were higher in the DoubleMutant and wild-type groups than in *Runx2*-Het group on Day 0 (D0C2), but they were lower in the DoubleMutant and wild-type groups than in the *Runx2*-Het group on Day 9 (D9C4). Unlike *Hif1a* or *Runx2*, *Vegfa* was not a direct target for the *Mir338* cluster, although we observed several binding sites of HIF1A in the promoter region of *Vegfa* (Fig. 6G). ChIP‒qPCR confirmed that both *RUNX2* and HIF1A could directly bind to the promoter of *Vegfa*. Moreover, *RUNX2* and HIF1A could bind to the promoter of *Vegfa* (Supplementary Fig. 22). Interestingly, *RUNX2* enrichment in the *Vegfa* promoter was not significantly different in the *Runx2*-Het and DoubleMutant BMSCs. We observed remarkably higher HIF1A enrichment in the DoubleMutant BMSCs compared to the *Runx2*-Het cells (Fig. 6G). This finding shows that the knockout of *miR338* rescued *Vegfa* expression in a *Runx2* haploinsufficiency-independent manner. Using a combination of inhibitors for miR-338-3p and siRNA targeting Hif1a, we validated *miR338*-Hif1a-Vegfa epistasis in MC3T3-E1, a mouse preosteoblast cell line (Fig. 6H). Finally, using qRT‒PCR, we found that supplementation with VEGFA (10 ng/mL) for osteoblastic induction restored the expression of *Postn*, *Ocn*, *Opn*, *Itga11*, *Osx,* and *Col1a1* in the *Runx2*-Het-derived BMSCs (Fig. 6I).

Taken together, our in vivo and in vitro experiments showed that ablation of miR338 safely and completely rescued bone defects induced by *Runx2* haploinsufficiency mainly by upregulating the *Hif1a*-*Vegfa* axis during osteoblast differentiation.

## Discussion

In the present study, *miR338* was identified as a negative regulatory target of *Runx2* in bone, and the interaction between the *miR338* cluster and *Runx2* was confirmed in vivo during osteoblast differentiation. These findings led us to generate *miR338*^−/−^; *Runx2*^+/−^ (DoubleMutant) mice, which rescued the dwarf phenotype induced by *Runx2* haploinsufficiency. In comparison to the wild-type control, the mutation of *Runx2* in bone decreased osteoclast activity, cell proliferation, and the osteoblast differentiation potential of bone marrow stromal cells. In contrast, bone marrow stromal cells in the DoubleMutant mice had higher proliferation and increased osteoblast lineage priming but no significant effect on osteoclast activity. To confirm this phenomenon, we conditionally ablated the *miR338* cluster and observed that bone density was increased in the *miR338*^fl/fl^; *Runx2*^+/−^ mice. Mechanistically, we found that *Hif1a* is a direct target of the *miR338* cluster, and knockout of miR338 enriched HIF1A binding to the promoter of *Vegfa* during the osteoblast differentiation of *Runx2* mutant bone marrow stromal cells. (Working model summarized in Supplementary Fig. 23) These in vivo results suggested a potential CCD treatment strategy based on post-transcriptional regulation of *Runx2*.

The CCD spectrum is a rare hereditary disease caused mostly by *Runx2* mutations^3,41^. This skeletal dysplasia is characterized by abnormal clavicles (hypoplastic clavicles and aplastic clavicles), short ribs, cervical ribs, patent sutures and fontanels, supernumerary teeth, short stature, and a number of other skeletal changes. In the clinic, management for this disease is mostly symptom‐based treatment, including a surgical-orthodontic approach for craniofacial and dental defects and supplementation with vitamin D and calcium for skeletal defects. In the recent decade, with the accumulated results of genes regulating skeletal development, advances in molecular and genetic biology have provided a new method for dissecting the mechanisms underlying genetic skeletal defects^8^. As such, mechanism-based drug selection could potentially improve the treatment of genetic bone diseases utilizing pharmacological approaches. It is possible to find a precise therapeutic target to compensate for the haploinsufficiency of *Runx2* in CCD based on the transcriptional regulation and pre- and post-translational modification of *Runx2*. For example, NELL1, a key functional mediator of *Runx2* osteogenic activity, rescues calvarial defects in *Runx2*^+/−^ mice^42^. In terms of post-translational modifications, HDAC inhibitors^43^ and direct PIN1 administration^44,45^ increase *RUNX2* acetylation and increase RUNX2 activity, thereby rescuing *Runx2* deficiency in CCD. Nicotinamide, a vitamin B3 and a class III histone deacetylase inhibitor, has been shown to substantially improve delayed tooth eruption in *Runx2*^+/−^ mice by inducing RUNX2 protein and transacting activity post-translationally with *Sirt2* inhibition^46^. We identified that members of the *miR338* cluster can directly target and decrease *Runx2* mRNA stability during osteoblast differentiation. Without any visible side effects, depletion of the *miR338* cluster or direct intravenous injection of miR338 cluster inhibitors significantly prevented osteoporosis after ovariectomy in mice, indicating the safety of *miR338* inhibition treatment^13^. In the present study, we further found that except for rescuing the dwarf phenotype in *Runx2*, knockout of *miR338* had no adverse effects on the daily activity of mice. However, since the ablation of *miR338* itself could mildly promote bone formation, which may compensate for the defect caused by *Runx2* haploinsufficiency, we cannot confirm the interaction between *Runx2* and the *miR338* cluster.

In the bulk RNA-seq profile for osteoblast differentiation, we identified *Vegfa* as one of the most significantly rescued genes during osteoblast differentiation of DoubleMutant BMSCs. Despite *Runx2* haploinsufficiency, transcriptional upregulation of *Vegfa* by knockout of the *miR338* cluster is sufficient, with increased enrichment of HIF1A but not *RUNX2* in the promoter of Vegfa. Moreover, when *Runx2*-Het and wild-type BMSCs cultured in osteoblast induction medium were compared, VEGFA supplementation significantly restored the expression of osteoblast-related markers. These findings suggested that VEGFA could potentially rescue osteoblast differentiation defects caused by *Runx2* mutation, but further in vivo validation is necessary.

Although knockout of *miR338* completely rescued the bone defect, the fontanel sutures of the DoubleMutant mice narrowed faster than those of the *Runx2*-Het mice, and the defect persisted for one year. In comparison to the *Runx2*-Het mice, the DoubleMutant mice had an earlier closure of sagittal suture. Suture closure is more related to intramembranous ossification, and *Runx2* is dispensable for both intramembranous and endochondral ossification. The incomplete rescue for suture closure and clavicle formation may be due to the differential role of *miR338* in intramembranous and endochondral ossification, which warrants further investigation.

## Supplementary information

Supplementary Information

## Data Availability

Raw sequencing data have been deposited to the National Genomics Data Center (https://ngdc.cncb.ac.cn/) with accession no. CRA005957 for all bulk RNA-seq profiles and CRA005954 for all single-cell RNA-seq profiles. Processed datasets and scripts are available upon request. Other detailed Materials and Methods, including histology and tissue preparation, immunohistochemistry and immunofluorescence, cell culture and osteoblastic induction, cell proliferation assays, quantitative reverse transcriptase PCR (qRT‒PCR), dual-luciferase activity assays for the promoter and 3′UTR, enzyme-linked immunosorbent assays (ELISAs), and chromatin immunoprecipitation quantitative PCR, are provided in the Supplementary Information.
