# Supplementary material for: Inhibition of miR338 rescues cleidocranial dysplasia in Runx2 mutant mice partially via the Hif1a-Vegfa axis
Source: Exp Mol Med. 2023 Jan 4;55(1):69–80. doi: 10.1038/s12276-022-00914-w (PMC9898552; doi:10.1038/s12276-022-00914-w)
Supplement: Supplementary file 1 — Supplementary Information [file 12276_2022_914_MOESM1_ESM.pdf]

Title: Inhibition of *miR338* rescues cleidocranial dysplasia in *Runx2* mutant mice partially via the *Hif1a*-*Vegfa* axis

Running title: Inhibition of *miR338* rescues CCD

Runze Jin[1*], Hanshu Zhang[1*], Chujiao Lin[1,2], Jinqiang Guo[1], Weiguo Zou[3], Zhi Chen[1], Huan Liu[1,4,5]

[1]The State Key Laboratory Breeding Base of Basic Science of Stomatology & Key Laboratory for Oral Biomedicine of Ministry of Education, School and Hospital of Stomatology, Wuhan University, 237 Luoyu Road, Wuhan 430079, China

[2]Division of Rheumatology, Department of Medicine, University of Massachusetts Medical School, Worcester, MA 01605, USA

[3]State Key Laboratory of Cell Biology, Shanghai Institute of Biochemistry and Cell Biology, CAS Center for Excellence in Molecular Cell Sciences, Chinese Academy of Sciences,University of Chinese Academy of Sciences, Shanghai, China

[4]Department of Periodontology, School of Stomatology, Wuhan University, Wuhan 430079, China

[5]Taikang Center for Life and Medical Sciences, Wuhan University, Wuhan, China

[*] These authors contributed equally: Runze Jin, Hanshu Zhang

Corresponding authors:

Zhi Chen,
State Key Laboratory Breeding Base of Basic Science of Stomatology (Hubei-MOST) and Key Laboratory for Oral Biomedicine of Ministry of Education (KLOBM), School and Hospital of Stomatology, Wuhan University, Wuhan 430079, China.
Tel: +86-13971037354; Email: zhichen@whu.edu.cn
Huan Liu,
State Key Laboratory Breeding Base of Basic Science of Stomatology (Hubei-MOST) and Key Laboratory for Oral Biomedicine of Ministry of Education (KLOBM), School and Hospital of Stomatology, Wuhan University, Wuhan 430079, China.
Tel: +86-13476211742; Email: liu.huan@whu.edu.cn

Contents for Supplementary Information

Supplementary Materials and Methods……………………………………………………3
Supplementary Figures and Figure Legend……………………………………………..…7
Supplementary Table 1………………………………………………………………..…29
Supplementary Table 2…………………………………………..………………..…393
Supplementary Table 3…………………………………………….……………..…398
Supplementary Table 4……………………………………………………………………402
Supplementary Table 5…………………………………………..………………..…406
Supplementary Table 6……………………………………...…………………….…413
Supplementary Table 7……………………………………………………………….…451

Supplementary Material and Methods

Histology and tissue preparation

All mice with indicated genotypes were sacrificed using CO_2, and then femurs were harvested, skinned, and eviscerated before fixing in 4% PFA at 4 °C overnight. Decalcification was performed in 10% EDTA for 2-4 weeks. Then, samples were subjected to dehydration using a graded ethanol series and paraffin embedding. For paraffin sections, samples were dehydrated in graded ethanol series, cleared in xylene, embedded in paraffin, sectioned at 6-μm thickness with a Leica microtome (Wetzlar, Hesse-Darmstadt, Germany), and mounted on SuperFrost™ Plus slides (Thermo Fisher Scientific, Waltham, MA, USA). The prepared sections were analyzed for H&E staining and other immunostaining. TRAP (Sigma, St Louis, MO, USA) staining was used to quantify a number of osteoclasts with more than three nuclei in six independent visions.

Immunohistochemistry and Immunofluorescence

For immunohistochemistry staining, tissue sections were treated with Pepsin Antigen Retrieval Kit (Maixin, Fuzhou, China). The slides were incubated with anti-MCM2 (abcam, ab108935), anti-SOX9 (abcam, ab185230), anti-PERIOSTIN (abcam, ab215199), anti-*RUNX2* (abcam, ab236639) and anti-OPN (abcam, ab216406) antibodies at 4 °C overnight. And according to the manufacturer's instructions, slides were visualized using a diaminobenzidine reagent kit (Maixin, Fuzhou, China) after incubation with horseradish peroxidase (HRP) secondary antibody. Then, the immunostained sections were counterstained with hematoxylin.

For immunofluorescence, after being dehydrated, and treated for antigen retrieval, tissue sections were blocked with 2.5% bovine serum albumin (BSA). Then, immunostainings were obtained using the following primary antibodies: anti-KI67 (abcam, ab15580), VEGFA (abcam, ab52917), anti-HIF1a (abcam, ab179483), anti-OCN (Santa, sc-390877) and anti-OSX (abcam, ab22552). After incubation with primary antibodies overnight at 4 °C, the secondary antibodies were added: Alexa Fluor 488 and 549 (Jackson Immuno Research, West Grove, PA, USA). The specimens were then counterstained with 40,6-

diamidino-2-phenylindole (DAPI) and subjected to ProLong^{TM} Gold Antifade Mountant (Life Technologies, Grand Island, NY, USA).

Radiographic procedures

For X-ray analysis, after genotyping with tail or fingers, mice at different ages and genotypes were subjected to a radio system (In-Vivo FX PRO; Bruker, Billerica, MA, USA) at 26 kV. For micro-CT analysis, the morphology of the femur and skull, the trabecular bone volume/tissue volume ratio (BV/TV: %), trabecular thickness (Tb.Th: m), trabecular number (Tb.N: mm-1), and trabecular bone separation (Tb.Sp: m) were recorded using SkyScan1176 (70 kV, 114 μA, 8 w, 200 ms, step=10.5 μm) (Bruker) and analyzed by CTAn (version 1.18.8.01) (Bruker).

Whole-mount Alizarin red and Alcian blue staining

The genotypes of different newborn transgenic mice (postnatal day 0, PN0) were subjected to PCR using tail or finger DNA. Only female mice were used for whole-mount staining. For skeletal preparation, after being sacrificed in CO_2, mice were skinned in hot tap water for 20 s at 65 °C. We then remove the eyes, skin, internal organs, adipose tissue, and other soft tissues from the body as much as possible. Next, the embryos were fixed in 95% ethanol overnight at room temperature and then in acetone for 24 h. Alizarin red (Sigma-Aldrich, St Louis, MO, USA) and/or Alcian blue solutions (Sigma-Aldrich) with 10% glacial acetic acid and 70% ethanol were used for staining for three days at 37 °C with mild rotation. After being washed in 1% KOH for 2-4 days, the bodies were subjected to photograph. The cartilages were characterized by blue staining, and mineralized bone was characterized by red staining.

Cell culture and osteoblastic induction

The primary mouse bone marrow stromal cells (BMSCs) were isolated from the femur from 4-6 – week-old female mice as described in a previous protocol[1]. After treatment with eBioscience™ 1x Red Blood Cell Lysis Buffer (Life Technologies, Carlsbad, CA, USA), remaining cells were maintained in α-MEM (Hyclone, Logan, UT, USA) containing 10% fetal bovine serum (FBS) (Gibco, Waltham, MA, USA), penicillin (100 U/mL), and streptomycin (100 μg/mL) in a 37 °C incubator containing 5% CO_2. When cells reached

80-85% confluence, BMSCs were subjected to induction by osteoblastic induction medium comprised of 50 μg/mL of ascorbic acid (Sigma-Aldrich), 10 mmol/L of sodium β-glycerophosphate (Sigma-Aldrich), and 10 nmol/L of dexamethasone (Sigma-Aldrich). 14 days after induction, fixed cells were stained with 2% Alizarin Red S (Sigma-Aldrich) at pH 4.2 to evaluate the cell-matrix mineralization. For hypoxia induction, MC3T3-E1 cells were maintained in an atmosphere containing 1% O_2, 5% CO_2, and 94% Nitrogen using an air-tight chamber (Billups- Rothenburg, Del Mar, CA) after changing the media 48 h-post transfection of siRNA or miRNA inhibitor. MC3T3-E1 cells were transfected with siHif1a (GenePharma, Suzhou, China) and a negative control scrambled siRNA (GenePharma) or inhibitors for miR-3065-5p and scrambled control (Ambion, USA) at a final concentration of 50 nM using Lipofectamine 2000 (Thermo Fisher Scientific) according to manufacturer's instructions.

Cell proliferation assay

For the CCK-8 assay, BMSCs were seeded into 96-well plates and maintained in a normal culture medium for different lengths of culture time (1,3, 5, 7, 9, and 11 days). After different culture times, 10 μL of CCK-8 (A311-01; Vanzyme, China) per 100 μL of culture medium was added to each well and subjected to incubation at 37 °C for 1 h. The optical density (OD) of each well was read at a wavelength of 450 nm according to manufacturer's instructions. Three replicates were performed.

Mouse serum biochemical assay

We analyzed the concentration of serum markers for bone turnover using carboxyl-terminal telopetide of type I collagen (CTX) and N-terminal propeptide of type I procollagen (PINP). Briefly, after fasting for 6 hours, 4-week-old mice with genotype of wildtype, Runx2$^{+/-}$, Mir338$^{-/-}$, and Mir338$^{-/-}$;Runx2$^{+/-}$ were subjected to blood collection using cheek pouch. Three individuals were used for each genotype, and 100uL of serum were collected from each mouse. Serum samples were freezed to -80°C immediately after collection. Measurements were performed together when all samples were collected. ELISA kit for CTX (RatLaps™ (CTX-I) EIA, AC-06F1, Fountain Hills, AZ) and PINP (Rat/Mouse PINP EIA, AC-33F1, Fountain Hills, AZ) were used for the assays.

Quantitative reverse transcriptase PCR (qRT-PCR)

Total RNA from cultured cells was isolated using miRNeasy mini kit (QIAGEN). The hind limbs from E16.5 mice embryos with different genotypes were grinded in liquid nitrogen and subjected for RNA extraction using miRNeasy mini kit (QIAGEN). After removal of genomic DNA using Turbo-DNaseI using DNaseI (Promega), equal amount of RNA were subjected to HiScript® Q RT SuperMix for qPCR (Vanzyme, R122-01) for quantification of RNA expression of coding genes, with GAPDH served as internal control. As for quantification of miRNA, total RNA was subjected to miRNA Reverse Transcription Kit (QIAGEN) and miScript SYBR Green PCR Kit (QIAGEN), with U6 as internal control. Quantitative real-time PCR (qRT-PCR) was performed using the CFX96 Touch Real-Time PCR Detection PCR System (BIO-RAD, Berkeley, CA, USA). $2^{-\Delta\Delta CT}$ values were used for comparing the fold-chages in different condition.

Dual luciferase activity assay for promoter and 3'UTR

For functionally validating miR-3065-5p directly targeting Hif1a, the DNA fragments of the mouse *Hif1a* 3'UTR, including the predicted miRNA binding sites, were synthesized by Sangon Biotech (Shanghai, China) and ligated into pMIR-reporter (Ambion, USA). Site-directed mutageneses were performed with overlapping PCR to remove the each or both binding sites of miR-3065-5p. Mimics for miR-3065-5p or scramble (Ambion) were co-transfected with related 3'UTR reporter plasmids along with pRL-TK (Promega, USA) into MC3T3-E1 cells using Lipofectamine 2000 (Life Technologies). 72 hours after transfection, cells were harvested, and total lysates were subjected to quantification of luminescent signal with Dual-Luciferase Reporter Assay System (Promega) according to the manufacturer's instructions. Each transfection group were repeated for at least 3 times, and quantification for each well was repeated for 3 times. Value for the firefly luciferase assay was normalized to the Renilla luciferase activity then normalized to the relative firefly activity from the empty control group.

Enzyme-linked immunosorbent assay (ELISA)

To determine the secretion of VEGFA, the concentration of VEGFA BMSCs supernatant or serum was measured using a VEGFA ELISA kit (ab119565, Abcam). Briefly, BMSCs harvested from the femur with different genotypes were seeded at $1x10^5$ in 24-well plate and culture until confluence. Cell were then serum-starved for 1 hour, and the culture

supernatant was collected which was subjected to ELISA quantification. The concentration of VEGFA in the supernatant was normalized to the number of BMSCs remaining in the well. Serum was collected from mouse blood with different genotypes and stored in -80°C.

Chromatin immunoprecipitation quantitative PCR

BMSCs from 4-week-old DoubleMutant and *Runx2*-Het femurs were harvested and exposed to osteoblastic induction or normal culture medium for 0 and 9 days. A Magna ChIP One-Day Chromatin Immunoprecipitation Kit (#17-100859; Millipore/Upstate, Lake Placid, NY) was used for ChIP assays, following the manufacturer's guideline. Approximately 1 × 10^6 cells were fixed with 1% formaldehyde. After chromatin preparation, 5% chromatin was left for input control and the remaining chromatin immunoprecipitation was performed using an anti-HIF1A antibody (abcam, ab179483) or anti-*RUNX2* antibody (#12556; Cell Signaling Technology). ChIP-RE-ChIP was performed using Re-ChIP-IT® (53016; Active Motif, USA) according to manufacturer's guidelines and previous protocol[2]. Briefly, 10x10^6 BMSCs were used for ChIP-RE-ChIP. Chromatin fragments were pulled down by anti-HIF1A antibody. The first eluted ChIP producted were diluted 20 times in the dilution buffer and incubated with anti-*RUNX2* antibody or rabbit IgG antibody (ABclonal Biotechnology, Woburn, MA). Realtime PCR was performed to analyze the ChIP or ChIP-RE-ChIP product. Three replicates were performed for BMSCs of each genotype.

References

1. Soleimani, M. & Nadri S. A protocol for isolation and culture of mesenchymal stem cells from mouse bone marrow. *Nat. Protoc.* **4**,102-106 (2009)
2. Beischlag, T. V., Prefontaine G. G. & Hankinson O. ChIP-re-ChIP: Co-occupancy Analysis by Sequential Chromatin Immunoprecipitation. *Methods Mol. Biol.* **1689**,103-112 (2018)

Supplementary Figure and Figure Legends

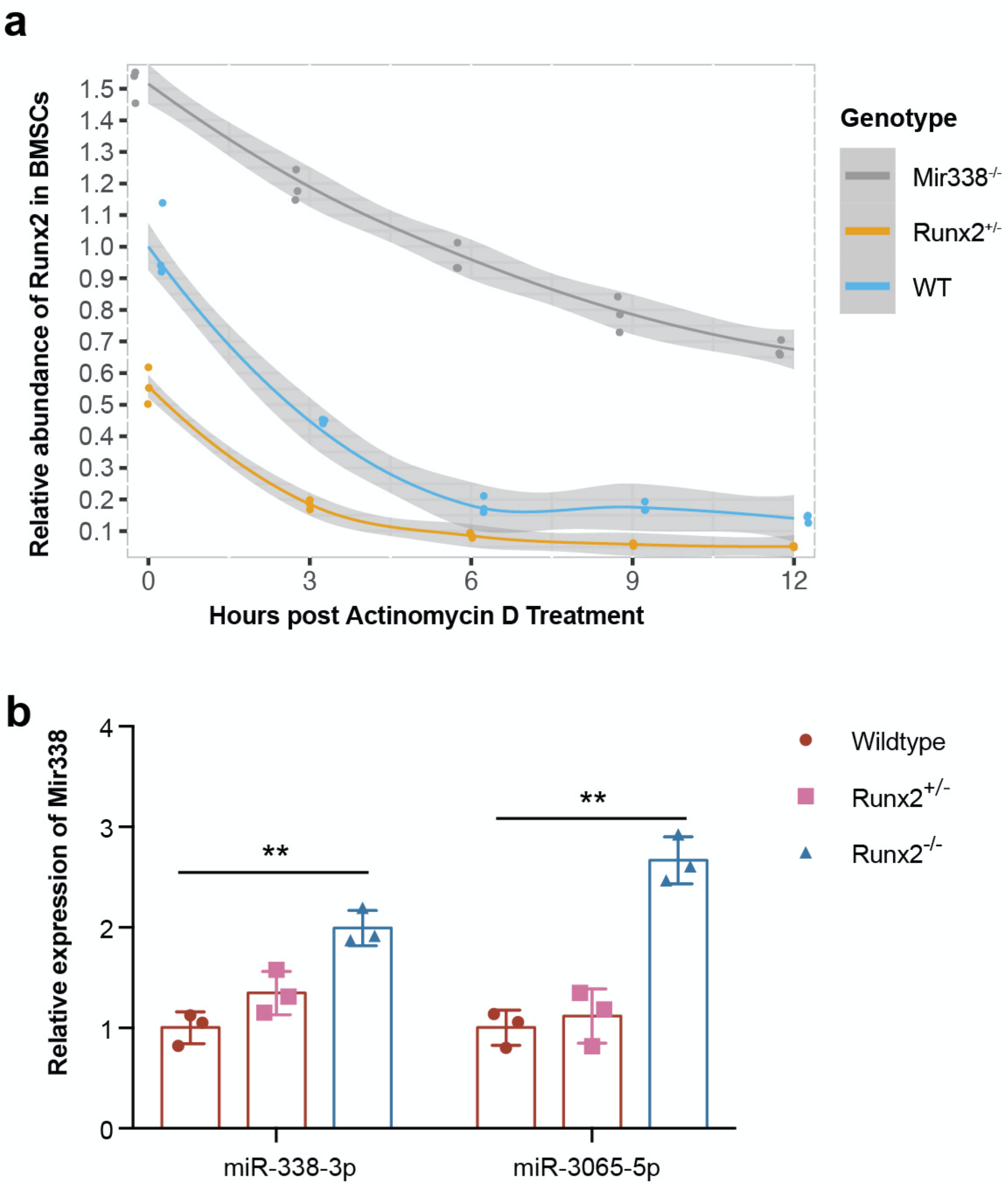

Supplementary Fig.1 Correlation between *Mir338* cluster and *Runx2* in BMSCs. **(a)** qRT-PCR quantification of mRNA abundance of *Runx2* in from BMSCs with different genotype after Actinomycin D treatment. All abundance of Runx2 was normalized to its expression in wildtype (WT) BMSCs 0 hours before actinomycin D treatment. **(b)** Expression of *Mir338* cluster in BMSCs from mice with different genotypes

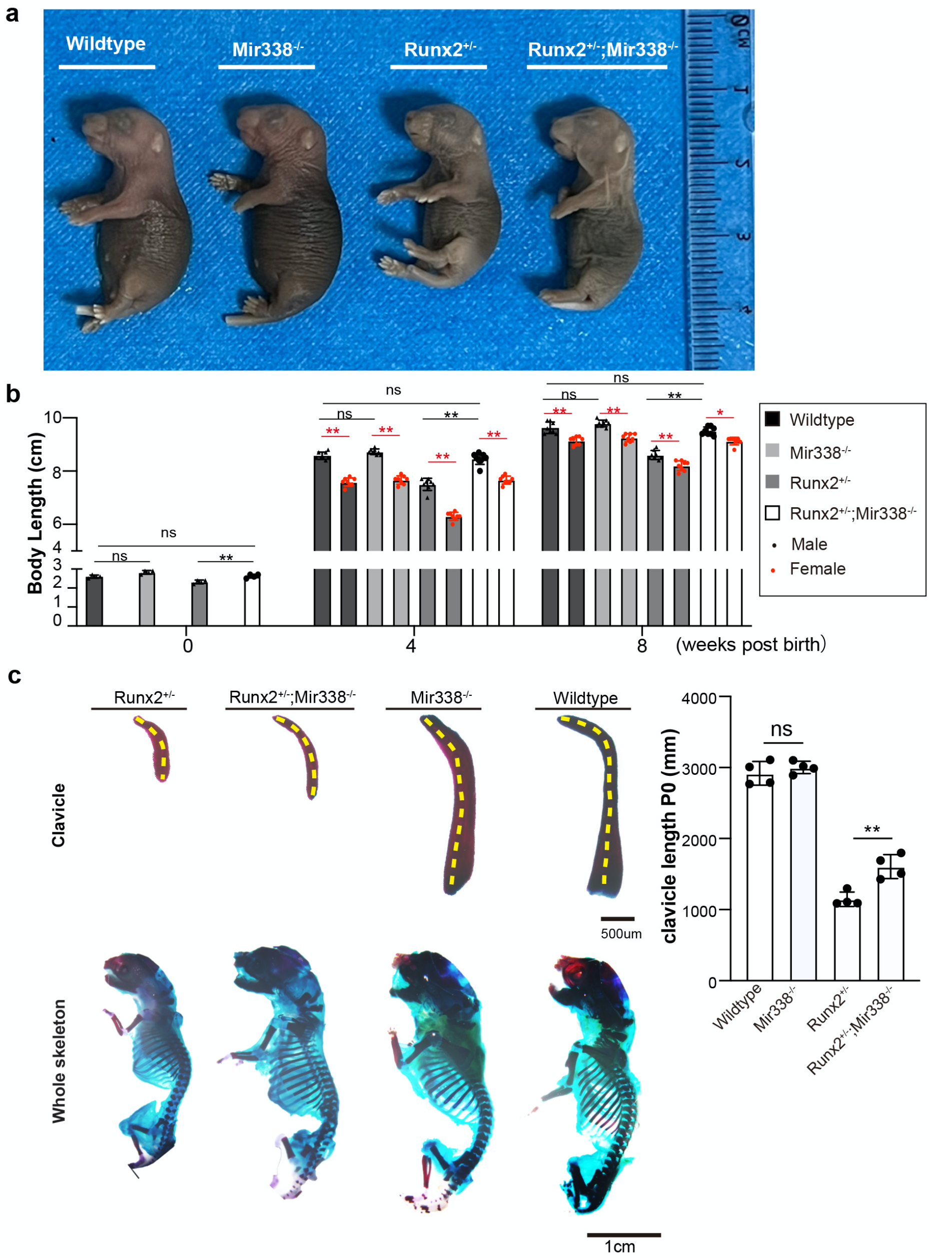

Supplementary Fig.2 Supplementary skeletal information for wildtype, Runx2$^{+/-}$, Mir338$^{-/-}$, and Mir338$^{-/-}$; Runx2$^{+/-}$ mice. **(a)** Representative gross images for newborn *Runx2*$^{+/-}$, Mir338$^{-/-}$, *Runx2*$^{+/-}$; *Mir338*$^{-/-}$ and wildtype mice (gender not tell) from the same breed. **(b)** Body length for *Runx2*$^{+/-}$, Mir338$^{-/-}$, *Runx2*$^{+/-}$; *Mir338*$^{-/-}$ and wildtype mice on 4 and 8 weeks after birth with different gender. The measurements for the newborn mice were performed for the same three individuals without telling the gender. Each dot represents mean measurement for each individual. The statisical difference caused by gender were depicted in red color. **(c)** Alizarin red/Alcian blue staining for the clavicles and whoe skeleton for the newborn *Runx2*$^{+/-}$, Mir338$^{-/-}$, *Runx2*$^{+/-}$; *Mir338*$^{-/-}$ and wildtype mice. Yellow dash lines indicates the path of measurement. The deformity on the skull was caused by

manual disturbance when taking the stereoscope pictures. *: P<0.05 **: P<0.01, ns: not significant. Each dot represents a individual mouse.

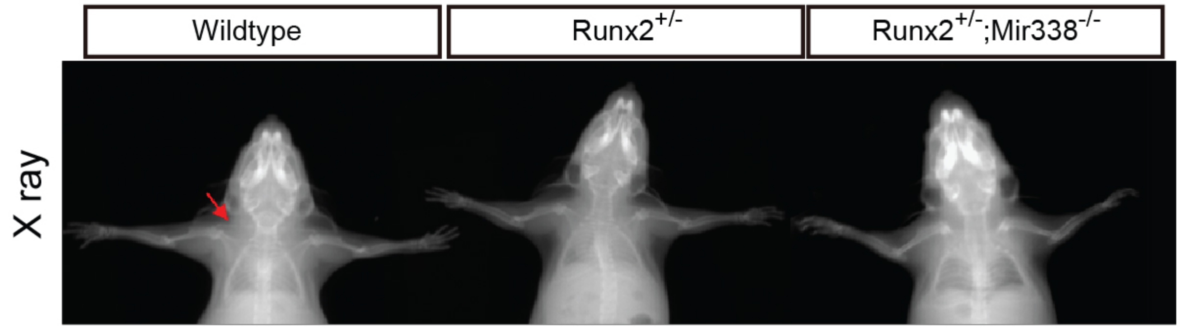

Supplementary Fig.3 X-ray image for head and neck region for 4-week-old mice. Red arrow indicates the presence of clavicle in wildtype but its absence in *Runx2*$^{+/-}$ and *Runx2*$^{+/-}$;*Mir338*$^{-/-}$ mice.

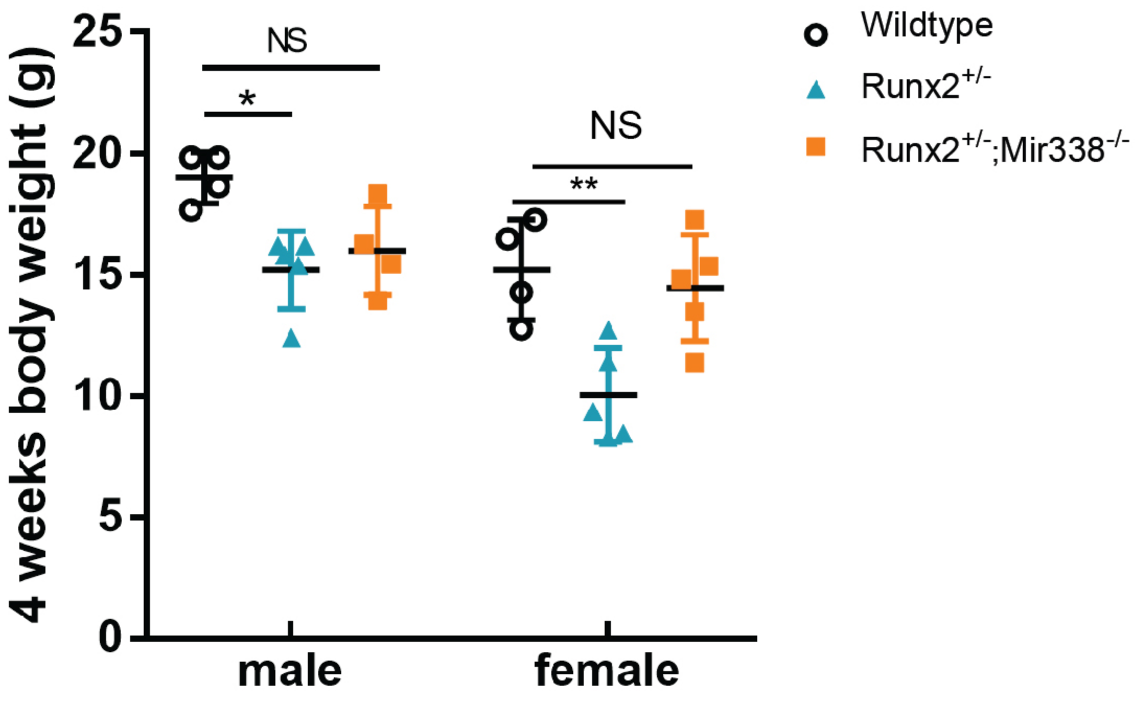

Supplementary Fig.4 Body weight for 4-week-old mice with different genotypes and different gender. Unpaired t-test was performed. *: P<0.05 **: P<0.01, NS: not significant.

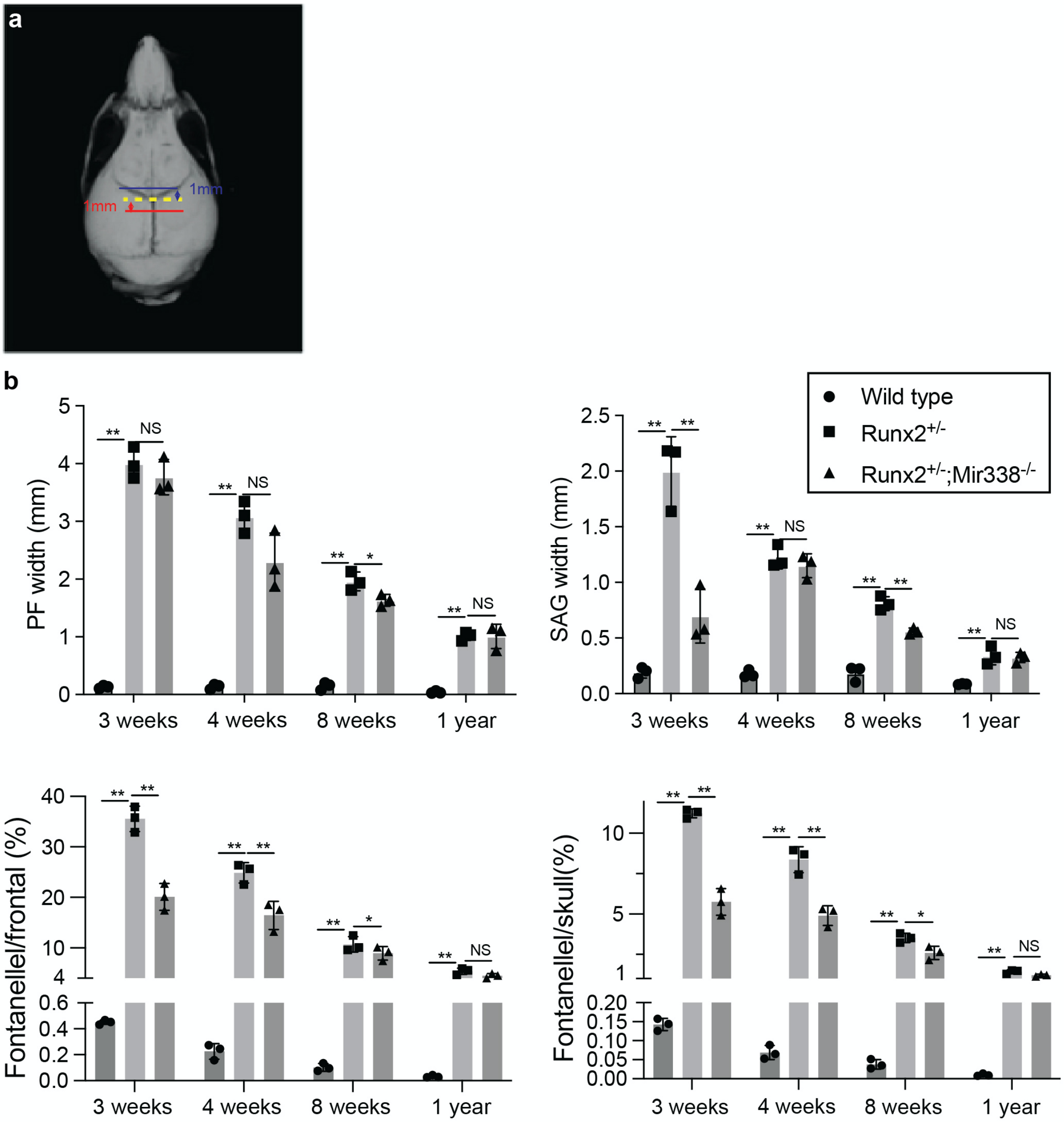

Supplementary Fig.5 Measurements of PF and SAG suture width in wildtype, Runx2$^{+/-}$; Mir338$^{-/-}$ mice at different ages. **(a)** Schematic diagram showing the fixed point for measurement. Blue line showing the position of PF suture measurement and red line showing the position of SAG measurement. . **(b)** Measurements of PF and SAG suture width. Each dot represents a individual. NS: not significant, *: *P*<0.05, **: *P*<0.01

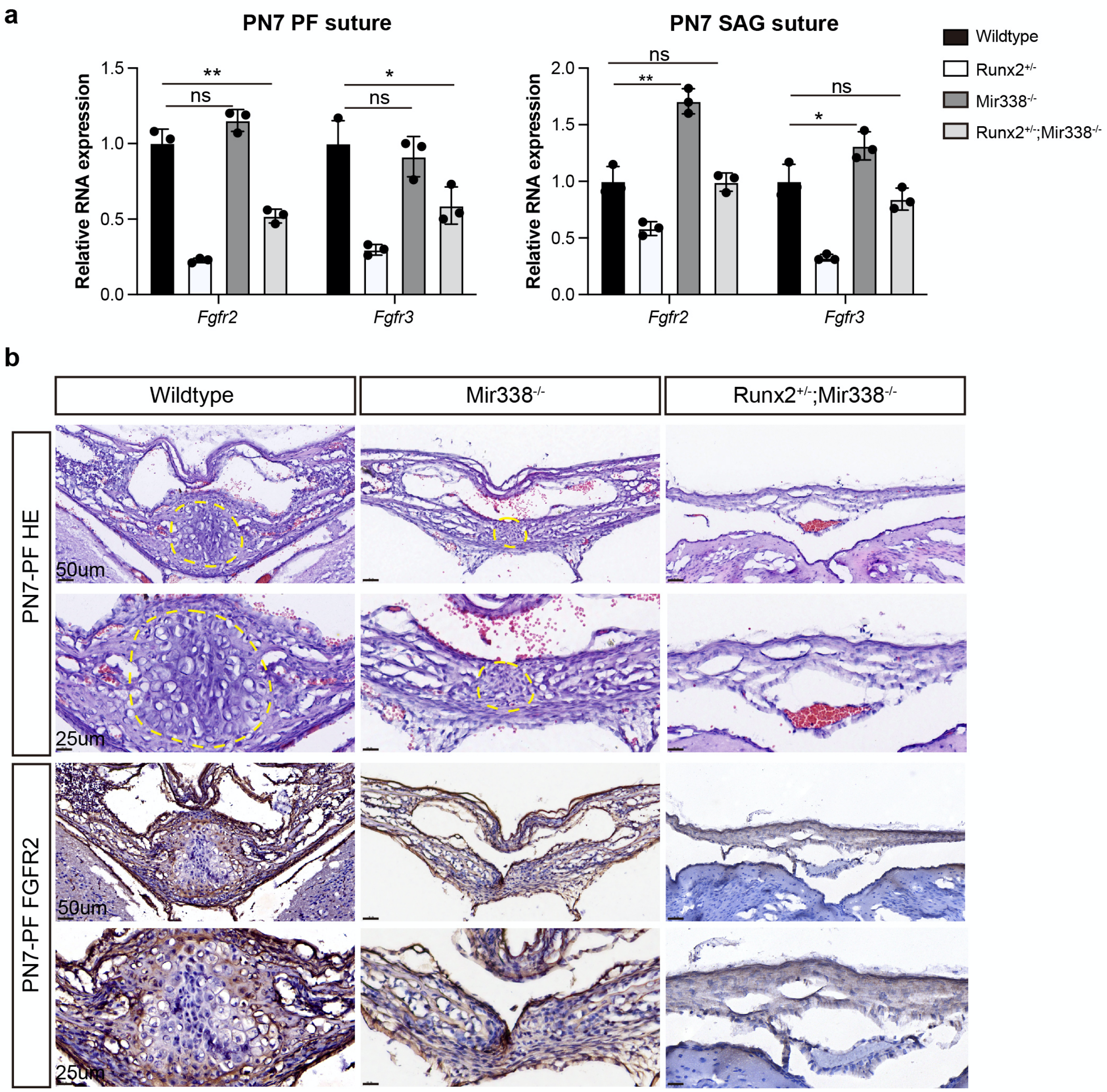

Supplementary Fig.6 Expression pattern of Fgfr2 and Fgfr3 in the PF and SAG suture. **(a)** qRT-PCR for Fgfr2 and Fgfr3 in PF and SAG suture from PN7 mice. An unpaired t-test was performed. *: *P*<0.05 **: *P*<0.01, NS: not significant. Data were presented as means ± SEMs, each dot represent a measurement from one individual mice. **(b)** HE staining and FGFR2 IHC in the PF sutures collected from PN7 wildtype, Mir338$^{-/-}$ and Runx2$^{+/-}$; Mir338$^{-/-}$ mice.

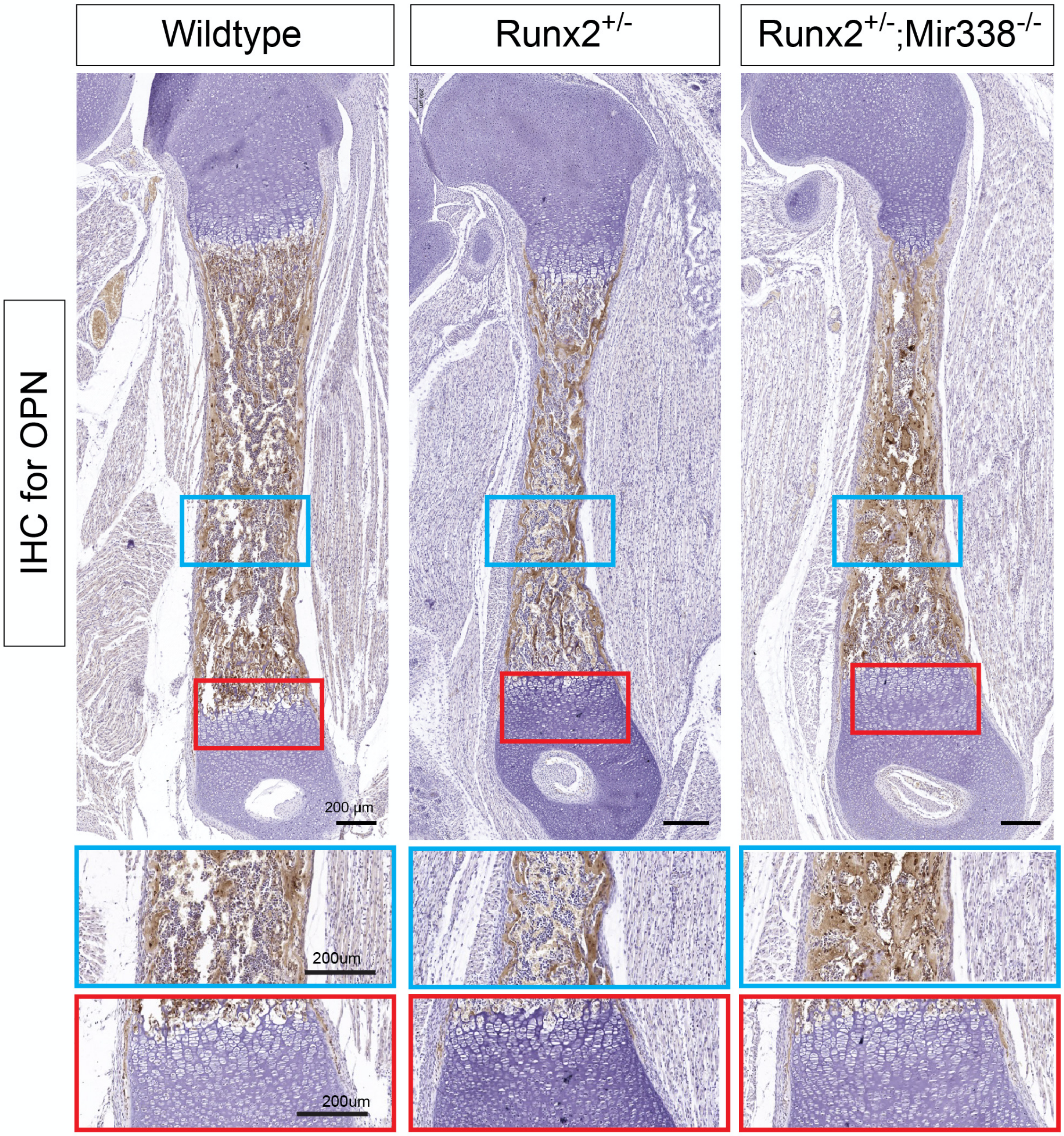

Supplementary Fig.7 OPN IHC staining in the femurs from PN0 *Runx2*$^{+/-}$, *Runx2*$^{+/-}$; *Mir338*$^{-/-}$ and wildtype mice (Supplemented to Figure 2)

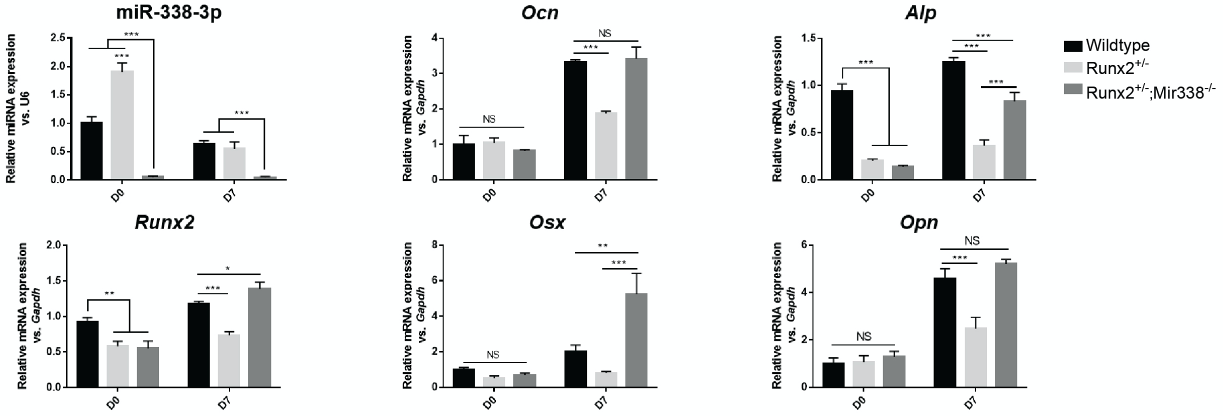

Supplementary Fig.8 qRT-PCR for miR-338-3p, *Ocn*, *Alp, Runx2*, *Osx*, and *Opn* in BMSCs with different genotypes cultured in osteoblastic induction medium for 9 days. An unpaired t-test was performed. *: *P*<0.05 **: *P*<0.01, NS: not significant. Data were presented as means ± SEMs.

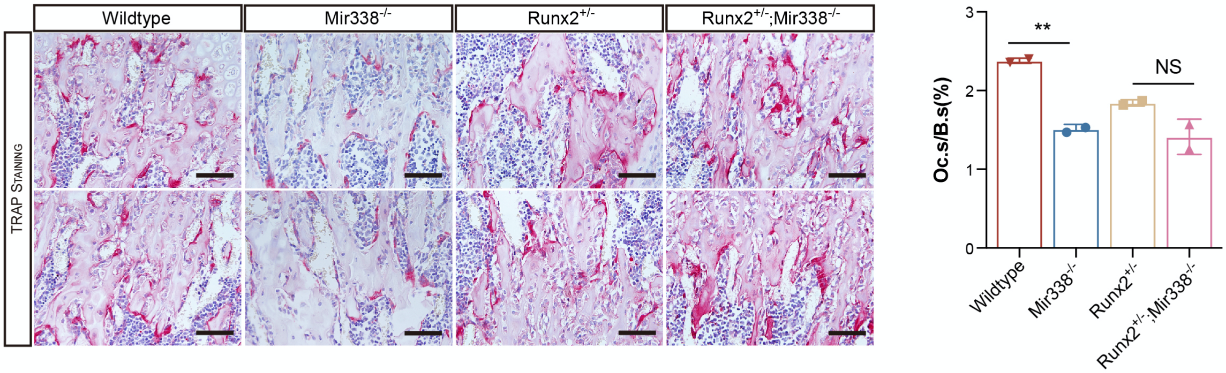

Supplementary Fig.9 Representative image for tartrate-resistant acid phosphatase (TRAP) staining for femurs from 8-week-old wildtype, *Mir338*$^{-/-}$, *Runx2*$^{+/-}$ and *Runx2*$^{+/-}$; *Mir338*$^{-/-}$ mice, along with quantification. Data were presented as means ± SEMs. An unpaired t-test was performed. **: *P*<0.01, NS: not significant.

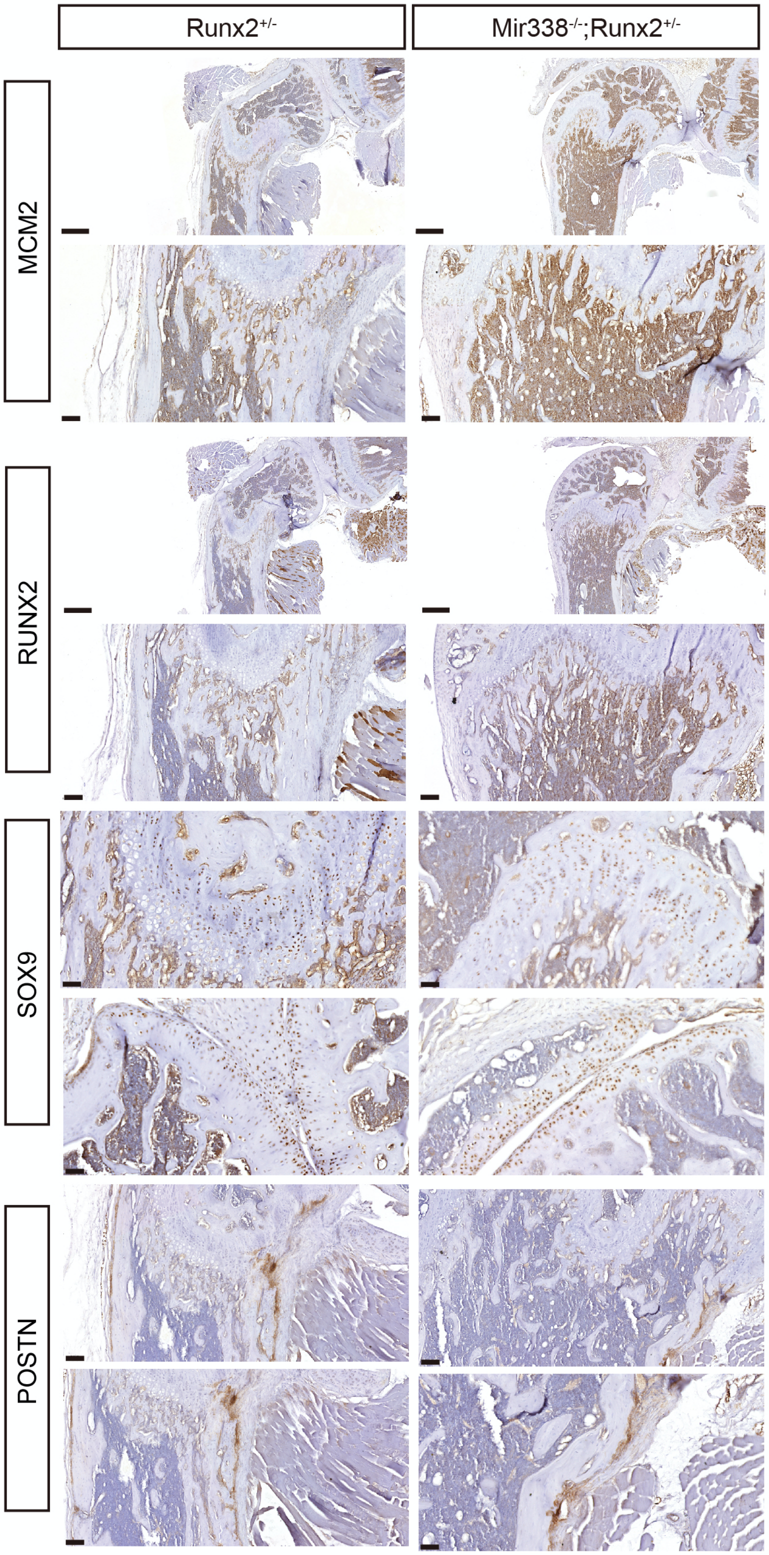

Supplementary Fig.10 Immunohistochemistry for MCM2, RUNX2, SOX9 and POSTN in femurs from 8-week-old *Runx2*$^{+/-}$ and *Runx2*$^{+/-}$; *Mir338*$^{-/-}$ mice. Scale = 200 μm

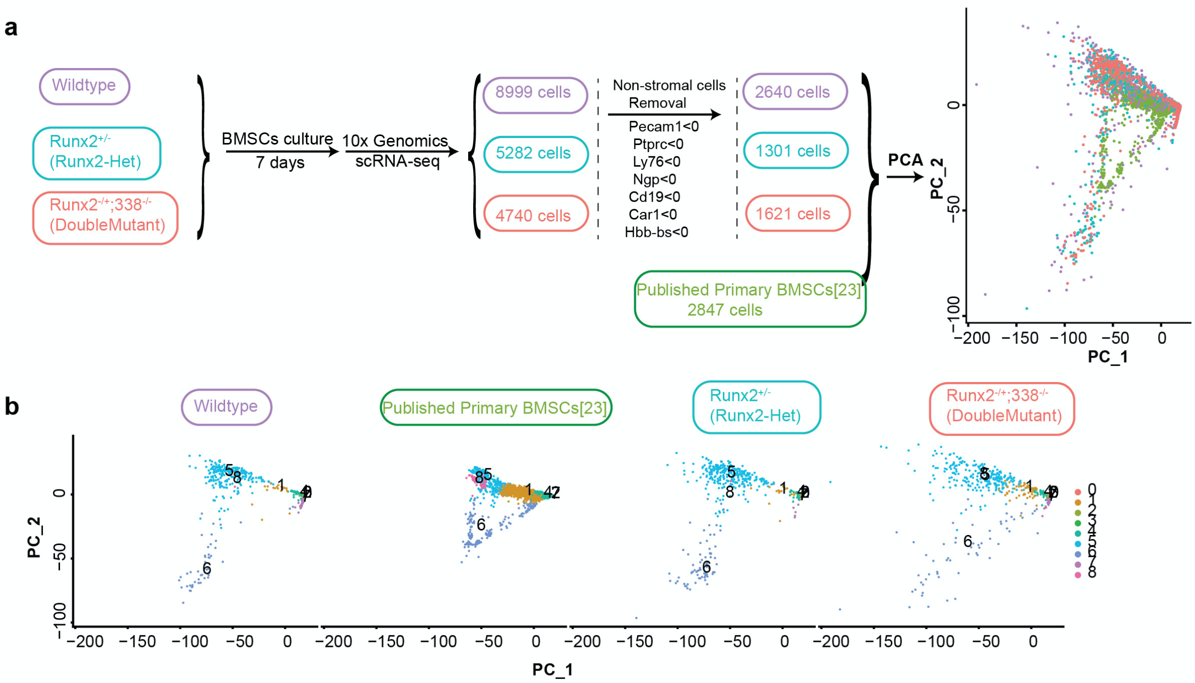

Supplementary Fig.11 Filtering and comparing scRNA-seq for BMSCs in this study and the published one[23] **(a)**. PCA was used for comparison **(b)**.

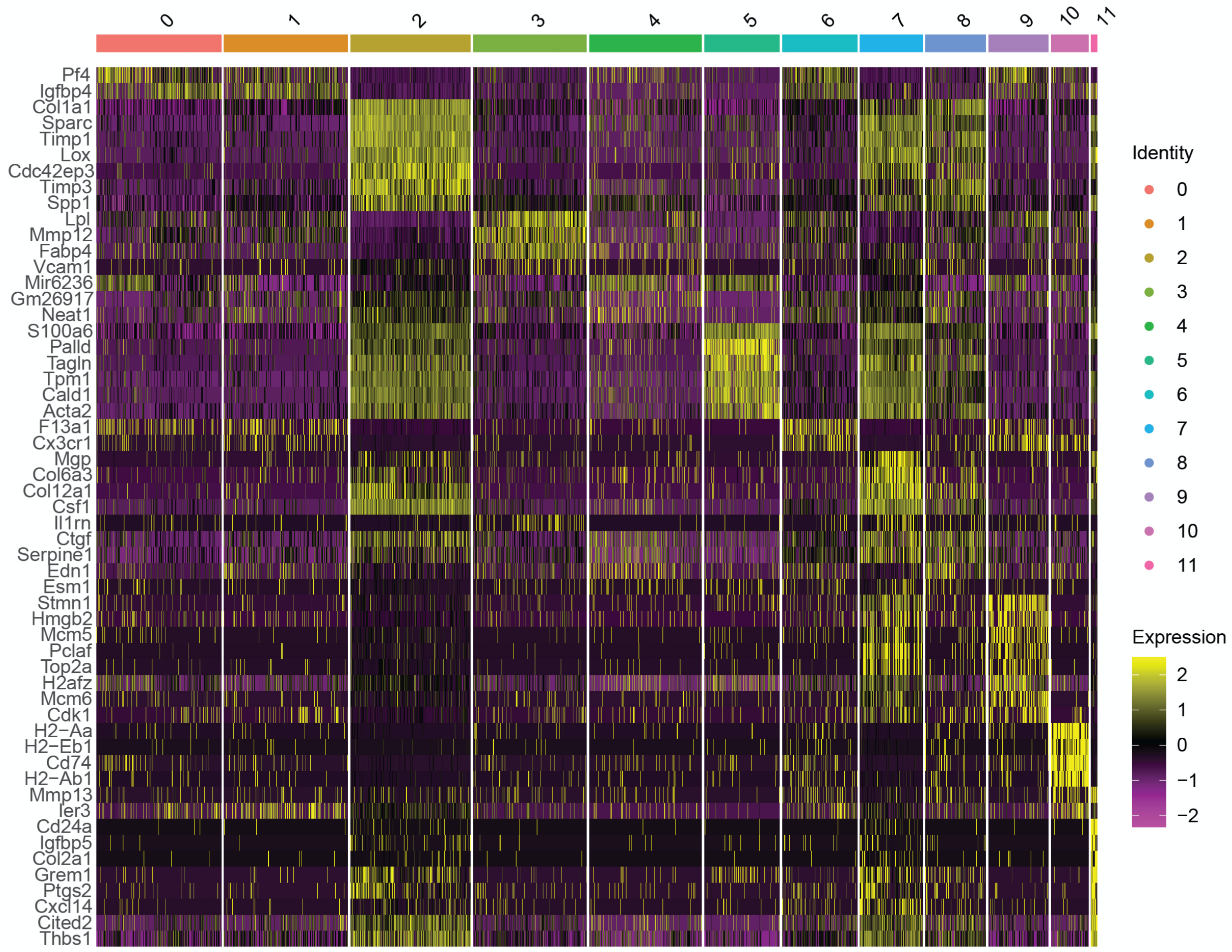

Supplementary Fig.12 Heatmap for top genes in different clusters of scRNA-seq for BMSCs across all genotypes.

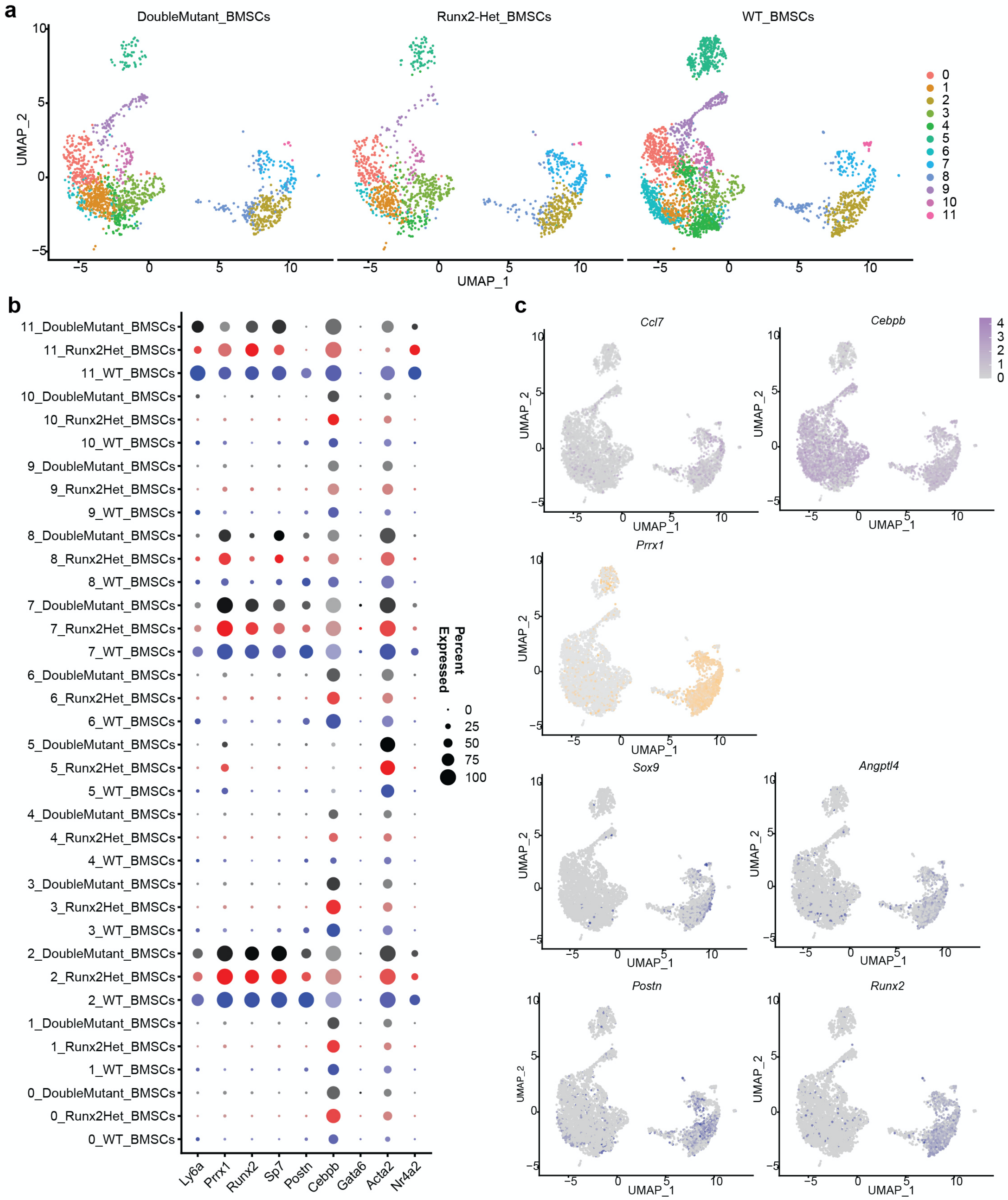

Supplementary Fig.13 Clustering and marker genes plot in scRNA-seq for BMSCs across all geneotype. **(a)** UMAP plot for the scRNA-seq profile with different genotypes. **(b)** Lineage-related genes enrichment in different cluster across three genotypes. **(c)** Feature plot for different lineage priming genes. Purple for adipogenesis, orange for mesenchymal stem cells, and blue for osteogenesis/chondrogenesis related genes.

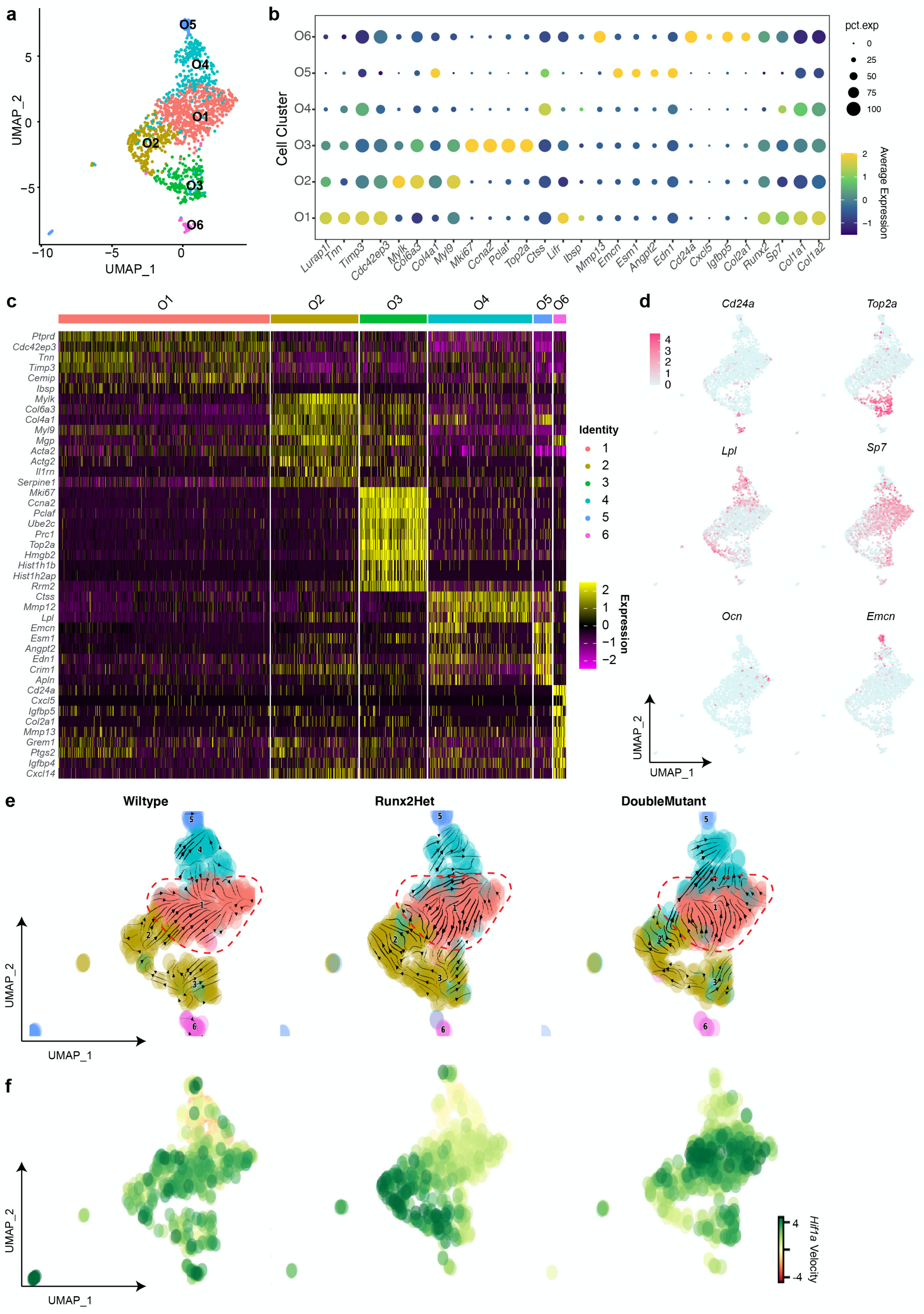

Supplementary Fig.14 Subclusters of the osteoblastic lineage. **(a)** UMAP plot for the subset osteoblastic lineages in the combined scRNA-seq profile. **(b)** Lineage-related genes enrichment in different cluster across three genotypes. **(c)** Heatmap for marker genes in the osteoblastic lineage **(d)** Feature plot for the marker genes in different subclusters or osteoblasti lineage. **(e)** RNA velocity plot in UMAP indicate the dynamical transcriptome changes in osteoblasti lineage of each genotype. **(f)** RNA velocity portrait for *Hif1a* in osteoblasti lineage of each genotype

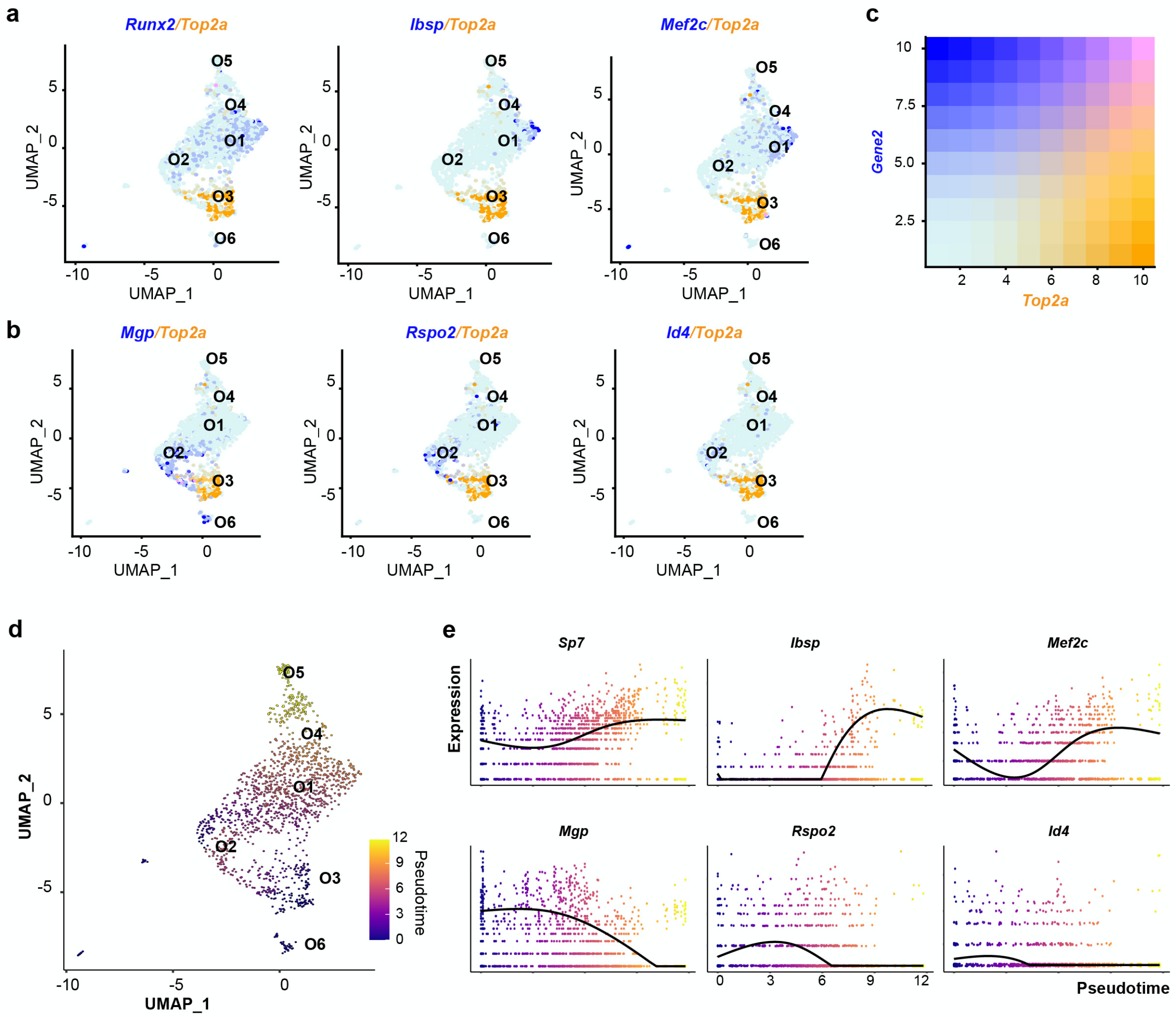

Supplementary Fig.15 Marker genes and pesodotime trajectory in osteo lineage. **(a)** Feature plots for marker genes in O1 subcluster. Marker genes in blue, and Top2a in orange. **(b)** Feature plots for marker genes in O1 subcluster. Marker genes in blue, and Top2a in orange. **(c)** Scale for the two-genes feature plots in (a) and (b). Top2a, in orange, was scaled from 0 to 10; marker gene, in blue, was scaled from 0 to 10. **(d)** UMAP plot for pseudotime trajectory in osteo-lineage. O3 and O6 were chosen as the root. **(e)** Gene expression for marker genes in O1 (*Sp7*, *Ibsp* and *Mef2c*) and O2 (*Mgp*, *Rspo2* and *Id4*) in pseudotime.

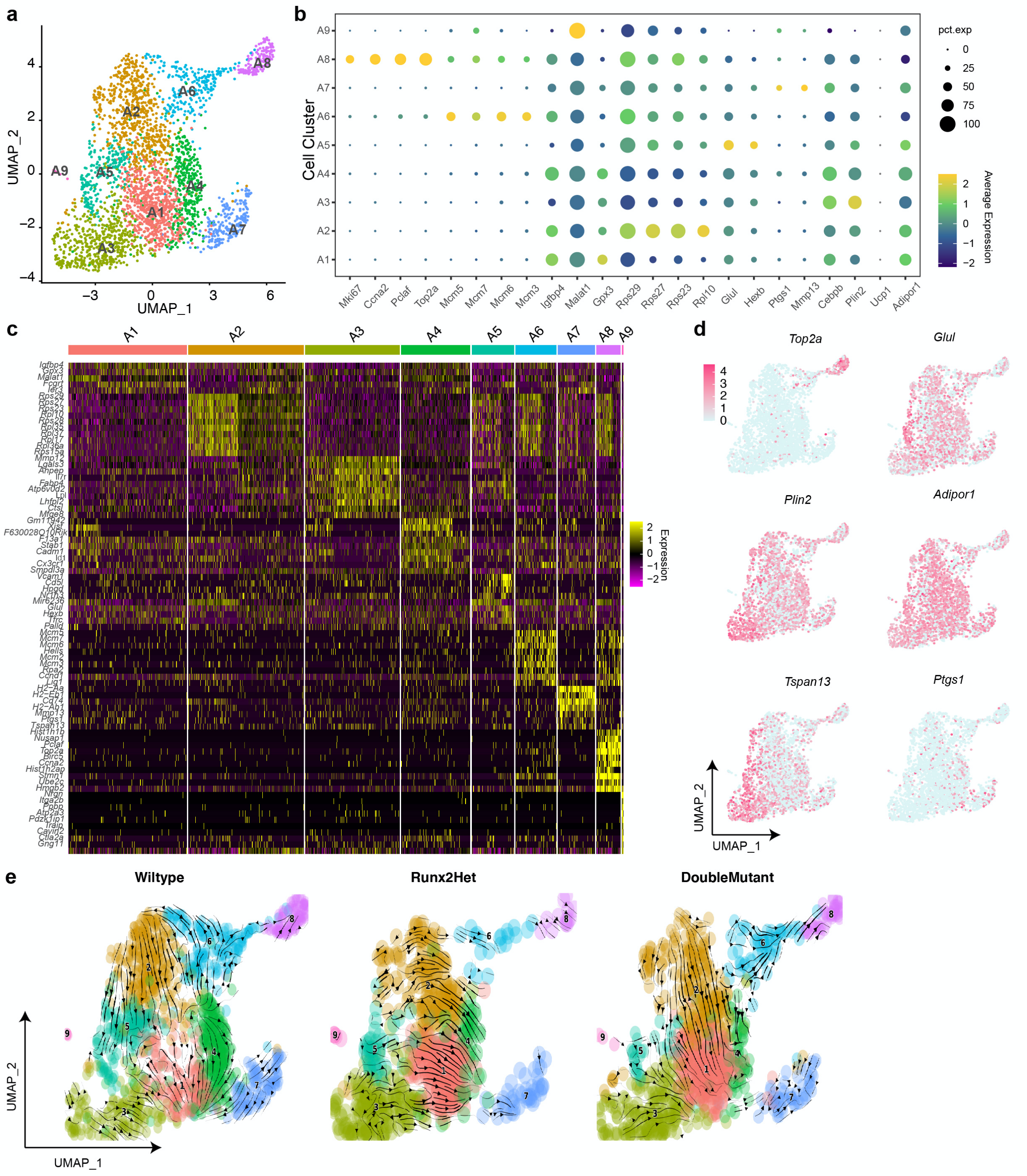

Supplementary Fig.16 Subclusters of the adipogenic lineage. **(a)** UMAP plot for the subset adipogenic lineages in the combined scRNA-seq profile. **(b)** Lineage-related genes enrichment in different cluster across three genotypes. **(c)** Heatmap for marker genes in the adipogenic lineage **(d)** Feature plot for the marker genes in different subclusters or adipogenic lineage. **(e)** RNA velocity plot in UMAP indicate the dynamical transcriptome changes in adipogenic lineage of each genotype.

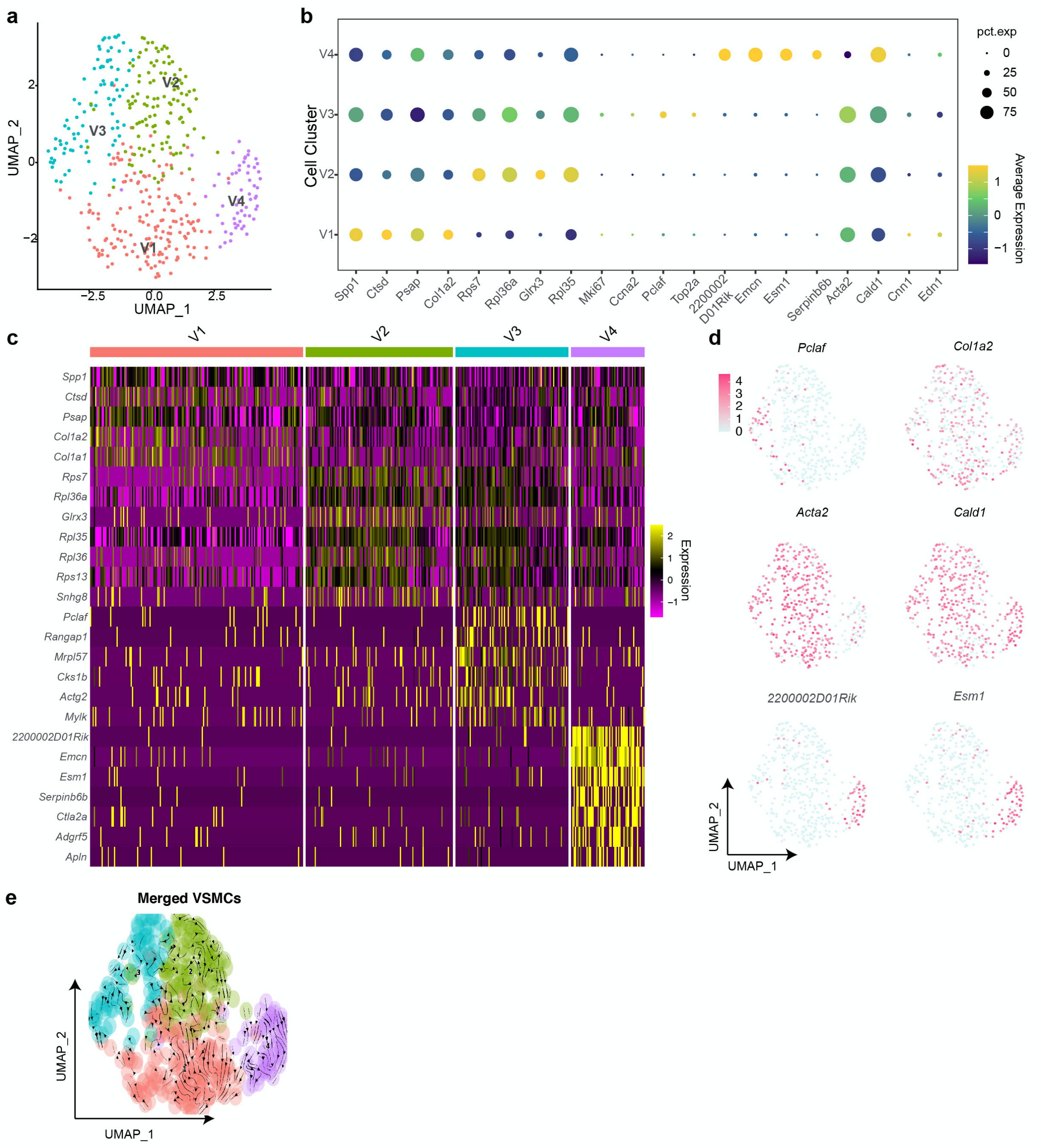

Supplementary Fig.17 Subclusters of the VSMCs lineage. **(a)** UMAP plot for the subset VSMCs lineages in the combined scRNA-seq profile. **(b)** Lineage-related genes enrichment in different cluster across three genotypes. **(c)** Heatmap for marker genes in the VSMCs lineage **(d)** Feature plot for the marker genes in different subclusters or VSMCs lineage. **(e)** RNA velocity plot in UMAP indicate the dynamical transcriptome changes in adipogenic lineage of combined genotypes (due to limited cell number, RNA velocity cannot be faithfully performed for the different genotypes respectively).

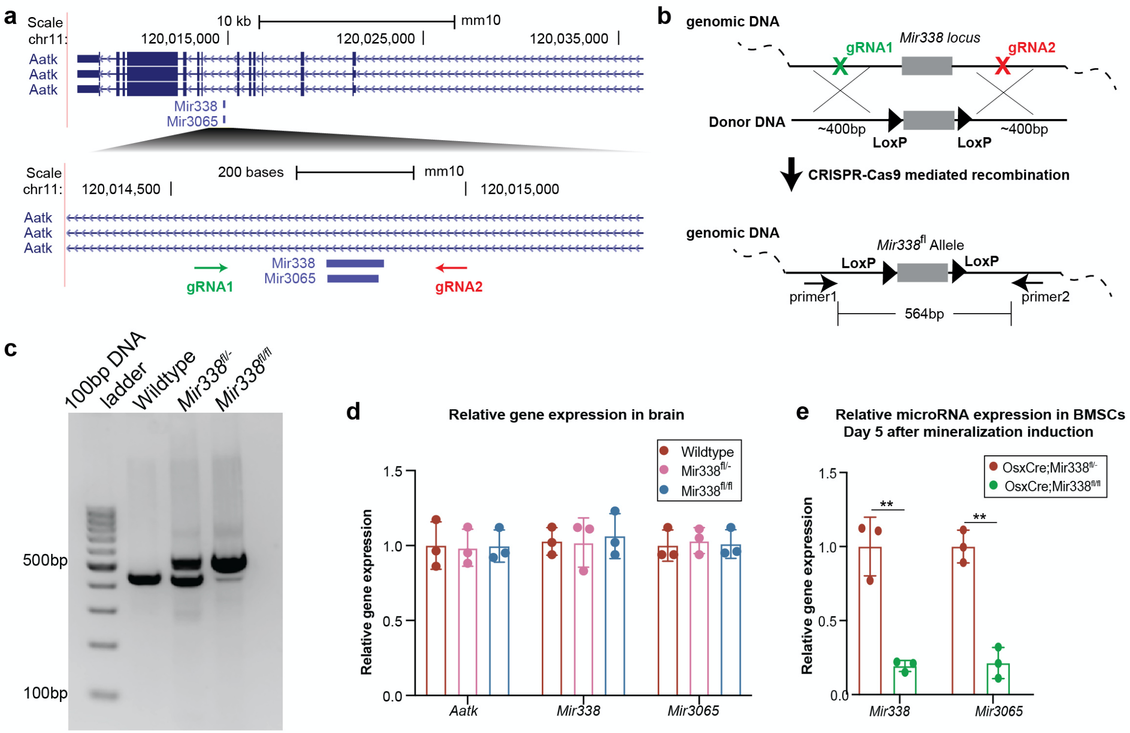

Supplementary Fig.18 Design, generation, and validation of Mir338 conditional knockout mice. **(a)** Genomic location of two guide RNAs used for CRISPR-Cas9-mediated knockin. **(b)** Design of Mir338^{fl} allele. **(c)** Representative image for PCR-base genotyping of Mir338$^{fl/fl}$ mice. **(d)** qRT-PCR for miR-338-3p, miR-3065-5p, and *Aatk* in the brain collected from different mice. **(e)** qRT-PCR for miR-338-3p and miR-3065-5p in the BMSCs from Mir338$^{fl/fl}$ and OsxCre; Mir338$^{fl/fl}$ cultured for five days in osteoblastic induction medium.

a

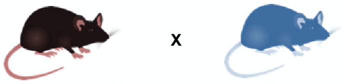

OsxCre;Mir338$^{fl/fl}$ x Mir338$^{fl/fl}$;Runx2$^{+/-}$

Number of genotyped newborn mice

Genotype	Cage_1	Cage_2	Cage_3	Cage_4	Cage_5	Total
Mir338$^{fl/fl}$	72	21	21	21	10	145
OsxCre;Mir338$^{fl/fl}$	51	18	18	19	7	113
Mir338$^{fl/fl}$;Runx2$^{+/-}$	32	15	12	12	5	76
OsxCre;Mir338$^{fl/fl}$;Runx2$^{+/-}$	12	4	6	7	2	32
						366

Percentage of genotypes (%)

Genotype	Actual	Expected
Mir338$^{fl/fl}$	100	100
OsxCre	30.87	50
Runx2$^{-/+}$	20.76	50
OsxCre;Runx2$^{+/-}$	8.731	25

b

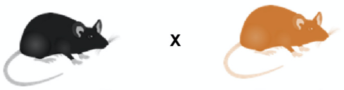

OsxCre;Mir338$^{fl/-}$ x Mir338$^{fl/-}$;Runx2$^{+/-}$

Number of genotyped newborn mice

Genotype	Cage_1	Cage_2	Cage_3	Cage_4	Total
Mir338$^{fl/fl}$	10	12	12	10	44
OsxCre;Mir338$^{fl/fl}$	5	6	6	5	22
Mir338$^{fl/fl}$;Runx2$^{+/-}$	2	3	2	3	10
OsxCre;Mir338$^{fl/fl}$;Runx2$^{+/-}$	1	1	2	1	5
Mir338$^{fl/-}$	22	22	23	20	87
OsxCre;Mir338$^{fl/-}$	4	3	4	4	15
Mir338$^{fl/-}$;Runx2$^{+/-}$	4	4	5	3	16
OsxCre;Mir338$^{fl/-}$;Runx2$^{+/-}$	1	0	0	1	2
Wildtype	9	10	11	9	39
OsxCre	5	6	5	5	21
Runx2$^{+/-}$	4	5	5	4	18
OsxCre;Runx2$^{+/-}$	0	0	0	0	0
					279

Percentage of genotypes (%)

Genotype	Actual	Expected
Mir338$^{fl/fl}$	29.03	25
Mir338$^{fl/-}$	43.01	50
OsxCre	20.78	50
Runx2$^{-/+}$	15.77	50
OsxCre;Runx2$^{+/-}$	2.509	25

c

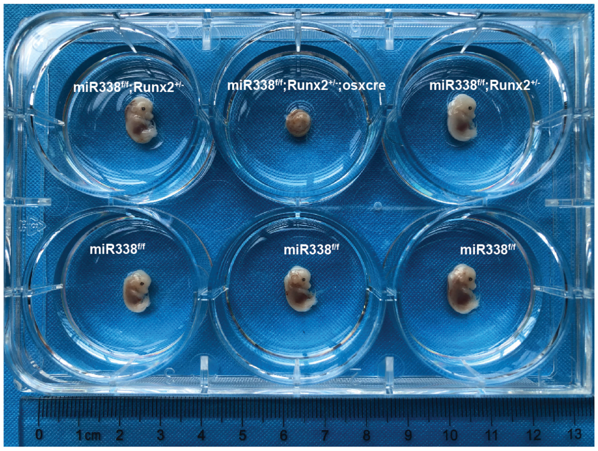

d

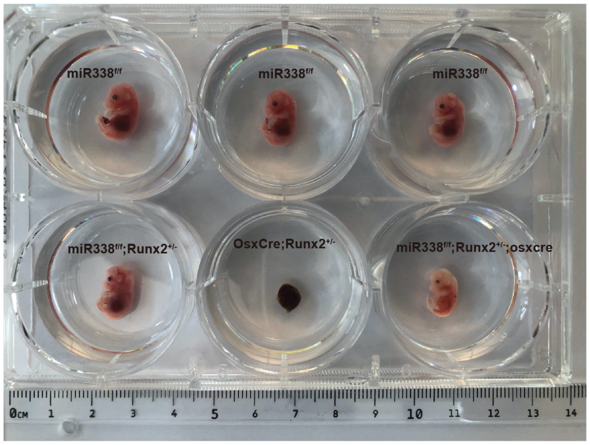

Supplementary Fig.19 Allele frequency for the offspring of different setups **(a, b)** of mouse breeding showing knockout of Mir338 in Osx lineage partially rescues the lethality caused by Runx2 haploinsufficiency and OsxCre allele. Gross image for embryos at the age of embryonic day 14.5 **(c)** and 16.5 **(d)**.

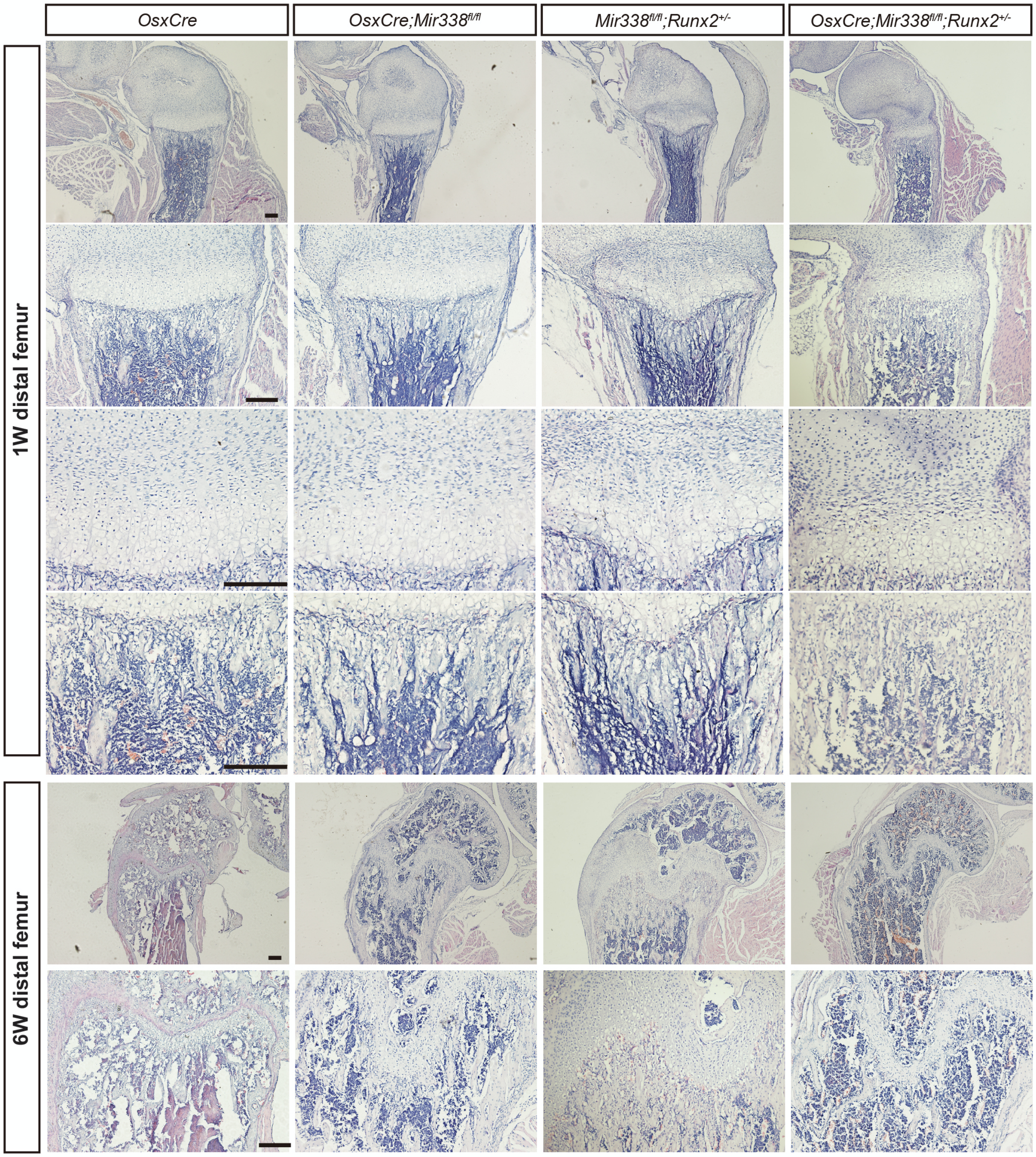

Supplementary Fig. 20 HE staining of distal femurs from 1-week and 6-week old mice with different genotype. Note that the panels for OsxCre and OsxCre;Mir338$^{fl/fl}$;Runx2$^{+/-}$ were exactly the same ones exhibited in Figure 5b

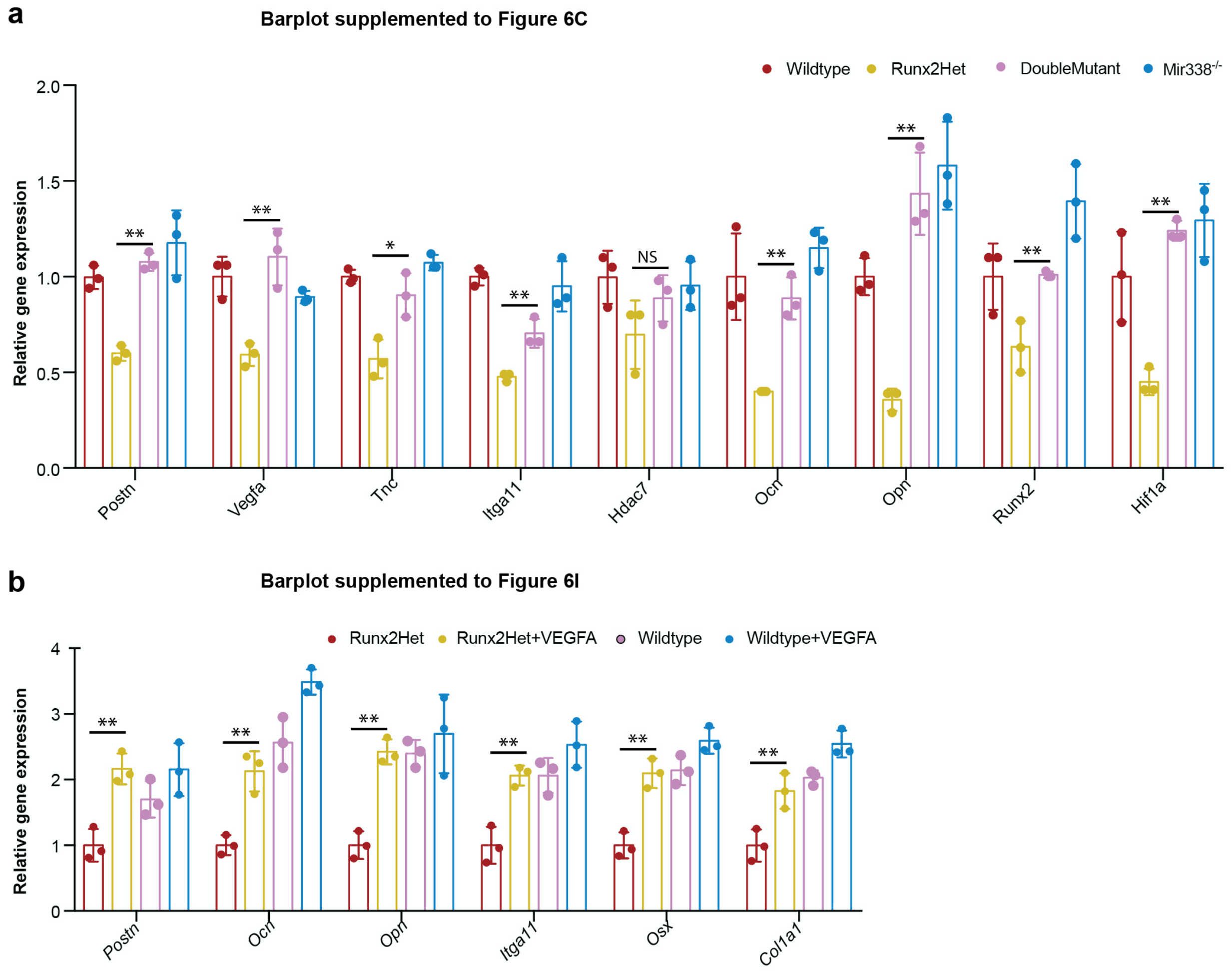

Supplementary Fig. 21 Supplemented bar chart for Figure 6c **(a)** and Figure 6i **(b)**. Data was presented as means ± SEMs. An unpaired t-test was performed. **: *P*<0.01, NS: not significant.

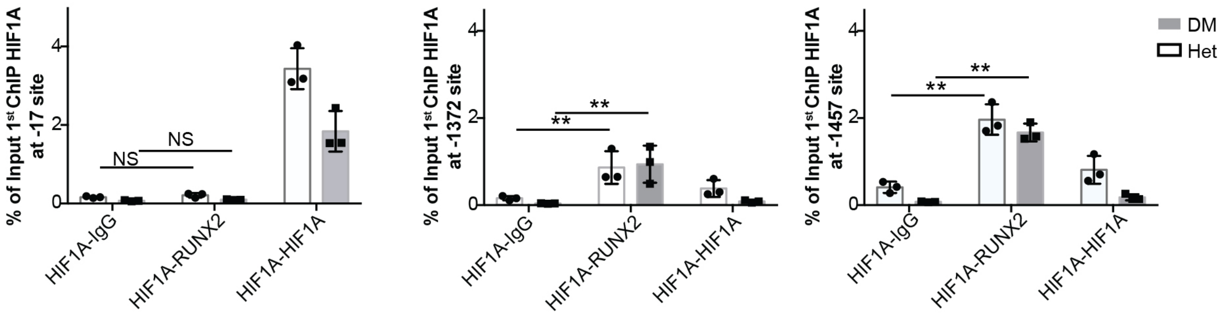

Supplementary Fig. 22 ChIP-RE-ChIP using 1st anti-HIF1A ChIP as input to validate the co-binding of RUNX2 in the promoter of *Vegfa*. Data were presented as means ± SEMs. An unpaired t-test was performed. **: *P*<0.01. NS: not significant.

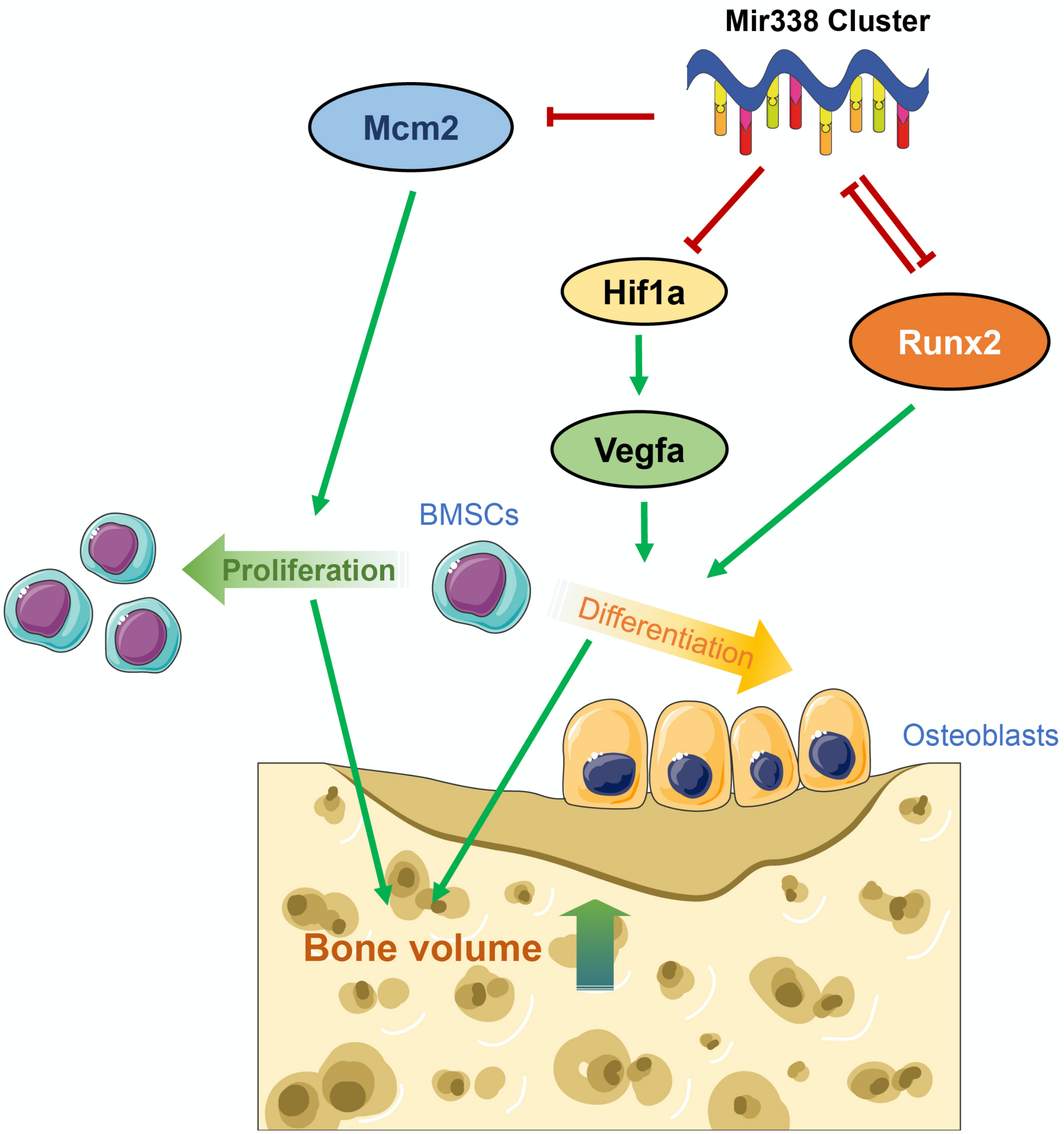

Supplementary Fig. 23 Graphical Abstract Schematic showing the cellular and molecular machoism on the knockout of Mir338 rescues bone defect caused by Runx2 haploinsufficiency.

Supplementary Table 1 Marker genes for different cluster in the merged scRNA-seq profiles of BMSCs with different genotypes

p_val	avg_log2FC	pct.1	pct.2	p_val_adj	cluster	gene
2.10E-230	1.25968356	0.999	0.974	5.08E-226	0	Ftl1
2.53E-163	1.03878637	1	0.997	6.13E-159	0	Fth1
1.06E-162	1.11133133	1	0.994	2.57E-158	0	Tmsb4x
2.66E-126	1.22659682	0.979	0.919	6.46E-122	0	Lyz2
2.88E-117	1.06966413	0.999	0.92	6.99E-113	0	Rps29
8.69E-117	1.20767034	0.86	0.57	2.11E-112	0	Fcer1g
3.62E-106	1.11206834	0.896	0.662	8.77E-102	0	Tyrobp
2.14E-85	0.82998758	0.943	0.799	5.19E-81	0	Fau
1.84E-78	0.94414535	0.811	0.581	4.45E-74	0	Ucp2
7.11E-77	1.05466005	0.818	0.614	1.72E-72	0	Selenop
2.46E-65	0.95779743	0.654	0.365	5.96E-61	0	Fyb
2.58E-65	0.76680669	0.877	0.737	6.25E-61	0	Arpc1b
2.58E-63	0.67514965	0.881	0.759	6.25E-59	0	Akr1a1
2.52E-59	0.81023226	0.576	0.292	6.11E-55	0	Ptpn18
1.46E-58	0.78698406	0.871	0.712	3.54E-54	0	Efhd2
2.17E-58	0.95470768	0.625	0.362	5.27E-54	0	Lst1
1.69E-57	0.53683224	0.993	0.918	4.09E-53	0	Rpl37a
2.15E-57	0.79175513	0.812	0.612	5.21E-53	0	Cotl1
5.32E-54	0.9688547	0.595	0.37	1.29E-49	0	Ninj1
7.35E-53	0.56949055	0.927	0.854	1.78E-48	0	Arpc2

3.42E-52	0.89195918	0.709	0.503	8.30E-48	0	Igfbp4
4.39E-52	0.87358308	0.744	0.578	1.06E-47	0	Cat
1.05E-51	1.30238161	0.7	0.476	2.54E-47	0	Pf4
5.70E-49	0.65542392	0.839	0.703	1.38E-44	0	Gabarap
5.82E-48	0.80819214	0.562	0.323	1.41E-43	0	Clec4d
7.06E-48	0.77088258	0.559	0.317	1.71E-43	0	Cd68
7.25E-47	0.78338501	0.777	0.581	1.76E-42	0	C1qb
8.38E-47	0.45406987	0.973	0.928	2.03E-42	0	Prdx1
3.31E-46	0.79453946	0.613	0.382	8.03E-42	0	Alox5ap
1.50E-45	0.70348511	0.754	0.558	3.65E-41	0	Tpd52
5.56E-45	0.60530194	0.906	0.629	1.35E-40	0	Rps23
3.22E-44	0.71054599	0.878	0.632	7.80E-40	0	Rps27
1.50E-43	0.71981414	0.639	0.394	3.65E-39	0	Aif1
1.86E-43	0.59949231	0.899	0.861	4.51E-39	0	Itm2b
6.30E-43	0.97457852	0.567	0.361	1.53E-38	0	Ccl6
7.21E-43	0.77411124	0.535	0.297	1.75E-38	0	Spi1
8.48E-43	1.08635078	0.567	0.357	2.06E-38	0	Wfdc17
1.16E-42	0.80365728	0.543	0.309	2.81E-38	0	Cd52
5.56E-42	0.74541719	0.717	0.473	1.35E-37	0	Rpl10
1.38E-41	0.54663545	0.868	0.784	3.35E-37	0	Npc2
6.09E-41	0.62015176	0.78	0.564	1.48E-36	0	Igf1

8.57E-40	0.71969312	0.902	0.841	2.08E-35	0	Ctss
1.55E-39	0.62158945	0.583	0.348	3.75E-35	0	Fcgr3
3.05E-38	0.6604121	0.846	0.665	7.40E-34	0	Rps25
6.31E-38	0.775373	0.564	0.354	1.53E-33	0	Rab32
6.58E-38	0.52187568	0.987	0.891	1.59E-33	0	Rps21
6.97E-38	0.70959876	0.373	0.177	1.69E-33	0	Rac2
1.32E-37	0.59249869	0.832	0.718	3.20E-33	0	Maf
3.40E-37	0.32190335	0.997	0.966	8.24E-33	0	Tpt1
6.51E-37	0.54015252	0.308	0.127	1.58E-32	0	AB124611
1.20E-36	0.62953622	0.655	0.455	2.90E-32	0	Trem2
1.66E-36	0.62555103	0.744	0.653	4.02E-32	0	Arpc3
1.88E-36	0.65422808	0.511	0.29	4.57E-32	0	Ms4a6c
2.69E-36	0.715722	0.678	0.485	6.51E-32	0	Ms4a7
7.33E-35	0.58352606	0.825	0.69	1.78E-30	0	C1qc
9.50E-34	0.48742529	0.874	0.791	2.30E-29	0	Taldo1
9.70E-34	0.58784452	0.905	0.688	2.35E-29	0	Rpl37
1.17E-33	0.47897956	0.992	0.963	2.84E-29	0	Apoe
2.66E-33	0.59323536	0.464	0.257	6.44E-29	0	Cyth4
3.81E-33	0.62384842	0.405	0.212	9.24E-29	0	Gngt2
3.06E-31	0.49185652	0.819	0.734	7.41E-27	0	Capza2
1.09E-30	0.40570727	0.959	0.891	2.65E-26	0	Cfl1
3.56E-30	0.63905851	0.669	0.546	8.64E-26	0	Wwp1
4.35E-30	0.58834463	0.472	0.27	1.06E-25	0	Irf8

7.93E-30	0.46346285	0.964	0.818	1.92E-25	0	Rps28
1.00E-29	0.6247062	0.696	0.614	2.43E-25	0	Hexa
3.45E-29	0.62515793	0.636	0.451	8.37E-25	0	Pla2g7
5.04E-29	0.61974975	0.366	0.195	1.22E-24	0	Gpsm3
5.53E-29	0.37343535	0.91	0.836	1.34E-24	0	Cdc42
7.32E-29	0.53337116	0.741	0.519	1.78E-24	0	Rpl17
4.40E-28	0.58560495	0.611	0.434	1.07E-23	0	Pid1
7.24E-28	0.46870352	0.825	0.74	1.76E-23	0	H3f3a
7.68E-28	0.69270073	0.552	0.403	1.86E-23	0	Blvrb
1.99E-27	0.65794111	0.679	0.553	4.82E-23	0	Gng5
2.85E-27	0.62572703	0.786	0.699	6.92E-23	0	Cd36
3.69E-27	0.72784181	0.396	0.245	8.94E-23	0	Rgs10
4.93E-27	0.51834264	0.256	0.113	1.20E-22	0	Gpr65
6.29E-27	0.62433718	0.683	0.572	1.52E-22	0	Lipa
9.22E-27	0.46884984	0.466	0.271	2.24E-22	0	Gmfg
1.72E-26	0.62663914	0.641	0.539	4.16E-22	0	Rab5c
3.53E-26	0.38912456	0.934	0.755	8.57E-22	0	Rpl35a
5.87E-26	0.47567384	0.503	0.314	1.42E-21	0	Hcls1
7.94E-26	0.52669177	0.703	0.601	1.92E-21	0	Sod1
1.45E-25	0.3565861	0.936	0.87	3.51E-21	0	BC005537
1.74E-25	0.46473131	0.958	0.868	4.21E-21	0	Rpl23
2.40E-25	0.57109683	0.437	0.261	5.83E-21	0	Ncf1

3.93E-25	0.45035461	0.934	0.817	9.53E-21	0	Rps27a
6.54E-25	0.406284	0.936	0.814	1.59E-20	0	Ybx1
7.78E-25	0.50020736	0.55	0.371	1.89E-20	0	Coro1a
8.14E-24	0.40803341	0.81	0.732	1.97E-19	0	Gnai2
1.10E-23	0.575182	0.669	0.588	2.67E-19	0	Clta
1.69E-23	0.36437477	0.943	0.918	4.11E-19	0	Ctsd
2.24E-23	0.53667804	0.695	0.549	5.43E-19	0	Mafb
2.40E-23	0.52729177	0.644	0.55	5.81E-19	0	Rhog
2.82E-23	0.51115542	0.824	0.649	6.85E-19	0	Rps11
3.64E-23	0.62831494	0.755	0.58	8.83E-19	0	Rpl30
3.69E-23	0.57688593	0.378	0.218	8.95E-19	0	AI662270
6.44E-23	0.47555875	0.745	0.678	1.56E-18	0	Adipor1
8.85E-23	0.54458179	0.675	0.584	2.15E-18	0	Pitpna
1.11E-22	0.50809	0.471	0.3	2.70E-18	0	Ncf2
1.57E-22	0.62075584	0.566	0.467	3.80E-18	0	Slc48a1
2.94E-22	0.46457465	0.59	0.422	7.12E-18	0	C3ar1
4.11E-22	0.5599688	0.51	0.367	9.97E-18	0	Lyn
6.52E-22	0.50197645	0.312	0.164	1.58E-17	0	Pou2f2
7.64E-22	0.46647634	0.577	0.416	1.85E-17	0	Csf1r
7.65E-22	0.39408071	0.867	0.816	1.86E-17	0	Cstb
7.88E-22	0.49840341	0.676	0.537	1.91E-17	0	Arhgdib
9.36E-22	0.41041974	0.962	0.832	2.27E-17	0	Rpl39
9.73E-22	0.48517623	0.751	0.528	2.36E-17	0	Rpl36a

1.27E-21	0.47967689	0.398	0.242	3.09E-17	0	Slfn2
1.77E-21	0.5454395	0.475	0.326	4.28E-17	0	Cd300c2
1.80E-21	0.53282631	0.352	0.201	4.37E-17	0	Myo1f
1.80E-21	0.29121676	1	0.951	4.37E-17	0	Eef1a1
3.95E-21	0.46809872	0.338	0.19	9.57E-17	0	Pycard
5.71E-21	0.37184373	0.333	0.183	1.39E-16	0	Dock2
1.11E-20	0.47893569	0.868	0.706	2.69E-16	0	Rps16
1.26E-20	0.3521396	0.898	0.788	3.06E-16	0	Lcp1
1.26E-20	0.52148899	0.672	0.608	3.06E-16	0	Bri3
1.57E-20	0.57046198	0.613	0.521	3.80E-16	0	Rab8b
1.99E-20	0.41373434	0.818	0.761	4.84E-16	0	Creg1
2.81E-20	0.49862469	0.35	0.203	6.81E-16	0	Arhgap30
2.97E-20	0.48623473	0.725	0.669	7.21E-16	0	Ptbp3
4.12E-20	0.49338721	0.588	0.459	9.99E-16	0	Glul
4.84E-20	0.28417325	0.966	0.936	1.17E-15	0	B2m
7.21E-20	0.5966139	0.622	0.548	1.75E-15	0	Gsr
9.62E-20	0.57823198	0.458	0.331	2.33E-15	0	Gng2
9.67E-20	0.57590351	0.556	0.455	2.34E-15	0	Ehd4
1.50E-19	0.39651661	0.755	0.702	3.64E-15	0	Snx3
1.59E-19	0.46546808	0.671	0.606	3.86E-15	0	Gltp
2.13E-19	0.68428469	0.472	0.365	5.16E-15	0	Rap2b
2.47E-19	0.49472186	0.345	0.204	5.99E-15	0	Syngr1
3.11E-19	0.46987559	0.845	0.809	7.53E-15	0	Lgals3

3.58E-19	0.51118018	0.672	0.632	8.69E-15	0	Fxyd5
5.31E-19	0.37239584	0.95	0.843	1.29E-14	0	Rpl38
6.57E-19	0.43260788	0.549	0.389	1.59E-14	0	Clec4a1
6.72E-19	0.70597975	0.387	0.248	1.63E-14	0	F13a1
1.04E-18	0.28584046	0.882	0.816	2.53E-14	0	Oaz1
1.25E-18	0.30095611	0.741	0.513	3.03E-14	0	Rpl35
1.48E-18	0.36280862	0.787	0.736	3.58E-14	0	Atp6v1g1
1.77E-18	0.50554399	0.571	0.491	4.30E-14	0	Aldh2
2.60E-18	0.43327303	0.51	0.367	6.31E-14	0	Tm6sf1
3.49E-18	0.48581752	0.825	0.784	8.46E-14	0	Gpnmb
3.93E-18	0.63675184	0.423	0.309	9.52E-14	0	Tnfaip8
4.41E-18	0.4437424	0.599	0.452	1.07E-13	0	Cd84
5.08E-18	0.4518794	0.773	0.629	1.23E-13	0	Rpl26
5.48E-18	0.38283209	0.388	0.24	1.33E-13	0	Pld4
5.67E-18	0.35198745	0.776	0.564	1.38E-13	0	Rps17
6.94E-18	0.44452428	0.433	0.284	1.68E-13	0	Clec4a2
9.55E-18	0.47365605	0.371	0.235	2.32E-13	0	Slc11a1
1.05E-17	0.39765462	0.493	0.344	2.55E-13	0	Vsir
1.22E-17	0.39001018	0.394	0.247	2.96E-13	0	Lmo2
1.34E-17	0.47315567	0.619	0.552	3.24E-13	0	Grb2
1.48E-17	0.36338846	0.783	0.63	3.59E-13	0	Rpl7a
1.66E-17	0.54806366	0.457	0.322	4.03E-13	0	Clec4n

2.41E-17	0.43590765	0.291	0.165	5.85E-13	0	Itga4
2.56E-17	0.62452156	0.574	0.485	6.20E-13	0	Dab2
4.16E-17	0.31237257	0.864	0.817	1.01E-12	0	Arpc5
8.09E-17	0.44331365	0.452	0.312	1.96E-12	0	Cfp
8.56E-17	0.38870378	0.72	0.531	2.08E-12	0	Rps15a
9.22E-17	0.40892658	0.506	0.361	2.23E-12	0	Nrros
1.06E-16	0.41984535	0.671	0.609	2.56E-12	0	Fam49b
1.14E-16	0.53833543	0.578	0.515	2.75E-12	0	Brk1
1.67E-16	0.4532314	0.639	0.577	4.04E-12	0	Mob1a
1.94E-16	0.4621317	0.476	0.346	4.71E-12	0	Arrb2
2.56E-16	0.36738262	0.676	0.554	6.22E-12	0	Cd53
3.90E-16	0.34746197	0.711	0.631	9.46E-12	0	Sh3bgrl
4.17E-16	0.36807754	0.682	0.633	1.01E-11	0	Rab7
4.76E-16	0.54267759	0.58	0.503	1.15E-11	0	Ubl5
4.78E-16	0.399997	0.689	0.63	1.16E-11	0	Ppp1r18
6.21E-16	0.3200011	0.875	0.811	1.51E-11	0	H3f3b
9.17E-16	0.38343031	0.728	0.559	2.22E-11	0	Rpl34
9.87E-16	0.34052887	0.782	0.729	2.39E-11	0	Gdi2
1.00E-15	0.45934442	0.507	0.375	2.43E-11	0	C5ar1
1.11E-15	0.2819512	0.829	0.775	2.68E-11	0	Dazap2
1.94E-15	0.45364465	0.686	0.601	4.70E-11	0	AC121965.1
1.98E-15	0.29282703	0.972	0.865	4.81E-11	0	Rps12
2.03E-15	0.45783158	0.443	0.308	4.91E-11	0	Apobec1

2.19E-15	0.4470138<br>9	0.699	0.669	5.30E-11	0	Cst3
2.43E-15	0.3454582<br>2	0.754	0.575	5.89E-11	0	Rps19
3.78E-15	0.3553450<br>2	0.724	0.676	9.17E-11	0	Cyba
3.91E-15	0.4495489<br>2	0.443	0.325	9.48E-11	0	Irf5
5.01E-15	0.2617111<br>5	0.839	0.793	1.21E-10	0	Mbnl1
6.27E-15	0.4804805<br>3	0.574	0.445	1.52E-10	0	Cox7c
1.02E-14	0.3858654<br>2	0.431	0.3	2.47E-10	0	Ms4a6d
1.08E-14	0.3577866<br>9	0.873	0.75	2.61E-10	0	Rpl22
1.11E-14	0.4893873<br>6	0.564	0.471	2.70E-10	0	Trf
1.13E-14	0.3158277<br>2	0.766	0.718	2.73E-10	0	Cap1
1.23E-14	0.3319127<br>2	0.608	0.467	2.99E-10	0	Ly86
1.35E-14	0.3851399<br>4	0.646	0.564	3.28E-10	0	Rac1
1.61E-14	0.2644380<br>6	0.769	0.581	3.90E-10	0	Rpl6
1.74E-14	0.3646248<br>3	0.776	0.697	4.22E-10	0	Cox8a
1.84E-14	0.5180263<br>1	0.44	0.35	4.46E-10	0	Gpx4
1.90E-14	0.3172024	0.847	0.794	4.61E-10	0	Ptp4a2
1.98E-14	0.6236471<br>1	0.545	0.479	4.80E-10	0	Gclm
2.03E-14	0.5436228<br>3	0.555	0.491	4.93E-10	0	Gstm1
2.17E-14	0.3669879<br>6	0.681	0.639	5.27E-10	0	Grk2
2.41E-14	0.3700931<br>8	0.332	0.206	5.84E-10	0	Rassf4
2.58E-14	0.3259087<br>9	0.711	0.544	6.25E-10	0	Rpl9
3.34E-14	0.3466084<br>1	0.81	0.661	8.10E-10	0	Rpl18a

3.47E-14	0.3896742	0.69	0.629	8.40E-10	0	Zfp36l2
3.48E-14	0.42567406	0.65	0.593	8.45E-10	0	Rab14
3.95E-14	0.3690429	0.64	0.51	9.58E-10	0	Rpl5
4.11E-14	0.29606567	0.853	0.78	9.96E-10	0	Laptm5
4.42E-14	0.41994562	0.532	0.417	1.07E-09	0	Celf2
4.77E-14	0.47045332	0.557	0.474	1.16E-09	0	Myl12b
4.84E-14	0.5501187	0.466	0.389	1.17E-09	0	Plekho1
8.56E-14	0.37919084	0.693	0.659	2.08E-09	0	Vamp8
9.82E-14	0.34859776	0.49	0.356	2.38E-09	0	Inpp5d
1.00E-13	0.39733344	0.668	0.617	2.44E-09	0	Plin2
1.05E-13	0.30154823	0.272	0.158	2.54E-09	0	Hcst
1.09E-13	0.34883908	0.444	0.315	2.64E-09	0	Ly9
1.40E-13	0.32201964	0.751	0.615	3.39E-09	0	Rpl29
1.64E-13	0.40023636	0.415	0.289	3.97E-09	0	Ptpn6
2.14E-13	0.53552643	0.465	0.383	5.18E-09	0	Cndp2
2.34E-13	0.46144542	0.359	0.247	5.66E-09	0	Fgd4
2.38E-13	0.4281835	0.623	0.532	5.76E-09	0	Tomm7
2.72E-13	0.42073723	0.521	0.433	6.59E-09	0	Gm2a
6.93E-13	0.33248631	0.339	0.22	1.68E-08	0	Lcp2
7.68E-13	0.28658682	0.878	0.777	1.86E-08	0	Eif1
9.96E-13	0.37517179	0.31	0.198	2.42E-08	0	Arhgap9
1.06E-12	0.36340083	0.584	0.481	2.56E-08	0	Smpdl3a
1.08E-12	0.44804446	0.483	0.388	2.61E-08	0	Pdxk

1.23E-12	0.47663368	0.689	0.595	2.98E-08	0	Atp5e
1.33E-12	0.38294985	0.375	0.261	3.22E-08	0	Rab3il1
1.66E-12	0.33733699	0.331	0.215	4.03E-08	0	Lrrc25
2.21E-12	0.32692024	0.333	0.217	5.36E-08	0	Nckap1l
2.94E-12	0.31891008	0.263	0.159	7.13E-08	0	Clec4a3
3.13E-12	0.45621215	0.571	0.494	7.59E-08	0	Atp5h
3.67E-12	0.40281165	0.375	0.264	8.90E-08	0	Arhgap18
5.21E-12	0.46190761	0.374	0.263	1.26E-07	0	Atp6v0d2
5.28E-12	0.48503097	0.609	0.556	1.28E-07	0	Atox1
5.69E-12	0.40319783	0.602	0.48	1.38E-07	0	Rpl31
6.35E-12	0.30434801	0.667	0.566	1.54E-07	0	Cybb
7.14E-12	0.40732563	0.59	0.526	1.73E-07	0	Idh1
9.09E-12	0.34509807	0.797	0.793	2.20E-07	0	Mpeg1
1.03E-11	0.52865035	0.48	0.414	2.49E-07	0	Stk17b
1.04E-11	0.32252698	0.331	0.217	2.52E-07	0	Hpgds
1.10E-11	0.29292948	0.727	0.712	2.66E-07	0	Atp6v1a
1.26E-11	0.33827042	0.699	0.682	3.06E-07	0	Tnfaip2
1.80E-11	0.40889859	0.681	0.643	4.36E-07	0	Sh3bgrl3
1.95E-11	0.26593134	0.968	0.967	4.74E-07	0	Psap
2.51E-11	0.39803097	0.634	0.6	6.08E-07	0	Atp6v1e1
2.64E-11	0.33056381	0.399	0.291	6.40E-07	0	Rassf2
2.65E-11	0.3577156	0.353	0.246	6.42E-07	0	Cd300lb

3.01E-11	0.4611183 4	0.419	0.331	7.30E-07	0	Rab7b
3.03E-11	0.3150757 2	0.686	0.605	7.34E-07	0	C1qa
3.58E-11	0.3370505 3	0.636	0.493	8.69E-07	0	Rps7
4.67E-11	0.3112098 5	0.709	0.628	1.13E-06	0	2010107E04Ri k
4.68E-11	0.3022636 5	0.261	0.159	1.14E-06	0	Ikzf1
4.72E-11	0.3406952 2	0.648	0.622	1.14E-06	0	Cox7a2l
4.76E-11	0.4561088	0.384	0.294	1.15E-06	0	Gnpda1
6.98E-11	0.3781547 6	0.571	0.53	1.69E-06	0	Zfp706
8.90E-11	0.3888189 9	0.661	0.613	2.16E-06	0	Zfp36l1
9.34E-11	0.3428274	0.503	0.388	2.26E-06	0	Msr1
1.09E-10	0.4562865 7	0.515	0.473	2.65E-06	0	Msrb1
1.19E-10	0.2740273 6	0.311	0.206	2.89E-06	0	Cd200r1
1.22E-10	0.2924005 2	0.706	0.665	2.97E-06	0	Atp6v1b2
1.55E-10	0.2766208 7	0.966	0.905	3.76E-06	0	Rps24
1.67E-10	0.4889003 8	0.461	0.397	4.05E-06	0	Sash1
3.09E-10	0.3714911 2	0.616	0.564	7.49E-06	0	Slc43a2
3.65E-10	0.2563818 6	0.275	0.175	8.85E-06	0	Trim30a
4.34E-10	0.2546646 9	0.291	0.19	1.05E-05	0	Tlr13
4.91E-10	0.3900781 5	0.587	0.55	1.19E-05	0	Ndufa6
5.06E-10	0.2667522 8	0.394	0.288	1.23E-05	0	Syk
5.92E-10	0.3429222 9	0.577	0.533	1.44E-05	0	Ubl3
1.00E-09	0.2948031 3	0.627	0.491	2.42E-05	0	Rpl12

1.17E-09	0.2927489<br>1	0.737	0.644	2.84E-05	0	Cox6b1
1.23E-09	0.2549800<br>6	0.35	0.245	2.99E-05	0	Entpd1
1.40E-09	0.3000851<br>6	0.683	0.66	3.40E-05	0	Irf2bp2
1.62E-09	0.2769682<br>7	0.548	0.446	3.92E-05	0	Plek
2.34E-09	0.3627020<br>4	0.412	0.317	5.67E-05	0	Ccr1
2.37E-09	0.3118893<br>9	0.298	0.21	5.74E-05	0	Prex1
3.05E-09	0.3270379<br>7	0.629	0.611	7.40E-05	0	Ostf1
3.42E-09	0.3025557<br>5	0.637	0.611	8.28E-05	0	Pfdn5
4.95E-09	0.4356676<br>9	0.559	0.528	0.0001199<br>3	0	Ly6e
5.41E-09	0.2992844<br>9	0.646	0.635	0.0001311<br>2	0	Atp6v0b
5.55E-09	0.3829736<br>7	0.556	0.446	0.0001344<br>7	0	Rpl21
5.57E-09	0.3519249<br>6	0.597	0.559	0.0001349<br>6	0	Prdx5
6.07E-09	0.4002083<br>8	0.501	0.453	0.0001471<br>2	0	Rnf130
6.21E-09	0.2910918<br>1	0.571	0.531	0.0001506<br>7	0	Prkcd
6.51E-09	0.3346420<br>6	0.682	0.636	0.0001578<br>8	0	Cox5a
9.97E-09	0.2649020<br>2	0.419	0.316	0.0002418	0	Itgal
1.16E-08	0.3624734<br>7	0.604	0.484	0.0002803<br>4	0	Rps13
1.43E-08	0.2738350<br>7	0.262	0.175	0.0003464<br>4	0	Mertk
1.74E-08	0.4361681<br>3	0.681	0.588	0.0004213<br>7	0	Rpl11
1.87E-08	0.2826117<br>9	0.35	0.257	0.0004528<br>9	0	Prkcb
1.99E-08	0.3265097<br>3	0.57	0.543	0.0004832<br>3	0	Gna12

2.16E-08	0.25695209	0.374	0.286	0.00052482	0	Cd48
2.42E-08	0.29879337	0.682	0.646	0.00058746	0	Ppp1ca
2.50E-08	0.3066498	0.296	0.213	0.00060563	0	Txnip
2.72E-08	0.3777714	0.503	0.458	0.00065867	0	Fam174a
3.05E-08	0.4339193	0.455	0.415	0.0007394	0	Tgfb1
3.14E-08	0.39750192	0.55	0.518	0.00076116	0	Gas6
3.22E-08	0.34878095	0.522	0.475	0.00078195	0	Plekhb2
4.13E-08	0.39652193	0.529	0.509	0.00100073	0	Sptssa
4.41E-08	0.29447473	0.622	0.597	0.00107018	0	Unc93b1
4.45E-08	0.33484673	0.501	0.441	0.0010786	0	Cxcl16
5.97E-08	0.3711623	0.469	0.418	0.00144838	0	Pdlim4
7.67E-08	0.34685383	0.555	0.541	0.00185987	0	Tmem256
7.72E-08	0.31005732	0.569	0.536	0.00187154	0	Aes
9.16E-08	0.41520949	0.521	0.506	0.00222216	0	Usf2
1.10E-07	0.28430202	0.658	0.621	0.00265638	0	Rcbtb2
1.27E-07	0.29113389	0.619	0.555	0.00307534	0	Atp5f1
1.30E-07	0.54571992	0.342	0.281	0.00315782	0	Fam111a
1.44E-07	0.27764978	0.632	0.594	0.00348893	0	Ctsc
1.65E-07	0.39711885	0.499	0.452	0.00400138	0	Kctd12
1.85E-07	0.47047524	0.336	0.279	0.00448284	0	Hacd4
2.01E-07	0.29699985	0.669	0.606	0.0048653	0	Cox6c
2.07E-07	0.25181268	0.695	0.656	0.00501867	0	Pomp

2.47E-07	0.2750339	0.71	0.685	0.00599589	0	Actr3
2.54E-07	0.372561	0.672	0.63	0.00616989	0	Gpx1
2.60E-07	0.41435117	0.473	0.439	0.00629672	0	Arl4c
2.65E-07	0.27566712	0.594	0.58	0.00643343	0	Pgd
2.72E-07	0.36315939	0.403	0.345	0.00658682	0	Fnbp1
2.78E-07	0.41394121	0.437	0.373	0.00674625	0	Fcgr2b
5.49E-07	0.26999786	0.307	0.228	0.01330954	0	Clec12a
5.61E-07	0.28352695	0.571	0.535	0.0136022	0	Sub1
5.84E-07	0.43579882	0.482	0.457	0.01416671	0	Ctsh
6.30E-07	0.44629558	0.457	0.418	0.01528008	0	Ost4
6.40E-07	0.4852562	0.387	0.343	0.01552665	0	Snx10
6.61E-07	0.43874039	0.464	0.445	0.01602161	0	Rragc
7.58E-07	0.26480355	0.646	0.624	0.01837028	0	Hmox1
1.09E-06	0.25665869	0.259	0.191	0.02636584	0	Camk1d
1.21E-06	0.28883661	0.503	0.433	0.02943277	0	Cd300ld
1.74E-06	0.32114733	0.487	0.453	0.04213933	0	Dhrs3
1.88E-06	0.44043406	0.437	0.388	0.04547366	0	Tgm2
1.96E-06	0.37043776	0.382	0.335	0.04746367	0	Znrf2
2.11E-06	0.25044006	0.64	0.623	0.05123985	0	Picalm
2.25E-06	0.5472211	0.381	0.354	0.05457678	0	Psme1
2.38E-06	0.26014947	0.272	0.198	0.05782749	0	Nfam1
2.57E-06	0.2661935	0.667	0.635	0.0623944	0	Slc40a1

3.06E-06	0.29221109	0.64	0.586	0.07427654	0	Slc25a5
3.17E-06	0.36039489	0.62	0.591	0.07679925	0	Tma7
3.50E-06	0.30396708	0.482	0.461	0.08480717	0	Grina
3.54E-06	0.35209576	0.492	0.484	0.08593594	0	Tsc22d4
3.64E-06	0.28704453	0.515	0.495	0.08822079	0	Erp29
4.14E-06	0.36142403	0.469	0.434	0.10026793	0	Bst2
5.08E-06	0.45339935	0.443	0.43	0.12313723	0	Abi1
5.54E-06	0.29642851	0.573	0.474	0.13429046	0	Rpl36
6.86E-06	0.38835037	0.262	0.211	0.16636224	0	Aldh3b1
7.28E-06	0.56623465	0.294	0.245	0.176435	0	Ccl9
8.37E-06	0.47155669	0.296	0.26	0.20301613	0	Scp2
8.79E-06	0.29518504	0.445	0.394	0.21307958	0	Tmem106a
9.52E-06	0.3029277	0.472	0.459	0.23077927	0	Lamtor4
9.61E-06	0.32378962	0.493	0.461	0.23294664	0	Mt1
1.07E-05	0.34553799	0.473	0.469	0.25891372	0	Asah1
1.11E-05	0.25721054	0.471	0.425	0.26860212	0	Cerk
1.17E-05	0.30212477	0.552	0.551	0.28308342	0	M6pr
1.19E-05	0.36461842	0.424	0.406	0.28914683	0	Fuca1
1.28E-05	0.37515906	0.429	0.411	0.30927736	0	Rnf7
1.36E-05	0.29940014	0.508	0.485	0.32963918	0	Clic1
1.47E-05	0.28086943	0.266	0.207	0.35573274	0	Tec

1.69E-05	0.43829947	0.394	0.377	0.40904674	0	Arl8a
1.73E-05	0.274333	0.597	0.607	0.41838631	0	Cmpk1
1.78E-05	0.26792664	0.314	0.255	0.43166115	0	Bcl2l11
1.79E-05	0.57593986	0.338	0.305	0.43463335	0	S100a1
2.07E-05	0.37650184	0.268	0.218	0.50191953	0	Zfp710
2.18E-05	0.35183035	0.373	0.33	0.52830713	0	Mgst1
2.88E-05	0.3344455	0.48	0.474	0.69869176	0	Sgpl1
3.17E-05	0.36147104	0.52	0.512	0.76775916	0	Plekho2
3.18E-05	0.37501211	0.433	0.422	0.77000642	0	Litaf
3.25E-05	0.33778186	0.394	0.346	0.78725864	0	Apbb1ip
3.45E-05	0.36604187	0.466	0.463	0.83702083	0	Ppp2r5c
3.95E-05	0.46477646	0.331	0.289	0.95828209	0	Ogfrl1
4.41E-05	0.32110967	0.641	0.609	1	0	Basp1
4.87E-05	0.26029713	0.359	0.301	1	0	Sesn1
4.90E-05	0.27154786	0.552	0.543	1	0	Vapa
5.27E-05	0.47519619	0.375	0.358	1	0	Limd2
8.25E-05	0.44796256	0.349	0.325	1	0	Fam96a
8.29E-05	0.25105245	0.585	0.588	1	0	Qk
0.00010105	0.34373125	0.359	0.34	1	0	Cmtm7
0.00010217	0.33640585	0.273	0.225	1	0	Bhlhe41
0.0001158	0.31924876	0.438	0.42	1	0	Rab31

0.00012421	0.26081131	0.548	0.52	1	0	Cox7a2
0.000143	0.29202218	0.471	0.466	1	0	Rnf13
0.0001473	0.25131849	0.587	0.593	1	0	Wdr26
0.00015619	0.30993624	0.492	0.497	1	0	Atp6v0d1
0.00015835	0.40350387	0.326	0.296	1	0	Card19
0.0001628	0.31865962	0.438	0.419	1	0	Skap2
0.00019657	0.29965502	0.464	0.461	1	0	Vps35
0.00021639	0.35973372	0.268	0.231	1	0	Blvra
0.00025895	0.25169129	0.466	0.445	1	0	Wipf1
0.0002747	0.25760798	0.256	0.206	1	0	Ccl3
0.0003689	0.27927454	0.539	0.525	1	0	St13
0.00039977	0.36182126	0.378	0.365	1	0	Ap1s2
0.00042765	0.35934578	0.266	0.229	1	0	Cyth1
0.00047935	0.39288694	0.273	0.243	1	0	Psmb8
0.00049256	0.27481855	0.594	0.57	1	0	Cops9
0.00049939	0.42311488	0.363	0.352	1	0	1500011K16Rik
0.00056709	0.26335682	0.5	0.508	1	0	Lamtor1
0.00082991	0.26676888	0.535	0.493	1	0	Bola2
0.00085238	0.26108175	0.517	0.528	1	0	Ap1b1
0.00090288	0.32338067	0.27	0.242	1	0	Tnfsf12
0.00090687	0.29876856	0.486	0.474	1	0	Fos

0.00094447	0.40154986	0.338	0.314	1	0	Mef2c
0.00107669	0.40490763	0.373	0.37	1	0	Akirin1
0.00118457	0.30998571	0.454	0.452	1	0	Atp6v1c1
0.00133866	0.35387449	0.452	0.465	1	0	Capg
0.00176477	0.31009477	0.303	0.261	1	0	Ptpre
0.00187249	0.31091896	0.375	0.364	1	0	Ypel3
0.00188235	0.32004655	0.41	0.407	1	0	5031439G07Rik
0.00189017	0.3837832	0.345	0.341	1	0	Tprgl
0.00235399	0.32213533	0.447	0.441	1	0	Ier3ip1
0.00286873	0.42302935	0.328	0.324	1	0	Pet100
0.00287972	0.56719947	0.31	0.303	1	0	Glrx
0.00289948	0.25511394	0.49	0.516	1	0	Gns
0.00302063	0.29262558	0.501	0.517	1	0	Ubxn1
0.00303842	0.27852505	0.513	0.525	1	0	Usp25
0.00331717	0.27080222	0.459	0.456	1	0	Hpcal1
0.00349313	0.3113266	0.283	0.252	1	0	Fnip2
0.00353135	0.27347454	0.403	0.406	1	0	Mia2
0.00387665	0.3589954	0.395	0.401	1	0	Ptpn1
0.00466844	0.30026836	0.45	0.468	1	0	Stx7
0.00486422	0.37056733	0.37	0.376	1	0	Gnaq
0.00508487	0.42632193	0.304	0.297	1	0	Taok3

0.00513707	0.38081332	0.312	0.302	1	0	Ncoa4
0.005463	0.33571409	0.396	0.406	1	0	Lamtor2
0.0059442	0.28207006	0.392	0.381	1	0	Kdm7a
0.00635051	0.37022462	0.321	0.311	1	0	Irf2
0.00672544	0.32177926	0.402	0.415	1	0	Mapkapk2
0.00680039	0.34406476	0.415	0.435	1	0	Ifitm2
0.00685627	0.32602041	0.465	0.465	1	0	Uqcr10
0.00691629	0.26733443	0.415	0.418	1	0	Ppp1r9b
0.00756542	0.28328466	0.419	0.393	1	0	Mrpl52
0.00765056	0.40009449	0.359	0.368	1	0	Hsbp1
0.00781051	0.3893734	0.326	0.326	1	0	Mkrn1
0.00821318	0.28161112	0.349	0.347	1	0	Tmem55b
0.0082563	0.25463488	0.276	0.248	1	0	Evi2a
0.00891928	0.25210151	0.252	0.349	1	0	Sod2
0.00933188	0.36157485	0.283	0.273	1	0	Wbp2
0.00980004	0.30519765	0.385	0.39	1	0	Ndufa1
3.83E-136	1.14054669	0.955	0.765	9.29E-132	1	Laptm5
2.36E-129	1.06226637	0.956	0.826	5.72E-125	1	Grn
4.60E-126	1.06975447	0.96	0.856	1.12E-121	1	Lgmn
1.49E-119	0.98844168	0.994	0.963	3.62E-115	1	Apoe
2.32E-115	0.86150964	0.993	0.963	5.62E-111	1	Psap

1.45E-110	0.87421188	0.996	0.975	3.52E-106	1	Ctsb
2.20E-107	1.28959951	0.782	0.442	5.35E-103	1	Ly86
1.68E-97	1.03935003	0.9	0.708	4.07E-93	1	Maf
2.25E-96	0.75207445	0.979	0.934	5.46E-92	1	B2m
6.00E-93	0.89605003	0.946	0.822	1.46E-88	1	H2-K1
1.64E-89	0.88466512	0.945	0.781	3.97E-85	1	Lcp1
1.37E-84	0.97314562	0.888	0.719	3.32E-80	1	Adgre1
5.37E-84	0.85430585	0.929	0.774	1.30E-79	1	Mpeg1
9.24E-83	0.74652717	0.942	0.837	2.24E-78	1	Lamp1
1.36E-82	0.73854702	0.977	0.92	3.30E-78	1	Lyz2
4.28E-82	0.8760375	0.927	0.782	1.04E-77	1	Sirpa
6.30E-82	0.96574904	0.853	0.714	1.53E-77	1	Serinc3
1.16E-81	1.0514259	0.864	0.703	2.82E-77	1	Mrc1
2.19E-76	0.71127956	0.939	0.855	5.32E-72	1	Itm2b
5.39E-76	1.06852837	0.751	0.497	1.31E-71	1	Igfbp4
7.06E-72	0.7584754	0.958	0.867	1.71E-67	1	BC005537
2.07E-70	0.87584511	0.873	0.683	5.03E-66	1	C1qc
3.79E-69	1.08519715	0.645	0.375	9.19E-65	1	Clec4a1
3.34E-68	0.77788672	0.917	0.746	8.10E-64	1	Creg1
7.58E-65	0.88797639	0.815	0.615	1.84E-60	1	Selenop
3.70E-64	0.77475022	0.876	0.768	8.96E-60	1	Dazap2
1.12E-63	0.60824893	0.98	0.912	2.72E-59	1	Ctsd
4.33E-62	0.76446339	0.88	0.775	1.05E-57	1	Arpc4

1.93E-57	0.95099837	0.791	0.589	4.68E-53	1	C1qa
3.21E-55	0.89144285	0.737	0.543	7.78E-51	1	Cd93
2.78E-54	0.87054721	0.78	0.606	6.75E-50	1	Itgam
7.40E-54	1.00189919	0.734	0.58	1.79E-49	1	Ctsc
1.19E-53	0.47978388	0.979	0.953	2.89E-49	1	Rps9
1.11E-52	0.84091284	0.706	0.481	2.69E-48	1	Ms4a7
3.66E-52	0.90743062	0.708	0.542	8.87E-48	1	Ftl1-ps1
1.03E-50	0.63345242	0.888	0.772	2.49E-46	1	H2-D1
1.10E-50	0.54526992	0.946	0.835	2.67E-46	1	Ctss
1.13E-47	0.78469297	0.758	0.648	2.74E-43	1	Tapbp
2.67E-47	0.72198233	0.774	0.581	6.47E-43	1	C1qb
5.70E-47	0.72055128	0.773	0.55	1.38E-42	1	Cybb
6.36E-47	0.88835782	0.708	0.55	1.54E-42	1	Slc43a2
6.52E-47	0.86678373	0.681	0.513	1.58E-42	1	Cd300a
4.12E-46	0.61525542	0.874	0.772	1.00E-41	1	Tmbim6
4.29E-45	0.70569719	0.831	0.693	1.04E-40	1	Cd36
5.29E-45	0.90085037	0.648	0.459	1.28E-40	1	Trf
4.50E-43	0.81761852	0.715	0.584	1.09E-38	1	Unc93b1
8.15E-43	0.68282889	0.785	0.69	1.98E-38	1	Tmem50a
8.50E-43	0.87961563	0.626	0.448	2.06E-38	1	Stab1
1.13E-41	0.79494162	0.725	0.612	2.74E-37	1	Rcbtb2

1.17E-41	0.75036963	0.739	0.634	2.83E-37	1	Atp2b1
1.27E-41	0.69027126	0.766	0.566	3.09E-37	1	Igf1
1.67E-39	0.52559032	0.884	0.814	4.05E-35	1	Arpc5
5.10E-39	0.53706298	0.9	0.796	1.24E-34	1	Marcks
1.51E-38	0.8121639	0.579	0.387	3.67E-34	1	Alox5ap
3.70E-38	0.55420802	0.887	0.82	8.96E-34	1	Sat1
6.89E-38	0.71168762	0.719	0.548	1.67E-33	1	Cd53
7.46E-37	0.93685283	0.506	0.315	1.81E-32	1	Clec4n
1.44E-36	0.34359046	0.966	0.929	3.50E-32	1	Prdx1
3.21E-36	0.5595376	0.871	0.788	7.78E-32	1	Mbnl1
1.84E-35	0.82205261	0.456	0.273	4.46E-31	1	Gmfg
5.19E-35	0.78290774	0.585	0.415	1.26E-30	1	Csf1r
5.28E-35	0.51224277	0.897	0.834	1.28E-30	1	Sdcbp
1.50E-33	0.86648129	0.494	0.313	3.63E-29	1	Ccr5
4.13E-33	0.8062761	0.602	0.459	1.00E-28	1	Fermt3
5.14E-33	0.82496497	0.579	0.432	1.25E-28	1	Fli1
2.33E-32	0.64650687	0.726	0.654	5.65E-28	1	Irf2bp2
4.14E-32	0.67086817	0.672	0.537	1.00E-27	1	Arhgdib
1.54E-31	0.78832441	0.475	0.309	3.74E-27	1	Cfp
1.89E-31	0.77488454	0.672	0.575	4.58E-27	1	Il10rb
2.90E-31	0.61941904	0.736	0.625	7.04E-27	1	Slc40a1
5.49E-31	0.7017302	0.689	0.602	1.33E-26	1	Cebpa
7.80E-31	0.948197	0.581	0.462	1.89E-26	1	Gpx3

3.96E-30	0.79592656	0.53	0.362	9.60E-26	1	Wfdc17
1.20E-29	0.67817707	0.576	0.403	2.91E-25	1	Aif1
1.24E-29	0.85347688	0.603	0.51	3.01E-25	1	Gas6
1.58E-29	0.8329334	0.535	0.41	3.84E-25	1	Neu1
2.68E-29	0.76981325	0.583	0.438	6.51E-25	1	Pid1
1.13E-28	0.48557911	0.836	0.764	2.74E-24	1	Zeb2
1.50E-28	0.34255149	0.929	0.878	3.64E-24	1	Fabp5
1.87E-28	0.35891756	0.966	0.924	4.52E-24	1	Pfn1
2.28E-28	0.56129553	0.74	0.668	5.52E-24	1	Btg1
2.56E-28	0.48032084	0.853	0.794	6.21E-24	1	Taldo1
1.10E-27	0.55310852	0.718	0.667	2.67E-23	1	Lamp2
2.16E-27	0.45058641	0.828	0.794	5.23E-23	1	Actr2
2.41E-27	0.59994504	0.716	0.677	5.85E-23	1	Ap2s1
2.55E-27	0.51975695	0.799	0.723	6.19E-23	1	Efhd2
4.30E-27	0.58996574	0.703	0.623	1.04E-22	1	Arl6ip1
8.52E-27	0.56035399	0.725	0.681	2.07E-22	1	Adipor1
2.09E-26	0.67435877	0.672	0.606	5.06E-22	1	Gltp
3.84E-26	0.78530259	0.311	0.167	9.32E-22	1	Ms4a6b
4.93E-26	0.86872767	0.585	0.518	1.20E-21	1	Ap1b1
1.27E-25	0.38057122	0.915	0.851	3.07E-21	1	Chchd2
1.71E-25	0.76766329	0.432	0.285	4.14E-21	1	Clec4a2
6.83E-25	0.81825167	0.576	0.492	1.66E-20	1	Dusp6

6.88E-25	0.75791023	0.387	0.24	1.67E-20	1	Entpd1
8.70E-25	0.71563707	0.623	0.577	2.11E-20	1	Mfsd1
6.72E-24	0.60286939	0.6	0.457	1.63E-19	1	Pla2g7
7.01E-24	0.44710875	0.791	0.744	1.70E-19	1	Cltc
1.39E-23	0.9011136	0.395	0.247	3.38E-19	1	F13a1
1.42E-23	0.59912268	0.685	0.61	3.43E-19	1	Zfp36l1
1.67E-23	0.95783562	0.506	0.43	4.04E-19	1	Fcgrt
1.83E-23	0.48953865	0.761	0.724	4.43E-19	1	Serp1
3.63E-23	0.58493013	0.596	0.453	8.80E-19	1	Cd84
4.23E-23	0.61164656	0.679	0.639	1.02E-18	1	Grk2
6.31E-23	0.70626203	0.476	0.347	1.53E-18	1	Vsir
1.69E-22	0.6761714	0.381	0.241	4.09E-18	1	Pld4
2.14E-22	0.51419046	0.708	0.659	5.20E-18	1	Ctsa
2.22E-22	0.53010974	0.701	0.672	5.38E-18	1	Ptbp3
2.31E-22	0.52163983	0.727	0.625	5.61E-18	1	Cotl1
4.25E-22	0.60180984	0.667	0.554	1.03E-17	1	Mafb
4.85E-22	0.61176158	0.62	0.554	1.18E-17	1	Wwp1
6.45E-22	0.60551516	0.499	0.361	1.56E-17	1	Fcgr3
8.37E-22	0.62890282	0.603	0.503	2.03E-17	1	Itgb2
2.52E-21	0.62391835	0.496	0.362	6.11E-17	1	Nrros
2.87E-21	0.64740197	0.482	0.354	6.96E-17	1	Rbm47
3.68E-21	0.75089874	0.48	0.367	8.93E-17	1	Fcgr2b

4.18E-21	0.66250234	0.436	0.301	1.01E-16	1	Ms4a6c
4.41E-21	0.65293859	0.644	0.613	1.07E-16	1	Fam49b
1.32E-20	0.62872256	0.597	0.524	3.19E-16	1	Rab8b
1.64E-20	0.60191267	0.661	0.634	3.97E-16	1	Fxyd5
1.82E-20	0.45593939	0.74	0.698	4.42E-16	1	Arf5
4.84E-20	0.58691147	0.627	0.601	1.17E-15	1	Cd164
6.40E-20	0.63480693	0.555	0.478	1.55E-15	1	Sh3bp5
9.21E-20	0.79027701	0.403	0.295	2.23E-15	1	Sesn1
9.44E-20	0.71397949	0.434	0.314	2.29E-15	1	Ccr1
1.09E-19	0.53503667	0.667	0.618	2.64E-15	1	Hexa
1.55E-19	0.46442909	0.725	0.689	3.76E-15	1	Mcl1
2.62E-19	0.53382979	0.62	0.488	6.35E-15	1	Pf4
2.88E-19	0.64347895	0.551	0.447	6.99E-15	1	Rnf128
3.74E-19	0.67636979	0.556	0.445	9.07E-15	1	Plek
7.21E-19	0.58610144	0.442	0.312	1.75E-14	1	Ptpn18
7.69E-19	0.46660236	0.754	0.72	1.87E-14	1	Ckb
8.52E-19	0.4942226	0.662	0.571	2.06E-14	1	Tpd52
1.58E-18	0.50956659	0.71	0.678	3.84E-14	1	Cyba
5.96E-18	0.53607348	0.644	0.607	1.45E-13	1	Itm2c
8.28E-18	0.72244582	0.565	0.542	2.01E-13	1	Ifngr1
1.11E-17	0.56583937	0.609	0.585	2.69E-13	1	Cd47
1.12E-17	0.68260916	0.353	0.23	2.71E-13	1	Abca1

1.41E-17	0.30445434	0.795	0.677	3.42E-13	1	Tyrobp
1.56E-17	0.64432799	0.37	0.254	3.77E-13	1	Prkcb
1.94E-17	0.72363511	0.516	0.449	4.71E-13	1	Kctd12
2.50E-17	0.69291095	0.431	0.315	6.05E-13	1	Itgal
3.97E-17	0.65786409	0.483	0.374	9.63E-13	1	Ccl6
1.37E-16	0.43696993	0.709	0.691	3.32E-12	1	Ddx39b
1.39E-16	0.55735011	0.466	0.36	3.37E-12	1	Inpp5d
2.14E-16	0.34395146	0.764	0.742	5.18E-12	1	Rsrp1
2.20E-16	0.37294284	0.751	0.727	5.33E-12	1	Srsf5
2.50E-16	0.64799098	0.404	0.297	6.07E-12	1	Fcgr4
3.18E-16	0.6468027	0.329	0.221	7.72E-12	1	Lcp2
3.33E-16	0.36458348	0.732	0.712	8.07E-12	1	Atp6v1a
3.46E-16	0.4736359	0.503	0.387	8.38E-12	1	Fyb
5.32E-16	0.42998132	0.709	0.708	1.29E-11	1	Snx3
6.51E-16	0.63275859	0.315	0.209	1.58E-11	1	Rassf4
7.86E-16	0.55239213	0.525	0.435	1.91E-11	1	Ptafr
1.09E-15	0.43385198	0.702	0.669	2.65E-11	1	Atp1a1
2.85E-15	0.60076903	0.339	0.236	6.90E-11	1	Il10ra
3.26E-15	0.35398514	0.696	0.594	7.90E-11	1	Fcer1g
3.52E-15	0.57231083	0.579	0.564	8.53E-11	1	Adam15
4.63E-15	0.53482562	0.468	0.374	1.12E-10	1	Tm6sf1
5.36E-15	0.50825194	0.613	0.605	1.30E-10	1	Rtn3

5.54E-15	0.31039777	0.855	0.814	1.34E-10	1	H3f3b
1.51E-14	0.36423219	0.785	0.772	3.67E-10	1	Hnrnpf
5.52E-14	0.5978778	0.565	0.563	1.34E-09	1	Sypl
1.05E-13	0.56040862	0.542	0.489	2.55E-09	1	Dab2
1.06E-13	0.48687553	0.532	0.431	2.57E-09	1	C3ar1
1.64E-13	0.35549028	0.754	0.74	3.97E-09	1	Atp6v1g1
1.95E-13	0.7093705	0.466	0.418	4.72E-09	1	Pdlim4
2.63E-13	0.3032613	0.809	0.741	6.37E-09	1	Lrp1
2.63E-13	0.58657427	0.412	0.32	6.38E-09	1	Ly9
2.72E-13	0.5174975	0.582	0.58	6.60E-09	1	Man2a1
3.09E-13	0.44163898	0.703	0.686	7.49E-09	1	Cebpb
5.71E-13	0.43633552	0.644	0.578	1.38E-08	1	Lipa
6.10E-13	0.52743912	0.49	0.424	1.48E-08	1	Celf2
6.32E-13	0.53692103	0.268	0.171	1.53E-08	1	Pou2f2
9.20E-13	0.55829229	0.555	0.531	2.23E-08	1	Idh1
1.35E-12	0.61511261	0.476	0.433	3.26E-08	1	Bst2
1.53E-12	0.43990826	0.63	0.611	3.72E-08	1	Klf6
2.37E-12	0.45916019	0.637	0.625	5.74E-08	1	Hmox1
3.64E-12	0.51961712	0.547	0.487	8.82E-08	1	Smpdl3a
4.05E-12	0.46838456	0.531	0.468	9.83E-08	1	Glul
4.52E-12	0.49049612	0.268	0.176	1.10E-07	1	Trim30a
6.25E-12	0.34282726	0.668	0.602	1.52E-07	1	Ucp2
7.36E-12	0.36310586	0.686	0.705	1.78E-07	1	Atp6v0e

7.60E-12	0.5529366<br>5	0.353	0.262	1.84E-07	1	Dock10
9.14E-12	0.6667541<br>7	0.314	0.232	2.22E-07	1	Tbxas1
1.00E-11	0.4882912<br>1	0.363	0.272	2.43E-07	1	Ncf1
1.12E-11	0.3687029	0.671	0.673	2.71E-07	1	Cst3
1.29E-11	0.5941715	0.379	0.304	3.13E-07	1	Fam105a
1.32E-11	0.2882968<br>3	0.733	0.747	3.21E-07	1	Capza2
1.36E-11	0.5731683<br>4	0.357	0.274	3.29E-07	1	mt-Tw
1.47E-11	0.6616123	0.504	0.501	3.57E-07	1	Scarb2
1.69E-11	0.6630551<br>7	0.465	0.406	4.09E-07	1	Rgs2
1.69E-11	0.5790079	0.564	0.562	4.11E-07	1	Rhog
1.96E-11	0.5449321	0.305	0.219	4.76E-07	1	Lrrc25
2.37E-11	0.4476505<br>9	0.603	0.618	5.75E-07	1	Bri3
2.39E-11	0.5106108<br>1	0.388	0.307	5.80E-07	1	Ms4a6d
2.58E-11	0.7186344<br>4	0.346	0.284	6.27E-07	1	Ptprj
3.23E-11	0.634769	0.442	0.394	7.83E-07	1	Pdxk
3.49E-11	0.4206586<br>9	0.638	0.648	8.46E-07	1	9-Sep
3.95E-11	0.7621276<br>5	0.434	0.4	9.58E-07	1	Ier3
4.34E-11	0.5994063<br>3	0.487	0.474	1.05E-06	1	Ier5
5.44E-11	0.4844746<br>7	0.588	0.593	1.32E-06	1	Tab2
5.62E-11	0.6020278<br>9	0.425	0.366	1.36E-06	1	Renbp
5.74E-11	0.2998791<br>1	0.734	0.727	1.39E-06	1	Pdcd6ip
6.01E-11	0.5698323<br>8	0.439	0.38	1.46E-06	1	Dnase2a
7.72E-11	0.5879643<br>3	0.318	0.238	1.87E-06	1	P2ry6
8.78E-11	0.3233900<br>1	0.78	0.714	2.13E-06	1	Canx

8.99E-11	0.47655303	0.499	0.433	2.18E-06	1	Cd300ld
1.32E-10	0.49311112	0.559	0.545	3.19E-06	1	Atp6v0a1
1.46E-10	0.60028703	0.511	0.521	3.53E-06	1	Susd6
2.70E-10	0.54939843	0.514	0.482	6.55E-06	1	Zfp36
3.34E-10	0.46299943	0.466	0.393	8.10E-06	1	Msr1
5.34E-10	0.49985258	0.496	0.457	1.29E-05	1	Tnfrsf1b
5.87E-10	0.56485846	0.379	0.308	1.42E-05	1	Itga6
6.80E-10	0.4247412	0.251	0.171	1.65E-05	1	Itga4
8.12E-10	0.47727776	0.579	0.604	1.97E-05	1	Atp6v1f
9.03E-10	0.5471056	0.362	0.297	2.19E-05	1	Rassf2
1.03E-09	0.41504603	0.63	0.637	2.51E-05	1	Atp6v0b
1.07E-09	0.55652898	0.537	0.548	2.58E-05	1	Gna12
1.23E-09	0.52335761	0.507	0.515	2.97E-05	1	Ptpra
1.56E-09	0.72086291	0.274	0.203	3.79E-05	1	Ccl3
1.69E-09	0.31604809	0.682	0.684	4.10E-05	1	Tnfaip2
2.45E-09	0.64673741	0.439	0.408	5.95E-05	1	Arap1
3.20E-09	0.50131661	0.487	0.465	7.76E-05	1	Ehd4
3.83E-09	0.44665177	0.285	0.21	9.29E-05	1	Cd200r1
4.25E-09	0.39513708	0.455	0.386	0.00010299	1	Coro1a
5.49E-09	0.50077795	0.41	0.356	0.00013323	1	Arrb2
6.64E-09	0.61354525	0.362	0.316	0.00016106	1	Orai1
6.73E-09	0.53571751	0.274	0.202	0.00016308	1	Srgn

7.06E-09	0.55492984	0.312	0.244	0.00017125	1	Slc11a1
7.54E-09	0.45989399	0.323	0.251	0.00018273	1	Cd300lb
7.62E-09	0.39494541	0.451	0.387	0.00018471	1	Lst1
8.41E-09	0.27641936	0.661	0.685	0.00020402	1	Sqstm1
1.26E-08	0.58127765	0.458	0.439	0.00030505	1	Plxnd1
1.41E-08	0.47849767	0.549	0.559	0.00034142	1	Gsr
1.46E-08	0.37955928	0.614	0.641	0.00035348	1	Ppp1r18
1.60E-08	0.40508715	0.367	0.296	0.00038761	1	Ptpn6
1.78E-08	0.25502708	0.718	0.738	0.00043132	1	Gdi2
1.79E-08	0.60812702	0.466	0.466	0.00043446	1	Rnf13
2.24E-08	0.44287209	0.623	0.623	0.00054324	1	Junb
2.42E-08	0.34706634	0.631	0.637	0.00058756	1	Zfp36l2
2.52E-08	0.72882915	0.338	0.289	0.00061014	1	Stat1
2.60E-08	0.53071129	0.583	0.602	0.00062985	1	Cat
3.30E-08	0.57487132	0.329	0.27	0.00079923	1	Arhgap18
3.41E-08	0.42003348	0.472	0.44	0.00082602	1	Gm2a
3.79E-08	0.48884045	0.523	0.544	0.00091879	1	Vps4b
4.07E-08	0.54066589	0.417	0.381	0.00098804	1	Arhgap19
4.38E-08	0.57131537	0.496	0.506	0.0010627	1	Vwa5a
4.55E-08	0.53242204	0.521	0.521	0.00110228	1	Abhd12
4.85E-08	0.28101553	0.638	0.667	0.00117483	1	Vamp8

4.94E-08	0.378903	0.596	0.616	0.00119721	1	Edem1
5.03E-08	0.46201201	0.393	0.331	0.00121886	1	Hcls1
5.08E-08	0.57843145	0.455	0.441	0.00123088	1	Arl4c
5.32E-08	0.54143365	0.349	0.3	0.00128991	1	Dock8
5.87E-08	0.43970965	0.379	0.314	0.00142357	1	Ncf2
6.12E-08	0.4313378	0.525	0.549	0.00148409	1	G3bp2
6.20E-08	0.48767587	0.298	0.233	0.00150285	1	Lpxn
6.37E-08	0.38320315	0.589	0.613	0.0015448	1	Degs1
6.66E-08	0.26119026	0.705	0.722	0.00161414	1	Srsf2
6.76E-08	0.37541055	0.527	0.474	0.00163819	1	Trem2
7.39E-08	0.37790604	0.299	0.23	0.0017918	1	AI662270
9.99E-08	0.68416797	0.292	0.244	0.00242132	1	Sgsh
1.06E-07	0.41996194	0.575	0.601	0.00257011	1	Emc10
1.19E-07	0.47929689	0.357	0.307	0.00287659	1	Ptk2b
1.34E-07	0.53874503	0.441	0.425	0.00325352	1	Npc1
1.62E-07	0.42312244	0.555	0.575	0.00393765	1	Snx2
1.68E-07	0.35003891	0.6	0.618	0.00408517	1	Soat1
2.03E-07	0.51382709	0.465	0.464	0.00492388	1	Fam174a
2.06E-07	0.5921034	0.483	0.485	0.00499339	1	Tfrc
2.35E-07	0.4429412	0.53	0.554	0.005692	1	M6pr
2.35E-07	0.54895411	0.462	0.459	0.00569669	1	Rnf130
3.11E-07	0.55690791	0.292	0.241	0.00755284	1	Cln3

3.46E-07	0.3967398	0.439	0.385	0.0083914<br>8	1	C5ar1
3.52E-07	0.6051642<br>4	0.336	0.296	0.0085308<br>9	1	Map3k1
3.98E-07	0.6824023<br>1	0.355	0.331	0.0096597<br>5	1	Notch1
4.49E-07	0.5483143<br>9	0.421	0.396	0.0108806<br>3	1	Ninj1
4.81E-07	0.3177221	0.596	0.647	0.0116572<br>4	1	H2afy
6.54E-07	0.4568068	0.285	0.224	0.0158592<br>4	1	Nckap1l
6.86E-07	0.3571193<br>8	0.41	0.346	0.0166427<br>5	1	Clec4d
7.62E-07	0.3968571<br>9	0.418	0.381	0.0184799<br>5	1	Lyn
8.07E-07	0.5888744<br>2	0.383	0.365	0.0195786<br>2	1	Man2b1
8.22E-07	0.2821845	0.633	0.67	0.0199332<br>3	1	Arpc3
9.25E-07	0.4052209<br>1	0.316	0.258	0.0224218<br>5	1	Lmo2
9.29E-07	0.4744923	0.459	0.456	0.0225309<br>4	1	Hpcal1
9.29E-07	0.2891965	0.716	0.726	0.0225340<br>8	1	Rap1b
9.58E-07	0.4711352<br>4	0.497	0.527	0.0232220<br>8	1	Lnpep
1.00E-06	0.5067198<br>2	0.256	0.201	0.0243666<br>2	1	Frmd4b
1.02E-06	0.3638587<br>3	0.597	0.642	0.0248075<br>4	1	Snx5
1.10E-06	0.6355030<br>5	0.4	0.4	0.0267262<br>8	1	Scamp2
1.11E-06	0.4757650<br>5	0.482	0.486	0.0268356<br>7	1	Myl12b
1.19E-06	0.5275446<br>5	0.428	0.421	0.0287879<br>1	1	Stk17b
1.26E-06	0.5270372<br>5	0.427	0.419	0.0305536<br>4	1	Cdc42se2
1.31E-06	0.4452383<br>4	0.525	0.538	0.0317906<br>5	1	Prkcd

1.48E-06	0.27247506	0.583	0.619	0.03588332	1	2900097C17Rik
1.69E-06	0.43516962	0.46	0.437	0.04089354	1	Nceh1
1.70E-06	0.42540954	0.287	0.231	0.04111847	1	Clec12a
1.87E-06	0.32632776	0.614	0.653	0.04542056	1	Ctsz
2.14E-06	0.48097423	0.483	0.504	0.05179319	1	Zfp703
2.17E-06	0.42079931	0.27	0.215	0.0525372	1	Arhgap30
2.19E-06	0.51372009	0.405	0.391	0.05320443	1	Pip4k2a
2.22E-06	0.48971699	0.473	0.482	0.05383534	1	Plekhb2
2.57E-06	0.25529102	0.165	0.288	0.06220966	1	Taok2
2.94E-06	0.40277119	0.319	0.269	0.07138189	1	Rab3il1
3.70E-06	0.51604143	0.362	0.337	0.08971263	1	Cmtm6
4.98E-06	0.71301641	0.347	0.338	0.1207927	1	Usf1
5.58E-06	0.31051279	0.627	0.637	0.13526718	1	Rhob
5.84E-06	0.30847542	0.603	0.652	0.14165195	1	Ddx17
6.59E-06	0.25426177	0.169	0.29	0.15970521	1	Setd1b
6.70E-06	0.41589389	0.487	0.526	0.16241447	1	Sdf4
6.71E-06	0.30177583	0.174	0.295	0.16275559	1	Srek1ip1
9.64E-06	0.45589251	0.456	0.447	0.23372373	1	Cxcl16
1.13E-05	0.38128116	0.52	0.542	0.27331619	1	Ubl3
1.26E-05	0.2892347	0.192	0.318	0.30630373	1	Nub1
1.30E-05	0.49738558	0.49	0.514	0.31558612	1	Nampt

1.33E-05	0.36920872	0.559	0.597	0.32277756	1	Mef2a
1.44E-05	0.4286256	0.49	0.514	0.34830901	1	Rassf3
1.56E-05	0.25710455	0.154	0.261	0.37910234	1	Insig2
1.76E-05	0.30042359	0.602	0.653	0.425577	1	Rab1a
1.77E-05	0.37225737	0.492	0.513	0.42838925	1	Xbp1
1.82E-05	0.50782112	0.456	0.488	0.44226884	1	Syngr2
1.87E-05	0.54317676	0.301	0.265	0.45354288	1	Plxnc1
1.98E-05	0.31367417	0.581	0.632	0.48009471	1	Cox7a2l
1.99E-05	0.29518944	0.164	0.274	0.48218194	1	Usp33
2.03E-05	0.65555462	0.34	0.33	0.49313121	1	P2rx4
2.07E-05	0.46804022	0.493	0.535	0.50295429	1	Samhd1
2.20E-05	0.4330104	0.271	0.227	0.5343692	1	Zdhhc14
2.26E-05	0.41683666	0.326	0.282	0.54895846	1	Ifi207
2.70E-05	0.39164706	0.294	0.252	0.65359905	1	Pirb
2.89E-05	0.31130456	0.562	0.612	0.70156601	1	Ppp3r1
3.33E-05	0.41019717	0.501	0.546	0.80807347	1	Atp6ap1
3.78E-05	0.47087611	0.261	0.218	0.91676942	1	Txnip
3.93E-05	0.40290182	0.297	0.256	0.95402357	1	Fgd4
4.78E-05	0.33125908	0.573	0.606	1	1	9530068E07Rik
4.89E-05	0.27192196	0.565	0.621	1	1	Pfdn5
5.94E-05	0.39516702	0.404	0.377	1	1	Rab32
6.05E-05	0.33479036	0.336	0.29	1	1	Irf8

6.31E-05	0.42051973	0.332	0.297	1	1	Syk
7.02E-05	0.34378263	0.52	0.569	1	1	Tmem30a
7.06E-05	0.39638629	0.479	0.475	1	1	Fos
7.12E-05	0.25180157	0.246	0.391	1	1	Eif4ebp2
7.18E-05	0.28326894	0.516	0.547	1	1	Tmed10
7.37E-05	0.26333276	0.185	0.299	1	1	Dyrk2
8.05E-05	0.33239786	0.589	0.618	1	1	Dusp1
8.74E-05	0.44161969	0.321	0.292	1	1	Pik3cd
9.70E-05	0.5598395	0.343	0.335	1	1	Etv3
0.00010292	0.37041567	0.285	0.239	1	1	Cd72
0.00010729	0.47375778	0.408	0.392	1	1	Tgm2
0.00013434	0.29050395	0.201	0.321	1	1	Tle3
0.00013469	0.293097	0.189	0.303	1	1	Zfx
0.0001386	0.25092585	0.169	0.27	1	1	Stxbp3
0.00013884	0.26408641	0.619	0.669	1	1	Snrpb
0.00014327	0.41045708	0.322	0.293	1	1	Cd48
0.00014933	0.31386236	0.544	0.593	1	1	Pea15a
0.00015013	0.26551505	0.172	0.276	1	1	Kdm6b
0.00017995	0.38923724	0.251	0.214	1	1	Lair1
0.00019919	0.36590211	0.353	0.322	1	1	Apobec1
0.00022942	0.31215061	0.205	0.321	1	1	Commd7
0.00028255	0.2615011	0.169	0.265	1	1	Trappc8

0.00030288	0.51172433	0.38	0.383	1	1	Kdm7a
0.00031994	0.27718615	0.198	0.307	1	1	Apaf1
0.00032628	0.26467958	0.232	0.359	1	1	Fhl3
0.0003638	0.43333689	0.446	0.479	1	1	Sgpl1
0.0003743	0.32258544	0.318	0.278	1	1	Cyth4
0.00038995	0.43262169	0.477	0.53	1	1	Usp25
0.000392	0.51931851	0.428	0.478	1	1	R3hdm4
0.00041349	0.28520795	0.177	0.276	1	1	Rps27rt
0.0004135	0.40129253	0.463	0.491	1	1	Gclm
0.00047349	0.51534866	0.318	0.309	1	1	Cln6
0.00049105	0.480599	0.397	0.401	1	1	Tmem106a
0.00063562	0.49674936	0.316	0.304	1	1	Gnpda1
0.00066785	0.2652205	0.169	0.264	1	1	Mid1ip1
0.00069442	0.34136126	0.5	0.563	1	1	Ndufa6
0.00070044	0.53256401	0.421	0.455	1	1	Tfe3
0.0007916	0.32621104	0.291	0.258	1	1	Slfn2
0.00089221	0.28004224	0.167	0.256	1	1	Zbtb7b
0.00090162	0.27417723	0.242	0.37	1	1	Emc1
0.00090185	0.39492072	0.424	0.432	1	1	Cerk
0.00090707	0.26762962	0.178	0.272	1	1	Zcchc11
0.00104954	0.51756854	0.268	0.252	1	1	Man1a

0.00107303	0.31498447	0.216	0.331	1	1	Ogfr
0.00111268	0.3126288	0.219	0.334	1	1	Gid8
0.00125845	0.52493658	0.414	0.46	1	1	Ube2q1
0.0013346	0.32080262	0.215	0.329	1	1	Zfp445
0.00142894	0.4311733	0.431	0.468	1	1	Arhgap17
0.00151799	0.2545044	0.191	0.288	1	1	Sumf1
0.0015647	0.39497265	0.411	0.427	1	1	Hebp1
0.00174822	0.28300966	0.208	0.312	1	1	Yipf4
0.00181543	0.25806018	0.186	0.28	1	1	Gatd1
0.00206725	0.29453853	0.194	0.293	1	1	Zdhhc9
0.00224487	0.25243678	0.206	0.304	1	1	Cyb5r4
0.00225823	0.31786111	0.521	0.591	1	1	Pgd
0.00232716	0.31245527	0.503	0.56	1	1	Dync1h1
0.00246402	0.25446718	0.258	0.386	1	1	Tbl1xr1
0.00255324	0.40063448	0.475	0.518	1	1	Plekho2
0.00265789	0.4140786	0.345	0.34	1	1	Irf5
0.0027199	0.37391759	0.453	0.495	1	1	Por
0.00286986	0.46464761	0.304	0.301	1	1	Rps13-ps1
0.00297487	0.26080116	0.237	0.352	1	1	Mllt10
0.00303982	0.28789179	0.514	0.55	1	1	Osbpl8
0.00322202	0.44134581	0.39	0.422	1	1	Ppp1r9b

0.0033887 6	0.2526419 3	0.25	0.218	1	1	Syngr1
0.0034414 5	0.3230214 7	0.476	0.526	1	1	Itgb5
0.0035240 8	0.4527084 5	0.363	0.376	1	1	Dapk1
0.0036355 6	0.2933050 1	0.229	0.341	1	1	Tmem9b
0.0037211 9	0.3119601 1	0.192	0.285	1	1	Vps50
0.0038927 2	0.3362523 6	0.49	0.551	1	1	Serinc1
0.0039416 7	0.3047559 9	0.171	0.253	1	1	Slc35a3
0.0040348 2	0.2844108 3	0.209	0.309	1	1	Slc25a37
0.0040870 8	0.4685543 2	0.421	0.473	1	1	Wdr89
0.0040980 8	0.2559941	0.551	0.587	1	1	Eif3f
0.0040985 7	0.2843224 7	0.199	0.292	1	1	Mindy2
0.0042111 4	0.3708746 3	0.421	0.452	1	1	Wipf1
0.0042689 7	0.3052816 4	0.201	0.298	1	1	Slc33a1
0.0043831 7	0.3133850 1	0.475	0.541	1	1	Scpep1
0.0045957 3	0.3047544 9	0.489	0.555	1	1	Pum2
0.0049801 8	0.3025651 7	0.209	0.307	1	1	Cstf2t
0.00517	0.2544023 6	0.562	0.638	1	1	Mapk3
0.0052251 4	0.3928996 1	0.346	0.354	1	1	Fnbp1
0.0052326 5	0.2779894 6	0.22	0.324	1	1	Derl2
0.0052937 9	0.4516255 3	0.404	0.454	1	1	Smarcc2
0.0053756 8	0.3803187 2	0.468	0.528	1	1	Myo5a

0.00561105	0.2538727	0.244	0.351	1	1	Trim25
0.00574514	0.26923128	0.251	0.368	1	1	N4bp1
0.00594395	0.27709095	0.484	0.55	1	1	Cers2
0.00612212	0.41080701	0.425	0.466	1	1	Ctsh
0.00644735	0.26721969	0.237	0.337	1	1	Hist1h2bc
0.00657868	0.37944815	0.326	0.323	1	1	Dck
0.00684637	0.29465716	0.294	0.414	1	1	Nfkbia
0.00704021	0.44960312	0.394	0.429	1	1	Rps2-ps10
0.00707375	0.27802425	0.253	0.371	1	1	Araf
0.00731709	0.29125071	0.52	0.603	1	1	Wdr26
0.00738436	0.29216958	0.198	0.284	1	1	Ccdc115
0.00753685	0.40518414	0.449	0.504	1	1	Rad21
0.00755735	0.25263986	0.246	0.355	1	1	Psmb10
0.00790013	0.27706266	0.239	0.346	1	1	Fbxl3
0.00795643	0.25595832	0.266	0.385	1	1	Ddi2
0.00803064	0.28588308	0.256	0.371	1	1	Blmh
0.00814837	0.42120661	0.432	0.486	1	1	Clk1
0.00825657	0.26323097	0.516	0.588	1	1	Ube2d2a
0.00859956	0.40419969	0.452	0.513	1	1	2610507B11Rik
0.00860731	0.42522881	0.448	0.531	1	1	Tm9sf2
0.0088836	0.25935872	0.281	0.41	1	1	Sdf2

0.00894102	0.28969373	0.218	0.313	1	1	Papd4
0	2.76934995	1	0.748	0	2	Col1a2
0	2.48160686	1	0.743	0	2	Col1a1
0	2.4407867	1	0.497	0	2	Sparc
0	2.27648854	1	0.402	0	2	Timp1
0	2.11120319	1	0.369	0	2	Serpinh1
0	2.09944519	1	0.407	0	2	Bgn
0	1.98637452	1	0.374	0	2	Lox
0	1.95588919	0.996	0.232	0	2	Cdc42ep3
0	1.9520727	0.997	0.291	0	2	Fstl1
0	1.9316675	0.993	0.259	0	2	Serpinf1
0	1.91712149	1	0.324	0	2	Ccnd2
0	1.87408877	0.869	0.114	0	2	Gas1
0	1.8279572	0.967	0.249	0	2	Inhba
0	1.82061784	0.855	0.145	0	2	Tnn
0	1.75072698	0.964	0.205	0	2	Loxl2
0	1.71063448	0.962	0.16	0	2	Cyp1b1
0	1.70725606	0.997	0.264	0	2	Aebp1
0	1.69982121	1	0.295	0	2	Col5a1
0	1.69747006	0.978	0.146	0	2	Dclk1
0	1.66310473	0.967	0.173	0	2	Cdh11
0	1.63703693	1	0.285	0	2	Col5a2
0	1.61311164	0.996	0.233	0	2	Prrx1

0	1.52842135	0.964	0.193	0	2	Serpine2
0	1.50413269	0.98	0.115	0	2	Cdh2
0	1.49180667	0.997	0.19	0	2	Fkbp10
0	1.46687794	0.81	0.105	0	2	Cemip
0	1.46321451	1	0.225	0	2	Ppic
0	1.45874872	0.991	0.161	0	2	Mrc2
0	1.44517731	0.923	0.118	0	2	Tgfb2
0	1.42579477	0.951	0.112	0	2	Ptprd
0	1.42161937	0.978	0.167	0	2	Plod2
0	1.38454608	0.906	0.134	0	2	Col8a1
0	1.37679294	0.975	0.156	0	2	Ccdc80
0	1.37249623	0.969	0.13	0	2	Snai2
0	1.36424655	0.974	0.18	0	2	Mmp2
0	1.36225165	0.958	0.142	0	2	Sp7
0	1.35814205	0.94	0.097	0	2	Col11a1
0	1.3518737	0.959	0.166	0	2	Fgf7
0	1.34383404	0.999	0.227	0	2	Dpysl3
0	1.3433075	0.892	0.175	0	2	Thbs2
0	1.34223919	0.99	0.199	0	2	Enah
0	1.311853	0.9	0.105	0	2	Ncam1
0	1.28722427	0.98	0.143	0	2	Sdc2
0	1.28015452	0.747	0.097	0	2	Alpl
0	1.27963474	0.958	0.121	0	2	Fgfr1

0	1.27522725	0.706	0.054	0	2	Dcn
0	1.27410595	0.985	0.184	0	2	Lhfp
0	1.26815181	0.898	0.107	0	2	Irx3
0	1.23697726	0.958	0.189	0	2	Pmepa1
0	1.20505036	0.951	0.107	0	2	Adamts2
0	1.19539224	0.971	0.178	0	2	Gpc1
0	1.19248096	0.987	0.166	0	2	Rcn1
0	1.18525803	0.999	0.191	0	2	Efemp2
0	1.18445891	0.983	0.163	0	2	Vasn
0	1.18272508	0.99	0.192	0	2	Nfix
0	1.18000969	0.987	0.195	0	2	Fkbp9
0	1.17841237	0.821	0.125	0	2	Pdk4
0	1.16570466	0.962	0.123	0	2	Olfml3
0	1.15811243	0.977	0.161	0	2	Fat1
0	1.15439928	0.985	0.233	0	2	Bmp1
0	1.15203945	0.964	0.163	0	2	Pcolce
0	1.14708425	0.922	0.102	0	2	Cdkn2a
0	1.13877831	0.929	0.138	0	2	Creb3l1
0	1.1182276	0.987	0.16	0	2	Ryk
0	1.11007792	0.962	0.134	0	2	Bicc1
0	1.11006569	0.946	0.155	0	2	Angptl2
0	1.10875299	0.977	0.175	0	2	Cpe

0	1.10801855	0.958	0.126	0	2	Mmp23
0	1.10351777	0.837	0.081	0	2	Cp
0	1.10332736	0.98	0.161	0	2	Chpf
0	1.1014004	0.927	0.153	0	2	Rgs3
0	1.08702651	0.953	0.14	0	2	Twist1
0	1.08497011	0.984	0.186	0	2	Ak1
0	1.08276641	0.959	0.158	0	2	Frmd6
0	1.06977146	0.852	0.094	0	2	Dkk3
0	1.06912	0.932	0.125	0	2	Efnb1
0	1.06089676	0.956	0.179	0	2	Amotl2
0	1.05912906	0.996	0.191	0	2	Rbfox2
0	1.04899829	0.869	0.117	0	2	Gja1
0	1.04322096	0.969	0.195	0	2	Sox4
0	1.03833882	0.959	0.151	0	2	Dcbld2
0	1.03458115	0.971	0.167	0	2	AI506816
0	1.02734484	0.956	0.184	0	2	Ext1
0	1.02596185	0.993	0.195	0	2	S100a16
0	1.02404628	0.968	0.185	0	2	Scd2
0	1.02114669	0.991	0.218	0	2	Maged1
0	1.01679494	0.839	0.104	0	2	Vcan
0	1.01264774	0.942	0.135	0	2	Nfib
0	1.00693474	0.961	0.177	0	2	Tmem176a

0	1.00409314	0.766	0.065	0	2	Adamts5
0	0.99241148	0.92	0.136	0	2	Phgdh
0	0.98685924	0.924	0.161	0	2	Vgll3
0	0.98002002	0.923	0.114	0	2	Mxra8
0	0.97630402	0.766	0.122	0	2	Hes1
0	0.97553297	0.969	0.216	0	2	Tsc22d1
0	0.9688072	0.922	0.113	0	2	Antxr1
0	0.96397452	0.938	0.126	0	2	Rnd3
0	0.9529184	0.916	0.125	0	2	Loxl1
0	0.95232341	0.92	0.138	0	2	Olfml2b
0	0.95041528	0.946	0.179	0	2	Pdgfa
0	0.94191757	0.961	0.127	0	2	Ptk7
0	0.93514918	0.858	0.087	0	2	Slit2
0	0.93294975	0.856	0.083	0	2	Lurap1l
0	0.93221777	0.923	0.139	0	2	Phlda3
0	0.92446144	0.919	0.117	0	2	Rcn3
0	0.92106246	0.965	0.193	0	2	Mical2
0	0.91204881	0.967	0.177	0	2	Tead1
0	0.89934357	0.826	0.088	0	2	Wisp2
0	0.89084324	0.968	0.181	0	2	Unc5b
0	0.88886525	0.818	0.131	0	2	Col6a2
0	0.88429808	0.77	0.092	0	2	Srpx2

0	0.8837857 3	0.968	0.165	0	2	Grb10
0	0.8799689 5	0.807	0.094	0	2	Nupr1
0	0.8749141	0.945	0.149	0	2	Cdc42bpa
0	0.8748490 9	0.952	0.125	0	2	Kirrel
0	0.8745232 6	0.932	0.174	0	2	Sdc1
0	0.8686912 5	0.924	0.113	0	2	P3h4
0	0.8657614 2	0.984	0.159	0	2	Ddr2
0	0.8639285 7	0.946	0.136	0	2	Amotl1
0	0.8594649 9	0.874	0.117	0	2	Ltbp1
0	0.8517931 5	0.977	0.196	0	2	Igf1r
0	0.8489763 8	0.891	0.108	0	2	Shox2
0	0.8437357 4	0.942	0.143	0	2	Wipi1
0	0.8369931 6	0.959	0.166	0	2	Utrn
0	0.8257332 1	0.953	0.185	0	2	Pbxip1
0	0.8209185 3	0.906	0.146	0	2	P4ha2
0	0.8171918 7	0.783	0.069	0	2	Wisp1
0	0.8114809 5	0.863	0.099	0	2	Snhg18
0	0.8091932 7	0.721	0.094	0	2	Mt2
0	0.7996010 4	0.919	0.127	0	2	Ephx1
0	0.7910399	0.962	0.179	0	2	Msrb3
0	0.7851509 3	0.89	0.121	0	2	Fbn1
0	0.7847509 5	0.856	0.087	0	2	Cryab

0	0.77828842	0.923	0.142	0	2	Pbx1
0	0.76923745	0.939	0.129	0	2	Tmem263
0	0.76840296	0.935	0.141	0	2	Pls3
0	0.76837672	0.906	0.101	0	2	Gpx8
0	0.76724575	0.906	0.109	0	2	Lpar1
0	0.76719733	0.714	0.051	0	2	Ptgis
0	0.76613097	0.897	0.112	0	2	Lamb1
0	0.76352404	0.824	0.129	0	2	Sema7a
0	0.76220947	0.942	0.153	0	2	Ehd2
0	0.75994925	0.946	0.167	0	2	Cttn
0	0.75659961	0.962	0.197	0	2	Ttc3
0	0.75349206	0.865	0.094	0	2	Prrx2
0	0.74794669	0.741	0.057	0	2	Inhbb
0	0.74710884	0.875	0.106	0	2	Dbn1
0	0.7467863	0.891	0.111	0	2	Runx2
0	0.74605604	0.939	0.138	0	2	Gstm2
0	0.74440094	0.853	0.09	0	2	2900026A02Rik
0	0.74286076	0.917	0.166	0	2	Crip2
0	0.74001126	0.94	0.167	0	2	Ece1
0	0.73879151	0.945	0.174	0	2	Smad7
0	0.73626201	0.795	0.117	0	2	Csrp2
0	0.73556464	0.922	0.104	0	2	Ptpn14

0	0.73467879	0.894	0.105	0	2	Kdelr3
0	0.73062672	0.792	0.082	0	2	Pcdh19
0	0.72759404	0.872	0.142	0	2	Psat1
0	0.72746844	0.872	0.121	0	2	Pkd2
0	0.7241237	0.856	0.115	0	2	Pdgfc
0	0.72186056	0.92	0.156	0	2	Tnfrsf12a
0	0.72066648	0.939	0.137	0	2	Snx7
0	0.71820204	0.804	0.088	0	2	Irs1
0	0.71628357	0.801	0.12	0	2	Pck2
0	0.71161273	0.964	0.175	0	2	Trove2
0	0.70665906	0.754	0.076	0	2	Ogn
0	0.70624552	0.682	0.093	0	2	Cthrc1
0	0.70029766	0.871	0.123	0	2	Bcat1
0	0.69019542	0.794	0.122	0	2	Kcnq1ot1
0	0.68947319	0.865	0.114	0	2	Epha2
0	0.68830525	0.782	0.074	0	2	Lgr6
0	0.68666096	0.926	0.141	0	2	Slc39a14
0	0.67421872	0.493	0.038	0	2	Wnt4
0	0.67106573	0.936	0.146	0	2	Me1
0	0.67065301	0.859	0.09	0	2	Npdc1
0	0.6693514	0.754	0.1	0	2	Lifr
0	0.66818001	0.914	0.145	0	2	Apbb2

0	0.66445397	0.94	0.15	0	2	Slc30a4
0	0.66441495	0.891	0.152	0	2	Socs5
0	0.66357053	0.833	0.133	0	2	Sgms2
0	0.66342929	0.711	0.071	0	2	Fam20a
0	0.66323794	0.827	0.108	0	2	Ebf1
0	0.66267513	0.891	0.154	0	2	Tbrg1
0	0.66181532	0.746	0.078	0	2	Pth1r
0	0.65967953	0.807	0.091	0	2	Rbms3
0	0.6589209	0.9	0.137	0	2	Tgfb1i1
0	0.65862192	0.765	0.113	0	2	Ddit4
0	0.65601538	0.862	0.14	0	2	Asns
0	0.64441565	0.871	0.132	0	2	Fhl2
0	0.63113803	0.849	0.12	0	2	Runx3
0	0.6308838	0.622	0.058	0	2	Gm38211
0	0.62843017	0.778	0.08	0	2	Itga11
0	0.62711253	0.926	0.15	0	2	Ikbip
0	0.62641925	0.712	0.078	0	2	Aldh1l2
0	0.62596264	0.792	0.103	0	2	Klf9
0	0.62591861	0.892	0.125	0	2	Copz2
0	0.62449356	0.64	0.06	0	2	Tubb3
0	0.6239542	0.9	0.138	0	2	Selenom
0	0.62153878	0.799	0.124	0	2	Klf4
0	0.61621149	0.879	0.124	0	2	Ttc28

0	0.610604	0.721	0.093	0	2	Snai1
0	0.6099269<br>5	0.82	0.09	0	2	Epdr1
0	0.6082146<br>3	0.692	0.073	0	2	Vdr
0	0.6028915<br>3	0.81	0.096	0	2	Wnt5a
0	0.6007334<br>8	0.85	0.082	0	2	Fkbp14
0	0.6002806<br>1	0.818	0.101	0	2	Gpr176
0	0.5998874<br>5	0.85	0.106	0	2	Rhobtb3
0	0.5977467<br>6	0.926	0.164	0	2	Samd4
0	0.5934848<br>9	0.762	0.077	0	2	3110039I08Rik
0	0.5927109<br>6	0.775	0.058	0	2	Cdkn2b
0	0.5906464<br>2	0.91	0.143	0	2	Nckap1
0	0.5900022<br>4	0.834	0.122	0	2	Fam198b
0	0.5888602<br>3	0.828	0.105	0	2	Epn2
0	0.5879928<br>6	0.894	0.116	0	2	Myo1b
0	0.5879179<br>3	0.901	0.167	0	2	Lats2
0	0.5840874<br>1	0.869	0.127	0	2	Scarf2
0	0.5832824<br>5	0.778	0.091	0	2	Zfhx4
0	0.5809734<br>6	0.692	0.094	0	2	Hbegf
0	0.5792601<br>3	0.811	0.106	0	2	Mast4
0	0.5761465<br>5	0.823	0.096	0	2	Hoxc10
0	0.5749017	0.847	0.098	0	2	Tanc1
0	0.5746075<br>5	0.908	0.164	0	2	Golim4
0	0.5742145	0.866	0.121	0	2	Col16a1

0	0.57113704	0.859	0.106	0	2	Nradd
0	0.56918519	0.942	0.156	0	2	Parva
0	0.56831865	0.856	0.11	0	2	Khdrbs3
0	0.56762196	0.805	0.095	0	2	Htra1
0	0.56684809	0.855	0.122	0	2	Ctdspl
0	0.56662947	0.863	0.124	0	2	Tpbg
0	0.56614866	0.863	0.126	0	2	Dlc1
0	0.56580194	0.901	0.144	0	2	Fam114a1
0	0.56513756	0.778	0.09	0	2	P3h3
0	0.56045271	0.895	0.145	0	2	Rras2
0	0.56009774	0.669	0.066	0	2	Irx5
0	0.55897435	0.786	0.098	0	2	Fads3
0	0.5564607	0.826	0.086	0	2	Fkbp7
0	0.55520337	0.863	0.141	0	2	Ssr2
0	0.55418741	0.782	0.102	0	2	Psph
0	0.55395555	0.868	0.15	0	2	Slc7a1
0	0.55251104	0.842	0.109	0	2	Emp2
0	0.55210399	0.82	0.122	0	2	Pdgfrb
0	0.5514941	0.802	0.087	0	2	Gpc4
0	0.54624527	0.84	0.129	0	2	Phldb2
0	0.54577542	0.759	0.092	0	2	Emilin1
0	0.5440067	0.686	0.059	0	2	Flnc
0	0.54159495	0.882	0.124	0	2	Rab34

0	0.53717635	0.908	0.162	0	2	Bzw2
0	0.53693584	0.773	0.072	0	2	Sytl2
0	0.53551562	0.882	0.152	0	2	Mtap
0	0.53476259	0.862	0.141	0	2	Tob1
0	0.53161769	0.847	0.106	0	2	Clstn1
0	0.53158722	0.519	0.023	0	2	Syn3
0	0.53126876	0.794	0.093	0	2	P4ha3
0	0.53004685	0.798	0.105	0	2	Lxn
0	0.52927491	0.794	0.08	0	2	Ptprg
0	0.52839214	0.866	0.133	0	2	Plp2
0	0.52542913	0.853	0.108	0	2	Cavin3
0	0.52531466	0.901	0.136	0	2	B3glct
0	0.52524243	0.703	0.083	0	2	Aqp1
0	0.52414424	0.856	0.145	0	2	Slc38a10
0	0.51957308	0.844	0.1	0	2	Arhgef25
0	0.51911076	0.868	0.118	0	2	Afap1
0	0.51903258	0.763	0.061	0	2	Cd276
0	0.51719275	0.766	0.087	0	2	Pcdh7
0	0.51637138	0.85	0.117	0	2	Serf1
0	0.51612908	0.73	0.082	0	2	Nsg1
0	0.51543264	0.762	0.073	0	2	Nbl1

0	0.5145747<br>7	0.769	0.078	0	2	Ptprf
0	0.5144521<br>7	0.821	0.087	0	2	Nxn
0	0.514279	0.869	0.139	0	2	Atp8b2
0	0.5137940<br>9	0.831	0.095	0	2	Scd1
0	0.5136091<br>3	0.756	0.076	0	2	Gli3
0	0.5122572<br>3	0.837	0.105	0	2	Kank2
0	0.5117630<br>3	0.885	0.14	0	2	Glipr2
0	0.5078736<br>4	0.823	0.09	0	2	Adgrl1
0	0.5064388<br>6	0.879	0.132	0	2	Pcbp4
0	0.5048764	0.721	0.068	0	2	Map1a
0	0.5043194<br>9	0.626	0.055	0	2	Islr
0	0.5029784<br>3	0.584	0.032	0	2	Clec11a
0	0.5029571<br>6	0.837	0.12	0	2	Chsy1
0	0.4990623<br>2	0.814	0.101	0	2	Prkca
0	0.4977532<br>8	0.738	0.074	0	2	Vldlr
0	0.4963308<br>7	0.773	0.082	0	2	Synpo
0	0.4916686<br>9	0.789	0.1	0	2	Pdcd4
0	0.4899447<br>6	0.612	0.043	0	2	Pdpn
0	0.4891774<br>1	0.73	0.077	0	2	Slc1a4
0	0.4884116	0.782	0.08	0	2	Pdia5
0	0.4844804<br>8	0.52	0.032	0	2	Kif26b
0	0.4841951<br>1	0.828	0.112	0	2	Tmem132a
0	0.4840238<br>2	0.811	0.086	0	2	Aldh18a1

0	0.48366495	0.747	0.093	0	2	Podnl1
0	0.48349236	0.89	0.154	0	2	Prnp
0	0.48245833	0.631	0.054	0	2	C130075A20Rik
0	0.48193361	0.757	0.073	0	2	Pard6g
0	0.47797	0.823	0.097	0	2	Mxra7
0	0.47697635	0.849	0.118	0	2	Yap1
0	0.4759942	0.762	0.09	0	2	Avpi1
0	0.47524065	0.798	0.092	0	2	Dab2ip
0	0.47507097	0.834	0.114	0	2	Tmem47
0	0.47493231	0.734	0.072	0	2	Gpc6
0	0.47265158	0.599	0.035	0	2	Lmo7
0	0.47122271	0.792	0.094	0	2	Sh3bp4
0	0.47107512	0.666	0.072	0	2	Fzd5
0	0.46979309	0.767	0.087	0	2	Bmpr1a
0	0.46947272	0.689	0.058	0	2	Satb2
0	0.46791023	0.846	0.14	0	2	Pdcd11
0	0.46767864	0.693	0.061	0	2	Fam171b
0	0.46760589	0.815	0.109	0	2	Hacd1
0	0.4630823	0.917	0.157	0	2	Gna11
0	0.46275422	0.826	0.115	0	2	Scn1b
0	0.46265133	0.561	0.042	0	2	Fap
0	0.46263576	0.67	0.073	0	2	Grb14
0	0.46126761	0.765	0.078	0	2	Cacna2d1

0	0.46000096	0.853	0.116	0	2	Ncs1
0	0.4596362	0.757	0.083	0	2	Steap2
0	0.45918665	0.82	0.114	0	2	Zcchc14
0	0.45895502	0.868	0.127	0	2	Ammecr1
0	0.45870795	0.674	0.073	0	2	Prkg2
0	0.45865875	0.817	0.119	0	2	Plxdc2
0	0.45800156	0.821	0.13	0	2	Iffo2
0	0.45467346	0.801	0.077	0	2	Ndrg4
0	0.45444172	0.722	0.087	0	2	Ddah2
0	0.454414	0.844	0.135	0	2	Cmtm4
0	0.45371992	0.882	0.154	0	2	Luzp1
0	0.45334539	0.718	0.068	0	2	Stox2
0	0.45282536	0.814	0.09	0	2	Cuedc1
0	0.45267031	0.767	0.103	0	2	Oaf
0	0.44960496	0.778	0.088	0	2	Tcaf1
0	0.44524908	0.77	0.108	0	2	Map1b
0	0.44396768	0.866	0.136	0	2	Kdm5b
0	0.44305773	0.781	0.091	0	2	Pard3
0	0.44264364	0.552	0.042	0	2	Fxyd1
0	0.44246494	0.64	0.062	0	2	Fbln5
0	0.44111898	0.882	0.148	0	2	Ctps
0	0.44035645	0.824	0.108	0	2	Lzts2

0	0.4403170 4	0.833	0.125	0	2	Prrc1
0	0.4396545 6	0.637	0.064	0	2	Dnm3os
0	0.4395370 2	0.804	0.129	0	2	Ctsk
0	0.4388352 6	0.628	0.07	0	2	Foxs1
0	0.4338049 9	0.693	0.074	0	2	Fkbp11
0	0.4325666 2	0.743	0.081	0	2	Fstl3
0	0.4316060 8	0.756	0.102	0	2	Ston2
0	0.4308576 2	0.74	0.083	0	2	Osmr
0	0.4304707 6	0.746	0.078	0	2	Glis2
0	0.4300036 7	0.605	0.057	0	2	Pcdh18
0	0.4281254 9	0.472	0.033	0	2	Sod3
0	0.4275986 2	0.839	0.118	0	2	Cpt1c
0	0.4260412 2	0.658	0.051	0	2	Mmp16
0	0.4239169 3	0.719	0.077	0	2	Myh10
0	0.4229187 3	0.91	0.157	0	2	Klhdc2
0	0.4221337 6	0.887	0.149	0	2	Lamc1
0	0.4217947 2	0.797	0.111	0	2	Gm28438
0	0.4214039 1	0.747	0.086	0	2	Dlg5
0	0.4186282 4	0.685	0.054	0	2	Pycr1
0	0.4181090 6	0.616	0.05	0	2	Tmem45a
0	0.4180349 8	0.767	0.087	0	2	Cdr2l

0	0.41755026	0.672	0.06	0	2	Myo1d
0	0.417337	0.587	0.039	0	2	Sphk1
0	0.41729186	0.68	0.074	0	2	Nfatc4
0	0.41643711	0.782	0.095	0	2	C1qtnf6
0	0.41586168	0.776	0.105	0	2	Smtn
0	0.41507247	0.635	0.059	0	2	Hoxc8
0	0.41444804	0.77	0.09	0	2	Arhgef17
0	0.41441504	0.705	0.072	0	2	Igsf3
0	0.41404833	0.878	0.154	0	2	Rnase4
0	0.41128873	0.775	0.085	0	2	Rarg
0	0.41119275	0.826	0.116	0	2	Bcar1
0	0.40828497	0.826	0.134	0	2	Ptgfrn
0	0.40823213	0.711	0.083	0	2	Gnai1
0	0.40813907	0.656	0.057	0	2	Sesn3
0	0.40772609	0.667	0.06	0	2	Scara3
0	0.40722744	0.712	0.086	0	2	Nexn
0	0.40516924	0.657	0.062	0	2	Pak3
0	0.40513003	0.75	0.083	0	2	Rgs12
0	0.40442025	0.75	0.084	0	2	P3h1
0	0.40377925	0.706	0.063	0	2	Shisa4
0	0.40202782	0.754	0.087	0	2	Neo1
0	0.40049629	0.66	0.054	0	2	Sspn

0	0.4004512	0.812	0.129	0	2	Sertad2
0	0.40004345	0.725	0.091	0	2	Rab23
0	0.39954637	0.673	0.074	0	2	Efnb2
0	0.39852651	0.708	0.078	0	2	Tspan6
0	0.39479946	0.626	0.06	0	2	Amot
0	0.39472395	0.815	0.115	0	2	Pabpn1
0	0.39414339	0.754	0.091	0	2	Eml1
0	0.39093739	0.763	0.092	0	2	Spats2
0	0.3855092	0.833	0.135	0	2	Erlec1
0	0.38474385	0.677	0.066	0	2	Msi1
0	0.38472845	0.734	0.093	0	2	Foxc1
0	0.38411117	0.767	0.09	0	2	H2afy2
0	0.38133344	0.645	0.056	0	2	Six1
0	0.380907	0.747	0.108	0	2	Nucb2
0	0.38055069	0.647	0.052	0	2	Itm2a
0	0.38023984	0.785	0.112	0	2	Tmem63b
0	0.37912679	0.535	0.034	0	2	Gxylt2
0	0.37662973	0.826	0.134	0	2	Cars
0	0.37609555	0.782	0.114	0	2	Otud7b
0	0.37500758	0.701	0.066	0	2	Gpx7
0	0.37195771	0.67	0.071	0	2	Steap1
0	0.37018847	0.676	0.074	0	2	Adgra2
0	0.36870159	0.568	0.053	0	2	Celf4

0	0.36795874	0.635	0.065	0	2	Gria3
0	0.36749721	0.538	0.045	0	2	Plat
0	0.36700336	0.747	0.097	0	2	Phldb1
0	0.36603073	0.791	0.116	0	2	Slc35a2
0	0.3637546	0.618	0.052	0	2	Fzd2
0	0.36144292	0.737	0.086	0	2	Cdc42ep1
0	0.36105772	0.751	0.103	0	2	Sesn2
0	0.36103175	0.826	0.118	0	2	Cep170b
0	0.36075255	0.815	0.121	0	2	Fam98a
0	0.36073807	0.812	0.12	0	2	Yeats2
0	0.36065355	0.795	0.118	0	2	Galnt10
0	0.36024664	0.605	0.039	0	2	Ptpn13
0	0.36013794	0.654	0.076	0	2	4632427E13Rik
0	0.36006915	0.775	0.115	0	2	Yars
0	0.35989703	0.757	0.097	0	2	Traf4
0	0.35922333	0.666	0.063	0	2	Casc4
0	0.35890102	0.831	0.135	0	2	Csnk1g1
0	0.35659415	0.628	0.047	0	2	Casp12
0	0.35563847	0.767	0.098	0	2	Syde1
0	0.35534852	0.606	0.064	0	2	Auts2
0	0.35247481	0.677	0.062	0	2	Pfn2
0	0.34941549	0.653	0.061	0	2	Bace1

0	0.3477389<br>3	0.663	0.057	0	2	Nnmt
0	0.3472135<br>8	0.689	0.08	0	2	Nectin3
0	0.3469156<br>6	0.731	0.092	0	2	Tmeff1
0	0.3466646<br>5	0.844	0.14	0	2	Oaz2
0	0.3446100<br>7	0.797	0.124	0	2	Rhoj
0	0.3445016<br>9	0.769	0.114	0	2	Itpr1
0	0.3430628<br>9	0.587	0.052	0	2	Zfp469
0	0.3426659<br>8	0.674	0.058	0	2	Plcd1
0	0.3422975<br>2	0.75	0.088	0	2	Arhgef40
0	0.3414984<br>9	0.743	0.102	0	2	Slc41a2
0	0.3412902<br>6	0.688	0.077	0	2	Tnks1bp1
0	0.3409941	0.75	0.093	0	2	Tjp1
0	0.3406826<br>8	0.672	0.073	0	2	Hoxa10
0	0.3395561<br>5	0.785	0.116	0	2	Ptp4a3
0	0.3376150<br>1	0.609	0.058	0	2	Arxes2
0	0.3373815<br>8	0.702	0.083	0	2	Cul7
0	0.3371222<br>8	0.699	0.082	0	2	Ube2e2
0	0.3371138<br>6	0.654	0.067	0	2	Il17rc
0	0.3369530<br>6	0.552	0.045	0	2	Setbp1
0	0.3359928<br>3	0.719	0.089	0	2	Adgra3
0	0.3350485<br>3	0.746	0.095	0	2	Gstm5
0	0.3332128<br>6	0.728	0.079	0	2	Sh3d19

0	0.3326546	0.494	0.04	0	2	Edil3
0	0.32920343	0.775	0.105	0	2	Hmgn3
0	0.32722232	0.658	0.063	0	2	Zfp462
0	0.32654535	0.669	0.081	0	2	Pcyox1l
0	0.32610711	0.661	0.064	0	2	Jam3
0	0.32540511	0.749	0.082	0	2	Osbpl5
0	0.32539962	0.76	0.098	0	2	Slco3a1
0	0.32533911	0.759	0.1	0	2	Parp3
0	0.32296873	0.685	0.084	0	2	Tead2
0	0.32200999	0.775	0.114	0	2	Atp11a
0	0.32167178	0.568	0.04	0	2	Jph2
0	0.32069526	0.773	0.117	0	2	Ano10
0	0.32056996	0.75	0.1	0	2	Zfp266
0	0.32050706	0.799	0.11	0	2	Megf8
0	0.31874651	0.669	0.051	0	2	Dysf
0	0.31620607	0.667	0.077	0	2	Cd109
0	0.31566238	0.65	0.075	0	2	Ndn
0	0.31492092	0.653	0.072	0	2	Lgalsl
0	0.31100924	0.677	0.08	0	2	Mpdz
0	0.31034576	0.625	0.05	0	2	Hebp2
0	0.30638356	0.65	0.069	0	2	Tbx15
0	0.30557651	0.734	0.093	0	2	Slc35e3

0	0.30464868	0.651	0.064	0	2	Cacnb3
0	0.30460071	0.766	0.109	0	2	Cbx6
0	0.30454401	0.733	0.097	0	2	Dnajc25
0	0.30419192	0.799	0.111	0	2	Snta1
0	0.30389277	0.587	0.063	0	2	Hoxc9
0	0.3038814	0.56	0.048	0	2	Slc6a9
0	0.30352856	0.666	0.072	0	2	Wfs1
0	0.29790856	0.66	0.076	0	2	Numbl
0	0.29738094	0.626	0.059	0	2	Praf2
0	0.29689427	0.781	0.106	0	2	Ptk2
0	0.29613231	0.599	0.055	0	2	Mcc
0	0.29456107	0.522	0.044	0	2	Zfpm2
0	0.29410434	0.779	0.112	0	2	Wwtr1
0	0.29405472	0.67	0.079	0	2	Cdr2
0	0.29334261	0.688	0.093	0	2	Pomgnt1
0	0.29282192	0.666	0.08	0	2	Btg3
0	0.29226151	0.775	0.117	0	2	Arhgap35
0	0.2922014	0.685	0.077	0	2	Armcx2
0	0.29153172	0.664	0.074	0	2	Smad3
0	0.29125521	0.574	0.055	0	2	Zfp9
0	0.29079278	0.808	0.128	0	2	Lrrc42
0	0.29047851	0.808	0.127	0	2	Fam92a

0	0.28750829	0.765	0.11	0	2	Mpzl1
0	0.2867062	0.576	0.037	0	2	Igdcc4
0	0.28431186	0.738	0.092	0	2	Trip6
0	0.28425705	0.746	0.106	0	2	Ptrh1
0	0.28384598	0.618	0.062	0	2	Adamtsl4
0	0.28138759	0.613	0.064	0	2	Kalrn
0	0.28114232	0.756	0.113	0	2	Ipo4
0	0.28067195	0.542	0.046	0	2	Ehd3
0	0.27817536	0.555	0.054	0	2	Adh7
0	0.27781003	0.625	0.066	0	2	Smo
0	0.27565085	0.812	0.123	0	2	Kctd17
0	0.27515679	0.568	0.059	0	2	Ubtd2
0	0.27460748	0.669	0.086	0	2	Chst11
0	0.27444396	0.688	0.08	0	2	Tnfrsf10b
0	0.27369729	0.513	0.045	0	2	Pik3r3
0	0.27369391	0.751	0.11	0	2	Camsap1
0	0.27294986	0.461	0.032	0	2	Slit3
0	0.27192348	0.642	0.069	0	2	Npr2
0	0.27147796	0.674	0.086	0	2	Pmm1
0	0.26785973	0.567	0.054	0	2	Tiam2
0	0.26692923	0.554	0.053	0	2	Srgap3
0	0.26606638	0.759	0.108	0	2	Chid1

0	0.26599243	0.621	0.07	0	2	Maged2
0	0.26577371	0.637	0.062	0	2	Fam149a
0	0.26317224	0.719	0.098	0	2	Yes1
0	0.26283716	0.503	0.039	0	2	Hhipl1
0	0.26205808	0.551	0.051	0	2	Ror2
0	0.26138461	0.676	0.078	0	2	Arl3
0	0.26088876	0.679	0.087	0	2	Uaca
0	0.26071827	0.629	0.07	0	2	Olfm1
0	0.2565897	0.625	0.073	0	2	Stk39
0	0.25422401	0.734	0.104	0	2	Yif1a
0	0.25182217	0.624	0.067	0	2	Mical3
0	0.25110938	0.533	0.04	0	2	Farp2
0	0.25108815	0.666	0.073	0	2	Src
5.41E-308	0.27702547	0.609	0.07	1.31E-303	2	Cxxc5
7.02E-308	0.29143506	0.584	0.065	1.70E-303	2	Syne1
8.68E-308	0.39802684	0.824	0.139	2.10E-303	2	Stim2
8.76E-308	0.32272844	0.763	0.116	2.12E-303	2	Glce
1.21E-307	0.33562856	0.783	0.122	2.94E-303	2	Grpel1
2.20E-307	0.57282986	0.523	0.048	5.34E-303	2	Fmod
2.87E-307	0.32139288	0.701	0.094	6.96E-303	2	Psrc1
3.12E-307	0.38680418	0.868	0.151	7.56E-303	2	Tusc3
4.58E-307	0.39938196	0.763	0.119	1.11E-302	2	Gm11478

4.74E-307	0.62062886	0.794	0.129	1.15E-302	2	Sorbs2
7.78E-307	0.30277477	0.842	0.14	1.89E-302	2	Ptcd3
1.51E-306	0.28244772	0.702	0.097	3.67E-302	2	Tacc2
1.67E-306	0.3632994	0.834	0.139	4.04E-302	2	Carhsp1
3.62E-306	1.78715027	1	0.368	8.79E-302	2	Csf1
5.26E-306	0.43493482	0.77	0.121	1.27E-301	2	Slc20a2
6.55E-306	0.3664751	0.859	0.149	1.59E-301	2	Mosmo
6.62E-306	0.42985062	0.878	0.152	1.60E-301	2	Aen
7.16E-306	0.3145184	0.76	0.117	1.74E-301	2	Me2
1.05E-305	0.34341567	0.802	0.127	2.55E-301	2	Camk2g
1.43E-305	0.38488589	0.84	0.144	3.47E-301	2	Gnpnat1
2.98E-305	0.62235309	0.929	0.183	7.22E-301	2	Fyn
4.15E-305	0.27326051	0.581	0.063	1.01E-300	2	Ccdc85b
9.72E-305	0.3673155	0.754	0.115	2.36E-300	2	Alkbh1
1.14E-304	0.36641285	0.846	0.143	2.77E-300	2	Slc35b1
1.29E-304	0.30610038	0.663	0.086	3.13E-300	2	Bag2
1.53E-304	0.28329416	0.769	0.12	3.71E-300	2	Kdelc2
1.80E-304	0.99301915	0.991	0.229	4.35E-300	2	Cnn3
2.01E-304	0.90499195	0.98	0.207	4.88E-300	2	Myo10
2.57E-304	0.27059034	0.814	0.134	6.22E-300	2	Pkd1
2.64E-304	0.31153029	0.67	0.087	6.40E-300	2	Bok
5.62E-304	0.62001434	0.943	0.181	1.36E-299	2	Cfl2
7.82E-304	0.35433659	0.781	0.125	1.90E-299	2	Sap18

7.88E-304	0.30868712	0.782	0.124	1.91E-299	2	Fam8a1
1.46E-303	0.25732023	0.75	0.111	3.53E-299	2	Pus7
3.99E-303	0.42856542	0.842	0.143	9.68E-299	2	Gys1
8.31E-303	1.0456414	0.983	0.214	2.01E-298	2	Fscn1
2.82E-302	0.32358257	0.859	0.149	6.83E-298	2	Anapc4
4.04E-302	0.30263285	0.847	0.144	9.78E-298	2	Creb3l2
1.02E-301	0.98391348	0.583	0.067	2.47E-297	2	Has2
1.06E-301	0.65914973	0.907	0.167	2.58E-297	2	Ktn1
2.70E-301	0.52181562	0.885	0.157	6.55E-297	2	Bcl9l
5.63E-301	0.25105223	0.635	0.079	1.36E-296	2	Lclat1
6.54E-301	0.26731551	0.75	0.114	1.59E-296	2	Frs2
1.98E-300	0.25914451	0.548	0.057	4.81E-296	2	Pacsin3
1.16E-299	0.32724519	0.731	0.107	2.82E-295	2	Mthfd1l
1.54E-299	0.30926785	0.795	0.13	3.74E-295	2	Ppan
4.61E-299	0.63371034	0.923	0.197	1.12E-294	2	mt-Atp8
5.60E-299	0.25956486	0.703	0.101	1.36E-294	2	Ublcp1
1.17E-298	0.33073456	0.855	0.151	2.84E-294	2	Lix1l
1.49E-298	0.30418772	0.669	0.089	3.62E-294	2	Ahnak2
2.02E-298	0.26301771	0.749	0.114	4.89E-294	2	Fasn
2.05E-298	0.46671482	0.892	0.164	4.97E-294	2	Nomo1
2.79E-298	0.29383636	0.548	0.057	6.78E-294	2	Clmp
4.75E-298	0.46493131	0.61	0.075	1.15E-293	2	Mustn1

7.13E-298	0.46517815	0.584	0.066	1.73E-293	2	Hs6st2
8.99E-298	0.31402446	0.615	0.075	2.18E-293	2	Dhcr24
1.53E-297	0.37401334	0.863	0.152	3.71E-293	2	Adpgk
1.70E-297	0.29591414	0.39	0.019	4.11E-293	2	Tspan11
2.68E-297	0.2986011	0.791	0.128	6.49E-293	2	Fnbp1l
3.20E-297	0.43318839	0.673	0.091	7.76E-293	2	Tgfb3
8.45E-297	0.44552079	0.881	0.162	2.05E-292	2	Kars
1.23E-295	0.26992554	0.603	0.074	2.98E-291	2	Sh3rf1
1.91E-295	0.5641334	0.92	0.182	4.62E-291	2	Tmed3
1.92E-295	0.56979283	0.932	0.188	4.65E-291	2	Fto
3.56E-295	0.78736112	0.876	0.164	8.62E-291	2	Myl9
3.83E-295	1.25674574	0.794	0.148	9.29E-291	2	Fbln2
5.21E-295	0.44185681	0.802	0.135	1.26E-290	2	Tes
5.45E-295	1.309918	0.981	0.254	1.32E-290	2	Vcl
2.87E-294	0.71075479	0.936	0.195	6.95E-290	2	Tmem176b
6.61E-294	1.68214289	0.99	0.325	1.60E-289	2	Ecm1
7.65E-294	0.47238103	0.542	0.056	1.85E-289	2	Medag
8.53E-294	0.79821372	0.719	0.112	2.07E-289	2	Ltbp2
1.21E-293	1.00810136	0.983	0.227	2.94E-289	2	2310022B05Rik
1.27E-293	0.29176968	0.538	0.055	3.07E-289	2	Mex3a
2.38E-293	0.37287972	0.82	0.137	5.78E-289	2	Igf2bp2
8.05E-293	0.67217496	0.968	0.205	1.95E-288	2	Ltbp3
8.63E-293	0.98617926	0.972	0.218	2.09E-288	2	Tm4sf1

2.84E-292	0.76158206	0.948	0.206	6.90E-288	2	Lars
3.21E-292	0.27311489	0.766	0.122	7.79E-288	2	Mgmt
3.76E-292	0.26688356	0.531	0.055	9.12E-288	2	Hoxc6
4.15E-292	0.25037705	0.703	0.102	1.01E-287	2	Ube2o
4.86E-292	0.61002961	0.933	0.188	1.18E-287	2	Dpy19l1
6.58E-292	0.81573842	0.977	0.229	1.60E-287	2	Ptprs
4.22E-291	0.2557629	0.467	0.039	1.02E-286	2	Gm42899
5.76E-291	0.31484987	0.558	0.06	1.40E-286	2	Tmem119
1.13E-290	0.33938778	0.631	0.082	2.73E-286	2	Ptprk
1.69E-290	0.41527143	0.882	0.162	4.11E-286	2	Dlg1
2.97E-290	0.30714792	0.84	0.147	7.20E-286	2	Gtf3c1
3.60E-290	0.47838323	0.906	0.178	8.72E-286	2	Sec23a
1.31E-289	0.26272515	0.641	0.083	3.16E-285	2	Cav1
2.46E-289	0.59588869	0.657	0.09	5.96E-285	2	Adamts1
3.05E-289	0.33564652	0.637	0.083	7.38E-285	2	Socs2
5.07E-289	1.05524276	0.904	0.188	1.23E-284	2	Prss23
7.94E-289	0.57735298	0.887	0.171	1.92E-284	2	Med13l
1.60E-288	1.26971634	0.981	0.256	3.88E-284	2	Tuba1a
2.81E-288	0.26318262	0.779	0.129	6.81E-284	2	Creb3
3.25E-288	0.72900213	0.868	0.167	7.89E-284	2	Nes
3.92E-288	0.42569901	0.794	0.129	9.51E-284	2	Ripk2
8.44E-288	0.28956389	0.834	0.146	2.05E-283	2	Mnat1

8.63E-288	0.3826344<br>7	0.483	0.042	2.09E-283	2	Enpp2
1.68E-287	0.5399506<br>2	0.807	0.14	4.08E-283	2	Tmem2
1.71E-287	0.5491606<br>3	0.935	0.19	4.14E-283	2	Gfpt1
3.11E-287	0.2608751<br>7	0.615	0.078	7.54E-283	2	Nfil3
3.68E-287	0.3549101<br>6	0.526	0.054	8.91E-283	2	Runx1t1
6.39E-287	1.3826686<br>8	1	0.295	1.55E-282	2	Qsox1
1.24E-286	0.2588943<br>1	0.612	0.077	3.00E-282	2	D16Ertd472e
1.52E-286	0.3896729	0.885	0.17	3.69E-282	2	Pros1
4.37E-286	0.7257616<br>5	0.968	0.208	1.06E-281	2	Dnajc10
7.63E-286	0.3125647<br>4	0.831	0.147	1.85E-281	2	Polr1a
1.08E-285	0.2692746<br>2	0.811	0.139	2.61E-281	2	Btbd7
2.75E-285	0.9106501<br>9	0.951	0.228	6.68E-281	2	Atf5
2.86E-285	1.1115003<br>7	0.932	0.206	6.95E-281	2	Col6a1
4.27E-285	0.3042141<br>5	0.497	0.046	1.04E-280	2	Ldb3
5.27E-285	0.3473965<br>7	0.824	0.145	1.28E-280	2	Exoc4
6.56E-285	0.4149361<br>8	0.923	0.183	1.59E-280	2	Slc39a13
1.36E-284	0.4065293<br>1	0.892	0.169	3.30E-280	2	Inf2
1.85E-284	0.2779835<br>2	0.733	0.112	4.50E-280	2	Lmf2
3.37E-284	0.4690975<br>2	0.862	0.159	8.17E-280	2	Polr2l
3.50E-284	0.5471775<br>8	0.778	0.128	8.49E-280	2	Phlda1
1.33E-283	0.2515363	0.81	0.139	3.21E-279	2	Gbf1
2.34E-283	0.2771748<br>2	0.637	0.084	5.68E-279	2	Fosl1

2.55E-283	0.27655231	0.834	0.144	6.18E-279	2	Ilf3
9.60E-283	0.3089135	0.674	0.098	2.33E-278	2	Sqle
1.01E-282	0.28898829	0.515	0.051	2.44E-278	2	Atp2b4
1.58E-282	1.22953844	0.945	0.23	3.83E-278	2	Errfi1
1.67E-282	0.27618686	0.853	0.156	4.05E-278	2	Ccser2
2.90E-282	0.51277359	0.917	0.181	7.04E-278	2	Colec12
3.17E-282	0.28244769	0.844	0.154	7.70E-278	2	Poldip2
3.46E-282	0.29110289	0.484	0.042	8.39E-278	2	Rgs17
3.14E-281	0.25090049	0.728	0.114	7.61E-277	2	Tsr1
3.54E-281	0.31828928	0.738	0.119	8.59E-277	2	Rrp8
4.31E-281	0.35591556	0.86	0.16	1.04E-276	2	Smpd1
6.74E-281	0.27526532	0.775	0.126	1.63E-276	2	Dkc1
1.06E-280	1.93198336	1	0.585	2.58E-276	2	Fn1
1.88E-280	0.77059668	0.949	0.215	4.55E-276	2	Gars
2.50E-280	0.2870829	0.52	0.054	6.07E-276	2	Eps8
4.76E-280	0.32758316	0.818	0.145	1.15E-275	2	Faf2
6.95E-280	0.26553069	0.436	0.032	1.68E-275	2	Nr4a2
1.23E-279	0.7707824	0.959	0.22	2.98E-275	2	Zbtb20
1.02E-278	0.2742144	0.754	0.124	2.47E-274	2	Znhit6
1.07E-278	1.43748813	1	0.329	2.59E-274	2	Hspg2
2.02E-278	0.33362008	0.485	0.045	4.90E-274	2	Chac1
2.73E-278	0.76514192	0.911	0.193	6.61E-274	2	S1pr1
2.76E-278	0.70164345	0.965	0.217	6.68E-274	2	Lman1

3.90E-278	0.27473346	0.849	0.157	9.46E-274	2	Helz
4.93E-278	0.61495456	0.932	0.201	1.19E-273	2	Tceal8
9.89E-278	0.46974187	0.952	0.197	2.40E-273	2	Pabpc4
1.21E-277	2.85202715	0.997	0.53	2.94E-273	2	Timp3
6.39E-277	0.60835184	0.914	0.186	1.55E-272	2	Srm
6.81E-277	0.28261165	0.766	0.127	1.65E-272	2	Adrm1
1.15E-276	0.95655325	0.956	0.242	2.80E-272	2	Ppib
1.21E-276	0.27594198	0.824	0.146	2.93E-272	2	Mak16
1.33E-276	0.26153583	0.817	0.143	3.21E-272	2	Pgrmc2
1.44E-276	1.72677777	0.984	0.317	3.48E-272	2	S100a4
7.97E-276	0.29629673	0.779	0.132	1.93E-271	2	Vars
1.02E-275	0.50882796	0.89	0.175	2.46E-271	2	Ppa1
1.05E-275	0.25198188	0.657	0.093	2.56E-271	2	Slc16a1
1.18E-275	0.41315414	0.429	0.031	2.87E-271	2	Slc13a5
9.43E-275	0.25768199	0.746	0.121	2.29E-270	2	Tmtc3
1.62E-274	0.29941133	0.891	0.172	3.92E-270	2	Smyd2
1.21E-273	0.26750967	0.539	0.061	2.95E-269	2	AC131339.2
1.26E-273	0.38537784	0.882	0.172	3.05E-269	2	Dnajc1
1.51E-273	0.3689018	0.592	0.074	3.67E-269	2	Gm2115
2.90E-273	0.25162452	0.849	0.159	7.03E-269	2	Rras
3.12E-273	0.9370823	0.991	0.26	7.58E-269	2	Selenow
3.93E-273	0.3070567	0.872	0.166	9.54E-269	2	Cdc16
6.82E-273	0.2525422	0.744	0.122	1.65E-268	2	Nectin2

7.06E-273	0.6147329<br>5	0.951	0.214	1.71E-268	2	Mxd4
1.06E-272	0.2911853<br>7	0.817	0.143	2.57E-268	2	Ddah1
1.07E-272	0.3832855<br>7	0.868	0.165	2.59E-268	2	Cd151
1.17E-272	0.3767883<br>2	0.792	0.135	2.84E-268	2	Gnb4
4.38E-272	0.3904635<br>4	0.603	0.079	1.06E-267	2	Masp1
6.64E-272	0.3388879	0.808	0.145	1.61E-267	2	Nfkbiz
1.70E-271	0.3279945<br>2	0.699	0.11	4.12E-267	2	Nedd9
4.14E-271	0.3780849<br>1	0.904	0.18	1.00E-266	2	Golt1b
6.05E-271	0.3832826<br>9	0.903	0.176	1.47E-266	2	Zmat3
6.23E-271	0.7722566<br>9	0.98	0.237	1.51E-266	2	Dst
1.50E-270	0.3750541<br>6	0.884	0.169	3.65E-266	2	Twsg1
1.59E-270	0.2676206<br>3	0.894	0.173	3.85E-266	2	Dmwd
2.39E-270	0.3210111<br>2	0.849	0.158	5.81E-266	2	Zrsr2
5.26E-270	0.2840838<br>4	0.368	0.02	1.28E-265	2	Osr1
4.33E-269	0.3384051	0.538	0.061	1.05E-264	2	Cspg4
7.28E-269	0.4862153<br>6	0.882	0.173	1.76E-264	2	Shmt2
4.14E-268	0.7775540<br>6	0.906	0.188	1.00E-263	2	Eno1
5.21E-268	0.3623418<br>5	0.69	0.108	1.26E-263	2	Slc7a5
7.54E-268	0.2662518<br>3	0.821	0.151	1.83E-263	2	Nol12
1.43E-267	0.2825728<br>3	0.756	0.129	3.47E-263	2	Akap9
2.09E-267	0.2700918<br>4	0.814	0.15	5.07E-263	2	Wars
2.35E-267	0.4932086<br>2	0.904	0.189	5.70E-263	2	Nfat5

5.22E-267	0.62087082	0.942	0.199	1.27E-262	2	Fam162a
7.43E-267	0.30660325	0.858	0.162	1.80E-262	2	Cyp20a1
8.85E-267	0.54896878	0.903	0.192	2.15E-262	2	Rai14
1.34E-266	0.27140822	0.812	0.148	3.25E-262	2	Prpsap1
1.43E-265	1.09060436	0.988	0.29	3.46E-261	2	Cd81
2.55E-265	0.61417671	0.946	0.207	6.18E-261	2	Il6st
4.97E-265	0.80599868	0.994	0.25	1.20E-260	2	Ppfibp1
1.31E-264	0.40010863	0.895	0.176	3.18E-260	2	Wwc2
3.00E-264	0.25818722	0.849	0.163	7.27E-260	2	Acadsb
3.98E-264	0.28371849	0.82	0.153	9.65E-260	2	Tmem70
5.01E-264	0.51133639	0.904	0.192	1.21E-259	2	Golgb1
2.49E-263	0.38215522	0.9	0.184	6.03E-259	2	Sec24d
2.97E-263	0.25437736	0.808	0.146	7.20E-259	2	Sigmar1
3.37E-263	1.30765538	1	0.341	8.18E-259	2	Nedd4
4.85E-263	0.28787372	0.555	0.069	1.18E-258	2	Chst15
2.35E-262	0.25695074	0.442	0.039	5.70E-258	2	Gm26523
4.39E-262	0.61105106	0.93	0.198	1.06E-257	2	Fermt2
5.33E-262	0.34756367	0.826	0.154	1.29E-257	2	Ddx18
5.35E-262	0.26287339	0.571	0.074	1.30E-257	2	Rps4l
7.10E-262	0.92402345	0.991	0.259	1.72E-257	2	Pam
1.02E-261	0.3227707	0.872	0.17	2.47E-257	2	Trp53
4.70E-261	0.32206687	0.814	0.153	1.14E-256	2	Smim3

7.34E-261	0.41119089	0.9	0.189	1.78E-256	2	Setd7
2.44E-260	0.3594571	0.84	0.162	5.91E-256	2	Snhg6
3.45E-260	0.31729535	0.897	0.182	8.36E-256	2	Ssbp3
9.57E-260	0.59554104	0.948	0.209	2.32E-255	2	Ankrd17
1.48E-258	0.29826228	0.807	0.147	3.59E-254	2	Polk
3.01E-258	0.55784911	0.945	0.213	7.30E-254	2	Tars
3.56E-258	0.25844338	0.812	0.153	8.64E-254	2	Vezf1
5.13E-258	0.29320638	0.625	0.092	1.24E-253	2	Ak4
6.73E-258	0.31086435	0.885	0.176	1.63E-253	2	Pcgf3
1.54E-257	0.29413936	0.743	0.131	3.74E-253	2	Mdn1
1.92E-257	0.29774272	0.776	0.143	4.65E-253	2	Phf10
2.74E-257	0.28815659	0.887	0.178	6.63E-253	2	Pitpnb
2.77E-257	0.25266166	0.844	0.162	6.72E-253	2	Mrps16
2.86E-257	1.09184355	0.959	0.231	6.94E-253	2	Col12a1
3.32E-257	1.0919246	0.983	0.291	8.05E-253	2	Plec
4.59E-257	0.34805194	0.858	0.169	1.11E-252	2	Cldnd1
2.71E-256	1.26331041	0.999	0.426	6.57E-252	2	Calu
3.34E-255	0.54285398	0.93	0.208	8.11E-251	2	Eif4e2
1.60E-254	0.70042961	0.855	0.185	3.88E-250	2	Uba52
1.91E-254	1.2782824	0.942	0.247	4.62E-250	2	Tnc
7.91E-254	0.3747898	0.868	0.172	1.92E-249	2	Slc39a6
1.29E-253	0.30332979	0.85	0.171	3.12E-249	2	Mbtps1
1.33E-253	0.2649647	0.557	0.073	3.22E-249	2	Tgfbr3

2.57E-253	0.45613909	0.908	0.19	6.23E-249	2	Psmd7
4.44E-253	0.26359072	0.82	0.159	1.08E-248	2	Rnf10
5.17E-253	0.27337105	0.355	0.021	1.25E-248	2	Lepr
1.09E-252	0.46164655	0.904	0.188	2.64E-248	2	Tsc22d2
1.29E-252	0.33146961	0.552	0.071	3.12E-248	2	Fst
3.11E-252	0.31930665	0.747	0.133	7.53E-248	2	Ier5l
4.20E-252	0.9659228	0.936	0.234	1.02E-247	2	Cxcl12
1.14E-251	0.4241626	0.943	0.208	2.77E-247	2	Ext2
1.34E-251	0.54111633	0.949	0.208	3.24E-247	2	Mprip
1.85E-251	0.54662179	0.901	0.192	4.48E-247	2	Klf7
2.69E-251	0.26402614	0.533	0.067	6.51E-247	2	Fzd1
2.96E-251	0.29205447	0.731	0.126	7.18E-247	2	Sacs
8.81E-251	0.77679908	0.969	0.252	2.14E-246	2	Eprs
1.26E-250	0.45975823	0.917	0.204	3.05E-246	2	Map1lc3a
1.33E-250	0.6268991	0.968	0.221	3.23E-246	2	Impad1
1.36E-250	0.62206899	0.955	0.224	3.31E-246	2	Fosl2
1.88E-250	0.26871181	0.82	0.162	4.57E-246	2	Gtf2i
7.87E-250	0.33624264	0.868	0.173	1.91E-245	2	Zdhhc5
1.02E-249	0.26368456	0.776	0.143	2.48E-245	2	Cpox
1.28E-249	0.3390602	0.888	0.182	3.11E-245	2	Arl6ip5
1.55E-249	0.88802084	0.983	0.26	3.76E-245	2	Kdelr2
1.68E-249	0.2895416	0.519	0.063	4.09E-245	2	Trib2
5.46E-249	0.35183497	0.92	0.201	1.32E-244	2	Cfap36

6.10E-249	0.2631542<br>2	0.824	0.161	1.48E-244	2	Rasa2
1.49E-248	0.2958750<br>5	0.865	0.174	3.61E-244	2	Gps1
2.07E-248	0.2976225<br>9	0.836	0.161	5.03E-244	2	Dnajb4
1.21E-247	0.4143536<br>3	0.876	0.179	2.92E-243	2	Pkn2
1.95E-247	0.3185461<br>8	0.334	0.017	4.72E-243	2	Ndnf
2.15E-247	0.9755665<br>8	0.981	0.271	5.22E-243	2	Pdia4
2.81E-247	0.3455616<br>1	0.885	0.188	6.80E-243	2	Ergic3
2.82E-247	0.2575233<br>5	0.74	0.134	6.85E-243	2	Kremen1
3.41E-247	0.2567771<br>7	0.823	0.16	8.28E-243	2	Mgat1
1.28E-246	0.2943698<br>1	0.916	0.195	3.10E-242	2	Cdk16
2.43E-246	0.3879624	0.801	0.153	5.90E-242	2	Epb41l3
5.10E-246	0.2758666<br>4	0.818	0.161	1.24E-241	2	Golga3
5.81E-246	0.4201571<br>8	0.895	0.189	1.41E-241	2	Plod3
7.36E-246	0.2746158	0.778	0.146	1.78E-241	2	Bri3bp
1.05E-245	0.5262681<br>8	0.564	0.078	2.55E-241	2	Smoc2
1.36E-245	0.5473331<br>4	0.818	0.167	3.30E-241	2	Cfh
1.51E-245	0.2997350<br>4	0.827	0.162	3.67E-241	2	Nfia
3.43E-245	0.7797980<br>2	0.943	0.233	8.31E-241	2	Lima1
7.01E-245	1.4871737<br>9	0.999	0.526	1.70E-240	2	Timp2
1.80E-244	1.3782600<br>2	1	0.421	4.37E-240	2	Ckap4
2.74E-244	0.3688098<br>9	0.808	0.16	6.65E-240	2	2810474O19Ri<br>k
4.88E-244	0.2884792<br>8	0.844	0.169	1.18E-239	2	Cebpz

2.02E-243	0.27166097	0.904	0.191	4.91E-239	2	Dap3
2.58E-243	0.26450557	0.856	0.173	6.26E-239	2	Smarcb1
4.08E-243	0.28438456	0.52	0.066	9.88E-239	2	Atoh8
7.14E-243	0.37258126	0.923	0.202	1.73E-238	2	Arhgef12
1.45E-242	0.25955816	0.847	0.171	3.51E-238	2	Sec11a
2.35E-242	1.71902049	0.997	0.527	5.70E-238	2	Gm10076
1.08E-241	0.63956505	0.956	0.236	2.61E-237	2	Rb1cc1
1.19E-241	0.45429659	0.939	0.211	2.89E-237	2	Tnk2
1.27E-241	0.48327309	0.888	0.192	3.07E-237	2	Taf1d
1.89E-241	0.41551001	0.933	0.211	4.59E-237	2	Vkorc1
4.86E-241	0.40369132	0.872	0.186	1.18E-236	2	Uba6
5.24E-241	0.43589763	0.6	0.092	1.27E-236	2	Arrdc4
1.71E-240	0.33317149	0.903	0.198	4.14E-236	2	Atxn10
2.30E-240	0.86151038	0.943	0.241	5.57E-236	2	Ugcg
2.51E-240	0.34050717	0.911	0.194	6.07E-236	2	Hars
7.13E-240	0.41933203	0.933	0.203	1.73E-235	2	Ccdc88a
1.12E-239	0.30720513	0.551	0.074	2.71E-235	2	Sorbs1
3.18E-239	0.30222798	0.898	0.188	7.71E-235	2	Dlst
2.12E-238	0.54709456	0.94	0.221	5.13E-234	2	Ergic1
2.38E-238	0.2501718	0.657	0.108	5.77E-234	2	Angptl4
4.45E-238	0.31764644	0.884	0.19	1.08E-233	2	Dars
9.31E-238	0.518125	0.852	0.185	2.26E-233	2	Nav2

2.46E-237	0.54866736	0.948	0.223	5.97E-233	2	Srpr
5.61E-237	0.60726478	0.945	0.223	1.36E-232	2	Peak1
2.89E-236	0.25369937	0.789	0.155	7.00E-232	2	Esf1
9.19E-236	0.25646614	0.608	0.096	2.23E-231	2	Slc1a5
1.29E-235	0.30437688	0.901	0.201	3.12E-231	2	Ift20
1.43E-235	0.44601238	0.938	0.212	3.48E-231	2	Dag1
1.96E-235	0.25336766	0.862	0.179	4.75E-231	2	Smarca4
3.05E-235	0.50764202	0.93	0.207	7.40E-231	2	Nop58
8.97E-235	0.42921314	0.935	0.211	2.18E-230	2	Ddx1
9.56E-235	0.37629335	0.879	0.19	2.32E-230	2	Wdr43
1.49E-233	0.33061317	0.91	0.201	3.62E-229	2	Tcf12
1.69E-233	1.552067	1	0.55	4.09E-229	2	Tmsb10
3.38E-233	1.16902305	0.997	0.343	8.19E-229	2	Cnn2
4.17E-233	0.26781111	0.878	0.188	1.01E-228	2	Anapc16
1.39E-232	0.34841681	0.919	0.204	3.37E-228	2	Galk1
3.00E-232	1.02124523	0.969	0.298	7.28E-228	2	Ifitm3
3.28E-232	0.58764986	0.958	0.237	7.94E-228	2	Yipf5
5.58E-232	0.2797226	0.481	0.057	1.35E-227	2	Trib3
1.00E-231	0.52622669	0.932	0.218	2.43E-227	2	Mybbp1a
1.56E-231	0.42947988	0.922	0.207	3.79E-227	2	Arhgap5
4.26E-231	0.54274564	0.906	0.203	1.03E-226	2	Prkar2b
7.83E-231	0.27344475	0.868	0.187	1.90E-226	2	Smad4

8.03E-231	0.56253316	0.831	0.177	1.95E-226	2	Ank
8.41E-231	1.19796813	0.999	0.394	2.04E-226	2	Cald1
1.06E-230	0.27362956	0.872	0.185	2.58E-226	2	Slc25a24
2.87E-230	0.26535578	0.817	0.165	6.97E-226	2	Bicd2
4.68E-230	0.77052204	0.965	0.264	1.13E-225	2	Pdap1
9.58E-230	0.25174062	0.872	0.185	2.32E-225	2	Psmd3
1.30E-229	0.31263294	0.721	0.135	3.15E-225	2	Slc20a1
3.71E-229	0.34632031	0.878	0.192	9.01E-225	2	Capn2
5.23E-229	0.45904756	0.932	0.22	1.27E-224	2	Uso1
5.77E-229	1.00266031	0.984	0.292	1.40E-224	2	Loxl3
7.05E-229	0.29648937	0.892	0.195	1.71E-224	2	Rbm28
6.76E-228	0.40591669	0.923	0.213	1.64E-223	2	Ganab
7.65E-228	0.45633453	0.916	0.214	1.86E-223	2	Mydgf
1.67E-227	0.55247151	0.827	0.177	4.05E-223	2	Slc16a3
1.76E-227	0.30724621	0.855	0.185	4.27E-223	2	Ero1l
4.88E-227	0.25548764	0.483	0.06	1.18E-222	2	Pdgfra
8.89E-227	0.29560652	0.866	0.191	2.16E-222	2	Myo18a
8.90E-227	0.80572629	0.994	0.281	2.16E-222	2	Sptbn1
1.05E-226	0.3918397	0.922	0.216	2.54E-222	2	Copg1
2.06E-226	0.25852875	0.856	0.189	5.00E-222	2	Usp22
2.17E-226	0.92274498	0.994	0.308	5.25E-222	2	Mfge8
4.46E-226	0.59893908	0.972	0.243	1.08E-221	2	Rcn2

1.63E-225	0.29316653	0.882	0.193	3.95E-221	2	Odc1
1.86E-225	0.26622069	0.868	0.186	4.50E-221	2	Pcm1
6.04E-225	0.57862067	0.94	0.239	1.47E-220	2	Smurf2
4.52E-224	0.45693621	0.93	0.224	1.10E-219	2	Snd1
4.80E-224	0.28236462	0.372	0.031	1.16E-219	2	Rarres1
3.02E-223	0.2689608	0.885	0.198	7.33E-219	2	Tspan3
3.57E-223	1.45141592	0.997	0.45	8.65E-219	2	Ccnd1
1.17E-222	0.59634713	0.927	0.236	2.84E-218	2	Eif4ebp1
1.63E-222	0.49284394	0.953	0.234	3.95E-218	2	Asap1
1.81E-222	0.5261433	0.946	0.231	4.38E-218	2	Asph
6.01E-222	0.49522742	0.722	0.143	1.46E-217	2	Hspb1
1.43E-221	0.255314	0.824	0.174	3.48E-217	2	Zmpste24
2.79E-221	0.45228844	0.709	0.14	6.76E-217	2	Grem1
4.72E-221	0.67737705	0.964	0.242	1.14E-216	2	Esyt2
7.56E-221	0.25927377	0.894	0.198	1.83E-216	2	Ebna1bp2
2.29E-220	0.26736235	0.414	0.043	5.56E-216	2	Meis2
3.10E-220	0.31200883	0.81	0.174	7.52E-216	2	Jak2
7.81E-220	0.26881086	0.881	0.194	1.89E-215	2	Usp47
7.96E-220	1.42855409	0.994	0.488	1.93E-215	2	Rps2
9.58E-220	0.25710925	0.878	0.196	2.32E-215	2	Tnrc6b
1.57E-219	0.88177131	0.993	0.301	3.81E-215	2	Lpp
5.27E-219	0.43355662	0.936	0.218	1.28E-214	2	Higd1a
1.20E-218	0.35603341	0.907	0.208	2.91E-214	2	Npm3

1.42E-218	0.25734274	0.894	0.204	3.45E-214	2	Usp16
1.87E-218	0.36793027	0.913	0.216	4.55E-214	2	Cops2
2.67E-218	0.37806934	0.89	0.204	6.48E-214	2	Ash1l
7.11E-218	0.37935465	0.929	0.215	1.72E-213	2	Stip1
1.57E-217	0.29783965	0.865	0.195	3.81E-213	2	Tpst2
2.55E-217	0.96638016	0.996	0.318	6.19E-213	2	11-Sep
4.21E-217	0.27609956	0.387	0.036	1.02E-212	2	Slc38a4
4.64E-217	0.29526999	0.897	0.203	1.13E-212	2	Tnfaip1
6.41E-217	0.27285027	0.894	0.204	1.55E-212	2	Eif3g
7.25E-217	0.42738102	0.741	0.151	1.76E-212	2	Gadd45g
1.95E-216	0.58048869	0.964	0.243	4.74E-212	2	Flnb
2.35E-216	0.33373927	0.439	0.051	5.70E-212	2	Sox9
4.39E-215	0.84070261	0.997	0.28	1.07E-210	2	Cavin1
5.66E-215	0.40676436	0.92	0.222	1.37E-210	2	Man1a2
8.53E-215	0.30967022	0.669	0.121	2.07E-210	2	Nid1
2.92E-214	0.26770484	0.436	0.051	7.08E-210	2	Unc5c
9.85E-214	0.44421245	0.926	0.22	2.39E-209	2	Stt3b
9.88E-214	0.45425311	0.999	0.668	2.40E-209	2	mt-Nd1
2.45E-213	0.26517091	0.882	0.2	5.93E-209	2	Ddx24
2.47E-213	1.2485759	1	0.614	5.98E-209	2	Ahnak
1.38E-212	0.50398976	0.432	0.05	3.35E-208	2	Pappa
2.92E-212	1.19891094	0.993	0.359	7.09E-208	2	Sdc4

4.38E-212	0.3072203	0.478	0.063	1.06E-207	2	Col5a3
6.79E-212	0.4568183<br>4	0.945	0.236	1.65E-207	2	Denr
7.01E-212	1.5167363<br>9	0.693	0.158	1.70E-207	2	Postn
7.26E-212	1.2242811<br>4	1	0.506	1.76E-207	2	Tpm1
7.43E-212	0.4319049<br>1	0.942	0.235	1.80E-207	2	Fam3c
5.45E-211	0.2537217<br>5	0.839	0.19	1.32E-206	2	Nrip1
5.70E-211	0.5263536<br>4	0.968	0.255	1.38E-206	2	Pdlim7
8.80E-211	0.2555138<br>4	0.923	0.214	2.13E-206	2	Map3k20
1.32E-210	0.3154945<br>9	0.475	0.063	3.21E-206	2	Eno3
1.41E-210	0.8145936	0.98	0.292	3.42E-206	2	Csrp1
2.05E-210	0.5594468<br>1	0.978	0.267	4.98E-206	2	Trim35
5.83E-210	0.4523277<br>7	0.946	0.241	1.41E-205	2	Zc3h15
7.35E-210	0.4164111	0.919	0.214	1.78E-205	2	Ttc39b
7.62E-210	0.3661461<br>6	0.92	0.228	1.85E-205	2	Sec13
1.31E-209	0.5715898<br>6	0.586	0.1	3.17E-205	2	Tnfrsf11b
1.50E-209	0.3445551<br>5	0.933	0.228	3.64E-205	2	Tbl1x
2.40E-209	0.2659727<br>5	0.826	0.182	5.82E-205	2	Ftsj3
3.33E-209	0.7341487<br>7	0.972	0.271	8.08E-205	2	Tpm2
3.52E-209	0.7108828<br>3	0.983	0.283	8.53E-205	2	Piezo1
7.76E-209	0.7828933<br>4	0.988	0.308	1.88E-204	2	Txndc5
2.93E-208	0.2803905<br>1	0.89	0.208	7.11E-204	2	2810004N23Ri<br>k
3.75E-208	0.9819046<br>8	0.988	0.332	9.10E-204	2	Myadm
4.52E-208	0.2608417<br>9	0.831	0.187	1.10E-203	2	Myc

4.54E-208	0.50762517	0.962	0.253	1.10E-203	2	Ddost
2.30E-207	0.52415839	0.935	0.243	5.59E-203	2	Eif3j1
3.94E-207	0.60288273	0.669	0.129	9.55E-203	2	Hmga2
5.61E-207	0.84456049	0.983	0.323	1.36E-202	2	Rpl13a
1.67E-206	1.03204322	0.994	0.368	4.05E-202	2	Pdia3
2.72E-206	0.99440719	0.701	0.156	6.60E-202	2	Rpl39l
4.86E-206	0.35670699	0.92	0.226	1.18E-201	2	Cdk4
5.36E-206	1.63572568	0.635	0.131	1.30E-201	2	Serpinb2
1.41E-205	0.2836615	0.914	0.22	3.41E-201	2	Pip5k1a
1.97E-205	0.31691881	0.933	0.234	4.78E-201	2	Ptov1
2.82E-205	1.18013246	0.996	0.496	6.84E-201	2	Rps18
7.92E-205	0.29278793	0.882	0.208	1.92E-200	2	Samd4b
8.36E-205	0.34060209	1	0.751	2.03E-200	2	mt-Cytb
1.13E-204	0.79435155	0.968	0.288	2.73E-200	2	Raph1
1.14E-204	0.84594227	0.997	0.328	2.76E-200	2	Crtap
3.06E-204	0.29782129	0.885	0.213	7.42E-200	2	Herpud1
5.48E-204	0.28016312	0.574	0.097	1.33E-199	2	Zfp503
7.36E-204	0.3796208	0.916	0.225	1.78E-199	2	Tmem5
1.76E-203	0.36062499	0.375	0.038	4.26E-199	2	Scube3
6.16E-203	0.29094961	0.878	0.199	1.49E-198	2	Tubb4b
2.10E-202	0.30419679	0.917	0.224	5.08E-198	2	Psmc5
3.04E-202	0.47718162	0.948	0.253	7.36E-198	2	Aimp1
8.15E-201	0.6216044	0.981	0.286	1.98E-196	2	Uqcc2

4.41E-200	1.35354629	0.991	0.479	1.07E-195	2	Rpl35
4.55E-200	0.48466692	0.959	0.258	1.10E-195	2	Wwp2
9.61E-200	0.27626215	0.903	0.216	2.33E-195	2	Prrc2b
3.34E-199	1.146063	1	0.924	8.10E-195	2	Rpl41
5.00E-199	0.78959373	0.985	0.336	1.21E-194	2	Slc25a4
5.32E-199	0.39834704	0.525	0.084	1.29E-194	2	Ptx3
1.84E-198	0.43568111	0.953	0.244	4.46E-194	2	Sfxn1
2.08E-198	0.51214226	0.975	0.275	5.04E-194	2	Map4
3.11E-198	0.67027242	0.99	0.307	7.54E-194	2	Ddb1
3.59E-197	0.31822931	0.938	0.232	8.70E-193	2	Mettl9
5.06E-197	1.00779123	0.985	0.392	1.23E-192	2	Rrbp1
1.43E-195	0.68265549	0.984	0.295	3.46E-191	2	Prkcsh
3.36E-194	0.28281883	0.927	0.24	8.14E-190	2	Park7
5.06E-194	0.27056252	0.772	0.169	1.23E-189	2	Pds5b
7.87E-194	0.25493609	0.891	0.221	1.91E-189	2	Bptf
1.75E-193	0.25636383	0.881	0.212	4.24E-189	2	Atxn1
2.56E-193	0.42489985	0.956	0.256	6.20E-189	2	Iars
5.20E-192	0.5882282	0.969	0.27	1.26E-187	2	Mlec
2.75E-191	0.64366659	0.987	0.315	6.68E-187	2	Sec31a
3.09E-191	0.7187888	0.985	0.31	7.50E-187	2	Glg1
5.86E-191	0.34696624	0.901	0.227	1.42E-186	2	Hyou1
5.92E-191	0.72663033	0.991	0.316	1.43E-186	2	Ostc
8.47E-191	0.90488962	0.999	0.413	2.05E-186	2	Hdlbp

9.65E-191	0.67695278	0.972	0.286	2.34E-186	2	Igfbp7
1.11E-189	0.95246543	0.991	0.408	2.69E-185	2	Rpl22l1
1.39E-189	0.99961523	0.997	0.49	3.36E-185	2	P4hb
1.67E-189	0.48462641	0.359	0.037	4.06E-185	2	Sfrp4
5.01E-189	1.25271926	1	0.777	1.21E-184	2	Rps20
5.58E-189	0.52635617	0.955	0.264	1.35E-184	2	Foxp1
7.45E-189	0.39865177	0.907	0.236	1.81E-184	2	Gnl3
1.60E-188	0.77305803	0.977	0.333	3.88E-184	2	Nfe2l1
3.01E-188	0.3186337	0.939	0.242	7.31E-184	2	Mrpl17
5.90E-188	0.31148442	0.917	0.24	1.43E-183	2	Ndufa13
6.01E-188	0.37993129	0.946	0.252	1.46E-183	2	Top2b
8.38E-188	0.51858766	0.972	0.283	2.03E-183	2	Sar1a
1.38E-187	0.30681853	0.916	0.24	3.36E-183	2	Prdx6
1.42E-187	0.25112439	0.913	0.234	3.44E-183	2	Selenon
8.63E-187	0.36170263	0.922	0.231	2.09E-182	2	Pgrmc1
1.67E-186	0.26040132	0.356	0.038	4.05E-182	2	Rasl11a
1.98E-186	0.8056689	0.98	0.321	4.80E-182	2	Ahcyl1
6.85E-186	0.31285107	0.952	0.251	1.66E-181	2	Cops6
1.28E-185	0.67764399	0.981	0.304	3.10E-181	2	Ugdh
2.73E-185	1.51139914	0.999	0.899	6.62E-181	2	Malat1
3.86E-185	0.33076961	0.904	0.236	9.36E-181	2	Ankrd11
7.46E-185	0.47422079	0.93	0.26	1.81E-180	2	Ssr4

1.01E-184	0.38955195	0.935	0.26	2.44E-180	2	Copb2
2.54E-184	0.31235263	0.926	0.234	6.15E-180	2	Fubp1
4.10E-184	0.74070389	0.984	0.317	9.94E-180	2	Nudt4
1.02E-183	0.28104781	0.353	0.037	2.48E-179	2	Npnt
1.47E-183	0.77161065	0.493	0.084	3.56E-179	2	Col3a1
1.52E-183	0.97581735	0.994	0.469	3.68E-179	2	Eef1g
1.71E-183	0.30541516	0.359	0.039	4.16E-179	2	Asb4
2.24E-183	0.36386079	0.936	0.248	5.43E-179	2	Fyttd1
3.21E-183	0.4361259	0.955	0.256	7.78E-179	2	Nfic
4.88E-183	0.64343049	0.592	0.12	1.18E-178	2	Rps15a-ps8
6.90E-183	0.7369571	0.968	0.325	1.67E-178	2	S100a10
1.18E-182	0.26739065	0.959	0.259	2.86E-178	2	Smim10l1
1.19E-182	0.41728483	0.913	0.248	2.89E-178	2	Edf1
2.31E-182	0.34218769	0.919	0.241	5.60E-178	2	Lsm12
6.80E-182	0.44525054	0.952	0.268	1.65E-177	2	Nenf
1.24E-181	0.28578185	0.278	0.019	3.01E-177	2	Pcdh20
1.70E-181	0.25797128	0.906	0.237	4.12E-177	2	Rhoq
2.67E-181	0.69299437	0.969	0.298	6.48E-177	2	Ybx3
6.69E-181	0.3663759	0.964	0.259	1.62E-176	2	Nudcd2
6.49E-180	0.88697018	0.967	0.364	1.57E-175	2	Tagln
9.47E-180	0.49981273	0.965	0.276	2.30E-175	2	Myo1c
2.83E-179	0.2741503	0.913	0.237	6.87E-175	2	Acbd3
4.07E-179	1.09530116	0.984	0.416	9.88E-175	2	Rpl36

4.68E-179	0.38527778	0.939	0.257	1.14E-174	2	Cdc37
5.22E-179	0.39421085	0.952	0.268	1.26E-174	2	Huwe1
1.57E-178	0.25965534	0.907	0.234	3.80E-174	2	Psma4
1.93E-178	1.14623066	1	0.761	4.69E-174	2	S100a6
5.37E-178	0.57694579	0.92	0.254	1.30E-173	2	Slc29a1
5.66E-178	0.49055295	0.968	0.293	1.37E-173	2	Sars
6.33E-178	0.60503187	0.983	0.322	1.53E-173	2	Mbnl2
2.18E-177	1.02709272	1	0.756	5.28E-173	2	Vim
3.38E-177	0.50334658	0.969	0.284	8.18E-173	2	Gorasp2
6.81E-177	0.93044551	1	0.422	1.65E-172	2	Lmna
7.99E-176	1.00047101	1	0.554	1.94E-171	2	Bsg
1.10E-175	0.43406656	0.945	0.273	2.67E-171	2	Aars
3.56E-175	1.14378745	0.996	0.542	8.62E-171	2	Rps19
1.04E-174	0.25330168	0.347	0.039	2.51E-170	2	Csmd1
4.22E-173	0.35501925	0.908	0.248	1.02E-168	2	Plxna1
4.91E-173	0.38885152	0.952	0.269	1.19E-168	2	Sec63
5.04E-173	0.25885093	0.903	0.242	1.22E-168	2	Yipf3
1.20E-172	0.29775879	0.962	0.265	2.90E-168	2	Oat
3.01E-172	0.31027914	0.938	0.26	7.29E-168	2	Tomm5
4.19E-172	0.29056885	0.94	0.258	1.01E-167	2	Kif1b
6.83E-172	0.35673835	0.914	0.253	1.66E-167	2	Atp5g1

4.42E-171	1.02855928	1	0.668	1.07E-166	2	Itgb1
1.82E-170	0.86645248	0.984	0.386	4.41E-166	2	Rpl21
1.73E-169	0.52337115	0.99	0.32	4.20E-165	2	Tulp4
2.60E-169	2.16152391	1	0.925	6.30E-165	2	Spp1
3.55E-169	0.96850644	0.996	0.513	8.60E-165	2	Rpl10a
7.24E-169	1.20601945	0.988	0.553	1.76E-164	2	Acta2
1.27E-168	0.9317898	0.994	0.443	3.09E-164	2	Rps7
9.16E-168	0.56260108	0.971	0.304	2.22E-163	2	Stt3a
1.09E-167	0.67391954	0.98	0.323	2.65E-163	2	Eif4g1
2.14E-167	1.06199281	0.999	0.747	5.19E-163	2	Rpl35a
2.62E-167	0.26590341	0.911	0.246	6.35E-163	2	Rwdd1
1.24E-166	1.08613576	0.985	0.441	3.01E-162	2	Rpl12
3.32E-166	0.40896295	0.266	0.02	8.05E-162	2	Ptn
3.24E-165	0.37222405	0.938	0.272	7.85E-161	2	Nktr
1.22E-164	0.34980042	0.885	0.235	2.95E-160	2	Tubb6
1.55E-164	0.59009178	0.953	0.318	3.77E-160	2	Mrpl52
5.48E-164	1.03810664	1	0.814	1.33E-159	2	Rps28
7.31E-164	1.06087063	0.997	0.55	1.77E-159	2	Rpl6
1.94E-163	0.29826028	0.943	0.271	4.71E-159	2	Clint1
2.61E-163	0.56451235	0.967	0.306	6.32E-159	2	Tspan4
4.15E-163	0.37858088	0.939	0.274	1.01E-158	2	Atp5d
9.14E-163	0.56738067	0.999	0.817	2.22E-158	2	mt-Rnr2

1.64E-162	0.25417955	0.913	0.254	3.97E-158	2	Sel1l
1.70E-162	0.46223969	0.936	0.28	4.11E-158	2	Tmed9
2.68E-162	0.96377002	0.996	0.494	6.49E-158	2	Rpl36a
6.21E-162	0.60906154	0.988	0.329	1.51E-157	2	Rhoc
6.93E-162	0.2731036	0.949	0.269	1.68E-157	2	Leprotl1
9.42E-162	0.98738296	1	0.553	2.28E-157	2	Actn1
1.82E-161	0.3993261	0.943	0.288	4.42E-157	2	Ndufb9
1.96E-161	0.28514192	0.955	0.272	4.74E-157	2	Dcun1d5
2.20E-161	1.08604522	0.991	0.494	5.33E-157	2	Rps15a
8.22E-161	0.38593555	0.961	0.29	1.99E-156	2	Ipo5
4.08E-160	0.72704755	0.996	0.454	9.89E-156	2	S100a11
1.07E-159	1.12075168	0.996	0.617	2.59E-155	2	Rps27
1.44E-159	0.496371	0.949	0.298	3.49E-155	2	Rpl15
1.52E-159	0.86507968	0.397	0.061	3.68E-155	2	Gzme
1.72E-159	0.33675271	0.878	0.249	4.16E-155	2	Mthfd2
3.86E-158	0.27212852	0.891	0.248	9.36E-154	2	Mrps21
6.73E-158	0.80562265	1	0.436	1.63E-153	2	Rock2
1.81E-157	0.36774865	0.938	0.276	4.39E-153	2	Tmem234
2.78E-157	0.30539499	0.949	0.279	6.74E-153	2	Vdac3
1.87E-156	0.29477921	0.932	0.272	4.54E-152	2	Rabac1
3.25E-156	0.97823291	0.996	0.523	7.89E-152	2	Rpl34
1.38E-155	0.42268319	0.975	0.301	3.34E-151	2	Arcn1
1.85E-155	0.99863916	1	0.691	4.48E-151	2	Hsp90b1

2.33E-155	0.71497034	0.993	0.367	5.65E-151	2	P4ha1
3.52E-155	0.29703161	0.964	0.284	8.53E-151	2	Psmd6
5.38E-155	0.28917711	0.265	0.022	1.30E-150	2	Cdsn
1.31E-154	0.86896233	1	0.769	3.18E-150	2	Lgals1
1.92E-154	0.29307651	0.959	0.288	4.65E-150	2	Cast
4.26E-154	0.30513986	0.935	0.271	1.03E-149	2	Abcc1
4.98E-154	0.40788503	0.955	0.293	1.21E-149	2	Eif1ax
6.60E-154	0.32983635	0.965	0.291	1.60E-149	2	Srp19
7.36E-154	1.0038222	0.999	0.608	1.78E-149	2	Rpl28
7.62E-154	1.2007364	0.993	0.516	1.85E-149	2	Cyr61
7.95E-154	0.88924324	0.978	0.432	1.93E-149	2	Rps13
1.59E-153	0.33553055	0.945	0.279	3.85E-149	2	Gpx4
1.93E-153	0.29231465	0.916	0.268	4.67E-149	2	Krtcap2
2.71E-153	0.31499682	0.891	0.257	6.58E-149	2	Tmem258
3.02E-153	0.71424085	1	0.876	7.32E-149	2	Rpsa
3.38E-153	0.54180684	0.953	0.319	8.20E-149	2	2410006H16Rik
4.14E-153	0.97340403	0.994	0.62	1.00E-148	2	Rpl13
4.63E-153	0.46980597	0.953	0.298	1.12E-148	2	Pdlim5
5.75E-153	0.27068989	0.938	0.277	1.39E-148	2	Srp72
6.18E-153	0.3235837	0.958	0.281	1.50E-148	2	Gtf2h5
2.28E-152	0.43162671	0.967	0.301	5.54E-148	2	Eif1a
3.19E-152	0.98787473	1	0.531	7.75E-148	2	Pdia6
3.93E-152	0.899672	1	0.861	9.53E-148	2	Rps12

5.69E-152	0.75725405	0.996	0.391	1.38E-147	2	Tns3
2.41E-151	0.45805498	0.278	0.027	5.83E-147	2	Mcpt8
8.36E-151	0.88312781	0.98	0.437	2.03E-146	2	Rpl10
1.09E-150	1.6753563	0.999	0.664	2.65E-146	2	Thbs1
1.20E-150	0.38354443	0.562	0.122	2.92E-146	2	Rgs16
2.48E-150	0.81135133	1	0.9	6.01E-146	2	Rps8
2.99E-150	0.2524697	0.901	0.254	7.24E-146	2	Arid5b
3.20E-150	0.96862994	0.991	0.428	7.76E-146	2	Cited2
7.43E-149	0.45729143	0.98	0.324	1.80E-144	2	Uba1
1.27E-147	0.34231915	0.948	0.288	3.08E-143	2	S100a13
2.05E-147	0.89757331	0.999	0.587	4.96E-143	2	Flna
2.65E-147	0.63093638	0.987	0.387	6.42E-143	2	Fndc3b
7.89E-147	0.92670462	0.999	0.598	1.91E-142	2	Sdc3
8.91E-147	0.49468731	0.978	0.347	2.16E-142	2	Nsa2
1.30E-146	0.57801295	0.993	0.363	3.16E-142	2	Ccng1
8.21E-146	0.94613966	0.98	0.379	1.99E-141	2	Lbh
1.90E-145	0.3644228	0.961	0.299	4.61E-141	2	Rab6a
3.73E-145	0.27466	0.948	0.28	9.04E-141	2	Snrpg
3.95E-145	0.31693162	0.965	0.291	9.57E-141	2	Strn3
3.96E-145	0.84740145	0.999	0.582	9.60E-141	2	Myh9
6.08E-145	0.31325936	0.419	0.073	1.48E-140	2	Crlf1
7.80E-145	0.59386699	0.983	0.356	1.89E-140	2	Ppp1r14b
8.18E-145	0.75797799	0.991	0.408	1.98E-140	2	Wls

1.51E-144	0.26260948	0.493	0.098	3.67E-140	2	Lrrc32
1.95E-144	0.44962287	0.936	0.302	4.72E-140	2	Zyx
3.83E-144	0.8038689	1	0.583	9.28E-140	2	Eif2s2
7.44E-144	0.28872695	0.927	0.275	1.80E-139	2	Magt1
9.53E-144	1.08325291	1	0.617	2.31E-139	2	Hspa5
2.98E-143	0.28143386	0.923	0.276	7.22E-139	2	Sec62
3.82E-143	0.27529869	0.951	0.295	9.26E-139	2	Eif3i
4.07E-143	0.38846092	0.968	0.304	9.87E-139	2	Psmd11
1.34E-142	0.48214768	0.988	0.351	3.26E-138	2	Zfhx3
1.55E-142	0.31697599	0.968	0.303	3.75E-138	2	Tmem167
4.22E-142	0.8811252	0.996	0.526	1.02E-137	2	Rpl18
5.29E-142	0.92795651	0.994	0.476	1.28E-137	2	Id3
9.48E-142	0.71648614	1	0.691	2.30E-137	2	Gnas
1.25E-141	0.85707262	0.991	0.535	3.03E-137	2	Rps17
9.42E-141	0.92682097	1	0.676	2.29E-136	2	Rpl37
3.63E-140	0.27453096	0.932	0.284	8.80E-136	2	Cux1
1.00E-139	0.5794834	0.581	0.14	2.44E-135	2	Ptgs2
1.15E-139	0.35269508	0.94	0.298	2.79E-135	2	Anxa6
2.04E-139	0.67188734	0.996	0.425	4.94E-135	2	Sec61b
6.00E-139	0.55309961	0.328	0.046	1.45E-134	2	Cyp26b1
1.27E-138	0.52885901	0.987	0.374	3.07E-134	2	Arf4
6.78E-138	0.73932817	1	0.779	1.64E-133	2	Rps10
8.83E-138	0.69958628	0.996	0.517	2.14E-133	2	Tpm4

1.17E-135	0.81520933	0.994	0.505	2.84E-131	2	Rpl9
4.29E-135	0.30598394	0.961	0.306	1.04E-130	2	Eef1d
1.09E-134	0.29241763	0.952	0.302	2.64E-130	2	1110004F10Rik
1.95E-134	0.2963534	0.952	0.29	4.72E-130	2	Rbbp7
3.14E-134	1.26719251	0.337	0.051	7.62E-130	2	Ibsp
5.00E-134	0.66875211	0.999	0.474	1.21E-129	2	Pebp1
1.87E-133	0.46510785	0.984	0.344	4.55E-129	2	Dad1
4.09E-133	0.76964218	1	0.691	9.91E-129	2	Anxa2
9.72E-133	0.81430637	0.991	0.485	2.36E-128	2	Rpl17
1.41E-132	0.71940062	1	0.732	3.41E-128	2	Rplp0
2.42E-132	0.47322745	0.975	0.365	5.88E-128	2	Anxa1
5.44E-132	0.36341958	0.951	0.321	1.32E-127	2	Elob
5.13E-131	0.76025349	0.999	0.568	1.24E-126	2	Rpl27a
5.50E-131	0.4282052	0.988	0.356	1.33E-126	2	Rexo2
7.62E-131	0.46900917	0.93	0.3	1.85E-126	2	Scaf11
9.84E-131	0.58242539	0.988	0.376	2.39E-126	2	Rbpj
3.26E-130	0.88834901	0.997	0.618	7.90E-126	2	Rps23
2.13E-129	0.39645951	0.967	0.334	5.16E-125	2	Lrrc59
2.83E-129	0.54258633	0.99	0.395	6.87E-125	2	Slc38a2
3.09E-129	0.2539596	0.971	0.312	7.49E-125	2	Uggt1
3.18E-129	0.73580596	1	0.574	7.70E-125	2	Rpl3
2.59E-128	0.29634281	0.943	0.3	6.29E-124	2	Dnajc3
3.68E-128	0.31988939	0.961	0.305	8.93E-124	2	Smarcc1

5.50E-128	0.36122726	0.959	0.323	1.33E-123	2	Ddx21
1.38E-127	0.4216122	0.974	0.339	3.35E-123	2	Cct3
2.32E-127	0.32161921	0.949	0.314	5.62E-123	2	Ilk
3.65E-127	0.65127791	1	0.746	8.86E-123	2	Npm1
6.54E-127	0.74535524	1	0.827	1.59E-122	2	Rpl39
9.74E-126	0.69770519	1	0.756	2.36E-121	2	Eef1b2
1.08E-125	0.8865417	0.996	0.636	2.61E-121	2	Rpl18a
1.32E-125	1.01993887	0.991	0.445	3.20E-121	2	Nrp2
1.92E-125	0.73440913	1	0.918	4.66E-121	2	Rpl37a
3.10E-125	0.70797299	1	0.455	7.53E-121	2	Mmp14
4.90E-125	0.26661509	0.974	0.309	1.19E-120	2	Dhx15
9.65E-125	0.6721205	0.996	0.566	2.34E-120	2	Rpl4
7.59E-124	0.45292441	0.997	0.45	1.84E-119	2	Fkbp1a
1.83E-123	0.61076762	0.997	0.457	4.43E-119	2	Clic4
2.99E-123	0.29629949	0.975	0.336	7.26E-119	2	Llph
5.81E-123	0.25460191	0.792	0.238	1.41E-118	2	Adh5
8.69E-123	0.60719492	0.987	0.456	2.11E-118	2	Uqcrq
9.14E-123	0.31387633	0.967	0.335	2.22E-118	2	Sh3kbp1
1.14E-122	0.72283108	0.997	0.646	2.77E-118	2	Rpl14
1.75E-122	0.28958229	0.974	0.319	4.25E-118	2	Rsl1d1
1.13E-121	0.55560842	0.978	0.428	2.75E-117	2	Nars
3.68E-121	0.6000447	0.988	0.399	8.93E-117	2	H13
3.97E-121	0.2810352	0.968	0.332	9.63E-117	2	Lrrc58

8.24E-121	0.6830975<br>3	0.999	0.527	2.00E-116	2	Gas5
2.17E-120	0.6648892<br>2	0.999	0.688	5.26E-116	2	Rpl23a
5.48E-120	0.4196841<br>4	0.981	0.36	1.33E-115	2	Prdx2
1.48E-119	0.5654223	0.987	0.42	3.60E-115	2	Gapdh
1.01E-118	0.6665094<br>2	0.991	0.426	2.44E-114	2	Cdkn1a
1.98E-118	0.2608980<br>5	0.961	0.309	4.80E-114	2	Tnpo3
5.67E-118	0.3491771<br>6	0.942	0.343	1.37E-113	2	Arl5a
8.53E-118	0.5605642<br>8	1	0.952	2.07E-113	2	Eef1a1
4.09E-117	0.7417020<br>8	1	0.836	9.92E-113	2	Rpl38
8.73E-117	0.5352073<br>9	0.994	0.476	2.12E-112	2	Hsp90aa1
3.82E-116	0.6205317<br>5	0.51	0.129	9.27E-112	2	Ccl7
9.76E-116	0.3374226<br>5	0.869	0.287	2.37E-111	2	Bnip3
1.04E-115	0.5669852<br>8	1	0.584	2.51E-111	2	Dstn
1.61E-115	0.6691961<br>4	0.997	0.48	3.90E-111	2	Rpn1
3.90E-115	0.3868688<br>8	0.984	0.352	9.45E-111	2	Cbx3
4.14E-115	0.3194428<br>5	0.965	0.344	1.00E-110	2	Sec61a1
5.15E-115	0.2649136<br>6	0.978	0.347	1.25E-110	2	Snhg8
1.17E-114	0.3037113<br>8	0.959	0.337	2.83E-110	2	C1qbp
4.07E-114	0.6021687<br>2	0.991	0.428	9.86E-110	2	Ssr1
2.76E-113	0.3568937<br>9	0.975	0.352	6.70E-109	2	Cdv3
1.38E-112	0.4951611<br>7	0.993	0.384	3.34E-108	2	Cnih1
4.91E-112	0.3845029<br>9	0.975	0.348	1.19E-107	2	Tnrc18

7.45E-112	0.4329407 2	0.961	0.391	1.81E-107	2	Cox7c
1.91E-111	0.5027958 5	0.978	0.428	4.62E-107	2	Rpl31
2.24E-111	0.6951455 2	0.999	0.653	5.43E-107	2	Rps27l
3.45E-111	0.3575672 5	0.775	0.251	8.37E-107	2	Col6a3
4.53E-111	0.5806113	0.999	0.465	1.10E-106	2	Hmgn1
2.51E-110	0.2750758 8	0.958	0.33	6.08E-106	2	Psmc3
2.64E-110	0.4835611 9	1	0.967	6.41E-106	2	Rplp1
3.37E-110	0.5040789 3	0.994	0.443	8.16E-106	2	Eif3e
3.03E-109	0.3593853 8	0.981	0.381	7.34E-105	2	Sfr1
2.11E-108	0.3611371 1	0.991	0.39	5.11E-104	2	Metap2
2.55E-108	0.3347975 7	0.991	0.356	6.18E-104	2	Prmt1
4.22E-107	0.2923827 1	0.974	0.364	1.02E-102	2	Rtraf
2.22E-106	0.7443172 6	0.999	0.689	5.39E-102	2	Rps16
5.15E-105	0.6297266	1	0.704	1.25E-100	2	Rps3
5.46E-105	0.2724100 7	0.971	0.344	1.32E-100	2	Kpnb1
1.30E-104	0.5024692 3	0.987	0.462	3.14E-100	2	Rpl5
3.01E-104	0.6078212 5	0.993	0.551	7.30E-100	2	Rpl7
6.57E-104	0.2695998 1	0.945	0.349	1.59E-99	2	Ost4
5.02E-103	0.5033432 1	0.996	0.421	1.22E-98	2	Kdelr1
5.98E-103	0.4245531 4	0.625	0.196	1.45E-98	2	Egr1
1.93E-102	0.6403777 6	1	0.631	4.69E-98	2	Tubb5
2.06E-102	0.5226909 9	1	0.901	5.00E-98	2	Rps24

2.26E-102	0.40360456	0.983	0.4	5.47E-98	2	Tcp1
7.38E-102	0.66140944	0.991	0.547	1.79E-97	2	Rpl30
4.67E-100	0.25409889	0.257	0.039	1.13E-95	2	Bmp2
1.30E-99	0.4678791	1	0.921	3.15E-95	2	Rplp2
2.69E-99	0.49998569	0.999	0.471	6.53E-95	2	Dap
2.43E-98	0.4351335	0.987	0.436	5.89E-94	2	Hspd1
4.26E-98	0.27566675	0.972	0.376	1.03E-93	2	Dynlrb1
9.71E-98	0.61407342	0.994	0.601	2.36E-93	2	Rpl7a
1.31E-97	1.15060772	0.959	0.551	3.18E-93	2	Ctgf
2.95E-97	0.28066028	0.974	0.367	7.15E-93	2	Gspt1
1.18E-96	0.51975448	0.994	0.48	2.86E-92	2	Set
1.10E-95	0.36389811	0.972	0.387	2.66E-91	2	Mtdh
1.35E-95	0.59576489	0.997	0.625	3.28E-91	2	Rps11
1.15E-94	0.56449564	1	0.619	2.79E-90	2	Pkm
2.12E-94	0.47580612	0.993	0.478	5.14E-90	2	Tcf4
2.70E-94	0.5200477	0.996	0.53	6.54E-90	2	Morf4l2
2.83E-94	0.28040563	0.975	0.377	6.86E-90	2	Ube2r2
1.87E-93	0.47600359	0.993	0.491	4.53E-89	2	Hspe1
1.95E-93	0.52820101	0.997	0.459	4.73E-89	2	Colgalt1
3.25E-93	0.42993835	1	0.921	7.89E-89	2	Rps26
4.21E-93	0.31281647	0.987	0.417	1.02E-88	2	Cct2
5.12E-93	0.56112236	1	0.733	1.24E-88	2	Rpl22
3.40E-92	0.36922306	0.254	0.041	8.26E-88	2	Igfbp5

4.30E-92	0.4479418	0.997	0.486	1.04E-87	2	Cct6a
5.97E-92	0.2678238 9	0.983	0.392	1.45E-87	2	Cct5
9.53E-92	0.4816858 5	0.99	0.483	2.31E-87	2	Rps6
1.53E-91	0.4204516 4	0.981	0.43	3.72E-87	2	Tln1
2.33E-90	0.5412340 9	0.993	0.544	5.65E-86	2	Rpl11
4.82E-89	0.3122241 2	0.983	0.412	1.17E-84	2	Mrfap1
1.36E-88	0.2765048 4	0.98	0.412	3.30E-84	2	Nisch
2.80E-88	0.3040425 4	0.968	0.39	6.80E-84	2	Emp3
1.20E-87	0.3584701 5	0.974	0.432	2.91E-83	2	Bola2
1.97E-87	0.6472689 4	1	0.647	4.78E-83	2	Actn4
4.85E-87	0.4182731	1	0.894	1.18E-82	2	Hsp90ab1
7.99E-87	0.3038713 7	0.988	0.395	1.94E-82	2	Celf1
3.91E-86	0.5936204 7	0.991	0.599	9.47E-82	2	Rpl26
8.94E-86	0.2771405 3	0.987	0.396	2.17E-81	2	Hnrnpa1
1.35E-85	0.5261247 5	0.997	0.581	3.28E-81	2	Rpl29
4.81E-85	0.3378040 7	0.983	0.394	1.17E-80	2	Prrc2c
1.59E-84	0.5896485 5	1	0.597	3.86E-80	2	Cd63
4.87E-84	0.5268560 9	0.999	0.536	1.18E-79	2	App
5.85E-84	0.2714623 5	0.987	0.405	1.42E-79	2	Psma7
6.06E-84	0.5593456 2	1	0.603	1.47E-79	2	Hspa9
7.08E-84	0.5232231 5	1	0.809	1.72E-79	2	Rps27a
8.14E-84	0.3567325 4	0.988	0.437	1.97E-79	2	Swi5

1.58E-83	0.4817369<br>8	0.999	0.503	3.83E-79	2	Rpn2
2.44E-83	0.4375385<br>1	1	0.662	5.92E-79	2	Ncl
2.52E-83	0.3164093<br>4	0.969	0.387	6.10E-79	2	Axl
7.85E-83	0.2911572<br>7	0.978	0.408	1.90E-78	2	Lmo4
1.35E-81	0.5078199<br>7	1	0.89	3.27E-77	2	Rps21
2.93E-81	0.3829468<br>1	0.99	0.438	7.11E-77	2	Selenos
1.15E-80	0.3706869<br>7	0.983	0.45	2.78E-76	2	Rpl27
1.51E-80	0.2734086<br>9	0.984	0.427	3.67E-76	2	Srrm2
1.54E-80	0.5158710<br>9	1	0.863	3.74E-76	2	Rpl23
2.23E-80	0.3242209<br>9	0.984	0.424	5.40E-76	2	Mcfd2
3.03E-80	0.3748564<br>4	0.994	0.458	7.35E-76	2	Map4k4
3.65E-80	0.2963442<br>5	0.987	0.441	8.85E-76	2	Tcf25
4.04E-80	0.4651899<br>8	1	0.813	9.80E-76	2	Rps4x
5.87E-80	0.3220983<br>6	0.985	0.418	1.42E-75	2	Sfpq
9.18E-80	0.3523440<br>7	0.993	0.466	2.23E-75	2	Eif3c
2.18E-79	0.5521640<br>2	1	0.755	5.30E-75	2	Rps3a1
8.82E-79	0.4866135<br>9	1	0.673	2.14E-74	2	Ldha
1.47E-77	0.4340175<br>4	1	0.883	3.57E-73	2	Myl6
2.18E-77	0.4225912<br>3	0.993	0.518	5.28E-73	2	Ran
5.32E-77	0.2659692<br>8	0.988	0.415	1.29E-72	2	G3bp1
5.34E-77	0.4613172<br>2	1	0.824	1.29E-72	2	Rps5

6.86E-77	0.43171218	0.997	0.536	1.66E-72	2	Uqcr11
1.56E-76	0.30585203	0.993	0.44	3.78E-72	2	Psmb4
1.25E-74	0.52403404	1	0.82	3.04E-70	2	Rpl32
6.21E-73	0.45185025	0.997	0.645	1.51E-68	2	Rps25
2.77E-70	0.43864142	1	0.66	6.71E-66	2	Serbp1
9.01E-70	0.26514232	0.985	0.429	2.18E-65	2	Prelid1
1.38E-69	0.25809247	0.977	0.459	3.35E-65	2	Neat1
3.26E-69	0.40943546	1	0.64	7.91E-65	2	Btf3
4.84E-69	0.30746693	0.99	0.466	1.17E-64	2	Laptm4a
2.91E-68	0.47270621	1	0.663	7.06E-64	2	Mif
2.40E-67	0.51227016	1	0.668	5.82E-63	2	Calr
7.42E-67	0.42811135	1	0.764	1.80E-62	2	Eef2
8.11E-67	0.35388586	1	0.917	1.97E-62	2	Rack1
3.27E-66	0.4169717	0.987	0.584	7.93E-62	2	Serf2
3.73E-66	0.35209243	0.999	0.503	9.04E-62	2	Tmed2
5.77E-64	0.39977668	0.997	0.545	1.40E-59	2	Dynll1
4.38E-63	0.28488025	0.996	0.48	1.06E-58	2	Rab2a
4.47E-63	0.40801276	1	0.585	1.08E-58	2	Nme1
5.36E-63	0.31935029	0.991	0.553	1.30E-58	2	Atp5e
1.24E-57	0.39337569	1	0.638	3.00E-53	2	Ptms
1.11E-56	0.3142418	0.997	0.524	2.69E-52	2	Tpi1
7.14E-56	0.50820573	0.66	0.272	1.73E-51	2	Ccl2
1.24E-54	0.322347	1	0.655	3.01E-50	2	Ywhae

6.77E-54	0.3295197<br>1	1	0.92	1.64E-49	2	Rps29
2.09E-52	0.2510542<br>4	0.997	0.485	5.06E-48	2	Surf4
5.81E-52	0.3571102<br>5	1	0.598	1.41E-47	2	Ssr3
1.67E-51	0.3001523<br>7	1	0.853	4.05E-47	2	Rps15
1.69E-51	0.3076784<br>3	1	0.818	4.09E-47	2	Rpl8
2.03E-50	0.3185850<br>2	0.999	0.595	4.92E-46	2	Vcp
4.11E-50	0.3943588<br>4	1	0.971	9.96E-46	2	Actg1
4.29E-50	0.2696772<br>7	0.991	0.526	1.04E-45	2	Anxa3
2.33E-47	0.3060208<br>1	1	0.862	5.65E-43	2	Rpl19
9.34E-47	0.2734482<br>2	0.994	0.549	2.26E-42	2	Cct8
8.45E-43	0.2673884<br>4	1	0.75	2.05E-38	2	Sem1
8.73E-42	0.2706839<br>5	1	0.915	2.12E-37	2	Rps14
2.21E-40	0.2951969<br>5	0.999	0.662	5.35E-36	2	Eif5a
3.91E-31	0.3987720<br>4	0.446	0.231	9.49E-27	2	Xist
6.02E-27	0.4716634<br>8	0.35	0.155	1.46E-22	2	Mmp13
4.48E-26	0.4130832<br>3	0.403	0.215	1.09E-21	2	Rps3a3
7.66E-20	0.4044948<br>1	0.965	0.622	1.86E-15	2	Serpine1
2.15E-207	1.3399547<br>4	1	0.975	5.21E-203	3	Ctsb
5.26E-180	1.3045351<br>8	0.986	0.912	1.27E-175	3	Ctsd
3.39E-169	1.2648431<br>3	0.998	0.963	8.21E-165	3	Psap
2.63E-165	1.8951528	0.944	0.796	6.37E-161	3	Lgals3
1.04E-155	1.5598255<br>2	0.67	0.226	2.53E-151	3	Atp6v0d2

8.26E-155	1.32630987	0.975	0.873	2.00E-150	3	Fabp5
6.17E-154	1.32022663	0.469	0.097	1.50E-149	3	Il7r
7.54E-149	1.31245586	1	0.997	1.83E-144	3	Fth1
4.02E-146	2.39799013	0.907	0.628	9.74E-142	3	Lpl
6.75E-143	1.38552333	0.98	0.832	1.64E-138	3	Ctss
6.42E-131	1.45083975	0.944	0.769	1.56E-126	3	Gpnmb
1.31E-129	2.73844882	0.892	0.647	3.18E-125	3	Mmp12
2.12E-129	1.16702623	0.954	0.743	5.15E-125	3	Creg1
2.50E-121	1.52809167	0.81	0.47	6.06E-117	3	Fabp4
2.57E-112	1.3294734	0.926	0.682	6.23E-108	3	Cd36
5.27E-112	1.10695243	0.988	0.927	1.28E-107	3	Prdx1
4.42E-108	1.46782361	0.676	0.338	1.07E-103	3	Anpep
3.67E-105	1.00683264	0.957	0.772	8.89E-101	3	Mpeg1
3.13E-102	1.59953982	0.778	0.453	7.58E-98	3	Clec7a
3.69E-100	1.31302871	0.792	0.514	8.95E-96	3	Atp6v0a1
4.38E-100	0.81452121	0.329	0.068	1.06E-95	3	Abcg1
7.87E-93	1.0375046	0.872	0.643	1.91E-88	3	Atp6v1b2
7.98E-92	0.94716516	0.48	0.157	1.93E-87	3	Arhgap25
8.05E-92	1.34412892	0.94	0.774	1.95E-87	3	Ctsl
1.27E-89	1.02594626	0.819	0.589	3.07E-85	3	Soat1
7.60E-89	1.28915808	0.821	0.598	1.84E-84	3	Plin2
1.67E-84	0.88399081	0.895	0.69	4.04E-80	3	Atp6v1a

2.78E-84	0.89497828	0.971	0.856	6.73E-80	3	Lgmn
6.29E-83	0.72540995	0.965	0.826	1.53E-78	3	Sdcbp
2.83E-81	0.76564703	0.977	0.825	6.85E-77	3	Grn
5.91E-81	0.92755577	0.852	0.533	1.43E-76	3	Cd53
4.69E-80	0.98497459	0.634	0.292	1.14E-75	3	Ly9
3.22E-79	1.17199886	0.58	0.269	7.82E-75	3	Lat2
7.88E-79	1.06499631	0.776	0.515	1.91E-74	3	Osbpl8
2.03E-77	0.97275753	0.414	0.136	4.91E-73	3	Gpr157
5.23E-76	0.87704165	0.833	0.624	1.27E-71	3	Ctsz
4.53E-75	0.78330382	0.94	0.807	1.10E-70	3	Cstb
6.46E-75	0.94453296	0.715	0.404	1.57E-70	3	Nceh1
6.59E-72	0.92156504	0.736	0.423	1.60E-67	3	Plek
5.03E-71	1.01070496	0.698	0.414	1.22E-66	3	Ptafr
1.07E-70	1.00262517	0.471	0.193	2.60E-66	3	Mfsd12
2.21E-70	1.09653067	0.711	0.427	5.37E-66	3	Rnf128
9.92E-70	0.79501534	0.776	0.431	2.41E-65	3	Cd84
1.30E-69	0.730122	0.969	0.765	3.16E-65	3	Laptm5
1.76E-69	0.77666151	0.949	0.781	4.26E-65	3	Sirpa
4.68E-68	0.76948429	0.926	0.769	1.13E-63	3	H2-D1
5.08E-67	0.69927352	0.994	0.975	1.23E-62	3	Ftl1
2.89E-66	0.8264043	0.761	0.537	7.02E-62	3	Vat1
9.41E-65	0.83055551	0.767	0.483	2.28E-60	3	Itgb2

4.80E-64	0.80542983	0.849	0.628	1.16E-59	3	Iqgap1
9.16E-64	0.81064936	0.812	0.557	2.22E-59	3	Lipa
1.02E-63	0.95625635	0.847	0.66	2.47E-59	3	Sqstm1
1.02E-62	0.81884597	0.37	0.127	2.47E-58	3	Aph1c
1.06E-62	0.83841289	0.728	0.406	2.58E-58	3	C3ar1
1.92E-62	0.8389068	0.789	0.534	4.67E-58	3	Ftl1-ps1
6.11E-62	1.24908501	0.548	0.305	1.48E-57	3	Dnmt3a
1.19E-61	0.75273377	0.85	0.673	2.89E-57	3	Mcl1
2.38E-61	0.79531134	0.852	0.66	5.77E-57	3	Cyba
2.08E-59	0.8738469	0.691	0.432	5.05E-55	3	Tnfrsf1b
1.08E-58	0.72462127	0.356	0.123	2.61E-54	3	Hvcn1
1.64E-58	0.68894891	0.949	0.783	3.97E-54	3	Lcp1
1.17E-57	0.78442468	0.804	0.537	2.84E-53	3	Mafb
2.78E-56	0.72682175	0.829	0.607	6.73E-52	3	Arl6ip1
3.05E-56	0.84937557	0.67	0.452	7.41E-52	3	Dusp3
2.24E-55	0.64935439	0.869	0.725	5.44E-51	3	Atp6v1g1
7.46E-54	0.65362507	0.835	0.642	1.81E-49	3	Ctsa
5.71E-53	0.89060689	0.627	0.415	1.38E-48	3	Pld3
3.15E-52	0.75525	0.333	0.12	7.63E-48	3	Nr1h3
1.09E-51	0.52445032	0.96	0.836	2.63E-47	3	Lamp1
1.72E-50	1.32829681	0.273	0.087	4.17E-46	3	Cd5l
3.95E-50	0.83503338	0.651	0.45	9.57E-46	3	Anxa4
8.08E-50	0.63676221	0.801	0.609	1.96E-45	3	Ctnnb1

1.73E-49	0.89845612	0.787	0.605	4.20E-45	3	Hmox1
1.82E-49	0.71079232	0.781	0.593	4.41E-45	3	Gltp
2.59E-49	0.60015393	0.85	0.683	6.29E-45	3	Atp6v0e
8.43E-49	0.74266632	0.437	0.194	2.04E-44	3	Syngr1
1.79E-48	0.58615762	0.926	0.785	4.33E-44	3	Taldo1
2.64E-48	0.69573852	0.832	0.664	6.40E-44	3	Tnfaip2
2.79E-48	0.51443471	0.924	0.81	6.76E-44	3	Arpc5
4.37E-48	0.62992344	0.417	0.178	1.06E-43	3	Slc15a3
1.53E-46	0.58858542	0.739	0.447	3.70E-42	3	Trem2
1.50E-45	0.87564834	0.517	0.296	3.63E-41	3	Rilpl2
1.67E-45	0.64449885	0.528	0.272	4.04E-41	3	Syk
1.87E-45	0.59002958	0.802	0.615	4.53E-41	3	Snx5
3.52E-45	0.6986748	0.606	0.358	8.54E-41	3	Dnase2a
9.26E-45	0.61770894	0.827	0.546	2.24E-40	3	Cybb
1.29E-44	0.67139128	0.465	0.226	3.13E-40	3	Slc37a2
3.99E-44	0.90733323	0.654	0.479	9.67E-40	3	Gstm1
1.19E-43	1.21983352	0.508	0.323	2.89E-39	3	Lhfpl2
1.43E-43	0.66032076	0.784	0.629	3.47E-39	3	9-Sep
1.81E-43	0.60917196	0.823	0.561	4.38E-39	3	Igf1
3.14E-43	0.66681037	0.64	0.404	7.61E-39	3	Cerk
6.00E-43	0.55960867	0.866	0.709	1.45E-38	3	Ywhah
7.49E-43	0.72453164	0.702	0.454	1.82E-38	3	Trf

8.97E-43	0.5908440 6	0.759	0.504	2.17E-38	3	Cd300a
1.86E-42	0.6355048 8	0.792	0.59	4.50E-38	3	Cebpa
7.56E-42	0.7564429 6	0.59	0.398	1.83E-37	3	Tmem189
1.05E-41	0.9511989 8	0.565	0.351	2.54E-37	3	Hist1h1c
1.74E-41	0.5900562 5	0.778	0.562	4.23E-37	3	Il10rb
2.64E-41	0.6540716 1	0.469	0.243	6.41E-37	3	Plxnc1
3.89E-41	0.6349098	0.381	0.169	9.43E-37	3	Pik3r5
2.14E-40	0.6407139 6	0.836	0.668	5.20E-36	3	Cebpb
2.35E-40	0.5285792 9	0.932	0.814	5.70E-36	3	Sat1
6.83E-40	0.7901625 5	0.532	0.317	1.65E-35	3	Rab7b
7.66E-40	0.5112608 6	0.29	0.105	1.86E-35	3	Slamf7
1.28E-39	0.5199326 8	0.824	0.653	3.09E-35	3	Cst3
1.30E-39	0.7374599 9	0.647	0.469	3.15E-35	3	Por
2.01E-39	0.7454287	0.444	0.219	4.88E-35	3	Abca1
3.09E-39	0.5374370 2	0.42	0.194	7.48E-35	3	Myo1f
6.69E-39	0.7734297 8	0.608	0.432	1.62E-34	3	Atp6v1c1
1.11E-38	0.6109971 1	0.765	0.606	2.69E-34	3	Hexa
1.73E-38	0.6427179 6	0.693	0.499	4.21E-34	3	Abhd12
2.14E-38	0.7851759 6	0.67	0.506	5.19E-34	3	Cd9
2.33E-38	0.5787845 8	0.562	0.32	5.64E-34	3	Cd68
3.35E-38	0.6125955	0.755	0.595	8.12E-34	3	Edem1
7.00E-38	0.7477355 6	0.409	0.208	1.70E-33	3	Bhlhe41
8.18E-38	0.8474595 8	0.478	0.288	1.98E-33	3	Pim1

1.63E-37	0.62432507	0.674	0.491	3.96E-33	3	Plekho2
2.01E-37	0.47919854	0.6	0.34	4.88E-33	3	Rbm47
2.46E-37	0.64002684	0.699	0.507	5.97E-33	3	Crip1
2.50E-37	0.56998835	0.782	0.617	6.06E-33	3	Atp6v0b
2.83E-37	0.44552405	0.957	0.823	6.85E-33	3	H2-K1
4.11E-37	0.66597894	0.494	0.298	9.97E-33	3	Lrp12
1.10E-36	0.59162357	0.724	0.568	2.66E-32	3	Pea15a
2.05E-36	0.59922295	0.644	0.415	4.98E-32	3	Cd300ld
3.00E-36	0.83714619	0.515	0.332	7.27E-32	3	Alcam
3.62E-36	0.45059843	0.903	0.766	8.77E-32	3	Dazap2
4.04E-36	0.54564404	0.753	0.587	9.81E-32	3	Rtn3
4.41E-36	0.56217634	0.838	0.655	1.07E-31	3	Jund
1.04E-35	0.91817709	0.372	0.189	2.51E-31	3	Gadd45a
1.54E-35	0.81440352	0.585	0.404	3.75E-31	3	Hebp1
1.84E-35	0.58407635	0.372	0.175	4.45E-31	3	Gas2l3
3.77E-35	0.75348411	0.426	0.234	9.15E-31	3	Fnip2
5.78E-35	0.57701176	0.569	0.348	1.40E-30	3	Renbp
8.98E-35	0.51426374	0.699	0.467	2.18E-30	3	Smpdl3a
2.47E-34	0.51732296	0.27	0.106	6.00E-30	3	Amz1
2.54E-34	0.483062	0.855	0.682	6.16E-30	3	Tmem50a
4.45E-34	0.51536338	0.728	0.589	1.08E-29	3	Atp6v1e1
7.96E-34	0.51801905	0.502	0.269	1.93E-29	3	Irf8

1.06E-33	0.67210841	0.6	0.438	2.58E-29	3	Dhrs3
2.28E-33	0.50021711	0.421	0.206	5.54E-29	3	Hpgds
4.39E-33	0.52992477	0.616	0.4	1.06E-28	3	Neu1
4.62E-33	0.95645074	0.461	0.279	1.12E-28	3	Gdf15
5.38E-33	0.57410665	0.785	0.637	1.30E-28	3	Id2
5.62E-33	0.54229538	0.403	0.204	1.36E-28	3	Rgs1
7.20E-33	0.46435455	0.773	0.622	1.74E-28	3	Rab7
7.79E-33	0.48469929	0.381	0.18	1.89E-28	3	Tlr13
9.60E-33	0.42829847	0.924	0.777	2.33E-28	3	Npc2
9.87E-33	0.46647011	0.761	0.545	2.39E-28	3	Slc43a2
1.17E-32	0.66720661	0.532	0.328	2.83E-28	3	Adam8
2.64E-32	0.48221216	0.775	0.631	6.39E-28	3	Sh3bgrl3
4.69E-32	0.67166278	0.6	0.45	1.14E-27	3	Mindy1
5.03E-32	0.70273707	0.625	0.481	1.22E-27	3	Tmem65
1.20E-31	0.48935983	0.267	0.11	2.90E-27	3	Gpr137b
1.58E-31	0.64670835	0.437	0.242	3.84E-27	3	Sema4d
4.24E-31	0.71272584	0.542	0.391	1.03E-26	3	Bnip3l
4.77E-31	0.92306955	0.42	0.261	1.16E-26	3	Il11ra1
8.38E-31	0.69767424	0.401	0.23	2.03E-26	3	Psen2
2.04E-30	0.4205798	0.881	0.703	4.94E-26	3	Mrc1
3.25E-30	0.46211313	0.386	0.189	7.89E-26	3	Arhgap9
3.61E-30	0.54520685	0.265	0.112	8.75E-26	3	Atf3

3.74E-30	0.3446327	0.986	0.934	9.08E-26	3	B2m
6.45E-30	0.45474688	0.437	0.233	1.56E-25	3	Pirb
7.32E-30	0.34973961	0.988	0.919	1.78E-25	3	Lyz2
8.72E-30	0.50629001	0.503	0.285	2.11E-25	3	Fcgr4
1.03E-29	0.42003523	0.318	0.141	2.50E-25	3	Coro2a
1.27E-29	0.41238194	0.915	0.772	3.07E-25	3	Arpc4
1.65E-29	0.56004019	0.304	0.147	4.01E-25	3	Gla
1.79E-29	0.71456083	0.492	0.337	4.33E-25	3	Plekhm2
2.08E-29	0.63635821	0.336	0.166	5.05E-25	3	Mertk
2.58E-29	0.44575026	0.733	0.583	6.26E-25	3	Atp6v1f
3.32E-29	0.5716853	0.668	0.522	8.04E-25	3	Ubl3
4.62E-29	0.53074848	0.562	0.365	1.12E-24	3	Lilr4b
5.10E-29	0.40691411	0.906	0.769	1.24E-24	3	Tmbim6
5.35E-29	0.47650242	0.755	0.584	1.30E-24	3	Slc6a6
7.24E-29	0.61755813	0.699	0.527	1.75E-24	3	Aplp2
7.92E-29	0.37358767	0.873	0.668	1.92E-24	3	Tyrobp
1.03E-28	0.43380673	0.816	0.654	2.49E-24	3	Lamp2
1.27E-28	0.43936219	0.829	0.668	3.08E-24	3	Fam120a
1.32E-28	0.57802003	0.582	0.407	3.21E-24	3	Npc1
1.64E-28	0.41537631	0.863	0.763	3.97E-24	3	Akr1a1
2.55E-28	0.66703795	0.583	0.448	6.17E-24	3	Capg
2.61E-28	0.42318671	0.673	0.451	6.34E-24	3	Fermt3

3.48E-28	0.55296131	0.619	0.463	8.45E-24	3	Plekhb2
7.15E-28	0.3461624	0.954	0.854	1.73E-23	3	Itm2b
7.46E-28	0.44083539	0.319	0.152	1.81E-23	3	Arl11
1.14E-27	0.54849253	0.637	0.497	2.75E-23	3	Gns
1.18E-27	0.46884425	0.747	0.592	2.86E-23	3	Degs1
3.06E-27	0.46120442	0.304	0.143	7.41E-23	3	Tbc1d9
4.37E-27	0.51030619	0.582	0.425	1.06E-22	3	Tcirg1
4.38E-27	0.56326851	0.6	0.442	1.06E-22	3	Ctsh
8.32E-27	0.56189928	0.602	0.458	2.02E-22	3	Sgpl1
1.04E-26	0.42638694	0.799	0.644	2.53E-22	3	Tapbp
1.71E-26	0.4230109	0.523	0.322	4.14E-22	3	Cd300c2
2.29E-26	0.49100657	0.503	0.302	5.55E-22	3	Apobec1
3.46E-26	0.46107109	0.543	0.329	8.38E-22	3	Clec4d
3.69E-26	0.3401915	0.949	0.848	8.95E-22	3	Chchd2
4.17E-26	0.49078793	0.673	0.518	1.01E-21	3	Prkcd
5.58E-26	0.42989825	0.485	0.29	1.35E-21	3	Ptk2b
7.40E-26	0.46888279	0.662	0.524	1.79E-21	3	Atp6ap1
1.46E-25	0.7765092	0.623	0.456	3.55E-21	3	Glul
1.12E-24	0.4547974	0.647	0.521	2.71E-20	3	Prr13
1.29E-24	0.52473623	0.644	0.518	3.12E-20	3	Scpep1
1.29E-24	0.45566516	0.671	0.546	3.13E-20	3	Grb2
2.14E-24	0.52728534	0.583	0.44	5.20E-20	3	Hpcal1
2.43E-24	0.41463879	0.88	0.746	5.90E-20	3	Anxa5

4.76E-24	0.50028618	0.721	0.571	1.15E-19	3	Psmd8
1.07E-23	0.44545391	0.671	0.515	2.60E-19	3	Rab8b
1.08E-23	0.36797798	0.569	0.353	2.63E-19	3	Fcgr3
1.14E-23	0.35372988	0.762	0.559	2.75E-19	3	Tpd52
1.33E-23	0.71253056	0.448	0.308	3.23E-19	3	Pmp22
1.42E-23	0.52578285	0.577	0.451	3.45E-19	3	Tpp1
1.75E-23	0.50012165	0.367	0.2	4.24E-19	3	Cd200r1
2.31E-23	0.48155483	0.634	0.505	5.61E-19	3	Myo5a
3.97E-23	0.59372598	0.515	0.384	9.63E-19	3	Evl
5.70E-23	0.26044855	0.977	0.923	1.38E-18	3	Pfn1
6.20E-23	0.36469687	0.795	0.617	1.50E-18	3	Cotl1
6.77E-23	0.61807595	0.449	0.308	1.64E-18	3	Hist1h2bc
7.88E-23	0.45147385	0.494	0.308	1.91E-18	3	Ccr1
9.83E-23	0.4489019	0.651	0.527	2.38E-18	3	Cers2
1.79E-22	0.38738529	0.731	0.597	4.33E-18	3	Reep5
1.88E-22	0.39208205	0.769	0.632	4.55E-18	3	Atp2b1
1.90E-22	0.42325278	0.603	0.477	4.61E-18	3	Zmiz1
1.98E-22	0.47047022	0.625	0.505	4.80E-18	3	Klhl9
2.38E-22	0.35817983	0.372	0.199	5.77E-18	3	Lair1
3.12E-22	0.37445468	0.753	0.591	7.56E-18	3	Ucp2
3.13E-22	0.30018892	0.519	0.315	7.60E-18	3	Hcls1
4.06E-22	0.42039123	0.637	0.521	9.84E-18	3	Ap3s1

4.41E-22	0.54965827	0.593	0.473	1.07E-17	3	Ldlrap1
4.58E-22	0.36297508	0.495	0.299	1.11E-17	3	Ncf2
4.74E-22	0.43952963	0.733	0.626	1.15E-17	3	Vdac2
5.48E-22	0.31908686	0.565	0.348	1.33E-17	3	Inpp5d
1.23E-21	0.31432736	0.389	0.217	2.99E-17	3	Gngt2
1.35E-21	0.63771675	0.623	0.501	3.26E-17	3	Sgk1
1.50E-21	0.39480427	0.418	0.238	3.63E-17	3	Entpd1
2.03E-21	0.3767938	0.377	0.22	4.92E-17	3	Sh3bp2
2.86E-21	0.57815639	0.418	0.283	6.93E-17	3	Cpeb4
3.34E-21	0.25370599	0.965	0.868	8.09E-17	3	BC005537
4.12E-21	0.28650303	0.256	0.119	1.00E-16	3	B430306N03Rik
4.91E-21	0.34339347	0.448	0.266	1.19E-16	3	Ifi207
7.05E-21	0.46761619	0.543	0.395	1.71E-16	3	Arap1
7.80E-21	0.58520435	0.336	0.21	1.89E-16	3	Agap1
1.07E-20	0.46052968	0.573	0.427	2.60E-16	3	Gm2a
1.15E-20	0.31054174	0.429	0.248	2.79E-16	3	Prkcb
1.34E-20	0.28785736	0.997	0.963	3.25E-16	3	Apoe
1.37E-20	0.38080364	0.269	0.132	3.32E-16	3	Selplg
2.01E-20	0.50427854	0.448	0.305	4.86E-16	3	Cln8
2.42E-20	0.36935911	0.434	0.265	5.88E-16	3	mt-Tw
3.63E-20	0.35507764	0.713	0.584	8.80E-16	3	Cat
4.44E-20	0.42680075	0.636	0.528	1.08E-15	3	Myof

4.90E-20	0.79159004	0.463	0.349	1.19E-15	3	Hexb
5.50E-20	0.6203214	0.29	0.165	1.33E-15	3	Sowahc
7.03E-20	0.45815829	0.605	0.491	1.70E-15	3	Vwa5a
9.41E-20	0.58055449	0.412	0.289	2.28E-15	3	Ncoa4
1.30E-19	0.44012995	0.381	0.23	3.16E-15	3	Cln3
1.31E-19	0.44963467	0.59	0.472	3.19E-15	3	Atp6ap2
1.32E-19	0.46549931	0.522	0.388	3.21E-15	3	Uap1l1
1.59E-19	0.75905049	0.414	0.305	3.86E-15	3	Rnh1
1.98E-19	0.33457231	0.864	0.724	4.80E-15	3	Adgre1
2.80E-19	0.58906932	0.508	0.406	6.79E-15	3	Arl8b
3.47E-19	0.34245034	0.739	0.582	8.40E-15	3	Unc93b1
3.56E-19	0.42874855	0.525	0.375	8.63E-15	3	Pip4k2a
5.39E-19	0.50612614	0.46	0.333	1.31E-14	3	Lmbrd1
5.67E-19	0.3593175	0.708	0.604	1.37E-14	3	Bri3
6.38E-19	0.41535216	0.639	0.54	1.55E-14	3	M6pr
7.24E-19	0.33368876	0.877	0.715	1.76E-14	3	Ubc
9.81E-19	0.33941144	0.372	0.214	2.38E-14	3	Nckap1l
1.02E-18	0.45809049	0.323	0.191	2.48E-14	3	Pnpla2
1.13E-18	0.39532898	0.59	0.484	2.75E-14	3	Atp6v0d1
1.23E-18	0.25473055	0.593	0.411	2.98E-14	3	Celf2
1.57E-18	0.3981391	0.617	0.497	3.81E-14	3	Rassf3
1.80E-18	0.40900824	0.63	0.522	4.36E-14	3	Chmp5
2.28E-18	0.42028743	0.565	0.432	5.54E-14	3	Wipf1

2.37E-18	0.3427624	0.764	0.622	5.74E-14	3	Slc40a1
2.86E-18	0.3570338	0.639	0.528	6.94E-14	3	Vps4b
2.94E-18	0.46827152	0.347	0.22	7.12E-14	3	Zfand2a
2.94E-18	0.48014818	0.718	0.601	7.13E-14	3	Dusp1
3.23E-18	0.40879642	0.525	0.384	7.83E-14	3	Tmem106a
3.53E-18	0.40109605	0.657	0.528	8.56E-14	3	Serinc1
3.63E-18	0.47743784	0.265	0.146	8.80E-14	3	Stx3
3.66E-18	0.57228385	0.573	0.469	8.88E-14	3	Clec2d
4.47E-18	0.27585297	0.779	0.674	1.08E-13	3	Adipor1
4.90E-18	0.57741135	0.421	0.318	1.19E-13	3	Plxna1
5.39E-18	0.31754768	0.769	0.61	1.31E-13	3	Itgam
5.47E-18	0.42217571	0.787	0.631	1.33E-13	3	Atf4
5.78E-18	0.28255397	0.884	0.787	1.40E-13	3	Actr2
5.85E-18	0.45217941	0.255	0.133	1.42E-13	3	Cd300lf
6.36E-18	0.50870754	0.426	0.305	1.54E-13	3	Ppt2
6.68E-18	0.32391506	0.417	0.255	1.62E-13	3	Dock10
6.85E-18	0.5603396	0.319	0.199	1.66E-13	3	Flvcr1
6.97E-18	0.33665556	0.735	0.624	1.69E-13	3	Fxyd5
7.87E-18	0.41976646	0.394	0.242	1.91E-13	3	Cd300lb
1.15E-17	0.42202508	0.39	0.25	2.79E-13	3	Ptpre
1.85E-17	0.30445832	0.79	0.698	4.48E-13	3	Snx3
2.38E-17	0.37187565	0.319	0.182	5.76E-13	3	St8sia4
2.69E-17	0.40058003	0.735	0.623	6.51E-13	3	Rhob

2.90E-17	0.4174691 8	0.511	0.389	7.03E-13	3	Acp2
3.75E-17	0.3890807 5	0.565	0.454	9.10E-13	3	Mdfic
5.23E-17	0.3000139 2	0.816	0.717	1.27E-12	3	Pdcd6ip
7.04E-17	0.3620056 7	0.87	0.734	1.71E-12	3	Lrp1
8.52E-17	0.3748896 8	0.505	0.379	2.07E-12	3	Cndp2
1.05E-16	0.2944240 2	0.715	0.548	2.55E-12	3	Cd93
1.33E-16	0.4144633	0.505	0.401	3.23E-12	3	Vps26a
1.71E-16	0.5377205 4	0.27	0.164	4.14E-12	3	Cipc
2.81E-16	0.4037908 9	0.596	0.46	6.82E-12	3	Fos
3.85E-16	0.3228869 4	0.687	0.566	9.33E-12	3	Man2a1
3.91E-16	0.5452897 1	0.369	0.266	9.47E-12	3	Ttyh2
4.08E-16	0.3214359 6	0.752	0.616	9.88E-12	3	Psmd2
4.45E-16	0.3475350 2	0.285	0.158	1.08E-11	3	Hcst
4.59E-16	0.3758160 6	0.586	0.49	1.11E-11	3	Scarb2
4.65E-16	0.4631076 3	0.421	0.313	1.13E-11	3	Mkrn1
5.06E-16	0.2567750 8	0.693	0.594	1.23E-11	3	Cmpk1
5.10E-16	0.3240015 9	0.701	0.619	1.24E-11	3	Mapk3
5.47E-16	0.6291573	0.384	0.287	1.33E-11	3	Samd8
6.32E-16	0.3396885 7	0.37	0.224	1.53E-11	3	Lpxn
7.20E-16	0.4233088	0.559	0.434	1.75E-11	3	Cxcl16
1.03E-15	0.3621176 8	0.66	0.55	2.49E-11	3	Tmem30a
1.11E-15	0.2916652 7	0.273	0.148	2.70E-11	3	Atp8a1
1.35E-15	0.3200065 7	0.514	0.368	3.27E-11	3	Arhgap19

1.83E-15	0.33529661	0.574	0.444	4.43E-11	3	Rnf130
2.21E-15	0.26270467	0.744	0.653	5.37E-11	3	Vamp8
2.42E-15	0.49853045	0.34	0.229	5.86E-11	3	Galns
3.03E-15	0.42818278	0.509	0.386	7.34E-11	3	Pdxk
5.24E-15	0.29941208	0.378	0.232	1.27E-10	3	Il10ra
5.25E-15	0.41397689	0.292	0.169	1.27E-10	3	Cxcr4
6.28E-15	0.42097305	0.52	0.439	1.52E-10	3	Tax1bp1
6.45E-15	0.57588757	0.415	0.321	1.56E-10	3	Srxn1
6.53E-15	0.43868881	0.438	0.322	1.58E-10	3	Mgst1
6.75E-15	0.45300005	0.446	0.349	1.64E-10	3	Gabarapl1
1.06E-14	0.29810079	0.654	0.559	2.57E-10	3	Chmp4b
1.16E-14	0.42103672	0.531	0.43	2.82E-10	3	Plxnd1
1.30E-14	0.44224693	0.333	0.227	3.15E-10	3	Adgre5
1.71E-14	0.47869148	0.478	0.394	4.14E-10	3	Cmas
1.84E-14	0.45626189	0.727	0.624	4.45E-10	3	Gpx1
1.89E-14	0.27032904	0.414	0.266	4.58E-10	3	Cyth4
2.42E-14	0.31513192	0.623	0.522	5.87E-10	3	Idh1
2.62E-14	0.26948233	0.815	0.712	6.35E-10	3	Cap1
2.67E-14	0.45509102	0.421	0.316	6.47E-10	3	Retreg2
2.87E-14	0.3626706	0.563	0.481	6.96E-10	3	Azin1
4.07E-14	0.46092481	0.505	0.419	9.86E-10	3	Gusb
5.28E-14	0.26161732	0.863	0.761	1.28E-09	3	Zeb2

5.99E-14	0.2664999<br>2	0.449	0.3	1.45E-09	3	Ms4a6d
6.25E-14	0.2778425<br>1	0.384	0.246	1.52E-09	3	Slfn2
6.59E-14	0.4975857<br>9	0.265	0.168	1.60E-09	3	Lyst
6.97E-14	0.2938497<br>8	0.824	0.692	1.69E-09	3	C1qc
7.28E-14	0.3415264<br>5	0.269	0.152	1.77E-09	3	Adap1
7.44E-14	0.3454304<br>3	0.549	0.452	1.80E-09	3	Arhgap17
8.38E-14	0.3799092<br>1	0.767	0.631	2.03E-09	3	Cd63
8.88E-14	0.4106384<br>3	0.301	0.189	2.15E-09	3	Pgap1
1.01E-13	0.2823937<br>6	0.606	0.517	2.45E-09	3	Nus1
1.15E-13	0.3662591	0.367	0.248	2.79E-09	3	Bcl2l11
1.15E-13	0.4269257<br>1	0.381	0.274	2.80E-09	3	Dram2
2.03E-13	0.9592682<br>5	0.535	0.478	4.92E-09	3	Tfrc
2.64E-13	0.3154275<br>2	0.353	0.223	6.39E-09	3	Clec12a
3.40E-13	0.4689575<br>4	0.343	0.241	8.25E-09	3	Tpcn2
3.71E-13	0.3325037<br>9	0.409	0.287	9.00E-09	3	Map3k1
3.99E-13	0.3120615<br>4	0.756	0.626	9.68E-09	3	Cd44
4.15E-13	0.4301638<br>9	0.332	0.229	1.01E-08	3	Mtss1
5.52E-13	0.2763527<br>3	0.574	0.434	1.34E-08	3	Fli1
6.58E-13	0.3517721	0.574	0.498	1.59E-08	3	Lamtor1
9.18E-13	0.4690472<br>8	0.289	0.187	2.23E-08	3	Tnfrsf21
9.83E-13	0.5523239<br>1	0.296	0.204	2.38E-08	3	Tusc1
1.16E-12	0.2999603<br>1	0.574	0.5	2.81E-08	3	Rap1a

1.39E-12	0.34772868	0.509	0.411	3.37E-08	3	Prcp
1.41E-12	0.38442802	0.486	0.405	3.43E-08	3	Gna13
1.51E-12	0.40215879	0.309	0.203	3.66E-08	3	Pnpla7
1.55E-12	0.42051172	0.497	0.417	3.75E-08	3	Glmp
2.38E-12	0.46321499	0.505	0.435	5.77E-08	3	Ago2
2.44E-12	0.5358779	0.264	0.171	5.92E-08	3	Slc30a1
2.49E-12	0.43172193	0.418	0.332	6.04E-08	3	Pon2
2.66E-12	0.35452182	0.586	0.514	6.44E-08	3	Lnpep
3.10E-12	0.29009164	0.602	0.499	7.53E-08	3	Nampt
3.71E-12	0.27225047	0.363	0.238	8.98E-08	3	Slc11a1
4.24E-12	0.36168827	0.483	0.398	1.03E-07	3	5031439G07Rik
4.71E-12	0.50450151	0.332	0.243	1.14E-07	3	Clcn7
5.69E-12	0.34067197	0.403	0.293	1.38E-07	3	Gnpda1
6.44E-12	0.31851999	0.52	0.399	1.56E-07	3	Rgs2
6.85E-12	0.41780033	0.258	0.166	1.66E-07	3	Arid3a
7.30E-12	0.2952449	0.404	0.283	1.77E-07	3	Cd48
1.35E-11	0.25645582	0.613	0.535	3.27E-07	3	Vapa
1.40E-11	0.50255787	0.321	0.235	3.38E-07	3	Tnfsf12
1.52E-11	0.42091443	0.35	0.258	3.70E-07	3	Tpra1
1.54E-11	0.35804368	0.509	0.433	3.74E-07	3	Leprot
1.66E-11	0.33165302	0.525	0.453	4.03E-07	3	D17Wsu92e
1.67E-11	0.42942077	0.316	0.224	4.05E-07	3	Trpv2

1.95E-11	0.2817206 8	0.275	0.168	4.73E-07	3	Slc16a10
2.56E-11	0.3857176 5	0.306	0.208	6.20E-07	3	Slc27a1
4.09E-11	0.3117266 3	0.563	0.488	9.92E-07	3	Rad21
4.47E-11	0.2984257 7	0.25	0.155	1.08E-06	3	Dnase1l1
4.59E-11	0.4796932 8	0.341	0.255	1.11E-06	3	Alas1
4.90E-11	0.3536902 4	0.421	0.325	1.19E-06	3	Etv3
6.26E-11	0.2824573 7	0.58	0.503	1.52E-06	3	Sptssa
6.35E-11	0.2893249 6	0.576	0.506	1.54E-06	3	Ptpra
6.64E-11	0.3158512 5	0.494	0.421	1.61E-06	3	Zfand5
7.09E-11	0.4599944 5	0.383	0.306	1.72E-06	3	Mpp1
7.54E-11	0.5247907 8	0.366	0.284	1.83E-06	3	Tsc22d3
8.98E-11	0.4164171 3	0.526	0.457	2.18E-06	3	Stx7
9.04E-11	0.3716322 6	0.409	0.321	2.19E-06	3	P2rx4
9.09E-11	0.4703283 9	0.443	0.371	2.21E-06	3	Igsf8
1.04E-10	0.5582729 5	0.377	0.301	2.53E-06	3	Lmbrd2
1.13E-10	0.2664236 8	0.472	0.349	2.75E-06	3	Vsir
1.24E-10	1.6939981 6	0.312	0.245	3.01E-06	3	Vcam1
1.36E-10	0.3403447 6	0.531	0.472	3.30E-06	3	Msrb1
1.57E-10	0.4150545	0.505	0.451	3.80E-06	3	Smim14
1.78E-10	0.2967155 8	0.327	0.22	4.31E-06	3	Zdhhc14
2.35E-10	0.2812763 7	0.424	0.338	5.69E-06	3	Snx10
2.59E-10	0.4865741 1	0.373	0.301	6.29E-06	3	S100a1

2.97E-10	0.4282408<br>3	0.252	0.17	7.20E-06	3	Gpcpd1
3.14E-10	0.3821558<br>3	0.269	0.182	7.62E-06	3	Daglb
3.37E-10	0.2862197<br>5	0.529	0.462	8.16E-06	3	Larp4b
3.86E-10	0.4623741<br>7	0.336	0.261	9.36E-06	3	Slc36a4
4.36E-10	0.3614673<br>8	0.458	0.395	1.06E-05	3	Plekhj1
4.76E-10	0.2535846	0.69	0.59	1.15E-05	3	9530068E07Ri<br>k
5.16E-10	0.5630167<br>1	0.307	0.237	1.25E-05	3	Bhlhe40
5.17E-10	0.3030406<br>4	0.485	0.419	1.25E-05	3	Trappc2l
5.72E-10	0.3088829<br>8	0.502	0.44	1.39E-05	3	Rragc
9.15E-10	0.3552567	0.306	0.225	2.22E-05	3	Mgat5
9.17E-10	0.2651817<br>1	0.438	0.328	2.22E-05	3	Irf5
1.07E-09	0.3704025<br>5	0.434	0.366	2.59E-05	3	Pank3
1.08E-09	0.4441465<br>7	0.417	0.356	2.61E-05	3	S1pr2
1.09E-09	0.2719700<br>1	0.261	0.165	2.64E-05	3	Vav3
1.19E-09	0.2863932<br>2	0.455	0.384	2.88E-05	3	Atp6v1h
1.30E-09	0.4249991<br>8	0.272	0.196	3.15E-05	3	Prune1
1.68E-09	0.3100000<br>5	0.688	0.604	4.07E-05	3	Klf6
1.73E-09	0.2671085<br>1	0.529	0.458	4.19E-05	3	Rnf13
2.04E-09	0.3653160<br>7	0.457	0.402	4.95E-05	3	Fuca1
2.65E-09	0.2756813<br>5	0.495	0.427	6.43E-05	3	Uhmk1
2.81E-09	0.2567361<br>7	0.614	0.541	6.81E-05	3	Txnrd1
2.88E-09	0.2617114	0.63	0.537	6.99E-05	3	Epb41l2

4.33E-09	0.27556197	0.565	0.493	0.00010501	3	Zfp703
4.79E-09	0.2837524	0.497	0.437	0.0001161	3	Nipbl
7.70E-09	0.3292492	0.471	0.414	0.00018668	3	Cbl
9.48E-09	0.40432499	0.27	0.196	0.00022977	3	Pqlc1
1.01E-08	0.26709897	0.577	0.513	0.00024448	3	Sdf4
1.01E-08	0.55782674	0.253	0.184	0.00024603	3	Plpp3
1.03E-08	0.26869055	0.616	0.514	0.00024895	3	Colgalt1
1.04E-08	0.39995096	0.332	0.239	0.00025169	3	Cadm1
1.06E-08	0.31164482	0.457	0.391	0.00025781	3	Plekho1
1.34E-08	0.2639023	0.739	0.669	0.00032401	3	Btg1
1.42E-08	0.44462944	0.265	0.194	0.00034549	3	Pitpnc1
1.56E-08	0.35890473	0.307	0.235	0.00037945	3	Cdk18
1.58E-08	0.25242942	0.535	0.477	0.00038404	3	Syngr2
1.64E-08	0.25442209	0.574	0.524	0.00039783	3	Copz1
1.65E-08	0.29147871	0.522	0.468	0.00040057	3	Ifngr2
2.07E-08	0.41435697	0.384	0.328	0.00050091	3	Zdhhc18
2.78E-08	0.43719729	0.367	0.313	0.00067344	3	6-Mar
3.90E-08	0.3655244	0.276	0.191	0.00094676	3	Ccl4
3.93E-08	0.2778764	0.295	0.213	0.0009523	3	Sft2d2
3.93E-08	0.33147892	0.363	0.293	0.00095381	3	Vamp4
4.02E-08	0.39442242	0.316	0.246	0.00097415	3	Tspan14
4.93E-08	0.32618712	0.478	0.412	0.00119441	3	Cdc42se2

5.54E-08	0.3235232 4	0.412	0.363	0.0013431 3	3	Ppp1r15b
5.62E-08	0.2768769 5	0.48	0.433	0.0013633 4	3	Cab39
7.26E-08	0.3980542 8	0.415	0.368	0.0017597	3	Fam129b
8.72E-08	0.2963294	0.506	0.453	0.0021140 6	3	Cyb5a
1.02E-07	0.3672591 4	0.306	0.235	0.0024835 1	3	Plekhm1
1.57E-07	0.2809864 9	0.503	0.458	0.0038094 9	3	Grina
2.07E-07	0.3569808 8	0.42	0.365	0.0050223 6	3	Metrnl
2.24E-07	0.3626627 6	0.377	0.318	0.0054229 1	3	Snx1
2.31E-07	0.2580827 9	0.443	0.375	0.0055905 2	3	Kdm7a
3.06E-07	0.2806824 9	0.472	0.419	0.0074208 5	3	Fcho2
3.33E-07	0.2925651 7	0.403	0.332	0.0080715 8	3	Cmtm6
4.07E-07	0.3930669	0.389	0.349	0.0098775 9	3	Polr2a
4.09E-07	0.3378618 3	0.336	0.278	0.0099238 6	3	Acer3
4.10E-07	0.2958732 1	0.52	0.48	0.0099487 8	3	Cpd
5.03E-07	0.2709019 1	0.389	0.331	0.0121903 5	3	Trim25
5.27E-07	0.2974764 9	0.509	0.469	0.0127874 1	3	Xiap
7.05E-07	0.4638858 6	0.279	0.227	0.0171045 6	3	Rgl1
7.85E-07	0.2750560 4	0.423	0.36	0.0190436 1	3	Man2b1
8.78E-07	0.2694945 2	0.463	0.425	0.0212860 8	3	Zbtb7a
1.01E-06	0.2676193 1	0.688	0.605	0.0244851 5	3	C1qa
1.08E-06	0.3673529 4	0.336	0.286	0.0261003 3	3	Pi4k2a

1.41E-06	0.28534276	0.481	0.443	0.03426295	3	Smarcc2
1.47E-06	0.35449995	0.773	0.718	0.0356169	3	Ckb
1.79E-06	0.30727167	0.287	0.225	0.04341331	3	Tgfbr1
2.47E-06	0.284943	0.426	0.377	0.05997784	3	Ggh
2.67E-06	0.28094516	0.4	0.344	0.06474897	3	Specc1
2.89E-06	0.38352664	0.333	0.284	0.07018386	3	Slc29a3
2.99E-06	0.260274	0.508	0.472	0.07239549	3	Ier5
3.00E-06	0.39252648	0.272	0.218	0.07280673	3	Edem2
3.18E-06	0.27987639	0.443	0.41	0.07699765	3	Cdk2ap2
3.82E-06	0.29695207	0.307	0.245	0.09254342	3	Stxbp5
4.48E-06	0.26465331	0.25	0.187	0.10854773	3	Epb41
5.19E-06	0.34216303	0.353	0.308	0.12578134	3	Snx27
5.62E-06	0.29181494	0.267	0.206	0.13635167	3	Ormdl1
5.65E-06	0.31652154	0.349	0.295	0.13707447	3	Hsd17b11
5.70E-06	0.30584101	0.377	0.334	0.13822279	3	Lamtor3
6.69E-06	0.26837992	0.457	0.422	0.16219495	3	Stag2
7.35E-06	0.32290627	0.356	0.306	0.17820103	3	Cpeb2
9.01E-06	0.28378562	0.449	0.419	0.21852815	3	Lypla2
9.70E-06	0.30525696	0.435	0.396	0.2352431	3	Scamp2
1.11E-05	0.34341901	0.427	0.402	0.26915	3	Atp6v1d
1.40E-05	0.29720311	0.316	0.264	0.33931775	3	Klhl24

1.91E-05	0.35250628	0.358	0.32	0.46375642	3	Rap2a
2.02E-05	0.33164777	0.389	0.351	0.49000794	3	Pbx2
2.06E-05	0.29641704	0.377	0.333	0.49885382	3	Mcur1
2.16E-05	0.29603699	0.384	0.352	0.52388578	3	Rtf2
2.19E-05	0.38485221	0.338	0.3	0.53104726	3	1700017B05Rik
4.68E-05	0.33882524	0.284	0.241	1	3	B4galt5
6.09E-05	0.30155269	0.367	0.342	1	3	Mpc1
7.07E-05	0.26544526	0.515	0.484	1	3	Sh3bp5
8.20E-05	0.37330891	0.332	0.297	1	3	Ccndbp1
8.53E-05	0.25907762	0.448	0.425	1	3	Frg1
8.82E-05	0.35196959	0.298	0.262	1	3	Galnt7
8.90E-05	0.25295221	0.375	0.335	1	3	Msmo1
0.00013936	0.32170004	0.284	0.244	1	3	Ifi30
0.00015969	0.3304329	0.36	0.333	1	3	Tmem168
0.00016779	0.33448737	0.282	0.245	1	3	Nabp1
0.00025227	0.26450246	0.304	0.261	1	3	Slc35a5
0.00026576	0.26890568	0.367	0.338	1	3	Elf1
0.00030377	0.26866351	0.29	0.253	1	3	4931406C07Rik
0.0003242	0.29868803	0.262	0.223	1	3	Pla2g15
0.00034938	0.27832966	0.377	0.361	1	3	Wsb2
0.00036933	0.27383203	0.389	0.378	1	3	Tgoln1

0.00043021	0.26478421	0.346	0.312	1	3	Galc
0.00046561	0.27515722	0.349	0.325	1	3	Rnf181
0.000499	0.28369768	0.344	0.324	1	3	Acbd5
0.00053554	0.36298667	0.338	0.315	1	3	Frrs1
0.00056017	0.35055051	0.307	0.287	1	3	Diaph1
0.00057173	0.31152512	0.367	0.351	1	3	Zc3hav1
0.0005902	0.27598252	0.281	0.249	1	3	Bmt2
0.00061875	0.25017084	0.321	0.286	1	3	Ggta1
0.00082286	0.32889917	0.293	0.271	1	3	Socs6
0.00088615	0.2565692	0.387	0.375	1	3	Otulin
0.00104331	0.41210721	0.316	0.283	1	3	Plau
0.00116422	0.35064129	0.323	0.309	1	3	Igf2r
0.00160268	0.25119853	0.278	0.248	1	3	Rftn1
0.00183963	0.25033487	0.324	0.305	1	3	Gtf2a1
0.00252454	0.25773005	0.301	0.28	1	3	Ubap1
0.00292835	0.30037679	0.292	0.274	1	3	Hs6st1
0.00320111	0.26286689	0.444	0.439	1	3	Fcgrt
0.00325888	0.26771128	0.285	0.266	1	3	Gyg
0.00326087	0.35784338	0.267	0.251	1	3	Iqsec1
0.00447287	0.34341358	0.292	0.285	1	3	Plin3
0.00451049	0.27876467	0.35	0.347	1	3	Got1

0.00485623	0.30180893	0.352	0.347	1	3	Irf2bpl
0.00552011	0.26030617	0.265	0.241	1	3	Slc9a3r1
0.00920215	0.2643111	0.269	0.247	1	3	Naga
0.009355	0.30004217	0.259	0.242	1	3	Chmp1b
2.24E-57	1.57118012	0.764	0.714	5.44E-53	4	Mir6236
7.70E-32	0.27905825	0.141	0.449	1.87E-27	4	Myo9b
8.00E-30	0.2617525	0.099	0.362	1.94E-25	4	Zfas1
1.10E-27	0.31443841	0.121	0.389	2.67E-23	4	Nfkb1
1.25E-27	0.28717998	0.115	0.378	3.03E-23	4	Klf3
2.13E-27	0.30071817	0.151	0.442	5.18E-23	4	Erbin
2.78E-27	0.28708638	0.135	0.413	6.75E-23	4	Dnm2
4.32E-27	0.30968756	0.154	0.445	1.05E-22	4	Nxf1
8.34E-27	0.26843934	0.12	0.381	2.02E-22	4	Prpf38b
1.52E-26	0.2899529	0.109	0.359	3.68E-22	4	Kmt2e
2.28E-26	0.33241355	0.109	0.36	5.53E-22	4	Sptan1
3.09E-26	0.25591251	0.101	0.343	7.48E-22	4	Cfap20
4.56E-26	0.31413528	0.121	0.378	1.10E-21	4	Ddx50
5.50E-26	0.26458777	0.166	0.458	1.33E-21	4	Ehmt2
8.78E-26	0.29643301	0.093	0.326	2.13E-21	4	Rbbp6
1.85E-25	0.34829189	0.172	0.469	4.49E-21	4	Vmp1
2.16E-25	0.28104344	0.107	0.349	5.24E-21	4	Zmynd8
3.43E-25	0.28717105	0.137	0.398	8.32E-21	4	Wtap

8.04E-25	0.25932795	0.099	0.333	1.95E-20	4	Smg1
1.28E-24	0.27937663	0.135	0.396	3.10E-20	4	Arglu1
1.68E-24	0.26000273	0.126	0.375	4.08E-20	4	Max
3.78E-24	0.26741625	0.197	0.508	9.17E-20	4	Ywhag
5.95E-24	0.31619025	0.093	0.316	1.44E-19	4	7-Mar
7.98E-24	0.34329645	0.093	0.316	1.93E-19	4	Ifi27
1.15E-23	0.26356377	0.123	0.366	2.79E-19	4	Naa20
1.37E-23	0.26588516	0.061	0.254	3.32E-19	4	Plaur
1.87E-23	0.27197424	0.211	0.521	4.55E-19	4	Atp6ap2
1.89E-23	0.28412603	0.112	0.345	4.59E-19	4	Dazap1
1.94E-23	0.35728081	0.102	0.33	4.72E-19	4	AI314180
2.19E-23	0.5105433	0.998	1	5.31E-19	4	Gm42418
3.08E-23	0.26216404	0.099	0.32	7.46E-19	4	Sh3bp1
3.82E-23	0.31760167	0.127	0.372	9.27E-19	4	Pdlim7
4.71E-23	0.25121553	0.183	0.469	1.14E-18	4	Nsd1
4.77E-23	0.48359092	0.14	0.397	1.16E-18	4	Snhg1
7.67E-23	0.31880085	0.075	0.275	1.86E-18	4	Arfgap1
9.76E-23	0.28352683	0.158	0.424	2.37E-18	4	Jun
1.22E-22	0.34187074	0.186	0.474	2.96E-18	4	Hnrnpdl
1.25E-22	0.27373462	0.245	0.58	3.02E-18	4	Vps4b
1.34E-22	0.32427467	0.107	0.333	3.24E-18	4	Adh5
1.42E-22	0.37942647	0.129	0.369	3.45E-18	4	Eaf1

1.47E-22	0.31470436	0.076	0.276	3.57E-18	4	Mir22hg
2.14E-22	0.32442126	0.089	0.297	5.20E-18	4	Fbxw11
2.17E-22	0.26461847	0.157	0.418	5.26E-18	4	Unc50
2.72E-22	0.33291301	0.084	0.288	6.59E-18	4	Otud4
2.92E-22	0.32956209	0.207	0.511	7.08E-18	4	Ogt
3.17E-22	0.30172138	0.079	0.279	7.68E-18	4	Naxd
3.17E-22	0.29535384	0.106	0.327	7.69E-18	4	Kmt2d
3.27E-22	0.26549827	0.116	0.346	7.93E-18	4	Rabggtb
5.02E-22	0.27585191	0.175	0.45	1.22E-17	4	Git2
5.84E-22	0.32232022	0.078	0.275	1.42E-17	4	Cdip1
9.20E-22	0.37439193	0.09	0.296	2.23E-17	4	Gmppa
1.00E-21	0.32102843	0.116	0.343	2.42E-17	4	Stk38
1.19E-21	0.25090781	0.189	0.467	2.88E-17	4	Uhmk1
1.51E-21	0.3316831	0.138	0.383	3.65E-17	4	Nktr
1.58E-21	0.26499672	0.182	0.453	3.84E-17	4	Skap2
1.63E-21	0.40985086	0.092	0.299	3.95E-17	4	Slc39a13
1.84E-21	0.33392617	0.101	0.314	4.47E-17	4	Usp48
1.90E-21	0.41992729	0.132	0.371	4.60E-17	4	Ccnl2
2.10E-21	0.27408142	0.093	0.298	5.09E-17	4	Rufy3
1.20E-20	0.28548971	0.078	0.267	2.92E-16	4	Bhlhe40
1.35E-20	0.3493574	0.185	0.457	3.27E-16	4	Fcho2
1.54E-20	0.38652663	0.109	0.321	3.73E-16	4	Slc25a37

1.59E-20	0.31752952	0.193	0.47	3.85E-16	4	Abl1
2.06E-20	0.26640641	0.081	0.27	4.99E-16	4	Map4k3
3.80E-20	0.35893029	0.104	0.311	9.21E-16	4	Pan3
3.99E-20	0.25007184	0.071	0.252	9.68E-16	4	Stk24
4.36E-20	0.29347046	0.22	0.521	1.06E-15	4	Gls
4.82E-20	0.4020068	0.098	0.299	1.17E-15	4	Ppp1r12c
5.72E-20	0.25338635	0.073	0.254	1.39E-15	4	Ip6k1
6.97E-20	0.28186103	0.2	0.48	1.69E-15	4	Stx4a
8.78E-20	0.33679654	0.202	0.483	2.13E-15	4	Ube2b
1.43E-19	0.28064013	0.098	0.296	3.47E-15	4	Cog3
1.49E-19	0.34212073	0.078	0.259	3.61E-15	4	Chfr
1.80E-19	0.26238284	0.089	0.278	4.36E-15	4	Crim1
2.41E-19	0.37326805	0.217	0.509	5.83E-15	4	Eif4a2
2.51E-19	0.39424147	0.085	0.272	6.09E-15	4	2310035C23Rik
2.64E-19	0.43136614	0.121	0.335	6.40E-15	4	2010111I01Rik
2.67E-19	0.25143446	0.236	0.539	6.48E-15	4	Hnrnph1
2.88E-19	0.29756041	0.084	0.269	6.99E-15	4	Asxl1
4.21E-19	0.43625313	0.082	0.266	1.02E-14	4	Pnisr
5.64E-19	0.31213802	0.082	0.264	1.37E-14	4	Atxn3
6.53E-19	0.2809899	0.082	0.264	1.58E-14	4	Mknk2
6.93E-19	0.28962847	0.095	0.285	1.68E-14	4	Rps27rt
7.39E-19	0.41373546	0.151	0.383	1.79E-14	4	Snhg9

7.74E-19	0.27058411	0.231	0.53	1.88E-14	4	Srrm2
7.97E-19	0.33001047	0.207	0.483	1.93E-14	4	Srsf6
9.57E-19	0.37868567	0.22	0.51	2.32E-14	4	Atxn2l
1.05E-18	0.42234479	0.124	0.337	2.53E-14	4	Sbno2
1.07E-18	0.32370135	0.255	0.567	2.59E-14	4	Wsb1
1.62E-18	0.27671637	0.14	0.359	3.93E-14	4	Dnmt3a
2.59E-18	0.51699958	0.141	0.366	6.28E-14	4	Tmem259
2.90E-18	0.29976158	0.124	0.332	7.02E-14	4	Gtf2a1
3.12E-18	0.27588288	0.09	0.272	7.57E-14	4	Nfya
3.68E-18	0.33570014	0.158	0.393	8.92E-14	4	Ypel3
5.72E-18	0.28996992	0.134	0.345	1.39E-13	4	Bach1
6.87E-18	0.37352974	0.22	0.495	1.67E-13	4	Grina
1.03E-17	0.28030564	0.092	0.271	2.51E-13	4	Clk3
1.14E-17	0.36572867	0.225	0.516	2.78E-13	4	Nisch
1.30E-17	0.39098045	0.18	0.429	3.15E-13	4	Safb
2.34E-17	0.26216413	0.126	0.324	5.66E-13	4	Stxbp2
3.38E-17	0.27322577	0.284	0.603	8.20E-13	4	Adam15
3.64E-17	0.48188711	0.095	0.275	8.82E-13	4	Fblim1
3.93E-17	0.35466307	0.138	0.351	9.53E-13	4	Bmp1
4.22E-17	0.27372763	0.96	0.968	1.02E-12	4	Apoe
4.32E-17	0.34042891	0.098	0.279	1.05E-12	4	Itga5

4.68E-17	0.26213457	0.2	0.451	1.13E-12	4	Blvrb
5.08E-17	0.31761708	0.224	0.495	1.23E-12	4	Arhgap17
1.81E-16	0.50995518	0.148	0.363	4.40E-12	4	Ccnl1
2.74E-16	0.27770332	0.109	0.29	6.64E-12	4	Tpra1
2.85E-16	0.38052669	0.12	0.31	6.91E-12	4	Rasa4
3.31E-16	0.26579586	0.208	0.46	8.02E-12	4	Cerk
3.74E-16	0.28326716	0.134	0.331	9.07E-12	4	Pkn1
6.31E-16	0.42241656	0.138	0.34	1.53E-11	4	Kansl1
2.58E-15	0.30089719	0.278	0.57	6.27E-11	4	Prkcd
1.71E-14	0.37059895	0.106	0.272	4.16E-10	4	Tpcn2
1.84E-14	0.36114532	0.298	0.608	4.46E-10	4	Snx2
2.76E-14	0.61646805	0.132	0.319	6.70E-10	4	Dmpk
1.52E-13	0.35490946	0.239	0.498	3.69E-09	4	Slc38a2
1.95E-13	0.27151093	0.172	0.371	4.73E-09	4	Cd68
3.73E-13	0.78529524	0.14	0.325	9.04E-09	4	mt-Atp6
4.77E-13	0.43979637	0.124	0.293	1.16E-08	4	Gclc
6.89E-13	0.3228019	0.104	0.257	1.67E-08	4	Gmip
9.65E-13	0.42679149	0.248	0.51	2.34E-08	4	Clk1
1.26E-12	0.63951257	0.118	0.279	3.06E-08	4	CT010467.1
1.47E-12	0.39854767	0.102	0.251	3.55E-08	4	Cyth1
1.61E-12	0.52750147	0.23	0.471	3.91E-08	4	Tcirg1
2.83E-12	0.46511414	0.165	0.353	6.85E-08	4	BC037034

3.29E-12	0.27281276	0.138	0.307	7.98E-08	4	Plau
4.49E-12	0.25806783	0.262	0.504	1.09E-07	4	Glul
5.27E-12	0.25967195	0.124	0.28	1.28E-07	4	Bcl2l11
1.56E-11	0.32730638	0.149	0.316	3.79E-07	4	Cd48
1.80E-11	0.63216434	0.155	0.336	4.37E-07	4	mt-Co3
2.24E-11	0.26057134	0.247	0.473	5.44E-07	4	Ptafr
2.26E-11	0.30775596	0.208	0.408	5.49E-07	4	Arhgap19
5.57E-11	0.26745762	0.356	0.673	1.35E-06	4	Atp6v0b
1.13E-10	0.25292709	0.362	0.661	2.73E-06	4	Rcbtb2
1.35E-10	0.34183238	0.272	0.514	3.28E-06	4	Zfp36
2.12E-10	0.41700173	0.773	0.899	5.14E-06	4	Fabp5
7.68E-10	0.27101575	0.273	0.496	1.86E-05	4	Stab1
1.51E-09	0.31865429	0.233	0.437	3.66E-05	4	Rgs2
2.01E-09	0.35802081	0.208	0.395	4.86E-05	4	Renbp
2.55E-09	0.45008874	0.118	0.25	6.18E-05	4	Lpar6
4.59E-09	0.26290188	0.362	0.643	0.0001113	4	Ucp2
9.01E-09	0.2905213	0.359	0.66	0.00021855	4	Picalm
1.52E-08	0.70735195	0.199	0.382	0.00036942	4	AC149090.1
1.60E-08	0.31145929	0.168	0.319	0.00038883	4	Syk
1.78E-08	0.37371542	0.14	0.272	0.00043127	4	Pirb
4.54E-08	0.36476701	0.214	0.391	0.00110183	4	Rbm47

2.17E-07	0.59110218	0.679	0.848	0.00525536	4	Sat1
5.37E-07	0.37666116	0.405	0.705	0.01301082	4	Atp6v1b2
3.02E-06	0.51805736	0.236	0.408	0.07311429	4	Lilr4b
5.59E-06	0.50166784	0.475	0.684	0.13554973	4	Lpl
6.79E-06	0.47182832	0.317	0.535	0.16459513	4	Fabp4
2.66E-05	0.36837102	0.39	0.644	0.64531312	4	Dusp1
2.82E-05	1.69430809	0.172	0.301	0.68414569	4	mt-Atp8
6.34E-05	0.68422262	0.32	0.541	1	4	Sgk1
0.00010295	2.99012288	0.627	0.769	1	4	mt-Rnr1
0.00031154	0.44323994	0.169	0.269	1	4	Xist
0.00114104	2.74886499	0.374	0.614	1	4	Gm26917
0.0015288	0.77182155	0.331	0.548	1	4	Neat1
0.00170292	0.39649395	0.432	0.683	1	4	Id2
0.00192337	0.7213884	0.564	0.769	1	4	Rsrp1
0.00259345	0.45601001	0.427	0.703	1	4	Snrnp70
0.00424931	0.32711335	0.43	0.654	1	4	Itgam
0.00634909	0.2888275	0.502	0.772	1	4	Adgre1
0.00807369	0.34652658	0.475	0.763	1	4	Srsf5
9.10E-171	2.24312402	0.993	0.773	2.21E-166	5	S100a6
1.43E-163	3.40656652	0.821	0.387	3.47E-159	5	Palld
1.04E-154	2.83428972	0.854	0.403	2.52E-150	5	Tagln

3.36E-150	2.19783905	0.91	0.538	8.15E-146	5	Tpm1
9.46E-143	2.35961606	0.856	0.437	2.29E-138	5	Cald1
1.67E-123	1.32903498	0.991	0.929	4.05E-119	5	Rpl41
3.72E-119	1.85466217	0.905	0.767	9.02E-115	5	Npm1
6.89E-119	2.04034963	0.833	0.555	1.67E-114	5	Tpm4
1.37E-116	2.08426438	0.796	0.498	3.31E-112	5	S100a11
9.71E-116	1.07756129	1	0.996	2.35E-111	5	Actb
3.61E-112	1.11836199	0.995	0.973	8.76E-108	5	Actg1
2.81E-108	1.03694151	0.974	0.956	6.81E-104	5	Eef1a1
2.54E-105	1.13224445	0.979	0.923	6.15E-101	5	Rpl37a
8.66E-104	1.64706492	0.907	0.776	2.10E-99	5	Vim
1.51E-102	1.18685734	0.951	0.929	3.67E-98	5	Rps26
3.81E-102	2.03135905	0.826	0.62	9.23E-98	5	Dstn
4.71E-98	2.38888626	0.828	0.588	1.14E-93	5	Acta2
2.02E-96	1.21065006	0.933	0.895	4.91E-92	5	Myl6
5.07E-96	1.6890847	0.84	0.586	1.23E-91	5	Tmsb10
7.92E-93	1.50861975	0.877	0.791	1.92E-88	5	Lgals1
8.03E-91	1.2534447	0.942	0.873	1.95E-86	5	Rps12
3.99E-90	1.82299585	0.835	0.693	9.68E-86	5	Ncl
7.31E-88	1.25504382	0.926	0.851	1.77E-83	5	Rpl38
1.99E-86	1.26069282	0.926	0.888	4.84E-82	5	Rpsa
5.34E-81	1.22882493	0.891	0.856	1.30E-76	5	Ppia
1.49E-78	1.8192076	0.701	0.399	3.60E-74	5	Nedd4

1.90E-77	1.43171497	0.821	0.721	4.61E-73	5	Gnas
1.44E-75	1.40876589	0.856	0.718	3.50E-71	5	Anxa2
2.96E-73	2.12171314	0.712	0.382	7.17E-69	5	Ccnd2
2.43E-70	1.00491069	0.937	0.91	5.90E-66	5	Rps8
1.56E-68	1.08755339	0.919	0.906	3.79E-64	5	Hsp90ab1
4.59E-68	0.8974147	0.93	0.912	1.11E-63	5	Rps24
1.74E-67	1.00892459	0.879	0.87	4.21E-63	5	Rps15
3.37E-66	1.22471553	0.896	0.705	8.17E-62	5	Mir6236
5.16E-66	1.71134299	0.636	0.346	1.25E-61	5	Cavin1
5.97E-66	1.51891072	0.805	0.706	1.45E-61	5	Ldha
1.58E-65	0.86106481	0.951	0.929	3.84E-61	5	Rplp2
1.52E-64	1.49301588	0.724	0.518	3.69E-60	5	Eef1g
3.48E-64	0.94592779	0.9	0.878	8.45E-60	5	Rpl23
3.83E-62	1.36536064	0.768	0.592	9.29E-58	5	Rpl6
5.31E-58	1.905809	0.589	0.338	1.29E-53	5	Tpm2
9.18E-58	1.31003657	0.77	0.523	2.23E-53	5	Rpl35
1.38E-56	1.14270332	0.796	0.736	3.35E-52	5	Rps3
2.31E-56	1.29786093	0.742	0.609	5.61E-52	5	Rpl4
1.74E-53	1.29717206	0.74	0.535	4.23E-49	5	Rps2
4.49E-52	0.98652275	0.852	0.772	1.09E-47	5	Rpl35a
7.88E-52	1.21033579	0.761	0.692	1.91E-47	5	Ywhae
7.65E-51	1.31673927	0.766	0.7	1.86E-46	5	Mif

1.85E-50	1.68064685	0.65	0.507	4.48E-46	5	Fkbp1a
3.03E-49	0.62524622	0.974	0.97	7.35E-45	5	Rplp1
1.15E-48	1.76598691	0.538	0.304	2.78E-44	5	Dpysl3
9.92E-48	0.88816257	0.861	0.83	2.40E-43	5	Rps27a
9.34E-47	1.10700541	0.78	0.767	2.26E-42	5	Myl12a
1.19E-46	1.05561319	0.814	0.783	2.89E-42	5	Rps3a1
1.73E-46	0.87462249	0.842	0.828	4.20E-42	5	Ybx1
3.82E-46	1.55763082	0.659	0.53	9.26E-42	5	Hsp90aa1
4.15E-46	1.30621358	0.689	0.536	1.01E-41	5	Rpl17
7.78E-46	0.91670188	0.882	0.833	1.89E-41	5	Rps28
1.71E-44	0.90324112	0.84	0.836	4.14E-40	5	Rps4x
2.24E-44	0.91605726	0.819	0.842	5.42E-40	5	Rpl8
3.27E-44	0.75624488	0.882	0.902	7.92E-40	5	Cfl1
6.95E-44	0.94584914	0.845	0.801	1.69E-39	5	Rps20
8.68E-44	1.2953008	0.712	0.641	2.11E-39	5	Txn1
3.76E-43	1.02426356	0.775	0.569	9.11E-39	5	Gm10076
4.96E-41	2.15572062	0.594	0.469	1.20E-36	5	Cyb5r3
9.85E-41	1.50402435	0.622	0.479	2.39E-36	5	Gapdh
1.09E-40	0.65341062	0.91	0.929	2.63E-36	5	Rack1
3.90E-40	0.99814936	0.787	0.764	9.46E-36	5	Rpl22
7.29E-40	1.23271355	0.705	0.629	1.77E-35	5	Eif2s2
2.46E-39	1.25670909	0.675	0.557	5.96E-35	5	Rpl9

3.05E-39	1.34387291	0.592	0.409	7.39E-35	5	Cnn2
5.74E-39	1.45328542	0.568	0.388	1.39E-34	5	11-Sep
1.06E-38	1.85288857	0.404	0.198	2.56E-34	5	Hspb1
1.08E-38	1.09651287	0.729	0.661	2.62E-34	5	Rpl13
2.42E-38	1.39286552	0.754	0.74	5.87E-34	5	Pgk1
5.85E-38	1.02101009	0.761	0.69	1.42E-33	5	Rps27l
2.07E-37	1.02434459	0.735	0.725	5.02E-33	5	Rpl23a
5.13E-37	0.54216368	0.979	0.97	1.25E-32	5	Tpt1
6.90E-37	1.0367897	0.747	0.699	1.67E-32	5	Serbp1
1.04E-36	0.79066493	0.826	0.847	2.53E-32	5	Rps5
4.03E-36	1.10028449	0.668	0.547	9.77E-32	5	Rpl36a
2.87E-35	0.97685831	0.782	0.71	6.95E-31	5	Rpl37
3.39E-35	1.12130962	0.715	0.662	8.21E-31	5	Pkm
8.20E-35	0.96271359	0.71	0.682	1.99E-30	5	Btf3
2.47E-34	1.20736995	0.633	0.488	5.98E-30	5	Rps13
3.24E-34	0.83899082	0.826	0.851	7.87E-30	5	Rpl39
3.73E-34	0.63307001	0.912	0.903	9.04E-30	5	Rps21
4.15E-34	1.61941148	0.566	0.43	1.01E-29	5	Anxa1
4.35E-34	0.93141751	0.754	0.794	1.05E-29	5	Calm1
3.08E-33	1.3544108	0.675	0.628	7.48E-29	5	Tagln2
1.07E-32	0.55872693	0.937	0.93	2.60E-28	5	Rps29
2.99E-32	0.91036633	0.768	0.655	7.24E-28	5	Rps27

8.69E-32	0.8563904 1	0.766	0.765	2.11E-27	5	Rplp0
1.62E-31	1.2753178 9	0.68	0.67	3.92E-27	5	Msn
4.52E-31	0.9618916 5	0.729	0.659	1.10E-26	5	Rps23
3.82E-30	1.2080144 6	0.682	0.679	9.26E-26	5	Nap1l1
5.34E-30	0.7729554 1	0.775	0.791	1.30E-25	5	Eif1
7.39E-30	1.4630356	0.552	0.448	1.79E-25	5	Abracl
4.18E-29	0.8401174 6	0.775	0.788	1.01E-24	5	Eef1b2
4.58E-28	1.3981178	0.65	0.641	1.11E-23	5	Anp32b
5.44E-28	1.2200778 2	0.596	0.534	1.32E-23	5	Pebp1
6.18E-28	1.0467482 5	0.661	0.624	1.50E-23	5	Rpl3
5.31E-27	1.4471942 1	0.506	0.366	1.29E-22	5	Csrp1
1.46E-26	1.1811217 1	0.603	0.545	3.54E-22	5	Cct6a
3.24E-26	0.7618607 3	0.749	0.786	7.87E-22	5	Rbm3
4.96E-26	0.8829445 5	0.691	0.705	1.20E-21	5	Eif5a
1.20E-25	1.0254059 5	0.633	0.549	2.92E-21	5	Rps15a
2.09E-25	0.8497577 8	0.735	0.726	5.06E-21	5	Rps16
4.78E-25	0.9845085 4	0.675	0.647	1.16E-20	5	Rpl7a
9.15E-25	1.2752204 3	0.494	0.371	2.22E-20	5	Ybx3
1.47E-23	1.0187602 5	0.619	0.581	3.56E-19	5	Rpl18
2.85E-23	1.0080405 6	0.622	0.604	6.91E-19	5	Rpl7
6.61E-23	1.0920781 3	0.599	0.501	1.60E-18	5	Rpl12
9.28E-23	0.9567444 1	0.633	0.597	2.25E-18	5	Rpl11

1.07E-22	0.99217237	0.647	0.636	2.59E-18	5	Nme1
1.08E-22	0.96467784	0.626	0.588	2.62E-18	5	Rps17
1.48E-22	0.90625223	0.717	0.673	3.60E-18	5	Tubb5
3.02E-22	1.14902028	0.578	0.523	7.33E-18	5	Rpl5
3.65E-22	1.09456964	0.619	0.602	8.85E-18	5	Pgam1
4.78E-22	0.6048892	0.84	0.882	1.16E-17	5	Rpl19
1.09E-21	1.20273833	0.508	0.402	2.64E-17	5	Rhoc
1.56E-21	1.09720925	0.573	0.487	3.78E-17	5	Lmna
4.07E-21	1.6307973	0.378	0.242	9.87E-17	5	Myl9
4.53E-21	0.57491388	0.919	0.935	1.10E-16	5	Ptma
1.36E-20	1.07823015	0.587	0.55	3.31E-16	5	Hspe1
9.51E-20	1.38036012	0.503	0.435	2.31E-15	5	Ccng1
1.79E-19	0.73523274	0.684	0.69	4.34E-15	5	Rpl14
1.66E-18	0.91790305	0.645	0.633	4.03E-14	5	Myh9
2.27E-18	1.29559549	0.39	0.267	5.51E-14	5	Eno1
7.22E-18	0.61319113	0.756	0.811	1.75E-13	5	Rps10
2.64E-17	0.75925618	0.668	0.671	6.39E-13	5	Rps11
2.73E-17	0.9261637	0.596	0.575	6.62E-13	5	Ran
4.14E-17	1.02528943	0.647	0.705	1.00E-12	5	Eif3a
4.35E-17	1.03812005	0.527	0.456	1.06E-12	5	Cox7c
6.15E-17	0.8379008	0.615	0.553	1.49E-12	5	Rps18
1.33E-16	0.99650903	0.599	0.583	3.23E-12	5	Anxa3
2.09E-16	0.66781567	0.719	0.786	5.07E-12	5	Sem1

3.20E-16	0.6460385	0.742	0.798	7.75E-12	5	Eef2
4.38E-16	0.98574563	0.568	0.542	1.06E-11	5	Set
1.08E-15	1.11985686	0.529	0.494	2.62E-11	5	Nars
1.98E-15	1.07658667	0.552	0.522	4.81E-11	5	Clic4
3.52E-15	1.06857186	0.606	0.51	8.53E-11	5	Ccnd1
4.32E-15	0.82109807	0.659	0.721	1.05E-10	5	Gnb1
4.53E-15	0.26133129	0.107	0.32	1.10E-10	5	Golga4
4.99E-15	0.94221081	0.587	0.617	1.21E-10	5	Cox6c
5.20E-15	1.18810064	0.487	0.392	1.26E-10	5	S100a4
6.98E-15	0.9696153	0.599	0.602	1.69E-10	5	Rpl30
8.04E-15	0.69467594	0.659	0.695	1.95E-10	5	Atp5g3
1.08E-14	1.09759987	0.462	0.4	2.63E-10	5	S100a10
6.39E-14	0.61046258	0.715	0.799	1.55E-09	5	Eif4g2
1.40E-13	0.93798281	0.51	0.456	3.38E-09	5	Rpl21
1.45E-13	0.7465333	0.626	0.685	3.52E-09	5	Naca
1.64E-13	0.48865906	0.891	0.932	3.98E-09	5	Pabpc1
2.06E-13	1.16165169	0.499	0.494	5.00E-09	5	Eloc
2.07E-13	0.75001617	0.613	0.597	5.01E-09	5	Rps19
3.19E-13	0.96346323	0.545	0.545	7.73E-09	5	Rps6
4.12E-13	0.97125454	0.541	0.53	9.98E-09	5	Hmgn1
4.34E-13	1.50995828	0.278	0.182	1.05E-08	5	Map1b
4.45E-13	1.16354738	0.515	0.503	1.08E-08	5	Hspd1
4.72E-13	0.71588856	0.733	0.827	1.14E-08	5	Hnrnpa2b1

5.74E-13	0.7976057	0.617	0.65	1.39E-08	5	Rpl26
6.76E-13	0.73326235	0.647	0.683	1.64E-08	5	Rpl18a
9.36E-13	0.83272287	0.585	0.581	2.27E-08	5	Rpl34
1.47E-12	0.67208526	0.645	0.657	3.56E-08	5	Rpl28
2.06E-12	1.00970152	0.564	0.584	4.99E-08	5	Tpi1
2.15E-12	0.49185938	0.747	0.823	5.21E-08	5	Fau
2.70E-12	1.18218933	0.397	0.317	6.56E-08	5	Cnn3
3.89E-12	0.77297658	0.643	0.726	9.44E-08	5	Mtpn
4.79E-12	0.93781919	0.531	0.521	1.16E-07	5	Uqcrq
6.44E-12	0.82711738	0.541	0.501	1.56E-07	5	Rpl10
7.62E-12	0.2632307	0.104	0.279	1.85E-07	5	Fxr2
8.35E-12	0.25076491	0.121	0.304	2.02E-07	5	Gabpa
1.15E-11	0.35353108	0.896	0.928	2.79E-07	5	Rps14
2.23E-11	0.45203999	0.773	0.848	5.40E-07	5	Rpl32
2.34E-11	1.00336296	0.413	0.34	5.66E-07	5	Tuba1a
2.93E-11	1.03872814	0.503	0.495	7.10E-07	5	Rpl31
2.93E-11	0.25798705	0.137	0.324	7.11E-07	5	Bms1
3.59E-11	0.32129273	0.128	0.313	8.69E-07	5	Apc
4.57E-11	0.26322051	0.116	0.293	1.11E-06	5	Kpna3
4.94E-11	0.26276825	0.137	0.324	1.20E-06	5	Srp54a
7.12E-11	0.91701539	0.503	0.495	1.73E-06	5	Sec61b
7.53E-11	1.10134831	0.441	0.414	1.82E-06	5	Slc25a4

8.22E-11	0.8635162<br>6	0.568	0.616	1.99E-06	5	AC121965.1
8.68E-11	1.2087073<br>3	0.353	0.279	2.10E-06	5	Ak1
8.70E-11	0.2581884<br>9	0.139	0.324	2.11E-06	5	Commd1
1.26E-10	0.8981305<br>3	0.587	0.63	3.04E-06	5	Esd
1.48E-10	0.5763848<br>1	0.666	0.777	3.58E-06	5	Ywhaz
1.50E-10	0.2696130<br>7	0.146	0.335	3.65E-06	5	Otud6b
1.82E-10	0.2897694<br>3	0.128	0.304	4.41E-06	5	Rsrc1
2.00E-10	0.2515749<br>9	0.13	0.308	4.84E-06	5	Gatad2a
2.12E-10	0.2622324<br>8	0.107	0.269	5.13E-06	5	Phf5a
2.40E-10	1.7221269<br>3	0.325	0.252	5.83E-06	5	Samd4
2.79E-10	1.3657224	0.32	0.247	6.78E-06	5	Parva
3.01E-10	1.1308319<br>6	0.376	0.303	7.29E-06	5	Fscn1
3.48E-10	0.7593917<br>4	0.573	0.603	8.43E-06	5	Dynll1
4.28E-10	0.2643978<br>3	0.125	0.298	1.04E-05	5	Glrx2
7.30E-10	0.3938824	0.114	0.277	1.77E-05	5	Msl3
7.87E-10	0.9423172<br>1	0.483	0.471	1.91E-05	5	Tcp1
8.35E-10	0.6897177<br>9	0.624	0.688	2.03E-05	5	Ptms
8.76E-10	1.1113135<br>3	0.397	0.338	2.12E-05	5	Ppfibp1
9.43E-10	0.3013126	0.104	0.259	2.29E-05	5	Sucla2
9.78E-10	0.6552687	0.626	0.694	2.37E-05	5	Rps25
1.12E-09	0.6752309<br>2	0.613	0.694	2.72E-05	5	Serpinb6a
1.17E-09	1.0975338<br>2	0.452	0.441	2.85E-05	5	Calm3
1.30E-09	0.2667996<br>3	0.104	0.256	3.15E-05	5	Lamtor5

1.43E-09	0.27537921	0.121	0.283	3.46E-05	5	Arl2bp
1.77E-09	1.178359	0.422	0.395	4.30E-05	5	Usmg5
2.00E-09	0.91009124	0.49	0.485	4.85E-05	5	Hdlbp
2.28E-09	0.25539377	0.209	0.417	5.53E-05	5	Exoc5
2.31E-09	0.4452617	0.146	0.325	5.59E-05	5	Zbtb20
2.33E-09	0.30173024	0.181	0.378	5.65E-05	5	Ralbp1
2.65E-09	1.10991175	0.429	0.383	6.42E-05	5	Lpp
2.71E-09	0.28565938	0.139	0.31	6.58E-05	5	Epb41l1
2.86E-09	1.25473366	0.295	0.217	6.94E-05	5	Rpl39l
3.46E-09	0.26988939	0.153	0.331	8.38E-05	5	Cop1
3.52E-09	0.33729497	0.128	0.291	8.53E-05	5	Usp47
3.63E-09	0.39126478	0.148	0.327	8.81E-05	5	Gxylt1
4.09E-09	1.03818936	0.529	0.574	9.92E-05	5	Kif5b
4.21E-09	0.25452145	0.151	0.323	0.00010202	5	Rnf20
4.55E-09	0.26018183	0.234	0.458	0.00011036	5	Jpt1
5.56E-09	0.27160431	0.118	0.271	0.00013472	5	Coa3
5.61E-09	0.8005821	0.557	0.614	0.00013594	5	Hdgf
5.83E-09	0.26482355	0.223	0.437	0.00014138	5	Snrpb2
5.97E-09	1.08791367	0.36	0.292	0.00014476	5	Enah
6.87E-09	0.3903262	0.125	0.286	0.00016664	5	Rps6ka4
6.96E-09	0.3068117	0.209	0.417	0.00016884	5	Hnrnpul2
7.26E-09	0.27183717	0.179	0.365	0.00017608	5	Csnk2a1

7.40E-09	0.2607502<br>5	0.202	0.405	0.0001795<br>3	5	Chmp3
7.91E-09	0.3104505<br>3	0.146	0.314	0.0001918<br>2	5	Usp14
7.97E-09	0.3383474<br>7	0.142	0.308	0.0001932	5	Arhgap5
8.33E-09	0.2765459<br>2	0.109	0.253	0.0002020<br>7	5	Aen
8.36E-09	0.3521386<br>8	0.144	0.313	0.0002027<br>3	5	Vbp1
8.38E-09	0.2820794<br>7	0.153	0.326	0.0002031<br>9	5	Rtcb
8.67E-09	0.3252962<br>7	0.155	0.334	0.0002102<br>2	5	Hdac3
9.99E-09	0.3827664<br>6	0.195	0.397	0.0002423<br>1	5	Ogdh
1.15E-08	0.2801254<br>9	0.118	0.268	0.0002795<br>2	5	Rps6kb1
1.23E-08	0.3046907<br>3	0.148	0.318	0.0002976<br>5	5	Cbx5
1.27E-08	0.2672975<br>6	0.137	0.299	0.0003077	5	Map3k7
1.49E-08	1.1432423<br>9	0.462	0.477	0.0003616	5	7-Sep
1.50E-08	0.3875445<br>3	0.116	0.264	0.0003625<br>8	5	Gart
1.66E-08	0.3373849<br>3	0.148	0.315	0.0004021<br>9	5	Flii
1.81E-08	0.3533150<br>1	0.135	0.294	0.0004389<br>8	5	Gfpt1
1.82E-08	0.5717119<br>6	0.55	0.549	0.0004405<br>8	5	Lars2
1.84E-08	0.2887314<br>5	0.16	0.332	0.0004472<br>8	5	Sec22b
1.96E-08	0.2585210<br>3	0.137	0.293	0.0004744<br>8	5	Smim11
1.97E-08	0.3281167<br>9	0.169	0.349	0.0004779<br>1	5	Ccz1
2.16E-08	0.2632746<br>6	0.209	0.406	0.0005244<br>1	5	Dhx15
2.49E-08	0.3497435	0.13	0.285	0.0006042<br>6	5	Trove2

2.51E-08	0.27213623	0.109	0.252	0.00060764	5	Zfr
2.60E-08	0.30507392	0.146	0.308	0.00063005	5	Srrt
2.77E-08	0.56792769	0.148	0.317	0.00067163	5	Zc3h13
2.96E-08	0.32899087	0.172	0.347	0.00071737	5	Zmynd11
2.99E-08	0.34431419	0.121	0.269	0.0007262	5	Lrrfip2
3.11E-08	1.33240509	0.285	0.221	0.00075458	5	Ddah1
3.30E-08	0.27599051	0.125	0.274	0.00080068	5	Dpy30
3.52E-08	0.30973505	0.204	0.4	0.00085408	5	Khsrp
3.60E-08	1.00574782	0.464	0.459	0.00087368	5	Hmgb1
3.86E-08	0.32324061	0.169	0.338	0.00093556	5	Rb1cc1
3.87E-08	0.93995775	0.49	0.513	0.00093864	5	Eif3e
4.04E-08	0.27933304	0.169	0.344	0.00097917	5	Lsm4
4.42E-08	0.3296629	0.139	0.296	0.00107073	5	Cdk16
4.70E-08	0.25449399	0.162	0.331	0.00113991	5	Ccdc6
5.16E-08	0.37062987	0.13	0.28	0.00125193	5	Arl6ip4
5.50E-08	0.91368206	0.441	0.433	0.00133273	5	Ppp1r14b
5.72E-08	0.39857906	0.153	0.318	0.00138792	5	Senp6
5.87E-08	0.30795549	0.118	0.261	0.00142424	5	Acadvl
6.01E-08	0.30326436	0.116	0.257	0.00145765	5	Appl1
6.52E-08	0.26615785	0.237	0.452	0.00158093	5	Papola
6.54E-08	0.96428729	0.561	0.648	0.00158537	5	Sh3bgrl

7.25E-08	0.30129095	0.176	0.349	0.00175885	5	Anapc11
7.32E-08	0.33481974	0.128	0.274	0.00177499	5	Magoh
7.33E-08	0.274826	0.116	0.257	0.00177812	5	Mrps16
7.72E-08	0.2626895	0.213	0.406	0.00187267	5	Ndufs2
7.75E-08	0.46840828	0.239	0.452	0.00187848	5	Ppig
8.79E-08	0.32577495	0.193	0.379	0.00213027	5	Ube2j1
8.88E-08	0.31849265	0.144	0.297	0.00215366	5	Ubxn6
9.28E-08	0.30537401	0.155	0.317	0.00224932	5	Lsm7
9.48E-08	0.76613665	0.501	0.512	0.00229927	5	Rps7
1.07E-07	0.30005259	0.118	0.256	0.00259588	5	Vamp7
1.08E-07	0.25240543	0.142	0.292	0.00262035	5	Dctn2
1.09E-07	0.75055897	0.548	0.612	0.00263307	5	Atp5e
1.45E-07	0.30427357	0.153	0.309	0.00351224	5	Mrps17
1.49E-07	0.27896417	0.193	0.37	0.00361381	5	Ptbp1
1.52E-07	0.26107468	0.193	0.368	0.00367878	5	Cops4
1.62E-07	0.30530551	0.137	0.284	0.00393991	5	Lypla1
1.84E-07	0.37843691	0.135	0.28	0.00445349	5	Babam1
2.10E-07	0.65184539	0.608	0.656	0.00509144	5	Hspa9
2.18E-07	0.36808439	0.128	0.264	0.00529652	5	Nes
2.41E-07	0.40250596	0.135	0.278	0.00584885	5	Abhd17a
2.50E-07	1.19288566	0.318	0.272	0.00606885	5	Cfl2

3.46E-07	0.2988614	0.146	0.291	0.00839502	5	Dap3
3.82E-07	0.26449363	0.13	0.271	0.00926137	5	Mbd3
3.95E-07	0.47153992	0.121	0.256	0.00958047	5	Rbms2
3.98E-07	0.33969531	0.118	0.252	0.00964602	5	Ccser2
4.28E-07	0.31697465	0.2	0.376	0.01038147	5	Ddx39
4.29E-07	0.25213014	0.123	0.258	0.01039354	5	Eef1e1
4.67E-07	0.271175	0.239	0.437	0.01132551	5	Fxr1
4.88E-07	0.47949458	0.731	0.851	0.01183954	5	Slc25a3
5.38E-07	0.2687032	0.248	0.448	0.01304052	5	Ifitm2
5.56E-07	0.32091645	0.223	0.414	0.01347442	5	Dctn1
5.67E-07	0.2997359	0.213	0.394	0.01374573	5	Eif3d
5.81E-07	0.29487363	0.125	0.259	0.01409125	5	Zc3h14
6.16E-07	0.62254697	0.585	0.636	0.01493692	5	Rpl29
6.46E-07	0.37008447	0.172	0.328	0.01565629	5	Ivns1abp
7.02E-07	0.36933666	0.132	0.269	0.01702183	5	Msi2
7.15E-07	0.3018734	0.269	0.485	0.01732661	5	Ndufb3
7.28E-07	0.25895221	0.144	0.287	0.01765638	5	Dlst
7.60E-07	0.33744638	0.162	0.316	0.01841752	5	Emc2
8.20E-07	0.30421634	0.206	0.381	0.01987462	5	Kmt5a
8.33E-07	0.34332801	0.148	0.294	0.02018778	5	Tubb4b
9.57E-07	0.33404131	0.121	0.25	0.02320953	5	Rnf10

9.74E-07	0.25002803	0.244	0.438	0.02362296	5	Litaf
1.07E-06	0.7426681	0.524	0.594	0.0258587	5	Atpif1
1.10E-06	0.2621742	0.2	0.367	0.02676256	5	Tsg101
1.17E-06	0.44570791	0.137	0.276	0.0284746	5	Nrd1
1.34E-06	0.36967002	0.132	0.266	0.03255825	5	Cetn2
1.38E-06	0.94944794	0.408	0.404	0.03335828	5	Rpl13a
1.39E-06	0.37391424	0.139	0.277	0.03372351	5	Zmat3
1.41E-06	0.60096464	0.677	0.794	0.0342411	5	Aldoa
1.65E-06	0.41329706	0.211	0.392	0.04012347	5	Cript
1.71E-06	0.34357346	0.151	0.291	0.04139164	5	Cetn3
1.83E-06	0.58486825	0.599	0.689	0.04445286	5	Uqcrh
1.85E-06	0.30735957	0.246	0.436	0.04497316	5	1110008F13Rik
1.89E-06	1.74466526	0.297	0.258	0.04592285	5	Ddr2
2.04E-06	0.69421001	0.548	0.613	0.04934585	5	Hnrnpab
2.05E-06	0.26902068	0.165	0.312	0.04965762	5	4833439L19Rik
2.07E-06	0.44630416	0.234	0.422	0.050122	5	Ppp1r2
2.09E-06	0.35462349	0.142	0.276	0.05061924	5	Tfdp1
2.11E-06	0.32249782	0.128	0.256	0.05122493	5	Dda1
2.26E-06	0.39490515	0.137	0.27	0.0548167	5	Ankrd13c
2.36E-06	0.94559748	0.432	0.451	0.05725297	5	Arf4
2.39E-06	1.00757399	0.304	0.259	0.05802718	5	Ryk
2.62E-06	0.32918415	0.237	0.425	0.06353924	5	Ctbp1

2.67E-06	0.42870499	0.13	0.26	0.06474151	5	Klhdc2
2.81E-06	0.28010686	0.23	0.416	0.06822112	5	Ddx21
2.83E-06	0.32928907	0.183	0.339	0.06856009	5	Mrpl30
2.93E-06	1.00952729	0.441	0.471	0.0711613	5	Vdac1
3.00E-06	0.40652892	0.22	0.397	0.07283641	5	Tbcb
3.48E-06	0.40390958	0.2	0.36	0.08447752	5	Dennd5a
3.48E-06	0.31528689	0.176	0.328	0.08449135	5	Uqcrc2
3.49E-06	0.88084212	0.455	0.49	0.08462317	5	Cct2
3.51E-06	0.25761763	0.29	0.515	0.08522669	5	Dnaja2
3.55E-06	0.3442693	0.142	0.274	0.0860352	5	Prelid3b
3.65E-06	0.29900721	0.135	0.265	0.08846543	5	Pdxdc1
3.70E-06	0.28567275	0.225	0.398	0.08972346	5	Mrps33
4.07E-06	0.42174369	0.211	0.383	0.09857962	5	Cyb5b
4.32E-06	1.10568907	0.392	0.4	0.10463173	5	Snrpf
4.37E-06	0.29496537	0.19	0.35	0.105978	5	Eif3m
4.45E-06	1.21240892	0.343	0.315	0.10798673	5	Atf5
4.54E-06	0.44003606	0.183	0.34	0.1101579	5	Egln1
4.60E-06	0.34243264	0.144	0.277	0.1114632	5	Idh3b
4.79E-06	0.47399647	0.13	0.257	0.11624028	5	Pgm2
4.82E-06	0.28923045	0.188	0.341	0.11679097	5	Rnf187
5.15E-06	0.34800104	0.167	0.31	0.12477482	5	Nfu1
5.59E-06	1.00972658	0.367	0.349	0.13556555	5	Pdap1

5.80E-06	0.2728279	0.295	0.503	0.14066556	5	Glrx5
5.82E-06	0.35083962	0.135	0.261	0.14105573	5	Gna11
5.94E-06	0.9151695	0.445	0.476	0.14404042	5	Eif2s3x
6.00E-06	0.51383142	0.132	0.261	0.14558651	5	Trim44
6.05E-06	0.77564411	0.501	0.569	0.146577	5	Bzw1
6.11E-06	0.31916621	0.148	0.281	0.14814867	5	Mtch2
6.31E-06	0.3830872	0.223	0.401	0.15289387	5	Rrp1
6.72E-06	0.98724576	0.413	0.436	0.16290545	5	Rexo2
6.73E-06	0.37786208	0.262	0.453	0.16327973	5	Commd3
6.78E-06	0.37483338	0.204	0.367	0.16433714	5	Gnl1
7.66E-06	0.32844677	0.158	0.294	0.18572974	5	Ube2e1
7.81E-06	0.65857513	0.564	0.626	0.18936273	5	Rpl27a
7.90E-06	0.29314069	0.197	0.352	0.19151886	5	Etfb
8.63E-06	0.3615405	0.142	0.269	0.20920992	5	Gps1
8.85E-06	0.2834523	0.2	0.359	0.21452052	5	Ubqln1
9.38E-06	0.30525437	0.211	0.376	0.22744604	5	Psmb2
9.48E-06	0.36121231	0.183	0.333	0.22994957	5	Hbs1l
9.78E-06	0.63050459	0.617	0.749	0.23715278	5	Csde1
9.90E-06	0.37548742	0.181	0.327	0.24002156	5	Rheb
1.00E-05	0.29067064	0.281	0.49	0.24248196	5	Dnaja1
1.01E-05	0.35959693	0.281	0.485	0.24467959	5	Celf1

1.02E-05	0.38032329	0.13	0.25	0.24841453	5	Ruvbl1
1.09E-05	0.29252301	0.302	0.516	0.26309833	5	Trir
1.09E-05	0.90952294	0.48	0.508	0.2647286	5	Rock2
1.15E-05	0.79858211	0.494	0.559	0.27778464	5	Hnrnpa3
1.16E-05	0.3413146	0.155	0.288	0.28034256	5	Mrto4
1.19E-05	0.44681611	0.179	0.326	0.28944223	5	Tbl1x
1.35E-05	1.40906027	0.411	0.439	0.32616914	5	Kif1c
1.38E-05	0.25110138	0.186	0.325	0.3347574	5	Maged1
1.38E-05	0.41330516	0.139	0.264	0.3353851	5	Bzw2
1.42E-05	0.32920867	0.299	0.511	0.34444646	5	Tpr
1.44E-05	0.26453691	0.271	0.466	0.34819266	5	Srsf6
1.55E-05	0.93266586	0.441	0.472	0.37485177	5	Hnrnpa1
1.55E-05	0.33700937	0.135	0.253	0.37546432	5	Suclg1
1.55E-05	0.43181337	0.181	0.326	0.37590816	5	Lap3
1.59E-05	0.47188349	0.647	0.77	0.38467696	5	Capzb
1.64E-05	0.33912986	0.26	0.443	0.39770728	5	Ssrp1
1.74E-05	0.63644886	0.483	0.487	0.42223567	5	Rpl36
1.83E-05	0.36560711	0.234	0.411	0.44430694	5	Sae1
1.85E-05	0.31536719	0.255	0.44	0.44806181	5	Hnrnpd
1.87E-05	0.38144342	0.234	0.404	0.45428356	5	Pole4
2.02E-05	0.49537666	0.195	0.34	0.49062439	5	Dst

2.08E-05	0.50853484	0.148	0.277	0.50336109	5	Myef2
2.16E-05	0.32533359	0.144	0.267	0.52311075	5	Ddx54
2.20E-05	0.32582662	0.251	0.431	0.53396927	5	Tomm22
2.23E-05	0.2688903	0.137	0.256	0.53952043	5	Emd
2.25E-05	0.33109354	0.232	0.393	0.54532577	5	Rab6a
2.29E-05	1.80682448	0.299	0.274	0.55450201	5	Sh3pxd2a
2.33E-05	0.3866572	0.19	0.335	0.56517856	5	Ndufa13
2.43E-05	0.49416771	0.19	0.342	0.58804942	5	Zranb2
2.43E-05	0.9409297	0.425	0.444	0.58834177	5	Gspt1
2.46E-05	0.42447425	0.165	0.3	0.59606147	5	Usp16
2.65E-05	0.34042917	0.288	0.486	0.64222451	5	Psmd4
2.66E-05	0.29816153	0.295	0.497	0.64435805	5	Sf3b5
2.72E-05	0.38727669	0.258	0.447	0.66035325	5	Elavl1
3.11E-05	0.45239638	0.155	0.284	0.75510996	5	Srf
3.12E-05	0.39723449	0.197	0.349	0.75731716	5	Nsun2
3.14E-05	0.69851723	0.51	0.587	0.76021373	5	Romo1
3.16E-05	0.35461109	0.137	0.255	0.76669305	5	Snhg6
3.21E-05	0.92675589	0.429	0.464	0.77738025	5	Snrpe
3.26E-05	0.36337062	0.174	0.312	0.79062314	5	Psmd14
3.35E-05	0.37883875	0.209	0.359	0.81300669	5	Eny2
3.41E-05	0.38394176	0.158	0.287	0.82688682	5	Eif2b5

3.49E-05	0.27924451	0.186	0.325	0.84582698	5	Mrpl51
3.63E-05	0.34388745	0.232	0.393	0.88066844	5	Zyx
3.80E-05	0.30930951	0.172	0.3	0.92122116	5	Prkar2b
3.81E-05	0.33209394	0.251	0.428	0.92463052	5	Rbm8a
3.81E-05	0.33596304	0.253	0.426	0.92484922	5	Map1lc3b
3.88E-05	0.31183741	0.188	0.329	0.94064093	5	Dazap1
3.96E-05	0.36293064	0.142	0.26	0.96005388	5	Dock1
4.11E-05	0.3938003	0.213	0.365	0.99675055	5	Napa
5.40E-05	0.31537751	0.318	0.526	1	5	Glud1
5.53E-05	0.45819025	0.169	0.302	1	5	Ttc3
5.80E-05	0.36142342	0.165	0.293	1	5	Nudt21
6.26E-05	0.32922389	0.285	0.479	1	5	Cdk11b
6.40E-05	0.38816522	0.186	0.32	1	5	Psmc5
6.45E-05	0.36705133	0.176	0.309	1	5	Psmc2
6.55E-05	0.51778631	0.155	0.28	1	5	Ssbp3
6.66E-05	0.41110523	0.155	0.276	1	5	Actr10
6.77E-05	0.39382043	0.195	0.337	1	5	Sri
6.86E-05	0.49993887	0.176	0.312	1	5	Samm50
7.34E-05	0.83068305	0.436	0.48	1	5	Psma7
7.56E-05	0.30132034	0.269	0.454	1	5	Sdhc
7.70E-05	0.26477163	0.239	0.402	1	5	Ndufa1

7.87E-05	0.35834039	0.246	0.409	1	5	Map2k2
8.41E-05	0.45471271	0.162	0.286	1	5	5-Mar
8.48E-05	0.6241832	0.573	0.684	1	5	Wdr1
9.37E-05	0.66507112	0.49	0.562	1	5	Hint1
9.40E-05	1.05657147	0.29	0.262	1	5	Cttn
9.56E-05	0.3729091	0.176	0.309	1	5	Mrpl57
0.00010172	0.37637907	0.239	0.4	1	5	Txnl1
0.00010593	0.27569655	0.327	0.537	1	5	Syncrip
0.0001064	0.4640842	0.162	0.285	1	5	Dars
0.00010648	0.66703514	0.515	0.577	1	5	Rpl10a
0.00011816	1.18844972	0.285	0.253	1	5	Vgll3
0.00011864	0.39333039	0.216	0.364	1	5	Nol7
0.00012694	0.81900512	0.385	0.397	1	5	Mrpl52
0.00012753	1.01279603	0.367	0.375	1	5	Eif1ax
0.00012873	0.49577445	0.629	0.771	1	5	Arhgdia
0.00013761	0.47354155	0.155	0.273	1	5	Pcgf3
0.00014282	0.39184826	0.223	0.38	1	5	Phb2
0.00015778	0.38683912	0.248	0.417	1	5	Ndufa10
0.00017892	0.289309	0.169	0.286	1	5	Ufc1
0.00018227	1.05605779	0.299	0.274	1	5	Msrb3
0.00018265	0.49193686	0.195	0.334	1	5	Kpna4
0.00018784	1.05088064	0.329	0.314	1	5	Tubb6
0.00019007	0.42129495	0.234	0.396	1	5	Anxa7

0.00019688	0.49273407	0.278	0.461	1	5	Rars
0.00020392	0.96766474	0.378	0.393	1	5	Ddb1
0.00020754	0.37349314	0.153	0.265	1	5	Slirp
0.00021512	0.55637815	0.216	0.369	1	5	Dcun1d5
0.00022161	0.33703997	0.318	0.514	1	5	Srsf3
0.00023483	0.97457166	0.362	0.37	1	5	S100a13
0.00023966	0.51138152	0.158	0.274	1	5	Wwc2
0.00025088	0.39129289	0.202	0.339	1	5	Zc3h15
0.00027251	0.4306011	0.216	0.357	1	5	Nudcd2
0.00028361	0.27483549	0.313	0.513	1	5	Ndufs6
0.00029121	0.40373454	0.186	0.313	1	5	Lsm6
0.00030124	0.45416577	0.225	0.375	1	5	Wsb2
0.00030381	0.44166079	0.211	0.349	1	5	Lsm5
0.0003043	0.35825108	0.206	0.347	1	5	Tmem258
0.00030882	0.44693726	0.213	0.357	1	5	Smarca5
0.00031565	0.53693053	0.559	0.689	1	5	Atp5b
0.00032722	0.69342219	0.503	0.595	1	5	Psmd8
0.00034279	0.79931022	0.452	0.51	1	5	Swi5
0.00035671	0.44151643	0.179	0.299	1	5	Acadl
0.00036069	0.44662735	0.181	0.305	1	5	Hras
0.00036435	0.40214026	0.172	0.286	1	5	Smc4

0.00037433	0.39916467	0.148	0.254	1	5	Cdk2ap1
0.00038254	0.4960605	0.241	0.4	1	5	Polr2m
0.00038542	0.28121636	0.285	0.458	1	5	Eif3h
0.00042032	0.43990782	0.151	0.259	1	5	Kars
0.00044083	0.86262235	0.471	0.537	1	5	Eif3c
0.00044261	0.5404087	0.218	0.363	1	5	Zcrb1
0.00044952	0.43280135	0.183	0.311	1	5	Ddx1
0.00045081	1.02046194	0.304	0.29	1	5	Fam162a
0.00046656	0.50130364	0.548	0.665	1	5	Cox6b1
0.0004773	0.45574522	0.188	0.314	1	5	Fkbp4
0.00049052	0.49264665	0.248	0.407	1	5	Strap
0.00052047	0.29365703	0.406	0.65	1	5	Ctnnb1
0.00052993	0.50892961	0.148	0.253	1	5	Suclg2
0.00053137	0.48481911	0.26	0.426	1	5	Akt1
0.00054565	0.48384116	0.179	0.299	1	5	Eif3g
0.00055134	0.39351762	0.32	0.514	1	5	Nop10
0.00056074	0.88858173	0.394	0.424	1	5	Atp5j
0.00057377	0.61816785	0.223	0.369	1	5	Synj2bp
0.00057789	0.4711402	0.596	0.738	1	5	Sh3glb1
0.00059489	0.42730548	0.629	0.776	1	5	Hnrnpk
0.00059691	0.39316165	0.262	0.421	1	5	U2af2

0.00060554	0.42573302	0.169	0.285	1	5	Mrpl13
0.00060959	0.55090571	0.246	0.4	1	5	Usp34
0.0006317	0.61350616	0.52	0.628	1	5	Arf1
0.00063376	0.44760348	0.297	0.479	1	5	Psmc1
0.00065445	0.39796006	0.274	0.438	1	5	Snhg8
0.00066587	0.29235563	0.318	0.502	1	5	Sfpq
0.00066592	0.40810137	0.283	0.459	1	5	Rbm17
0.00066661	0.36807714	0.206	0.34	1	5	Edf1
0.0006754	0.45288659	0.237	0.385	1	5	Fam129b
0.00068676	0.50503999	0.183	0.302	1	5	Galk1
0.00070037	0.36406253	0.148	0.25	1	5	Mpp6
0.00074306	0.53677625	0.248	0.407	1	5	Dnajc8
0.00077445	1.05302867	0.35	0.363	1	5	Map4
0.00078415	0.63660139	0.536	0.647	1	5	2010107E04Rik
0.00079212	0.44511317	0.197	0.324	1	5	Dtymk
0.00085218	0.61974888	0.148	0.252	1	5	Rras
0.00085331	0.59665018	0.283	0.453	1	5	Itpripl2
0.00085677	0.71846571	0.471	0.55	1	5	Tomm7
0.00093488	0.40807411	0.209	0.338	1	5	Nhp2
0.00099639	0.48242657	0.2	0.324	1	5	Snrnp27
0.00100504	0.76614082	0.425	0.48	1	5	Atp5o.1

0.0010261 1	0.472406	0.193	0.311	1	5	Cops2
0.0010383 6	0.4590561 9	0.271	0.432	1	5	Ccni
0.0010740 6	0.4080098 5	0.329	0.525	1	5	1810037I17Rik
0.001081	0.4448967 9	0.253	0.409	1	5	Nudt3
0.0011009 3	0.3883603 1	0.251	0.396	1	5	Arcn1
0.0011013 8	0.9310665 5	0.36	0.377	1	5	Eif3i
0.0011046 7	0.4318185	0.19	0.307	1	5	Lars
0.0011554 9	1.1137753 2	0.274	0.258	1	5	Crip2
0.0011626 6	0.4415111 1	0.165	0.27	1	5	Uqcrc1
0.0011742 9	0.9497436 2	0.306	0.292	1	5	S100a16
0.0011943 9	0.5710252 9	0.183	0.297	1	5	Tnfaip1
0.0011992 1	0.4321130 5	0.23	0.37	1	5	Mapk6
0.0013322 8	0.4344296 9	0.255	0.405	1	5	Ipo7
0.0013448 5	0.5284091 7	0.206	0.329	1	5	Mettl9
0.0013596 3	0.4395968 1	0.153	0.253	1	5	Tbrg1
0.0013884 4	0.4614475 8	0.186	0.301	1	5	Map1lc3a
0.0013934 1	0.3065256 4	0.172	0.276	1	5	Uba52
0.0014459 7	0.4233949 5	0.197	0.315	1	5	Mybbp1a
0.0014846 6	0.4498357 5	0.202	0.327	1	5	Snrpc
0.0015057 3	0.4873550 2	0.155	0.255	1	5	Pum3
0.0015811 4	0.5712619 2	0.169	0.277	1	5	Cox20

0.00161243	0.58152774	0.193	0.31	1	5	Sltm
0.00173907	0.42824507	0.213	0.335	1	5	Smurf2
0.00187033	0.42482481	0.253	0.399	1	5	Tsen34
0.00188092	0.45526843	0.155	0.252	1	5	Dlat
0.00191364	0.44795275	0.271	0.415	1	5	Mbnl2
0.0019772	0.46374575	0.225	0.357	1	5	Mrps14
0.00199905	0.47753927	0.204	0.327	1	5	Psmc4
0.00203956	0.50732471	0.237	0.372	1	5	Myo1c
0.00206474	0.50961259	0.223	0.352	1	5	Rala
0.00221102	0.4383889	0.239	0.378	1	5	Raly
0.00235225	0.48506247	0.19	0.303	1	5	Npm3
0.00241023	0.64154168	0.234	0.37	1	5	Bnip3
0.00241943	0.41238495	0.265	0.418	1	5	Csnk2b
0.00253192	0.51397849	0.204	0.322	1	5	Sec13
0.00259723	0.3116451	0.234	0.36	1	5	Selenow
0.00266414	0.75095483	0.466	0.529	1	5	Map4k4
0.00274428	0.48566031	0.281	0.441	1	5	Sin3b
0.00282321	0.44168003	0.26	0.404	1	5	Ilk
0.00305812	0.31782013	0.353	0.556	1	5	Aes
0.00307875	0.75544654	0.443	0.522	1	5	Rpl27
0.00308839	0.46304354	0.193	0.305	1	5	Nop58

0.00322203	0.4695227	0.239	0.379	1	5	Abcf1
0.00340215	0.44695299	0.23	0.363	1	5	Ndufa8
0.00344088	0.84423663	0.26	0.249	1	5	Ehd2
0.00350144	0.39093598	0.183	0.286	1	5	Ndufs5
0.00350403	0.44017816	0.186	0.291	1	5	Hars
0.00355614	0.53908942	0.2	0.317	1	5	Sugt1
0.00370066	0.77302976	0.399	0.451	1	5	Eif3b
0.00381602	0.43892222	0.248	0.383	1	5	Rbbp7
0.00384715	0.50863886	0.225	0.354	1	5	Tomm5
0.00393293	0.717262	0.439	0.484	1	5	Rpl22l1
0.0040219	0.91676671	0.401	0.462	1	5	Cox7b
0.0040487	0.55286362	0.244	0.379	1	5	Rpl36al
0.00406087	0.29730825	0.327	0.501	1	5	Clic1
0.00422359	0.38509623	0.225	0.344	1	5	Atp5g1
0.00440722	0.79773307	0.392	0.438	1	5	Prmt1
0.00466798	0.74659909	0.443	0.533	1	5	Cct7
0.0046756	0.51776753	0.211	0.329	1	5	Snrpd1
0.00472952	0.69649583	0.478	0.57	1	5	Atp5f1
0.00497251	0.59159954	0.169	0.269	1	5	Smyd2
0.00498844	0.53162649	0.19	0.297	1	5	Tmem160
0.00559432	0.51838132	0.176	0.277	1	5	Prdx3

0.00560067	0.42524035	0.299	0.46	1	5	Nono
0.00563663	0.41566655	0.255	0.396	1	5	Tmem167
0.00590403	0.36805811	0.267	0.411	1	5	Nudt4
0.00590576	0.44310013	0.193	0.297	1	5	Ccdc124
0.00596541	0.33932589	0.223	0.335	1	5	Mthfd2
0.00609378	0.54872	0.225	0.344	1	5	Gsto1
0.00634478	0.44206447	0.276	0.427	1	5	Llph
0.00656843	0.28286406	0.392	0.595	1	5	Ndufa3
0.00716774	0.91324928	0.411	0.477	1	5	Ranbp1
0.00720333	0.55942891	0.237	0.367	1	5	Ndufs4
0.00742232	0.27507338	0.381	0.579	1	5	Prdx5
0.00754792	0.63071102	0.167	0.255	1	5	Cks1b
0.00791555	0.55276801	0.186	0.287	1	5	Psmd7
0.00811149	0.57249824	0.19	0.292	1	5	Ebna1bp2
0.00822876	0.69220106	0.452	0.543	1	5	Dap
0.00824765	0.49062409	0.216	0.332	1	5	Prdx6
0.00825328	0.37043549	0.239	0.363	1	5	Ak2
0.00884559	0.45949114	0.181	0.275	1	5	Sec23a
0.00924343	0.80967288	0.429	0.512	1	5	Atp5k
0.0092718	0.73302408	0.211	0.326	1	5	Nbeal1
0.00944284	0.45615341	0.269	0.409	1	5	Pdha1

0.0095234	0.40306068	0.318	0.486	1	5	Txn2
0.00971753	0.54448102	0.206	0.317	1	5	Ndufa5
0.00985225	0.77974875	0.411	0.469	1	5	Cct5
3.02E-100	1.11071511	1	0.964	7.31E-96	6	Apoe
1.89E-88	1.00557003	0.993	0.869	4.59E-84	6	BC005537
4.06E-82	0.76716038	0.995	0.935	9.85E-78	6	B2m
5.14E-81	1.04230474	0.846	0.439	1.25E-76	6	Stab1
5.93E-81	1.18028879	0.643	0.234	1.44E-76	6	F13a1
1.28E-76	0.89207108	0.988	0.826	3.09E-72	6	H2-K1
2.52E-75	1.03676865	0.911	0.551	6.12E-71	6	Cybb
2.33E-68	1.27876352	0.492	0.156	5.64E-64	6	Cx3cr1
4.63E-68	0.74238058	0.333	0.075	1.12E-63	6	F630028O10Rik
4.63E-67	0.9502305	0.858	0.454	1.12E-62	6	Ly86
1.51E-62	0.5659172	1	0.995	3.67E-58	6	Tmsb4x
9.47E-62	0.84670437	0.914	0.604	2.30E-57	6	Itgam
1.37E-60	0.77051203	0.995	0.859	3.32E-56	6	Lgmn
8.45E-60	0.80732682	0.974	0.685	2.05E-55	6	C1qc
1.15E-59	0.98410957	0.797	0.469	2.79E-55	6	Smpdl3a
6.00E-58	0.79925336	0.963	0.772	1.45E-53	6	H2-D1
8.80E-58	0.88845008	0.685	0.307	2.13E-53	6	Ccr5
4.88E-57	0.70134717	0.998	0.914	1.18E-52	6	Ctsd
5.51E-57	0.77657076	0.981	0.773	1.33E-52	6	Laptm5

6.42E-57	0.93089614	0.552	0.224	1.56E-52	6	Cadm1
6.72E-57	0.81721559	0.883	0.605	1.63E-52	6	Rcbtb2
9.47E-57	0.70592817	0.988	0.855	2.30E-52	6	Itm2b
1.14E-56	0.80226506	0.846	0.571	2.76E-52	6	Mef2a
2.94E-56	0.72811167	0.83	0.478	7.12E-52	6	Pf4
3.56E-56	0.83400686	0.937	0.715	8.64E-52	6	Maf
2.09E-54	0.81005361	0.867	0.577	5.07E-50	6	Ctsc
1.15E-53	0.96001592	0.881	0.593	2.78E-49	6	C1qa
5.87E-53	0.80750847	0.648	0.303	1.42E-48	6	Itgal
5.97E-53	0.81355475	0.709	0.365	1.45E-48	6	C5ar1
1.25E-51	0.8592892	0.779	0.449	3.02E-47	6	Pla2g7
3.57E-51	0.69731515	0.995	0.921	8.66E-47	6	Lyz2
7.19E-50	0.66219952	1	0.976	1.74E-45	6	Ctsb
3.27E-49	0.81778294	0.611	0.293	7.92E-45	6	Itga6
5.03E-49	0.76551741	0.364	0.124	1.22E-44	6	Gm11942
6.77E-49	0.82711837	0.765	0.489	1.64E-44	6	Nampt
9.01E-49	0.79268318	0.709	0.377	2.18E-44	6	Msr1
1.40E-48	0.71668576	0.967	0.751	3.39E-44	6	Creg1
1.40E-48	0.63961914	0.767	0.396	3.39E-44	6	Aif1
4.33E-48	0.73778738	0.632	0.292	1.05E-43	6	Ms4a6c
6.45E-48	0.74925112	0.823	0.484	1.56E-43	6	Ms4a7
2.14E-47	0.72549354	0.797	0.444	5.19E-43	6	Cd84

3.33E-47	0.66664203	0.981	0.787	8.06E-43	6	Lcp1
1.53E-46	0.63598012	0.946	0.728	3.70E-42	6	Rsrp1
2.04E-46	0.70581765	0.695	0.353	4.94E-42	6	Nrros
3.37E-46	0.70933853	0.748	0.41	8.18E-42	6	Csf1r
3.65E-45	0.72024528	0.741	0.426	8.85E-41	6	Fli1
1.88E-44	0.7439489	0.776	0.456	4.55E-40	6	Trem2
5.21E-44	0.61056245	0.986	0.831	1.26E-39	6	Grn
5.46E-44	0.84685561	0.818	0.567	1.32E-39	6	Lipa
6.16E-44	0.67902314	0.762	0.417	1.49E-39	6	C3ar1
6.27E-44	0.57510111	0.967	0.785	1.52E-39	6	Mbnl1
1.45E-43	0.72509888	0.562	0.232	3.51E-39	6	Xist
2.68E-43	0.69643616	0.879	0.568	6.51E-39	6	Igf1
2.13E-42	0.63454829	0.816	0.506	5.15E-38	6	Igfbp4
3.98E-42	0.77594944	0.767	0.501	9.64E-38	6	Gas6
7.52E-42	0.73033458	0.751	0.436	1.82E-37	6	Rnf128
7.26E-41	0.58603147	0.862	0.543	1.76E-36	6	Cd93
8.64E-41	0.59787462	0.988	0.777	2.10E-36	6	Mpeg1
1.31E-40	0.61990398	0.967	0.787	3.19E-36	6	Sirpa
3.47E-40	0.63825896	0.671	0.362	8.40E-36	6	Tm6sf1
5.24E-40	0.61223898	0.876	0.583	1.27E-35	6	C1qb
5.33E-40	0.607709	0.907	0.718	1.29E-35	6	Efhd2
2.25E-38	0.55864797	0.45	0.185	5.46E-34	6	Slc7a8

3.10E-38	0.60457775	0.828	0.567	7.52E-34	6	Il10rb
5.28E-38	0.77632797	0.627	0.36	1.28E-33	6	Rab32
1.01E-37	0.59575126	0.343	0.119	2.44E-33	6	Fcrls
1.43E-37	0.65672897	0.811	0.55	3.46E-33	6	Slc43a2
3.02E-37	0.55589623	0.657	0.35	7.32E-33	6	Inpp5d
5.02E-37	0.59039174	0.716	0.417	1.22E-32	6	Nceh1
6.58E-37	0.66956394	0.727	0.466	1.59E-32	6	Zfp36
6.80E-37	0.67177032	0.963	0.779	1.65E-32	6	Ctsl
9.49E-37	0.70131919	0.769	0.549	2.30E-32	6	Adam15
1.00E-36	0.67151661	0.73	0.433	2.43E-32	6	Pid1
2.47E-35	0.67809615	0.841	0.638	5.98E-31	6	Id2
3.33E-35	0.68300482	0.657	0.42	8.07E-31	6	Bst2
4.27E-35	0.6281875	0.611	0.317	1.03E-30	6	Clec4n
4.93E-35	0.59963516	0.529	0.252	1.20E-30	6	Dock10
1.15E-34	0.42974627	0.331	0.115	2.79E-30	6	Gpr65
4.24E-34	0.78505149	0.594	0.357	1.03E-29	6	Id1
4.59E-34	0.63197347	0.606	0.355	1.11E-29	6	Dapk1
4.73E-34	0.55707091	0.865	0.586	1.15E-29	6	Fcer1g
4.83E-34	0.64859143	0.706	0.469	1.17E-29	6	Sh3bp5
6.18E-34	0.47841289	0.974	0.838	1.50E-29	6	Ctss
7.78E-34	0.46274033	0.988	0.839	1.89E-29	6	Lamp1
1.50E-33	0.56988482	0.636	0.348	3.63E-29	6	Rbm47

5.10E-33	0.5639095<br>5	0.685	0.386	1.24E-28	6	Clec4a1
7.54E-33	0.5435732	0.814	0.543	1.83E-28	6	Ftl1-ps1
7.87E-33	0.5303355<br>3	0.667	0.372	1.91E-28	6	Coro1a
1.46E-32	0.5786429<br>1	0.807	0.583	3.54E-28	6	Unc93b1
1.78E-32	0.5980693<br>4	0.569	0.296	4.31E-28	6	Ms4a6d
2.12E-32	0.5661607<br>6	0.879	0.687	5.15E-28	6	Tmem50a
2.39E-32	0.6126951<br>6	0.816	0.547	5.80E-28	6	Mafb
3.06E-32	0.3914740<br>8	1	0.964	7.42E-28	6	Psap
4.57E-32	0.6020615<br>1	0.664	0.406	1.11E-27	6	Neu1
7.20E-32	0.5474891<br>7	0.49	0.229	1.75E-27	6	Il10ra
7.28E-32	0.5809641<br>7	0.767	0.495	1.76E-27	6	Itgb2
3.90E-31	0.5371294<br>9	0.907	0.717	9.47E-27	6	Serinc3
5.94E-31	0.5520277<br>4	0.839	0.632	1.44E-26	6	Atp2b1
6.00E-31	0.5170052<br>8	0.895	0.675	1.46E-26	6	Tyrobp
8.62E-31	0.4755632<br>5	0.832	0.62	2.09E-26	6	Atp6v0b
1.30E-30	0.5051836<br>5	0.958	0.775	3.15E-26	6	Arpc4
1.39E-30	0.5694902<br>3	0.452	0.214	3.36E-26	6	Zdhhc14
1.48E-30	0.5391249<br>1	0.918	0.77	3.59E-26	6	Dazap2
2.21E-30	0.5289737<br>5	0.858	0.667	5.35E-26	6	Cyba
2.33E-30	0.6508640<br>9	0.62	0.361	5.66E-26	6	Fcgr2b
2.39E-30	0.5237584<br>9	0.874	0.673	5.78E-26	6	Cebpb
2.87E-30	0.6464286<br>3	0.713	0.505	6.96E-26	6	Abhd12

3.78E-30	0.5856778	0.725	0.51	9.16E-26	6	Ap1b1
7.88E-30	0.67931	0.695	0.486	1.91E-25	6	Dusp6
9.06E-30	0.4506348 5	0.811	0.533	2.20E-25	6	Arhgdib
1.01E-29	0.6158019 7	0.755	0.546	2.44E-25	6	Wwp1
1.24E-29	0.5568568 2	0.471	0.226	3.02E-25	6	Cd72
1.27E-29	0.4489944 2	0.427	0.19	3.09E-25	6	Nfam1
1.32E-29	0.4737025 1	0.867	0.619	3.21E-25	6	Cotl1
1.81E-29	0.5077636 1	0.923	0.761	4.40E-25	6	Zeb2
2.12E-29	0.4614247 3	0.583	0.309	5.14E-25	6	Cfp
2.37E-29	0.5044916	0.639	0.356	5.75E-25	6	Fcgr3
3.10E-29	0.5488137 5	0.545	0.283	7.53E-25	6	Clec4a2
7.13E-29	0.6148189 2	0.55	0.307	1.73E-24	6	Apobec1
8.12E-29	0.6215608 6	0.58	0.333	1.97E-24	6	Adam8
1.39E-28	0.4122093 2	0.925	0.692	3.38E-24	6	Cd36
1.61E-28	0.5835204	0.541	0.291	3.91E-24	6	Fcgr4
1.64E-28	0.5463245	0.914	0.708	3.97E-24	6	Mrc1
2.12E-28	0.4316394 3	0.967	0.832	5.14E-24	6	Sdcbp
2.61E-28	0.6619616 7	0.683	0.5	6.32E-24	6	Ptpra
2.82E-28	0.4993383 6	0.816	0.63	6.85E-24	6	Grk2
2.94E-28	0.5758822 5	0.751	0.547	7.13E-24	6	Tmem30a
4.77E-28	0.4915375 8	0.825	0.617	1.16E-23	6	Arl6ip1
5.88E-28	0.5320067 1	0.788	0.603	1.43E-23	6	Fam49b
1.33E-27	0.4985751 8	0.562	0.302	3.22E-23	6	Ncf2

1.33E-27	0.44108082	0.573	0.308	3.22E-23	6	Ptpn18
1.84E-27	0.52945797	0.683	0.444	4.46E-23	6	Tnfrsf1b
2.53E-27	0.46496425	0.364	0.156	6.13E-23	6	Ikzf1
2.54E-27	0.26796949	0.998	0.929	6.16E-23	6	Prdx1
3.32E-27	0.45814552	0.923	0.725	8.05E-23	6	Adgre1
3.91E-27	0.45292453	0.38	0.165	9.47E-23	6	Itga4
3.95E-27	0.34455947	0.946	0.776	9.58E-23	6	Gpnmb
4.01E-27	0.44134085	0.837	0.65	9.72E-23	6	Ctsa
4.26E-27	0.52690636	0.531	0.296	1.03E-22	6	Fam105a
6.18E-27	0.71364742	0.55	0.353	1.50E-22	6	St3gal5
6.39E-27	0.62905102	0.49	0.275	1.55E-22	6	Lfng
7.68E-27	0.38041795	0.669	0.38	1.86E-22	6	Fyb
1.35E-26	0.68333482	0.531	0.331	3.27E-22	6	Ccdc71l
1.37E-26	0.52714218	0.804	0.612	3.32E-22	6	Hmox1
1.57E-26	0.50685285	0.699	0.509	3.80E-22	6	Usp25
1.63E-26	0.53143231	0.515	0.289	3.96E-22	6	Dock8
1.67E-26	0.47319718	0.51	0.265	4.06E-22	6	Ncf1
1.69E-26	0.48966584	0.529	0.276	4.11E-22	6	Gmfg
2.09E-26	0.59120959	0.622	0.411	5.07E-22	6	Npc1
2.18E-26	0.55182565	0.382	0.171	5.30E-22	6	Trim30a
2.41E-26	0.52102942	0.795	0.595	5.85E-22	6	Degs1

3.27E-26	0.51373688	0.592	0.344	7.94E-22	6	Vsir
4.16E-26	0.46737835	0.485	0.247	1.01E-21	6	Lmo2
4.97E-26	0.42893183	0.846	0.662	1.21E-21	6	Ptbp3
7.59E-26	0.48064627	0.727	0.529	1.84E-21	6	Ifngr1
1.07E-25	0.43681924	0.902	0.705	2.59E-21	6	Srsf2
1.30E-25	0.43421546	0.832	0.623	3.15E-21	6	Slc40a1
1.54E-25	0.38007523	0.406	0.185	3.74E-21	6	Dock2
1.82E-25	0.45338787	0.855	0.622	4.42E-21	6	Selenop
2.03E-25	0.48018468	0.378	0.169	4.93E-21	6	Ms4a6b
2.21E-25	0.50197792	0.583	0.344	5.37E-21	6	Arrb2
3.05E-25	0.54024393	0.739	0.529	7.38E-21	6	Osbpl8
3.09E-25	0.40907048	0.536	0.286	7.49E-21	6	Ptpn6
3.32E-25	0.37359959	0.97	0.816	8.06E-21	6	Sat1
3.35E-25	0.37962241	0.93	0.76	8.12E-21	6	Hnrnpf
3.36E-25	0.54880406	0.739	0.57	8.16E-21	6	Mfsd1
3.85E-25	0.51876238	0.643	0.414	9.34E-21	6	Celf2
4.50E-25	0.39046517	0.606	0.333	1.09E-20	6	Clec4d
5.25E-25	0.3867557	0.867	0.679	1.27E-20	6	Mcl1
5.29E-25	0.43234549	0.876	0.661	1.28E-20	6	Btg1
7.58E-25	0.41451534	0.329	0.139	1.84E-20	6	Ebi3
8.15E-25	0.53322089	0.629	0.395	1.97E-20	6	Rgs2
8.42E-25	0.53494172	0.704	0.52	2.04E-20	6	Idh1

1.01E-24	0.46478206	0.655	0.391	2.45E-20	6	Alox5ap
1.22E-24	0.38683608	0.427	0.202	2.95E-20	6	Lair1
1.42E-24	0.5370934	0.494	0.285	3.45E-20	6	Map3k1
1.48E-24	0.54133036	0.639	0.432	3.58E-20	6	Wipf1
1.49E-24	0.45814118	0.681	0.422	3.61E-20	6	Cd300ld
2.39E-24	0.51629305	0.692	0.518	5.79E-20	6	Ly6e
3.02E-24	0.53422235	0.508	0.292	7.33E-20	6	Sesn1
3.66E-24	0.43359316	0.855	0.658	8.88E-20	6	Lamp2
3.97E-24	0.52271775	0.385	0.183	9.62E-20	6	St8sia4
4.39E-24	0.51693822	0.536	0.324	1.07E-19	6	Cmtm6
4.77E-24	0.43041682	0.501	0.269	1.16E-19	6	Ifi207
4.85E-24	0.47255811	0.79	0.552	1.18E-19	6	Cd53
7.14E-24	0.43018476	0.944	0.734	1.73E-19	6	Lrp1
7.96E-24	0.50294461	0.678	0.459	1.93E-19	6	Fos
8.97E-24	0.35133518	0.294	0.118	2.17E-19	6	Ncf4
1.21E-23	0.41844093	0.846	0.647	2.94E-19	6	Tapbp
1.44E-23	0.47472029	0.737	0.558	3.50E-19	6	Snx2
2.00E-23	0.43710451	0.865	0.657	4.86E-19	6	Atp1a1
2.24E-23	0.58309931	0.592	0.427	5.43E-19	6	Adam10
2.43E-23	0.43790863	0.438	0.226	5.90E-19	6	Tbxas1
2.58E-23	0.4881167	0.415	0.22	6.26E-19	6	Lpar6
3.75E-23	0.36418683	0.62	0.377	9.10E-19	6	Lst1

6.96E-23	0.4349048 1	0.688	0.48	1.69E-18	6	Dab2
8.75E-23	0.5422532 3	0.618	0.429	2.12E-18	6	Arl4c
1.21E-22	0.4485697 1	0.755	0.516	2.93E-18	6	Cd300a
1.47E-22	0.3910401 8	0.97	0.769	3.57E-18	6	Tmbim6
2.16E-22	0.3972230 4	0.918	0.792	5.23E-18	6	Taldo1
2.44E-22	0.4593562 6	0.641	0.432	5.91E-18	6	Cxcl16
2.45E-22	0.5155878 9	0.515	0.324	5.95E-18	6	Ncoa3
2.46E-22	0.4764735 5	0.503	0.289	5.97E-18	6	Rassf2
2.86E-22	0.5236746 7	0.674	0.507	6.93E-18	6	Itgb5
3.84E-22	0.3916084	0.811	0.597	9.30E-18	6	Cebpa
4.90E-22	0.5080988 7	0.545	0.352	1.19E-17	6	Man2b1
5.78E-22	0.5252896 7	0.611	0.451	1.40E-17	6	Top1
6.00E-22	0.5622257 4	0.585	0.416	1.46E-17	6	Gusb
8.21E-22	0.5175062 7	0.634	0.454	1.99E-17	6	Ehd4
1.00E-21	0.3638589 2	0.984	0.849	2.43E-17	6	Chchd2
1.15E-21	0.3688852 8	0.256	0.104	2.78E-17	6	Mtus1
1.18E-21	0.4449522 9	0.422	0.216	2.86E-17	6	Nckap1l
1.25E-21	0.5081897 6	0.646	0.474	3.02E-17	6	Gclm
1.59E-21	0.4723715 9	0.718	0.518	3.86E-17	6	Rab8b
1.72E-21	0.4268115 6	0.788	0.602	4.16E-17	6	Soat1
2.22E-21	0.4362041 7	0.744	0.567	5.38E-17	6	Man2a1
2.52E-21	0.6531592 9	0.429	0.267	6.12E-17	6	Nrp1

3.33E-21	0.53353039	0.618	0.454	8.07E-17	6	Rnf13
3.34E-21	0.4129223	0.382	0.193	8.09E-17	6	Frmd4b
3.76E-21	0.4272309	0.769	0.593	9.12E-17	6	Rtn3
4.00E-21	0.42497758	0.434	0.225	9.69E-17	6	Lpxn
4.40E-21	0.50578482	0.527	0.338	1.07E-16	6	Fnbp1
5.55E-21	0.35771728	0.862	0.717	1.35E-16	6	Serp1
5.74E-21	0.50211584	0.506	0.316	1.39E-16	6	BC037034
6.42E-21	0.44680699	0.317	0.147	1.56E-16	6	Hhex
6.58E-21	0.40236905	0.357	0.171	1.60E-16	6	Mertk
8.49E-21	0.4221824	0.765	0.585	2.06E-16	6	Cat
1.02E-20	0.53368853	0.499	0.32	2.48E-16	6	Notch1
1.02E-20	0.34259747	0.706	0.458	2.48E-16	6	Fermt3
1.20E-20	0.43108621	0.345	0.167	2.91E-16	6	Slc16a10
1.63E-20	0.36695774	0.783	0.566	3.95E-16	6	Tpd52
2.33E-20	0.38275941	0.604	0.369	5.65E-16	6	Ccl6
3.13E-20	0.41107162	0.45	0.243	7.59E-16	6	Pld4
3.17E-20	0.43498352	0.483	0.282	7.69E-16	6	Cd48
3.41E-20	0.45576137	0.688	0.535	8.28E-16	6	G3bp2
3.92E-20	0.3560821	0.448	0.241	9.50E-16	6	Pirb
3.99E-20	0.48722946	0.443	0.252	9.68E-16	6	Ptpre
5.55E-20	0.40671353	0.697	0.527	1.34E-15	6	Atp6ap1
5.95E-20	0.36438046	0.417	0.216	1.44E-15	6	Hpgds
6.53E-20	0.44409025	0.793	0.605	1.58E-15	6	Zfp36l1

6.58E-20	0.46145529	0.697	0.552	1.60E-15	6	Sypl
7.35E-20	0.32918263	0.55	0.322	1.78E-15	6	Cd52
7.37E-20	0.42836578	0.448	0.244	1.79E-15	6	Cd300lb
1.11E-19	0.41975524	0.448	0.249	2.70E-15	6	Sema4d
1.25E-19	0.36022089	0.261	0.107	3.04E-15	6	Mmp8
1.48E-19	0.5087028	0.79	0.609	3.59E-15	6	Junb
1.60E-19	0.45156232	0.716	0.519	3.88E-15	6	Itgav
1.63E-19	0.35500602	0.839	0.668	3.94E-15	6	Ap2s1
2.31E-19	0.40755873	0.536	0.329	5.60E-15	6	Cd300c2
3.31E-19	0.37202373	0.35	0.169	8.03E-15	6	Pou2f2
3.94E-19	0.4398239	0.655	0.492	9.55E-15	6	2610507B11Rik
4.17E-19	0.31995551	0.287	0.127	1.01E-14	6	Sorl1
4.37E-19	0.51169407	0.599	0.428	1.06E-14	6	Plxnd1
5.48E-19	0.316606	0.907	0.746	1.33E-14	6	Ddx5
5.90E-19	0.35907225	0.408	0.22	1.43E-14	6	Hck
6.42E-19	0.35287046	0.828	0.66	1.56E-14	6	Cst3
6.92E-19	0.37697589	0.38	0.201	1.68E-14	6	Mndal
7.58E-19	0.38112154	0.678	0.502	1.84E-14	6	Sgk1
7.69E-19	0.37040517	0.713	0.549	1.86E-14	6	Rhog
8.83E-19	0.43300941	0.625	0.464	2.14E-14	6	Ogt
1.07E-18	0.39127066	0.371	0.189	2.59E-14	6	Tlr13
1.11E-18	0.43116659	0.634	0.473	2.68E-14	6	Atp6ap2

1.61E-18	0.5855795 5	0.517	0.369	3.90E-14	6	Skil
1.96E-18	0.3252316 5	0.937	0.786	4.75E-14	6	Actr2
2.75E-18	0.4568418 2	0.573	0.372	6.68E-14	6	Dnase2a
4.19E-18	0.2649552 8	0.73	0.506	1.02E-13	6	Neat1
5.49E-18	0.3828523 5	0.385	0.206	1.33E-13	6	Cd200r1
5.68E-18	0.3731691 6	0.301	0.144	1.38E-13	6	Slfn5
6.12E-18	0.3018610 5	0.862	0.696	1.48E-13	6	Snx3
6.74E-18	0.2974684 5	0.417	0.224	1.64E-13	6	AI662270
6.84E-18	0.3704490 2	0.639	0.472	1.66E-13	6	Myl12b
7.12E-18	0.4233009 5	0.431	0.247	1.73E-13	6	Fgd4
7.17E-18	0.3694810 5	0.569	0.37	1.74E-13	6	Arhgap19
7.99E-18	0.4296001 6	0.552	0.393	1.94E-13	6	Sash1
8.13E-18	0.3300972	0.571	0.389	1.97E-13	6	Uap1l1
9.00E-18	0.3949060 4	0.408	0.224	2.18E-13	6	Clec12a
9.09E-18	0.3762817 6	0.604	0.417	2.20E-13	6	Cerk
1.28E-17	0.3660217 5	0.657	0.443	3.11E-13	6	Plek
1.48E-17	0.3993477 7	0.566	0.373	3.59E-13	6	Lilr4b
1.68E-17	0.4647791	0.429	0.27	4.07E-13	6	Rnf150
2.46E-17	0.3751495 7	0.807	0.651	5.96E-13	6	Irf2bp2
2.65E-17	0.2939917 4	0.802	0.594	6.42E-13	6	Ucp2
3.16E-17	0.4133409 7	0.324	0.165	7.67E-13	6	Iqgap2
4.67E-17	0.3124514 6	0.312	0.154	1.13E-12	6	Neurl3

4.81E-17	0.2829919<br>7	0.944	0.856	1.17E-12	6	Arpc2
5.98E-17	0.4259780<br>8	0.641	0.493	1.45E-12	6	Vwa5a
6.71E-17	0.3671906<br>9	0.676	0.519	1.63E-12	6	Wsb1
8.68E-17	0.4251490<br>7	0.415	0.231	2.10E-12	6	Abca1
8.76E-17	0.2896309<br>6	0.953	0.812	2.12E-12	6	Arpc5
8.79E-17	0.2565968<br>7	0.869	0.724	2.13E-12	6	Gdi2
9.33E-17	0.3278290<br>7	0.31	0.154	2.26E-12	6	Adap1
9.71E-17	0.4264950<br>5	0.441	0.282	2.35E-12	6	Ogfrl1
1.11E-16	0.4891719<br>6	0.601	0.464	2.68E-12	6	Sgpl1
1.14E-16	0.3635978<br>7	0.811	0.627	2.76E-12	6	Cd44
1.19E-16	0.3015471<br>5	0.531	0.322	2.90E-12	6	Hcls1
1.26E-16	0.2561477	0.695	0.474	3.05E-12	6	Clec7a
1.34E-16	0.3247236<br>7	0.457	0.27	3.24E-12	6	mt-Tw
1.57E-16	0.2581275<br>9	0.522	0.312	3.82E-12	6	Spi1
1.93E-16	0.3070500<br>5	0.935	0.783	4.67E-12	6	Npc2
1.96E-16	0.3005710<br>7	0.312	0.164	4.74E-12	6	Egfl7
1.98E-16	0.4509043<br>4	0.506	0.34	4.79E-12	6	Apbb1ip
2.00E-16	0.3598949<br>9	0.431	0.244	4.85E-12	6	Entpd1
2.00E-16	0.4014708<br>7	0.676	0.536	4.86E-12	6	Gna12
2.35E-16	0.3359646<br>6	0.678	0.536	5.71E-12	6	Pum2
2.42E-16	0.3452423<br>8	0.315	0.16	5.88E-12	6	Akr1c13
2.51E-16	0.3990610<br>5	0.58	0.412	6.09E-12	6	Hebp1

3.32E-16	0.3592343<br>8	0.413	0.235	8.06E-12	6	P2ry6
3.50E-16	0.3519788<br>3	0.713	0.583	8.48E-12	6	Wdr26
3.50E-16	0.3178469<br>8	0.872	0.712	8.49E-12	6	Ckb
5.50E-16	0.4139851<br>8	0.599	0.447	1.33E-11	6	Rnf130
5.92E-16	0.3123892<br>3	0.543	0.36	1.44E-11	6	Renbp
8.85E-16	0.2761822<br>8	0.478	0.281	2.15E-11	6	Irf8
9.50E-16	0.4206267<br>8	0.622	0.487	2.30E-11	6	Erp29
9.62E-16	0.3210335<br>4	0.417	0.239	2.33E-11	6	Slc11a1
9.83E-16	0.4124364	0.275	0.137	2.38E-11	6	Evi2
1.24E-15	0.4235931<br>1	0.543	0.392	3.00E-11	6	Acp2
1.41E-15	0.5021776	0.499	0.375	3.41E-11	6	Zdhhc20
1.53E-15	0.4089720<br>8	0.625	0.479	3.71E-11	6	Por
1.59E-15	0.2873202<br>7	0.83	0.669	3.84E-11	6	Ddx3x
1.71E-15	0.3633611<br>3	0.585	0.431	4.14E-11	6	Tcirg1
1.90E-15	0.3124815<br>6	0.282	0.139	4.60E-11	6	Ccdc88b
2.49E-15	0.3519838<br>5	0.664	0.518	6.04E-11	6	Samhd1
2.53E-15	0.4680225<br>8	0.392	0.251	6.15E-11	6	Etv5
2.63E-15	0.2522725<br>7	0.981	0.877	6.38E-11	6	Fabp5
2.67E-15	0.5082309<br>1	0.508	0.372	6.48E-11	6	Ggh
2.75E-15	0.2925053<br>7	0.301	0.15	6.66E-11	6	Coro2a
3.43E-15	0.3865758<br>7	0.59	0.464	8.31E-11	6	Ifngr2
3.88E-15	0.3204147<br>8	0.795	0.677	9.42E-11	6	Adipor1

5.17E-15	0.31585402	0.263	0.128	1.25E-10	6	Aatk
5.31E-15	0.33832885	0.643	0.5	1.29E-10	6	Rassf3
5.86E-15	0.30624308	0.259	0.123	1.42E-10	6	Tnfrsf13b
6.41E-15	0.33180144	0.751	0.591	1.55E-10	6	Slc6a6
6.55E-15	0.35121185	0.643	0.463	1.59E-10	6	Gpx3
7.31E-15	0.42817674	0.441	0.289	1.77E-10	6	Stxbp2
7.61E-15	0.48877829	0.462	0.311	1.85E-10	6	Orai1
9.65E-15	0.47165869	0.345	0.209	2.34E-10	6	Dock4
1.08E-14	0.37039495	0.573	0.44	2.62E-10	6	Tfe3
1.20E-14	0.37845425	0.772	0.598	2.92E-10	6	Itm2c
1.47E-14	0.31117476	0.744	0.603	3.57E-10	6	Edem1
1.71E-14	0.27623188	0.524	0.333	4.16E-10	6	Cd68
1.90E-14	0.3564742	0.69	0.573	4.60E-10	6	Pgd
2.03E-14	0.32784013	0.569	0.41	4.92E-10	6	Blvrb
2.13E-14	0.29128452	0.772	0.626	5.18E-10	6	Fxyd5
2.36E-14	0.27660809	0.256	0.126	5.72E-10	6	B3gnt8
2.72E-14	0.377855	0.592	0.456	6.58E-10	6	Mdfic
2.85E-14	0.4691396	0.343	0.206	6.92E-10	6	Irf9
2.96E-14	0.40006767	0.427	0.266	7.18E-10	6	Arhgap18
2.98E-14	0.39921835	0.352	0.212	7.22E-10	6	Trib1
3.21E-14	0.35154319	0.592	0.463	7.78E-10	6	Epn1
3.27E-14	0.53038593	0.399	0.269	7.92E-10	6	Wdfy2
3.79E-14	0.39083448	0.599	0.446	9.20E-10	6	Kctd12

3.96E-14	0.30418284	0.499	0.327	9.60E-10	6	Irf5
4.68E-14	0.34153014	0.655	0.532	1.13E-09	6	Vps4b
4.81E-14	0.36177677	0.599	0.469	1.17E-09	6	Clk1
4.88E-14	0.40452057	0.594	0.453	1.18E-09	6	Fam174a
5.96E-14	0.33712737	0.746	0.601	1.44E-09	6	Reep5
6.00E-14	0.30218568	0.802	0.633	1.45E-09	6	Ddx17
6.27E-14	0.32729481	0.287	0.148	1.52E-09	6	Tnfrsf11a
7.88E-14	0.300015	0.639	0.502	1.91E-09	6	Gns
8.37E-14	0.29501735	0.38	0.217	2.03E-09	6	Lrrc25
9.82E-14	0.27536571	0.716	0.597	2.38E-09	6	Cmpk1
1.18E-13	0.39938229	0.49	0.342	2.86E-09	6	Alcam
1.22E-13	0.42791224	0.464	0.324	2.95E-09	6	Mgst1
1.42E-13	0.32208248	0.639	0.489	3.45E-09	6	Macf1
1.44E-13	0.30220912	0.683	0.562	3.49E-09	6	Rab11a
1.75E-13	0.36212312	0.413	0.276	4.23E-09	6	Hacd4
1.90E-13	0.29780064	0.648	0.512	4.61E-09	6	Lnpep
1.96E-13	0.28373162	0.259	0.129	4.75E-09	6	Slc46a3
2.06E-13	0.32365481	0.329	0.185	5.00E-09	6	Il6ra
2.99E-13	0.30383889	0.678	0.547	7.25E-09	6	Eid1
3.29E-13	0.33939121	0.368	0.228	7.97E-09	6	Gmip
4.49E-13	0.26672789	0.657	0.469	1.09E-08	6	Trf
4.99E-13	0.33508785	0.441	0.284	1.21E-08	6	Pik3cd

5.59E-13	0.32597465	0.394	0.242	1.36E-08	6	Slc37a2
5.60E-13	0.37544662	0.41	0.279	1.36E-08	6	Tet3
5.62E-13	0.50475618	0.497	0.376	1.36E-08	6	B4galt6
6.25E-13	0.31477757	0.55	0.401	1.52E-08	6	Arap1
6.26E-13	0.43015242	0.359	0.228	1.52E-08	6	Zfp385a
6.89E-13	0.36456879	0.573	0.457	1.67E-08	6	Tpp1
8.15E-13	0.32869141	0.301	0.162	1.98E-08	6	Hcst
1.06E-12	0.36203972	0.601	0.477	2.58E-08	6	Ldlrap1
1.07E-12	0.41323731	0.41	0.282	2.60E-08	6	Emb
1.11E-12	0.30589554	0.648	0.51	2.69E-08	6	Sdf4
1.18E-12	0.25478604	0.753	0.613	2.86E-08	6	Hexa
1.21E-12	0.2707779	0.723	0.595	2.94E-08	6	Cd164
1.43E-12	0.38202221	0.576	0.461	3.47E-08	6	Asah1
1.85E-12	0.26407769	0.716	0.597	4.50E-08	6	Ppp3r1
2.30E-12	0.37538312	0.329	0.197	5.59E-08	6	Pygl
2.59E-12	0.30206556	0.462	0.337	6.27E-08	6	Tcf7l2
2.59E-12	0.30199708	0.415	0.278	6.28E-08	6	Fam111a
2.97E-12	0.38817383	0.317	0.19	7.20E-08	6	Camk1d
3.54E-12	0.27620015	0.765	0.584	8.57E-08	6	Tm9sf3
3.58E-12	0.27830711	0.709	0.591	8.68E-08	6	Atp6v1f
3.60E-12	0.29715874	0.718	0.588	8.72E-08	6	Emc10
4.95E-12	0.31270075	0.417	0.264	1.20E-07	6	Rab3il1

5.07E-12	0.31717143	0.557	0.448	1.23E-07	6	Tmed7
5.86E-12	0.3458842	0.524	0.416	1.42E-07	6	Ehmt2
6.67E-12	0.32468807	0.338	0.205	1.62E-07	6	Tec
7.36E-12	0.25614714	0.459	0.301	1.78E-07	6	Ptk2b
8.46E-12	0.33079294	0.452	0.313	2.05E-07	6	Dck
8.49E-12	0.39265459	0.569	0.466	2.06E-07	6	Xiap
9.06E-12	0.29364742	0.695	0.579	2.20E-07	6	Cd47
9.08E-12	0.25634462	0.282	0.153	2.20E-07	6	Atp8a1
9.43E-12	0.2750583	0.415	0.257	2.29E-07	6	Prkcb
1.02E-11	0.46199307	0.413	0.285	2.48E-07	6	Stat1
1.18E-11	0.28543867	0.692	0.584	2.86E-07	6	Tab2
1.24E-11	0.39357063	0.343	0.218	3.02E-07	6	Man1c1
1.45E-11	0.39508481	0.527	0.42	3.52E-07	6	Eps15
1.51E-11	0.47046052	0.466	0.366	3.67E-07	6	Eea1
1.55E-11	0.40893379	0.508	0.404	3.75E-07	6	Nipa2
1.57E-11	0.29008509	0.59	0.476	3.80E-07	6	Tsc22d4
1.66E-11	0.28777961	0.627	0.512	4.01E-07	6	Myo5a
1.70E-11	0.38324588	0.494	0.373	4.12E-07	6	Kdm7a
1.92E-11	0.40969164	0.452	0.332	4.66E-07	6	Znrf2
2.07E-11	0.34966478	0.543	0.415	5.01E-07	6	Pdlim4
2.10E-11	0.38168236	0.501	0.395	5.10E-07	6	Ep300
2.23E-11	0.37943089	0.531	0.429	5.42E-07	6	Tor1aip1

2.24E-11	0.2636635 3	0.832	0.658	5.43E-07	6	Snrnp70
2.33E-11	0.3018191 3	0.585	0.468	5.64E-07	6	Atxn2l
2.44E-11	0.4048636 7	0.627	0.533	5.92E-07	6	Selenot
3.18E-11	0.3443668 6	0.522	0.39	7.72E-07	6	Tmem106a
3.19E-11	0.3024118 2	0.536	0.432	7.73E-07	6	Numa1
3.22E-11	0.3589937 1	0.259	0.147	7.81E-07	6	Dmxl2
3.30E-11	0.2791845 3	0.62	0.514	8.00E-07	6	Atp1b3
3.96E-11	0.3167543 8	0.569	0.457	9.60E-07	6	Stx7
4.07E-11	0.2556058 1	0.527	0.374	9.88E-07	6	Lyn
4.41E-11	0.3814617	0.452	0.326	1.07E-06	6	Etv3
4.52E-11	0.2970378 7	0.648	0.527	1.10E-06	6	Prkcd
4.59E-11	0.2545971 5	0.746	0.628	1.11E-06	6	Ppp1r18
4.71E-11	0.2949383 6	0.613	0.492	1.14E-06	6	Zfp703
5.65E-11	0.2693732 4	0.336	0.201	1.37E-06	6	Srgn
5.72E-11	0.2726112 4	0.909	0.706	1.39E-06	6	Canx
6.45E-11	0.3139288	0.583	0.471	1.56E-06	6	Slc48a1
6.45E-11	0.2526503 1	0.319	0.188	1.56E-06	6	Gas2l3
6.57E-11	0.2939683 3	0.524	0.383	1.59E-06	6	Tgm2
7.31E-11	0.2727344 4	0.585	0.462	1.77E-06	6	Kansl2
8.54E-11	0.3024599 6	0.702	0.54	2.07E-06	6	Dync1h1
1.03E-10	0.3724663 5	0.277	0.167	2.50E-06	6	Slc17a5
1.14E-10	0.3306868 3	0.527	0.419	2.77E-06	6	Frg1
1.16E-10	0.2945126	0.653	0.541	2.81E-06	6	Txnrd1

1.19E-10	0.33274903	0.326	0.206	2.90E-06	6	Pnpla7
1.31E-10	0.294821	0.469	0.332	3.18E-06	6	Rab7b
1.35E-10	0.33016229	0.28	0.159	3.26E-06	6	Gpr157
1.50E-10	0.32137943	0.576	0.477	3.63E-06	6	Syngr2
1.52E-10	0.43285957	0.448	0.34	3.70E-06	6	Lmbrd1
1.98E-10	0.36050127	0.459	0.351	4.80E-06	6	Fam129a
2.00E-10	0.25142362	0.298	0.174	4.86E-06	6	Cxcr4
2.10E-10	0.39468957	0.361	0.232	5.10E-06	6	Gatm
2.27E-10	0.52974506	0.406	0.311	5.52E-06	6	Frrs1
2.42E-10	0.25776731	0.263	0.148	5.86E-06	6	Hpse
2.76E-10	0.26007125	0.641	0.53	6.69E-06	6	Dnajb6
2.90E-10	0.30798491	0.464	0.318	7.03E-06	6	Ccr1
3.19E-10	0.3807147	0.464	0.357	7.74E-06	6	Ifnar2
3.31E-10	0.38516345	0.387	0.26	8.02E-06	6	Plxnc1
3.71E-10	0.36448294	0.445	0.343	9.00E-06	6	Lgals8
3.91E-10	0.33297847	0.336	0.216	9.48E-06	6	Sgk3
3.97E-10	0.34171314	0.34	0.225	9.63E-06	6	Cyth1
4.96E-10	0.35776587	0.741	0.604	1.20E-05	6	Dusp1
5.00E-10	0.3800167	0.476	0.38	1.21E-05	6	Bcap31
5.42E-10	0.27226836	0.357	0.235	1.31E-05	6	Trim12c
5.89E-10	0.45790326	0.41	0.303	1.43E-05	6	Cpeb2
6.40E-10	0.39119087	0.531	0.447	1.55E-05	6	Dse
6.62E-10	0.2622082	0.622	0.523	1.61E-05	6	Copz1

7.20E-10	0.32527499	0.28	0.168	1.75E-05	6	Slc25a45
1.10E-09	0.37854738	0.485	0.393	2.66E-05	6	Scamp2
1.12E-09	0.38945858	0.31	0.192	2.71E-05	6	Ccl4
1.17E-09	0.25186256	0.555	0.456	2.85E-05	6	Grina
1.20E-09	0.2909148	0.266	0.16	2.92E-05	6	Ncapg2
1.27E-09	0.35316621	0.354	0.241	3.07E-05	6	Sgsh
1.51E-09	0.48060616	0.326	0.229	3.65E-05	6	1810026B05Rik
1.53E-09	0.4569099	0.406	0.308	3.72E-05	6	Galc
1.59E-09	0.39344883	0.438	0.342	3.87E-05	6	Pnp
1.64E-09	0.43505814	0.415	0.309	3.97E-05	6	Mef2c
1.73E-09	0.39480453	0.308	0.204	4.20E-05	6	D1Ertd622e
1.78E-09	0.3481899	0.256	0.154	4.32E-05	6	Gcnt1
1.80E-09	0.37841589	0.315	0.212	4.36E-05	6	Rgl2
1.87E-09	0.2990803	0.571	0.469	4.53E-05	6	Hsd17b12
1.92E-09	0.32150021	0.291	0.186	4.65E-05	6	Epb41
1.97E-09	0.25967507	0.611	0.513	4.77E-05	6	Tm9sf2
2.02E-09	0.32383593	0.524	0.431	4.90E-05	6	Abl1
2.25E-09	0.31182811	0.587	0.506	5.44E-05	6	Sptssa
2.53E-09	0.34346267	0.289	0.182	6.13E-05	6	Pctp
3.00E-09	0.3657319	0.443	0.351	7.26E-05	6	Ddrgk1
3.28E-09	0.34402676	0.319	0.219	7.94E-05	6	Sipa1
3.52E-09	0.27257015	0.627	0.532	8.53E-05	6	Ubl3
5.74E-09	0.27307056	0.254	0.152	0.0001392	6	Tifa

5.94E-09	0.36136452	0.371	0.268	0.00014402	6	Ifi204
6.42E-09	0.28769429	0.487	0.399	0.00015563	6	Mia2
6.81E-09	0.35317968	0.322	0.219	0.00016513	6	Tmem86a
7.06E-09	0.36516249	0.324	0.225	0.00017127	6	Tgfbr1
7.23E-09	0.25497729	0.343	0.239	0.00017539	6	Psmb8
8.55E-09	0.308452	0.51	0.415	0.0002074	6	Prcp
8.63E-09	0.28298746	0.329	0.215	0.00020933	6	Txnip
9.26E-09	0.28763034	0.501	0.411	0.00022463	6	Git2
9.40E-09	0.29739141	0.499	0.41	0.00022797	6	Smarcd2
1.02E-08	0.28802177	0.49	0.402	0.00024651	6	Erbin
1.03E-08	0.42127834	0.34	0.247	0.0002494	6	Tspan14
1.08E-08	0.33778673	0.417	0.314	0.00026177	6	Cln8
1.10E-08	0.31765547	0.543	0.457	0.00026633	6	Arhgap17
1.37E-08	0.4027567	0.445	0.363	0.0003322	6	Pkig
1.41E-08	0.32201594	0.513	0.412	0.00034296	6	Cdc42se2
1.54E-08	0.33000468	0.389	0.283	0.00037332	6	Ptprj
1.66E-08	0.27962074	0.508	0.415	0.00040201	6	Stk17b
1.71E-08	0.34519683	0.387	0.292	0.00041581	6	Zfp395
1.76E-08	0.32151139	0.564	0.446	0.00042638	6	Vegfa
2.08E-08	0.31886448	0.259	0.161	0.0005054	6	H2-DMb1
2.34E-08	0.44157904	0.436	0.357	0.00056751	6	S1pr2
2.35E-08	0.25187245	0.354	0.239	0.00056946	6	Cln3

2.80E-08	0.33306928	0.503	0.419	0.00067982	6	Fcho2
2.89E-08	0.39274402	0.49	0.415	0.00070139	6	Swap70
3.35E-08	0.38822789	0.282	0.19	0.00081318	6	Retreg1
3.56E-08	0.28070923	0.571	0.478	0.00086224	6	Cpd
3.57E-08	0.3835229	0.366	0.275	0.00086669	6	Fkbp15
3.87E-08	0.30651142	0.548	0.449	0.00093725	6	Hpcal1
4.16E-08	0.2906804	0.378	0.279	0.00100879	6	Dram2
4.21E-08	0.33576501	0.503	0.427	0.00102115	6	Smim7
4.82E-08	0.3115595	0.345	0.244	0.00116981	6	Evi2a
4.87E-08	0.36604109	0.331	0.238	0.00118164	6	Dennd1a
4.91E-08	0.39552234	0.331	0.245	0.00119016	6	Rftn1
4.97E-08	0.26226691	0.49	0.385	0.00120477	6	Pip4k2a
5.34E-08	0.25266715	0.541	0.452	0.00129369	6	Cyb5a
9.25E-08	0.37114068	0.445	0.366	0.0022438	6	Aftph
1.07E-07	0.31996338	0.392	0.305	0.00260634	6	Rin2
1.18E-07	0.26052204	0.315	0.217	0.00286025	6	Zfp710
1.28E-07	0.29493643	0.375	0.284	0.0031035	6	Rnf115
1.54E-07	0.27862678	0.429	0.35	0.00373597	6	Snhg9
1.79E-07	0.31626009	0.401	0.318	0.00434976	6	Rnf44
1.94E-07	0.27566009	0.45	0.359	0.00470605	6	Ypel3
2.42E-07	0.28338368	0.368	0.273	0.0058608	6	Rp2

2.46E-07	0.31227865	0.303	0.214	0.0059627	6	Zfp217
2.55E-07	0.39975458	0.41	0.344	0.00618062	6	Dnajb14
3.35E-07	0.31396949	0.452	0.379	0.00811149	6	Ids
4.00E-07	0.27206528	0.45	0.383	0.0097001	6	Paip1
4.98E-07	0.29159195	0.308	0.223	0.01206996	6	Slc12a6
4.99E-07	0.33114723	0.333	0.247	0.01210504	6	Man1a
5.25E-07	0.31660533	0.394	0.298	0.01272635	6	Gnpda1
5.40E-07	0.39089917	0.322	0.237	0.01310337	6	Plekhm1
5.79E-07	0.2683143	0.378	0.293	0.01404258	6	Card19
6.07E-07	0.38749026	0.42	0.345	0.01472152	6	Cyp51
6.10E-07	0.32837632	0.256	0.172	0.01477919	6	Mylip
6.16E-07	0.35147318	0.448	0.383	0.01492546	6	Mef2d
6.68E-07	0.3387587	0.275	0.192	0.01618524	6	Atp13a2
6.73E-07	0.32442583	0.373	0.291	0.01631051	6	Tbc1d14
6.91E-07	0.25999175	0.494	0.421	0.01674817	6	Glmp
6.95E-07	0.30587704	0.52	0.466	0.0168453	6	Wapl
7.33E-07	0.28026424	0.49	0.422	0.01778422	6	Amfr
7.75E-07	0.29259602	0.394	0.303	0.01878067	6	Cln6
7.77E-07	0.28251967	0.336	0.256	0.01882862	6	Cttnbp2nl
7.78E-07	0.36453675	0.343	0.268	0.0188544	6	Rufy3
7.94E-07	0.32465673	0.347	0.267	0.01924546	6	Ccdc115

8.38E-07	0.3109498 5	0.399	0.322	0.0203077 3	6	Retreg2
1.00E-06	0.3238710 6	0.415	0.352	0.0242639 6	6	Tmx3
1.02E-06	0.4004141 7	0.361	0.295	0.0246439 3	6	Ifnar1
1.39E-06	0.3328719 3	0.452	0.387	0.0337781 5	6	Atp6v1h
1.54E-06	0.2562104 4	0.448	0.387	0.0373253 5	6	Zcchc6
1.70E-06	0.3061337 8	0.394	0.325	0.0413004 9	6	Arhgef1
1.84E-06	0.3427333 4	0.366	0.297	0.0445404 8	6	Akap13
1.99E-06	0.2636347 4	0.476	0.405	0.0483252 5	6	Camk1
2.37E-06	0.4236879 1	0.431	0.371	0.0573728 2	6	Hgsnat
2.48E-06	0.3561502 5	0.368	0.298	0.0600676 3	6	Lpin2
2.50E-06	0.285953	0.464	0.413	0.0605472 1	6	Ppp4c
2.55E-06	0.3490067 7	0.403	0.334	0.0619212 2	6	Msmo1
2.57E-06	0.2791647 2	0.434	0.375	0.0623414 6	6	Tgoln1
2.84E-06	0.2794554	0.494	0.437	0.0688651 4	6	Ankrd13a
3.13E-06	0.2670890 7	0.287	0.204	0.0758330 9	6	Ppm1h
3.32E-06	0.301704	0.343	0.269	0.0804944 8	6	Synj1
3.48E-06	0.3266291 9	0.457	0.4	0.0844654 9	6	Cmas
3.68E-06	0.2672564 9	0.499	0.439	0.0891414 6	6	Wdr83os
4.06E-06	0.2725326	0.373	0.302	0.0983256 8	6	Hk2
4.42E-06	0.3186207	0.387	0.328	0.1071652 6	6	Zfp516
4.63E-06	0.2577133 8	0.513	0.463	0.1121472 7	6	Selenok

4.75E-06	0.27236454	0.49	0.419	0.11527066	6	Rps2-ps10
6.19E-06	0.25525458	0.431	0.372	0.15020871	6	Snap23
6.73E-06	0.31603176	0.357	0.284	0.16314415	6	Ggta1
6.78E-06	0.35440946	0.408	0.351	0.1643035	6	Slc31a1
7.20E-06	0.25905055	0.508	0.452	0.17458743	6	Irak1
8.04E-06	0.26407085	0.457	0.395	0.19486713	6	Ptpn1
8.20E-06	0.28416181	0.354	0.289	0.19874466	6	Ikbkb
1.01E-05	0.36180637	0.31	0.244	0.24469167	6	Nabp1
1.10E-05	0.34473543	0.347	0.29	0.26771301	6	Pias1
1.16E-05	0.26592425	0.441	0.385	0.28213948	6	Dcaf12
1.39E-05	0.30481324	0.352	0.285	0.33795378	6	Slc29a3
1.70E-05	0.25076837	0.382	0.315	0.41233954	6	Bach1
1.86E-05	0.25864293	0.41	0.355	0.44996827	6	Sp3
2.13E-05	0.32332294	0.296	0.227	0.5158331	6	Fuca2
2.64E-05	0.33686073	0.354	0.301	0.6399147	6	Psenen
2.73E-05	0.26877114	0.396	0.349	0.66197472	6	Smap2
2.84E-05	0.28344876	0.436	0.391	0.68916621	6	Scamp3
3.03E-05	0.36557638	0.273	0.21	0.73420898	6	Hps3
3.57E-05	0.29389293	0.385	0.332	0.86631527	6	Tmem168
3.84E-05	0.32433688	0.287	0.224	0.9315634	6	Il17ra
3.98E-05	0.3953399	0.331	0.278	0.96545481	6	Scamp1

4.49E-05	0.31314112	0.399	0.35	1	6	Emc1
5.35E-05	0.25514603	0.471	0.418	1	6	Rab31
5.63E-05	0.3120339	0.394	0.334	1	6	Mcur1
8.46E-05	0.28456577	0.273	0.208	1	6	Ormdl1
8.70E-05	0.30312074	0.324	0.272	1	6	Sumf1
9.09E-05	0.31586177	0.298	0.242	1	6	Vps33a
0.00010412	0.3071146	0.338	0.293	1	6	Slc25a37
0.00011189	0.25796551	0.401	0.351	1	6	Pbx2
0.00011309	0.27096007	0.387	0.334	1	6	Trim25
0.00012386	0.32780053	0.277	0.221	1	6	Nfxl1
0.0001313	0.28569176	0.387	0.345	1	6	Nucb1
0.00013143	0.25183468	0.38	0.329	1	6	Itsn2
0.00014197	0.3651626	0.34	0.293	1	6	Hexim1
0.00014922	0.30496232	0.35	0.3	1	6	Glrx
0.00016494	0.30999115	0.308	0.26	1	6	Fbxo33
0.00018703	0.31052787	0.284	0.231	1	6	Snx30
0.00020843	0.26299441	0.399	0.36	1	6	Cdc26
0.0003535	0.26263456	0.382	0.345	1	6	Irf2bpl
0.00044237	0.31280005	0.408	0.371	1	6	Pank3
0.00053958	0.27997711	0.275	0.223	1	6	Mif4gd
0.00056509	0.30203828	0.256	0.208	1	6	Tgfbrap1
0.0006049	0.25335598	0.266	0.216	1	6	Xylt2

0.0006283 6	0.2574247 1	0.277	0.23	1	6	Hbp1
0.0007995 7	0.2951865 8	0.268	0.216	1	6	Gda
0.0009034 1	0.2540726	0.326	0.288	1	6	Cd302
0.0009054 5	0.3274687 5	0.326	0.29	1	6	Fhod1
0.0013766 4	0.2516104 5	0.31	0.27	1	6	Pikfyve
0.0019527 8	0.2778829 3	0.326	0.296	1	6	Yipf4
0.0023887 6	0.2804944 7	0.263	0.226	1	6	Taz
0.0030914 2	0.3083845 2	0.284	0.252	1	6	Tgfbr2
0.0033637 4	0.25383	0.399	0.38	1	6	Derl1
0.0036401 4	0.2697234 4	0.284	0.253	1	6	Fibp
0.0038157 8	0.2882554 8	0.308	0.277	1	6	Fam45a
0.003983	0.2723701	0.289	0.258	1	6	Zbtb44
0.0041766 1	0.2604756 8	0.317	0.287	1	6	Enc1
0.0058248	0.2516437 9	0.294	0.266	1	6	Tomm34
0.0075452 4	0.2730620 1	0.378	0.364	1	6	Med8
0.0075739 6	0.2816695 7	0.347	0.328	1	6	Dmac1
0.0085415 9	0.3639811 2	0.263	0.241	1	6	Jmjd1c
0	0.9892223 5	0.814	0.091	0	7	Ly75
0	0.9147957 8	0.679	0.058	0	7	Anln
0	0.7231407 4	0.526	0.026	0	7	Hmmr
0	0.7088724 5	0.56	0.03	0	7	Ckap2l
0	0.6730941 4	0.463	0.011	0	7	Dpep1

0	0.58970879	0.548	0.029	0	7	S1pr3
0	0.54169772	0.568	0.035	0	7	Pimreg
0	0.44361301	0.507	0.025	0	7	Kif15
0	0.43239996	0.49	0.02	0	7	Pbk
0	0.42602501	0.526	0.022	0	7	Bub1
0	0.35899919	0.432	0.014	0	7	Knl1
0	0.34932036	0.543	0.031	0	7	Syne2
0	0.33170405	0.548	0.035	0	7	Adam12
9.29E-307	0.32051167	0.443	0.017	2.25E-302	7	Kif4
2.25E-304	0.4895035	0.548	0.035	5.46E-300	7	Foxm1
4.24E-303	0.49366629	0.524	0.03	1.03E-298	7	Kif20a
7.53E-301	0.33300659	0.493	0.025	1.83E-296	7	Nuf2
7.59E-301	0.39192951	0.662	0.061	1.84E-296	7	Gjc1
9.73E-300	0.52876248	0.524	0.032	2.36E-295	7	Gpm6b
2.12E-299	0.81935735	0.925	0.138	5.14E-295	7	Gpr176
2.38E-299	0.53751294	0.571	0.04	5.78E-295	7	Id4
1.55E-294	0.33129493	0.44	0.019	3.75E-290	7	Melk
3.19E-294	0.45587722	0.399	0.013	7.74E-290	7	Aspm
1.34E-293	0.35244101	0.427	0.017	3.24E-289	7	Sgo2a
9.15E-292	0.38369592	0.429	0.017	2.22E-287	7	Kif2c
6.93E-290	0.34590864	0.443	0.02	1.68E-285	7	Clspn
1.61E-286	0.65211089	0.809	0.107	3.90E-282	7	Fosl1

4.49E-283	0.37057279	0.443	0.021	1.09E-278	7	Ect2
7.73E-281	0.53989544	0.812	0.109	1.87E-276	7	Nectin3
2.30E-280	0.29055232	0.427	0.019	5.56E-276	7	Dlgap5
1.03E-279	1.17036938	0.906	0.147	2.50E-275	7	Ebf1
7.61E-274	1.35462135	0.953	0.172	1.85E-269	7	Loxl1
1.60E-273	0.38409797	0.468	0.026	3.88E-269	7	Nek2
2.28E-272	0.25178173	0.407	0.017	5.52E-268	7	Ttk
4.13E-272	0.35030746	0.504	0.032	1.00E-267	7	Rad51
1.03E-271	0.79145951	0.637	0.065	2.50E-267	7	Diaph3
1.08E-271	0.41185207	0.44	0.022	2.61E-267	7	Sgo1
4.18E-271	0.48595565	0.535	0.039	1.01E-266	7	Cdca8
7.76E-271	1.30593707	0.942	0.167	1.88E-266	7	Phldb2
1.14E-270	0.45531444	0.604	0.054	2.77E-266	7	Spc24
1.17E-269	0.79386811	0.731	0.092	2.83E-265	7	Sorbs1
1.17E-269	1.20555576	0.92	0.159	2.84E-265	7	Pdgfrb
1.91E-268	0.94453273	0.886	0.141	4.63E-264	7	Nid1
2.32E-268	0.30502272	0.432	0.022	5.61E-264	7	Cit
1.77E-266	0.75543703	0.878	0.14	4.29E-262	7	Smtn
2.04E-266	0.54815166	0.806	0.114	4.95E-262	7	Bok
6.32E-266	0.77667711	0.609	0.059	1.53E-261	7	Smoc1
1.28E-265	0.53637219	0.781	0.105	3.09E-261	7	Adgra2

6.33E-265	0.44846286	0.515	0.038	1.54E-260	7	Svep1
9.79E-265	0.30224675	0.529	0.038	2.37E-260	7	Tk1
1.07E-264	0.299533	0.537	0.042	2.60E-260	7	Hmgb3
4.93E-263	0.31120612	0.305	0.006	1.20E-258	7	Ebf2
8.42E-263	0.85399153	0.936	0.159	2.04E-258	7	Lamb1
3.02E-262	0.76541569	0.85	0.129	7.33E-258	7	Zfhx4
1.28E-261	0.48733995	0.476	0.029	3.10E-257	7	Kif23
2.69E-260	0.53351272	0.449	0.026	6.53E-256	7	Plk1
4.06E-260	0.67932513	0.831	0.126	9.85E-256	7	Foxc1
3.48E-259	0.50141348	0.681	0.08	8.43E-255	7	Cobll1
1.67E-258	0.73753895	0.512	0.037	4.05E-254	7	Cenpe
2.19E-258	0.30496476	0.432	0.023	5.31E-254	7	Ncapg
3.10E-258	0.69173213	0.911	0.149	7.53E-254	7	Rhobtb3
5.45E-258	0.46150802	0.706	0.086	1.32E-253	7	Hmga1
2.07E-257	0.66691063	0.748	0.1	5.01E-253	7	H1fx
5.92E-257	1.08220146	0.701	0.083	1.44E-252	7	Pclaf
3.71E-256	0.78853852	0.709	0.09	9.00E-252	7	Fst
4.07E-256	0.48821001	0.72	0.089	9.86E-252	7	Foxc2
2.55E-255	1.19788653	0.576	0.053	6.19E-251	7	Mki67
8.96E-255	0.41539382	0.571	0.05	2.17E-250	7	C330027C09Rik
4.88E-254	0.53507615	0.607	0.06	1.18E-249	7	Ncapd2
8.61E-254	0.26411277	0.393	0.018	2.09E-249	7	Cdca2

2.14E-253	0.47227716	0.285	0.005	5.18E-249	7	Plin4
7.01E-253	0.42102225	0.548	0.048	1.70E-248	7	Pde3a
6.11E-252	0.48631428	0.839	0.128	1.48E-247	7	Arhgef17
3.64E-250	1.17113844	0.715	0.093	8.83E-246	7	Slc5a3
7.02E-250	0.85640326	0.856	0.138	1.70E-245	7	Wnt5a
1.06E-249	0.95462121	0.895	0.146	2.57E-245	7	Vcan
3.47E-249	1.01071175	0.637	0.069	8.42E-245	7	Prc1
4.38E-249	1.3509063	0.784	0.116	1.06E-244	7	Tnfrsf11b
7.72E-249	1.32644641	0.925	0.167	1.87E-244	7	Col6a2
1.71E-248	0.59679148	0.895	0.147	4.14E-244	7	Hacd1
4.15E-248	1.20019868	0.906	0.16	1.01E-243	7	Phlda1
7.30E-248	0.35458654	0.529	0.043	1.77E-243	7	Trip13
2.02E-247	0.88063888	0.587	0.059	4.89E-243	7	Rspo2
2.20E-247	0.56852872	0.881	0.141	5.33E-243	7	Prkca
1.36E-246	0.82583347	0.848	0.143	3.31E-242	7	Selenoh
4.41E-246	0.5046371	0.468	0.032	1.07E-241	7	Aurka
4.44E-246	0.92953657	0.62	0.069	1.08E-241	7	Tpx2
5.67E-246	0.68418886	0.704	0.091	1.37E-241	7	Hs6st2
7.22E-246	0.28665799	0.521	0.043	1.75E-241	7	Cenpq
2.44E-244	0.63451834	0.856	0.136	5.91E-240	7	Fads3
4.10E-243	0.31514922	0.277	0.005	9.95E-239	7	Aoc3
9.71E-243	0.35902157	0.501	0.039	2.35E-238	7	Kif22

3.01E-242	1.10923824	0.986	0.199	7.30E-238	7	Ehd2
5.66E-242	1.05819833	0.97	0.184	1.37E-237	7	Nfib
9.19E-242	0.64055661	0.521	0.044	2.23E-237	7	Cdc20
9.52E-242	0.42852168	0.745	0.105	2.31E-237	7	Rbpms2
1.32E-241	1.55262323	0.909	0.174	3.21E-237	7	Rrm2
1.71E-241	0.31794535	0.357	0.015	4.15E-237	7	Trpc6
1.97E-241	1.38712812	0.986	0.212	4.78E-237	7	Fat1
7.41E-241	0.60322967	0.85	0.14	1.80E-236	7	Ets1
3.06E-240	0.72204324	0.604	0.064	7.41E-236	7	Kitl
3.70E-240	0.25294095	0.465	0.032	8.97E-236	7	Spdl1
2.04E-239	0.689719	0.9	0.152	4.94E-235	7	Gpx8
2.49E-239	1.41001768	0.74	0.109	6.04E-235	7	Mylk
1.19E-238	0.26596917	0.391	0.02	2.88E-234	7	Prr11
2.39E-238	0.66757659	0.848	0.14	5.80E-234	7	Hoxc10
2.65E-238	1.0982488	0.524	0.046	6.43E-234	7	Cenpf
7.27E-237	0.30498321	0.673	0.084	1.76E-232	7	Tcf7l1
2.37E-236	1.03475376	0.734	0.106	5.74E-232	7	Lrrc32
3.57E-236	0.38235695	0.587	0.062	8.65E-232	7	Lama4
5.24E-236	0.95564933	0.892	0.164	1.27E-231	7	Ripk2
1.79E-235	0.46299456	0.806	0.125	4.35E-231	7	Neo1
1.93E-235	0.5271227	0.789	0.119	4.68E-231	7	Nsg1
1.37E-234	0.46051369	0.704	0.093	3.32E-230	7	Tgfbr3
3.12E-234	0.76237571	0.928	0.175	7.57E-230	7	Plp2

3.74E-234	0.54884455	0.82	0.134	9.08E-230	7	Traf4
6.05E-234	0.87048716	0.922	0.167	1.47E-229	7	Fbn1
1.71E-233	0.73867081	0.922	0.168	4.14E-229	7	Dlc1
8.73E-233	0.47434426	0.853	0.14	2.12E-228	7	Mxra7
2.36E-232	0.43701386	0.623	0.07	5.73E-228	7	Uhrf1
2.61E-232	0.4629125	0.85	0.142	6.33E-228	7	Hmgn3
2.21E-231	0.42619746	0.634	0.076	5.35E-227	7	Cenpw
4.91E-231	0.63624601	0.62	0.073	1.19E-226	7	Ckap2
5.42E-231	1.42651734	0.839	0.151	1.32E-226	7	Hmga2
2.33E-230	0.62729585	0.729	0.103	5.64E-226	7	Masp1
7.17E-230	0.33523527	0.482	0.039	1.74E-225	7	Lockd
7.68E-230	0.43062917	0.604	0.067	1.86E-225	7	F2r
1.36E-229	0.54721299	0.895	0.156	3.31E-225	7	Tmem47
2.35E-229	0.44788833	0.759	0.112	5.69E-225	7	Gpc6
5.58E-229	0.68748221	0.823	0.136	1.35E-224	7	Zc2hc1a
2.24E-228	0.6343948	0.662	0.085	5.43E-224	7	Itga1
8.92E-228	0.49973264	0.906	0.16	2.16E-223	7	Yap1
1.82E-227	1.13138106	0.986	0.195	4.40E-223	7	Sdc2
2.02E-227	0.95427954	0.956	0.186	4.89E-223	7	Phlda3
2.86E-227	0.40150307	0.657	0.084	6.95E-223	7	Fah
5.94E-227	1.66313529	0.911	0.182	1.44E-222	7	Col8a1
7.85E-227	0.30393037	0.501	0.042	1.90E-222	7	Cenph
1.13E-225	0.3704225	0.753	0.112	2.75E-221	7	Mpdz

1.59E-225	0.5305869<br>4	0.748	0.115	3.86E-221	7	Mrps6
3.80E-225	0.6309041<br>5	0.87	0.158	9.20E-221	7	Bcar1
4.32E-225	0.4641332<br>8	0.515	0.046	1.05E-220	7	Knstrn
1.15E-224	0.6595603	0.798	0.126	2.78E-220	7	Pcdh19
1.94E-224	0.3818884	0.632	0.077	4.70E-220	7	Tln2
4.03E-224	1.0222626	0.925	0.176	9.76E-220	7	Efnb1
4.09E-224	0.3829001<br>8	0.435	0.031	9.92E-220	7	Syt13
6.80E-224	0.4152588<br>7	0.825	0.136	1.65E-219	7	Syde1
1.55E-223	0.4593983<br>7	0.798	0.127	3.77E-219	7	Bmpr1a
1.01E-222	0.9080684<br>6	0.964	0.186	2.45E-218	7	Bicc1
1.51E-222	0.5271452<br>5	0.665	0.087	3.66E-218	7	Lmnb1
2.87E-222	0.3204351<br>2	0.526	0.049	6.96E-218	7	Dhfr
1.65E-220	0.5268412<br>5	0.626	0.073	3.99E-216	7	Cdca3
2.87E-220	0.3507080<br>9	0.67	0.09	6.95E-216	7	Hoxc9
3.28E-220	0.6985165<br>6	0.95	0.191	7.95E-216	7	Lamc1
9.16E-220	0.5110574<br>6	0.62	0.077	2.22E-215	7	Pdgfra
1.16E-219	0.4712516<br>8	0.767	0.121	2.81E-215	7	Cacna2d1
1.36E-219	0.5476384	0.454	0.036	3.30E-215	7	Sfrp1
2.17E-219	0.2637745	0.488	0.043	5.26E-215	7	St3gal6
1.48E-218	0.3809581<br>6	0.737	0.112	3.58E-214	7	Btg3
1.53E-218	0.4474619<br>3	0.798	0.13	3.72E-214	7	Prdx4
1.98E-218	0.4840251<br>9	0.46	0.035	4.80E-214	7	Kif11
2.46E-218	0.4887761<br>7	0.568	0.059	5.97E-214	7	Ccnb2

3.15E-218	0.64404003	0.903	0.168	7.63E-214	7	Tpbg
4.27E-218	0.35209353	0.704	0.102	1.03E-213	7	Tbx15
9.67E-218	0.50975912	0.74	0.117	2.34E-213	7	Ghr
9.98E-218	0.59324969	0.731	0.111	2.42E-213	7	Adam19
1.27E-217	0.6181541	0.648	0.083	3.08E-213	7	Chst1
1.32E-217	0.43145373	0.789	0.126	3.21E-213	7	Rab23
5.38E-217	0.74372177	0.936	0.182	1.30E-212	7	Tgfb1i1
1.97E-215	0.27363219	0.573	0.065	4.78E-211	7	Mbnl3
2.08E-215	0.38379195	0.618	0.077	5.05E-211	7	Tbx18
2.51E-215	0.67178202	0.748	0.114	6.08E-211	7	Akap12
2.53E-215	0.41246923	0.507	0.047	6.13E-211	7	Bub1b
2.55E-215	0.70738102	0.884	0.158	6.19E-211	7	Shox2
7.82E-215	0.40525742	0.723	0.11	1.90E-210	7	Cd109
8.77E-215	0.87026129	0.9	0.177	2.13E-210	7	Fhl2
1.30E-214	0.44771584	0.77	0.122	3.14E-210	7	Osmr
1.31E-214	0.25575702	0.637	0.081	3.19E-210	7	Rbmx
3.78E-214	0.28055795	0.485	0.043	9.17E-210	7	Gtse1
4.76E-214	1.19548795	0.956	0.207	1.15E-209	7	Vgll3
8.52E-214	0.83757885	0.942	0.191	2.06E-209	7	Pls3
9.36E-214	0.73941001	0.953	0.19	2.27E-209	7	Apbb2
1.14E-213	2.7117106	0.856	0.182	2.77E-209	7	Mgp
2.86E-213	0.26133619	0.396	0.025	6.93E-209	7	Espl1
4.94E-213	0.6021843	0.875	0.156	1.20E-208	7	Kdelr3

5.52E-213	0.37375304	0.717	0.107	1.34E-208	7	Pxylp1
9.48E-213	0.61714691	0.889	0.166	2.30E-208	7	Myo1b
1.28E-212	1.35739206	0.967	0.211	3.11E-208	7	Cyp1b1
1.39E-212	0.27498422	0.573	0.065	3.38E-208	7	Pitx1
2.69E-212	0.68425508	0.889	0.17	6.52E-208	7	Gnb4
6.98E-212	0.86922349	0.615	0.077	1.69E-207	7	Birc5
9.35E-212	0.5024614	0.853	0.148	2.27E-207	7	Mast4
1.72E-211	0.2575468	0.396	0.025	4.18E-207	7	Aurkb
2.38E-211	0.84548675	0.593	0.07	5.77E-207	7	Ccna2
2.44E-211	1.13488918	0.972	0.218	5.91E-207	7	AI506816
3.21E-211	0.51662475	0.814	0.136	7.78E-207	7	Rbms3
2.91E-210	0.69986605	0.934	0.178	7.05E-206	7	Kirrel
3.49E-210	0.29813238	0.551	0.062	8.47E-206	7	Rad18
1.91E-209	0.4887431	0.632	0.084	4.63E-205	7	Trib2
2.28E-209	0.37011365	0.77	0.125	5.53E-205	7	Adgra3
3.30E-209	0.49272658	0.85	0.153	8.00E-205	7	Clstn1
7.81E-209	0.40223205	0.565	0.066	1.89E-204	7	Slc4a4
1.51E-208	1.07201367	0.983	0.212	3.65E-204	7	Ryk
2.98E-208	0.89093988	0.972	0.201	7.24E-204	7	Dcbld2
4.36E-208	0.31414905	0.479	0.042	1.06E-203	7	Kif20b
1.03E-207	0.28356216	0.324	0.015	2.50E-203	7	Ret
1.30E-207	0.51692896	0.756	0.123	3.14E-203	7	Nexn
1.41E-207	1.13406456	0.983	0.214	3.41E-203	7	Vasn

2.00E-207	0.36540229	0.648	0.091	4.85E-203	7	Ccdc85b
2.03E-207	0.89051456	0.947	0.202	4.92E-203	7	Tnfrsf12a
2.23E-207	0.39297817	0.751	0.12	5.40E-203	7	Gnai1
2.48E-207	0.27992971	0.44	0.035	6.02E-203	7	Kifc1
3.64E-207	0.47546181	0.867	0.161	8.82E-203	7	Ncs1
3.99E-207	0.33382585	0.753	0.123	9.68E-203	7	Cdca4
1.84E-206	0.8523806	0.934	0.19	4.46E-202	7	Pbx1
2.23E-206	0.76588162	0.936	0.182	5.41E-202	7	Ptk7
2.25E-206	0.39723739	0.848	0.154	5.46E-202	7	Arhgap35
8.04E-206	0.42381867	0.77	0.126	1.95E-201	7	Ptprg
1.06E-205	0.63718452	0.803	0.142	2.58E-201	7	Oaf
1.08E-205	0.49790542	0.751	0.121	2.61E-201	7	3110039I08Rik
1.13E-205	1.08462029	0.981	0.226	2.73E-201	7	Tead1
1.44E-205	0.28534694	0.676	0.099	3.48E-201	7	Pop1
2.48E-205	0.37418205	0.767	0.128	6.01E-201	7	Akap1
2.55E-205	0.4969486	0.684	0.101	6.18E-201	7	Foxs1
5.43E-205	0.65557157	0.92	0.185	1.32E-200	7	Glipr2
1.05E-204	0.39927146	0.734	0.118	2.53E-200	7	Tead2
5.03E-204	0.63437755	0.823	0.147	1.22E-199	7	Epas1
7.87E-204	0.62886452	0.903	0.166	1.91E-199	7	Antxr1
8.62E-204	0.32034079	0.41	0.031	2.09E-199	7	D030025P21Rik
9.79E-204	1.03173551	0.74	0.123	2.37E-199	7	Tgfb3

1.54E-203	0.33673992	0.601	0.079	3.73E-199	7	Ddr1
5.45E-203	0.36212175	0.751	0.124	1.32E-198	7	Plekhg2
5.70E-203	0.50965066	0.715	0.113	1.38E-198	7	Socs2
5.84E-203	2.05029042	0.964	0.271	1.42E-198	7	Col6a3
6.00E-203	0.53485787	0.801	0.139	1.45E-198	7	Pdlim1
9.69E-203	0.3701214	0.77	0.128	2.35E-198	7	Trim47
1.02E-202	1.16777287	0.449	0.039	2.47E-198	7	Hist1h2ap
1.37E-202	1.26466402	0.925	0.206	3.33E-198	7	Myl9
1.85E-202	0.43958965	0.759	0.127	4.50E-198	7	Dlg5
2.22E-202	0.40610354	0.643	0.089	5.38E-198	7	Ptges
2.84E-202	0.75177476	0.889	0.176	6.88E-198	7	Tmem2
5.87E-202	0.59285688	0.579	0.072	1.42E-197	7	Slco2a1
5.89E-202	0.45666826	0.77	0.134	1.43E-197	7	Hmgn2
7.58E-202	0.80757781	0.814	0.144	1.84E-197	7	Ltbp2
7.66E-202	0.84970325	0.861	0.165	1.86E-197	7	Ltbp1
1.37E-201	1.68992412	0.986	0.275	3.33E-197	7	Col12a1
6.27E-201	0.42755699	0.837	0.152	1.52E-196	7	Lzts2
1.18E-200	0.53299017	0.906	0.177	2.86E-196	7	Pcbp4
5.07E-200	0.26620023	0.546	0.063	1.23E-195	7	Hdgfl3
7.21E-200	0.29035793	0.543	0.061	1.75E-195	7	Chaf1a
1.02E-199	0.27890478	0.427	0.034	2.47E-195	7	Egln3
1.05E-199	0.30474073	0.673	0.099	2.54E-195	7	Cad

2.55E-199	0.68273219	0.831	0.157	6.19E-195	7	Sacs
4.01E-199	1.0925618	0.931	0.211	9.73E-195	7	Slc16a3
6.60E-199	0.32289205	0.717	0.114	1.60E-194	7	Aimp2
7.83E-199	0.42645427	0.798	0.142	1.90E-194	7	Mthfd1l
8.10E-199	0.28620509	0.687	0.106	1.96E-194	7	Adcy6
1.01E-198	1.25891707	0.961	0.228	2.45E-194	7	Amotl2
1.21E-198	0.3773241	0.632	0.087	2.93E-194	7	Atoh8
2.12E-198	0.64064825	0.507	0.055	5.13E-194	7	Sulf1
3.69E-198	0.31876245	0.535	0.061	8.94E-194	7	Fgf2
8.32E-198	1.32336508	0.964	0.229	2.02E-193	7	Prss23
1.50E-197	1.07869866	0.953	0.201	3.64E-193	7	Dclk1
2.14E-197	0.26834258	0.546	0.064	5.18E-193	7	Nkd2
5.70E-197	0.33646261	0.706	0.113	1.38E-192	7	Armcx2
6.51E-197	0.44804577	0.845	0.157	1.58E-192	7	Znhit6
6.71E-197	0.31404389	0.452	0.039	1.63E-192	7	Iqgap3
7.45E-197	0.44907265	0.742	0.123	1.81E-192	7	Ptprf
1.53E-196	0.29034902	0.482	0.049	3.72E-192	7	Mark1
1.99E-196	0.26585476	0.665	0.099	4.83E-192	7	Erbb2
2.67E-196	0.35082544	0.778	0.133	6.47E-192	7	Spats2
2.88E-196	0.49519648	0.814	0.146	6.97E-192	7	Map1b
3.41E-196	0.4493064	0.801	0.144	8.26E-192	7	Fbxo30
7.06E-195	0.39000694	0.789	0.137	1.71E-190	7	Dab2ip
1.66E-194	1.17157338	0.958	0.209	4.03E-190	7	Ccdc80

1.71E-194	0.98075356	0.875	0.172	4.15E-190	7	Tgfb2
1.97E-194	0.35296516	0.751	0.125	4.77E-190	7	Tulp3
2.23E-194	0.34924039	0.734	0.12	5.41E-190	7	Glis2
2.25E-194	0.26596156	0.773	0.132	5.46E-190	7	Mast2
2.46E-194	0.44883186	0.845	0.156	5.97E-190	7	Tmem132a
2.92E-194	0.25416323	0.302	0.014	7.07E-190	7	Avpr1a
6.00E-194	1.5455519	0.981	0.249	1.45E-189	7	Col6a1
9.18E-194	0.58371054	0.931	0.19	2.23E-189	7	Nckap1
9.93E-194	0.46256534	0.861	0.164	2.41E-189	7	Chsy1
1.22E-193	0.69758828	0.593	0.078	2.96E-189	7	Racgap1
4.72E-193	0.52186822	0.823	0.155	1.15E-188	7	Slc2a1
1.08E-192	0.68795113	0.958	0.198	2.62E-188	7	Cdc42bpa
1.13E-192	0.33245047	0.77	0.133	2.74E-188	7	Tjp1
1.20E-192	0.45219785	0.693	0.111	2.91E-188	7	Spry2
1.53E-192	0.32226413	0.709	0.117	3.71E-188	7	Tnfrsf10b
1.69E-192	0.54068928	0.612	0.087	4.11E-188	7	Spry4
1.87E-192	0.32982938	0.801	0.145	4.54E-188	7	10-Sep
6.23E-192	0.84140227	0.942	0.211	1.51E-187	7	Ktn1
6.37E-192	0.30957357	0.767	0.134	1.54E-187	7	Yes1
8.67E-192	0.60273529	0.953	0.204	2.10E-187	7	Parva
1.34E-191	0.59978383	0.792	0.14	3.26E-187	7	Slit2
1.53E-191	0.54156421	0.643	0.1	3.70E-187	7	Trim59

3.36E-191	0.26882161	0.828	0.154	8.16E-187	7	Rnf126
5.86E-191	0.56223185	0.598	0.079	1.42E-186	7	Tacc3
7.34E-191	0.70640829	0.889	0.187	1.78E-186	7	Epb41l3
8.00E-191	0.31901928	0.512	0.058	1.94E-186	7	Ddit4l
1.13E-190	0.33778654	0.795	0.146	2.73E-186	7	Map4k5
2.35E-190	0.5138526	0.407	0.032	5.69E-186	7	Nusap1
2.37E-190	0.37941887	0.565	0.072	5.75E-186	7	Ngf
5.91E-190	0.31262921	0.731	0.125	1.43E-185	7	Ndufaf2
7.06E-190	0.3106801	0.723	0.119	1.71E-185	7	Ube2e2
9.58E-190	0.52182868	0.831	0.163	2.32E-185	7	Dkc1
1.10E-189	0.46397719	0.759	0.133	2.66E-185	7	Podnl1
1.39E-189	0.28800289	0.715	0.118	3.37E-185	7	Rpa3
1.45E-189	0.38692713	0.723	0.119	3.52E-185	7	Ak4
1.56E-189	0.43389912	0.607	0.086	3.77E-185	7	AC131339.2
1.58E-189	0.36863409	0.856	0.167	3.83E-185	7	Fam92a
3.30E-189	0.28254453	0.596	0.081	8.00E-185	7	Hoxa9
6.28E-189	0.58774297	0.878	0.181	1.52E-184	7	Ddah1
1.10E-188	0.37269654	0.77	0.135	2.66E-184	7	Pard3
1.14E-188	0.46669838	0.831	0.156	2.76E-184	7	Emp2
1.27E-188	0.51241387	0.864	0.168	3.08E-184	7	Ctdspl
1.27E-188	0.53028638	0.873	0.177	3.09E-184	7	Igf2bp2
1.47E-188	0.33366404	0.745	0.13	3.57E-184	7	Arhgef40
1.61E-188	0.4130785	0.82	0.151	3.91E-184	7	Epn2

1.77E-188	0.27237778	0.593	0.081	4.30E-184	7	Mum1l1
1.85E-188	0.45344606	0.576	0.076	4.49E-184	7	Peg10
3.52E-188	0.63529625	0.928	0.188	8.53E-184	7	Amotl1
4.58E-188	0.49947611	0.767	0.142	1.11E-183	7	H2afx
4.86E-188	0.25270283	0.537	0.066	1.18E-183	7	Fbln1
5.85E-188	0.28113097	0.662	0.101	1.42E-183	7	Etv4
1.52E-187	0.4915926	0.839	0.163	3.68E-183	7	Rhoj
1.66E-187	0.45828649	0.825	0.157	4.02E-183	7	Cavin3
2.16E-187	0.25670871	0.407	0.034	5.24E-183	7	1500009L16Rik
4.21E-187	0.28681423	0.748	0.128	1.02E-182	7	Dgkh
4.29E-187	0.26880984	0.825	0.151	1.04E-182	7	Wwtr1
8.82E-187	0.34720165	0.745	0.127	2.14E-182	7	Pdia5
8.90E-187	1.07944777	0.634	0.095	2.16E-182	7	Ube2c
1.65E-186	0.62072272	0.706	0.118	4.00E-182	7	Zfp503
4.05E-186	0.47323965	0.781	0.141	9.83E-182	7	Htra1
5.64E-186	0.44353692	0.77	0.14	1.37E-181	7	Adgrl1
2.50E-185	0.40153904	0.787	0.147	6.05E-181	7	Lmf2
6.30E-185	0.52762093	0.792	0.154	1.53E-180	7	Dut
6.50E-185	0.33964871	0.623	0.09	1.58E-180	7	C130075A20Rik
7.20E-185	0.34307717	0.537	0.066	1.75E-180	7	Plk4
8.37E-185	0.57583335	0.596	0.082	2.03E-180	7	Crlf1
1.21E-184	0.65209832	0.922	0.2	2.94E-180	7	Bcl9l

1.63E-184	0.3638438<br>2	0.709	0.119	3.96E-180	7	Bag2
2.34E-184	0.3021986<br>9	0.657	0.102	5.68E-180	7	Cfap43
2.37E-184	0.2869536<br>8	0.643	0.098	5.74E-180	7	Orc6
2.96E-184	0.3278771<br>2	0.825	0.153	7.18E-180	7	Yars
4.49E-184	1.0899684<br>9	0.95	0.23	1.09E-179	7	Eno1
1.15E-183	0.3747066<br>6	0.814	0.152	2.79E-179	7	Tmem63b
2.09E-183	0.2822811<br>1	0.626	0.094	5.06E-179	7	Trmt10a
7.15E-183	1.1189862<br>3	0.972	0.27	1.73E-178	7	Tubb6
8.01E-183	0.2589124<br>9	0.626	0.092	1.94E-178	7	Slc19a2
1.25E-182	0.4696046<br>5	0.479	0.053	3.04E-178	7	Lmod1
1.67E-182	0.2886400<br>5	0.773	0.136	4.05E-178	7	Phldb1
1.69E-182	0.4087142	0.828	0.158	4.11E-178	7	Zcchc14
4.02E-182	0.6151433<br>8	0.934	0.192	9.75E-178	7	P4ha2
4.15E-182	0.2653688<br>1	0.748	0.132	1.01E-177	7	Trip6
4.72E-182	0.7395590<br>2	0.892	0.186	1.14E-177	7	Psat1
4.85E-182	0.8247340<br>3	0.914	0.187	1.18E-177	7	Snai2
5.70E-182	0.5819385<br>2	0.665	0.107	1.38E-177	7	Hells
7.44E-182	0.4336583<br>8	0.687	0.116	1.80E-177	7	Ndc1
8.24E-182	0.4357639<br>6	0.839	0.168	2.00E-177	7	Vars
1.30E-181	0.356604	0.748	0.136	3.15E-177	7	Creld2
2.65E-181	0.3288346<br>3	0.687	0.113	6.42E-177	7	Igsf3
3.21E-181	0.5568332<br>2	0.765	0.135	7.79E-177	7	Srpx2

3.58E-181	0.5379117 8	0.906	0.196	8.68E-177	7	Aen
4.32E-181	0.2605972 2	0.659	0.103	1.05E-176	7	Il17rc
4.46E-181	0.2766633 1	0.604	0.088	1.08E-176	7	Farp1
5.07E-181	0.6241355 9	0.848	0.165	1.23E-176	7	Gja1
7.61E-181	0.2934399 3	0.798	0.149	1.85E-176	7	Mpzl1
7.87E-181	0.9194164 6	0.837	0.168	1.91E-176	7	Pdk4
8.26E-181	0.3175725 4	0.634	0.096	2.00E-176	7	Auts2
1.16E-180	0.3192659 1	0.704	0.118	2.80E-176	7	Tspan6
1.27E-180	0.6067693 8	0.87	0.166	3.08E-176	7	Adamts2
2.32E-180	0.2611002 9	0.643	0.1	5.64E-176	7	Smo
3.98E-180	0.3624448 9	0.695	0.117	9.65E-176	7	Gcnt2
4.03E-180	0.7988194 8	0.961	0.216	9.77E-176	7	Grb10
4.34E-180	1.4665781 7	0.997	0.299	1.05E-175	7	Vcl
5.59E-180	0.2971492 9	0.648	0.104	1.35E-175	7	Dis3
5.72E-180	0.3577040 7	0.892	0.172	1.39E-175	7	Scarf2
9.88E-180	1.0505991 1	0.992	0.254	2.40E-175	7	Myo10
1.29E-179	0.5495811 7	0.834	0.162	3.12E-175	7	Kcnq1ot1
1.30E-179	0.6485382 5	0.892	0.18	3.14E-175	7	Rnd3
1.42E-179	0.3206399 9	0.898	0.184	3.45E-175	7	Uck2
2.12E-179	1.0114390 9	0.911	0.21	5.15E-175	7	Crim1
2.41E-179	0.2683507 7	0.604	0.088	5.85E-175	7	Micall2

3.36E-179	0.43224116	0.848	0.167	8.15E-175	7	Afap1
4.17E-179	0.42547269	0.687	0.112	1.01E-174	7	Fam20a
8.94E-179	0.51375863	0.839	0.163	2.17E-174	7	Lpar1
8.95E-179	0.75526644	0.706	0.123	2.17E-174	7	Thbd
1.07E-178	0.94893913	0.939	0.237	2.59E-174	7	Tubb4b
1.51E-178	0.25922576	0.504	0.06	3.67E-174	7	Egfr
1.82E-178	0.48890688	0.895	0.194	4.42E-174	7	Pfkl
1.90E-178	0.25480399	0.662	0.105	4.61E-174	7	Stk39
2.33E-178	0.98537393	0.961	0.244	5.65E-174	7	Prkar2b
2.45E-178	0.31791569	0.787	0.148	5.95E-174	7	Ptk2
2.50E-178	0.35286096	0.715	0.125	6.06E-174	7	Slc16a1
2.70E-178	0.26578346	0.657	0.103	6.54E-174	7	Sh3rf1
3.43E-178	0.52398094	0.429	0.041	8.32E-174	7	Ccne2
6.66E-178	0.35220922	0.806	0.156	1.62E-173	7	Tmtc3
7.62E-178	0.46166361	0.609	0.093	1.85E-173	7	Dbf4
1.20E-177	0.33843064	0.615	0.09	2.90E-173	7	Mad2l1
1.36E-177	0.34539728	0.681	0.113	3.30E-173	7	Ptprk
3.45E-177	0.37337274	0.662	0.111	8.38E-173	7	Hoxa10
4.21E-177	0.63651673	0.945	0.218	1.02E-172	7	Pkn2
5.52E-177	0.27992309	0.698	0.12	1.34E-172	7	Tmem185a
1.11E-176	0.326503	0.867	0.18	2.70E-172	7	Acot7
1.25E-176	0.65777923	0.898	0.2	3.04E-172	7	Dnajb4

1.45E-176	0.39036864	0.798	0.149	3.51E-172	7	Tanc1
1.46E-176	0.48897145	0.9	0.193	3.54E-172	7	Ctps
1.65E-176	0.33638769	0.814	0.159	3.99E-172	7	Jmjd6
1.86E-176	0.67894192	0.958	0.216	4.52E-172	7	Cttn
3.31E-176	0.30459142	0.67	0.11	8.04E-172	7	Ndn
5.25E-176	0.35362049	0.806	0.153	1.27E-171	7	Kank2
5.26E-176	0.35827499	0.745	0.134	1.27E-171	7	Tcaf1
8.63E-176	0.40423202	0.571	0.081	2.09E-171	7	Runx1t1
1.66E-175	0.58676163	0.488	0.055	4.03E-171	7	Ccnb1
1.71E-175	0.53055003	0.922	0.197	4.15E-171	7	Me1
1.77E-175	0.75883351	0.928	0.207	4.29E-171	7	Nes
1.95E-175	0.28250475	0.776	0.144	4.72E-171	7	Ptrh1
2.40E-175	0.27990031	0.338	0.023	5.83E-171	7	Cox4i2
2.59E-175	0.35601201	0.712	0.125	6.28E-171	7	Fstl3
2.73E-175	1.04083961	0.942	0.217	6.62E-171	7	Fgf7
3.20E-175	0.30321324	0.801	0.154	7.76E-171	7	Snta1
3.42E-175	0.42741788	0.618	0.094	8.28E-171	7	Gm38211
3.65E-175	0.49217299	0.895	0.184	8.85E-171	7	Ssr2
3.91E-175	0.41766144	0.861	0.169	9.48E-171	7	Pkd2
4.46E-175	0.52153529	0.867	0.19	1.08E-170	7	Lyar
4.73E-175	0.43656163	0.853	0.178	1.15E-170	7	Tmem97

5.45E-175	0.52596527	0.825	0.164	1.32E-170	7	Slc20a1
1.69E-174	0.25197974	0.726	0.129	4.09E-170	7	Sipa1l1
2.49E-174	0.7946322	0.95	0.222	6.05E-170	7	Smad7
3.32E-174	0.31956782	0.737	0.135	8.04E-170	7	H2afy2
3.34E-174	0.81218536	0.867	0.182	8.10E-170	7	Olfml3
3.64E-174	0.28938006	0.668	0.112	8.83E-170	7	Gm14706
3.89E-174	0.37101809	0.814	0.159	9.43E-170	7	Pabpn1
5.23E-174	0.76071477	0.972	0.227	1.27E-169	7	Msrb3
5.28E-174	0.37897554	0.823	0.164	1.28E-169	7	Fam98a
5.40E-174	0.65176447	0.911	0.189	1.31E-169	7	Creb3l1
7.04E-174	0.47877799	0.864	0.168	1.71E-169	7	P3h4
1.36E-173	1.19051753	0.972	0.241	3.31E-169	7	Serpine2
1.92E-173	0.3388942	0.831	0.167	4.66E-169	7	Fnbp1l
2.01E-173	0.2862027	0.723	0.128	4.87E-169	7	Pomgnt1
2.35E-173	0.27882636	0.695	0.118	5.71E-169	7	Mllt3
2.52E-173	0.72127543	0.956	0.216	6.10E-169	7	Utrn
2.69E-173	0.36919714	0.817	0.163	6.53E-169	7	Cep170b
2.93E-173	1.19169522	0.983	0.262	7.11E-169	7	Fscn1
3.26E-173	0.92483909	0.975	0.245	7.90E-169	7	Igf1r
5.00E-173	0.98369643	0.399	0.036	1.21E-168	7	Hist1h1b
5.98E-173	0.37530788	0.778	0.144	1.45E-168	7	Npdc1
8.84E-173	0.30802809	0.734	0.133	2.14E-168	7	Tacc2
1.09E-172	0.2639069	0.598	0.089	2.64E-168	7	Ubtd2

1.36E-172	0.32674805	0.759	0.143	3.29E-168	7	Ubap2
1.45E-172	0.68282585	0.931	0.214	3.52E-168	7	Shmt2
1.82E-172	0.56978292	0.845	0.172	4.40E-168	7	Bcat1
2.36E-172	0.47820487	0.82	0.159	5.73E-168	7	Dbn1
2.78E-172	0.25918308	0.82	0.164	6.75E-168	7	Smg5
2.88E-172	1.84228545	0.997	0.409	6.99E-168	7	Serpinh1
3.34E-172	1.43946417	0.989	0.281	8.11E-168	7	Prrx1
5.04E-172	0.63652682	0.903	0.198	1.22E-167	7	Socs5
6.02E-172	0.31582645	0.305	0.018	1.46E-167	7	Serping1
6.23E-172	0.30687098	0.864	0.179	1.51E-167	7	Utp20
6.58E-172	0.86649618	0.961	0.25	1.60E-167	7	Nop58
6.62E-172	0.58739492	0.922	0.193	1.60E-167	7	Twist1
7.51E-172	0.2986999	0.784	0.149	1.82E-167	7	Mex3d
8.26E-172	0.27841127	0.443	0.045	2.00E-167	7	Slfn9
8.55E-172	0.56196025	0.803	0.164	2.07E-167	7	Ptpn14
1.38E-171	0.29581769	0.806	0.16	3.35E-167	7	Dpp9
2.03E-171	0.30030488	0.679	0.116	4.91E-167	7	Tnks1bp1
2.04E-171	0.82653031	0.837	0.189	4.94E-167	7	Cks2
3.41E-171	0.3314637	0.615	0.095	8.27E-167	7	Chst15
4.50E-171	1.41064767	0.654	0.115	1.09E-166	7	Top2a
4.68E-171	0.35501176	0.801	0.161	1.13E-166	7	Nup205
5.83E-171	0.32830823	0.873	0.184	1.41E-166	7	Aida

6.14E-171	0.25933322	0.41	0.037	1.49E-166	7	Fbxo5
1.07E-170	0.4384717	0.82	0.169	2.59E-166	7	Camk2g
1.15E-170	0.31256698	0.776	0.15	2.79E-166	7	Cbx6
1.20E-170	0.2872136	0.773	0.147	2.91E-166	7	Tex10
2.15E-170	0.30421938	0.731	0.132	5.20E-166	7	Tmeff1
3.19E-170	0.78437172	0.643	0.104	7.75E-166	7	Ptx3
3.72E-170	0.31235112	0.767	0.14	9.03E-166	7	Epdr1
4.60E-170	0.43476804	0.676	0.121	1.12E-165	7	Myh10
5.08E-170	0.93890932	0.956	0.242	1.23E-165	7	Fermt2
6.43E-170	0.44053788	0.903	0.201	1.56E-165	7	Cdk2ap1
6.73E-170	1.4115732	1	0.344	1.63E-165	7	Lpp
6.96E-170	0.26599411	0.77	0.147	1.69E-165	7	Gm6863
7.95E-170	0.56232752	0.687	0.123	1.93E-165	7	Aqp1
8.23E-170	0.28326209	0.784	0.151	2.00E-165	7	Fasn
1.03E-169	0.28411132	0.615	0.097	2.50E-165	7	Sox12
1.12E-169	0.27221618	0.784	0.151	2.72E-165	7	Usp10
1.76E-169	0.2523803	0.74	0.134	4.27E-165	7	Eml1
1.90E-169	0.2542643	0.745	0.141	4.61E-165	7	Cinp
3.89E-169	0.28234726	0.767	0.149	9.42E-165	7	Larp7
4.97E-169	0.42247407	0.856	0.186	1.21E-164	7	Ilf3
7.63E-169	0.52168379	0.922	0.201	1.85E-164	7	Slc30a4
8.25E-169	1.04978347	0.961	0.237	2.00E-164	7	Pmepa1
8.69E-169	0.67007241	0.632	0.104	2.11E-164	7	Smoc2

9.85E-169	0.7223758<br>2	0.947	0.229	2.39E-164	7	Cfl2
1.21E-168	0.3700077<br>5	0.801	0.163	2.92E-164	7	Ppm1g
1.37E-168	0.8838832<br>4	0.964	0.245	3.32E-164	7	Fam162a
1.43E-168	0.4194227<br>2	0.881	0.184	3.46E-164	7	Atp8b2
1.66E-168	1.3760623<br>8	0.989	0.314	4.02E-164	7	Tpm2
7.76E-168	0.3276066<br>5	0.773	0.152	1.88E-163	7	Mettl1
9.96E-168	0.8407646<br>2	0.97	0.255	2.41E-163	7	Ttc39b
1.80E-167	0.2623207<br>9	0.72	0.132	4.36E-163	7	Bysl
1.87E-167	0.2962082<br>7	0.648	0.109	4.54E-163	7	Lgalsl
2.85E-167	0.4820111<br>6	0.773	0.148	6.92E-163	7	Klf9
3.20E-167	0.6842391<br>6	0.956	0.214	7.77E-163	7	Chpf
3.69E-167	0.3689678<br>7	0.518	0.069	8.94E-163	7	Unc5c
3.93E-167	0.3356691<br>4	0.596	0.092	9.53E-163	7	Pcdh18
4.40E-167	0.4353641<br>3	0.72	0.132	1.07E-162	7	Pcdh7
6.76E-167	0.5427652<br>8	0.884	0.192	1.64E-162	7	Snx7
7.56E-167	0.3250551<br>1	0.745	0.144	1.83E-162	7	Rcc1
9.51E-167	0.7950164<br>8	0.676	0.126	2.31E-162	7	Smc2
1.22E-166	0.2757964<br>7	0.798	0.159	2.96E-162	7	Magohb
1.50E-166	0.4676782<br>5	0.942	0.21	3.64E-162	7	Samd4
1.93E-166	0.2696575<br>1	0.507	0.067	4.68E-162	7	Nav3
4.01E-166	0.2813530<br>7	0.704	0.128	9.73E-162	7	Timm8a1

5.68E-166	0.25682848	0.77	0.148	1.38E-161	7	Ankrd27
5.89E-166	0.94310373	0.465	0.055	1.43E-161	7	Actg2
8.13E-166	0.29405738	0.526	0.072	1.97E-161	7	Ptprn
8.25E-166	0.86908094	0.753	0.153	2.00E-161	7	Plpp3
1.64E-165	0.28617174	0.85	0.174	3.97E-161	7	Ammecr1
2.04E-165	0.70313655	0.95	0.229	4.95E-161	7	Srm
2.31E-165	0.29547023	0.823	0.164	5.59E-161	7	Mdn1
2.81E-165	0.71408874	0.809	0.163	6.80E-161	7	Irx3
4.74E-165	0.502006	0.895	0.199	1.15E-160	7	Luzp1
5.27E-165	0.64221827	0.861	0.206	1.28E-160	7	Cks1b
5.28E-165	0.40572877	0.742	0.141	1.28E-160	7	Slc7a5
7.89E-165	0.3205332	0.69	0.121	1.91E-160	7	Slc1a4
1.10E-164	1.79751318	0.997	0.444	2.68E-160	7	Bgn
1.13E-164	0.48247917	0.878	0.185	2.73E-160	7	Tmem263
2.18E-164	0.62996424	0.684	0.123	5.30E-160	7	Ogn
4.12E-164	0.89301007	0.978	0.235	1.00E-159	7	Lhfp
5.34E-164	0.34039057	0.781	0.155	1.29E-159	7	Rrp8
5.75E-164	0.32421511	0.693	0.123	1.39E-159	7	Gli3
1.10E-163	0.66696567	0.623	0.1	2.67E-159	7	Col3a1
1.12E-163	0.40638657	0.867	0.186	2.72E-159	7	Gys1
1.26E-163	0.38788047	0.842	0.174	3.05E-159	7	Rab34
1.46E-163	0.50809611	0.856	0.196	3.53E-159	7	Lbr

1.66E-163	0.43912302	0.809	0.174	4.03E-159	7	Nsd2
2.44E-163	0.28591111	0.673	0.118	5.91E-159	7	Hat1
3.63E-163	0.2606377	0.759	0.148	8.81E-159	7	Gmds
5.56E-163	0.30851618	0.759	0.147	1.35E-158	7	Scd1
8.03E-163	0.38889377	0.889	0.187	1.95E-158	7	Selenom
1.35E-162	0.25309006	0.576	0.088	3.28E-158	7	Zfp9
1.69E-162	0.26164503	0.715	0.134	4.09E-158	7	Erc1
1.76E-162	0.29857846	0.806	0.163	4.27E-158	7	Fam8a1
2.30E-162	0.47773979	0.529	0.074	5.58E-158	7	Spc25
3.47E-162	0.37602377	0.834	0.17	8.40E-158	7	Col16a1
3.97E-162	0.26081409	0.665	0.117	9.63E-158	7	Cdr2
4.02E-162	0.32304901	0.69	0.127	9.74E-158	7	Tipin
6.43E-162	0.29318877	0.507	0.068	1.56E-157	7	Vegfa
7.62E-162	0.3049617	0.853	0.183	1.85E-157	7	Ptcd3
1.34E-161	1.05498492	0.983	0.286	3.25E-157	7	Esyt2
1.54E-161	0.48997094	0.892	0.205	3.74E-157	7	Cdk14
1.96E-161	0.35791729	0.798	0.157	4.76E-157	7	Nradd
2.03E-161	0.42599234	0.856	0.183	4.93E-157	7	Pdcd11
2.08E-161	0.92126671	0.95	0.22	5.04E-157	7	Plod2
2.74E-161	0.27518715	0.474	0.059	6.64E-157	7	Efna5
3.31E-161	0.83667826	0.67	0.124	8.03E-157	7	Adamts1
4.69E-161	0.29737058	0.784	0.162	1.14E-156	7	Ahctf1

4.75E-161	0.47246211	0.958	0.213	1.15E-156	7	Ddr2
6.09E-161	0.39536332	0.698	0.133	1.48E-156	7	Psrc1
6.41E-161	0.29600647	0.693	0.126	1.55E-156	7	Arhgap29
8.39E-161	0.46904173	0.914	0.2	2.03E-156	7	Ikbip
1.05E-160	0.52968555	0.886	0.201	2.55E-156	7	Polr2l
1.69E-160	0.37428654	0.886	0.202	4.10E-156	7	Emd
2.10E-160	0.90559662	0.994	0.244	5.10E-156	7	Fkbp9
4.11E-160	0.53704896	0.873	0.21	9.97E-156	7	Psip1
6.46E-160	0.25904331	0.789	0.155	1.57E-155	7	Otud7b
6.49E-160	0.25732433	0.762	0.151	1.57E-155	7	Tsr1
8.71E-160	0.55625977	0.928	0.217	2.11E-155	7	Ppa1
9.27E-160	0.54855538	0.925	0.22	2.25E-155	7	Wwc2
9.66E-160	0.31404672	0.717	0.138	2.34E-155	7	Usp1
1.03E-159	0.5969477	0.945	0.233	2.49E-155	7	Psmd7
1.21E-159	0.43311548	0.903	0.206	2.93E-155	7	Gna11
1.28E-159	0.27101945	0.607	0.099	3.10E-155	7	Kalrn
1.60E-159	0.31315379	0.801	0.165	3.87E-155	7	Adrm1
1.98E-159	0.44148603	0.834	0.174	4.81E-155	7	Ttc28
2.05E-159	0.26017036	0.759	0.151	4.96E-155	7	Arhgap21
2.52E-159	0.41012435	0.864	0.195	6.12E-155	7	Ppp2r2d
3.26E-159	0.28194441	0.745	0.148	7.91E-155	7	Lnpk
3.61E-159	0.37661497	0.85	0.187	8.74E-155	7	Mak16

3.89E-159	1.68288159	0.997	0.408	9.44E-155	7	Csf1
4.04E-159	0.36967215	0.629	0.106	9.80E-155	7	Irx5
4.05E-159	0.32079947	0.659	0.115	9.82E-155	7	Fkbp11
4.47E-159	0.66359127	0.861	0.177	1.08E-154	7	Cdh2
5.84E-159	1.3543127	0.992	0.31	1.42E-154	7	Aebp1
6.22E-159	0.25478893	0.607	0.101	1.51E-154	7	Chst2
1.34E-158	0.28235594	0.557	0.083	3.24E-154	7	BC002163
1.84E-158	0.33385396	0.842	0.177	4.46E-154	7	Ptgfrn
1.84E-158	0.32955043	0.69	0.127	4.47E-154	7	Clcf1
2.11E-158	0.34190944	0.693	0.127	5.12E-154	7	Mcm2
2.20E-158	0.41058295	0.845	0.186	5.32E-154	7	Polk
2.53E-158	0.27565174	0.817	0.17	6.13E-154	7	Lrrc42
2.75E-158	0.30281502	0.839	0.18	6.66E-154	7	Btbd7
3.89E-158	0.33540619	0.828	0.182	9.42E-154	7	Eftud2
4.02E-158	0.2804083	0.612	0.102	9.76E-154	7	Scara3
4.14E-158	0.26259506	0.784	0.163	1.00E-153	7	Mrpl18
5.30E-158	0.25062133	0.77	0.153	1.29E-153	7	Frs2
1.01E-157	0.34474084	0.723	0.137	2.44E-153	7	Gpc4
1.15E-157	0.44705234	0.795	0.165	2.79E-153	7	Plxdc2
1.79E-157	0.6873463	0.925	0.211	4.34E-153	7	Frmd6
1.82E-157	0.28862934	0.759	0.147	4.42E-153	7	Nucb2
2.02E-157	0.48357632	0.712	0.133	4.89E-153	7	Snai1
2.32E-157	0.38871081	0.637	0.107	5.64E-153	7	Mcm5

4.77E-157	0.27619109	0.532	0.078	1.16E-152	7	Cx3cl1
4.95E-157	0.80024375	0.967	0.261	1.20E-152	7	Higd1a
6.63E-157	0.3818839	0.881	0.193	1.61E-152	7	Rras2
1.01E-156	0.25116522	0.681	0.124	2.46E-152	7	Uaca
1.09E-156	0.31094426	0.751	0.154	2.63E-152	7	Ezh2
1.11E-156	0.88872927	0.981	0.243	2.68E-152	7	Nfix
2.02E-156	0.29517817	0.751	0.152	4.91E-152	7	Pus7
2.14E-156	0.27110488	0.421	0.047	5.19E-152	7	Kazald1
2.56E-156	0.29117985	0.773	0.155	6.21E-152	7	Itpr1
2.73E-156	0.26597486	0.429	0.049	6.61E-152	7	Rem1
2.77E-156	0.69342671	0.975	0.253	6.72E-152	7	Mprip
3.19E-156	0.29285086	0.623	0.105	7.74E-152	7	Mcm4
3.34E-156	0.40583939	0.87	0.2	8.10E-152	7	Ilf2
3.67E-156	0.26177781	0.681	0.123	8.91E-152	7	Slc1a5
4.76E-156	0.28430222	0.709	0.138	1.15E-151	7	Brip1os
1.06E-155	0.42892417	0.92	0.222	2.56E-151	7	Myef2
1.08E-155	1.06949815	0.626	0.111	2.61E-151	7	Cxcl14
1.09E-155	0.31049063	0.798	0.171	2.64E-151	7	Pitrm1
1.18E-155	0.45921291	0.465	0.059	2.85E-151	7	Lmcd1
1.22E-155	0.27357685	0.842	0.184	2.96E-151	7	Polr2c
1.35E-155	0.32491716	0.582	0.095	3.26E-151	7	Arxes2
1.64E-155	0.34780576	0.825	0.173	3.98E-151	7	Iffo2

1.68E-155	0.58407328	0.695	0.132	4.08E-151	7	Hbegf
1.98E-155	0.25922731	0.795	0.165	4.79E-151	7	Erh
2.05E-155	0.66343428	0.911	0.214	4.98E-151	7	Crip2
2.08E-155	0.26154137	0.792	0.165	5.03E-151	7	Ndufaf4
2.39E-155	0.34033341	0.77	0.159	5.79E-151	7	Ptp4a3
4.96E-155	0.36013154	0.85	0.191	1.20E-150	7	Got2
5.23E-155	0.58979931	0.69	0.141	1.27E-150	7	Hjurp
7.94E-155	0.86496761	0.981	0.243	1.92E-150	7	Efemp2
8.27E-155	1.32753043	0.997	0.325	2.01E-150	7	Cavin1
9.66E-155	0.5282128	0.9	0.213	2.34E-150	7	Slc39a6
1.78E-154	0.26825019	0.659	0.118	4.30E-150	7	Pcyox1l
2.24E-154	0.33146755	0.452	0.056	5.44E-150	7	Sema3c
3.63E-154	0.54017194	0.895	0.21	8.81E-150	7	Bzw2
4.00E-154	0.25895718	0.676	0.122	9.69E-150	7	Pard6g
4.69E-154	0.9426902	0.983	0.241	1.14E-149	7	Fkbp10
8.70E-154	0.47723429	0.934	0.23	2.11E-149	7	Dlst
1.05E-153	0.26187037	0.814	0.171	2.55E-149	7	Ppan
1.67E-153	0.28968022	0.776	0.161	4.06E-149	7	Ankrd50
1.94E-153	0.28317496	0.77	0.154	4.71E-149	7	Alkbh1
2.11E-153	0.44231158	0.798	0.166	5.12E-149	7	Runx2
2.45E-153	0.2747722	0.507	0.072	5.95E-149	7	Gm43343
3.49E-153	0.90152614	0.701	0.14	8.45E-149	7	Rgs16
4.00E-153	0.26058387	0.717	0.138	9.70E-149	7	Dnajc25

5.71E-153	0.25555766	0.787	0.162	1.38E-148	7	Selenoi
5.74E-153	0.51697773	0.953	0.24	1.39E-148	7	mt-Atp8
6.56E-153	0.95365641	0.992	0.273	1.59E-148	7	2310022B05Rik
7.91E-153	0.27389816	0.789	0.165	1.92E-148	7	Yeats2
1.82E-152	0.30945026	0.604	0.103	4.42E-148	7	Pak3
4.65E-152	0.2783782	0.681	0.13	1.13E-147	7	Wtip
5.24E-152	0.86845611	0.898	0.245	1.27E-147	7	Rrm1
7.34E-152	0.41061912	0.859	0.186	1.78E-147	7	Asns
8.55E-152	0.43391092	0.853	0.194	2.07E-147	7	Ddx18
1.35E-151	0.78961025	0.942	0.228	3.28E-147	7	Cpe
1.53E-151	0.33970248	0.839	0.184	3.70E-147	7	Pgrmc2
5.55E-151	0.71220084	0.956	0.229	1.35E-146	7	Gpc1
6.75E-151	0.67784086	0.936	0.228	1.64E-146	7	Pdgfa
6.81E-151	0.25351779	0.845	0.185	1.65E-146	7	Slc4a2
7.11E-151	0.30822744	0.837	0.186	1.72E-146	7	Sigmar1
9.55E-151	0.36250731	0.889	0.206	2.32E-146	7	Klhdc2
9.97E-151	0.29394359	0.831	0.185	2.42E-146	7	Impdh2
1.70E-150	0.28498316	0.787	0.164	4.13E-146	7	Grpel1
2.05E-150	0.5297982	0.928	0.233	4.96E-146	7	Odc1
3.66E-150	0.43275232	0.62	0.108	8.88E-146	7	Mustn1
3.69E-150	0.91947775	0.906	0.219	8.95E-146	7	Thbs2
4.63E-150	0.5385125	0.925	0.221	1.12E-145	7	Nav2
5.01E-150	0.26139359	0.546	0.085	1.21E-145	7	Mex3a

5.17E-150	0.2502241 8	0.778	0.166	1.25E-145	7	Adk
7.34E-150	0.3141366 1	0.72	0.143	1.78E-145	7	Sh3bp4
1.10E-149	0.2989891 8	0.587	0.098	2.68E-145	7	Hoxc8
1.53E-149	0.2769281 1	0.709	0.14	3.72E-145	7	Rif1
3.06E-149	0.4543177 2	0.817	0.188	7.41E-145	7	Nasp
3.16E-149	0.2776295 1	0.476	0.065	7.66E-145	7	Pkp2
3.84E-149	0.3199670 6	0.812	0.172	9.31E-145	7	Sertad2
4.11E-149	0.3785703 5	0.837	0.195	9.97E-145	7	Lsm8
9.72E-149	0.2525493 7	0.792	0.172	2.36E-144	7	Mrpl45
1.06E-148	0.2535413 5	0.584	0.097	2.57E-144	7	Six1
1.31E-148	0.2743893 6	0.787	0.17	3.17E-144	7	Wdr75
2.13E-148	0.4173134	0.928	0.229	5.17E-144	7	Srf
3.36E-148	0.2700039 1	0.864	0.199	8.14E-144	7	Mpp6
3.40E-148	0.4974102 1	0.909	0.211	8.24E-144	7	Golim4
3.71E-148	0.6359550 4	0.934	0.216	8.99E-144	7	Pcolce
3.94E-148	0.2937085 2	0.765	0.16	9.55E-144	7	Nectin2
4.32E-148	0.4158492 7	0.809	0.177	1.05E-143	7	Tes
4.32E-148	0.4251525 3	0.886	0.207	1.05E-143	7	Cd151
4.76E-148	0.3405713 3	0.393	0.044	1.16E-143	7	Adamts12
5.77E-148	0.4505295	0.864	0.202	1.40E-143	7	Nfia
6.39E-148	0.2790353 2	0.845	0.188	1.55E-143	7	Mnat1
7.02E-148	0.2873621 9	0.687	0.13	1.70E-143	7	Steap2

1.27E-147	0.30941719	0.875	0.201	3.09E-143	7	Suclg2
1.38E-147	0.43180613	0.864	0.204	3.34E-143	7	Nop56
1.49E-147	0.26164861	0.715	0.137	3.61E-143	7	P3h3
2.12E-147	0.30305714	0.762	0.162	5.13E-143	7	Mesd
2.57E-147	0.27850982	0.468	0.063	6.24E-143	7	Meis2
3.08E-147	0.26855359	0.776	0.168	7.46E-143	7	Heatr5a
3.18E-147	0.36506058	0.884	0.209	7.70E-143	7	Tomm40
3.32E-147	0.27766703	0.709	0.142	8.06E-143	7	Sec61g
4.61E-147	0.34634493	0.803	0.177	1.12E-142	7	Cblb
6.30E-147	0.6094437	0.917	0.234	1.53E-142	7	Taf1d
7.51E-147	0.31209758	0.881	0.208	1.82E-142	7	Acp1
8.19E-147	0.39071136	0.9	0.213	1.99E-142	7	Twsg1
1.12E-146	1.05320888	0.994	0.326	2.72E-142	7	Sptbn1
1.13E-146	0.42396897	0.848	0.181	2.74E-142	7	Fgfr1
1.19E-146	0.409791	0.806	0.18	2.89E-142	7	Dok1
1.45E-146	0.42263203	0.825	0.174	3.52E-142	7	Rcn3
1.54E-146	0.54626223	0.906	0.233	3.75E-142	7	Siva1
1.88E-146	0.41526231	0.892	0.196	4.55E-142	7	Wipi1
2.44E-146	1.13066629	0.695	0.14	5.91E-142	7	Ccl7
2.68E-146	0.42855571	0.892	0.21	6.50E-142	7	Nomo1
4.90E-146	0.38549358	0.864	0.202	1.19E-141	7	Bicd2
7.88E-146	0.25437162	0.756	0.165	1.91E-141	7	Cmc2

9.73E-146	1.18590619	0.997	0.361	2.36E-141	7	11-Sep
1.15E-145	0.28515863	0.831	0.186	2.79E-141	7	Pde4a
1.40E-145	0.40092028	0.828	0.198	3.38E-141	7	Ckap5
2.08E-145	1.39492844	0.953	0.29	5.05E-141	7	Tnc
2.22E-145	0.26592019	0.546	0.088	5.38E-141	7	Clmp
2.43E-145	1.79246421	1	0.776	5.89E-141	7	S100a6
2.81E-145	0.36306487	0.884	0.207	6.82E-141	7	Dlg1
4.86E-145	0.26228326	0.759	0.16	1.18E-140	7	Naa25
6.49E-145	0.39182091	0.886	0.201	1.57E-140	7	Tbrg1
7.65E-145	0.54279662	0.92	0.231	1.86E-140	7	Plod3
1.02E-144	0.32946026	0.704	0.143	2.48E-140	7	Mcm7
1.09E-144	0.34755982	0.856	0.198	2.65E-140	7	Adpgk
1.52E-144	0.43436597	0.864	0.199	3.68E-140	7	Mtap
2.06E-144	0.58939739	0.992	0.242	4.99E-140	7	Rbfox2
2.26E-144	0.60285215	0.961	0.219	5.48E-140	7	Rcn1
3.90E-144	1.02561042	0.989	0.275	9.45E-140	7	Ppic
8.17E-144	0.36549289	0.878	0.21	1.98E-139	7	Shc1
9.79E-144	1.36144607	0.997	0.335	2.37E-139	7	Fstl1
1.13E-143	0.75336267	0.992	0.249	2.73E-139	7	Enah
1.42E-143	0.47555905	0.958	0.246	3.44E-139	7	Galk1
1.62E-143	0.39784401	0.618	0.115	3.94E-139	7	Prkg2

1.94E-143	0.25164473	0.74	0.156	4.70E-139	7	Ahsa1
3.02E-143	0.31658389	0.892	0.215	7.33E-139	7	Thap12
3.10E-143	0.70307977	0.967	0.271	7.51E-139	7	Pgrmc1
8.07E-143	0.35852738	0.659	0.126	1.96E-138	7	Pth1r
1.17E-142	0.69246803	0.931	0.262	2.83E-138	7	Cbx5
1.29E-142	0.36013894	0.889	0.21	3.12E-138	7	Cldnd1
1.30E-142	0.29376995	0.828	0.186	3.16E-138	7	Faf2
1.57E-142	0.29673441	0.709	0.142	3.80E-138	7	P4ha3
1.68E-142	0.30835691	0.837	0.192	4.07E-138	7	Esf1
1.81E-142	0.27468341	0.551	0.092	4.39E-138	7	Fzd2
2.30E-142	1.00706845	0.961	0.293	5.58E-138	7	Slc29a1
3.27E-142	0.55898221	0.884	0.204	7.93E-138	7	Rgs3
5.22E-142	0.74874765	0.936	0.244	1.27E-137	7	Mical2
7.25E-142	1.07831738	0.983	0.301	1.76E-137	7	Tuba1a
7.28E-142	0.34500769	0.806	0.181	1.76E-137	7	Csnk1g1
8.99E-142	0.68453073	0.961	0.238	2.18E-137	7	Ak1
1.36E-141	0.28180778	0.828	0.177	3.29E-137	7	Copz2
1.88E-141	0.52330598	0.928	0.222	4.56E-137	7	Sdc1
2.29E-141	0.66543142	0.97	0.274	5.55E-137	7	Fubp1
2.82E-141	0.67559235	0.947	0.27	6.84E-137	7	Dtymk
2.96E-141	0.28269845	0.873	0.205	7.17E-137	7	Ube3c

3.69E-141	0.94877927	0.953	0.266	8.95E-137	7	Tm4sf1
4.18E-141	0.25064384	0.626	0.114	1.01E-136	7	4632427E13Rik
5.21E-141	0.40969939	0.828	0.183	1.26E-136	7	Ephx1
6.67E-141	0.75769666	0.776	0.168	1.62E-136	7	Gas1
1.17E-140	0.3614105	0.82	0.172	2.85E-136	7	Mxra8
1.51E-140	0.31006153	0.825	0.189	3.66E-136	7	Slc35b1
1.94E-140	0.28122538	0.806	0.179	4.70E-136	7	Cars
2.00E-140	0.39688973	0.906	0.222	4.85E-136	7	Zmat3
2.01E-140	0.55953082	0.958	0.255	4.87E-136	7	Tnk2
3.08E-140	0.29672041	0.87	0.208	7.46E-136	7	Prmt5
3.54E-140	0.3180928	0.886	0.211	8.59E-136	7	Zmpste24
3.96E-140	0.28818677	0.837	0.194	9.61E-136	7	Hsph1
4.10E-140	0.373781	0.881	0.213	9.94E-136	7	Trp53
4.11E-140	0.33469143	0.859	0.201	9.98E-136	7	Zrsr2
6.60E-140	0.45052893	0.917	0.24	1.60E-135	7	Ebna1bp2
7.12E-140	0.40986157	0.748	0.164	1.73E-135	7	Khdrbs3
7.17E-140	0.26217854	0.82	0.19	1.74E-135	7	Hdac1
7.75E-140	0.30929456	0.648	0.125	1.88E-135	7	Nbl1
1.34E-139	0.56102107	0.95	0.259	3.24E-135	7	Stip1
1.72E-139	0.44606345	0.922	0.232	4.17E-135	7	Capn2
2.72E-139	0.36966462	0.884	0.216	6.59E-135	7	Zdhhc5
2.98E-139	0.49210593	0.839	0.187	7.24E-135	7	Mmp23
5.00E-139	1.14913016	0.986	0.335	1.21E-134	7	Loxl3

5.02E-139	0.4346347 7	0.787	0.189	1.22E-134	7	Ccdc34
1.08E-138	0.2630497	0.825	0.189	2.61E-134	7	Prpsap1
1.12E-138	0.2749055	0.759	0.154	2.70E-134	7	Snhg18
1.39E-138	0.5374714 2	0.839	0.191	3.37E-134	7	Phgdh
1.58E-138	1.0630167 2	0.931	0.255	3.83E-134	7	Loxl2
2.28E-138	0.4331292	0.92	0.237	5.52E-134	7	Frmd4a
2.40E-138	0.5603862 4	0.906	0.233	5.82E-134	7	Tsc22d2
4.45E-138	0.3014231	0.881	0.211	1.08E-133	7	Sec11a
5.76E-138	0.8894183 3	0.712	0.157	1.40E-133	7	Adm
5.98E-138	0.3451374 8	0.861	0.195	1.45E-133	7	Slc39a14
6.09E-138	0.4240210 3	0.848	0.192	1.48E-133	7	Olfml2b
8.70E-138	1.4420512 3	1	0.771	2.11E-133	7	Vim
1.38E-137	0.4578983 7	0.753	0.162	3.34E-133	7	Csrp2
2.34E-137	0.3669135	0.693	0.141	5.67E-133	7	Irs1
4.12E-137	0.5711865	0.914	0.236	9.99E-133	7	Klf7
4.17E-137	0.3474860 9	0.875	0.208	1.01E-132	7	Kars
6.02E-137	1.5594289 3	1	0.414	1.46E-132	7	Lox
6.34E-137	0.7780878 8	0.947	0.248	1.54E-132	7	S100a16
6.62E-137	0.3178744 6	0.546	0.093	1.61E-132	7	Kpna2
6.90E-137	0.8632100 7	0.981	0.278	1.67E-132	7	Cnn3
9.36E-137	0.6551695 5	0.975	0.267	2.27E-132	7	Impad1
1.23E-136	0.5144224 8	0.942	0.217	2.99E-132	7	Mrc2
1.54E-136	0.3335392 7	0.9	0.227	3.74E-132	7	Cep170
1.55E-136	0.4954137 6	0.95	0.25	3.76E-132	7	Arhgap5

2.95E-136	0.98646171	0.989	0.339	7.16E-132	7	Ybx3
3.95E-136	0.80878148	0.981	0.287	9.57E-132	7	Flnb
4.78E-136	0.33568276	0.892	0.22	1.16E-131	7	Pcgf3
6.39E-136	0.44593755	0.909	0.231	1.55E-131	7	Wdr43
6.95E-136	0.27906141	0.321	0.029	1.68E-131	7	Hist1h1d
1.00E-135	0.54636683	0.967	0.245	2.42E-131	7	Ttc3
1.02E-135	1.0754251	0.992	0.327	2.48E-131	7	Igfbp7
1.04E-135	0.48817583	0.945	0.258	2.52E-131	7	Lsm6
2.31E-135	0.45599655	0.931	0.227	5.60E-131	7	Trove2
2.53E-135	0.25113879	0.474	0.07	6.13E-131	7	Gm42047
2.69E-135	1.23690523	1	0.383	6.53E-131	7	Nedd4
3.68E-135	0.27548591	0.662	0.132	8.92E-131	7	Ddah2
4.06E-135	0.66243432	0.925	0.226	9.86E-131	7	Cdh11
4.10E-135	0.49394733	0.684	0.14	9.95E-131	7	Cp
4.70E-135	1.30395247	1	0.432	1.14E-130	7	Cald1
5.04E-135	0.32284197	0.573	0.103	1.22E-130	7	Fbln5
5.80E-135	0.31626326	0.861	0.213	1.41E-130	7	Brd3
7.42E-135	0.30467908	0.934	0.243	1.80E-130	7	Pgp
1.40E-134	0.26016919	0.781	0.167	3.40E-130	7	Epha2
1.52E-134	0.37642364	0.837	0.202	3.68E-130	7	Pds5b
1.92E-134	0.28286087	0.618	0.116	4.66E-130	7	Map1a
2.08E-134	0.6237064	0.753	0.165	5.05E-130	7	Ncam1

2.13E-134	0.29625526	0.867	0.212	5.17E-130	7	Pfdn1
2.36E-134	0.27212238	0.834	0.196	5.72E-130	7	Mrpl54
4.90E-134	0.32088926	0.765	0.171	1.19E-129	7	Ier5l
5.31E-134	0.27412451	0.612	0.116	1.29E-129	7	Efnb2
6.00E-134	0.44292433	0.925	0.244	1.45E-129	7	Tnfaip1
6.50E-134	1.33804888	1	0.457	1.58E-129	7	Ckap4
7.13E-134	0.37596586	0.651	0.136	1.73E-129	7	G2e3
7.28E-134	0.62031123	0.961	0.256	1.77E-129	7	Dnajc10
8.17E-134	0.39514838	0.947	0.245	1.98E-129	7	Arhgef12
9.71E-134	0.49396049	0.947	0.246	2.35E-129	7	Tceal8
1.56E-133	0.27774925	0.856	0.207	3.79E-129	7	Ppme1
1.68E-133	0.78025812	0.983	0.298	4.06E-129	7	Ppfibp1
1.95E-133	0.56928882	0.693	0.142	4.72E-129	7	Alpl
2.99E-133	0.3546686	0.92	0.231	7.26E-129	7	Dars
3.22E-133	0.44369047	0.831	0.198	7.80E-129	7	Slc7a1
5.24E-133	0.34015392	0.914	0.233	1.27E-128	7	Eif2b5
5.35E-133	0.27951118	0.853	0.195	1.30E-128	7	Fam114a1
6.18E-133	0.26006102	0.806	0.185	1.50E-128	7	Carhsp1
6.43E-133	0.28045765	0.878	0.222	1.56E-128	7	Otud4
7.95E-133	0.25350528	0.85	0.205	1.93E-128	7	Ccdc127
1.30E-132	0.36440614	0.892	0.227	3.16E-128	7	Slc25a24
1.50E-132	0.29760177	0.889	0.222	3.64E-128	7	Pitpnb

1.68E-132	0.34346677	0.911	0.218	4.07E-128	7	Ece1
1.73E-132	0.77315826	0.958	0.277	4.21E-128	7	Lima1
1.74E-132	0.26029885	0.806	0.189	4.21E-128	7	Tmem245
2.63E-132	0.34175692	0.604	0.115	6.37E-128	7	Grb14
2.75E-132	0.27923911	0.845	0.197	6.68E-128	7	Tusc3
2.80E-132	0.32155277	0.873	0.217	6.80E-128	7	Gps1
3.95E-132	0.5682914	0.753	0.173	9.57E-128	7	Sorbs2
4.43E-132	0.67658003	0.942	0.246	1.07E-127	7	Sox4
4.96E-132	0.37862219	0.911	0.237	1.20E-127	7	Timm17a
8.46E-132	0.28886273	0.784	0.182	2.05E-127	7	Cpox
9.28E-132	0.42594502	0.886	0.216	2.25E-127	7	Med13l
1.17E-131	0.65200514	0.737	0.165	2.84E-127	7	Hes1
1.56E-131	0.26551843	0.875	0.223	3.79E-127	7	Tfdp1
2.05E-131	0.36684177	0.911	0.24	4.98E-127	7	Nudt21
2.71E-131	0.64618662	0.931	0.263	6.57E-127	7	Mybbp1a
2.82E-131	0.62873915	0.964	0.279	6.83E-127	7	Denr
2.93E-131	0.25398668	0.695	0.142	7.10E-127	7	Fkbp7
3.11E-131	0.26674515	0.762	0.174	7.55E-127	7	Smad6
1.20E-130	0.33630691	0.878	0.216	2.91E-126	7	Inf2
2.44E-130	0.25375402	0.825	0.205	5.93E-126	7	Brd8
2.70E-130	0.25004373	0.679	0.141	6.55E-126	7	Aldh18a1
4.81E-130	0.32372487	0.845	0.205	1.17E-125	7	Snhg6

7.16E-130	0.33696577	0.803	0.194	1.73E-125	7	Tcof1
1.18E-129	0.34110183	0.701	0.147	2.86E-125	7	Nupr1
1.19E-129	1.16359577	1	0.547	2.89E-125	7	Tpm4
1.78E-129	0.59528147	0.848	0.217	4.32E-125	7	Ank
2.53E-129	0.25400387	0.828	0.196	6.14E-125	7	Mosmo
4.84E-129	0.65952585	0.95	0.268	1.17E-124	7	Peak1
5.60E-129	0.52541451	0.925	0.25	1.36E-124	7	Ccdc88a
5.80E-129	1.17682773	0.994	0.351	1.41E-124	7	Mfge8
9.50E-129	0.59943544	0.928	0.235	2.30E-124	7	Ext1
1.15E-128	0.41289419	0.914	0.25	2.78E-124	7	Zwint
1.24E-128	0.52646347	0.795	0.201	3.00E-124	7	Stmn1
1.32E-128	0.36729271	0.798	0.176	3.20E-124	7	Ptprd
1.65E-128	0.31165183	0.895	0.233	4.01E-124	7	Ifrd1
1.72E-128	0.25517088	0.568	0.106	4.16E-124	7	Gria3
2.00E-128	0.25284374	0.751	0.168	4.84E-124	7	Akap9
3.00E-128	0.35656469	0.335	0.035	7.27E-124	7	Col15a1
5.42E-128	0.76806787	0.837	0.238	1.32E-123	7	Smc4
1.46E-127	0.27253536	0.834	0.206	3.55E-123	7	Pgm2
1.58E-127	1.43437945	0.992	0.52	3.84E-123	7	Rps2
1.81E-127	0.51396346	0.9	0.247	4.39E-123	7	Enc1
1.85E-127	0.33660461	0.87	0.22	4.48E-123	7	Ftsj3

2.12E-127	0.46075205	0.95	0.273	5.14E-123	7	Dhx9
2.20E-127	0.69844466	0.978	0.268	5.33E-123	7	Maged1
2.22E-127	0.26067112	0.864	0.217	5.39E-123	7	Zc3h18
2.97E-127	0.42077567	0.928	0.245	7.20E-123	7	Ash1l
3.80E-127	0.6565582	0.956	0.285	9.21E-123	7	Eif3j1
4.66E-127	0.30606572	0.886	0.227	1.13E-122	7	Psmd3
8.40E-127	0.27832666	0.873	0.219	2.04E-122	7	Smyd2
1.02E-126	1.06992895	0.992	0.373	2.47E-122	7	Myadm
1.39E-126	0.52594929	0.917	0.234	3.36E-122	7	Unc5b
1.42E-126	0.35101764	0.898	0.236	3.45E-122	7	Usp47
1.93E-126	1.04831959	0.981	0.306	4.67E-122	7	Serpinf1
2.07E-126	0.30265804	0.737	0.173	5.01E-122	7	Topbp1
2.13E-126	0.91993264	0.986	0.364	5.16E-122	7	Eif4g1
2.23E-126	0.42381178	0.512	0.089	5.40E-122	7	Medag
2.33E-126	0.53832826	0.834	0.218	5.64E-122	7	Itga5
1.16E-125	0.59343705	0.848	0.211	2.81E-121	7	Angptl2
1.27E-125	0.31507358	0.909	0.235	3.09E-121	7	Zzef1
1.67E-125	0.26062227	0.742	0.176	4.05E-121	7	Rpa1
1.93E-125	0.3277344	0.917	0.247	4.68E-121	7	Rnps1
2.03E-125	0.71713181	0.731	0.174	4.92E-121	7	Grem1
2.10E-125	0.3270669	0.856	0.217	5.10E-121	7	Nip7
3.88E-125	0.59225269	0.69	0.153	9.41E-121	7	Dkk3
5.48E-125	0.36641323	0.909	0.239	1.33E-120	7	Hars

5.48E-125	0.4136491 6	0.765	0.177	1.33E-120	7	Sema7a
5.63E-125	0.3945894 6	0.762	0.18	1.36E-120	7	Col4a2
6.24E-125	0.9867258 5	0.939	0.276	1.51E-120	7	Errfi1
7.83E-125	1.1431136	1	0.458	1.90E-120	7	Lmna
8.48E-125	0.3003874 3	0.864	0.222	2.06E-120	7	Smarca4
1.17E-124	1.2528137 6	0.997	0.399	2.84E-120	7	Sdc4
1.27E-124	0.5237481 4	0.945	0.263	3.07E-120	7	Stt3b
1.87E-124	0.3002417 2	0.873	0.226	4.54E-120	7	Lpgat1
4.78E-124	0.4081185	0.9	0.247	1.16E-119	7	Lrrfip1
5.51E-124	0.3749171 9	0.898	0.229	1.34E-119	7	Colec12
6.70E-124	0.3293389 1	0.695	0.152	1.62E-119	7	Mcm3
8.81E-124	0.5218870 1	0.942	0.256	2.14E-119	7	Ankrd17
9.43E-124	0.2642879 1	0.909	0.236	2.29E-119	7	Dap3
9.44E-124	0.2686098 1	0.861	0.216	2.29E-119	7	Smarcb1
1.05E-123	0.3325976 8	0.884	0.238	2.55E-119	7	Tcerg1
1.14E-123	0.4682349 2	0.947	0.254	2.76E-119	7	Il6st
1.93E-123	0.7498943 1	0.97	0.286	4.69E-119	7	Ppib
2.31E-123	0.2897844 3	0.615	0.125	5.59E-119	7	Vldlr
2.90E-123	0.2615266 6	0.837	0.207	7.03E-119	7	Cyp20a1
3.62E-123	0.2687959 1	0.878	0.23	8.78E-119	7	Ankrd28
4.71E-123	0.4319959 8	0.706	0.163	1.14E-118	7	Atad2
8.02E-123	0.3182847 1	0.925	0.244	1.94E-118	7	Tcf12

8.95E-123	0.30987078	0.837	0.212	2.17E-118	7	Cebpz
1.15E-122	0.25936483	0.831	0.201	2.79E-118	7	Golga3
1.69E-122	0.26356302	0.776	0.19	4.10E-118	7	Emsy
2.36E-122	0.34620498	0.878	0.232	5.73E-118	7	Tmem120a
5.32E-122	0.36394757	0.889	0.246	1.29E-117	7	Cnih4
5.66E-122	0.36725658	0.928	0.246	1.37E-117	7	Pabpc4
6.78E-122	0.27565139	0.892	0.235	1.64E-117	7	Mrto4
6.87E-122	0.42891405	0.947	0.256	1.67E-117	7	Mydgf
1.24E-121	0.78209964	0.972	0.308	3.00E-117	7	Pdap1
1.27E-121	0.27279293	0.881	0.226	3.08E-117	7	Ero1l
1.34E-121	0.2802672	0.825	0.215	3.24E-117	7	Rfc1
1.99E-121	0.26846406	0.85	0.219	4.83E-117	7	Mbd3
4.40E-121	0.69571506	0.277	0.025	1.07E-116	7	Stc1
1.02E-120	0.34510494	0.911	0.254	2.47E-116	7	Nudc
1.49E-120	0.27401477	0.892	0.225	3.61E-116	7	Sec23a
1.63E-120	0.59292219	0.953	0.267	3.96E-116	7	Zbtb20
2.14E-120	1.41238186	1	0.529	5.18E-116	7	Sparc
2.15E-120	0.32300298	0.939	0.264	5.22E-116	7	Hdac7
2.19E-120	1.09532205	0.994	0.384	5.30E-116	7	Cnn2
4.28E-120	0.31720762	0.889	0.241	1.04E-115	7	Eif2s1
4.79E-120	0.27222594	0.895	0.237	1.16E-115	7	Adss
6.01E-120	0.34354494	0.892	0.239	1.46E-115	7	Rbm28

1.48E-119	1.00258691	0.837	0.248	3.59E-115	7	Col4a1
1.56E-119	0.3244857	0.548	0.102	3.78E-115	7	Pparg
2.13E-119	0.35868967	0.928	0.259	5.17E-115	7	Map3k20
4.22E-119	1.31026195	0.997	0.439	1.02E-114	7	Timp1
4.40E-119	0.41389045	0.947	0.267	1.07E-114	7	Cdk4
4.44E-119	0.25710035	0.892	0.235	1.08E-114	7	Pafah1b2
5.04E-119	1.43056914	0.989	0.557	1.22E-114	7	Gm10076
5.19E-119	0.25080293	0.814	0.192	1.26E-114	7	Slc38a10
5.69E-119	0.37710396	0.889	0.249	1.38E-114	7	Smc1a
5.99E-119	0.40006088	0.917	0.251	1.45E-114	7	Npm3
7.87E-119	0.88232589	0.958	0.283	1.91E-114	7	Cdc42ep3
2.09E-118	0.27527118	0.579	0.116	5.07E-114	7	Fzd5
3.74E-118	0.40324939	0.931	0.277	9.06E-114	7	Snrpd1
4.32E-118	0.41429807	0.609	0.128	1.05E-113	7	Plpp1
4.53E-118	0.42132442	0.947	0.259	1.10E-113	7	Tars
5.65E-118	0.35577268	0.911	0.249	1.37E-113	7	2810004N23Rik
1.44E-117	0.47239777	0.961	0.268	3.49E-113	7	Srpr
1.47E-117	0.49455489	0.934	0.253	3.56E-113	7	Lars
1.54E-117	0.36678407	0.853	0.231	3.74E-113	7	Nolc1
1.79E-117	0.33286552	0.867	0.23	4.34E-113	7	Uba6
2.65E-117	0.29970923	0.859	0.216	6.43E-113	7	Lats2
3.41E-117	0.4623229	0.95	0.288	8.27E-113	7	Cycs
4.81E-117	0.6271117	0.654	0.145	1.17E-112	7	Rps15a-ps8

6.45E-117	0.25529167	0.911	0.243	1.56E-112	7	Cul5
8.77E-117	0.43698864	0.873	0.226	2.13E-112	7	Uba52
1.65E-116	0.32451123	0.875	0.249	3.99E-112	7	Cse1l
1.68E-116	0.28731186	0.889	0.244	4.08E-112	7	Cpsf2
2.47E-116	0.41878852	0.903	0.231	6.00E-112	7	Tmem176a
2.82E-116	0.28127393	0.681	0.154	6.83E-112	7	Yae1d1
2.96E-116	0.95305306	0.994	0.339	7.17E-112	7	Qsox1
3.36E-116	0.59748687	0.972	0.3	8.14E-112	7	Pdlim7
3.44E-116	1.03641925	0.989	0.331	8.34E-112	7	Col5a2
1.64E-115	0.29420518	0.648	0.143	3.98E-111	7	Angptl4
1.94E-115	1.30841668	0.983	0.4	4.69E-111	7	Tagln
2.54E-115	0.26251871	0.895	0.247	6.17E-111	7	Nup50
4.95E-115	0.87522829	0.981	0.335	1.20E-110	7	Plec
5.41E-115	0.35942443	0.925	0.258	1.31E-110	7	Prrc2b
8.48E-115	0.31339582	0.909	0.253	2.06E-110	7	Hras
1.58E-114	0.36778807	0.726	0.172	3.83E-110	7	Klf4
2.75E-114	0.32424405	0.906	0.239	6.66E-110	7	Gfpt1
5.59E-114	0.66046558	0.986	0.33	1.36E-109	7	Rbbp7
6.00E-114	0.76689954	0.975	0.306	1.46E-109	7	Kdelr2
6.56E-114	0.47895814	0.95	0.265	1.59E-109	7	Lman1
7.39E-114	0.31909775	0.903	0.251	1.79E-109	7	Gtpbp4

1.69E-113	0.40653683	0.914	0.262	4.10E-109	7	Emc2
1.77E-113	0.3464678	0.601	0.127	4.29E-109	7	Wisp1
2.87E-113	0.38632959	0.934	0.253	6.95E-109	7	Eif4e2
9.74E-113	0.28618684	0.947	0.273	2.36E-108	7	Snrpc
2.43E-112	0.30322903	0.936	0.26	5.89E-108	7	Bptf
2.49E-112	0.29575903	0.906	0.259	6.04E-108	7	Fkbp3
3.28E-112	0.43628268	0.92	0.28	7.94E-108	7	Xpo1
4.76E-112	0.49610091	0.936	0.276	1.16E-107	7	Gnl3
6.12E-112	0.2500267	0.867	0.228	1.48E-107	7	Golt1b
9.34E-112	0.29203583	0.861	0.228	2.27E-107	7	Arl6ip5
1.14E-111	0.62216822	0.975	0.324	2.75E-107	7	Prkar2a
1.19E-111	0.32597818	0.543	0.11	2.88E-107	7	Gm2115
1.21E-111	1.20979167	0.997	0.581	2.94E-107	7	Actn1
1.50E-111	0.30357194	0.92	0.262	3.63E-107	7	Sav1
2.35E-111	0.48841718	0.936	0.262	5.70E-107	7	Gars
4.19E-111	0.27707639	0.892	0.234	1.02E-106	7	Nfat5
4.27E-111	0.2704068	0.853	0.23	1.04E-106	7	Pcm1
5.63E-111	0.33345314	0.515	0.099	1.37E-106	7	Islr
6.38E-111	0.45887016	0.958	0.284	1.55E-106	7	Zc3h15
7.34E-111	1.12537476	1	0.669	1.78E-106	7	Actn4
7.93E-111	0.79529669	0.986	0.315	1.92E-106	7	Pdia4
1.02E-110	0.36907297	0.864	0.244	2.48E-106	7	Filip1l
1.26E-110	0.39885498	0.837	0.24	3.05E-106	7	Tmpo

2.34E-110	0.32318625	0.909	0.258	5.67E-106	7	Rnf6
4.33E-110	0.67938923	0.983	0.305	1.05E-105	7	Pam
5.60E-110	0.2901103	0.889	0.254	1.36E-105	7	Smc3
1.08E-109	0.57485291	0.956	0.3	2.61E-105	7	Nfic
1.48E-109	1.18414665	1	0.537	3.59E-105	7	Tpm1
4.24E-109	0.47629797	0.95	0.276	1.03E-104	7	Asph
6.23E-109	0.43986495	0.961	0.281	1.51E-104	7	Smurf2
6.93E-109	0.33102722	0.942	0.271	1.68E-104	7	Tbl1x
9.28E-109	0.55430852	0.731	0.189	2.25E-104	7	Gadd45g
1.33E-108	0.27665425	0.357	0.05	3.23E-104	7	Nid2
1.51E-108	0.30179163	0.873	0.244	3.67E-104	7	Ddx24
1.60E-108	0.50859577	0.95	0.289	3.88E-104	7	Sfxn1
1.63E-108	0.46256633	0.44	0.075	3.96E-104	7	Sox9
2.33E-108	0.83543051	0.737	0.193	5.65E-104	7	Fbln2
4.19E-108	0.36484719	0.903	0.244	1.02E-103	7	Tmem176b
4.96E-108	1.57222555	0.992	0.58	1.20E-103	7	Acta2
5.30E-108	1.09342328	0.997	0.694	1.28E-103	7	Ldha
7.62E-108	0.29337165	0.947	0.271	1.85E-103	7	Lap3
1.14E-107	0.42831032	0.945	0.292	2.77E-103	7	Psmd1
1.69E-107	0.28620488	0.939	0.257	4.10E-103	7	Ganab
2.89E-107	0.40533685	0.936	0.266	7.01E-103	7	Ergic1
2.90E-107	0.34230227	0.928	0.267	7.04E-103	7	Psmc5

3.44E-107	0.29396006	0.906	0.262	8.34E-103	7	Fkbp4
4.10E-107	0.28222468	0.958	0.282	9.95E-103	7	Hdac2
1.24E-106	0.27031907	0.936	0.273	3.00E-102	7	Ccar1
2.22E-106	1.8284417	0.468	0.089	5.37E-102	7	Il1rn
2.58E-106	0.292038	0.903	0.237	6.26E-102	7	Pbxip1
3.69E-106	0.75227303	0.986	0.334	8.94E-102	7	Csrp1
5.12E-106	0.55539392	0.967	0.278	1.24E-101	7	Dpysl3
5.17E-106	0.47489685	0.565	0.12	1.25E-101	7	Cenpa
6.67E-106	0.25973506	0.909	0.262	1.62E-101	7	Cnot3
6.95E-106	0.48403684	0.956	0.306	1.69E-101	7	Banf1
7.99E-106	0.56765763	0.939	0.292	1.94E-101	7	Arid5b
8.95E-106	0.25748915	0.889	0.248	2.17E-101	7	Eif3g
9.82E-106	0.91864461	0.994	0.508	2.38E-101	7	Hsp90aa1
1.47E-105	0.26847412	0.914	0.249	3.55E-101	7	Map1lc3a
2.44E-105	0.37211303	0.9	0.259	5.90E-101	7	Ddx1
3.01E-105	0.3037732	0.931	0.274	7.29E-101	7	Cfdp1
3.53E-105	0.31367733	0.958	0.284	8.56E-101	7	Cops5
4.05E-105	0.93196169	0.994	0.372	9.82E-101	7	Hspg2
5.81E-105	0.28409586	0.914	0.268	1.41E-100	7	Ppp1r12a
6.69E-105	0.57165544	0.961	0.307	1.62E-100	7	Foxp1
8.01E-105	0.28954729	0.95	0.269	1.94E-100	7	Sec13
8.09E-105	0.35985315	0.931	0.256	1.96E-100	7	Ltbp3
8.31E-105	0.29690929	0.925	0.27	2.01E-100	7	Cers5

1.50E-104	1.2575062<br>3	0.997	0.578	3.63E-100	7	Tmsb10
1.54E-104	0.4542212<br>2	0.961	0.296	3.73E-100	7	Aimp1
1.77E-104	0.5305595<br>6	0.967	0.321	4.30E-100	7	Snrpg
2.26E-104	0.4158374<br>3	0.95	0.299	5.49E-100	7	Naa15
2.51E-104	0.2563923<br>3	0.535	0.108	6.09E-100	7	Incenp
3.29E-104	0.2833314<br>6	0.934	0.259	7.97E-100	7	Copg1
3.92E-104	0.5281047<br>3	0.975	0.296	9.50E-100	7	Eprs
4.90E-104	0.8954494<br>3	0.986	0.423	1.19E-99	7	Hmgb1
6.02E-104	0.5102385<br>9	0.958	0.311	1.46E-99	7	Ak2
6.21E-104	0.3803445<br>7	0.939	0.286	1.51E-99	7	Rwdd1
1.12E-103	0.4048086<br>6	0.884	0.275	2.72E-99	7	Rangap1
1.48E-103	0.3610682	0.911	0.26	3.60E-99	7	Dag1
1.78E-103	1.0622017<br>6	0.997	0.611	4.32E-99	7	Dstn
2.23E-103	0.3700960<br>7	0.482	0.095	5.42E-99	7	Cspg4
2.74E-103	0.3682383<br>3	0.571	0.126	6.64E-99	7	Arrdc4
3.00E-103	0.3407736<br>4	0.934	0.265	7.27E-99	7	Uso1
3.58E-103	0.3162241<br>6	0.934	0.275	8.68E-99	7	Psma4
4.42E-103	0.4312001<br>6	0.947	0.291	1.07E-98	7	Fyttd1
4.96E-103	0.2889383<br>2	0.892	0.254	1.20E-98	7	Atxn1
5.43E-103	0.2559058<br>2	0.903	0.257	1.32E-98	7	Mrpl57
7.74E-103	0.2996452<br>9	0.729	0.188	1.88E-98	7	Btg2
1.26E-102	0.4973182<br>7	0.956	0.285	3.06E-98	7	Dst

1.67E-102	0.34114649	0.939	0.29	4.04E-98	7	Mrpl12
2.38E-102	0.42821509	0.947	0.305	5.76E-98	7	Smarca5
2.73E-102	0.31742512	0.931	0.286	6.61E-98	7	Egln1
2.73E-102	0.33476461	0.922	0.268	6.62E-98	7	Tmem5
2.92E-102	0.47782763	0.97	0.289	7.09E-98	7	Rcn2
3.52E-102	0.27539652	0.892	0.263	8.53E-98	7	Csnk2a2
4.08E-102	0.76873856	0.961	0.339	9.89E-98	7	Pdlim5
4.62E-102	0.44736152	0.947	0.31	1.12E-97	7	Baz1b
5.06E-102	0.27780008	0.56	0.121	1.23E-97	7	Vdr
8.11E-102	0.26210215	0.886	0.256	1.97E-97	7	Epb41l1
2.47E-101	0.60459632	0.961	0.32	5.98E-97	7	Myo1c
3.22E-101	0.30576201	0.657	0.157	7.81E-97	7	Prrx2
4.11E-101	0.40645301	0.623	0.148	9.97E-97	7	Lurap1l
4.13E-101	0.3877104	0.859	0.242	1.00E-96	7	S1pr1
5.38E-101	0.25148559	0.889	0.25	1.30E-96	7	Samd4b
1.10E-100	0.27370813	0.332	0.046	2.66E-96	7	Gprin3
1.65E-100	1.03001651	0.892	0.281	3.99E-96	7	Cxcl12
2.20E-100	0.76966219	0.994	0.395	5.34E-96	7	Ppp1r14b
5.86E-100	1.00316746	1	0.613	1.42E-95	7	Flna
1.06E-99	0.35804103	0.914	0.268	2.57E-95	7	Hyou1
1.44E-99	0.7171154	0.978	0.347	3.49E-95	7	Ugdh
1.79E-99	0.76932039	0.983	0.392	4.34E-95	7	Cbx3
4.12E-99	0.340752	0.922	0.276	1.00E-94	7	Ankrd11

4.25E-99	0.7509850<br>9	0.975	0.335	1.03E-94	7	Cd81
5.86E-99	0.3106799<br>9	0.928	0.286	1.42E-94	7	Nhp2
7.36E-99	0.3618419<br>9	0.942	0.282	1.78E-94	7	Lsm12
9.28E-99	0.3054270<br>2	0.867	0.24	2.25E-94	7	Dpy19l1
2.83E-98	0.3547716<br>8	0.911	0.278	6.87E-94	7	Mettl9
3.25E-98	0.3550579<br>5	0.695	0.181	7.88E-94	7	Hspb1
3.69E-98	0.4080795<br>3	0.961	0.314	8.95E-94	7	Ptbp1
4.65E-98	0.3128260<br>9	0.92	0.266	1.13E-93	7	Man1a2
5.01E-98	0.4340752<br>2	0.95	0.315	1.22E-93	7	Thoc7
1.04E-97	0.8795953<br>3	0.986	0.412	2.52E-93	7	Ube2s
1.71E-97	0.2844057<br>3	0.584	0.133	4.15E-93	7	Lgr6
1.75E-97	0.3792247<br>5	0.939	0.279	4.25E-93	7	Eif4ebp1
2.21E-97	0.9696433<br>7	1	0.462	5.35E-93	7	Calu
2.37E-97	0.5806597<br>7	0.958	0.315	5.74E-93	7	Mlec
2.41E-97	0.8973964	0.994	0.408	5.85E-93	7	Pdia3
3.12E-97	0.3123375<br>3	0.607	0.142	7.57E-93	7	Mt2
3.39E-97	1.4299327<br>6	0.986	0.546	8.21E-93	7	Cyr61
4.54E-97	0.7670844<br>9	0.983	0.362	1.10E-92	7	Ahcyl1
8.87E-97	0.8054748<br>9	0.983	0.403	2.15E-92	7	Anxa1
9.54E-97	0.2896455<br>8	0.942	0.288	2.31E-92	7	Scoc
3.39E-96	0.3074959<br>1	0.931	0.288	8.21E-92	7	Kmt2e
3.73E-96	1.0674045<br>7	0.992	0.654	9.03E-92	7	Tubb5

6.27E-96	0.34233862	0.925	0.286	1.52E-91	7	Mrps21
6.75E-96	0.45537103	0.981	0.319	1.64E-91	7	Map4
1.81E-95	0.38154159	0.914	0.273	4.38E-91	7	Fosl2
1.89E-95	0.26431529	0.953	0.294	4.59E-91	7	Kctd10
2.06E-95	0.75812859	0.958	0.337	5.00E-91	7	Scaf11
2.78E-95	0.33721559	0.945	0.28	6.75E-91	7	Asap1
3.08E-95	0.33942284	0.945	0.303	7.48E-91	7	Cacybp
3.92E-95	0.78688647	0.92	0.32	9.52E-91	7	Bnip3
4.34E-95	0.26847561	0.95	0.297	1.05E-90	7	Gatad1
5.11E-95	0.37328502	0.355	0.055	1.24E-90	7	Cnn1
5.98E-95	0.84226062	0.994	0.47	1.45E-90	7	Hspd1
6.83E-95	0.49520297	0.964	0.341	1.66E-90	7	Pa2g4
8.14E-95	0.9932777	1	0.582	1.97E-90	7	Bsg
8.90E-95	0.52538561	0.964	0.342	2.16E-90	7	Rdx
9.32E-95	0.52844504	0.975	0.336	2.26E-90	7	Anxa6
1.34E-94	0.39205785	0.939	0.283	3.26E-90	7	Yipf5
1.56E-94	0.37222298	0.958	0.305	3.77E-90	7	Hectd1
1.66E-94	0.27953739	0.512	0.11	4.02E-90	7	Flnc
2.32E-94	0.48874991	0.986	0.332	5.62E-90	7	Eif1ax
3.92E-94	0.25053053	0.947	0.29	9.50E-90	7	Srsf11
4.29E-94	0.30799358	0.947	0.295	1.04E-89	7	Cops6
4.38E-94	0.53415486	0.978	0.36	1.06E-89	7	Snrpf

4.55E-94	0.2996613 8	0.997	0.689	1.10E-89	7	mt-Nd1
6.75E-94	0.8687423 2	0.994	0.472	1.64E-89	7	Rock2
7.74E-94	0.4259909 3	0.85	0.242	1.88E-89	7	Scd2
1.64E-93	0.2948178 2	0.953	0.3	3.97E-89	7	Psma3
3.21E-93	0.8773190 9	1	0.547	7.79E-89	7	Ran
3.54E-93	0.2523052 9	0.457	0.091	8.59E-89	7	Col5a3
3.88E-93	0.3404712 7	0.67	0.178	9.40E-89	7	Fam198b
3.93E-93	0.6285958 8	0.964	0.331	9.53E-89	7	Raph1
5.40E-93	0.2887592 3	0.983	0.381	1.31E-88	7	Palld
7.59E-93	0.9716046 2	1	0.929	1.84E-88	7	Rpl41
1.13E-92	0.8670788 5	0.997	0.784	2.73E-88	7	Lgals1
1.63E-92	0.8871897 9	0.997	0.71	3.95E-88	7	Anxa2
1.84E-92	0.3037223 6	0.914	0.29	4.45E-88	7	Lsm4
1.98E-92	0.3480818 2	0.967	0.303	4.80E-88	7	Nudcd2
2.15E-92	1.0799752 5	0.956	0.361	5.20E-88	7	S100a4
2.81E-92	0.2968385 8	0.928	0.287	6.82E-88	7	Mrpl17
5.09E-92	0.2803392 2	0.936	0.301	1.23E-87	7	Cnot6
7.19E-92	0.8817292 7	0.997	0.502	1.74E-87	7	Eef1g
1.04E-91	0.3807795	0.983	0.325	2.52E-87	7	Psmd6
3.06E-91	0.2855323 1	0.947	0.304	7.41E-87	7	Ptpn11
3.13E-91	1.0138490 6	0.997	0.71	7.59E-87	7	Hsp90b1
3.19E-91	0.5666104 8	0.909	0.288	7.74E-87	7	Ugcg

4.79E-91	0.50256668	0.97	0.328	1.16E-86	7	S100a13
8.53E-91	1.4643295	0.997	0.685	2.07E-86	7	Thbs1
9.74E-91	0.28787675	0.609	0.152	2.36E-86	7	Cryab
4.44E-90	0.38259384	0.956	0.298	1.08E-85	7	Ddost
5.17E-90	0.34031368	0.263	0.032	1.25E-85	7	Slpi
5.18E-90	0.26645285	0.945	0.302	1.26E-85	7	Map2k3
8.40E-90	0.34185408	0.939	0.296	2.04E-85	7	Top2b
2.48E-89	0.39892242	0.97	0.323	6.01E-85	7	Gtf2h5
3.74E-89	0.47531208	0.402	0.076	9.07E-85	7	Pappa
4.10E-89	0.89971114	0.997	0.684	9.95E-85	7	Ncl
4.27E-89	0.29255792	0.618	0.155	1.04E-84	7	2900026A02Rik
6.74E-89	0.29950508	0.925	0.31	1.63E-84	7	H2afv
1.02E-88	0.60902663	0.978	0.383	2.48E-84	7	Kpnb1
1.42E-88	0.80259461	0.994	0.455	3.44E-84	7	Gapdh
1.71E-88	0.27024475	0.922	0.293	4.14E-84	7	Birc6
2.22E-88	0.32526002	0.956	0.321	5.39E-84	7	Immt
4.40E-88	0.32515766	0.956	0.312	1.07E-83	7	Anapc5
4.97E-88	0.33063145	0.961	0.326	1.20E-83	7	Snhg1
5.73E-88	0.30372158	0.945	0.302	1.39E-83	7	Tomm5
6.11E-88	1.059683	0.992	0.511	1.48E-83	7	Rpl35
6.32E-88	0.49990509	0.981	0.352	1.53E-83	7	Ilk
7.74E-88	0.36074723	0.942	0.3	1.88E-83	7	Cdc37

8.21E-88	0.6559904<br>1	0.994	0.436	1.99E-83	7	Tcp1
9.96E-88	0.4401472<br>5	0.972	0.331	2.41E-83	7	Ipo5
1.38E-87	0.3432355<br>8	0.931	0.294	3.34E-83	7	Atp5g1
1.44E-87	0.8170380<br>8	0.889	0.3	3.48E-83	7	Inhba
1.52E-87	0.3306707<br>9	0.964	0.31	3.69E-83	7	Huwe1
1.59E-87	0.4102171<br>5	0.972	0.354	3.85E-83	7	Anp32e
1.76E-87	0.7394188	0.994	0.453	4.27E-83	7	Sfpq
1.91E-87	0.3199559<br>2	0.909	0.276	4.64E-83	7	Atf5
2.08E-87	0.6100024<br>3	0.992	0.37	5.04E-83	7	Rhoc
2.47E-87	0.2635389<br>7	0.9	0.271	5.98E-83	7	Snd1
2.74E-87	0.3218639<br>7	0.92	0.289	6.64E-83	7	Edf1
4.37E-87	0.3375775<br>7	0.255	0.031	1.06E-82	7	Grem2
4.51E-87	0.3787745<br>8	0.953	0.315	1.09E-82	7	Atp5d
6.36E-87	0.4888507<br>7	0.983	0.327	1.54E-82	7	Piezo1
7.29E-87	0.4676983<br>7	0.972	0.351	1.77E-82	7	Dhx15
7.68E-87	0.3301343<br>7	0.97	0.32	1.86E-82	7	Vdac3
9.18E-87	0.3100316<br>5	0.936	0.294	2.23E-82	7	Tmem258
1.36E-86	1.0153728<br>6	0.997	0.611	3.29E-82	7	Fn1
1.42E-86	0.3127296<br>6	0.95	0.318	3.45E-82	7	Mapk6
1.71E-86	0.2845755<br>5	0.939	0.306	4.14E-82	7	Atic
3.51E-86	0.5372903<br>6	0.986	0.35	8.51E-82	7	Ddb1
4.22E-86	0.4414256<br>4	0.953	0.334	1.02E-81	7	Tmed5

4.42E-86	0.31185106	0.958	0.316	1.07E-81	7	Aco2
4.66E-86	0.41183592	0.981	0.332	1.13E-81	7	Strn3
5.62E-86	0.59774344	0.989	0.39	1.36E-81	7	Cdv3
5.68E-86	0.61039869	0.357	0.062	1.38E-81	7	Cyp26b1
7.36E-86	0.99216364	0.756	0.278	1.78E-81	7	Hmgb2
7.62E-86	0.26124241	0.922	0.292	1.85E-81	7	Dnajb11
8.04E-86	0.90852238	0.994	0.528	1.95E-81	7	Rps18
8.51E-86	0.29008971	0.95	0.283	2.06E-81	7	Bmp1
1.05E-85	0.34205764	0.964	0.314	2.54E-81	7	Magt1
1.08E-85	0.55764546	0.994	0.377	2.63E-81	7	Cct3
1.46E-85	0.85244159	0.992	0.513	3.55E-81	7	Set
1.52E-85	0.47621958	0.97	0.345	3.70E-81	7	Psmd11
1.71E-85	0.46487237	0.983	0.349	4.14E-81	7	Tnpo3
2.71E-85	0.27575515	0.934	0.314	6.57E-81	7	Smc6
2.74E-85	0.36806042	0.953	0.327	6.64E-81	7	Raly
4.06E-85	0.34373763	0.28	0.038	9.83E-81	7	Sox11
5.56E-85	0.63645598	0.975	0.365	1.35E-80	7	Rpl13a
1.09E-84	0.4408556	0.939	0.322	2.64E-80	7	Emp1
1.39E-84	0.42168611	0.939	0.321	3.37E-80	7	Gpx4
2.10E-84	0.29184276	0.906	0.268	5.10E-80	7	Tsc22d1
2.58E-84	0.26660603	0.892	0.3	6.25E-80	7	Lsm5
3.06E-84	0.32707288	0.97	0.31	7.41E-80	7	Nenf

6.86E-84	0.25434347	0.956	0.317	1.66E-79	7	Pdcl3
1.93E-83	0.29057951	0.942	0.311	4.69E-79	7	Prpf4b
3.03E-83	0.2747864	0.934	0.325	7.34E-79	7	Srsf7
3.15E-83	0.63323419	0.981	0.377	7.63E-79	7	Slc25a4
3.92E-83	0.3058031	0.956	0.316	9.50E-79	7	Utp11
7.38E-83	0.30994262	0.947	0.301	1.79E-78	7	Ssr4
1.90E-82	0.29337542	0.465	0.101	4.60E-78	7	Mcam
2.24E-82	0.34462977	0.906	0.285	5.44E-78	7	Rb1cc1
2.41E-82	0.26370365	0.945	0.311	5.83E-78	7	Oat
2.81E-82	0.28923877	0.947	0.316	6.82E-78	7	Dcun1d5
3.66E-82	0.7784925	1	0.682	8.88E-78	7	Serbp1
4.20E-82	0.70657698	0.662	0.193	1.02E-77	7	Rpl39l
5.08E-82	0.49155887	0.975	0.361	1.23E-77	7	Ddx21
7.66E-82	0.33380147	0.956	0.322	1.86E-77	7	Rnf11
1.26E-81	0.81844828	1	0.608	3.06E-77	7	Myh9
1.32E-81	0.86844624	0.986	0.452	3.20E-77	7	Rpl36
1.35E-81	0.31342105	0.947	0.312	3.28E-77	7	Abcc1
1.75E-81	0.52181794	0.972	0.356	4.23E-77	7	Mrpl52
1.76E-81	0.51295964	0.981	0.338	4.27E-77	7	Prkcsh
1.78E-81	0.25441871	0.939	0.323	4.32E-77	7	Ddx39
2.61E-81	0.50977099	0.967	0.347	6.34E-77	7	Tspan4
2.63E-81	0.31883757	0.964	0.326	6.37E-77	7	Phb2
3.53E-81	0.2619017	0.343	0.061	8.56E-77	7	Slc38a4

3.54E-81	0.62682002	0.992	0.433	8.58E-77	7	Hnrnpa1
4.67E-81	0.30329659	0.975	0.344	1.13E-76	7	Khsrp
5.16E-81	0.68549247	0.992	0.488	1.25E-76	7	S100a11
5.16E-81	0.33714617	0.823	0.24	1.25E-76	7	Mmp2
7.33E-81	0.85640722	0.997	0.685	1.78E-76	7	Mif
8.20E-81	0.48458523	0.992	0.372	1.99E-76	7	Lrrc59
1.65E-80	0.40795119	0.97	0.331	4.00E-76	7	Uqcc2
2.59E-80	0.6377978	0.986	0.465	6.28E-76	7	Srsf3
3.68E-80	0.28105572	0.953	0.321	8.92E-76	7	Tnrc6a
4.37E-80	0.44722348	0.964	0.346	1.06E-75	7	Smarcc1
8.50E-80	0.72662433	0.994	0.499	2.06E-75	7	Hmgn1
1.73E-79	0.250206	0.945	0.318	4.20E-75	7	Chp1
1.83E-79	0.33833222	0.892	0.302	4.44E-75	7	H1f0
2.21E-79	0.91523	1	0.689	5.35E-75	7	Itgb1
7.97E-79	0.37768226	0.958	0.359	1.93E-74	7	Usmg5
1.07E-78	0.58762869	0.978	0.341	2.60E-74	7	Col5a1
2.20E-78	0.2628278	0.382	0.077	5.33E-74	7	Enpp2
2.57E-78	0.27768004	0.931	0.302	6.23E-74	7	Iars
3.19E-78	0.43748023	0.978	0.368	7.74E-74	7	Psmc3
4.11E-78	0.3088613	0.95	0.323	9.96E-74	7	Mycbp2
4.76E-78	0.25307429	0.49	0.117	1.15E-73	7	Inhbb
7.43E-78	0.67165714	0.994	0.491	1.80E-73	7	Clic4
7.62E-78	0.93343097	1	0.791	1.85E-73	7	Rps20

1.27E-77	0.4378716<br>9	0.997	0.366	3.08E-73	7	Ccnd2
1.32E-77	0.7716641<br>5	1	0.762	3.19E-73	7	Npm1
1.51E-77	0.5465160<br>2	0.975	0.425	3.65E-73	7	Snrpe
4.88E-77	0.5679854<br>3	0.978	0.436	1.18E-72	7	Ranbp1
6.52E-77	0.3555518	0.967	0.358	1.58E-72	7	Sae1
7.13E-77	0.2988650<br>9	0.942	0.313	1.73E-72	7	Sec63
7.59E-77	0.6356535<br>4	0.994	0.422	1.84E-72	7	Axl
8.33E-77	0.2766892<br>8	0.95	0.313	2.02E-72	7	Nktr
9.08E-77	0.8346208<br>3	1	0.643	2.20E-72	7	Rpl13
9.79E-77	0.4964755<br>7	0.992	0.404	2.37E-72	7	Gspt1
1.09E-76	0.3526886<br>8	0.972	0.327	2.64E-72	7	Gorasp2
1.22E-76	0.3376614<br>3	0.407	0.087	2.97E-72	7	Spata7
1.92E-76	0.3971343<br>4	0.961	0.376	4.66E-72	7	Alyref
2.86E-76	0.8964847<br>5	1	0.639	6.93E-72	7	Ahnak
3.90E-76	0.8888295<br>7	0.997	0.763	9.46E-72	7	Rpl35a
4.04E-76	0.5003759<br>8	0.972	0.366	9.80E-72	7	S100a10
7.24E-76	0.5179989	0.992	0.396	1.76E-71	7	Prmt1
1.07E-75	0.8993742<br>9	0.997	0.578	2.59E-71	7	Rpl6
1.25E-75	0.6928927<br>8	0.997	0.519	3.03E-71	7	Cct6a
2.10E-75	0.3656606<br>5	0.981	0.349	5.08E-71	7	Ubr5
2.13E-75	0.3798388<br>7	0.967	0.363	5.17E-71	7	Trim28
2.92E-75	0.3387662<br>4	0.953	0.32	7.08E-71	7	Tmed9

3.74E-75	0.33945062	0.46	0.108	9.06E-71	7	Has2
6.15E-75	0.30844424	0.97	0.348	1.49E-70	7	Polr2m
7.58E-75	0.26723343	0.931	0.334	1.84E-70	7	Arpp19
1.59E-74	0.48762709	0.978	0.395	3.86E-70	7	Pbrm1
2.35E-74	0.34187688	0.717	0.228	5.69E-70	7	Cdk1
3.52E-74	0.69621136	0.983	0.43	8.54E-70	7	Rrbp1
4.08E-74	0.84368239	0.994	0.571	9.90E-70	7	Rps19
4.75E-74	1.66929783	0.967	0.576	1.15E-69	7	Ctgf
5.84E-74	0.31542598	0.947	0.329	1.42E-69	7	Ndufb9
6.14E-74	0.3442917	0.95	0.308	1.49E-69	7	Selenow
6.39E-74	0.56539538	0.997	0.432	1.55E-69	7	Celf1
7.79E-74	0.71311665	1	0.973	1.89E-69	7	Actg1
1.10E-73	0.71566431	1	0.643	2.67E-69	7	Pkm
1.78E-73	0.80527699	0.997	0.675	4.31E-69	7	Rps27l
1.84E-73	0.40813151	0.97	0.361	4.46E-69	7	Rsl1d1
1.85E-73	0.83621426	0.994	0.69	4.49E-69	7	Tuba1b
2.14E-73	0.35211571	0.981	0.343	5.20E-69	7	Arcn1
2.51E-73	0.49551649	0.978	0.351	6.10E-69	7	Txndc5
3.34E-73	0.34074697	0.989	0.37	8.10E-69	7	Pdcd5
3.37E-73	0.36442466	0.945	0.348	8.18E-69	7	Eef1d
4.44E-73	0.25288128	0.936	0.316	1.08E-68	7	Sec62
4.70E-73	0.32086962	0.958	0.343	1.14E-68	7	1110004F10Rik

6.08E-73	0.45979718	0.967	0.392	1.47E-68	7	Ssrp1
7.40E-73	1.00178994	1	0.641	1.79E-68	7	Hspa5
7.47E-73	0.25775074	0.975	0.333	1.81E-68	7	Srp19
1.12E-72	0.76713095	0.994	0.475	2.71E-68	7	Rpl12
1.13E-72	0.41360799	0.983	0.384	2.74E-68	7	Abce1
1.58E-72	0.61929251	0.981	0.424	3.82E-68	7	Rpl21
1.71E-72	0.76675505	0.997	0.554	4.14E-68	7	Tpi1
2.50E-72	0.41155248	0.981	0.387	6.07E-68	7	Hnrnpd
3.19E-72	0.25081791	0.97	0.347	7.73E-68	7	Tsen34
4.49E-72	0.69041556	0.989	0.523	1.09E-67	7	Hspe1
6.25E-72	0.42004492	0.59	0.167	1.52E-67	7	Ptgs2
7.47E-72	0.3223561	0.964	0.34	1.81E-67	7	Zyx
8.72E-72	0.2774016	0.942	0.337	2.11E-67	7	Fbl
9.27E-72	0.49074562	0.997	0.484	2.25E-67	7	Fkbp1a
9.81E-72	0.58084458	0.961	0.427	2.38E-67	7	Cox7c
1.31E-71	0.36184937	0.961	0.343	3.17E-67	7	Eif1a
1.48E-71	0.26705332	0.942	0.318	3.59E-67	7	Tmem234
1.60E-71	0.2989541	0.967	0.327	3.88E-67	7	Sar1a
1.89E-71	0.2502831	0.95	0.314	4.58E-67	7	Trim35
1.98E-71	0.4143298	0.975	0.346	4.81E-67	7	Stt3a
4.09E-71	0.56903399	0.981	0.452	9.92E-67	7	G3bp1
6.73E-71	0.37294937	0.978	0.384	1.63E-66	7	Purb
7.65E-71	0.45984586	0.992	0.352	1.86E-66	7	Glg1

9.19E-71	0.40666067	0.967	0.34	2.23E-66	7	Ifitm3
1.86E-70	0.29477963	0.97	0.34	4.52E-66	7	Rab6a
1.92E-70	0.27358543	0.956	0.348	4.66E-66	7	Txnl1
2.30E-70	0.43277014	0.981	0.359	5.57E-66	7	Nudt4
2.37E-70	0.57196338	0.967	0.404	5.75E-66	7	Ccng1
7.35E-70	0.31236674	0.898	0.318	1.78E-65	7	Mdm2
8.56E-70	1.85118375	0.989	0.642	2.08E-65	7	Serpine1
1.42E-69	0.49962843	0.978	0.359	3.44E-65	7	Ostc
3.78E-69	0.42368866	0.978	0.405	9.16E-65	7	Calm3
6.98E-69	0.34760574	0.961	0.36	1.69E-64	7	Elob
7.52E-69	0.36986415	0.989	0.374	1.82E-64	7	C1qbp
8.27E-69	0.41926901	0.989	0.408	2.01E-64	7	Ywhaq
9.20E-69	0.31145814	0.956	0.34	2.23E-64	7	Dnajc3
9.74E-69	0.39964725	0.997	0.829	2.36E-64	7	mt-Rnr2
1.20E-68	0.84089093	0.961	0.463	2.90E-64	7	Cdkn1a
1.67E-68	0.31945013	0.978	0.359	4.05E-64	7	Cand1
1.74E-68	0.25054837	0.953	0.341	4.22E-64	7	Glrx3
1.81E-68	0.33305878	0.956	0.36	4.38E-64	7	Bclaf1
2.11E-68	0.3483784	0.964	0.379	5.11E-64	7	Srsf1
3.19E-68	0.28277158	0.975	0.366	7.74E-64	7	Psmc6
3.39E-68	0.32645174	0.975	0.375	8.23E-64	7	Rbm8a
3.58E-68	0.28701812	0.975	0.375	8.69E-64	7	Ctnna1

4.47E-68	0.64491065	0.994	0.492	1.08E-63	7	Map4k4
5.44E-68	0.3911764	0.97	0.381	1.32E-63	7	Tomm70a
2.18E-67	0.60545085	1	0.884	5.29E-63	7	Rpsa
7.47E-67	0.79002795	0.997	0.561	1.81E-62	7	Pdia6
1.17E-66	0.62374412	0.997	0.45	2.83E-62	7	Hdlbp
1.92E-66	0.30408116	0.953	0.339	4.66E-62	7	Rpl15
2.56E-66	0.4116604	0.967	0.379	6.21E-62	7	Mat2a
3.21E-66	0.81889358	0.992	0.641	7.78E-62	7	Rps27
6.95E-66	0.51506793	0.989	0.471	1.68E-61	7	Hnrnph1
9.36E-66	0.26665084	0.95	0.346	2.27E-61	7	Tmem167
1.23E-65	0.33007235	0.967	0.364	2.98E-61	7	Tulp4
1.26E-65	0.63803678	0.994	0.478	3.05E-61	7	Rps7
2.43E-65	0.44045479	0.986	0.387	5.89E-61	7	Tnrc18
4.20E-65	0.75754391	1	0.723	1.02E-60	7	Pgk1
4.52E-65	0.33706139	0.964	0.375	1.10E-60	7	Mrpl33
4.69E-65	0.5306377	0.95	0.369	1.14E-60	7	Ecm1
4.73E-65	0.66500064	0.994	0.61	1.15E-60	7	Eif2s2
1.01E-64	0.70772069	0.992	0.526	2.46E-60	7	Rpl36a
1.87E-64	0.35924643	0.964	0.365	4.53E-60	7	Mbnl2
1.88E-64	0.69064269	0.994	0.668	4.55E-60	7	Rpl14
2.50E-64	0.70733203	0.992	0.523	6.07E-60	7	P4hb
4.20E-64	0.25291003	0.953	0.362	1.02E-59	7	Eif3l
1.40E-63	0.42645163	0.997	0.37	3.39E-59	7	Crtap

1.41E-63	0.5937751	0.997	0.507	3.42E-59	7	Pebp1
3.80E-63	0.54233441	0.994	0.533	9.22E-59	7	Hnrnpa0
3.94E-63	0.34110744	0.967	0.366	9.54E-59	7	Uba1
5.64E-63	0.36570275	0.994	0.42	1.37E-58	7	Etf1
7.41E-63	0.58273314	0.994	0.46	1.80E-58	7	Hif1a
9.39E-63	0.71277537	0.997	0.696	2.28E-58	7	Rpl37
1.51E-62	0.56865669	1	0.575	3.65E-58	7	Pgam1
2.19E-62	0.71496216	0.992	0.525	5.32E-58	7	Rps15a
2.64E-62	0.70112716	0.989	0.564	6.39E-58	7	Rps17
2.70E-62	0.49599338	0.997	0.849	6.55E-58	7	Ppia
5.27E-62	0.61645272	0.986	0.471	1.28E-57	7	Rpl10
8.99E-62	0.69745642	0.989	0.553	2.18E-57	7	Rpl34
2.26E-61	0.49661271	0.983	0.431	5.47E-57	7	Prrc2c
2.99E-61	0.58754398	0.994	0.606	7.25E-57	7	Tagln2
3.71E-61	0.75214265	0.975	0.464	8.99E-57	7	Cited2
5.54E-61	0.30248079	0.92	0.386	1.34E-56	7	Dek
6.56E-61	0.40678608	0.986	0.428	1.59E-56	7	Metap2
1.20E-60	0.68793133	1	0.846	2.91E-56	7	Rpl38
1.76E-60	0.66033022	1	0.87	4.27E-56	7	Rps12
2.64E-60	0.70214419	0.997	0.826	6.40E-56	7	Rps28
4.16E-60	0.67393234	0.989	0.556	1.01E-55	7	Rpl18
2.93E-59	0.56819291	0.994	0.546	7.10E-55	7	Nucks1

3.36E-59	0.64838243	0.989	0.536	8.14E-55	7	Rpl9
4.54E-59	0.29560685	0.983	0.358	1.10E-54	7	Sec31a
5.47E-59	0.47715904	0.994	0.406	1.33E-54	7	P4ha1
7.25E-59	0.47777168	0.978	0.415	1.76E-54	7	Rbpj
1.41E-58	0.53396619	1	0.676	3.42E-54	7	Ywhae
2.37E-58	0.40971516	0.992	0.426	5.74E-54	7	Atp2a2
3.33E-58	0.66791386	1	0.641	8.07E-54	7	Rps23
2.51E-57	0.26677408	0.967	0.404	6.09E-53	7	Jpt1
2.75E-57	0.43041589	0.983	0.423	6.66E-53	7	Mtdh
3.26E-57	0.32449175	0.978	0.407	7.91E-53	7	Lims1
8.72E-57	0.50643045	0.997	0.478	2.12E-52	7	Eif3e
1.03E-56	0.68358655	0.997	0.632	2.51E-52	7	Rpl28
1.25E-56	0.51730975	0.981	0.466	3.02E-52	7	Rps13
1.70E-56	0.78361412	1	0.764	4.12E-52	7	Col1a2
1.08E-55	0.3494967	0.934	0.381	2.62E-51	7	Arl5a
1.14E-55	0.4376007	1	0.997	2.75E-51	7	Actb
1.15E-55	0.33201954	0.975	0.385	2.78E-51	7	Dad1
2.68E-55	0.33680272	0.97	0.397	6.50E-51	7	Rexo2
3.84E-55	0.35327528	0.648	0.221	9.31E-51	7	Egr1
4.05E-55	0.73699507	0.997	0.906	9.82E-51	7	Malat1
7.89E-55	0.28389819	0.975	0.387	1.91E-50	7	Nsa2
8.83E-55	0.42641784	0.983	0.437	2.14E-50	7	Manf

1.10E-54	0.60230408	0.992	0.626	2.66E-50	7	Rpl7a
1.46E-54	0.33125963	0.992	0.45	3.53E-50	7	Tra2b
1.86E-54	0.71273518	0.759	0.29	4.51E-50	7	Ccl2
1.92E-54	0.60969027	0.997	0.555	4.65E-50	7	Anxa3
2.60E-54	0.27263566	0.972	0.42	6.30E-50	7	Abracl
4.64E-54	0.56240406	1	0.906	1.13E-49	7	Rps8
7.53E-54	0.34229301	0.981	0.429	1.82E-49	7	Cct5
1.42E-53	0.41245194	0.994	0.464	3.43E-49	7	Prelid1
1.65E-53	0.35632065	0.983	0.442	4.01E-49	7	Psma7
1.70E-53	0.52279027	0.994	0.581	4.12E-49	7	Hnrnpab
2.30E-53	0.36013705	0.992	0.412	5.57E-49	7	Arf4
5.29E-53	0.45734371	0.997	0.622	1.28E-48	7	Txn1
5.64E-53	0.53457269	0.989	0.464	1.37E-48	7	Ssr1
8.04E-53	0.40340702	0.986	0.496	1.95E-48	7	Ptges3
1.07E-52	0.25035117	0.975	0.412	2.59E-48	7	Rbm25
1.62E-52	0.33987915	0.975	0.426	3.92E-48	7	Zfp207
1.92E-52	0.26641205	0.983	0.414	4.64E-48	7	Mdh2
2.03E-52	0.44997575	0.989	0.524	4.91E-48	7	Hnrnpa3
2.92E-52	0.4540964	1	0.901	7.08E-48	7	Hsp90ab1
3.26E-52	0.30052258	0.981	0.433	7.90E-48	7	Vdac1
3.63E-52	0.4068857	0.981	0.465	8.81E-48	7	Bola2
3.83E-52	0.60764248	0.992	0.516	9.28E-48	7	Aplp2

4.03E-52	0.25251036	0.975	0.41	9.76E-48	7	Eif3b
6.79E-52	0.57955993	0.992	0.574	1.65E-47	7	Dynll1
2.19E-51	0.30719057	0.983	0.441	5.31E-47	7	7-Sep
4.81E-51	0.54869225	1	0.648	1.17E-46	7	Rps11
5.49E-51	0.56487596	0.997	0.49	1.33E-46	7	Mmp14
1.58E-50	0.52216225	0.992	0.517	3.83E-46	7	Rpl17
5.30E-50	0.41929184	0.992	0.489	1.29E-45	7	Uqcrq
1.04E-49	0.40523317	0.986	0.465	2.53E-45	7	Tln1
1.14E-49	0.4861338	0.997	0.582	2.78E-45	7	Hdgf
1.21E-49	0.53013846	1	0.627	2.93E-45	7	Fus
1.27E-49	0.27039477	0.989	0.435	3.07E-45	7	Snrpd2
1.47E-49	0.43125211	0.986	0.462	3.56E-45	7	Sec61b
4.38E-49	0.50067958	0.997	0.593	1.06E-44	7	Rpl4
8.69E-49	0.32782162	0.989	0.432	2.11E-44	7	Slc38a2
8.76E-49	0.3779073	0.972	0.463	2.12E-44	7	Rpl31
2.11E-48	0.31530774	0.989	0.472	5.11E-44	7	Atp5k
2.42E-48	0.52107723	1	0.595	5.86E-44	7	Rpl27a
2.91E-48	0.45318867	0.997	0.891	7.06E-44	7	Myl6
4.44E-48	0.38764032	0.983	0.423	1.08E-43	7	Cnih1
7.88E-48	0.40358151	0.997	0.526	1.91E-43	7	Hint1
1.63E-47	0.57293183	1	0.708	3.96E-43	7	Rps16
3.05E-47	0.33541181	0.994	0.495	7.39E-43	7	Cnbp

6.77E-47	0.31139278	0.986	0.448	1.64E-42	7	Mrfap1
2.57E-46	0.33998027	0.989	0.43	6.23E-42	7	Tns3
6.01E-46	0.40485579	0.983	0.502	1.46E-41	7	Tuba1c
8.38E-46	0.49376513	0.992	0.543	2.03E-41	7	Rpl10a
1.62E-45	0.29816912	0.986	0.473	3.93E-41	7	Mapre1
2.14E-45	0.26386072	0.975	0.419	5.19E-41	7	Sfr1
4.24E-45	0.27728411	0.983	0.453	1.03E-40	7	Cct2
5.65E-45	0.33412411	0.989	0.478	1.37E-40	7	Rbm39
5.83E-45	0.32951755	0.983	0.463	1.41E-40	7	Nars
1.57E-44	0.50266074	1	0.513	3.81E-40	7	Rpn1
2.00E-44	0.41228802	0.997	0.657	4.85E-40	7	Nap1l1
2.27E-44	0.26195125	0.978	0.429	5.51E-40	7	Larp1
2.35E-44	0.3171672	0.97	0.475	5.70E-40	7	Hnrnpm
4.07E-44	0.35964607	0.989	0.5	9.87E-40	7	Eif3c
5.84E-44	0.47069695	0.997	0.612	1.42E-39	7	Nme1
8.77E-44	0.51399339	0.554	0.202	2.13E-39	7	Postn
1.35E-43	0.3919735	0.989	0.436	3.27E-39	7	H13
1.98E-43	0.48548709	0.994	0.607	4.81E-39	7	Rpl29
2.30E-43	0.51985789	1	0.749	5.58E-39	7	Rplp0
8.00E-43	0.48900081	0.997	0.684	1.94E-38	7	Eif5a
1.10E-42	0.34269532	0.986	0.495	2.67E-38	7	Rpl5
1.39E-42	0.46301687	0.997	0.838	3.36E-38	7	Rpl39

3.77E-42	0.28995862	0.994	0.488	9.15E-38	7	Syncrip
7.84E-42	0.5370176	0.986	0.509	1.90E-37	7	Id3
1.09E-41	0.36230946	0.992	0.485	2.63E-37	7	Ccnd1
1.39E-41	0.81644381	1	0.93	3.36E-37	7	Spp1
2.99E-41	0.27803785	0.975	0.462	7.26E-37	7	Srrm2
3.97E-41	0.42605107	0.997	0.71	9.62E-37	7	Gnas
1.02E-40	0.26117225	0.986	0.425	2.47E-36	7	Fndc3b
1.41E-40	0.45479354	1	0.923	3.41E-36	7	Rpl37a
2.31E-39	0.43344212	0.961	0.574	5.59E-35	7	H2afz
1.13E-38	0.31562259	0.997	0.533	2.75E-34	7	Bzw1
1.13E-38	0.38835203	0.983	0.576	2.75E-34	7	Rpl30
1.78E-38	0.39753126	0.986	0.573	4.32E-34	7	Rpl11
4.40E-38	0.32208747	0.989	0.508	1.07E-33	7	Chd4
1.76E-37	0.44079596	0.997	0.723	4.28E-33	7	Rps3
2.02E-37	0.40247314	0.992	0.493	4.91E-33	7	Colgalt1
2.05E-37	0.33006809	0.986	0.515	4.98E-33	7	Rps6
2.23E-37	0.33039774	0.986	0.445	5.41E-33	7	Wls
2.41E-37	0.47078672	0.997	0.793	5.84E-33	7	Rps10
3.19E-37	0.26462858	0.989	0.475	7.74E-33	7	Psmb4
3.22E-37	0.4355294	0.482	0.173	7.81E-33	7	Serpinb2
5.66E-37	0.50641394	1	0.689	1.37E-32	7	Calr
2.59E-36	0.32492545	0.992	0.533	6.28E-32	7	Prrc2a

5.07E-36	0.30006492	0.978	0.484	1.23E-31	7	Rpl27
5.66E-36	0.32797945	0.997	0.607	1.37E-31	7	Hnrnpu
6.97E-36	0.36967894	0.994	0.565	1.69E-31	7	Uqcr11
1.12E-35	0.44770965	0.994	0.658	2.72E-31	7	Rpl18a
2.24E-35	0.27831155	0.986	0.472	5.43E-31	7	Swi5
3.46E-34	0.35346882	0.997	0.56	8.38E-30	7	Morf4l2
3.47E-34	0.40342395	0.992	0.624	8.42E-30	7	Rpl26
3.94E-34	0.33522448	1	0.926	9.55E-30	7	Rplp2
5.86E-34	0.28289393	0.992	0.618	1.42E-29	7	Anp32b
6.13E-34	0.39343176	0.997	0.714	1.49E-29	7	Eif4a1
1.03E-33	0.37924575	0.997	0.75	2.51E-29	7	Rpl22
1.71E-33	0.35512293	1	0.829	4.14E-29	7	Rpl8
1.73E-33	0.30226429	0.997	0.648	4.20E-29	7	Msn
2.07E-33	0.40492847	0.994	0.629	5.02E-29	7	Hspa9
3.34E-33	0.35439148	0.997	0.654	8.11E-29	7	Wdr1
4.16E-33	0.40701844	0.994	0.661	1.01E-28	7	Ptms
2.19E-32	0.34320292	0.997	0.578	5.31E-28	7	Rpl7
5.59E-32	0.41730712	0.997	0.623	1.36E-27	7	Cd63
1.07E-31	0.37353715	0.997	0.556	2.59E-27	7	Timp2
1.49E-31	0.31340508	0.997	0.907	3.60E-27	7	Rps24
2.13E-31	0.30717274	0.989	0.535	5.17E-27	7	Tmed2

5.61E-31	0.35656223	1	0.671	1.36E-26	7	Atp5g3
1.49E-30	0.33431734	0.997	0.779	3.62E-26	7	Eif4g2
1.95E-30	0.36967107	0.997	0.63	4.74E-26	7	Iqgap1
2.89E-30	0.31203678	0.992	0.561	7.00E-26	7	Tomm20
3.74E-30	0.3843059	0.997	0.707	9.06E-26	7	Rpl23a
4.13E-30	0.27112172	0.997	0.597	1.00E-25	7	Rbms1
5.31E-30	0.37236015	1	0.77	1.29E-25	7	Rps3a1
2.95E-29	0.38982826	1	0.772	7.14E-25	7	Eef1b2
2.16E-28	0.31073261	0.994	0.602	5.24E-24	7	Rpl3
3.37E-28	0.25298121	0.997	0.557	8.18E-24	7	Gas5
4.36E-28	0.33757211	0.997	0.662	1.06E-23	7	Btf3
4.79E-28	0.32977193	0.994	0.534	1.16E-23	7	Rpn2
7.67E-28	0.3086429	1	0.661	1.86E-23	7	Csnk1a1
1.43E-27	0.26859601	0.997	0.517	3.46E-23	7	Surf4
1.69E-27	0.25142035	1	0.925	4.09E-23	7	Rps29
6.28E-27	0.27754319	1	0.955	1.52E-22	7	Eef1a1
8.34E-27	0.42459474	0.997	0.73	2.02E-22	7	Rtn4
1.26E-26	0.32868976	0.997	0.93	3.05E-22	7	Ptma
9.13E-25	0.30827799	1	0.77	2.21E-20	7	Aldoa
1.33E-24	0.26177101	1	0.658	3.23E-20	7	Naca
3.85E-24	0.25368428	1	0.871	9.33E-20	7	Rpl23
9.82E-24	0.29746169	1	0.821	2.38E-19	7	Rps27a

1.09E-23	0.27985598	1	0.775	2.65E-19	7	Eif1
1.31E-22	0.29169987	1	0.623	3.17E-18	7	Ssr3
7.65E-22	0.2754208	1	0.897	1.85E-17	7	Rps21
7.81E-22	0.27099735	1	0.824	1.89E-17	7	Rps4x
1.39E-19	0.45714299	0.986	0.76	3.37E-15	7	Col1a1
2.42E-19	0.28366286	1	0.831	5.86E-15	7	Rpl32
5.99E-19	0.29677145	0.454	0.218	1.45E-14	7	Tnn
3.94E-18	0.28456718	0.994	0.624	9.55E-14	7	Sdc3
1.02E-15	0.25752905	0.457	0.243	2.48E-11	7	Xist
5.61E-15	0.40230918	0.413	0.226	1.36E-10	7	Rps3a3
5.59E-93	2.04277226	0.667	0.266	1.36E-88	8	Rbfox2
8.02E-80	1.43787717	0.814	0.386	1.95E-75	8	Hspg2
8.26E-80	1.54828351	0.957	0.763	2.00E-75	8	Col1a1
1.23E-73	1.29989674	0.954	0.767	2.99E-69	8	Col1a2
2.63E-62	1.33467585	0.968	0.932	6.37E-58	8	Spp1
8.72E-61	1.28027414	0.887	0.568	2.11E-56	8	Timp3
4.65E-58	1.26791647	0.71	0.346	1.13E-53	8	Cavin1
7.82E-57	1.24400073	0.739	0.359	1.90E-52	8	Col5a1
3.27E-54	1.13608316	0.89	0.583	7.92E-50	8	Ctgf
3.54E-54	0.85135602	0.948	0.534	8.59E-50	8	Sparc
4.11E-54	1.19987448	0.817	0.453	9.97E-50	8	Timp1
7.53E-54	1.03338304	0.881	0.554	1.83E-49	8	Cyr61

5.88E-49	0.84278069	0.954	0.615	1.42E-44	8	Fn1
2.74E-47	1.00369141	0.629	0.277	6.65E-43	8	Loxl2
5.11E-47	1.13546846	0.548	0.245	1.24E-42	8	Mrc2
3.27E-44	1.12512261	0.667	0.363	7.93E-40	8	Qsox1
1.72E-43	1.0713932	0.8	0.499	4.17E-39	8	Ccnd1
4.65E-42	0.92761692	0.896	0.649	1.13E-37	8	Serpine1
2.56E-41	0.99241757	0.565	0.275	6.22E-37	8	S100a16
5.15E-40	1.05364095	0.545	0.253	1.25E-35	8	Cdh11
2.39E-39	0.73727172	0.852	0.455	5.79E-35	8	Bgn
2.02E-38	0.68294306	0.754	0.384	4.91E-34	8	Ccnd2
6.72E-38	1.03089072	0.748	0.476	1.63E-33	8	Ckap4
1.48E-36	1.2172995	0.49	0.247	3.59E-32	8	Chpf
4.93E-34	0.85259207	0.67	0.393	1.19E-29	8	Rhoc
6.17E-34	0.73715553	0.777	0.425	1.50E-29	8	Serpinh1
1.88E-33	0.68193824	0.771	0.431	4.55E-29	8	Lox
2.17E-32	1.48274308	0.667	0.422	5.25E-28	8	Edn1
4.97E-32	0.6724309	0.91	0.856	1.21E-27	8	Chchd2
7.29E-32	0.96481656	0.797	0.571	1.77E-27	8	Timp2
8.80E-32	0.87577631	0.461	0.207	2.13E-27	8	Rpl39l
1.79E-31	0.82038502	0.646	0.36	4.34E-27	8	Loxl3
3.32E-31	0.86897593	0.6	0.316	8.05E-27	8	Tnc
3.86E-31	0.63723073	0.716	0.356	9.35E-27	8	Fstl1
2.59E-30	0.93999768	0.571	0.304	6.29E-26	8	Cxcl12

2.50E-29	1.20153122	0.449	0.229	6.06E-25	8	Sp7
6.96E-29	0.49780042	0.954	0.894	1.69E-24	8	Myl6
1.24E-28	0.73978551	0.594	0.303	3.02E-24	8	Ppic
2.38E-28	0.79422039	0.565	0.311	5.77E-24	8	Bmp1
1.38E-27	0.40516678	0.849	0.591	3.35E-23	8	Acta2
1.77E-27	0.70377577	0.794	0.64	4.30E-23	8	Atf4
6.88E-27	0.43477979	0.719	0.42	1.67E-22	8	Tagln
1.03E-26	0.74126792	0.539	0.273	2.50E-22	8	Fkbp10
1.62E-26	0.79244604	0.777	0.577	3.93E-22	8	Pdia6
4.96E-26	0.42642424	0.907	0.692	1.20E-21	8	Thbs1
5.24E-26	0.72224176	0.591	0.31	1.27E-21	8	Prrx1
6.44E-26	0.70478279	0.548	0.318	1.56E-21	8	Flnb
8.61E-26	0.53138816	1	1	2.09E-21	8	Gm42418
1.50E-25	0.9304265	0.545	0.296	3.64E-21	8	Tm4sf1
1.11E-24	0.62488777	0.591	0.321	2.69E-20	8	Inhba
1.15E-24	0.75973963	0.499	0.279	2.78E-20	8	Fkbp9
1.90E-24	0.55733455	0.896	0.718	4.62E-20	8	Anxa2
2.27E-24	0.52410176	0.742	0.426	5.50E-20	8	Csf1
2.72E-24	0.7828339	0.388	0.191	6.60E-20	8	Cdkn2a
4.15E-24	0.53326998	0.849	0.592	1.01E-19	8	Actn1
5.82E-24	0.65942774	0.49	0.252	1.41E-19	8	Plod2
1.58E-23	0.84890814	0.464	0.251	3.82E-19	8	Vasn
1.97E-23	1.2182486	0.368	0.181	4.77E-19	8	Adm

4.11E-23	0.68331384	0.858	0.679	9.96E-19	8	Actn4
4.91E-23	0.47442977	0.58	0.305	1.19E-18	8	Dpysl3
6.51E-23	0.78298608	0.475	0.261	1.58E-18	8	Cpe
8.17E-23	0.32674513	0.864	0.547	1.98E-18	8	Tpm1
2.36E-22	0.61131299	0.49	0.275	5.71E-18	8	Mical2
3.84E-22	0.59106648	0.809	0.596	9.30E-18	8	Bsg
4.17E-22	0.60279978	0.594	0.328	1.01E-17	8	Vcl
4.37E-22	0.73602392	0.467	0.266	1.06E-17	8	Unc5b
5.57E-22	0.70886261	0.528	0.306	1.35E-17	8	2310022B05Rik
8.47E-22	0.59911813	0.78	0.627	2.05E-17	8	Nme1
8.63E-22	0.74789811	0.461	0.255	2.09E-17	8	Rcn1
8.74E-22	0.75620402	0.681	0.502	2.12E-17	8	Rpl24
1.14E-21	0.68784298	0.432	0.239	2.77E-17	8	Dcbld2
1.91E-21	0.67752291	0.843	0.7	4.62E-17	8	Calr
3.82E-21	0.61556595	0.739	0.481	9.26E-17	8	Calu
5.22E-21	0.81831074	0.414	0.229	1.27E-16	8	Twist1
2.79E-20	0.63874951	0.629	0.357	6.76E-16	8	Col5a2
2.90E-20	0.68516527	0.646	0.424	7.03E-16	8	Sdc4
3.96E-20	0.6329941	0.594	0.356	9.61E-16	8	Igfbp7
3.98E-20	0.62444128	0.516	0.301	9.65E-16	8	Maged1
4.67E-20	0.55097018	0.652	0.408	1.13E-15	8	Cnn2
1.65E-19	0.5975406	0.472	0.262	4.00E-15	8	Amotl2
3.59E-19	0.4861453	0.838	0.739	8.70E-15	8	Rsrp1

4.38E-19	0.7119891<br>1	0.458	0.264	1.06E-14	8	Gpc1
5.86E-19	0.4524355<br>3	0.609	0.333	1.42E-14	8	Serpinf1
5.99E-19	0.6777356<br>6	0.423	0.221	1.45E-14	8	Tnn
8.84E-19	0.7214752<br>3	0.472	0.279	2.14E-14	8	Sox4
1.04E-18	0.7754674<br>3	0.565	0.364	2.52E-14	8	Csrp1
1.90E-18	0.5459994<br>4	0.623	0.393	4.61E-14	8	Ecm1
2.05E-18	0.4640506<br>9	0.62	0.385	4.98E-14	8	S100a4
2.60E-18	0.5782814<br>7	0.71	0.523	6.31E-14	8	Itgav
3.25E-18	0.5535137<br>8	0.814	0.626	7.88E-14	8	Flna
3.75E-18	0.6173582<br>8	0.719	0.51	9.08E-14	8	Mmp14
4.70E-18	0.5262617<br>2	0.713	0.553	1.14E-13	8	Prrc2a
5.34E-18	0.4658305<br>1	0.748	0.592	1.29E-13	8	9530068E07Ri<br>k
5.90E-18	0.7082995<br>3	0.446	0.252	1.43E-13	8	Fgf7
1.29E-17	0.5858881<br>1	0.51	0.306	3.14E-13	8	Errfi1
1.39E-17	0.4667168<br>7	0.791	0.663	3.36E-13	8	Snrnp70
1.58E-17	0.5150975<br>4	0.852	0.695	3.82E-13	8	Mif
2.34E-17	0.5178316	0.742	0.595	5.68E-13	8	Cct8
2.71E-17	0.6264212<br>4	0.617	0.478	6.58E-13	8	Glrx5
2.88E-17	0.7248894<br>3	0.452	0.265	6.97E-13	8	Prss23
3.60E-17	0.5491208<br>3	0.49	0.283	8.72E-13	8	Col6a1
4.95E-17	0.4634445<br>7	0.623	0.388	1.20E-12	8	11-Sep
4.97E-17	0.6022370<br>8	0.475	0.276	1.21E-12	8	Serpine2

6.07E-17	0.7032540<br>4	0.386	0.211	1.47E-12	8	Cdh2
9.90E-17	0.2795473<br>8	0.733	0.402	2.40E-12	8	Nedd4
1.22E-16	0.7346099<br>7	0.412	0.243	2.96E-12	8	Nes
1.39E-16	0.5138264<br>7	0.53	0.313	3.38E-12	8	Cdc42ep3
1.86E-16	0.5789617<br>3	0.458	0.291	4.51E-12	8	Tnk2
2.07E-16	0.4796959<br>2	0.872	0.699	5.01E-12	8	Itgb1
3.10E-16	0.6371154<br>1	0.565	0.424	7.51E-12	8	Klc1
3.41E-16	0.5847204<br>8	0.678	0.516	8.27E-12	8	Cct7
3.71E-16	0.7357006	0.707	0.531	8.99E-12	8	Tcf4
4.12E-16	0.4851511<br>4	0.814	0.642	1.00E-11	8	Hspa9
4.14E-16	0.4171366<br>3	0.988	0.97	1.00E-11	8	Rplp1
4.86E-16	0.6335255<br>5	0.626	0.462	1.18E-11	8	Manf
5.38E-16	0.4505043<br>8	0.507	0.296	1.30E-11	8	Fscn1
7.78E-16	0.5781356	0.513	0.308	1.89E-11	8	Col12a1
9.09E-16	0.5106421<br>5	0.719	0.59	2.20E-11	8	Emc10
1.25E-15	0.6361912<br>3	0.432	0.246	3.03E-11	8	Ccdc80
1.67E-15	0.4141105<br>1	0.959	0.956	4.05E-11	8	Rps9
1.74E-15	0.7446757<br>3	0.559	0.416	4.22E-11	8	Rps2-ps10
2.83E-15	0.6697915<br>6	0.62	0.471	6.87E-11	8	Ctnnd1
3.72E-15	0.6252695<br>3	0.493	0.333	9.02E-11	8	Iars
4.35E-15	0.5749661<br>7	0.487	0.318	1.05E-10	8	Ugcg
4.45E-15	0.6911806	0.42	0.271	1.08E-10	8	Pbxip1
1.15E-14	0.5518457<br>6	0.446	0.282	2.78E-10	8	Igf1r

1.81E-14	0.62694138	0.383	0.239	4.39E-10	8	Slc30a4
2.15E-14	0.75967847	0.597	0.443	5.21E-10	8	Lbh
2.21E-14	0.67000988	0.368	0.226	5.36E-10	8	Olfml2b
2.23E-14	0.53996903	0.794	0.638	5.42E-10	8	Sdc3
2.86E-14	0.46415343	0.516	0.332	6.93E-10	8	Pdlim7
4.23E-14	0.71481184	0.351	0.198	1.02E-09	8	Gas1
5.90E-14	0.458801	0.745	0.526	1.43E-09	8	Id3
6.41E-14	0.41925978	0.739	0.602	1.55E-09	8	Degs1
8.23E-14	0.41361101	0.832	0.713	2.00E-09	8	Srsf2
1.58E-13	0.46843624	0.675	0.542	3.84E-09	8	Psmd13
1.86E-13	0.49517421	0.62	0.462	4.50E-09	8	H13
2.00E-13	0.34642852	0.794	0.686	4.85E-09	8	Ddx39b
2.56E-13	0.55344303	0.614	0.456	6.20E-09	8	Tns3
4.13E-13	0.48682318	0.733	0.541	1.00E-08	8	P4hb
6.12E-13	0.3536814	0.583	0.339	1.48E-08	8	Aebp1
6.26E-13	0.63353248	0.359	0.22	1.52E-08	8	Mmp23
7.11E-13	0.34998304	0.586	0.38	1.72E-08	8	Mfge8
8.51E-13	0.6099323	0.643	0.504	2.06E-08	8	Nrp2
9.35E-13	0.48109497	0.426	0.265	2.27E-08	8	Tead1
1.05E-12	0.59577829	0.565	0.411	2.54E-08	8	Sec61a1
1.17E-12	0.43897974	0.788	0.638	2.83E-08	8	Ssr3
1.50E-12	0.5416077	0.577	0.459	3.63E-08	8	Wdr89
1.65E-12	0.65274424	0.426	0.3	4.01E-08	8	Hdac7

1.84E-12	0.47733387	0.655	0.487	4.46E-08	8	Cited2
1.98E-12	1.05081721	0.377	0.248	4.80E-08	8	Crim1
2.49E-12	0.60973058	0.391	0.237	6.04E-08	8	Sdc2
3.11E-12	0.48777421	0.319	0.194	7.55E-08	8	Khdrbs3
3.16E-12	0.29136474	0.945	0.926	7.66E-08	8	Rack1
3.51E-12	0.4773554	0.417	0.249	8.50E-08	8	Cyp1b1
3.51E-12	0.41332564	0.414	0.252	8.52E-08	8	Ryk
3.62E-12	0.68551758	0.365	0.228	8.77E-08	8	Bicc1
3.85E-12	0.43905359	0.6	0.463	9.33E-08	8	Kansl2
4.19E-12	0.48191274	0.441	0.273	1.02E-07	8	Lhfp
4.31E-12	0.58536279	0.339	0.203	1.05E-07	8	Pdk4
4.51E-12	0.46123585	0.696	0.555	1.09E-07	8	Rpn2
4.70E-12	0.50085136	0.765	0.671	1.14E-07	8	Jund
5.66E-12	0.48543428	0.533	0.383	1.37E-07	8	Txndc5
7.37E-12	0.46621557	0.551	0.393	1.79E-07	8	Tulp4
9.72E-12	0.50565348	0.516	0.391	2.36E-07	8	Cand1
1.39E-11	0.5616822	0.533	0.358	3.36E-07	8	Sptbn1
1.42E-11	0.42066177	0.675	0.494	3.43E-07	8	Rock2
1.71E-11	0.42162083	0.484	0.313	4.15E-07	8	Cnn3
1.86E-11	0.33914373	0.713	0.578	4.51E-07	8	Gm26917
2.33E-11	0.40662927	0.696	0.581	5.65E-07	8	Psmd8
2.42E-11	0.35332761	0.678	0.514	5.87E-07	8	Map4k4

3.12E-11	0.48353833	0.345	0.223	7.56E-07	8	Ptk7
3.86E-11	0.34478225	0.843	0.785	9.35E-07	8	Arpc4
4.74E-11	0.45568676	0.455	0.313	1.15E-06	8	Ptprs
7.59E-11	0.3753072	0.733	0.625	1.84E-06	8	Psmd2
1.09E-10	0.42097647	0.562	0.426	2.65E-06	8	Prmt1
1.12E-10	0.47800804	0.464	0.338	2.70E-06	8	Nudcd2
1.18E-10	0.36086983	0.675	0.522	2.86E-06	8	Hmgn1
1.30E-10	0.44761486	0.475	0.319	3.16E-06	8	Dst
1.36E-10	0.94693618	0.412	0.278	3.29E-06	8	Col4a1
1.62E-10	0.69023444	0.287	0.169	3.93E-06	8	Ccl7
2.00E-10	0.70737387	0.443	0.312	4.84E-06	8	Ccl2
2.22E-10	0.39058997	0.754	0.628	5.38E-06	8	Rhob
2.59E-10	0.40694514	0.438	0.281	6.28E-06	8	Efemp2
4.06E-10	0.4926429	0.493	0.362	9.85E-06	8	Piezo1
4.49E-10	0.39589132	0.742	0.638	1.09E-05	8	Vcp
4.88E-10	1.26274171	0.267	0.155	1.18E-05	8	Esm1
4.98E-10	0.5078881	0.455	0.307	1.21E-05	8	Col6a3
5.85E-10	0.36356933	0.429	0.274	1.42E-05	8	Pmepa1
6.01E-10	0.41715069	0.652	0.485	1.46E-05	8	Cdkn1a
6.78E-10	0.36405714	0.855	0.721	1.64E-05	8	Hsp90b1
7.04E-10	0.42292547	0.484	0.367	1.71E-05	8	Strn3
7.31E-10	1.24755584	0.304	0.194	1.77E-05	8	Apln
7.78E-10	0.50726995	0.391	0.255	1.89E-05	8	Thbs2

8.29E-10	0.42853468	0.539	0.403	2.01E-05	8	Crtap
8.32E-10	0.45539851	0.354	0.22	2.02E-05	8	Fbln2
9.61E-10	0.5026017	0.38	0.249	2.33E-05	8	Frmd6
9.61E-10	0.309288	0.875	0.779	2.33E-05	8	Tmbim6
1.03E-09	0.42922023	0.359	0.221	2.49E-05	8	Col8a1
1.12E-09	0.41032312	0.606	0.484	2.72E-05	8	Kdelr1
1.14E-09	0.57816914	0.403	0.293	2.76E-05	8	Ltbp3
1.23E-09	0.64674244	0.31	0.209	2.98E-05	8	Tpbg
1.32E-09	0.44916767	0.354	0.216	3.20E-05	8	Postn
1.47E-09	0.45563133	0.354	0.226	3.55E-05	8	Snai2
1.56E-09	0.42341402	0.626	0.497	3.78E-05	8	Swi5
1.79E-09	0.45876102	0.374	0.241	4.35E-05	8	Dclk1
1.96E-09	0.32288714	0.858	0.762	4.75E-05	8	Myl12a
2.07E-09	0.57309238	0.351	0.243	5.01E-05	8	Rps26-ps1
2.15E-09	0.44529879	0.594	0.473	5.21E-05	8	Wls
3.21E-09	0.52168706	0.455	0.349	7.79E-05	8	Snhg9
3.29E-09	0.3634923	0.377	0.251	7.97E-05	8	Crip2
3.61E-09	0.66345102	0.333	0.232	8.75E-05	8	Rras2
3.78E-09	0.57137538	0.313	0.194	9.16E-05	8	Col11a1
6.13E-09	0.36849351	0.655	0.557	0.00014862	8	Pgls
7.16E-09	0.48765783	0.481	0.377	0.00017371	8	Zbtb38
7.86E-09	0.31085591	0.783	0.677	0.00019068	8	Csnk1a1
8.18E-09	0.44202746	0.522	0.392	0.00019845	8	Nudt4

9.81E-09	0.37130944	0.299	0.186	0.00023791	8	Cemip
1.10E-08	0.96636347	0.345	0.235	0.00026714	8	Emcn
1.15E-08	0.58848273	0.267	0.174	0.00027914	8	Slc7a5
1.30E-08	0.47769839	0.371	0.258	0.0003143	8	Grb10
1.72E-08	0.36159101	0.438	0.288	0.00041757	8	Enah
2.00E-08	0.34250623	0.67	0.536	0.00048549	8	Rpn1
2.74E-08	0.33270053	0.849	0.734	0.0006655	8	Pgk1
2.76E-08	0.41230184	0.496	0.391	0.00066845	8	Sae1
3.02E-08	0.34733097	0.47	0.361	0.00073175	8	Snhg1
3.05E-08	0.37184364	0.577	0.462	0.00073926	8	Celf1
3.39E-08	0.27040562	0.855	0.743	0.00082167	8	Lrp1
3.55E-08	0.35699329	0.397	0.253	0.00085985	8	Fat1
3.60E-08	0.35126042	0.386	0.269	0.00087193	8	Msrb3
3.66E-08	0.4827342	0.348	0.244	0.00088758	8	Tnfrsf12a
3.95E-08	0.45645992	0.377	0.274	0.00095824	8	Rai14
4.04E-08	0.34320175	0.661	0.546	0.00098046	8	Dync1h1
4.78E-08	0.40717216	0.435	0.327	0.00115813	8	Rcn2
5.10E-08	0.5103767	0.536	0.441	0.00123616	8	Rars
6.49E-08	0.52578462	0.348	0.242	0.00157291	8	Rgs3
6.78E-08	0.3119027	0.516	0.347	0.00164493	8	Tpm2
6.92E-08	0.39341726	0.472	0.351	0.00167798	8	Pdia4

7.29E-08	0.33711161	0.296	0.186	0.00176716	8	Serpinb2
8.53E-08	0.27287995	0.829	0.723	0.00206811	8	Srsf5
9.23E-08	0.42530785	0.391	0.291	0.0022381	8	Dmpk
9.28E-08	0.34569837	0.325	0.216	0.00224986	8	Loxl1
9.62E-08	0.46238526	0.319	0.219	0.0023328	8	Efnb1
1.14E-07	0.3868478	0.568	0.477	0.00277482	8	Mcrip1
1.19E-07	0.68124558	0.284	0.202	0.00287972	8	Epha2
1.49E-07	0.39459807	0.417	0.304	0.00361049	8	Zbtb20
1.55E-07	0.43759738	0.267	0.182	0.0037524	8	Trim2
1.66E-07	0.48448532	0.275	0.173	0.00403678	8	Mmp13
1.67E-07	0.32742476	0.629	0.53	0.00403894	8	Dap
1.98E-07	0.30160173	0.751	0.644	0.0047927	8	Fus
2.02E-07	0.34978465	0.562	0.436	0.00490699	8	P4ha1
2.35E-07	0.2823961	0.62	0.476	0.00569005	8	Hdlbp
2.46E-07	0.31892475	0.409	0.283	0.00595764	8	Nfix
2.67E-07	0.28850806	0.783	0.669	0.00646401	8	Tubb5
3.00E-07	0.45172927	0.583	0.461	0.0072799	8	Slc38a2
3.35E-07	0.45271908	0.307	0.21	0.00812646	8	Ptprd
3.78E-07	0.31225958	0.684	0.609	0.00916792	8	Edem1
3.96E-07	0.46079298	0.4	0.316	0.00959679	8	Wdr77
4.05E-07	0.51256542	0.377	0.291	0.00981416	8	Herpud1

4.45E-07	0.28091578	0.791	0.623	0.01079385	8	Myh9
4.91E-07	0.504866	0.446	0.368	0.01190458	8	Tmem43
5.17E-07	0.40720634	0.328	0.227	0.01253121	8	Phgdh
5.23E-07	0.40486426	0.53	0.444	0.01268257	8	Ube2s
5.37E-07	0.30554724	0.516	0.417	0.0130139	8	Zcchc24
5.39E-07	0.36796102	0.461	0.342	0.01305856	8	Pam
5.43E-07	0.3628636	0.464	0.341	0.01315851	8	Kdelr2
5.46E-07	0.37959547	0.548	0.475	0.01324344	8	Clk1
5.88E-07	0.59643315	0.258	0.17	0.01426675	8	Egfl7
6.54E-07	0.39613246	0.42	0.32	0.01585935	8	Mthfd2
6.94E-07	0.42278624	0.481	0.398	0.01683256	8	Phc2
7.23E-07	0.49819839	0.301	0.22	0.01753301	8	Ephx1
9.36E-07	0.35484179	0.38	0.275	0.02270284	8	Scd2
1.02E-06	0.33223108	0.675	0.6	0.02470706	8	Cd164
1.16E-06	0.49300807	0.31	0.232	0.02801149	8	Gstm2
1.18E-06	0.37051316	0.417	0.32	0.02851853	8	Smurf2
1.21E-06	0.39118528	0.368	0.26	0.02931785	8	AI506816
1.30E-06	0.38848064	0.365	0.285	0.03152006	8	Arfip1
1.30E-06	0.48281085	0.386	0.296	0.03158659	8	Rps13-ps1
1.48E-06	0.35600068	0.583	0.476	0.03582357	8	Nisch
1.61E-06	0.27139258	0.429	0.294	0.0390646	8	Myo10

1.68E-06	0.32436237	0.751	0.668	0.04068442	8	Atp1a1
1.80E-06	0.33030429	0.739	0.628	0.04357875	8	Eif2s2
1.87E-06	0.31525761	0.655	0.509	0.04545489	8	Fkbp1a
1.95E-06	0.78768846	0.293	0.212	0.04723443	8	Col4a2
2.00E-06	0.31440633	0.635	0.574	0.04857979	8	Snrpd3
2.02E-06	0.29433408	0.574	0.472	0.04886113	8	Lmo4
2.11E-06	0.31951041	0.386	0.282	0.05112363	8	Fermt2
2.46E-06	0.48263242	0.29	0.206	0.05976413	8	Sorbs2
2.61E-06	0.26300298	0.725	0.658	0.06327703	8	Tapbp
2.62E-06	0.40073984	0.478	0.394	0.06344028	8	Xpot
2.78E-06	0.48973103	0.307	0.233	0.06742458	8	Slc39a14
3.02E-06	0.37585532	0.29	0.197	0.07321198	8	Ncam1
3.05E-06	0.27687562	0.736	0.683	0.0740459	8	Fam120a
3.31E-06	0.35142042	0.414	0.337	0.08024861	8	Gcn1l1
3.37E-06	0.45378992	0.278	0.189	0.08165049	8	Ptgs2
4.16E-06	0.34734535	0.438	0.344	0.10079841	8	Selenow
4.16E-06	0.3556527	0.351	0.256	0.10096772	8	Pcolce
4.37E-06	0.3108849	0.438	0.351	0.10587184	8	Aars
4.47E-06	0.54978787	0.293	0.223	0.10847668	8	Fam208b
5.37E-06	0.25353241	0.136	0.269	0.13030182	8	CT010467.1
5.40E-06	0.38753903	0.423	0.349	0.13102102	8	Smarce1

5.62E-06	0.47888056	0.255	0.172	0.13629034	8	Rgs16
6.39E-06	0.30135195	0.606	0.491	0.1549172	8	Ssr1
6.39E-06	0.45033565	0.432	0.363	0.15493752	8	Setd5
6.95E-06	0.28910305	0.551	0.466	0.16846527	8	Snrpd2
7.76E-06	0.47330496	0.299	0.223	0.1880986	8	Kirrel
8.19E-06	0.30334083	0.38	0.279	0.19856686	8	Ak1
8.88E-06	0.51043412	0.281	0.209	0.21524147	8	P3h4
9.09E-06	0.28569087	0.554	0.482	0.22045922	8	Stub1
9.45E-06	0.29509279	0.501	0.432	0.22923946	8	Nsd1
1.23E-05	0.28767218	0.51	0.406	0.2979711	8	Nfe2l1
1.34E-05	0.35335674	0.487	0.394	0.32413113	8	Ostc
1.43E-05	0.29660226	0.53	0.472	0.3474393	8	Csnk1g2
1.55E-05	0.39556356	0.371	0.305	0.37530474	8	Akt1s1
1.62E-05	0.42474559	0.571	0.502	0.39397157	8	Selenos
1.64E-05	0.40211801	0.368	0.294	0.3969595	8	Il6st
1.67E-05	0.29090151	0.557	0.471	0.40376255	8	Snx18
1.67E-05	0.45647784	0.609	0.517	0.4060618	8	Neat1
1.76E-05	0.28090439	0.499	0.369	0.42722844	8	Cd81
1.80E-05	0.476644	0.299	0.226	0.43709945	8	Klf2
1.88E-05	0.3074386	0.328	0.258	0.4558465	8	Dmwd
1.90E-05	0.36781165	0.354	0.28	0.46003177	8	Ppp2r3d
1.90E-05	0.34524533	0.455	0.389	0.46106088	8	Uggt1

1.96E-05	0.36512109	0.475	0.382	0.47508759	8	Tspan4
2.00E-05	0.30307589	0.388	0.31	0.48529929	8	Fosl2
2.15E-05	0.35574359	0.368	0.299	0.52022893	8	Smg7
2.22E-05	0.36461466	0.394	0.303	0.53731726	8	Tsc22d1
2.47E-05	0.40265563	0.484	0.37	0.5994477	8	Plec
2.52E-05	0.39927858	0.31	0.236	0.61109966	8	P4ha2
2.56E-05	0.33499161	0.33	0.256	0.62042073	8	Shmt2
2.64E-05	0.58300965	0.264	0.188	0.63942577	8	Ctla2a
2.75E-05	0.35768541	0.461	0.366	0.66682009	8	Raph1
2.91E-05	0.32958411	0.522	0.455	0.70571183	8	Cnih1
3.07E-05	0.38687976	0.293	0.212	0.74476881	8	Tgfb2
3.23E-05	0.3108377	0.513	0.43	0.78295445	8	Vmp1
3.66E-05	0.28118908	0.487	0.396	0.88755853	8	Ddx21
4.17E-05	0.42862104	0.339	0.275	1	8	Parl
4.30E-05	0.32958689	0.429	0.348	1	8	Nenf
4.59E-05	0.29298298	0.496	0.436	1	8	Sumo1
4.78E-05	0.31355216	0.501	0.421	1	8	Tnrc18
5.20E-05	0.26469211	0.577	0.517	1	8	Sdf4
5.57E-05	0.38474723	0.362	0.3	1	8	mt-Atp6
5.64E-05	0.2895266	0.446	0.363	1	8	Sar1a
5.95E-05	0.26936216	0.487	0.385	1	8	Ddb1
6.13E-05	0.32814131	0.307	0.24	1	8	Me1

6.32E-05	0.27840092	0.467	0.374	1	8	Pdlim5
6.34E-05	0.39684315	0.336	0.272	1	8	Ifrd1
6.40E-05	0.29976844	0.371	0.272	1	8	Mmp2
6.95E-05	0.40308465	0.272	0.2	1	8	Irx3
7.03E-05	0.32735045	0.351	0.269	1	8	Pdgfa
8.02E-05	0.36030781	0.258	0.197	1	8	Rcl1
9.26E-05	0.29698866	0.455	0.375	1	8	Prkcsh
9.36E-05	0.39053485	0.409	0.36	1	8	Cdc26
9.81E-05	0.59649463	0.881	0.775	1	8	mt-Cytb
0.00010147	0.31852178	0.455	0.395	1	8	Pdha1
0.00010194	0.2871285	0.417	0.351	1	8	Trim35
0.00010356	0.45306045	0.33	0.271	1	8	Slc39a13
0.00010568	0.31848371	0.284	0.212	1	8	Phldb2
0.000114	0.2896905	0.528	0.47	1	8	Gm16286
0.00011879	0.28190506	0.452	0.393	1	8	Acin1
0.00011986	0.27681879	0.51	0.461	1	8	Cdk11b
0.00012322	0.3121819	0.426	0.355	1	8	Bnip3
0.00012721	0.28431996	0.336	0.281	1	8	Cdk16
0.00014491	0.38365729	0.328	0.272	1	8	Sertad1
0.00018208	0.3301272	0.38	0.308	1	8	Peak1
0.00018594	0.27430534	0.481	0.417	1	8	Swap70
0.0001896	0.45228214	0.336	0.252	1	8	Xist

0.00019159	0.26973204	0.423	0.362	1	8	Arglu1
0.00021198	0.27087014	0.432	0.352	1	8	Mlec
0.00024218	0.27451711	0.722	0.702	1	8	Arf5
0.0002425	0.33574446	0.394	0.337	1	8	Naa15
0.00025065	0.30144814	0.472	0.42	1	8	Lman2
0.00025143	0.42060291	0.296	0.244	1	8	Bcl9l
0.00026851	0.25686277	0.484	0.415	1	8	Tomm70a
0.00027624	0.25993846	0.507	0.444	1	8	Psmb3
0.00029165	0.3916915	0.345	0.296	1	8	Ext2
0.00030267	0.3754299	0.4	0.348	1	8	Cyp51
0.00030358	0.40985103	0.278	0.223	1	8	Ddah1
0.00032181	0.28041272	0.438	0.391	1	8	Sdf2
0.00033086	0.45815559	0.4	0.341	1	8	Wwp2
0.0003479	0.37225341	0.351	0.301	1	8	Llgl1
0.00035177	0.34995745	0.342	0.276	1	8	Ext1
0.00035236	0.33147524	0.307	0.244	1	8	Cfh
0.00035305	0.25057734	0.322	0.246	1	8	Ehd2
0.0003668	0.25792357	0.446	0.394	1	8	Nsfl1c
0.00037749	0.39078407	0.394	0.352	1	8	Gnl1
0.00039069	0.27709019	0.345	0.289	1	8	Galk1
0.00039143	0.29463015	0.362	0.292	1	8	Nop58

0.0003964<br>3	0.3916860<br>3	0.264	0.205	1	8	Pdgfrb
0.0004095<br>4	0.4352916<br>9	0.258	0.205	1	8	Klf4
0.0004151<br>1	0.2567898<br>3	0.49	0.407	1	8	Lrrc59
0.0004674<br>1	0.2510584<br>8	0.577	0.5	1	8	Hnrnph1
0.0004852<br>1	0.3022292<br>1	0.278	0.227	1	8	Tob1
0.0004859	0.2981483<br>8	0.31	0.243	1	8	Cdc42bpa
0.0005133<br>9	0.3206974<br>1	0.441	0.371	1	8	Id1
0.0005397<br>7	0.2510447<br>1	0.429	0.382	1	8	Ubr4
0.0006032	0.5314002	0.803	0.678	1	8	mt-Nd4
0.0006145<br>7	0.2739417<br>8	0.394	0.35	1	8	Krit1
0.0006608<br>4	0.2582456<br>9	0.461	0.41	1	8	Gm8624
0.0007555<br>2	0.2836646<br>9	0.452	0.415	1	8	Ppp4c
0.0008093<br>7	0.4223070<br>3	0.261	0.217	1	8	Ptgfrn
0.0008131<br>3	0.2745774<br>3	0.452	0.409	1	8	Emg1
0.0008649<br>6	0.2708320<br>8	0.832	0.808	1	8	Marcks
0.0008726<br>9	0.2543298<br>7	0.304	0.256	1	8	Tarbp2
0.0008781<br>1	0.2955086<br>1	0.562	0.513	1	8	Sgk1
0.0009214<br>8	0.3977589<br>2	0.264	0.208	1	8	Adamts2
0.0009233<br>4	0.3072311<br>9	0.394	0.327	1	8	Esyt2
0.000945	0.3235912<br>5	0.377	0.334	1	8	Agpat5
0.0010826<br>3	0.6206203<br>9	0.823	0.701	1	8	mt-Nd1
0.0011639<br>9	0.2589690<br>5	0.351	0.301	1	8	Rnf145

0.00116883	0.33439972	0.38	0.331	1	8	Arid5b
0.00121379	0.27589584	0.496	0.449	1	8	Mia3
0.00142213	0.27338622	0.261	0.205	1	8	Phlda1
0.00146137	0.30194999	0.354	0.302	1	8	Mxd4
0.001771	0.32930334	0.371	0.334	1	8	Ilkap
0.00195368	0.39285076	0.267	0.227	1	8	Gys1
0.00199864	0.4170971	0.304	0.26	1	8	Ece1
0.00209303	0.28361337	0.403	0.368	1	8	Lrpprc
0.00216875	0.47387891	0.272	0.221	1	8	Fgfr1
0.00222346	0.31817006	0.261	0.213	1	8	Rcn3
0.00237441	0.29106208	0.293	0.252	1	8	Mbtps1
0.00277777	0.26887525	0.328	0.271	1	8	Tmem176a
0.00288628	0.31961514	0.351	0.307	1	8	Cdk6
0.00307842	0.26708978	0.351	0.299	1	8	Dnajc10
0.00317073	0.30172748	0.359	0.32	1	8	U2af1
0.00324649	0.28231552	0.333	0.293	1	8	Arhgap5
0.00336426	0.32255717	0.281	0.233	1	8	Amotl1
0.0034177	0.25909684	0.307	0.264	1	8	Myc
0.00351094	0.41155234	0.287	0.249	1	8	Gna11
0.00365746	0.39287852	0.252	0.211	1	8	Antxr1
0.00447829	0.28404206	0.255	0.217	1	8	Scarf2

0.00458363	0.29229566	0.406	0.378	1	8	Igsf8
0.00507455	0.28180034	0.284	0.241	1	8	Socs5
0.00523205	0.354749	0.278	0.245	1	8	Erlin2
0.00585036	0.28863829	0.316	0.278	1	8	Dpy19l1
0.0058645	0.38429437	0.299	0.255	1	8	Lats2
0.00643055	0.26102805	0.351	0.317	1	8	Txndc12
0.00685731	0.35971904	0.287	0.252	1	8	Nomo1
0.00700795	0.27076172	0.551	0.491	1	8	Hif1a
0.00702631	0.26912207	0.267	0.219	1	8	Rrm2
0.0081811	0.35103366	0.333	0.3	1	8	Dag1
0.00969676	0.28707359	0.441	0.391	1	8	Glg1
5.47E-103	2.32451148	0.632	0.214	1.33E-98	9	Stmn1
1.10E-97	1.16990289	0.977	0.931	2.68E-93	9	Ptma
3.01E-79	2.11550064	0.649	0.286	7.31E-75	9	Hmgb2
9.38E-77	1.69638956	0.443	0.122	2.27E-72	9	Mcm5
4.80E-75	0.94701374	0.997	0.995	1.16E-70	9	Tmsb4x
1.17E-72	2.06699263	0.403	0.104	2.84E-68	9	Pclaf
2.81E-69	1.41651165	0.875	0.699	6.82E-65	9	Tuba1b
1.50E-65	2.06593172	0.435	0.131	3.64E-61	9	Top2a
1.24E-58	1.67705191	0.794	0.587	3.01E-54	9	H2afz
7.74E-56	1.49056961	0.446	0.162	1.88E-51	9	Mcm7

1.06E-55	1.4012589<br>5	0.304	0.073	2.58E-51	9	Asf1b
2.51E-51	1.6181901<br>7	0.467	0.163	6.09E-47	9	Cx3cr1
6.93E-51	1.4967154<br>2	0.478	0.198	1.68E-46	9	Mcm6
7.96E-51	1.4924465<br>8	0.325	0.089	1.93E-46	9	Ccna2
2.15E-47	1.3554911<br>9	0.516	0.224	5.21E-43	9	Gatm
9.40E-45	1.5383523<br>4	0.513	0.243	2.28E-40	9	Cdk1
3.11E-44	1.4976481<br>7	0.762	0.488	7.53E-40	9	Pf4
8.73E-40	1.3774261<br>1	0.414	0.172	2.12E-35	9	Mcm3
2.70E-39	1.3999696<br>4	0.397	0.162	6.54E-35	9	Lig1
8.82E-38	1.3928487<br>7	0.304	0.099	2.14E-33	9	Birc5
1.86E-37	1.0105656<br>3	0.843	0.665	4.51E-33	9	Tubb5
1.84E-35	0.7148366<br>1	0.942	0.875	4.47E-31	9	BC005537
4.88E-34	1.4166419	0.536	0.32	1.18E-29	9	Pcna
4.09E-32	1.3592355<br>3	0.53	0.31	9.93E-28	9	Dck
1.92E-31	1.1982669<br>9	0.301	0.112	4.66E-27	9	Rpa2
7.16E-30	1.2585694<br>1	0.472	0.266	1.74E-25	9	Tmpo
2.69E-29	1.1704776<br>4	0.278	0.101	6.53E-25	9	Racgap1
5.01E-29	1.3236040<br>2	0.58	0.411	1.21E-24	9	Dek
6.76E-29	1.0746721<br>4	0.267	0.095	1.64E-24	9	Uhrf1
7.28E-29	1.3547651<br>6	0.388	0.186	1.76E-24	9	Atad2
3.64E-28	1.3504201<br>3	0.464	0.265	8.83E-24	9	Smc4
5.52E-27	0.8889346<br>4	0.742	0.599	1.34E-22	9	Hnrnpab

3.36E-26	1.20098741	0.284	0.114	8.14E-22	9	Lmnb1
6.93E-25	1.28330011	0.328	0.151	1.68E-20	9	Smc2
2.22E-24	1.19141762	0.258	0.099	5.37E-20	9	Cdca3
5.83E-23	0.91713183	0.643	0.486	1.41E-18	9	Dab2
3.12E-21	1.06403276	0.316	0.154	7.55E-17	9	Mcm2
8.62E-21	1.11597287	0.441	0.278	2.09E-16	9	Rrm1
3.36E-20	0.90167574	0.67	0.569	8.14E-16	9	Nucks1
1.25E-19	0.8252048	0.272	0.121	3.03E-15	9	Fen1
1.84E-19	0.96175915	0.261	0.115	4.47E-15	9	Mad2l1
3.37E-19	0.90320357	0.591	0.464	8.17E-15	9	Ranbp1
3.45E-19	1.11717052	0.394	0.239	8.36E-15	9	Cks1b
2.54E-18	0.69387584	0.725	0.636	6.15E-14	9	Anp32b
3.70E-18	0.49274777	0.881	0.862	8.97E-14	9	Arpc2
1.22E-17	0.6236729	0.783	0.773	2.97E-13	9	Hnrnpf
1.87E-17	1.34900336	0.261	0.122	4.54E-13	9	Ube2c
3.24E-17	0.60810741	0.852	0.799	7.84E-13	9	Lcp1
5.17E-17	0.39272712	0.754	0.654	1.25E-12	9	Lpl
5.42E-16	0.76523905	0.551	0.393	1.31E-11	9	Msr1
7.84E-16	1.06490766	0.267	0.135	1.90E-11	9	Hells
1.39E-15	1.22821774	0.351	0.213	3.37E-11	9	Rrm2
1.94E-15	1.00008952	0.348	0.21	4.71E-11	9	Cdt1
1.13E-14	1.26052575	0.313	0.185	2.74E-10	9	Ezh2

1.94E-14	0.8717198 6	0.333	0.202	4.69E-10	9	Topbp1
5.76E-14	0.9068588 2	0.565	0.452	1.40E-09	9	Hmgb1
7.22E-14	0.7189621 8	0.487	0.329	1.75E-09	9	Cd52
1.81E-13	0.5862006	0.777	0.749	4.38E-09	9	H3f3a
1.83E-13	0.8165615 1	0.417	0.256	4.43E-09	9	F13a1
3.60E-13	0.7095040 7	0.728	0.727	8.73E-09	9	Ywhah
3.86E-13	0.5521563 9	0.783	0.73	9.37E-09	9	Efhd2
4.78E-13	0.8143954 4	0.536	0.444	1.16E-08	9	Ube2s
6.82E-13	1.0744791 7	0.342	0.223	1.65E-08	9	Cks2
1.67E-12	0.319058	0.928	0.929	4.04E-08	9	Pfn1
7.60E-12	0.6713531 9	0.614	0.575	1.84E-07	9	Naa50
1.26E-11	0.8121407 6	0.49	0.409	3.06E-07	9	Alyref
1.47E-11	0.4862657 1	0.73	0.72	3.56E-07	9	Srsf2
2.86E-11	0.6509742 3	0.565	0.449	6.94E-07	9	Pid1
5.29E-11	0.4809005 9	0.649	0.522	1.28E-06	9	Igfbp4
1.69E-10	0.6911934 9	0.504	0.387	4.10E-06	9	Coro1a
1.74E-10	0.4837832 6	0.875	0.826	4.21E-06	9	Ybx1
2.00E-10	0.6389283 4	0.452	0.32	4.85E-06	9	Spi1
2.55E-10	0.6249633 3	0.623	0.55	6.19E-06	9	Hnrnpa3
3.09E-10	0.6450338 9	0.365	0.23	7.48E-06	9	AI662270
3.75E-10	0.9045835 5	0.258	0.158	9.08E-06	9	Tipin
4.94E-10	0.6015364 2	0.974	0.927	1.20E-05	9	Rps29

1.16E-09	0.53898485	0.629	0.55	2.81E-05	9	Arhgdib
1.17E-09	0.49588416	0.71	0.677	2.85E-05	9	Nap1l1
1.98E-09	0.33371118	0.658	0.508	4.80E-05	9	Ccnd1
2.79E-09	0.3245024	0.925	0.898	6.76E-05	9	Cfl1
2.86E-09	0.25263038	0.991	0.976	6.94E-05	9	Ftl1
3.24E-09	0.72026143	0.51	0.425	7.84E-05	9	Cbx3
3.54E-09	0.83491362	0.354	0.261	8.59E-05	9	Dnmt1
4.41E-09	0.52311993	0.319	0.198	0.00010685	9	Slc7a8
4.76E-09	0.42994491	0.757	0.741	0.00011544	9	Gnai2
5.54E-09	0.58810528	0.588	0.523	0.00013424	9	Ptges3
5.83E-09	0.57354771	0.672	0.668	0.00014138	9	Tpm3
6.32E-09	0.32327656	0.817	0.82	0.00015323	9	Hnrnpa2b1
7.75E-09	0.95497675	0.365	0.285	0.0001878	9	Slbp
7.79E-09	0.33814649	0.896	0.856	0.00018877	9	Ppia
9.12E-09	0.34515777	0.82	0.819	0.00022117	9	H3f3b
9.23E-09	0.40061708	0.768	0.687	0.00022376	9	Tyrobp
1.29E-08	0.8445611	0.417	0.35	0.00031307	9	Smc6
1.35E-08	0.55722614	0.357	0.238	0.00032763	9	Cd72
1.45E-08	0.55272865	0.643	0.572	0.00035186	9	Ran
1.84E-08	0.79896169	0.383	0.3	0.00044591	9	Cbx5
1.89E-08	0.35094312	0.797	0.782	0.0004584	9	Rbm3
2.12E-08	0.43851087	0.762	0.754	0.00051445	9	Arpc1b

2.30E-08	0.7432910<br>9	0.281	0.188	0.0005588<br>9	9	E2f1
3.49E-08	0.7873521<br>5	0.371	0.283	0.0008451<br>3	9	Fam111a
3.49E-08	0.8898812<br>7	0.258	0.171	0.0008456<br>2	9	Usp1
4.25E-08	0.4640422<br>8	0.742	0.718	0.0010293<br>2	9	Mir6236
7.52E-08	0.4376720<br>5	0.667	0.629	0.0018241<br>3	9	Tagln2
7.81E-08	0.5446116<br>6	0.62	0.617	0.0018927	9	Fam49b
1.12E-07	0.5016536<br>2	0.699	0.726	0.0027136<br>6	9	Ckb
1.13E-07	0.4588055<br>9	0.687	0.635	0.0027285<br>7	9	Cotl1
2.34E-07	0.6112197<br>1	0.472	0.387	0.0056768<br>6	9	C5ar1
2.37E-07	0.6906193<br>6	0.342	0.245	0.0057528<br>1	9	Ccl9
2.58E-07	0.3303698<br>1	0.777	0.795	0.0062445<br>7	9	Pcbp2
2.96E-07	0.4058258<br>3	0.728	0.746	0.0071803<br>8	9	Capza2
4.28E-07	0.3940137<br>5	0.736	0.732	0.0103810<br>4	9	Maf
6.16E-07	0.4710950<br>8	0.649	0.648	0.0149405<br>3	9	Sh3bgrl3
7.94E-07	0.5617549<br>3	0.574	0.556	0.0192525<br>1	9	Eid1
1.02E-06	0.6790932<br>4	0.571	0.572	0.0246973<br>7	9	Snx2
1.27E-06	0.4372413	0.629	0.597	0.0307758<br>3	9	Ctsc
1.38E-06	0.5370219<br>1	0.623	0.642	0.0335425<br>7	9	H2afy
1.66E-06	0.4136230<br>9	0.672	0.601	0.0402153<br>5	9	C1qb
1.87E-06	0.5289860<br>1	0.299	0.203	0.0453050<br>9	9	Pycard
1.88E-06	0.5736862<br>5	0.455	0.376	0.0454722<br>9	9	Rab32

1.97E-06	0.43056307	0.661	0.65	0.04768789	9	Fus
2.85E-06	0.4406803	0.643	0.663	0.06917849	9	Snrpb
3.07E-06	0.61130615	0.53	0.496	0.07448498	9	Srsf3
3.18E-06	0.82095005	0.278	0.211	0.0770264	9	Nsd2
3.43E-06	0.34779999	0.757	0.801	0.08316307	9	Actr2
4.49E-06	0.32399852	0.339	0.235	0.10891757	9	Lpxn
4.72E-06	0.83039881	0.362	0.311	0.11450883	9	Rangap1
5.79E-06	0.64445321	0.464	0.427	0.14051023	9	Ssrp1
6.66E-06	0.25301506	0.803	0.79	0.16151874	9	Calm1
6.93E-06	0.56393709	0.484	0.429	0.16801724	9	Celf2
7.88E-06	0.83270826	0.333	0.277	0.19114415	9	Hpf1
8.53E-06	0.45003703	0.638	0.642	0.20677135	9	Cox5a
8.56E-06	0.84587237	0.29	0.225	0.20749157	9	Nasp
8.64E-06	0.39114245	0.51	0.419	0.20955972	9	Aif1
9.01E-06	0.81693844	0.255	0.192	0.21848896	9	Dut
1.26E-05	0.77167222	0.496	0.497	0.30612549	9	Rad21
1.31E-05	0.39334514	0.667	0.587	0.31858594	9	Igf1
1.33E-05	0.54699488	0.591	0.599	0.32204613	9	Clta
1.39E-05	0.68432684	0.409	0.37	0.33755181	9	Rbbp7
1.41E-05	0.41211547	0.646	0.631	0.3410718	9	Hnrnpu
2.10E-05	0.59477479	0.432	0.395	0.5097442	9	Sae1

2.61E-05	0.52478365	0.455	0.414	0.63392534	9	Srsf1
2.62E-05	0.45081463	0.391	0.29	0.63528832	9	Gmfg
3.56E-05	0.43622613	0.658	0.604	0.86271512	9	Fcer1g
3.70E-05	0.67745962	0.42	0.392	0.89751477	9	Suz12
4.88E-05	0.67955664	0.432	0.404	1	9	Smchd1
5.07E-05	0.64626591	0.441	0.422	1	9	Rbbp4
5.08E-05	0.30234165	0.748	0.802	1	9	Mbnl1
6.18E-05	0.70780296	0.403	0.366	1	9	St3gal5
6.72E-05	0.8016673	0.388	0.362	1	9	Paics
6.81E-05	0.58575923	0.27	0.214	1	9	Rfwd3
8.05E-05	0.58172679	0.455	0.424	1	9	Hnrnpd
8.85E-05	0.27898753	0.786	0.793	1	9	Eif4g2
9.22E-05	0.26141927	0.762	0.704	1	9	C1qc
9.32E-05	0.50395173	0.307	0.233	1	9	Clec12a
0.00011287	0.49771513	0.617	0.587	1	9	Atpif1
0.00011999	0.44216676	0.588	0.583	1	9	Tpd52
0.00013766	0.29451934	0.803	0.722	1	9	Rps16
0.00015144	0.45541314	0.42	0.359	1	9	Arrb2
0.00016892	0.41100243	0.571	0.568	1	9	Sumo2
0.00021134	0.58444712	0.351	0.312	1	9	Dtymk
0.00024681	0.34262987	0.383	0.298	1	9	Clec4a2
0.00027352	0.87873414	0.368	0.349	1	9	H2afv

0.00028672	0.39600096	0.47	0.398	1	9	Fyb
0.00030255	0.44972373	0.583	0.555	1	9	Hint1
0.00030598	0.41725108	0.27	0.198	1	9	Dock2
0.00030731	0.36914698	0.655	0.683	1	9	Ap2s1
0.00032494	0.44352108	0.348	0.279	1	9	Cyth4
0.0003519	0.55487798	0.409	0.384	1	9	B4galt6
0.00038694	0.29507591	0.154	0.267	1	9	Sarnp
0.0004304	0.56920852	0.472	0.474	1	9	Supt16
0.00045716	0.36004171	0.646	0.678	1	9	Ptbp3
0.0005207	0.51620732	0.499	0.485	1	9	G3bp1
0.00053334	0.35024751	0.641	0.667	1	9	Arpc3
0.00055697	0.74490301	0.287	0.248	1	9	Eif1ad
0.00055976	0.36053964	0.588	0.607	1	9	Cmpk1
0.0006462	0.40081387	0.374	0.314	1	9	Itga6
0.00065687	0.58392735	0.386	0.363	1	9	Srsf7
0.00070597	0.60714953	0.446	0.445	1	9	Hprt
0.00085135	0.35276556	0.652	0.669	1	9	Hspa8
0.00097736	0.46322534	0.397	0.349	1	9	Adam8
0.00120087	0.39113514	0.588	0.616	1	9	Sod1
0.00123084	0.36340405	0.684	0.708	1	9	Cox8a
0.00136698	0.54879669	0.374	0.347	1	9	Pnp

0.00160879	0.52078362	0.49	0.511	1	9	1810037I17Rik
0.0016576	0.35028588	0.583	0.541	1	9	Set
0.00180945	0.30440768	0.159	0.26	1	9	Ythdf2
0.00214404	0.31886108	0.487	0.433	1	9	Csf1r
0.00249013	0.27403599	0.209	0.326	1	9	Appbp2
0.00270345	0.4138192	0.272	0.223	1	9	Sgk3
0.0034039	0.40473077	0.571	0.601	1	9	Tkt
0.00351314	0.61903206	0.388	0.394	1	9	Anp32e
0.00401165	0.63334599	0.325	0.303	1	9	Glrx
0.00417506	0.29792877	0.183	0.285	1	9	Ndufs7
0.00423711	0.25313923	0.162	0.261	1	9	Sec11a
0.0043841	0.29473394	0.617	0.638	1	9	Zfp36l2
0.00450083	0.37741648	0.522	0.527	1	9	Cnbp
0.00479771	0.65280855	0.391	0.383	1	9	Marcksl1
0.0048296	0.50980374	0.51	0.533	1	9	Ly6e
0.00498611	0.33759712	0.383	0.325	1	9	Ptpn18
0.0057758	0.33486672	0.322	0.27	1	9	Dock10
0.00587358	0.35358204	0.313	0.259	1	9	Slfn2
0.00590943	0.34343134	0.557	0.529	1	9	Hmgn1
0.0065545	0.26916943	0.707	0.702	1	9	Serbp1
0.00662203	0.52080941	0.29	0.265	1	9	Parp1

0.00696887	0.35744112	0.49	0.469	1	9	Stab1
0.00731069	0.27641899	0.38	0.327	1	9	Cfp
0.00738061	0.55823838	0.504	0.536	1	9	Tuba1c
0.00765298	0.40865333	0.426	0.383	1	9	Tm6sf1
0.00801674	0.37966829	0.49	0.505	1	9	Anp32a
0.00808478	0.62133978	0.386	0.389	1	9	Cbfb
0.0080962	0.32908335	0.484	0.468	1	9	Hnrnpa1
0.00814442	0.46072533	0.464	0.486	1	9	Tra2b
0.00872332	0.52085318	0.464	0.492	1	9	Azin1
0.00914006	0.2796015	0.203	0.306	1	9	Vbp1
0.00918419	0.36769931	0.577	0.594	1	9	Slc25a5
0.00974378	0.3138884	0.174	0.266	1	9	Mmgt1
0.00999197	0.44324148	0.336	0.309	1	9	Cln6
1.72E-251	3.38277847	0.761	0.08	4.17E-247	10	H2-Aa
3.41E-214	2.64206577	0.582	0.049	8.27E-210	10	H2-Eb1
2.49E-174	3.56305201	0.803	0.145	6.04E-170	10	Cd74
1.51E-156	2.82183108	0.657	0.099	3.66E-152	10	H2-Ab1
1.37E-24	0.65979455	0.93	0.94	3.33E-20	10	B2m
2.06E-23	0.76543642	0.981	0.966	4.99E-19	10	Psap
2.99E-22	0.92111629	0.859	0.827	7.25E-18	10	Sat1
2.39E-21	0.85595299	0.873	0.786	5.79E-17	10	Laptm5

1.77E-19	0.84360239	0.822	0.703	4.28E-15	10	C1qc
1.15E-18	1.16814213	0.728	0.635	2.78E-14	10	Slc40a1
1.72E-18	1.2042369	0.282	0.104	4.17E-14	10	Tspan13
6.89E-18	0.71290075	0.901	0.868	1.67E-13	10	Lgmn
8.16E-17	0.91516548	0.746	0.61	1.98E-12	10	C1qa
5.40E-16	1.40425263	0.371	0.174	1.31E-11	10	Cx3cr1
8.45E-16	0.4130919	1	0.995	2.05E-11	10	Tmsb4x
5.56E-15	0.83928641	0.676	0.477	1.35E-10	10	Ly86
1.18E-14	0.77967094	0.742	0.6	2.85E-10	10	C1qb
1.65E-14	0.95023392	0.596	0.418	4.00E-10	10	Aif1
2.36E-14	0.90823084	0.723	0.682	5.72E-10	10	Tnfaip2
5.25E-14	1.03358596	0.329	0.162	1.27E-09	10	H2-DMb1
7.83E-14	0.65208885	0.84	0.799	1.90E-09	10	Sirpa
9.75E-14	0.47150775	0.977	0.966	2.37E-09	10	Apoe
2.00E-13	0.68897014	0.836	0.838	4.84E-09	10	H2-K1
5.30E-13	0.44560989	0.92	0.846	1.29E-08	10	Ctss
2.63E-12	1.30993037	0.329	0.173	6.38E-08	10	Mmp13
3.71E-12	0.65002121	0.836	0.792	8.98E-08	10	Mpeg1
5.42E-12	0.81786471	0.681	0.574	1.31E-07	10	Cybb
1.47E-11	0.85434602	0.638	0.53	3.56E-07	10	Cd300a
6.50E-11	1.0026174	0.272	0.136	1.58E-06	10	Ptgs1
7.45E-11	0.8801275	0.601	0.47	1.81E-06	10	Pla2g7
1.08E-10	0.82378561	0.671	0.596	2.61E-06	10	Ctsc

1.36E-10	0.73730727	0.413	0.253	3.30E-06	10	Pld4
2.80E-10	0.7997682	0.535	0.39	6.79E-06	10	Lst1
1.76E-09	0.93870098	0.474	0.334	4.26E-05	10	Clec4n
2.66E-09	0.36617667	0.986	0.978	6.46E-05	10	Ctsb
3.38E-09	1.07579324	0.531	0.441	8.20E-05	10	Gm2a
4.90E-09	0.540727	0.808	0.801	0.00011892	10	Taldo1
8.12E-09	0.46316608	0.859	0.8	0.00019677	10	Lcp1
8.24E-09	0.88245265	0.437	0.309	0.00019991	10	Fam105a
9.52E-09	0.72591852	0.531	0.397	0.00023087	10	Msr1
1.74E-08	0.4687636	0.812	0.794	0.00042152	10	Npc2
2.16E-08	0.72096025	0.469	0.334	0.00052429	10	Cd52
2.55E-08	0.8580547	0.535	0.455	0.00061943	10	Kctd12
3.59E-08	0.70061093	0.254	0.134	0.00087041	10	Sorl1
3.64E-08	0.83200112	0.432	0.306	0.0008816	10	Fcgr4
6.28E-08	0.90419344	0.563	0.509	0.00152167	10	Nampt
6.85E-08	0.70594253	0.545	0.432	0.00166088	10	Csf1r
7.50E-08	0.65326233	0.653	0.58	0.00181754	10	Tpd52
1.41E-07	0.52952297	0.69	0.604	0.00342885	10	Fcer1g
1.90E-07	0.54379567	0.723	0.746	0.00461277	10	Capza2
2.77E-07	0.53589589	0.648	0.567	0.00670838	10	Slc43a2
3.05E-07	0.69765129	0.629	0.566	0.00739608	10	Mafb
3.25E-07	0.80823057	0.498	0.41	0.00787224	10	Rgs2

5.45E-07	0.44843414	0.812	0.844	0.013224	10	Grn
6.37E-07	0.95453099	0.474	0.391	0.01545326	10	Tgm2
8.08E-07	1.09156457	0.465	0.402	0.0195825	10	Ier3
8.88E-07	0.2902412	0.897	0.93	0.02153601	10	Pfn1
1.10E-06	0.86197079	0.427	0.325	0.02662931	10	Itgal
1.35E-06	0.39501013	0.817	0.809	0.03274966	10	Marcks
1.45E-06	0.67160927	0.643	0.625	0.03518244	10	Rcbtb2
2.11E-06	0.70044153	0.502	0.406	0.05107279	10	Clec4a1
2.12E-06	0.41285678	0.808	0.844	0.05137898	10	Sdcbp
2.59E-06	0.66839606	0.601	0.506	0.06271792	10	Ms4a7
4.48E-06	0.32166978	0.845	0.867	0.10871572	10	Itm2b
5.14E-06	0.45223762	0.737	0.732	0.12468973	10	Maf
5.65E-06	0.32452407	0.873	0.879	0.13700467	10	BC005537
9.14E-06	0.69467112	0.258	0.159	0.2215999	10	Atp8a1
1.28E-05	0.45706793	0.695	0.638	0.30981095	10	Selenop
1.39E-05	0.43931424	0.732	0.784	0.3365436	10	Dazap2
1.43E-05	0.66975744	0.315	0.215	0.34586612	10	Lair1
2.75E-05	0.4044553	0.746	0.732	0.66713615	10	Efhd2
2.96E-05	0.75453149	0.423	0.332	0.7167007	10	Ccr5
3.62E-05	0.58082771	0.601	0.566	0.87855949	10	Cd93
4.05E-05	0.63807866	0.474	0.399	0.98263207	10	Fyb

4.89E-05	0.73603955	0.545	0.489	1	10	Clec7a
5.98E-05	0.37598609	0.751	0.774	1	10	Zeb2
6.55E-05	0.32383222	0.793	0.767	1	10	Creg1
6.77E-05	0.5842802	0.408	0.315	1	10	Ms4a6c
7.32E-05	0.26137579	0.155	0.32	1	10	Rab8a
8.48E-05	0.59241592	0.46	0.381	1	10	Wfdc17
9.27E-05	0.37862449	0.737	0.791	1	10	Arpc4
9.72E-05	0.25729741	0.127	0.274	1	10	Vta1
0.00010131	0.56110063	0.418	0.335	1	10	Hcls1
0.00010187	0.88585881	0.352	0.29	1	10	Emb
0.0001068	0.33448959	0.751	0.789	1	10	H2-D1
0.00013236	0.7089057	0.507	0.485	1	10	Zfp36
0.00014518	0.50715757	0.535	0.474	1	10	Fermt3
0.00014829	0.66180204	0.441	0.371	1	10	Inpp5d
0.00016143	0.79464938	0.432	0.384	1	10	Lyn
0.0001669	0.32200761	0.761	0.802	1	10	Ptp4a2
0.00018593	0.2568438	0.155	0.312	1	10	Ubr3
0.0001865	0.62688529	0.286	0.205	1	10	Frmd4b
0.00018665	0.63851824	0.291	0.208	1	10	Srgn
0.00020045	0.45294266	0.634	0.665	1	10	Vamp8
0.00021567	0.54847331	0.315	0.232	1	10	Lcp2
0.00021591	0.57539893	0.408	0.325	1	10	Spi1

0.00022409	0.53418017	0.596	0.605	1	10	Cd164
0.00025053	0.39796655	0.136	0.282	1	10	Usp15
0.00028605	0.45245961	0.662	0.683	1	10	Cyba
0.0003032	0.5786727	0.268	0.18	1	10	Pou2f2
0.00030967	0.34473351	0.723	0.802	1	10	Mbnl1
0.00031905	0.63344507	0.451	0.392	1	10	Coro1a
0.0003358	0.4733224	0.714	0.741	1	10	Adgre1
0.00038465	0.30968126	0.15	0.295	1	10	Cse1l
0.00039836	0.66628271	0.315	0.242	1	10	Cd72
0.00044751	0.42008161	0.653	0.637	1	10	Cotl1
0.00045007	0.41931902	0.704	0.725	1	10	Ckb
0.00047726	0.54294273	0.333	0.256	1	10	Entpd1
0.0005707	0.58535572	0.479	0.46	1	10	Ctsh
0.00060267	0.3729708	0.69	0.737	1	10	Gdi2
0.0006353	0.48824149	0.549	0.514	1	10	Itgb2
0.00068132	0.68305181	0.357	0.294	1	10	Irf8
0.00069504	0.58515694	0.371	0.311	1	10	Ptk2b
0.00070125	0.51704031	0.371	0.301	1	10	Clec4a2
0.00082501	0.53759626	0.624	0.664	1	10	Tapbp
0.00089616	0.38116105	0.127	0.252	1	10	Chrac1
0.00107854	0.38923902	0.685	0.73	1	10	Serp1
0.00131318	0.43465606	0.601	0.611	1	10	Ucp2

0.00135834	0.25601563	0.23	0.399	1	10	Kdm2a
0.00142125	0.27657533	0.197	0.347	1	10	Cyb5r1
0.00145599	0.27758718	0.779	0.825	1	10	Arpc5
0.00169951	0.29385559	0.718	0.776	1	10	Hnrnpf
0.00185585	0.59792716	0.272	0.207	1	10	Pycard
0.00202071	0.30152199	0.169	0.305	1	10	Ptpn12
0.00205862	0.49015357	0.62	0.667	1	10	Ctsa
0.00223	0.63021723	0.385	0.328	1	10	Cfp
0.00242478	0.53463414	0.559	0.554	1	10	Arhgdib
0.00246583	0.27231569	0.141	0.262	1	10	Spg7
0.00247204	0.37129252	0.653	0.704	1	10	Tmem50a
0.00249626	0.51828064	0.596	0.646	1	10	Grk2
0.00262589	0.25840327	0.15	0.273	1	10	Creb1
0.00265139	0.40853671	0.638	0.688	1	10	Adipor1
0.00273976	0.68665075	0.516	0.532	1	10	Ly6e
0.00287613	0.58178607	0.465	0.448	1	10	Cxcl16
0.00304444	0.26375284	0.15	0.268	1	10	Fbxo33
0.00307533	0.31769079	0.188	0.331	1	10	Csk
0.00321805	0.26520757	0.192	0.331	1	10	Acbd5
0.00341037	0.33566631	0.15	0.271	1	10	Stag1
0.00369642	0.47147783	0.573	0.602	1	10	Unc93b1

0.00386343	0.26228359	0.249	0.412	1	10	Smchd1
0.00391599	0.30931453	0.141	0.258	1	10	Ythdf2
0.00395426	0.25252111	0.146	0.262	1	10	Vcpip1
0.00408371	0.3059281	0.16	0.283	1	10	Nr3c1
0.00418479	0.26392912	0.207	0.354	1	10	Cdc42se1
0.00430702	0.53181914	0.376	0.328	1	10	Ccr1
0.00431483	0.32381356	0.732	0.787	1	10	Tmbim6
0.00450341	0.2601002	0.146	0.261	1	10	Mfsd14a
0.00477317	0.63046622	0.282	0.225	1	10	Rgs1
0.0048106	0.28711037	0.183	0.315	1	10	Psmc5
0.00503571	0.39076296	0.634	0.696	1	10	Mcl1
0.00505906	0.3153609	0.216	0.357	1	10	Usp8
0.0051241	0.35718219	0.192	0.328	1	10	Map1s
0.005306	0.25402507	0.169	0.289	1	10	Filip1l
0.00548165	0.58922208	0.413	0.377	1	10	Fcgr3
0.00573153	0.37900202	0.681	0.693	1	10	Tyrobp
0.00581733	0.49048126	0.3	0.246	1	10	P2ry6
0.00583606	0.44583477	0.315	0.255	1	10	Pirb
0.00621498	0.46087681	0.469	0.45	1	10	Fli1
0.00661238	0.43200504	0.178	0.305	1	10	Stat6
0.00671473	0.40224911	0.296	0.236	1	10	AI662270

0.00671972	0.53018973	0.512	0.537	1	10	Prkcd
0.00682882	0.25420769	0.164	0.282	1	10	Ranbp9
0.00687497	0.55022635	0.333	0.282	1	10	Ncf1
0.00700782	0.45616313	0.573	0.627	1	10	Picalm
0.00700871	0.67624791	0.488	0.503	1	10	Dusp6
0.00801781	0.39027393	0.427	0.384	1	10	Tm6sf1
0.0080684	0.27574339	0.718	0.801	1	10	Actr2
0.0082196	0.34962039	0.202	0.332	1	10	Cnot6l
0.00822932	0.35808261	0.23	0.376	1	10	Etfa
0.00827233	0.29367189	0.263	0.421	1	10	Gmfb
0.00857524	0.2630847	0.155	0.265	1	10	Limd1
0.00884706	0.3306835	0.16	0.272	1	10	Tomm34
0.00904281	0.32289315	0.197	0.328	1	10	Atg3
0.00909526	0.53276255	0.563	0.588	1	10	Il10rb
0.00916452	0.49021755	0.366	0.32	1	10	Ncf2
0.00964156	0.27214655	0.174	0.292	1	10	Cnih4
0.00965096	0.25381412	0.357	0.543	1	10	Zfp91
0.00966265	0.32151639	0.183	0.304	1	10	Rbbp6
0	1.11625946	0.889	0.014	0	11	Sox5
0	0.27824395	0.361	0.001	0	11	Grpr
5.42E-185	1.09085731	0.333	0.003	1.31E-180	11	Mmp3

6.11E-185	1.15488555	0.778	0.021	1.48E-180	11	Foxf1
4.84E-182	0.40321783	0.389	0.004	1.17E-177	11	Foxq1
7.34E-159	0.59821559	0.389	0.005	1.78E-154	11	Bmp7
1.92E-149	0.25273244	0.306	0.003	4.65E-145	11	Penk
8.48E-145	1.14178632	0.639	0.018	2.06E-140	11	Cbln1
3.23E-141	1.7412237	0.778	0.029	7.84E-137	11	Nrn1
3.29E-139	2.13506356	0.861	0.037	7.98E-135	11	Cd24a
3.22E-126	0.44794924	0.417	0.008	7.80E-122	11	Spon2
9.25E-120	0.33073291	0.444	0.01	2.24E-115	11	Ephx2
4.69E-118	0.34884416	0.472	0.011	1.14E-113	11	Clu
2.56E-100	0.90578399	0.667	0.03	6.21E-96	11	Angpt1
8.13E-98	0.25456828	0.278	0.004	1.97E-93	11	Rnf152
5.08E-96	1.07911385	0.611	0.026	1.23E-91	11	Anxa8
2.03E-90	0.54778003	0.722	0.04	4.92E-86	11	3632451O06Rik
3.98E-90	0.37305852	0.528	0.02	9.66E-86	11	Stk26
2.44E-82	1.37777776	0.917	0.077	5.91E-78	11	Nt5e
1.16E-81	0.83909315	0.528	0.023	2.82E-77	11	Gm2694
2.91E-79	0.67816382	0.5	0.021	7.07E-75	11	Rspo3
6.76E-79	0.5906999	0.361	0.01	1.64E-74	11	Ism1
3.87E-76	0.41711493	0.583	0.031	9.39E-72	11	Eps8l2
6.92E-71	0.37697572	0.472	0.021	1.68E-66	11	Mgst2
1.13E-68	1.97373337	0.833	0.076	2.74E-64	11	Sema3c

3.67E-67	0.99429628	0.5	0.025	8.90E-63	11	Efemp1
1.23E-66	0.61969062	0.722	0.056	2.98E-62	11	Sobp
1.25E-66	0.69193231	0.472	0.023	3.03E-62	11	Fgf10
2.91E-65	0.46354091	0.611	0.04	7.06E-61	11	Csgalnact1
7.38E-65	0.80473922	0.722	0.058	1.79E-60	11	Kank1
7.39E-65	1.11594224	0.583	0.036	1.79E-60	11	Prelp
2.44E-64	2.08774779	0.333	0.011	5.91E-60	11	Cxcl5
7.24E-63	1.70401124	0.972	0.12	1.75E-58	11	Ptges
2.15E-62	0.47759443	0.5	0.028	5.20E-58	11	Rab3b
3.89E-61	4.39093392	0.722	0.063	9.43E-57	11	Igfbp5
2.35E-59	2.92826309	0.583	0.04	5.69E-55	11	Col2a1
2.80E-59	0.30108161	0.333	0.012	6.78E-55	11	Aff3
2.88E-59	1.2768373	0.528	0.032	6.98E-55	11	Ereg
4.80E-59	0.95552894	0.778	0.073	1.16E-54	11	Rasl11a
1.39E-58	0.59100742	0.5	0.029	3.36E-54	11	Panx3
1.00E-57	1.45210646	0.528	0.033	2.43E-53	11	Sned1
1.82E-56	1.29312848	0.833	0.092	4.41E-52	11	Rgs17
2.09E-54	0.5982865	0.778	0.08	5.07E-50	11	Hspb6
3.94E-54	0.35936267	0.417	0.022	9.56E-50	11	Foxf2
5.84E-53	1.61292656	0.528	0.036	1.42E-48	11	Ndufa4l2
2.51E-52	0.46912509	0.583	0.044	6.10E-48	11	Tiam1
1.44E-51	0.35402753	0.306	0.012	3.50E-47	11	Megf6

1.63E-51	1.62037205	0.806	0.094	3.94E-47	11	Nqo1
2.65E-51	1.89506424	1	0.165	6.42E-47	11	Pcdh7
4.39E-51	0.30309203	0.444	0.026	1.07E-46	11	Gm42639
1.32E-50	1.39077091	0.833	0.094	3.20E-46	11	Fabp7
4.12E-50	1.03346552	0.75	0.08	9.98E-46	11	Sulf1
5.03E-49	1.18656995	0.694	0.071	1.22E-44	11	Ccbe1
9.64E-48	0.3029184	0.361	0.018	2.34E-43	11	Gm42480
4.76E-47	1.70727016	0.917	0.139	1.15E-42	11	Masp1
7.96E-47	0.27856303	0.444	0.028	1.93E-42	11	Gramd2
1.77E-46	1.11120932	0.889	0.13	4.28E-42	11	Pak3
2.26E-46	0.98375781	0.889	0.128	5.48E-42	11	Tgfbr3
7.83E-46	0.93086393	0.583	0.051	1.90E-41	11	Ackr3
1.35E-44	0.41152051	0.361	0.02	3.27E-40	11	S100b
1.50E-44	0.97615703	0.5	0.039	3.64E-40	11	Rbp4
1.74E-43	0.55787597	0.694	0.076	4.22E-39	11	Pde3a
2.25E-43	0.3932871	0.556	0.048	5.46E-39	11	Dnm1
1.13E-42	0.91129548	0.722	0.087	2.73E-38	11	Fgf2
1.35E-42	0.49689772	0.694	0.077	3.28E-38	11	Cdkn2c
3.67E-42	0.25904479	0.472	0.036	8.89E-38	11	Styk1
4.51E-42	0.57910953	0.722	0.088	1.09E-37	11	Elovl6
5.43E-42	0.26166631	0.444	0.031	1.32E-37	11	Gstt3
2.29E-41	1.12184424	0.889	0.143	5.54E-37	11	Prkg2

3.99E-41	0.52432974	0.5	0.042	9.69E-37	11	Igsf10
5.28E-41	0.65131142	0.639	0.07	1.28E-36	11	Plscr2
5.35E-41	0.9015664	0.861	0.125	1.30E-36	11	Fst
5.52E-41	0.32503217	0.528	0.047	1.34E-36	11	Gprc5a
7.18E-41	0.25170186	0.583	0.056	1.74E-36	11	Itga3
1.69E-40	0.39619941	0.639	0.07	4.09E-36	11	Bhlhb9
4.83E-40	0.31513777	0.333	0.019	1.17E-35	11	Epha4
5.44E-40	0.80162789	0.75	0.098	1.32E-35	11	F2r
9.70E-40	1.83635433	0.972	0.182	2.35E-35	11	Ltbp2
3.15E-39	0.64073905	0.667	0.078	7.64E-35	11	Nr4a2
3.19E-39	0.54519252	0.778	0.103	7.73E-35	11	Spata7
4.75E-39	0.97017466	0.833	0.126	1.15E-34	11	Auts2
9.47E-39	0.66731248	0.583	0.062	2.30E-34	11	Col4a5
9.80E-39	0.84120041	0.583	0.062	2.38E-34	11	Gprin3
1.12E-38	0.74257013	0.861	0.138	2.71E-34	11	Shisa4
4.76E-38	0.53744005	0.833	0.126	1.15E-33	11	Gramd4
5.96E-38	1.30587428	0.889	0.137	1.45E-33	11	Ptgs1
1.05E-37	0.25030689	0.417	0.031	2.54E-33	11	Gata2
1.55E-37	0.67686333	0.722	0.09	3.75E-33	11	Smoc1
5.38E-37	0.42352885	0.361	0.024	1.31E-32	11	Kcnma1
6.12E-37	0.86756395	0.778	0.118	1.48E-32	11	Atoh8
6.71E-37	0.26550693	0.417	0.032	1.63E-32	11	Ntn1

8.60E-37	0.35424939	0.389	0.028	2.09E-32	11	Gm43289
1.54E-36	1.11527365	1	0.202	3.72E-32	11	Epha2
3.55E-36	1.50198629	0.917	0.185	8.60E-32	11	Prrx2
4.31E-36	0.59003903	0.667	0.086	1.05E-31	11	Spsb1
4.73E-36	3.07912189	0.944	0.205	1.15E-31	11	Grem1
1.59E-35	0.51364854	0.472	0.043	3.87E-31	11	Bdnf
1.70E-35	1.80027168	0.972	0.191	4.12E-31	11	Hmga2
2.43E-35	0.49727248	0.694	0.094	5.89E-31	11	Pitx1
2.65E-35	0.50382986	0.75	0.114	6.42E-31	11	Tiam2
3.04E-35	0.82723161	0.75	0.116	7.37E-31	11	AC131339.2
6.45E-35	0.55794581	0.583	0.069	1.56E-30	11	Ephb2
6.78E-35	1.99976061	0.944	0.196	1.64E-30	11	Col11a1
8.97E-35	0.38635452	0.583	0.066	2.17E-30	11	Cgnl1
1.01E-34	1.07201559	0.806	0.134	2.45E-30	11	Gm2115
1.30E-34	0.80581123	0.75	0.111	3.15E-30	11	Pex11a
1.39E-34	0.87554574	0.778	0.12	3.36E-30	11	Ahr
1.67E-34	0.73127077	0.889	0.16	4.04E-30	11	Osmr
3.50E-34	0.2697064	0.5	0.049	8.48E-30	11	Cavin4
8.67E-34	1.55166707	0.944	0.187	2.10E-29	11	Plpp3
1.35E-33	1.10453991	0.861	0.163	3.28E-29	11	Synpo
1.39E-33	0.26565488	0.306	0.019	3.37E-29	11	Baiap2l1
2.97E-33	0.93224465	0.917	0.168	7.20E-29	11	Slc12a2

3.38E-33	0.8687712<br>8	0.861	0.162	8.19E-29	11	Steap2
3.68E-33	0.404543	0.417	0.036	8.93E-29	11	Cyp7b1
3.77E-33	1.5798130<br>1	0.944	0.212	9.14E-29	11	Gnb4
4.97E-33	0.9632827<br>8	0.694	0.109	1.21E-28	11	Ror2
1.46E-32	0.9053704<br>1	0.75	0.118	3.53E-28	11	Itga1
1.99E-32	0.3154386<br>5	0.556	0.065	4.83E-28	11	B930095G15Ri<br>k
2.20E-32	0.4021383<br>6	0.639	0.084	5.33E-28	11	Ddit4l
2.40E-32	1.4139716	0.944	0.206	5.82E-28	11	Ltbp1
4.46E-32	0.4122095<br>6	0.722	0.109	1.08E-27	11	Hoxc6
4.57E-32	1.3728085<br>7	0.944	0.205	1.11E-27	11	Gja1
5.13E-32	0.8031186	0.806	0.145	1.24E-27	11	Ptprk
5.70E-32	0.5288049<br>7	0.75	0.115	1.38E-27	11	Cobll1
6.31E-32	1.0897834<br>4	0.722	0.116	1.53E-27	11	Cspg4
7.24E-32	0.3935216<br>7	0.667	0.094	1.76E-27	11	Mbnl3
7.44E-32	0.3055013<br>7	0.528	0.058	1.80E-27	11	Plagl1
7.87E-32	0.3770129<br>6	0.528	0.061	1.91E-27	11	Sdk2
8.03E-32	0.3570216<br>7	0.778	0.122	1.95E-27	11	1700025G04Ri<br>k
1.02E-31	0.5505867	0.694	0.108	2.48E-27	11	Ttll7
1.09E-31	0.9822761<br>5	0.778	0.142	2.64E-27	11	Wfs1
1.29E-31	0.7099130<br>4	0.889	0.167	3.12E-27	11	Foxc1
1.39E-31	1.4352891<br>9	0.667	0.095	3.37E-27	11	Sox9
1.88E-31	1.7561615<br>1	0.917	0.204	4.56E-27	11	Phlda1
1.92E-31	1.9295974<br>7	1	0.262	4.66E-27	11	Plod2

3.24E-31	1.2238724<br>4	0.861	0.176	7.86E-27	11	Zc2hc1a
4.78E-31	1.9474887<br>1	1	0.298	1.16E-26	11	Myo10
6.30E-31	1.9557567<br>9	1	0.295	1.53E-26	11	Ltbp3
6.68E-31	0.7980294<br>4	0.722	0.114	1.62E-26	11	Clmp
7.13E-31	0.5677187	0.806	0.136	1.73E-26	11	Kifc3
9.75E-31	1.1218579<br>7	1	0.24	2.37E-26	11	Prnp
1.04E-30	1.0665292<br>3	0.944	0.202	2.51E-26	11	Lpar1
1.11E-30	0.7094532<br>3	0.861	0.163	2.70E-26	11	Adgra3
1.22E-30	0.4424509<br>3	0.528	0.061	2.96E-26	11	Scx
1.33E-30	0.4692210<br>4	0.667	0.098	3.23E-26	11	Prkar1b
1.50E-30	0.6962784<br>6	0.833	0.148	3.64E-26	11	Fosl1
1.62E-30	0.9523886<br>1	0.806	0.143	3.92E-26	11	Tinagl1
1.66E-30	0.4852541<br>6	0.556	0.07	4.01E-26	11	Gm48996
2.11E-30	1.0393474<br>2	0.917	0.201	5.10E-26	11	Plxdc2
2.27E-30	1.0091320<br>3	0.667	0.097	5.49E-26	11	Cavin2
2.71E-30	4.3438754	0.833	0.175	6.56E-26	11	Mmp13
5.48E-30	0.6573261<br>8	0.611	0.087	1.33E-25	11	Hspb8
5.53E-30	1.5779390<br>7	0.861	0.173	1.34E-25	11	Alpl
5.62E-30	1.1565588<br>2	0.806	0.158	1.36E-25	11	Lgr6
9.01E-30	0.3424584<br>6	0.333	0.026	2.19E-25	11	Gm43423
1.04E-29	0.7544523	0.806	0.141	2.52E-25	11	Steap1
1.47E-29	0.2831269<br>2	0.389	0.035	3.57E-25	11	Cetn4
1.52E-29	1.2959035<br>3	0.972	0.254	3.69E-25	11	Msi2

1.97E-29	1.39145072	0.972	0.211	4.78E-25	11	Col6a2
2.03E-29	0.76179549	0.972	0.208	4.93E-25	11	Col16a1
2.34E-29	1.22969825	0.944	0.226	5.68E-25	11	B3glct
2.56E-29	0.42499272	0.583	0.076	6.20E-25	11	Slc38a4
2.95E-29	1.60954349	0.944	0.234	7.15E-25	11	Pbx1
3.86E-29	0.89913744	0.833	0.152	9.37E-25	11	Zfp503
4.23E-29	0.69180032	0.722	0.122	1.03E-24	11	Itm2a
5.03E-29	0.95962639	0.389	0.035	1.22E-24	11	Mme
5.16E-29	2.04053048	0.972	0.259	1.25E-24	11	Fgf7
6.19E-29	0.63745681	0.722	0.125	1.50E-24	11	Syne1
6.50E-29	0.47427174	0.694	0.111	1.58E-24	11	Srgap3
7.74E-29	0.96205801	0.861	0.181	1.88E-24	11	Hoxc10
1.08E-28	0.63084393	0.75	0.132	2.61E-24	11	Cxxc5
1.21E-28	0.45284813	0.694	0.115	2.93E-24	11	Papss2
1.76E-28	0.54193642	0.639	0.095	4.27E-24	11	Unc5c
1.97E-28	0.78651881	0.583	0.085	4.78E-24	11	Sema3a
2.01E-28	0.64059049	0.611	0.09	4.87E-24	11	Etv1
2.29E-28	0.82219189	0.972	0.212	5.56E-24	11	Dlc1
2.69E-28	0.26313848	0.5	0.06	6.51E-24	11	Mtmr11
4.36E-28	1.06685662	0.861	0.181	1.06E-23	11	Oaf
5.62E-28	0.57649458	0.778	0.143	1.36E-23	11	Hoxa10

5.86E-28	0.6190269<br>8	0.667	0.101	1.42E-23	11	Slco2a1
6.73E-28	0.3813786<br>6	0.5	0.06	1.63E-23	11	C1s1
7.29E-28	2.3861309<br>6	0.861	0.19	1.77E-23	11	Ptgs2
7.36E-28	1.7266172<br>1	1	0.286	1.79E-23	11	Efemp2
7.88E-28	0.9192213	0.972	0.225	1.91E-23	11	Tmem263
8.20E-28	0.4794392<br>6	0.75	0.131	1.99E-23	11	Aig1
8.27E-28	0.3336618<br>6	0.556	0.075	2.01E-23	11	Smad9
8.30E-28	0.8044032<br>5	0.861	0.182	2.01E-23	11	Ets1
1.57E-27	0.7887983<br>8	1	0.225	3.80E-23	11	Tob1
1.58E-27	0.2696783<br>7	0.417	0.043	3.83E-23	11	Prickle2
1.76E-27	0.9219036<br>2	0.694	0.126	4.26E-23	11	Hoxc8
1.90E-27	0.4148687	0.75	0.134	4.61E-23	11	Myo6
1.90E-27	0.5415971<br>7	0.861	0.168	4.61E-23	11	Slc35e3
2.61E-27	0.6128108<br>4	0.722	0.127	6.34E-23	11	Sesn3
2.78E-27	0.2968622<br>8	0.417	0.043	6.74E-23	11	Gm13056
3.15E-27	0.3921162<br>4	0.583	0.081	7.64E-23	11	Tlcd2
5.12E-27	0.5991731<br>6	0.917	0.193	1.24E-22	11	Clstn1
6.26E-27	3.2756991<br>6	0.917	0.221	1.52E-22	11	Mgp
7.37E-27	0.3672758<br>4	0.5	0.062	1.79E-22	11	Gpm6b
7.43E-27	0.7269050<br>6	0.833	0.155	1.80E-22	11	Tnfrsf11b
1.13E-26	0.5912296<br>4	0.75	0.138	2.73E-22	11	Dhcr24
1.14E-26	1.6694809	0.722	0.135	2.77E-22	11	Ptx3
1.74E-26	0.3209409<br>6	0.833	0.159	4.23E-22	11	Mtif2

2.04E-26	1.6556643 8	1	0.324	4.95E-22	11	Dst
2.87E-26	0.871838	0.778	0.157	6.97E-22	11	Tiparp
2.95E-26	1.2227631 2	0.75	0.152	7.15E-22	11	Mllt3
4.11E-26	0.2993822 3	0.667	0.108	9.96E-22	11	B230354K17Ri k
4.30E-26	0.3449261 9	0.694	0.119	1.04E-21	11	Aifm1
5.76E-26	0.3673545 4	0.694	0.12	1.40E-21	11	Rpgrip1l
6.59E-26	0.8163051 2	0.861	0.189	1.60E-21	11	Snhg18
7.32E-26	0.3874608 2	0.667	0.109	1.77E-21	11	Pank1
7.33E-26	0.7550381 6	0.861	0.185	1.78E-21	11	Prkca
7.59E-26	0.6824792 4	0.806	0.165	1.84E-21	11	Neo1
8.65E-26	0.8192834 1	0.833	0.172	2.10E-21	11	Irs1
9.51E-26	0.6578894 6	0.861	0.181	2.31E-21	11	Pdcd4
1.01E-25	0.8967123 5	0.833	0.182	2.46E-21	11	Scd1
1.07E-25	1.4255635 9	1	0.286	2.60E-21	11	Nfix
1.61E-25	0.9049056 2	0.861	0.196	3.90E-21	11	Gpx8
2.09E-25	0.4242139 7	0.833	0.163	5.07E-21	11	Dlg5
2.15E-25	1.4937123 7	0.75	0.157	5.22E-21	11	Thbd
2.19E-25	0.4409963 3	0.25	0.017	5.31E-21	11	Trabd2b
2.20E-25	0.3984848 8	0.667	0.114	5.33E-21	11	1110012L19Ri k
2.20E-25	1.6321091 8	0.694	0.134	5.34E-21	11	Smoc2
2.49E-25	0.2796054 8	0.639	0.104	6.05E-21	11	Dlg3
2.96E-25	0.4923460 1	0.722	0.132	7.17E-21	11	Gria3

3.44E-25	0.79476816	0.778	0.15	8.33E-21	11	Rabgap1l
4.41E-25	0.5887915	0.778	0.155	1.07E-20	11	Cul7
4.57E-25	0.38703158	0.806	0.163	1.11E-20	11	Sipa1l1
4.63E-25	0.84719698	0.889	0.216	1.12E-20	11	Ptgfrn
5.60E-25	0.30278671	0.611	0.096	1.36E-20	11	Ssbp2
6.68E-25	0.80871349	0.806	0.166	1.62E-20	11	Snai1
6.96E-25	0.56742583	0.917	0.202	1.69E-20	11	Cpt1c
8.97E-25	0.46101499	0.528	0.074	2.17E-20	11	Cdon
1.06E-24	0.77572834	0.611	0.104	2.57E-20	11	Ehd3
1.10E-24	0.28841361	0.417	0.048	2.67E-20	11	Car5b
1.24E-24	0.78313118	0.861	0.185	3.01E-20	11	Nid1
1.25E-24	0.29388603	0.444	0.054	3.03E-20	11	5930430L01Rik
1.43E-24	0.7071169	0.944	0.219	3.48E-20	11	Plp2
1.56E-24	0.69125563	0.722	0.136	3.79E-20	11	Irx5
1.67E-24	0.32238837	0.667	0.117	4.06E-20	11	Sgce
1.83E-24	1.1022224	0.944	0.23	4.44E-20	11	Nfib
2.13E-24	0.35457041	0.556	0.085	5.18E-20	11	Gm45338
2.67E-24	0.89758641	0.889	0.21	6.48E-20	11	Pkd2
3.10E-24	0.70307707	0.833	0.191	7.52E-20	11	Lrrk1
3.12E-24	0.61159405	0.778	0.159	7.56E-20	11	Cacna2d1
3.36E-24	0.27404196	0.528	0.076	8.14E-20	11	Cdkl2
5.14E-24	0.69639425	0.75	0.154	1.25E-19	11	Chst11
5.23E-24	0.42705568	0.667	0.118	1.27E-19	11	Fah

5.97E-24	2.23818063	0.694	0.141	1.45E-19	11	Cxcl14
8.39E-24	1.77508661	1	0.255	2.03E-19	11	Cyp1b1
1.08E-23	0.37826906	0.722	0.14	2.63E-19	11	H6pd
1.17E-23	0.54678882	0.778	0.16	2.85E-19	11	Hsd17b7
1.21E-23	1.02381906	0.917	0.238	2.93E-19	11	Trps1
1.33E-23	0.35602841	0.806	0.163	3.21E-19	11	Alg2
1.38E-23	1.89834868	1	0.292	3.35E-19	11	Col6a1
1.52E-23	0.75201598	0.75	0.159	3.68E-19	11	Nsg1
1.69E-23	0.37633442	0.556	0.085	4.11E-19	11	Pcolce2
1.78E-23	1.23642117	0.944	0.268	4.31E-19	11	Colec12
2.08E-23	0.92496247	0.944	0.233	5.05E-19	11	Slc39a14
2.29E-23	1.34451811	0.778	0.171	5.55E-19	11	Cp
2.63E-23	0.40371363	0.833	0.172	6.37E-19	11	Pard3
2.93E-23	0.28561015	0.306	0.028	7.11E-19	11	Nrg1
3.07E-23	0.77772974	0.917	0.223	7.43E-19	11	Kirrel
3.25E-23	1.07728033	0.972	0.242	7.88E-19	11	Sdc2
3.35E-23	0.63929223	0.806	0.17	8.12E-19	11	Emilin1
3.58E-23	1.36444767	0.889	0.217	8.68E-19	11	Rrm2
3.59E-23	0.44636461	0.778	0.154	8.71E-19	11	Sytl2
3.86E-23	0.79978489	0.833	0.184	9.36E-19	11	Klf9
4.73E-23	0.76314442	0.833	0.196	1.15E-18	11	Emp2

5.60E-23	0.5050951<br>3	0.806	0.176	1.36E-18	11	Zfp266
5.95E-23	0.3018133<br>5	0.444	0.058	1.44E-18	11	Morn2
6.09E-23	1.3283815<br>2	1	0.288	1.48E-18	11	Fkbp9
6.34E-23	0.4846192	0.528	0.081	1.54E-18	11	Fzd8
6.58E-23	0.4000560<br>8	0.722	0.142	1.60E-18	11	Ndn
7.47E-23	0.7421095	0.917	0.209	1.81E-18	11	Antxr1
8.30E-23	0.3748364<br>9	0.806	0.168	2.01E-18	11	Hddc2
8.76E-23	0.6846025<br>8	0.556	0.093	2.12E-18	11	Gxylt2
1.23E-22	0.5898043<br>9	0.694	0.13	2.99E-18	11	Slc5a3
1.24E-22	0.6375938<br>7	0.861	0.205	3.01E-18	11	Hipk2
1.38E-22	0.3418412<br>2	0.694	0.136	3.35E-18	11	Npr2
1.43E-22	0.7352507<br>1	0.833	0.184	3.47E-18	11	Ly6a
1.56E-22	1.2169375<br>4	0.972	0.258	3.79E-18	11	Fat1
1.58E-22	0.8404721<br>7	0.889	0.212	3.83E-18	11	Phldb2
1.74E-22	0.8350351<br>5	0.889	0.204	4.21E-18	11	Pdgfrb
1.79E-22	1.3413779<br>1	0.944	0.271	4.34E-18	11	Amotl2
1.94E-22	0.6296328<br>9	0.833	0.189	4.71E-18	11	Mast4
2.03E-22	2.0240017<br>5	1	0.434	4.93E-18	11	Sdc4
2.25E-22	0.4118852<br>2	0.833	0.185	5.45E-18	11	Ptk2
2.39E-22	0.2640578<br>7	0.583	0.097	5.80E-18	11	Fitm2
2.52E-22	1.0899226<br>4	1	0.28	6.10E-18	11	Ak1
2.59E-22	0.8691603<br>4	1	0.265	6.28E-18	11	Smad7

2.64E-22	0.27236477	0.5	0.074	6.39E-18	11	Dzip1
2.68E-22	1.21601142	0.944	0.253	6.51E-18	11	Frmd6
2.75E-22	0.38379279	0.806	0.173	6.66E-18	11	Phldb1
2.83E-22	1.19495777	0.889	0.231	6.86E-18	11	Creb3l1
2.84E-22	0.74136076	0.861	0.221	6.88E-18	11	Uxs1
2.91E-22	0.40470747	0.861	0.194	7.05E-18	11	Tmtc3
2.95E-22	0.32862534	0.778	0.162	7.15E-18	11	Tulp3
3.12E-22	0.48197928	0.75	0.156	7.56E-18	11	Gli3
3.23E-22	0.49824074	0.778	0.17	7.83E-18	11	Tcaf1
4.12E-22	0.27590117	0.778	0.157	9.98E-18	11	Gnai1
5.60E-22	0.38794498	0.611	0.11	1.36E-17	11	Armcx4
6.03E-22	0.37159011	0.722	0.148	1.46E-17	11	Rtl8a
6.10E-22	0.33241573	0.806	0.171	1.48E-17	11	Gstm5
8.43E-22	0.34217708	0.667	0.13	2.04E-17	11	Rhod
9.37E-22	0.39903157	0.722	0.149	2.27E-17	11	Slc10a7
9.82E-22	0.37549718	0.667	0.131	2.38E-17	11	Plcd1
1.05E-21	1.14636624	0.944	0.263	2.55E-17	11	Sdc1
1.13E-21	1.22151606	1	0.307	2.73E-17	11	Zbtb20
1.14E-21	0.28182928	0.639	0.116	2.77E-17	11	Zfp518b
1.15E-21	1.36031216	0.944	0.277	2.79E-17	11	Scd2
1.16E-21	0.30204749	0.417	0.053	2.80E-17	11	D030025P21Rik
1.22E-21	0.9763319	0.944	0.246	2.97E-17	11	Ehd2

1.33E-21	0.7158118<br>3	0.944	0.24	3.21E-17	11	Nfia
1.49E-21	0.9068120<br>9	0.778	0.175	3.61E-17	11	Wisp2
1.50E-21	0.5173612<br>2	0.778	0.173	3.64E-17	11	Fkbp14
1.53E-21	0.3546776<br>7	0.472	0.069	3.70E-17	11	Kazald1
1.64E-21	0.3957262<br>6	0.833	0.192	3.97E-17	11	1110008P14Ri<br>k
1.74E-21	0.7262721<br>1	0.75	0.168	4.22E-17	11	Tacc2
1.87E-21	0.3932211<br>2	0.778	0.167	4.53E-17	11	Ormdl3
1.89E-21	1.1081464<br>4	0.917	0.255	4.58E-17	11	Crip2
1.96E-21	0.4568931<br>3	0.75	0.159	4.74E-17	11	AU022252
1.99E-21	1.1277630<br>3	1	0.26	4.83E-17	11	Vasn
2.02E-21	0.2729878<br>2	0.722	0.141	4.90E-17	11	Fam43a
2.18E-21	0.3257159<br>1	0.75	0.153	5.29E-17	11	Mrps6
2.70E-21	0.4624133<br>2	0.694	0.144	6.56E-17	11	4632427E13Ri<br>k
2.79E-21	0.3144036<br>2	0.333	0.036	6.77E-17	11	Dact1
2.98E-21	0.2754325<br>5	0.333	0.035	7.23E-17	11	Atp1b1
3.00E-21	0.3074776<br>4	0.611	0.111	7.26E-17	11	Hoxa9
3.09E-21	0.8865434<br>1	0.917	0.254	7.50E-17	11	Twsg1
3.10E-21	0.6411027<br>4	1	0.27	7.52E-17	11	Slc39a13
3.26E-21	0.2629991<br>5	0.5	0.076	7.89E-17	11	Rftn2
3.28E-21	0.4145917<br>8	0.361	0.042	7.94E-17	11	Nrep
3.93E-21	0.7841228<br>8	0.778	0.172	9.53E-17	11	Srpx2

4.23E-21	0.32629786	0.611	0.113	1.02E-16	11	Stard13
4.62E-21	0.30108696	0.694	0.14	1.12E-16	11	Slc35a1
4.72E-21	1.08334878	0.833	0.201	1.15E-16	11	Irx3
4.80E-21	2.22379976	1	0.494	1.16E-16	11	Cited2
4.84E-21	0.65148718	0.889	0.212	1.17E-16	11	Rcn3
5.56E-21	0.86987017	0.972	0.26	1.35E-16	11	Grb10
5.98E-21	0.332525	0.75	0.162	1.45E-16	11	Pdk3
6.62E-21	0.40156918	0.806	0.176	1.60E-16	11	C1qtnf6
7.59E-21	0.58404674	0.778	0.187	1.84E-16	11	Tanc1
7.63E-21	0.30184427	0.639	0.118	1.85E-16	11	Micall2
8.11E-21	1.88179287	0.667	0.132	1.97E-16	11	Dcn
8.61E-21	0.52847098	0.972	0.249	2.09E-16	11	Cdc16
9.38E-21	0.47945857	0.75	0.171	2.27E-16	11	P3h3
9.87E-21	0.30583563	0.694	0.141	2.39E-16	11	Lgalsl
1.20E-20	0.72676819	0.944	0.235	2.90E-16	11	Pls3
1.21E-20	0.66060064	0.861	0.209	2.94E-16	11	Ctdspl
1.42E-20	0.37270996	0.694	0.141	3.45E-16	11	Gpx7
1.61E-20	0.96149235	0.972	0.258	3.90E-16	11	Ryk
1.61E-20	0.66109887	0.944	0.24	3.91E-16	11	Tbrg1
1.62E-20	0.45195395	0.667	0.135	3.93E-16	11	Olfm1
1.65E-20	0.39117179	0.639	0.118	3.99E-16	11	Spry4
1.73E-20	0.80449881	0.917	0.254	4.20E-16	11	Inf2

1.79E-20	0.38346487	0.917	0.228	4.34E-16	11	Abcf2
1.85E-20	0.32891464	0.694	0.142	4.50E-16	11	Smox
1.88E-20	0.43002636	0.778	0.171	4.56E-16	11	Creld2
1.94E-20	0.34787397	0.639	0.124	4.72E-16	11	Hoxc9
2.00E-20	0.45727779	0.778	0.176	4.85E-16	11	Cuedc1
2.02E-20	0.57073594	0.778	0.191	4.91E-16	11	Wwtr1
2.08E-20	0.31867354	0.694	0.14	5.04E-16	11	D16Ertd472e
2.17E-20	0.30709107	0.639	0.123	5.25E-16	11	Antxr2
2.31E-20	1.32831161	0.972	0.345	5.61E-16	11	Foxp1
2.54E-20	1.35795486	0.806	0.203	6.16E-16	11	Slc20a1
2.63E-20	0.58763304	0.778	0.184	6.38E-16	11	Smtn
2.66E-20	0.68117741	0.944	0.265	6.45E-16	11	Arl6ip5
2.69E-20	0.30172559	0.75	0.162	6.53E-16	11	Fbxo9
2.91E-20	0.34443439	0.611	0.118	7.06E-16	11	Hebp2
3.05E-20	0.47297653	0.722	0.153	7.39E-16	11	Myh10
3.20E-20	0.71881483	0.917	0.242	7.77E-16	11	Ikbip
3.45E-20	0.36986747	0.861	0.209	8.37E-16	11	Eci2
3.49E-20	0.31375533	0.667	0.134	8.47E-16	11	Maged2
3.54E-20	0.43948741	0.75	0.168	8.58E-16	11	St3gal2
3.55E-20	0.34624291	0.556	0.097	8.62E-16	11	Stk38l
3.92E-20	0.36069198	0.778	0.171	9.50E-16	11	Yes1

4.09E-20	1.9014071 1	0.972	0.384	9.91E-16	11	Ugdh
4.58E-20	0.4715187	0.778	0.184	1.11E-15	11	Gmds
4.84E-20	0.2520504	0.389	0.05	1.17E-15	11	Cfap69
4.85E-20	0.5113668 7	0.861	0.201	1.18E-15	11	Shox2
5.06E-20	0.2563995 2	0.333	0.037	1.23E-15	11	Sema5a
5.44E-20	1.3938585 1	0.75	0.168	1.32E-15	11	Mt2
6.71E-20	0.6594887	0.917	0.237	1.63E-15	11	Wipi1
6.74E-20	0.3074611 8	0.444	0.067	1.63E-15	11	Tshz2
6.75E-20	0.6781489 1	0.944	0.231	1.64E-15	11	Phlda3
7.57E-20	0.2761566 8	0.556	0.1	1.83E-15	11	Azin2
7.86E-20	0.8987158 9	0.972	0.258	1.91E-15	11	Pcolce
7.99E-20	0.3193507 6	0.361	0.045	1.94E-15	11	Bmper
8.13E-20	0.2729763 9	0.667	0.132	1.97E-15	11	Ebpl
8.52E-20	0.3995322	0.667	0.138	2.06E-15	11	Zfp507
8.70E-20	0.527948	0.722	0.168	2.11E-15	11	Wdr6
9.43E-20	0.9225837 6	0.944	0.276	2.29E-15	11	Dpy19l1
1.04E-19	0.4822845 1	0.944	0.25	2.52E-15	11	Lrp10
1.05E-19	0.5501648 3	0.75	0.182	2.54E-15	11	Orai2
1.16E-19	0.7156128 7	0.944	0.246	2.82E-15	11	Tnfrsf12a
1.21E-19	0.3393867 5	0.639	0.127	2.93E-15	11	Pim3
1.21E-19	0.3639437 7	0.611	0.116	2.93E-15	11	Trib2
1.42E-19	1.6105262 6	0.972	0.286	3.44E-15	11	Sox4
1.42E-19	0.3874639 5	0.667	0.139	3.45E-15	11	Ercc5

1.43E-19	0.30701111	0.75	0.163	3.46E-15	11	Homer3
1.46E-19	0.37127459	0.861	0.21	3.54E-15	11	Gnl3l
1.47E-19	1.32573349	0.944	0.305	3.58E-15	11	Tsc22d1
1.62E-19	0.43367557	0.667	0.141	3.94E-15	11	Magee1
1.63E-19	0.44037817	0.778	0.174	3.95E-15	11	Rfx7
1.74E-19	1.29271766	1	0.317	4.21E-15	11	Ppic
1.81E-19	0.2558508	0.472	0.075	4.38E-15	11	Frk
1.85E-19	0.81811769	0.972	0.289	4.48E-15	11	Ccdc88a
2.07E-19	0.57093367	0.833	0.21	5.03E-15	11	Sertad2
2.12E-19	0.97544188	0.806	0.199	5.13E-15	11	Cdkn2a
2.58E-19	0.32703531	0.889	0.216	6.25E-15	11	Chst12
3.05E-19	0.65213247	0.833	0.209	7.39E-15	11	P3h4
3.42E-19	0.5088445	0.861	0.216	8.29E-15	11	Copz2
3.60E-19	0.84757134	0.861	0.226	8.72E-15	11	Ptk7
3.64E-19	0.60434488	0.917	0.239	8.82E-15	11	Me1
3.67E-19	0.63668857	0.917	0.243	8.90E-15	11	Bcl9l
3.83E-19	0.47286699	0.528	0.094	9.29E-15	11	A4galt
4.02E-19	0.47072361	0.861	0.221	9.74E-15	11	Pdrg1
4.04E-19	1.61805055	1	0.374	9.79E-15	11	Fstl1
4.22E-19	0.31273084	0.833	0.203	1.02E-14	11	Gtf2a2
4.27E-19	0.32288966	0.833	0.201	1.04E-14	11	Adk
4.78E-19	0.28566245	0.694	0.151	1.16E-14	11	Snap47

4.90E-19	0.84092459	0.972	0.253	1.19E-14	11	Ccdc80
4.97E-19	0.27837617	0.611	0.119	1.20E-14	11	Tcf7l1
5.44E-19	0.93074529	0.972	0.309	1.32E-14	11	Impad1
5.57E-19	0.45899105	0.722	0.167	1.35E-14	11	Rarg
6.11E-19	0.32732738	0.694	0.159	1.48E-14	11	Xxylt1
7.13E-19	0.54683648	0.694	0.157	1.73E-14	11	Uaca
8.81E-19	0.65498991	0.778	0.195	2.14E-14	11	Rhobtb3
9.60E-19	0.35548814	0.528	0.092	2.33E-14	11	Tle2
9.61E-19	0.35791165	0.833	0.202	2.33E-14	11	Sap18
1.10E-18	0.25030002	0.639	0.132	2.67E-14	11	Smo
1.11E-18	0.2741646	0.667	0.144	2.69E-14	11	Ahi1
1.25E-18	0.45677223	0.667	0.138	3.03E-14	11	Mustn1
1.31E-18	0.56258381	0.861	0.212	3.17E-14	11	Fbn1
1.36E-18	0.45251946	0.722	0.167	3.31E-14	11	Bmpr1a
1.37E-18	0.39251107	0.694	0.154	3.31E-14	11	Tmem185a
1.52E-18	0.30366802	0.611	0.126	3.69E-14	11	Praf2
1.53E-18	0.2782431	0.611	0.124	3.70E-14	11	Slc19a2
1.54E-18	0.34077229	0.639	0.133	3.73E-14	11	Maml3
1.56E-18	0.39036764	0.583	0.117	3.77E-14	11	Zfp365
1.72E-18	0.36643937	0.528	0.099	4.17E-14	11	Glis3
1.84E-18	0.7643702	0.917	0.273	4.45E-14	11	Nfat5
1.87E-18	0.30464486	0.361	0.048	4.53E-14	11	Dlx2
1.90E-18	0.44116049	0.806	0.19	4.60E-14	11	Epn2

1.93E-18	0.5219576	0.806	0.208	4.68E-14	11	Smad6
2.19E-18	0.27319926	0.694	0.152	5.31E-14	11	Tnfrsf10b
2.46E-18	0.34596793	0.694	0.158	5.97E-14	11	Epm2aip1
2.92E-18	0.37357492	0.806	0.198	7.08E-14	11	Pabpn1
3.44E-18	0.58826521	0.722	0.17	8.34E-14	11	Podnl1
3.57E-18	0.93621973	0.972	0.289	8.65E-14	11	S100a16
3.73E-18	0.30323275	0.75	0.177	9.04E-14	11	Syde1
3.74E-18	0.32217845	0.806	0.192	9.06E-14	11	Otud7b
4.07E-18	0.54473249	0.889	0.238	9.88E-14	11	Mgat1
4.10E-18	0.33195058	0.306	0.035	9.93E-14	11	AC127341.3
4.17E-18	0.3238224	0.917	0.245	1.01E-13	11	Zc3h14
4.40E-18	0.26543834	0.639	0.135	1.07E-13	11	Gm26782
4.43E-18	0.50584363	0.694	0.156	1.07E-13	11	Plpp1
4.56E-18	1.0890212	0.917	0.259	1.11E-13	11	Thbs2
4.66E-18	0.50841864	0.917	0.238	1.13E-13	11	Mtap
4.78E-18	0.3429683	0.667	0.148	1.16E-13	11	Armcx2
5.58E-18	0.33180044	0.889	0.222	1.35E-13	11	Kdm5b
5.58E-18	0.32244332	0.722	0.171	1.35E-13	11	Ift46
5.67E-18	0.26373951	0.333	0.042	1.38E-13	11	Pkia
5.96E-18	0.36740975	0.833	0.216	1.45E-13	11	Cep250
6.06E-18	0.54861526	0.833	0.207	1.47E-13	11	Ripk2
6.15E-18	0.38499531	0.639	0.14	1.49E-13	11	Plscr1
6.21E-18	0.33280594	0.722	0.168	1.51E-13	11	Erc1

6.30E-18	1.05378699	0.972	0.285	1.53E-13	11	Fkbp10
6.79E-18	0.47313788	0.722	0.175	1.65E-13	11	Traf4
7.00E-18	0.99454181	0.944	0.272	1.70E-13	11	Gpc1
7.14E-18	0.52771167	0.833	0.205	1.73E-13	11	Lamb1
7.38E-18	1.40325522	1	0.448	1.79E-13	11	Rbpj
7.38E-18	2.58654356	0.972	0.704	1.79E-13	11	Thbs1
7.46E-18	0.45434093	0.833	0.207	1.81E-13	11	Afap1
7.56E-18	0.35539658	0.806	0.204	1.83E-13	11	Sra1
8.81E-18	0.43311557	0.667	0.154	2.14E-13	11	Ahdc1
8.88E-18	0.42515833	0.583	0.118	2.15E-13	11	Tfdp2
9.15E-18	0.6194611	0.889	0.242	2.22E-13	11	Polr2l
9.84E-18	0.32117172	0.917	0.233	2.39E-13	11	Xpnpep1
1.05E-17	0.2833458	0.583	0.12	2.55E-13	11	Tbc1d8b
1.13E-17	0.26047882	0.667	0.145	2.73E-13	11	Gm14706
1.13E-17	0.69865305	1	0.297	2.75E-13	11	Ttc39b
1.19E-17	0.67381264	0.944	0.278	2.89E-13	11	Gfpt1
1.21E-17	0.27245752	0.722	0.17	2.93E-13	11	Mitd1
1.26E-17	0.86463378	0.944	0.319	3.05E-13	11	Denr
1.27E-17	0.51710722	0.833	0.232	3.08E-13	11	Hsph1
1.27E-17	0.25512538	0.444	0.071	3.08E-13	11	Rarres1
1.36E-17	2.05726857	1	0.634	3.29E-13	11	Fn1
1.37E-17	0.65022724	0.944	0.272	3.32E-13	11	Tsc22d2

1.42E-17	0.35807837	0.667	0.153	3.45E-13	11	Tspan6
1.43E-17	0.49944506	0.917	0.232	3.46E-13	11	Amotl1
1.43E-17	0.38768848	0.889	0.238	3.47E-13	11	Ccser2
1.46E-17	1.28611286	1	0.533	3.54E-13	11	Dap
1.57E-17	0.39474714	0.778	0.197	3.80E-13	11	1810013L24Rik
1.60E-17	0.45850394	0.583	0.124	3.89E-13	11	Ccdc85b
1.69E-17	0.34953088	0.944	0.246	4.10E-13	11	Gna11
1.69E-17	0.28236412	0.889	0.229	4.10E-13	11	Uri1
1.70E-17	0.27519315	0.639	0.143	4.11E-13	11	Klhl26
1.72E-17	0.3372853	0.722	0.172	4.17E-13	11	AI837181
1.83E-17	0.29250386	0.778	0.197	4.43E-13	11	Dvl3
1.86E-17	0.66568871	0.833	0.22	4.52E-13	11	Btg2
1.88E-17	0.27633582	0.75	0.178	4.55E-13	11	Pigp
1.98E-17	0.36486351	0.861	0.224	4.80E-13	11	Faf2
2.03E-17	0.25613518	0.722	0.172	4.93E-13	11	Ublcp1
2.12E-17	0.36479293	0.5	0.093	5.15E-13	11	Lgr4
2.13E-17	0.41221283	0.778	0.191	5.16E-13	11	Tmem63b
2.16E-17	0.44303767	0.611	0.132	5.23E-13	11	Scara3
2.18E-17	0.35268206	0.75	0.183	5.29E-13	11	Nucb2
2.24E-17	0.33689682	0.917	0.251	5.44E-13	11	Pdxdc1
2.30E-17	0.45194648	0.778	0.198	5.58E-13	11	Zcchc14
2.50E-17	0.67691852	0.806	0.211	6.06E-13	11	Mxra8

2.50E-17	0.30573024	0.5	0.093	6.07E-13	11	Acyp2
2.58E-17	1.09293413	0.833	0.231	6.27E-13	11	Olfml2b
2.79E-17	0.37537275	0.889	0.235	6.76E-13	11	Pfkl
3.02E-17	0.52186255	0.917	0.25	7.33E-13	11	Nomo1
3.33E-17	0.29235642	0.75	0.185	8.07E-13	11	Map4k5
3.47E-17	0.67617025	0.944	0.258	8.40E-13	11	Chpf
3.48E-17	0.27908242	0.5	0.09	8.43E-13	11	Smim1
3.84E-17	1.89877687	1	0.448	9.32E-13	11	Lox
3.89E-17	0.4456135	0.806	0.199	9.43E-13	11	Kdelr3
4.23E-17	0.49932499	0.806	0.21	1.03E-12	11	Pla2g4a
4.61E-17	0.32311404	0.75	0.179	1.12E-12	11	Nfatc1
4.77E-17	0.54227234	0.972	0.294	1.16E-12	11	Nufip2
5.08E-17	0.617002	0.889	0.236	1.23E-12	11	Twist1
5.59E-17	0.4787085	0.889	0.247	1.36E-12	11	Dlg1
5.68E-17	0.39180625	0.333	0.043	1.38E-12	11	Grem2
5.77E-17	0.25558845	0.583	0.116	1.40E-12	11	Rnd1
5.79E-17	0.41891963	0.556	0.119	1.40E-12	11	Farp1
6.35E-17	0.38333652	0.778	0.203	1.54E-12	11	Zadh2
6.47E-17	0.35155778	0.611	0.131	1.57E-12	11	Dnm3os
7.14E-17	0.38400864	0.889	0.247	1.73E-12	11	Acp1
7.19E-17	0.34659478	0.806	0.2	1.74E-12	11	Bcar1
7.28E-17	0.28079283	0.833	0.223	1.76E-12	11	Mapkap1
7.37E-17	0.41991263	0.861	0.234	1.79E-12	11	Lix1l

7.63E-17	0.62189076	0.944	0.298	1.85E-12	11	Map3k20
8.04E-17	1.47704475	0.778	0.204	1.95E-12	11	Gas1
8.31E-17	0.32243537	0.389	0.062	2.02E-12	11	Plce1
8.66E-17	0.28923386	0.694	0.163	2.10E-12	11	2310009B15Rik
8.70E-17	0.26759476	0.833	0.204	2.11E-12	11	Yap1
9.31E-17	0.49658418	0.667	0.165	2.26E-12	11	Tram2
9.57E-17	0.6862393	0.944	0.27	2.32E-12	11	Tmem176a
9.86E-17	0.34090106	0.778	0.201	2.39E-12	11	Grpel1
1.01E-16	0.98902369	0.917	0.287	2.44E-12	11	Fam162a
1.03E-16	0.47978895	0.667	0.155	2.51E-12	11	Nbl1
1.05E-16	0.35424387	0.75	0.181	2.55E-12	11	Npdc1
1.05E-16	0.29116396	0.694	0.163	2.55E-12	11	P3h1
1.08E-16	0.34116991	0.889	0.229	2.61E-12	11	Slc38a10
1.09E-16	0.45383561	0.806	0.203	2.65E-12	11	Runx2
1.18E-16	0.36117558	0.917	0.257	2.87E-12	11	Abcd3
1.24E-16	0.74830426	0.889	0.249	3.01E-12	11	Angptl2
1.26E-16	0.44900511	0.639	0.147	3.05E-12	11	Fkbp11
1.28E-16	0.83139136	0.806	0.213	3.11E-12	11	Uap1
1.37E-16	0.31566201	0.611	0.135	3.33E-12	11	Gmppb
1.41E-16	0.51974485	0.917	0.279	3.43E-12	11	Tspan3
1.45E-16	0.36479356	0.722	0.177	3.52E-12	11	Sh3bp4
1.48E-16	0.31394906	0.556	0.117	3.59E-12	11	Zfp9

1.50E-16	0.2923221<br>1	0.667	0.152	3.65E-12	11	Csad
1.54E-16	0.3429722<br>3	0.806	0.207	3.73E-12	11	Txndc15
1.75E-16	0.2551648<br>9	0.778	0.196	4.24E-12	11	Cpped1
1.77E-16	0.3673010<br>9	0.917	0.254	4.30E-12	11	Pros1
1.77E-16	0.4598104<br>1	0.889	0.252	4.30E-12	11	Crim1
1.87E-16	0.3056004<br>4	0.833	0.225	4.54E-12	11	Atl3
1.98E-16	0.8308937<br>2	0.861	0.23	4.80E-12	11	Snai2
2.08E-16	0.4936864	0.861	0.239	5.04E-12	11	Ilf2
2.15E-16	0.3738412<br>3	0.833	0.218	5.22E-12	11	Igf2bp2
2.19E-16	0.4466025<br>7	0.833	0.223	5.32E-12	11	Pdcd11
2.21E-16	0.3051555<br>8	0.861	0.224	5.35E-12	11	Slc4a2
2.23E-16	0.5918386<br>1	0.694	0.165	5.40E-12	11	Hbegf
2.24E-16	0.8433001<br>2	0.917	0.298	5.44E-12	11	Dnajc10
2.38E-16	0.5327107<br>7	0.806	0.232	5.77E-12	11	Uba5
2.60E-16	0.3315395<br>2	0.75	0.192	6.30E-12	11	Stxbp1
2.63E-16	0.3539590<br>8	0.694	0.17	6.37E-12	11	Eml1
2.68E-16	0.3855690<br>8	0.472	0.085	6.51E-12	11	Sod3
2.70E-16	0.4230133	0.778	0.209	6.55E-12	11	Abhd2
2.71E-16	0.3819263<br>2	0.806	0.221	6.57E-12	11	Gigyf2
2.85E-16	0.7475626<br>8	0.75	0.193	6.90E-12	11	Ebf1
2.86E-16	0.8603803<br>3	0.889	0.27	6.92E-12	11	Pdgfa
2.96E-16	0.3537073<br>3	0.694	0.171	7.17E-12	11	Tjp1

2.98E-16	0.2553383<br>1	0.611	0.133	7.23E-12	11	Guf1
3.01E-16	0.5395810<br>2	0.833	0.218	7.31E-12	11	Tmem2
3.03E-16	0.6440155<br>9	0.972	0.3	7.35E-12	11	Mxd4
3.03E-16	0.3053137<br>4	0.833	0.217	7.35E-12	11	Erlec1
3.08E-16	0.6405178<br>5	0.833	0.24	7.46E-12	11	Luzp1
3.09E-16	0.4402235	0.806	0.223	7.49E-12	11	Oaz2
3.16E-16	0.3492797<br>7	0.75	0.192	7.67E-12	11	Dut
3.20E-16	0.2824666<br>8	0.75	0.184	7.75E-12	11	Gm6863
3.33E-16	0.2909068<br>8	0.611	0.134	8.09E-12	11	Bcl9
3.34E-16	0.2744877<br>8	0.611	0.135	8.10E-12	11	Casc4
3.34E-16	0.2875125<br>6	0.556	0.114	8.11E-12	11	Gypc
3.52E-16	0.4371443	0.639	0.144	8.54E-12	11	Cdkn2b
3.56E-16	0.2742893<br>3	0.75	0.194	8.64E-12	11	Sec16a
3.97E-16	0.2594172	0.417	0.07	9.62E-12	11	Dusp14
4.27E-16	0.6031524<br>3	0.556	0.113	1.04E-11	11	Crlf1
4.47E-16	0.4734922<br>5	0.861	0.238	1.08E-11	11	Aen
4.62E-16	0.6794357<br>4	0.694	0.181	1.12E-11	11	Wnt5a
4.84E-16	0.2740051<br>5	0.778	0.195	1.17E-11	11	Arhgap35
4.85E-16	0.7572229<br>9	0.917	0.263	1.18E-11	11	Rcn1
4.99E-16	0.4442259<br>1	0.917	0.268	1.21E-11	11	Sec24d
5.18E-16	0.2836276<br>7	0.528	0.109	1.25E-11	11	Pla2g16
5.25E-16	0.4460476<br>2	0.639	0.144	1.27E-11	11	Grb14
5.30E-16	0.3472770<br>5	0.722	0.177	1.29E-11	11	Adgrl1

5.33E-16	0.65858815	0.889	0.276	1.29E-11	11	Golgb1
5.34E-16	0.2944693	0.694	0.171	1.30E-11	11	Lpcat3
5.94E-16	0.43091044	0.722	0.191	1.44E-11	11	Megf8
6.12E-16	0.27938006	0.889	0.242	1.48E-11	11	Zcchc17
6.26E-16	0.42840735	0.722	0.189	1.52E-11	11	Zfp281
6.88E-16	0.25304328	0.667	0.157	1.67E-11	11	Cfap97
7.24E-16	0.84722715	1	0.357	1.76E-11	11	Tmed9
7.55E-16	0.76543704	0.861	0.232	1.83E-11	11	Bicc1
7.58E-16	0.27421068	0.889	0.25	1.84E-11	11	Lats1
7.64E-16	0.46054175	0.861	0.262	1.85E-11	11	Fam168a
7.82E-16	0.27496121	0.889	0.236	1.90E-11	11	Adpgk
7.88E-16	1.26830968	0.944	0.366	1.91E-11	11	S100a13
7.92E-16	0.56680149	0.889	0.243	1.92E-11	11	Cdc42bpa
8.33E-16	0.38247421	0.833	0.223	2.02E-11	11	Nfkbiz
8.51E-16	0.59766323	0.778	0.204	2.06E-11	11	Klf4
8.83E-16	0.38228558	0.75	0.192	2.14E-11	11	Gm28438
9.32E-16	0.77872264	1	0.286	2.26E-11	11	Rbfox2
9.53E-16	0.85872542	0.889	0.288	2.31E-11	11	Igf1r
1.02E-15	0.29807629	0.472	0.089	2.47E-11	11	Rnf24
1.02E-15	0.32832406	0.5	0.098	2.48E-11	11	Fgfrl1
1.04E-15	0.45401448	0.806	0.225	2.53E-11	11	Bola3
1.06E-15	0.26972753	0.472	0.088	2.57E-11	11	Lrig3

1.06E-15	0.2784223<br>3	0.694	0.167	2.57E-11	11	Cdr2l
1.08E-15	0.2711028<br>2	0.833	0.214	2.61E-11	11	Cblb
1.09E-15	0.4682716<br>9	0.917	0.276	2.63E-11	11	Tnrc6b
1.15E-15	0.4035854	0.861	0.253	2.79E-11	11	Sntb2
1.19E-15	0.2866753<br>2	0.694	0.165	2.88E-11	11	Rora
1.21E-15	0.3730316<br>5	0.778	0.208	2.94E-11	11	Fam92a
1.22E-15	0.2848840<br>2	0.611	0.14	2.96E-11	11	Crybg3
1.24E-15	1.6723557<br>4	1	0.789	3.01E-11	11	S100a6
1.25E-15	0.4257722<br>3	0.944	0.257	3.02E-11	11	Ddr2
1.31E-15	0.2874304<br>1	0.556	0.116	3.18E-11	11	Casp12
1.44E-15	0.3907651<br>1	0.528	0.11	3.50E-11	11	4930523C07Ri<br>k
1.55E-15	0.2527502<br>5	0.806	0.216	3.75E-11	11	Slc44a2
1.61E-15	0.5332392<br>5	0.917	0.271	3.90E-11	11	Myo18a
1.72E-15	0.2514838	0.611	0.138	4.17E-11	11	Gnb5
1.72E-15	1.1793142<br>6	0.889	0.307	4.17E-11	11	Tm4sf1
1.73E-15	0.7189476<br>9	0.917	0.275	4.20E-11	11	Unc5b
1.83E-15	0.2685554<br>7	0.778	0.206	4.44E-11	11	Eef1akmt1
1.90E-15	0.3718150<br>8	0.778	0.205	4.61E-11	11	Chsy1
1.92E-15	0.8636727<br>4	0.528	0.113	4.65E-11	11	Medag
1.94E-15	0.3409762<br>9	0.806	0.224	4.69E-11	11	Naa38
1.99E-15	0.2781813<br>7	0.75	0.19	4.84E-11	11	Ints3
2.04E-15	0.3005525<br>2	0.694	0.176	4.94E-11	11	Rlf

2.04E-15	0.84201028	0.917	0.319	4.95E-11	11	Asap1
2.09E-15	0.5459058	0.917	0.275	5.07E-11	11	Fto
2.12E-15	0.3623003	0.694	0.172	5.15E-11	11	Hmgn2
2.14E-15	0.28701243	0.444	0.082	5.19E-11	11	Ninl
2.20E-15	0.46220448	0.944	0.259	5.34E-11	11	Cttn
2.21E-15	0.25895415	0.639	0.149	5.36E-11	11	Tnks1bp1
2.23E-15	1.48679317	1	0.477	5.42E-11	11	Bgn
2.41E-15	0.73147336	0.444	0.079	5.83E-11	11	Cyp26b1
2.42E-15	0.27418916	0.583	0.128	5.87E-11	11	Tstd3
2.43E-15	0.26271912	0.75	0.193	5.89E-11	11	Bop1
2.46E-15	0.27263219	0.694	0.174	5.96E-11	11	Polr3d
2.51E-15	0.25889152	0.611	0.141	6.09E-11	11	Nfil3
2.54E-15	0.36621897	0.75	0.186	6.15E-11	11	Gpr176
2.57E-15	0.69981133	0.917	0.262	6.23E-11	11	AI506816
2.67E-15	0.60673911	0.944	0.297	6.48E-11	11	Ganab
2.68E-15	0.28682038	0.861	0.237	6.50E-11	11	Ruvbl1
2.72E-15	0.28377952	0.5	0.102	6.61E-11	11	Slc35d1
2.73E-15	0.48787827	0.833	0.231	6.62E-11	11	Thra
2.76E-15	0.29123439	0.361	0.054	6.70E-11	11	Abcb1a
2.81E-15	0.6446545	0.972	0.305	6.81E-11	11	Lman1
2.87E-15	0.61227082	0.917	0.291	6.95E-11	11	Atxn1
2.87E-15	0.28098819	0.667	0.16	6.96E-11	11	Rps19bp1
2.88E-15	0.32425306	0.944	0.271	6.98E-11	11	Dars

2.98E-15	0.4259859	0.861	0.25	7.23E-11	11	Zmpste24
3.10E-15	0.27287276	0.861	0.236	7.52E-11	11	Rnf10
3.11E-15	0.3115428	0.722	0.188	7.54E-11	11	Mpzl1
3.13E-15	0.34734309	0.889	0.257	7.58E-11	11	Minpp1
3.27E-15	0.33122673	0.778	0.209	7.94E-11	11	Zfp260
3.32E-15	0.9065426	0.944	0.341	8.05E-11	11	Wwp2
3.39E-15	0.26993527	0.861	0.241	8.22E-11	11	Thyn1
3.41E-15	0.46798031	0.611	0.146	8.27E-11	11	Map1a
3.54E-15	0.54108892	0.917	0.26	8.59E-11	11	Utrn
3.60E-15	0.29179184	0.583	0.131	8.74E-11	11	Bace1
3.64E-15	1.10504423	0.972	0.351	8.83E-11	11	Aebp1
4.14E-15	0.33052719	0.722	0.188	1.00E-10	11	Dgcr2
4.17E-15	0.60794774	0.806	0.222	1.01E-10	11	Rnd3
4.22E-15	0.35674279	0.778	0.215	1.02E-10	11	Acvr1
4.22E-15	0.32510305	0.694	0.174	1.02E-10	11	Fkbp7
4.40E-15	0.90395809	0.972	0.384	1.07E-10	11	Tspan4
4.85E-15	0.31277074	0.75	0.19	1.18E-10	11	Carnmt1
4.98E-15	0.47218073	0.611	0.151	1.21E-10	11	Bcl2
5.16E-15	0.60940656	0.833	0.252	1.25E-10	11	Golim4
5.37E-15	0.27861387	0.444	0.085	1.30E-10	11	Abca5
5.49E-15	0.69773349	0.806	0.226	1.33E-10	11	Gys1
5.60E-15	0.2727123	0.861	0.22	1.36E-10	11	Fgfr1
5.75E-15	0.29733795	0.833	0.229	1.39E-10	11	Gtf3c1

5.81E-15	0.3288936	0.75	0.19	1.41E-10	11	Ddit4
6.15E-15	0.42511641	0.694	0.189	1.49E-10	11	Mettl1
6.34E-15	0.31358728	0.806	0.218	1.54E-10	11	Tmem97
6.84E-15	0.59971414	0.889	0.263	1.66E-10	11	Nav2
7.19E-15	0.34724949	0.806	0.227	1.74E-10	11	Creb3l2
7.22E-15	0.32367518	0.889	0.25	1.75E-10	11	Cldnd1
7.25E-15	0.67041618	0.944	0.305	1.76E-10	11	Ergic1
7.27E-15	0.28373217	0.75	0.192	1.76E-10	11	Kank2
7.44E-15	0.28063232	0.861	0.247	1.80E-10	11	Mrpl43
7.60E-15	0.27524187	0.833	0.237	1.84E-10	11	Zfr
7.77E-15	0.2513714	0.833	0.235	1.88E-10	11	Fnta
8.08E-15	0.30740139	0.556	0.126	1.96E-10	11	Sspn
8.16E-15	0.4657586	0.972	0.309	1.98E-10	11	Guk1
8.54E-15	0.44202591	0.833	0.246	2.07E-10	11	Klhdc2
8.84E-15	0.84416076	0.889	0.273	2.14E-10	11	Eno1
9.74E-15	0.25649857	0.556	0.122	2.36E-10	11	Optn
9.78E-15	0.46198764	0.972	0.309	2.37E-10	11	Sec13
9.89E-15	0.2577573	0.528	0.112	2.40E-10	11	Mex3a
9.90E-15	0.36985795	0.722	0.199	2.40E-10	11	Mgmt
1.01E-14	0.4374591	0.833	0.236	2.44E-10	11	Lamc1
1.03E-14	0.64993247	0.917	0.296	2.51E-10	11	Mprip
1.06E-14	0.37629419	0.361	0.059	2.57E-10	11	Adam23
1.10E-14	0.32665873	0.556	0.123	2.66E-10	11	Phtf2

1.17E-14	0.34315302	1	0.298	2.83E-10	11	Lsm6
1.19E-14	0.33597117	0.861	0.253	2.88E-10	11	Notch2
1.22E-14	0.29395302	0.722	0.188	2.95E-10	11	Arhgef25
1.23E-14	0.5821272	0.972	0.303	2.98E-10	11	Ier2
1.49E-14	0.80916653	0.972	0.359	3.61E-10	11	Rnf11
1.49E-14	1.21440233	1	0.408	3.62E-10	11	Hspg2
1.50E-14	0.34204967	0.333	0.051	3.64E-10	11	Bmp5
1.53E-14	0.66873644	0.889	0.288	3.71E-10	11	Ttc3
1.56E-14	0.34287094	0.611	0.145	3.78E-10	11	Cd276
1.64E-14	0.39133347	0.917	0.283	3.97E-10	11	Ift20
1.74E-14	1.13106745	1	0.419	4.21E-10	11	Nedd4
1.79E-14	0.32938054	0.861	0.251	4.34E-10	11	Mbtps1
1.79E-14	0.45876616	0.806	0.221	4.35E-10	11	Ephx1
1.88E-14	0.28385469	0.778	0.206	4.56E-10	11	Plk2
1.89E-14	0.28031178	0.667	0.169	4.59E-10	11	Ggps1
2.06E-14	0.3298608	0.75	0.203	4.99E-10	11	Mdn1
2.10E-14	0.36051539	0.694	0.173	5.08E-10	11	Zfhx4
2.17E-14	0.27387424	0.861	0.243	5.26E-10	11	Pgm2
2.18E-14	0.27905006	0.917	0.267	5.29E-10	11	Arl2bp
2.26E-14	0.35247702	0.917	0.264	5.48E-10	11	Sec23a
2.27E-14	0.36955245	0.861	0.257	5.50E-10	11	Smyd2
2.30E-14	0.79970224	0.861	0.286	5.57E-10	11	Enc1

2.34E-14	0.32368935	0.778	0.219	5.67E-10	11	Bag3
2.35E-14	0.32504056	0.722	0.191	5.69E-10	11	Brwd1
2.37E-14	0.58199768	0.611	0.144	5.75E-10	11	Lrrc32
2.51E-14	0.5109538	0.583	0.157	6.09E-10	11	Sh3d19
2.63E-14	0.25578454	0.75	0.199	6.37E-10	11	Scn1b
2.66E-14	0.29671318	0.889	0.261	6.44E-10	11	Fam114a2
2.76E-14	0.27319317	0.694	0.176	6.68E-10	11	P4ha3
2.85E-14	0.33145182	0.722	0.198	6.92E-10	11	Galnt10
2.98E-14	0.38003848	0.778	0.214	7.23E-10	11	Tes
3.01E-14	0.43748793	0.722	0.184	7.29E-10	11	Yae1d1
3.02E-14	0.65844112	0.972	0.329	7.33E-10	11	Rcn2
3.09E-14	0.55175392	0.694	0.195	7.48E-10	11	Ptp4a3
3.09E-14	0.2501457	0.806	0.225	7.49E-10	11	Kdm4a
3.16E-14	0.41447114	0.861	0.268	7.67E-10	11	Ankrd28
3.34E-14	0.30776354	0.639	0.151	8.10E-10	11	Arrdc4
3.68E-14	0.29127872	0.611	0.154	8.93E-10	11	Zeb1
3.81E-14	0.26016879	0.417	0.078	9.23E-10	11	Fat4
3.82E-14	0.34767446	0.889	0.249	9.26E-10	11	Parva
4.11E-14	0.33734809	0.889	0.266	9.98E-10	11	Psmd3
4.55E-14	0.68137021	0.944	0.31	1.10E-09	11	Fosl2
4.56E-14	0.46462063	0.917	0.301	1.11E-09	11	Sav1
4.57E-14	0.30482203	0.472	0.098	1.11E-09	11	Gm43343

4.63E-14	1.30075101	1	0.443	1.12E-09	11	Serpinh1
5.12E-14	0.45567597	0.861	0.254	1.24E-09	11	Lats2
5.20E-14	0.66653464	0.889	0.26	1.26E-09	11	Mrc2
5.21E-14	0.49305558	0.917	0.291	1.26E-09	11	Arhgap5
5.45E-14	0.28722027	0.861	0.255	1.32E-09	11	Ash2l
5.61E-14	0.51924586	0.917	0.279	1.36E-09	11	Tubb4b
5.65E-14	0.37034407	0.722	0.211	1.37E-09	11	Tmem184c
5.92E-14	0.32531463	0.75	0.212	1.44E-09	11	Abcb1b
5.96E-14	0.27488423	0.833	0.239	1.45E-09	11	Helz
6.38E-14	0.45452074	0.944	0.295	1.55E-09	11	Ext2
6.60E-14	0.50080269	0.917	0.287	1.60E-09	11	Tceal8
7.23E-14	1.32881323	1	0.557	1.75E-09	11	Sparc
7.55E-14	0.6920476	0.833	0.239	1.83E-09	11	Sp7
7.99E-14	0.32253126	0.806	0.234	1.94E-09	11	Mrpl54
8.14E-14	0.37064503	0.75	0.202	1.97E-09	11	Ptpn14
8.18E-14	0.45600307	0.944	0.27	1.98E-09	11	Cpe
8.22E-14	0.35630089	0.889	0.244	1.99E-09	11	Rgs3
8.39E-14	0.27267753	0.639	0.171	2.03E-09	11	Ntan1
8.71E-14	0.80895293	0.861	0.276	2.11E-09	11	Ext1
9.23E-14	0.70807216	0.833	0.252	2.24E-09	11	Vgll3
9.25E-14	0.81044806	0.972	0.345	2.24E-09	11	Kdelr2
9.69E-14	0.39095051	0.556	0.136	2.35E-09	11	Pfn2

1.05E-13	0.62451167	1	0.351	2.56E-09	11	Nktr
1.06E-13	0.57242275	0.944	0.309	2.56E-09	11	Srpr
1.09E-13	0.31461328	0.778	0.225	2.65E-09	11	Pde4a
1.11E-13	0.26766308	0.806	0.233	2.68E-09	11	Anapc4
1.20E-13	0.63602035	0.833	0.268	2.92E-09	11	Sulf2
1.23E-13	0.27950291	0.75	0.216	2.99E-09	11	Gcc2
1.28E-13	0.25790801	0.389	0.072	3.10E-09	11	Hoxc11
1.29E-13	0.88761058	0.917	0.284	3.12E-09	11	Serpine2
1.32E-13	0.53840842	0.944	0.312	3.20E-09	11	Pgrmc1
1.39E-13	0.30340225	0.778	0.221	3.36E-09	11	Bri3bp
1.41E-13	0.35451836	0.667	0.184	3.41E-09	11	Hmgn3
1.48E-13	0.25337011	0.75	0.213	3.60E-09	11	Pofut1
1.58E-13	0.40047185	0.778	0.226	3.84E-09	11	Atp8b2
1.58E-13	0.27014555	0.361	0.059	3.84E-09	11	Fosb
1.61E-13	0.78344922	1	0.394	3.92E-09	11	Sec31a
1.67E-13	0.66533905	0.944	0.34	4.06E-09	11	Map2k3
1.71E-13	0.43192951	0.833	0.236	4.15E-09	11	Apbb2
1.76E-13	0.55564778	0.528	0.125	4.26E-09	11	Gm38211
1.76E-13	0.76329447	1	0.346	4.28E-09	11	Selenow
1.80E-13	0.29154307	0.472	0.096	4.36E-09	11	Kitl
1.82E-13	0.29248177	0.694	0.193	4.42E-09	11	Alg5

1.89E-13	0.28619502	0.611	0.155	4.57E-09	11	Bag2
2.01E-13	0.28534106	0.861	0.248	4.88E-09	11	Cd151
2.02E-13	0.27235492	0.75	0.214	4.91E-09	11	Kifap3
2.04E-13	0.26850465	0.861	0.253	4.95E-09	11	Trp53
2.11E-13	0.29698878	0.389	0.071	5.12E-09	11	Htra3
2.16E-13	0.81489964	1	0.388	5.24E-09	11	Txndc5
2.17E-13	0.28216823	0.778	0.225	5.27E-09	11	Klhl21
2.18E-13	0.25277824	0.833	0.24	5.30E-09	11	Rasa2
2.20E-13	0.61432115	0.889	0.297	5.35E-09	11	Ankrd17
2.22E-13	0.35617769	0.694	0.197	5.38E-09	11	Kdelc2
2.34E-13	0.28938536	0.639	0.17	5.66E-09	11	Fmn1
2.38E-13	0.30032998	0.694	0.187	5.78E-09	11	Cbx6
2.39E-13	0.43141707	0.611	0.161	5.80E-09	11	Heg1
2.63E-13	0.28924554	0.75	0.222	6.38E-09	11	Plod1
2.67E-13	0.55299064	0.833	0.255	6.48E-09	11	Ktn1
2.67E-13	0.27406516	0.778	0.227	6.48E-09	11	Mnat1
2.84E-13	0.25086943	0.611	0.156	6.88E-09	11	Ppard
2.84E-13	0.39748262	0.556	0.128	6.89E-09	11	Has2
2.90E-13	0.40619728	0.917	0.288	7.04E-09	11	Map1lc3a
2.91E-13	0.2644327	0.667	0.178	7.06E-09	11	Parp3
2.94E-13	0.35701508	0.806	0.243	7.12E-09	11	Smpd1
3.02E-13	0.39245701	0.778	0.236	7.33E-09	11	Tusc3

3.03E-13	0.31513892	0.861	0.267	7.35E-09	11	Pura
3.23E-13	0.45592997	0.778	0.227	7.83E-09	11	Tgfb1i1
3.58E-13	0.39415134	0.778	0.229	8.67E-09	11	Selenom
3.72E-13	0.54936153	0.917	0.304	9.02E-09	11	Uso1
3.76E-13	0.36572963	0.972	0.321	9.13E-09	11	Park7
3.77E-13	0.73531294	0.917	0.31	9.15E-09	11	Maged1
3.79E-13	0.37655391	0.778	0.24	9.20E-09	11	Zrsr2
3.81E-13	0.25627746	0.806	0.247	9.23E-09	11	Acadvl
3.81E-13	0.38868044	0.778	0.227	9.25E-09	11	Ssr2
3.87E-13	0.39458586	0.444	0.097	9.39E-09	11	Eya4
3.91E-13	0.79539721	0.972	0.365	9.48E-09	11	Sptbn1
4.14E-13	0.281981	0.806	0.244	1.00E-08	11	Acadsb
4.21E-13	0.28582487	0.778	0.224	1.02E-08	11	Emsy
4.44E-13	0.87710147	0.889	0.305	1.08E-08	11	Fscn1
4.77E-13	0.38268283	0.806	0.25	1.16E-08	11	Shc1
4.87E-13	0.41332121	0.917	0.298	1.18E-08	11	Cops2
5.02E-13	0.5572654	0.861	0.277	1.22E-08	11	Pbxip1
5.13E-13	0.37367809	0.694	0.198	1.24E-08	11	Slc20a2
5.18E-13	0.37327712	0.667	0.18	1.26E-08	11	Fads3
5.42E-13	0.38829977	0.861	0.261	1.31E-08	11	Wwc2
5.57E-13	0.39037643	0.75	0.219	1.35E-08	11	Snapc1
5.76E-13	0.34876015	0.889	0.28	1.40E-08	11	Cdk16
6.50E-13	0.4146568	0.889	0.275	1.58E-08	11	Psmd7

6.93E-13	0.34047079	0.667	0.184	1.68E-08	11	Abhd5
6.94E-13	0.40232621	0.75	0.225	1.68E-08	11	Polk
6.96E-13	0.39799751	1	0.344	1.69E-08	11	Nucb1
7.08E-13	0.46964464	0.25	0.033	1.72E-08	11	Igfbp6
7.16E-13	0.272135	0.861	0.275	1.73E-08	11	Cyld
7.27E-13	0.58644011	0.694	0.202	1.76E-08	11	Kcnq1ot1
7.27E-13	0.37895689	0.889	0.286	1.76E-08	11	Cfap36
7.35E-13	0.57313066	0.889	0.272	1.78E-08	11	Msrb3
7.48E-13	1.26627172	1	0.582	1.81E-08	11	Timp2
7.55E-13	0.25586687	0.722	0.203	1.83E-08	11	Akap9
7.74E-13	0.28546483	0.806	0.24	1.88E-08	11	Sec23ip
8.95E-13	0.90296774	0.972	0.355	2.17E-08	11	Pdia4
9.12E-13	0.38234449	0.583	0.149	2.21E-08	11	Socs2
9.32E-13	0.65581894	0.944	0.328	2.26E-08	11	Sfxn1
9.35E-13	0.95354379	1	0.664	2.27E-08	11	Pkm
9.57E-13	0.43856691	0.444	0.094	2.32E-08	11	Enpp2
9.88E-13	0.29929373	0.889	0.277	2.40E-08	11	Timm17a
1.01E-12	0.2677637	0.917	0.282	2.44E-08	11	mt-Atp8
1.02E-12	0.84457343	0.944	0.368	2.48E-08	11	Raph1
1.02E-12	0.56118413	0.833	0.254	2.48E-08	11	Ank
1.05E-12	0.28626799	0.889	0.275	2.55E-08	11	Usp47
1.05E-12	0.25091245	0.75	0.203	2.55E-08	11	Pdgfc

1.08E-12	0.36301064	0.944	0.315	2.62E-08	11	Sec22b
1.09E-12	1.1838272	1	0.489	2.65E-08	11	Ckap4
1.10E-12	0.34745402	0.972	0.341	2.66E-08	11	Dynll2
1.10E-12	0.32423561	0.444	0.1	2.67E-08	11	Wdr35
1.11E-12	0.44361125	0.861	0.272	2.70E-08	11	Sh3pxd2a
1.18E-12	0.35383945	0.556	0.14	2.86E-08	11	H1fx
1.19E-12	0.27852122	0.833	0.237	2.88E-08	11	P4ha2
1.20E-12	0.38747861	0.75	0.223	2.90E-08	11	Il4ra
1.20E-12	0.99336287	0.917	0.317	2.91E-08	11	Col12a1
1.22E-12	0.25942337	0.861	0.277	2.97E-08	11	Gdi1
1.23E-12	0.44932342	0.972	0.34	2.98E-08	11	Copb2
1.32E-12	0.39987746	0.778	0.229	3.21E-08	11	Glipr2
1.33E-12	0.41396686	0.861	0.269	3.22E-08	11	Trove2
1.34E-12	0.25194519	0.583	0.14	3.26E-08	11	Gm11942
1.44E-12	0.54624978	0.944	0.338	3.48E-08	11	Cdc37
1.46E-12	0.40663073	0.861	0.259	3.53E-08	11	Ece1
1.54E-12	0.25995583	0.556	0.138	3.74E-08	11	Tbx15
1.55E-12	0.25811062	0.722	0.212	3.77E-08	11	Imp3
1.61E-12	0.31027535	0.806	0.234	3.91E-08	11	Nckap1
1.71E-12	0.37223578	0.694	0.196	4.16E-08	11	Nectin2
1.75E-12	0.27071597	0.806	0.233	4.23E-08	11	Ddx18
1.75E-12	0.25563974	0.944	0.31	4.23E-08	11	Rbm5

1.78E-12	0.80870229	1	0.457	4.33E-08	11	Fndc3b
1.80E-12	0.34672792	0.722	0.214	4.36E-08	11	Rab34
1.84E-12	0.26862756	0.861	0.27	4.45E-08	11	4932438A13Rik
1.88E-12	0.32400906	0.75	0.226	4.55E-08	11	Exoc4
1.88E-12	0.34490101	0.917	0.291	4.57E-08	11	Tmem147
1.93E-12	0.25661891	0.639	0.171	4.68E-08	11	Arhgef17
1.96E-12	0.27349611	0.639	0.181	4.76E-08	11	Tmem63a
1.99E-12	0.28719501	0.75	0.223	4.82E-08	11	Ptcd3
2.09E-12	0.27434052	0.639	0.172	5.07E-08	11	Spats2
2.10E-12	1.04607199	0.972	0.365	5.10E-08	11	Cavin1
2.11E-12	0.45211065	0.833	0.259	5.12E-08	11	Ppa1
2.13E-12	0.25585048	0.75	0.227	5.16E-08	11	Bex3
2.27E-12	0.38214284	0.806	0.265	5.49E-08	11	Cox20
2.27E-12	0.57073324	0.972	0.36	5.50E-08	11	Cux1
2.29E-12	0.33242825	0.639	0.175	5.56E-08	11	Fam20c
2.42E-12	0.31635188	0.833	0.26	5.86E-08	11	Pcgf3
2.45E-12	0.31335141	0.889	0.29	5.93E-08	11	Ncbp2
2.47E-12	0.3922345	0.833	0.263	5.99E-08	11	Zmat3
2.53E-12	1.98263674	1	0.934	6.12E-08	11	Spp1
2.64E-12	0.6781363	0.972	0.353	6.40E-08	11	Mlec
2.80E-12	0.4647936	0.778	0.246	6.79E-08	11	Cdk14
2.92E-12	0.97301591	1	0.442	7.08E-08	11	Pdia3
3.15E-12	0.60087099	0.944	0.317	7.64E-08	11	Ptprs

3.16E-12	0.37866148	0.861	0.264	7.66E-08	11	Uba52
3.25E-12	0.27840945	0.861	0.27	7.88E-08	11	Tmem120a
3.40E-12	0.81248684	0.972	0.373	8.25E-08	11	Cd81
3.42E-12	0.29958902	0.472	0.11	8.30E-08	11	Tbx18
3.67E-12	1.06657602	1	0.633	8.89E-08	11	Gpx1
3.70E-12	0.25506712	0.861	0.266	8.98E-08	11	Slc25a24
3.72E-12	0.37440712	0.833	0.279	9.01E-08	11	Rraga
4.25E-12	0.60416326	0.972	0.316	1.03E-07	11	2310022B05Rik
4.29E-12	0.43233388	0.917	0.303	1.04E-07	11	Stt3b
4.69E-12	0.3619783	0.667	0.193	1.14E-07	11	Hacd1
4.72E-12	0.42579381	0.722	0.214	1.15E-07	11	Ttc28
4.80E-12	0.29542293	0.972	0.343	1.16E-07	11	Acly
4.84E-12	0.42624709	0.889	0.299	1.17E-07	11	Copg1
5.08E-12	0.31781973	0.917	0.3	1.23E-07	11	Bptf
5.14E-12	0.71681178	0.972	0.322	1.25E-07	11	Cdc42ep3
5.18E-12	0.41122433	0.861	0.299	1.26E-07	11	Ube4b
5.25E-12	0.37842362	0.972	0.334	1.27E-07	11	Tra2a
5.40E-12	0.28912285	0.722	0.222	1.31E-07	11	Anapc13
5.60E-12	0.51661466	0.917	0.296	1.36E-07	11	Vkorc1
6.20E-12	0.27505718	0.389	0.08	1.50E-07	11	Nol3
6.55E-12	0.98341426	0.972	0.494	1.59E-07	11	Calu
6.69E-12	0.25965518	0.778	0.226	1.62E-07	11	Slc35b1

6.72E-12	0.3705786	0.778	0.223	1.63E-07	11	Olfml3
6.91E-12	0.25012585	0.528	0.134	1.68E-07	11	Zfp462
6.96E-12	0.55118241	1	0.381	1.69E-07	11	Arcn1
7.07E-12	0.8926367	1	0.522	1.72E-07	11	Cd9
7.10E-12	0.31659934	0.889	0.298	1.72E-07	11	Spg20
7.12E-12	0.3138145	0.361	0.069	1.73E-07	11	Nptxr
7.18E-12	1.21767685	0.972	0.572	1.74E-07	11	Cyr61
7.31E-12	0.46830904	0.889	0.283	1.77E-07	11	Tmem176b
7.54E-12	0.28473007	0.889	0.269	1.83E-07	11	Tmed3
7.55E-12	0.41213828	0.583	0.159	1.83E-07	11	3110039I08Rik
7.70E-12	0.60126722	0.917	0.339	1.87E-07	11	Kif1b
7.70E-12	0.51532578	0.806	0.254	1.87E-07	11	Slc16a3
7.73E-12	0.42967236	0.917	0.306	1.88E-07	11	Hyou1
8.28E-12	0.34206535	0.778	0.256	2.01E-07	11	Ufsp2
8.66E-12	0.25480429	0.444	0.1	2.10E-07	11	Uggt2
9.05E-12	0.47297248	1	0.381	2.19E-07	11	Tmem167
9.80E-12	0.46671449	0.583	0.157	2.38E-07	11	Ogn
1.05E-11	0.61219935	0.778	0.245	2.55E-07	11	Egr1
1.07E-11	0.51704642	0.861	0.276	2.58E-07	11	Rai14
1.08E-11	0.35646404	0.611	0.172	2.62E-07	11	Gpc4
1.10E-11	0.27339834	0.667	0.198	2.66E-07	11	Ankrd50
1.10E-11	0.28958833	0.833	0.265	2.66E-07	11	Csnk1g3
1.12E-11	0.36418236	0.944	0.308	2.72E-07	11	Snd1

1.13E-11	0.38207102	1	0.707	2.75E-07	11	mt-Nd1
1.14E-11	0.34958814	0.778	0.243	2.76E-07	11	Snhg6
1.17E-11	0.37929706	0.861	0.272	2.84E-07	11	Cfl2
1.18E-11	1.4928653	1	0.911	2.85E-07	11	Malat1
1.20E-11	0.64715703	0.944	0.365	2.91E-07	11	St3gal5
1.21E-11	0.59796299	0.861	0.303	2.94E-07	11	Higd1a
1.24E-11	0.90602656	1	0.55	3.00E-07	11	P4hb
1.30E-11	0.3742188	0.667	0.197	3.16E-07	11	Cavin3
1.31E-11	0.47603506	0.889	0.295	3.17E-07	11	Il6st
1.31E-11	0.31202316	0.833	0.283	3.17E-07	11	Tm2d2
1.55E-11	0.32424448	0.639	0.184	3.75E-07	11	Tex10
1.55E-11	0.25170073	0.833	0.275	3.75E-07	11	Rest
1.56E-11	0.35040596	0.917	0.296	3.77E-07	11	Mydgf
1.58E-11	0.34260396	0.861	0.279	3.83E-07	11	Hars
1.62E-11	0.4842787	0.944	0.367	3.92E-07	11	Cast
1.66E-11	0.28994211	0.75	0.234	4.02E-07	11	Phf14
1.73E-11	0.42996077	0.917	0.326	4.19E-07	11	Kmt2e
1.76E-11	0.32408355	0.972	0.345	4.27E-07	11	Ddx50
1.81E-11	0.2768915	0.889	0.29	4.40E-07	11	Midn
1.91E-11	0.78514403	1	0.403	4.64E-07	11	Ccnd2
1.92E-11	0.3625201	0.944	0.315	4.65E-07	11	Mettl9
2.04E-11	0.37361476	0.861	0.287	4.95E-07	11	Arhgef12
2.10E-11	0.58273586	0.667	0.202	5.10E-07	11	Cebpd

2.28E-11	0.50328573	0.972	0.361	5.53E-07	11	Gtf2h5
2.30E-11	0.62884702	0.917	0.327	5.57E-07	11	Ppib
2.30E-11	0.32271235	0.639	0.188	5.58E-07	11	Lxn
2.39E-11	0.41172718	0.889	0.274	5.78E-07	11	Mmp2
2.81E-11	0.25667307	0.639	0.182	6.81E-07	11	Mthfd1l
3.15E-11	0.7201601	1	0.422	7.65E-07	11	Tnrc18
3.31E-11	0.26802079	0.806	0.266	8.03E-07	11	Thoc2
3.33E-11	0.61224734	0.917	0.318	8.08E-07	11	Dpysl3
3.48E-11	0.26303598	0.806	0.275	8.43E-07	11	Nr3c1
3.49E-11	0.28474736	1	0.355	8.47E-07	11	Crebbp
3.77E-11	0.47291398	0.861	0.298	9.13E-07	11	Dag1
4.01E-11	0.36352169	0.861	0.305	9.73E-07	11	Galk2
4.19E-11	0.94259927	1	0.727	1.01E-06	11	Anxa2
4.27E-11	0.34476795	0.861	0.29	1.04E-06	11	Npm3
4.28E-11	0.53981987	1	0.366	1.04E-06	11	Piezo1
4.39E-11	0.28579383	0.75	0.235	1.06E-06	11	Rras2
4.58E-11	0.28247421	0.917	0.313	1.11E-06	11	Cfdp1
4.72E-11	0.43743288	0.972	0.366	1.15E-06	11	Ell2
4.82E-11	0.25605369	0.556	0.151	1.17E-06	11	Mpdz
4.92E-11	0.34611563	0.917	0.313	1.19E-06	11	Psma4
5.00E-11	0.309316	0.722	0.231	1.21E-06	11	Got2
5.10E-11	0.31120978	0.944	0.324	1.24E-06	11	Mrpl17

5.22E-11	0.25025651	0.833	0.256	1.27E-06	11	Med13l
5.33E-11	0.25531947	0.667	0.202	1.29E-06	11	Yeats2
5.71E-11	0.28251491	0.861	0.275	1.38E-06	11	Taf1d
5.74E-11	0.72136687	0.972	0.373	1.39E-06	11	Plec
5.78E-11	0.26249888	0.583	0.157	1.40E-06	11	Pth1r
5.86E-11	0.25675031	0.722	0.226	1.42E-06	11	Mak16
6.16E-11	0.82318056	1	0.465	1.49E-06	11	Slc38a2
6.18E-11	0.29896943	0.861	0.3	1.50E-06	11	Golga4
6.64E-11	1.0295681	1	0.646	1.61E-06	11	Sdc3
6.76E-11	0.25417701	0.972	0.336	1.64E-06	11	Aebp2
6.98E-11	0.34716555	0.917	0.315	1.69E-06	11	Appbp2
7.06E-11	0.27248014	0.583	0.168	1.71E-06	11	Tmeff1
7.37E-11	0.39014173	0.889	0.32	1.79E-06	11	Prdx6
7.43E-11	0.34760403	0.944	0.326	1.80E-06	11	Edf1
7.69E-11	0.32470355	0.667	0.205	1.86E-06	11	Serf1
7.98E-11	0.26799257	0.806	0.266	1.93E-06	11	Prdx3
8.33E-11	0.2639019	0.833	0.274	2.02E-06	11	Tpst2
8.50E-11	0.54500218	1	0.401	2.06E-06	11	Uba1
8.62E-11	0.33648495	0.889	0.293	2.09E-06	11	Eif4e2
8.66E-11	0.3002844	0.722	0.238	2.10E-06	11	Camkk2
8.69E-11	0.70424168	0.944	0.39	2.11E-06	11	Glg1
9.55E-11	0.26413996	0.722	0.225	2.32E-06	11	Ccdc34
9.56E-11	0.32219091	0.722	0.235	2.32E-06	11	Fam114a1

9.94E-11	0.28913625	0.861	0.299	2.41E-06	11	Cops3
1.00E-10	0.25783952	0.833	0.285	2.43E-06	11	Chd8
1.00E-10	0.25324885	0.917	0.3	2.43E-06	11	Stip1
1.04E-10	0.27700842	0.417	0.094	2.53E-06	11	Gm42047
1.05E-10	0.42329535	0.694	0.212	2.54E-06	11	Iffo2
1.08E-10	0.57259838	0.944	0.397	2.63E-06	11	Pten
1.12E-10	0.35374639	0.972	0.348	2.71E-06	11	Oat
1.13E-10	0.27044612	0.583	0.166	2.73E-06	11	Sqle
1.19E-10	0.52135369	0.861	0.302	2.88E-06	11	Cbx5
1.20E-10	0.68940598	0.972	0.401	2.91E-06	11	Eif4g1
1.23E-10	0.55040038	1	0.376	2.99E-06	11	Prkcsh
1.35E-10	0.30302759	0.944	0.348	3.27E-06	11	Zcrb1
1.36E-10	0.31292838	0.889	0.271	3.30E-06	11	Tead1
1.49E-10	0.48132936	0.972	0.372	3.61E-06	11	Id1
1.55E-10	0.25514573	0.861	0.292	3.76E-06	11	Pigk
1.59E-10	0.83861149	1	0.535	3.85E-06	11	Pebp1
1.59E-10	0.253846	0.667	0.199	3.85E-06	11	Sccpdh
1.64E-10	0.41984135	0.694	0.22	3.98E-06	11	Fhl2
1.65E-10	0.37459977	0.944	0.322	4.00E-06	11	Yipf5
1.80E-10	0.29634895	0.389	0.087	4.36E-06	11	Lif
1.81E-10	0.86621992	1	0.712	4.38E-06	11	Ldha
1.88E-10	0.25092608	0.389	0.091	4.57E-06	11	St5

1.95E-10	0.59653476	0.917	0.347	4.74E-06	11	Pdap1
1.97E-10	0.58398307	0.972	0.388	4.77E-06	11	Ddb1
2.02E-10	0.9304819	1	0.933	4.89E-06	11	Rpl41
2.08E-10	0.34548338	0.833	0.27	5.05E-06	11	Anapc16
2.10E-10	0.77388293	1	0.727	5.09E-06	11	Gnas
2.16E-10	0.30872649	0.5	0.132	5.25E-06	11	Vamp5
2.22E-10	0.28208313	0.806	0.262	5.39E-06	11	Pkn2
2.26E-10	0.4407158	0.917	0.342	5.49E-06	11	Nudcd2
2.43E-10	0.78935005	1	0.482	5.90E-06	11	Hdlbp
2.44E-10	0.36788339	0.972	0.349	5.93E-06	11	Huwe1
2.48E-10	0.40591351	0.917	0.332	6.01E-06	11	Tmem258
2.53E-10	0.77419144	0.778	0.281	6.13E-06	11	Pmepa1
2.71E-10	0.44538769	0.889	0.344	6.57E-06	11	Hectd1
2.75E-10	0.2879389	0.861	0.31	6.67E-06	11	Aff4
2.78E-10	0.92927054	1	0.552	6.73E-06	11	Ppp3ca
2.87E-10	0.71148928	0.806	0.293	6.96E-06	11	Epb41l1
2.88E-10	0.37600837	0.917	0.324	6.98E-06	11	Zc3h15
3.17E-10	0.71121664	1	0.437	7.69E-06	11	Ccng1
3.35E-10	0.31821761	0.972	0.359	8.12E-06	11	Ube2k
3.62E-10	0.41744319	0.417	0.105	8.77E-06	11	Cx3cl1
4.29E-10	0.95449946	1	0.607	1.04E-05	11	Bsg
4.99E-10	1.02503376	1	0.514	1.21E-05	11	Ccnd1
5.42E-10	1.03839023	1	0.727	1.31E-05	11	Hsp90b1

5.49E-10	0.83600685	1	0.396	1.33E-05	11	S100a4
5.50E-10	0.25245243	0.806	0.273	1.33E-05	11	Siva1
6.47E-10	0.55398363	0.972	0.39	1.57E-05	11	B4galt1
7.00E-10	0.31604324	0.861	0.307	1.70E-05	11	Cdk6
7.39E-10	0.39267903	0.917	0.337	1.79E-05	11	Ddost
8.06E-10	1.01702918	0.694	0.254	1.95E-05	11	Xist
8.11E-10	0.27383278	0.917	0.318	1.97E-05	11	Fam3c
8.61E-10	0.40031276	0.944	0.346	2.09E-05	11	H2afv
9.00E-10	0.31495844	0.889	0.322	2.18E-05	11	Lsm12
9.23E-10	0.32965828	0.944	0.354	2.24E-05	11	Mgat2
9.57E-10	0.56446056	0.944	0.419	2.32E-05	11	Ost4
1.07E-09	0.444645	1	0.384	2.59E-05	11	Polr2m
1.11E-09	0.52974876	0.917	0.359	2.69E-05	11	Emp1
1.15E-09	0.98335447	0.972	0.513	2.78E-05	11	Sgk1
1.16E-09	0.27241966	0.917	0.344	2.82E-05	11	Tecr
1.19E-09	0.25260778	0.667	0.224	2.88E-05	11	Pgrmc2
1.21E-09	0.27586607	0.972	0.358	2.92E-05	11	Tnrc6a
1.24E-09	0.25565345	0.889	0.329	3.00E-05	11	Srsf11
1.25E-09	0.36941209	0.917	0.337	3.04E-05	11	Hmgcs1
1.37E-09	0.37182491	0.5	0.141	3.31E-05	11	Dgat1
1.45E-09	0.41591872	0.778	0.286	3.52E-05	11	Ash1l
1.45E-09	0.27953308	0.75	0.251	3.53E-05	11	Bzw2

1.50E-09	0.28689613	0.833	0.298	3.63E-05	11	Prrc2b
1.53E-09	0.39552079	0.944	0.338	3.72E-05	11	Ppfibp1
1.53E-09	0.32838834	0.917	0.334	3.72E-05	11	Top2b
1.54E-09	0.25879201	0.889	0.317	3.73E-05	11	Acbd3
1.54E-09	0.62372441	0.944	0.409	3.73E-05	11	Nfe2l1
1.58E-09	0.43061525	0.972	0.345	3.83E-05	11	Pam
1.63E-09	0.30924968	0.833	0.297	3.95E-05	11	Ddx1
1.75E-09	0.2804433	0.806	0.286	4.25E-05	11	Usp16
2.00E-09	0.30698221	0.861	0.311	4.85E-05	11	Tbl1x
2.15E-09	0.52281811	0.972	0.4	5.22E-05	11	Mbnl2
2.18E-09	0.31499979	0.889	0.342	5.29E-05	11	Smim10l1
2.46E-09	0.54296532	0.528	0.161	5.97E-05	11	Slc16a1
2.69E-09	0.68128412	0.972	0.463	6.52E-05	11	Tns3
2.90E-09	0.2511257	0.889	0.324	7.04E-05	11	Cops5
2.93E-09	0.45553154	0.833	0.322	7.11E-05	11	Rb1cc1
2.96E-09	0.59156503	1	0.406	7.17E-05	11	Rhoc
3.08E-09	0.26717262	0.611	0.199	7.47E-05	11	Prdm2
3.09E-09	0.47051646	0.889	0.358	7.49E-05	11	Gpx4
3.13E-09	0.25908377	0.583	0.184	7.58E-05	11	Mxra7
3.16E-09	0.43630866	0.944	0.376	7.66E-05	11	Dnajc3
3.24E-09	0.30255218	1	0.376	7.86E-05	11	Glrx3
3.61E-09	0.35533598	0.861	0.324	8.74E-05	11	Mrps21

3.68E-09	0.71203428	0.944	0.37	8.93E-05	11	Col5a2
3.76E-09	0.64286851	1	0.403	9.12E-05	11	Ecm1
4.34E-09	0.27382027	0.889	0.332	0.00010516	11	Hook3
4.89E-09	0.2870331	0.806	0.289	0.00011847	11	Galk1
4.97E-09	0.92748321	1	0.582	0.00012043	11	Gm10076
5.03E-09	0.8593232	1	0.694	0.00012202	11	Rps27l
5.14E-09	0.28122341	0.917	0.348	0.00012452	11	Bmpr2
5.60E-09	0.52390237	0.25	0.048	0.00013586	11	Htr2a
5.87E-09	0.34544184	0.917	0.355	0.0001423	11	Srp72
6.28E-09	0.28252336	0.944	0.384	0.00015231	11	Rsrc2
6.44E-09	0.38856478	0.917	0.371	0.00015608	11	Srp19
6.67E-09	0.39164326	0.833	0.309	0.00016164	11	Peak1
6.90E-09	0.28551813	1	0.394	0.00016741	11	Usmg5
7.22E-09	0.53604275	1	0.401	0.00017511	11	Rpl13a
7.53E-09	0.5030404	0.583	0.19	0.0001825	11	Adm
7.58E-09	0.37191477	0.861	0.33	0.00018371	11	Fyttd1
7.78E-09	0.25181775	0.917	0.325	0.00018874	11	Eif3j1
7.88E-09	0.29784726	0.861	0.329	0.000191	11	Elk3
8.69E-09	0.80346222	0.944	0.452	0.0002108	11	Vegfa
8.74E-09	0.42307572	0.944	0.37	0.0002119	11	Strn3
8.78E-09	0.33512957	1	0.383	0.00021294	11	Smarcc1
9.21E-09	0.25820259	0.806	0.313	0.00022341	11	Ivns1abp

9.70E-09	0.41641479	0.917	0.358	0.00023514	11	Myo1c
1.04E-08	0.4544205	0.944	0.41	0.00025185	11	Mrpl33
1.08E-08	0.3739796	0.389	0.106	0.00026094	11	Setbp1
1.22E-08	0.39328825	0.944	0.365	0.00029561	11	Gorasp2
1.26E-08	0.42344191	0.833	0.293	0.00030554	11	Enah
1.27E-08	0.28117192	0.861	0.332	0.00030764	11	Sel1l
1.27E-08	0.50031729	0.972	0.427	0.00030864	11	Cbx3
1.29E-08	0.8987468	1	0.472	0.00031269	11	Timp1
1.29E-08	0.46922505	0.917	0.367	0.00031366	11	Igfbp7
1.30E-08	0.72080569	0.972	0.462	0.0003157	11	Rrbp1
1.31E-08	0.67896247	0.944	0.409	0.00031885	11	Myadm
1.34E-08	0.63849171	1	0.493	0.00032597	11	Sec61b
1.41E-08	0.72885322	1	0.58	0.00034101	11	Tpi1
1.51E-08	0.27301116	0.972	0.358	0.00036545	11	Map4
1.51E-08	0.92970001	1	0.587	0.00036692	11	Pdia6
1.52E-08	0.67755186	0.889	0.356	0.00036798	11	Bnip3
1.54E-08	0.30137925	0.972	0.39	0.00037226	11	Uggt1
1.56E-08	0.31679603	0.861	0.33	0.00037707	11	Arid5b
1.59E-08	0.46217676	1	0.378	0.00038438	11	Qsox1
1.71E-08	0.41305908	1	0.379	0.00041424	11	Col5a1
1.71E-08	0.45877295	0.944	0.393	0.00041535	11	Mrpl52

1.92E-08	0.27561808	0.778	0.296	0.00046459	11	Mrpl57
1.96E-08	0.28523032	0.417	0.118	0.00047515	11	Tmem45a
2.02E-08	0.3317889	0.944	0.379	0.00049079	11	1110004F10Rik
2.22E-08	0.32500762	0.917	0.32	0.00053932	11	Cnn3
2.47E-08	1.31665501	0.528	0.19	0.00059911	11	Cemip
2.67E-08	0.35638889	0.917	0.377	0.00064771	11	Ski
2.81E-08	0.54604936	1	0.502	0.00068192	11	Swi5
2.84E-08	0.6623428	1	0.539	0.00068965	11	Tcf4
3.01E-08	0.48292847	1	0.434	0.00072941	11	Prdx2
3.05E-08	0.6507514	0.583	0.2	0.00073921	11	Hes1
3.19E-08	0.79781582	1	0.803	0.00077373	11	Rps20
3.24E-08	0.40467364	0.944	0.379	0.00078637	11	Zbtb38
3.32E-08	0.26418592	0.889	0.353	0.00080498	11	Dcun1d5
3.47E-08	0.32633903	0.889	0.355	0.00084145	11	Tmem234
3.70E-08	0.36556121	0.861	0.353	0.00089718	11	Magt1
4.26E-08	0.25112363	0.917	0.365	0.00103362	11	Abcf1
4.33E-08	0.26768779	0.417	0.118	0.00104881	11	Chst1
4.59E-08	1.0312951	1	0.662	0.00111346	11	Hspa5
4.66E-08	0.27254762	0.917	0.364	0.00112932	11	Psmd6
4.68E-08	0.66664928	0.972	0.52	0.00113472	11	Neat1
4.88E-08	0.45316364	1	0.425	0.00118234	11	Cdv3

4.97E-08	0.48633561	0.944	0.396	0.00120483	11	Ostc
5.11E-08	0.46773212	0.944	0.41	0.00123903	11	Far1
5.15E-08	0.31020021	1	0.421	0.0012486	11	Snhg8
5.52E-08	0.59736195	1	0.508	0.00133832	11	Rps7
5.78E-08	0.5030793	0.972	0.413	0.00140164	11	Slc25a4
6.06E-08	0.63759653	1	0.503	0.00146835	11	Selenos
6.74E-08	0.65473409	0.778	0.313	0.00163465	11	Col6a3
6.75E-08	0.38111719	0.889	0.347	0.00163695	11	Cyp51
8.36E-08	0.38999737	0.944	0.377	0.00202812	11	Ybx3
8.37E-08	0.36052802	0.917	0.365	0.00202945	11	Sar1a
8.54E-08	0.41138899	0.444	0.132	0.00207021	11	Col3a1
8.78E-08	0.38976132	0.306	0.073	0.00212782	11	Angpt4
8.82E-08	0.34551618	0.917	0.386	0.00213905	11	Ubr5
8.96E-08	0.38453778	0.889	0.383	0.0021721	11	Psmd11
9.39E-08	0.66508622	1	0.553	0.00227588	11	Rpl36a
9.60E-08	0.26228703	0.972	0.375	0.00232875	11	Rpl15
1.18E-07	0.37262918	1	0.445	0.00285008	11	Luc7l2
1.19E-07	0.55423711	0.944	0.457	0.00287723	11	Rpl21
1.21E-07	0.70108577	1	0.784	0.00294375	11	Vim
1.23E-07	0.36727613	1	0.438	0.00297178	11	Ier3ip1
1.24E-07	0.53538469	1	0.466	0.00300188	11	Cald1

1.26E-07	0.66248146	1	0.505	0.00304847	11	Rpl12
1.27E-07	0.25881922	0.778	0.288	0.00307157	11	Samd4b
1.27E-07	0.41783677	0.917	0.383	0.00307566	11	Stt3a
1.30E-07	0.45658419	0.917	0.452	0.00315494	11	Sfr1
1.39E-07	0.43085458	0.5	0.158	0.00336842	11	Adamts1
1.41E-07	0.42240014	0.972	0.446	0.00342784	11	Arf4
1.49E-07	0.28876634	0.944	0.395	0.00360989	11	Bclaf1
1.50E-07	0.43342545	0.917	0.374	0.00364389	11	Loxl3
1.51E-07	0.37350601	0.861	0.345	0.00366027	11	Slk
1.56E-07	0.43406713	1	0.458	0.0037872	11	Cox7c
1.56E-07	0.67262053	0.389	0.111	0.00379317	11	Pdpn
1.73E-07	0.64186417	1	0.49	0.00419214	11	Lmna
1.89E-07	0.68411136	0.944	0.443	0.00457344	11	Csf1
1.96E-07	0.33105789	0.917	0.403	0.00476224	11	Psmc6
2.18E-07	0.28666523	0.972	0.417	0.00528389	11	Ndufv3
2.25E-07	0.2567209	0.944	0.377	0.00544632	11	Rab6a
2.26E-07	0.34742619	0.944	0.383	0.00548895	11	Lpp
2.28E-07	0.26221848	0.972	0.394	0.00552127	11	Cdc34
2.29E-07	0.35453045	0.861	0.371	0.00555339	11	Tmed5
2.36E-07	0.71523771	1	0.603	0.00571571	11	Tmsb10
2.63E-07	0.29666501	0.417	0.124	0.00638111	11	Islr

2.72E-07	0.28638796	0.944	0.387	0.00659947	11	Dhx15
3.00E-07	0.5124667	0.889	0.379	0.00727938	11	Ugp2
3.19E-07	0.27425189	0.917	0.371	0.00774564	11	Eif1ax
3.32E-07	0.29817365	0.917	0.374	0.00804235	11	Anxa6
3.43E-07	0.33055792	0.944	0.44	0.0083087	11	Atrx
3.48E-07	0.60684736	0.972	0.531	0.00843776	11	Eef1g
3.75E-07	0.92557666	1	0.564	0.00909657	11	Tpm1
3.82E-07	0.57720256	1	0.57	0.00925501	11	Rpl10a
4.06E-07	0.31542191	0.75	0.273	0.00984119	11	Prss23
4.31E-07	0.4065669	0.972	0.407	0.0104545	11	Crtap
4.34E-07	0.78271681	0.944	0.548	0.01053229	11	Rps2
4.42E-07	0.25445209	0.944	0.389	0.01071007	11	Ilk
4.57E-07	0.46747568	0.722	0.287	0.01109042	11	Prkar2b
4.63E-07	0.64846782	0.972	0.492	0.01121607	11	Cdkn1a
5.22E-07	0.37330576	0.944	0.448	0.01266423	11	Ube2r2
5.23E-07	0.51622985	1	0.595	0.0126902	11	Tm9sf3
5.28E-07	0.29367284	0.639	0.222	0.012813	11	Efnb1
5.49E-07	0.59030567	1	0.521	0.01330515	11	Clic4
5.97E-07	0.44186397	0.944	0.43	0.01446699	11	Ppp1r14b
6.05E-07	0.39749609	0.917	0.389	0.01466734	11	Mfge8
6.56E-07	0.64801161	1	0.836	0.01590236	11	Rps28
6.75E-07	0.60698091	0.972	0.555	0.01636347	11	Rps18

6.81E-07	0.55223052	1	0.545	0.01651864	11	Rpl17
7.31E-07	0.30667619	0.944	0.4	0.01772066	11	Tulp4
7.42E-07	0.52306482	1	0.585	0.01798932	11	Morf4l2
7.43E-07	0.37125051	0.972	0.479	0.01802425	11	Nisch
7.76E-07	0.74703199	1	0.675	0.01881479	11	Btg1
8.73E-07	0.48685427	0.972	0.497	0.02116864	11	Rps13
9.11E-07	0.53719442	1	0.912	0.02209367	11	Rps8
9.19E-07	0.64635831	1	0.54	0.02229444	11	Rpl35
9.65E-07	0.32277334	0.944	0.447	0.02338848	11	Dynlrb1
9.75E-07	0.51853151	0.917	0.488	0.02364957	11	Gapdh
1.03E-06	0.52425097	1	0.695	0.02496057	11	Ywhae
1.05E-06	0.34343367	0.361	0.11	0.0255086	11	Pdgfra
1.09E-06	0.26684584	0.917	0.396	0.02652561	11	Elob
1.18E-06	0.44586904	1	0.485	0.0285556	11	Sfpq
1.23E-06	0.59367145	1	0.598	0.02976275	11	Dynll1
1.25E-06	0.55889605	1	0.633	0.03037447	11	Dstn
1.27E-06	0.31248999	1	0.459	0.0308841	11	Zfp207
1.38E-06	0.41327929	0.944	0.437	0.03348877	11	Anxa1
1.50E-06	0.37741357	1	0.518	0.03648165	11	S100a11
1.87E-06	0.45454413	1	0.604	0.04528071	11	Atp5e
1.99E-06	0.2732233	0.917	0.436	0.04815622	11	Itpripl2

1.99E-06	0.44799519	0.444	0.15	0.04818745	11	Adamts5
2.03E-06	0.27711772	0.611	0.221	0.04916077	11	Rpl39l
2.04E-06	0.64350699	0.944	0.553	0.04954283	11	Rps15a
2.34E-06	0.55935954	0.944	0.503	0.05669124	11	Rock2
2.36E-06	0.30542187	0.944	0.417	0.05731871	11	Sec61a1
2.53E-06	0.55944655	1	0.878	0.06127118	11	Rps12
2.81E-06	0.54031334	1	0.588	0.06818273	11	Rps17
3.01E-06	0.47465454	1	0.494	0.07309855	11	Ssr1
3.26E-06	0.47973444	1	0.528	0.07892967	11	Hmgn1
3.44E-06	0.35883348	0.944	0.459	0.08352855	11	Atp2a2
3.77E-06	0.59286032	0.944	0.52	0.09144858	11	Mmp14
3.91E-06	0.3891158	1	0.495	0.09476732	11	Prelid1
3.95E-06	0.48683909	1	0.688	0.09576001	11	Actn4
4.08E-06	0.31633655	0.917	0.422	0.09886896	11	Nsa2
4.09E-06	0.30904725	1	0.839	0.09909761	11	mt-Rnr2
4.16E-06	0.25104762	0.917	0.404	0.10083031	11	Psmc3
4.16E-06	0.28858599	0.917	0.457	0.10088534	11	Lamtor4
4.47E-06	0.46901477	1	0.59	0.10838026	11	Uqcr11
4.50E-06	0.7002047	1	0.707	0.10917943	11	Calr
4.58E-06	0.25909108	0.972	0.43	0.11093543	11	Pbrm1
4.70E-06	0.37010145	0.972	0.505	0.11393812	11	Hnrnpm

4.78E-06	0.29983909	1	0.479	0.11597735	11	Mrfap1
5.02E-06	0.32357438	1	0.493	0.12181328	11	Rpl31
5.71E-06	0.44078699	0.972	0.491	0.13849224	11	Hif1a
5.83E-06	0.33847636	0.972	0.469	0.14145418	11	Manf
6.20E-06	0.58006347	1	0.603	0.15022483	11	Rpl6
6.35E-06	1.78829836	0.833	0.528	0.15403004	11	Igfbp4
6.38E-06	0.53989879	1	0.596	0.1546722	11	Rps19
6.49E-06	0.59067907	1	0.661	0.15740228	11	Rps27
7.54E-06	0.2960626	0.944	0.419	0.18292905	11	Dad1
8.87E-06	0.98141741	0.917	0.474	0.21501003	11	Gpx3
9.58E-06	0.54306926	0.972	0.541	0.23236479	11	Rpn1
9.95E-06	0.27130447	0.917	0.414	0.24136069	11	Cct3
1.01E-05	0.53300165	1	0.634	0.24504766	11	Nme1
1.03E-05	0.41773787	0.972	0.489	0.25016676	11	Kdelr1
1.07E-05	0.30327925	0.944	0.498	0.25842655	11	Aldh2
1.19E-05	0.58036268	1	0.621	0.28733639	11	Junb
1.27E-05	0.50327496	1	0.763	0.30844668	11	Rplp0
1.42E-05	0.43641075	0.944	0.517	0.34318893	11	Itgb5
1.52E-05	0.47114786	0.917	0.484	0.36805209	11	Rpl36
1.55E-05	0.26685695	0.944	0.461	0.3768398	11	Metap2
1.68E-05	0.52698646	0.972	0.612	0.40641843	11	Dusp1

1.71E-05	0.45484955	1	0.855	0.41344213	11	Rpl38
1.79E-05	0.40415489	1	0.68	0.43416206	11	Uqcrh
1.95E-05	0.41237732	1	0.545	0.47238198	11	Surf4
2.03E-05	0.26190292	0.972	0.456	0.49176242	11	Hmgb1
2.27E-05	0.61710334	1	0.66	0.5499292	11	Ahnak
2.36E-05	0.35995993	0.972	0.468	0.57328447	11	H13
2.39E-05	0.34719521	0.917	0.464	0.58002905	11	Prrc2c
2.44E-05	0.27827854	0.972	0.466	0.59155294	11	Hnrnpa1
2.86E-05	0.27710405	0.972	0.498	0.69299837	11	Macf1
2.91E-05	0.298274	0.944	0.456	0.7052507	11	Cnih1
2.96E-05	0.4323957	0.972	0.563	0.717805	11	Rpl9
3.07E-05	0.62292533	1	0.544	0.74327748	11	Aplp2
3.08E-05	0.33859241	0.306	0.104	0.74629765	11	Lmo7
3.31E-05	0.32094348	0.972	0.495	0.8015258	11	Tln1
3.38E-05	0.26431557	1	0.501	0.82016458	11	Hnrnph1
3.48E-05	0.30107663	1	0.561	0.84295913	11	Prdx5
3.96E-05	0.2990825	1	0.585	0.95955015	11	Minos1
3.97E-05	0.42335861	1	0.625	0.96302297	11	Rpl3
4.28E-05	0.34059311	1	0.957	1	11	Eef1a1
4.83E-05	0.26072908	0.972	0.477	1	11	Rpl22l1
4.97E-05	0.37600769	0.972	0.6	1	11	Rpl30
4.98E-05	0.30896349	0.944	0.509	1	11	Spcs1

5.14E-05	0.38689643	1	0.522	1	11	Colgalt1
5.54E-05	0.27152983	1	0.505	1	11	Psmb4
6.29E-05	0.36988929	0.972	0.617	1	11	Rpl4
6.42E-05	0.41737066	1	0.848	1	11	Rpl39
6.55E-05	0.48022861	1	0.739	1	11	Pgk1
6.79E-05	0.27091011	0.972	0.539	1	11	Nedd8
7.06E-05	0.46029229	1	0.654	1	11	Rpl28
7.46E-05	0.27581888	0.972	0.509	1	11	Rbm39
7.51E-05	0.26681612	0.944	0.541	1	11	Tomm7
7.66E-05	0.29296508	1	0.537	1	11	Hsp90aa1
8.08E-05	0.32309603	1	0.507	1	11	Xbp1
8.43E-05	0.37180276	1	0.891	1	11	Rpsa
8.52E-05	0.53658764	0.278	0.092	1	11	Ntn4
8.69E-05	0.50199921	0.972	0.777	1	11	Rpl35a
8.91E-05	0.34876112	0.944	0.537	1	11	Chd4
8.95E-05	0.4137924	1	0.714	1	11	Rpl37
9.16E-05	0.77530886	0.778	0.381	1	11	Marcksl1
9.48E-05	0.30499085	1	0.579	1	11	Romo1
0.00010605	0.36163994	0.972	0.597	1	11	Rpl11
0.00010659	0.26340785	0.917	0.42	1	11	Cnn2
0.00010905	0.3231261	1	0.547	1	11	Nptn
0.00010979	0.3524049	1	0.583	1	11	Gas5

0.0001177	0.43314446	0.972	0.678	1	11	Rpl18a
0.00012525	0.36395535	0.944	0.462	1	11	Mt1
0.00012925	0.36061633	0.972	0.581	1	11	Anxa3
0.00014178	0.67416251	0.306	0.107	1	11	Peg10
0.00015372	0.47803921	0.972	0.639	1	11	Cd44
0.00015628	0.25063486	0.944	0.441	1	11	P4ha1
0.00016137	0.3487427	0.972	0.579	1	11	Rpl34
0.00016554	0.38327584	0.944	0.601	1	11	Slc6a6
0.00017374	0.26957451	0.972	0.541	1	11	Rab2a
0.00021506	0.54882003	1	0.703	1	11	Mif
0.0002386	0.41423208	1	0.63	1	11	Rpl29
0.00024282	0.35959972	1	0.619	1	11	Rpl27a
0.00024793	0.37043158	0.972	0.663	1	11	Rps23
0.00027581	0.3905286	0.278	0.095	1	11	Slc7a2
0.00029911	0.79203005	0.25	0.083	1	11	Ccdc163
0.00030909	0.41828632	1	0.708	1	11	Itgb1
0.0003296	0.33228761	0.972	0.541	1	11	Set
0.00035335	0.38925707	1	0.725	1	11	Rps16
0.00037181	0.2965768	1	0.93	1	11	Rplp2
0.00038341	0.33717534	0.944	0.582	1	11	Rpl18
0.0003896	0.31502289	0.972	0.601	1	11	Pgam1

0.00040822	0.33723603	1	0.927	1	11	Rpl37a
0.00044166	0.32989444	1	0.645	1	11	Ssr3
0.0004524	0.56401441	0.667	0.316	1	11	Errfi1
0.00047233	0.25913758	0.917	0.502	1	11	Rpl10
0.00052207	0.26504748	1	0.912	1	11	Rps24
0.00053926	0.31151219	1	0.765	1	11	Rpl22
0.00059041	0.32658077	1	0.903	1	11	Rps21
0.00065668	0.43759732	1	0.928	1	11	Pabpc1
0.00071928	0.31674472	1	0.784	1	11	Rps3a1
0.00075485	0.35629385	1	0.724	1	11	Rpl23a
0.00100214	0.30667954	1	0.739	1	11	Rps3
0.00100334	0.26213601	1	0.561	1	11	Tmed2
0.00102953	0.35432986	0.944	0.665	1	11	Rpl13
0.00102958	0.2679438	0.722	0.324	1	11	Prrx1
0.00106431	0.32822589	0.944	0.475	1	11	Lmo4
0.00108265	0.56920351	0.472	0.216	1	11	Tgfb2
0.00118956	0.3064699	0.972	0.669	1	11	Rps11
0.00139519	0.26895231	1	0.649	1	11	Fus
0.00145695	0.32696583	1	0.72	1	11	Canx
0.00149166	0.3195208	1	0.647	1	11	Rpl7a
0.00152607	0.26714101	1	0.835	1	11	Rps4x

0.00157255	0.25218408	0.944	0.646	1	11	Rpl26
0.00181339	0.3339721	0.389	0.175	1	11	Ccl7
0.0018758	0.30385064	1	0.687	1	11	Rpl14
0.00257903	0.30237723	1	0.785	1	11	Eef1b2
0.00281713	0.26657852	1	0.682	1	11	Btf3
0.00310597	0.31891077	0.889	0.527	1	11	Samhd1
0.00342892	0.34738391	0.667	0.336	1	11	Inhba
0.00414053	0.26228447	1	0.641	1	11	Dbi
0.00440056	0.68340644	1	0.599	1	11	Ctgf
0.00555441	0.28756711	1	0.805	1	11	Rps10
0.00630649	0.33593786	1	0.748	1	11	Lrp1
0.00819887	0.50359982	0.417	0.237	1	11	Rps3a3
0.00863794	0.28508217	1	0.635	1	11	Flna

Supplementary Table 2 Driver genes for different clusters of BMSCs from wildtype mouse femurs
(“C_ ” stands for “Cluster_”)

C_0	C_1	C_2	C_3	C_4	C_5	C_6	C_7	C_8	C_9	C_10	C_11
Rps21	Mef2a	Gpc6	Gpnmb	Gm26917	Actb	Pid1	Cacna2d1	Thsd4	Rps21	Gab2	Sox5
Actb	Gab2	Mmp16	Dock10	Osbpl8	Psma2	Dock10	Gpam	Epha3	Ctss	Alcam	Angpt1
Apobec1	Pid1	Hhat	Rbm47	Lurap1l	Fstl1	Gab2	Lrig1	Ece1	Mrpl52	Cpeb2	Pak3
Ctss	Dock10	Bnc2	Dnmt3a	Ctsc	Prdx1	Fyb	Gpr176	Sparc	Vim	Lrp12	Lrig1
Rpl38	Iqgap2	Fam13c	Lrp12	Unc5c	Pabpc4	Fgd4	Cald1	Prrx1	Cd74	Zdhhc14	Nrn1
Lgals1	Fgd4	Cacna2d1	Fyb	Cd36	Eprs	Iqgap2	Tmem2	Prkca	Ptpn18	Nrros	Styk1
Mrpl52	Frmd4b	Efna5	Igf1	Bicc1	Ak5	Dapk1	Prkca	Rbfox2	Rpl38	Ctsc	Zfp955b
Cd74	Fyb	Slit3	Myo5a	Frmd4b	Lgals1	Myo5a	Pcolce2	Lpar1	Lgals1	S100a6	Efna5
Vim	Myo5a	Pard3	Pid1	Creb3l1	Calm3	Cpeb2	Efna5	Pard3	S100a6	Ptpn18	Cacna2d1
Fth1	Abhd12	Epn2	Dapk1	Tgfb2	S100a6	Dock8	Rem1	Olfml2b	Cdh11	Mef2a	Ror2
Ptpn18	Fli1	Errfi1	Gab2	Inpp5d	Fth1	Gpnmb	Ebf1	Pak3	Actb	Rps21	Prkca
Prdx1	Dock8	Prkca	Fgd4	Pid1	Col1a2	Rbm47	Usp13	Lamb1	Fn1	Fli1	Lgr6
Pf4	Zdhhc14	Cald1	Inpp5d	Gab2	Rps21	Mrc1	Hif1a	Acan	Apobec1	Tpm4	Hmga2
Calm3	Atp8a1	Rbfox2	Alcam	Cybb	Cep126	Frmd4b	Grb14	Apbb2	Postn	Gpnmb	Eps8l2
Psma2	Cpeb2	Grb14	Dock8	Cd300a	Nedd4	Fli1	Gpc6	Satb2	Rock2	Atp2b1	Grem2
Ctla2b	Rbm47	Pcdh19	Iqgap2	Ptprt	Vim	Mef2a	Apbb2	Csf1	Pabpc4	Dstn	Gm6634
Gm5345	Ly86	Setbp1	Nrros	Nrros	Tbx15	Alcam	Depdc1a	Bnc2	Nedd4	Gm15910	Cdc42ep5
S100a6	Osbpl8	Thsd4	Fli1	Zfp13	BC005537	Zdhhc14	Tbx15	Clmp	Prdx1	Fgd4	Cage1
Anxa1	Igf1	Zfp955b	S100a6	Car2	Bgn	Inpp5d	Fam19a2	Setbp1	Anp32b	Dock10	Slc4a4
Fn1	Inpp5d	Kif26b	Dstn	S100a6	Rpl38	Atp8a1	Igf1r	Dock10	Gm16556	Abhd12	Setbp1
Tpm4	Cd36	Ank2	Gsto2	Pmepa1	Tgfb2	Nrros	Hoxc9	Cyp7b1	Mrc1	Iqgap2	Cald1
Pabpc4	Alcam	Lrig3	Zdhhc14	Dnmt3a	11-Sep	Abhd12	Mgat3	Bicc1	Col1a2	Atp8a1	Dync2li1
Tnc	Cybb	Usp13	Tsc22d1	Cdh2	Zfp101	Dstn	Ngf	Ly86	Fth1	Car3	Igf1r
Dstn	Atp2b1	Pdgfc	Frmd4b	Atp8a1	Hs6st2	Abca1	Pard3	Frmd4b	Dstn	Gspt2	Nipal2

Cdh11	Dab2	Gm6634	Atp8a1	Mrc2	Dio3os	Gspt2	Ccdc80	Six1	Timp1	Rbm47	Kcnma1
Clec7a	Dstn	Cdc42bpa	Vim	Lrp12	Timp1	Lrp12	Zfp955b	Syt6	Psma2	Fyb	Gpam
Lcp1	Dapk1	Eef1akmt3	Pla2g7	Neo1	Lgals3	Dab2	Hhat	Gxylt2	Sulf1	Tnn	Spon2
Fabp5	Lrp12	Gli3	Cd300a	Rbfox2	Tnc	S100a6	Lrig3	Gja1	Calm3	Cd84	Col4a5
Tnn	Ctsc	Apbb2	Mrc1	Ptpn18	Uchl1	Itgal	Tln2	Enpp2	Sirpa	Dock8	Sulf1
H19	Pla2g7	Lgr6	Cpeb2	Lcp1	Gsto2	Sirpa	Ptk7	Unc5c	H19	Abca1	Pard3
Mrc1	Clec7a	Lepr	Osbpl8	Tmem45a	Mrpl52	4930452B06Rik	Vegfa	Lurap1l	Pmepa1	Pla2g7	Zfp970
Col8a1	Sema5a	4632427E13Rik	Abca1	Lhfp	Anp32b	Igf1	Slc4a4	Epn2	Tpm4	Dapk1	Lrig3
Eprs	Tmprss4	Satb2	Psap	Prrx1	S100a11	Ptpra	Brsk1	Gm4924	Tnn	Psap	Gpr176
Inhba	Itgal	P3h3	Fam189a1	Ltbp1	Fgfr1	Map2k6	Trib2	Fbln2	Anxa1	Vim	Nebl
Postn	B130034C11Rik	Slc6a15	Ibsp	Mcmdc2	Ebf3	Atp2b1	Ptchd4	Ror2	Ctla2b	Mrc1	Tbx15
Stra6l	Gm10801	Lpar1	Sdc2	Ext1	Col12a1	Gm15327	Fat1	Csrnp3	Alcam	Gm5345	Fam13c
Timp3	Map2k6	Dync2li1	Itga8	Ncam1	Tnfrsf11b	Ly86	Unc5b	Cald1	Tnc	Rufy4	Trib2
Psap	Ptpra	Nmnat2	Fabp5	Srpx2	Cdh11	Fads3	4632427E13Rik	Cdkn2a	Adamts2	B3galt2	Hmgcll1
Col5a1	Abca1	Tbx15	Gm15910	Sorbs1	Zfp385b	Cd36	Dlg5	Gpc6	Psap	Cd36	Rspo3
Col12a1	Nrros	Ptchd4	Ly86	Col8a1	Col6a3	Gm16556	Gm30524	Gm14322	Sparc	Dab2	Gpc6
Car3	S100a6	Brinp3	Car3	BC005537	Srpx2	Fbln2	Syde2	Gli3	Irx5	Pmepa1	Apbb2
Lgals3	Gpnmb	Igf1r	Ptpra	H19	4930593A02Rik	Cd300a	Pak3	Hhat	B3galt2	Zfhx4	Cyp7b1
Col1a2	Gm15327	Cemip	Cd84	Ibsp	Dab2	Pmepa1	Rnd3	1700051A21Rik	4930452B06Rik	Fabp5	Unc5b
Parm1	Gspt2	Brsk1	Abhd12	Tnn	Lgmn	Nxph4	Zfp970	Ltbp1	Dab2	Pid1	Ngf
Kcnma1	Ext1	Gpr176	Clnk	Nlrp5-ps	Sirpa	Dnmt3a	Ebf2	Mapt	Car3	Lcp1	Mgat3
Fyb	Anxa1	Fads2	Gm10801	Sema3d	Ece1	Sema5a	Utrn	Antxr1	Eprs	Inpp5d	Lpar1
Smoc2	BC005537	Rdh13	Bspry	Bspry	Parm1	Serpini1	Styk1	Nmnat2	Fbln2	Anxa1	Baiap2l1

Rock2	4930452B06Rik	Unc5b	Lgmn	Fgf7	Postn	Anxa1	Tnfrsf11b	Vcan	Fabp5	Apobec1	Sync
Cdc42ep3	Cul7	Pgbd5	Gm18113	Frmd6	Col5a2	Car3	Irx3os	Ncam1	Col5a1	Igf1	Eef1akmt3
Zdhhc14	Sema3d	Mgat3	Frmd6	Olfml2b	Car3	Itga8	Rdh13	Gm42303	Col8a1	Calm3	Zdbf2
Cyp1b1	Mrc1	Ptprg	Dcn	Utrn	Actg2	Clnk	Ptprg	Tbx15	Fgfr1	Lgmn	Tmem45a
Rufy4	Bspry	Lhfp	AC163354.1	Dcn	Akap12	Bspry	Epn2	Cdc42bpa	Clec7a	Frmd4b	Hhat
Dio3os	Dnmt3a	Cdkn2a	Anxa1	Dock10	Anxa1	Nebl	Gm26652	Neo1	Smoc2	Col5a1	Ank
Dab2	Gm18113	Bgn	Gm4924	Timp3	Hoxc13	Kcnmb4	Cage1	Fat3	4930593A02Rik	Dcn	Gm47336
Zfp101	H19	Pcolce2	Dnajc6	Adamts2	Ctss	Cdc42ep3	Setbp1	Gem	Gspt2	4930452B06Rik	Cemip
Col11a1	Fads3	Syt12	Aebp1	Dstn	Ctla2b	A930017M01Rik	Runx1t1	Brinp3	Inhba	Myo5a	Pdgfrb
Fbn1	Areg	Col11a1	H19	Cpe	Cd84	AC163354.1	Fbn1	Slit3	Ctsc	Uts2b	Hamp
Zfhx4	Vim	Trib2	Ext1	Satb2	Gm10801	Gm10801	Ror2	Fign	Cd84	Ext1	Ebf1
Tgfb2	Gm11351	Tmem100	Col8a1	Ebf1	Fhl1	Cd84	Cdc42bpa	Nrros	Col5a2	BC005537	4632427E13Rik
Irx5	Uts2b	Pak3	Stra6l	Epha3	Inhba	Fhl1	Slit3	Cp	Rufy4	Prdx1	Ltbp1
Phldb2	Car3	Rem1	4930452B06Rik	Adamts20	Col4a5	Vim	Egfr	Trib2	Gab2	Areg	Brsk1
Actn1	Rufy4	Fign	Hmgcll1	Cd84	H19	Aebp1	3110021N24Rik	Cacna2d1	Pla2g7	Fbln2	Crispld2
Timp1	Clnk	Six1	Itgal	Rbm47	Antxr1	BC005537	Myh10	Utrn	Lcp1	Parm1	Fbn1
Clnk	Lgals3	Slc4a4	Anp32b	Cpeb2	Gm15910	Osbpl8	Pdgfrb	Arhgef40	Itga8	Itga8	Utrn
Itgal	Cend1	Rnd3	Kcnb2	4930452B06Rik	Stra6l	Slc8a3	Lhfp	Fgf10	Uts2b	Tmprss4	Pcolce2
Gm48708	Dcn	Cp	Areg	Fbln2	Grb14	Gm15910	Grem2	Pappa	Acan	4930593A02Rik	Emc1
Gem	Smoc2	Dlg5	Gm5345	Car3	Sfrp4	Clec7a	Bgn	Tmem151a	Dcn	Cpe	Cdc42bpa
Fam20a	Gm16556	Cdh2	Tgfb2	Cdc42ep3	Gm2366	Psap	Hmga2	Myl4	Atp8a1	Inhba	Rbfox2
Fgf7	AC163354.1	Myh10	Mef2a	Pard3	Gm18113	Tsc22d1	Zfp599	Tpbg	Fyb	Fam20a	Tmem2

Anp32b	Sesn3	Gpr137c	Gspt2	Fyb	Gm5345	Cdh11	P3h3	Fxyd1	Parm1	Ly86	Zfp385b
AC163354.1	Ndnf	Gm42303	Tpm4	Lrrc56	Bdnf	B130034C11Rik	Dpep1	Smarca1	Cdc42ep3	Anp32b	Kng2
Csf1	Tpm4	Fam20a	Ncam1	Pla2g7	Klk8	Dcn	Eef1akmt3	Apcdd1	Stra6l	Gm15327	4930471E19Rik
Bspry	Anp32b	Irx3os	Cdh11	Gspt2	Itga8	Pla2g7	Sorbs1	Syt12	Lgals3	Sema3d	Tprg
Gpnmb	Sdc2	Gpam	Lifr	Aebp1	Areg	Myh10	5930430L01Rik	Kif26b	Itgal	Cdh11	Epn2
Sirpa	Gm15910	Ptk7	Atp2b1	Serpini1	Fabp5	Areg	Lmod1	Runx1t1	Lrrc56	Itgal	Fbln1
Areg	B3galt2	Runx1t1	Map2k6	Fabp5	Lrrc56	Sdc2	Unc5c	Gm30524	Areg	Mrpl52	Map2
Rnd3	Tsc22d1	Vdr	Fgf10	Loxl2	Uts2b	Dnajc6	Tmem266	Ccdc116	Csf1	Gm48708	Actn1
Acan	Cyp1b1	3010001F23Rik	Akap12	Igf1	Timp3	Hmgcll1	Vdr	Dync2li1	Klf9	Map2k6	Hapln3
Serpini1	Akap12	Tspan11	Csrnp3	Atp2b1	Triqk	Ptpn18	A430105J06Rik	Wisp2	Timp3	Ptpra	Tnfaip8l3
Atp1b1	B230312C02Rik	Utrn	Lcp1	Areg	Phf24	Gm48708	Lamb1	Lhfp	Gm5345	H19	Col4a6
Loxl2	Gucy1a1	3110021N24Rik	Gm48708	Anp32b	B230208H11Rik	Cybb	Bet1l	Pdgfc	Uchl1	Baiap2l1	Myl3
Ndnf	Cd300a	Cyp7b1	Parm1	Uts2b	Clec7a	Fgf10	Hmgcll1	Fetub	Gm15327	Acan	Unc5c
Sdc2	Slc4a4	Rasl12	Pdgfrb	Gm16833	Ptprd	Cyp1b1	Fbln1	Baiap2l1	Zfhx4	Clec7a	Clmp
Itga8	Fbln2	Gm3830	Lrrc56	Smoc2	B3galt2	Tpm4	Ip6k2	Efs	Ebf3	Gm16556	Dlg5
Loxl3	Pmepa1	2410018L13Rik	Atp1b1	Ptpra	Col5a1	Col3a1	Trpc6	Mgat3	Cyp1b1	Hoxc13	Neo1
Fgfr1	Scube2	Mrc2	Vdr	Antxr1	Acan	Rufy4	Ltbp1	Ank2	Atp1b1	Rpl38	Car12
Cd84	Nxph4	Myl3	Rps21	Myo5a	Rufy4	Cul7	Baiap2l1	Inhba	Antxr1	Car2	Mmp16
Pmepa1	Atp1b1	3010003L21Rik	Pmepa1	4930593A02Rik	Lifr	Lrrc56	Vcl	Gm16141	Pf4	Tgfb2	Vcl
Gm15327	Cdc42ep3	Vat1l	Psma2	Fgf10	Cd36	Gucy1a1	Rbfox2	Efna5	Mcmdc2	Atp1b1	Ptprg
Plod2	Car2	Tmem2	Gucy1a1	Cald1	Gm48708	Lpar1	2010300F17Rik	4933411E08Rik	Plod2	Ebf3	Slc8a3

Dcn	Zfp101	Vcan	Ece1	Acan	Fam186b	Fat1	Cacna1b	Cd276	Serpini1	Gucy1a1	Cp
Lrrc56	Gm8883	Vcl	4930593A02Rik	Alcam	Mef2a	Ndnf	Errfi1	Cage1	Gm48708	Arhgef40	Ppara
Fstl1	Dnajc6	Egfr	Ptprt	Gm18113	Col8a1	Arhgef40	Cpe	Klrb1a	Zfp101	Alpk2	Dpep1
4930452B06Rik	Uchl1	Wisp1	Dio3os	Ror2	Ctsc	Actg2	Hs6st2	Nlrp5-ps	Gm18113	Akap12	Rnd3
Sulf1	Ibsp	Gm12992	Cacna2d1	Psap	Atp2b1	Gm18113	Fam13c	Map2k6	Tgfb2	Antxr1	Rdh13
Ncam1	Ptpn18	Gm16835	Inhba	Brinp3	Pla2g7	Lifr	Pcdh19	Bgn	Fgf7	Rock2	Wasf3
B3galt2	4930593A02Rik	Sesn3	Postn	Fth1	Gm15327	Gm16833	Ank	Ebf1	Col4a5	Col8a1	Pth1r
Alcam	Baiap2l1	Lmo7	Rufy4	Apobec1	Lcp1	Rdh13	Fam189a1	Gm45515	Car2	Actb	Dok6
Nedd4	Nlrp5-ps	Sbsn	Cyp1b1	Ctss	Ebf2	Ibsp	Pex26	A330076H08Rik	Dock8	Gm10801	Lmo7
Gm18113	Aebp1	Pcdh18	Serpini1	Mef2a	Loxl3	Hhat	Kcnb2	Ngf	Abca1	Smoc2	Fat1

Supplementary Table 3 Driver genes for different clusters of BMSCs from Runx2 heterozygous mouse femurs
(“C_ ” stands for “Cluster_”)

C_0	C_1	C_2	C_3	C_4	C_5	C_6	C_7	C_8	C_9	C_10	C_11
Apoe	Runx1	Ddr2	Apobec1	Celf2	Anxa1	Runx1	Pawr	Tbx15	Cx3cr1	Itga9	Gm32618
Prkcb	Sash1	Pard3	Dock10	Nrros	Epb41l3	Plek	Mast4	Col1a2	Lyn	Frmd4b	Zfp423
Ophn1	Plek	Klf12	Apoe	Rasgef1b	Adam12	Sash1	Cblb	Tmtc2	B3glct	Plek	Prr16
Ptpn18	Zfp710	Rbms3	Mitf	Runx1	Pdlim7	Zfp710	Gulp1	Zfp521	Slc9a9	Celf2	AC144408.3
Mrc1	Gab2	Dclk1	Gpnmb	Gab2	Col5a1	Mertk	Nectin3	Tshz3	Ubash3b	Lyn	Tiam2
Fyb	Cd36	Ak5	Atp6v0d2	Tfec	Col1a2	Cd36	Cacna1c	Nxn	Picalm	Runx1	Rab3b
Adgre1	Mertk	Slc2a13	Trf	Itga9	Nnmt	Ubash3b	Arsj	Zfpm2	Ltbp1	Slc9a9	Pcx
Elmo1	Rreb1	Sytl2	Gab2	Stim1	Nfix	1-Mar	Lrig1	Thsd4	Cd36	Bank1	Eya4
Mitf	Mitf	Khdrbs3	Msr1	Arhgap18	Psmd11	Ifi207	Cdon	Fign	Atp6v1b2	Pid1	Tcf7l1
Atf3	Ppm1h	Npas3	Nfib	Ank3	Slit2	Rreb1	Cacnb2	Srgap1	Clec4d	Sash1	Apbb2
Mef2a	Vav3	Satb2	Orai1	Tmcc3	Efemp2	Wwp1	Fam19a1	Cp	Rasgef1b	Mertk	Wwc1
Orai1	Dock2	Cald1	Arhgap10	Lima1	Antxr1	Prkch	Farp1	Zfp948	Ctsc	Lipa	Mbnl3
Plek	Slc9a9	Greb1l	Ptk2b	Arhgap10	Nap1l1	Iqgap2	Gpr176	Ppp2r3a	Pid1	Tlr7	Fam19a1
Arhgap24	Arhgap10	Nhsl1	Vav3	Ttll7	Me1	Picalm	Prkca	Arhgap42	Cenpf	Picalm	Myo10
Lcp1	Fyb	Enah	Gnaq	Fgd4	Psmd1	Pid1	Mtcl1	Robo2	Myo5a	Zfp710	Prr11
Trf	Frmd4b	Pcdh7	Gramd1b	Pid1	Fbn1	Tfec	Smad9	Asap1	Wwp1	Iqgap2	Pdlim1
Iqgap2	Wwp1	Rbfox2	Ttll7	Dock10	Mrc2	Atp2b1	Apol8	Smarca1	Lima1	Cd36	Nav2
Gab2	Gnaq	Tanc1	Nrp1	Rreb1	Map4k4	Mitf	Ube2e2	Ankrd6	Mef2a	Clec4n	Acvr1
Myo5a	Ophn1	Mmp16	Ebf1	Frmd4b	Col1a1	Gnaq	Cdk14	Slc20a1	Pycard	Pdzrn4	Cblb
Clec4a2	Tmcc3	Cdc42bpa	Elmo1	Zdhhc14	Acot7	Frmd4b	Gm17501	Zfp462	Dock2	Rreb1	Sh3d19
Ms4a6c	Iqgap2	Zfpm2	Sash1	Selenoh	Ckap2	Enox2	Eya4	Zeb1	Lpl	Rbm47	Pawr
Man1a	Gramd1b	Plekha5	Fyb	Tnfrsf11a	Dlg5	Fli1	Mbnl3	Dst	Skap2	Inpp5d	Prtg
Arhgap18	Picalm	Eml1	Col3a1	Entpd1	Diaph3	Camk1d	Tead1	Fndc3b	Hk3	Ppm1h	Pcdh7

Arhgap17	Slc16a7	Nbea	Itga6	Nfib	Tmtc2	Orai1	Acvr1	Snx7	Ptpn18	Skap2	Farp1
Gnaq	Rbm47	Myo10	Fli1	Ptk2b	Mrpl33	Gab2	Cep55	Zbtb7c	Igfbp7	Ubash3b	Adgrg2
Atp2b1	Snx24	Robo1	Acer3	Myo5a	Itpr1	Gramd1b	Ankrd28	Fam135a	Runx1	Tpd52	Tbx3
Dock8	Celf2	Tns3	Slc16a10	Ncam1	BC055324	Kcnk13	Gjc1	Nfib	Retreg1	Arhgap10	Tgfbr3
Itga6	Kcnk13	Csmd1	Arhgap17	Sash1	Ank	Vav3	Speg	Lamb1	Tlr7	Lcp2	Prkca
Itgb1	Prkch	Tiam2	Tnfrsf11a	Lyn	Cpe	Slc16a7	Rhoj	Ttc39b	Zfp710	Nrros	Lrrc28
Abhd12	Elmo1	Nhs	Myo5a	Tpd52	Prdm5	Elmo1	Kalrn	Tes	Ccdc88a	Mbnl1	Dpy19l3
Man1c1	Rasgef1b	Pbx1	Hk3	Nrp1	Rpl36	Tlr7	Pbx1	Arfgap3	Nrros	Fgd4	Spsb1
Ccdc80	Nrros	Srgap3	Psap	Phldb2	Timp1	Ppm1h	Ngf	Slc12a2	Frmd4b	Apbb1ip	Nfat5
Col3a1	Enox2	Myo1b	Enox2	Fbxo44	Fth1	Ttll7	St5	Chst15	Rassf4	Gnaq	Smad9
Clec4n	Atp8a1	Sh3d19	Pitpnc1	Mertk	Csrp2	Lyn	Adam12	Rarres1	Iqgap2	Stxbp5	Pbx1
Runx1	Ubash3b	Snx7	Stim1	Dock8	Prdx1	Nrros	Pde3a	Fyn	Camk1d	Fli1	Ggt7
Tec	Ttll7	Agbl3	Selenoh	Rab8b	Serf2	Ms4a6c	Pcx	Syt6	Acer3	Pycard	Cd276
Atp8a1	Pik3cg	Prkca	Fnip2	Dock2	Dstn	Fgd4	Prr16	Fn1	Fxyd1	Gramd1b	Mast2
Osbpl8	Zdhhc14	Mast2	Entpd1	Snx24	Pf4	Rasgef1b	Rhobtb3	Ank2	Nrp2	Rasgef1b	Adam23
Ppm1h	Mbnl1	Sh3rf3	Anxa8	Cx3cr1	Xrcc5	Slc9a9	Prdm16	Nckap1	Gm49027	Adamts5	Nr2f2
Loxl1	Arhgap25	Smurf2	Prkch	Cytip	Map4	Ptpn18	Calu	Aox1	Ugcg	Maf	Arsj
Pik3cg	1-Mar	Wnk3	Tenm3	Parp14	Bgn	Ophn1	Mastl	Aldh7a1	Nap1l1	Kcnk13	Mast4
Camk1d	Bank1	Bicc1	Kif13b	Wdfy4	Col5a3	Dock2	Chn1	Large1	Mt2	Gab2	Eps8
Vav3	Skap2	Eda2r	Osbpl8	Myo1f	Ctnna1	Fyb	Cks2	Arhgap28	Nedd4	Abcg3	Wnk3
1-Mar	Maf	Kalrn	Hlf	Enox2	Malat1	Rbm47	Adam23	Ctif	Dock10	Elmo1	Mcc
Tenm3	Tpd52	Lmo7	Lcp2	Dpysl3	Tbx15	Inpp5d	Nectin1	Evc2	Tbc1d4	Fgfr1	Twist2
Id4	Ifi207	Luzp1	Cd84	Icosl	Nrp1	Tpd52	Tiam2	Boc	Bank1	Stim1	Cemip
Entpd1	Fgd4	Calu	Rpl36	Psap	Aebp1	Cx3cr1	Sh3rf1	Eml1	Myo1e	Arhgap25	Ptprk
Rbm47	Pid1	St5	Fgfr1	Slc9a9	Anxa2	Myo1f	C330027C09Rik	Akap6	Car3	Tfec	Il1r1
Apobec1	Cpeb4	Mcc	Vsig8	Diaph3	Tacc3	Dab2	Glis3	Ift43	Mgat5	Pik3cg	Mllt3

Slc20a2	Tbxas1	Prickle2	Id4	Tenm3	Nrp2	Mgat5	Smurf2	Cdc42bpa	Cdca8	Abca9	Bicc1
Fnip2	Abca9	Ank2	Dock8	Wdfy2	Id3	Snx8	Nbea	Ppm1e	Slc7a8	Ext1	Tes
Enox2	Creb5	Setbp1	Prkcb	Gm26563	Gpc6	Stim1	Esco2	Sh3rf1	Celf2	Ttll7	Mtcl1
Pla2g7	Atp2b1	Asap1	Nedd4	Abca9	Thbs2	Nrp1	Mast2	Epha3	Slc16a10	Dock8	Rarg
Tbc1d4	Fli1	Ube2e2	Arhgap24	Ankrd44	Pcdh19	Prkcb	Parpbp	Mmp16	Kirrel	Ifi207	Sox5
Fgfr1	Snx8	Glis3	Tfec	Nid1	Fgfr1	Snx24	Polr1a	Dpy19l1	Vsir	Slc16a7	Neo1
Anxa8	Ms4a6c	Msrb3	Mt2	Rrm2	Postn	Fgfr1	Arhgef40	Thsd7a	Dstn	Vav3	Glis3
Tmcc3	Stim1	Faxc	Lyst	Ubash3b	Smc4	Cytip	Plekha5	Prrx1	Rreb1	Atp2b1	St5
Fst	Tlr7	Meis2	Cdh19	Vav3	Ltbp2	Ctsc	Fgfr2	B3glct	Cenpe	1-Mar	Cdk6
Fat4	Tsc22d1	Adamts6	Ccdc80	Tbxas1	Olfm2	Man1c1	Anln	Unc5c	Fgfr1	Crtam	Cyp20a1
Thbs2	Arhgap17	Id3	Ldha	Kcnk13	Rspo2	Loxl1	Neo1	Frk	Tenm3	Snx24	Gjc1
Rasgef1b	Itga4	Stard13	Col5a3	Arhgap24	Cenpe	Ankrd44	Esyt2	Trib2	Loxl1	Wdfy2	Prickle2
Snx8	Tfec	Vgll3	Mertk	Gramd1b	Nr2f2	Pip4k2a	Spc25	Sytl2	Col5a3	Wwp1	Tnfaip6
Msr1	Kif13b	Stox2	Tmcc1	Lipa	Col6a2	Skap2	Col4a2	Ebf3	Fst	Cx3cr1	Mboat1
Ubash3b	Inpp5d	Gpr176	Tbxas1	Acer3	1700012B09Rik	Rassf4	Faxc	Dclk1	Sned1	Ptk2b	Ube2e2
Arhgap25	Wdfy2	Plcd3	Tmem154	Mgat5	Nop58	Ext1	Tshz3	Hectd2	Thbs2	Msr1	Eda2r
Cd36	Clec7a	Hoxc5	Dpep1	Sorbs2	Stc2	Hlf	Yap1	Col24a1	Id4	Tmcc3	Reck
Kirrel	Itga9	Ankrd28	Inhba	Esm1	Agrn	Tsc22d1	Msrb3	Sorbs2	Ston2	Hlf	Zfp521
Nedd4	Lyn	Pde4dip	Zdhhc14	Ets1	Unc5b	Dock10	Apbb2	Dlg1	Prkcb	Gpnmb	Ngf
Slc16a7	Ankrd44	Casp12	Ppib	Heyl	Nipal2	Tmcc3	Tcf7l1	Mcc	Bmp1	Psmd1	Csmd1
Kif13b	Atp6v1b2	Chic1	Alkal1	Fli1	Anxa8	Id4	Pard3	Prrc2c	Cdc20	Camk1d	Tjp1
Atp8b4	Cytip	Rnft2	Fscn1	Ly86	Lcn2	Mylip	Nav2	Adarb1	Tec	Selenoh	Nbea
Snx2	Camk1d	Ephx1	Apbb1ip	Fgfr1	Svep1	Anxa8	Plagl1	Arntl2	Nrp1	Snx8	Cdk14
Kcnk13	Arhgap18	Gria3	Wdfy4	Osbpl8	11-Sep	Parp14	Tgfbr3	Slc41a2	Nipal2	Anxa8	Cdon
Selenop	Fgfr1	Galnt13	Ubash3b	Gng11	Fn1	Lipa	Rbms3	Pdgfc	Msr1	Col3a1	Hivep2
Arhgap10	Nrp1	Epn2	Slc40a1	Col5a3	Ebf3	Myo5a	Tead4	Mme	Kcnk13	Prkch	Zfpm2

Slc40a1	Ext1	Kcnt2	Dkk3	Syt14	Ctss	Mrc1	Col4a1	Ube2e2	Fbn1	Col5a3	Tmem44
Tcf4	Wdfy4	Apbb2	Cdc42ep3	Vsir	P3h3	Msr1	P4ha3	Slc2a13	Alcam	Atp8a1	Fermt2
Myo1f	Adgre1	Ptprk	Atp8b4	Cdh2	Ppm1e	Pik3cg	Ubtd2	Adamts12	Dkk3	Zdhhc14	Ptpn14
Hlf	Arhgap24	Mboat2	Heyl	Slc43a2	Pafah1b1-ps1	Celf2	11-Sep	Sertad2	Ctnna1	Itga4	Calu
Phldb2	Man1c1	Tead1	Itga4	Alcam	Cemip	Zdhhc14	Tln2	Esyt2	Apbb1ip	Id4	Enpep
Slc43a2	Prkcb	Irx5	Anxa2	Abcg3	Agtr1a	Pdzrn4	Ptprk	Ptpn13	Aebp1	F13a1	Arhgap42
Fli1	Gpnmb	Arhgap42	Ly75	Mitf	Lgals1	Bank1	Cdca3	Col4a5	Lama4	Tenm3	Apol8
Dock10	Rassf4	Nav2	Olfml3	Lurap1l	Lrrc28	Gpnmb	Socs2	Polr1a	Angpt1	Acer3	Neto2
Cfh	Apbb1ip	P4ha3	Kirrel	Prkar2b	Col6a3	Kif13b	St3gal6	Eda2r	Slc16a7	Golim4	Trabd2b
Prkch	Atp6v0d2	Nxn	Mgll	Rbm47	Lifr	Tenm3	Tanc1	Prdm16	Uhrf1	Wdfy4	Kank2
Mertk	Id4	Tead3	Aebp1	Picalm	Thsd4	Atp6v1b2	Afap1	Tanc1	Anxa8	Dock2	Cacnb2
Nrp1	Cx3cr1	Map4	Tsc22d1	Hs3st1	Npr3	Atp8a1	Prickle2	Uggt2	Mbnl1	Ly75	Pbk
Ncam1	Dkk3	Spats2	Lpar1	Abca1	Mtnr1a	Arhgap10	Ptchd4	Bnc2	Selenoh	Ncam1	Slc9a5
Zfp710	Anxa8	Lrrc28	Snx24	Sgpl1	Ighg2b	Clec7a	Csmd1	Yap1	Lcp2	Fbn1	Parpbp
Gm13091	Ctsc	Sh3rf1	Loxl1	Tgm2	S1pr3	Nfib	Sh3d19	Nhs	Gab2	Pip4k2a	Ddr2
Maf	Tbc1d4	Sgce	Fgd4	Cacna1b	Ldha	Nedd4	Stard13	Ctps	Ttll7	Enox2	Sema3a
Apbb1ip	Orai1	Sobp	Abca1	Plek	Mmp2	Ank	Spats2	Cdk6	Mitf	Car3	BC055324
Pip4k2a	Col3a1	Pard3bos3	Runx1	Igf1	Spc25	Col3a1	Styk1	Col18a1	Sash1	Slc7a8	Col12a1
Snx24	Col5a3	Gm32618	Adamts1	Plxnc1	Cep55	Plxnc1	Reck	Cap2	1-Mar	Olfml3	Creb3l1
Nfib	Lipa	Fign	Cdca8	Sbsn	Ndnf	Apbb1ip	Spsb1	Ninl	Ank	Retreg1	Tns3
Lyst	Tenm3	Tcf7l1	Cytip	Kirrel	Wnt5a	Acer3	Slc9a5	Itgb1	Cdk14	Lifr	Ankrd28
Gpnmb	Msr1	Hoxc4	Skap2	Apbb1ip	Brinp1	Arhgap18	Mllt3	Myef2	Lifr	Cytip	P4ha3
Prdx1	Mef2a	Afap1	Fst	Tlr7	Gm28496	Arhgap17	Pdlim1	Prickle2	Ankrd44	Loxl1	Fndc3b
Mbnl1	Myo5a	Gm17501	Rbm47	Anxa8	Gm30211	Stxbp5	Myo10	Srgap3	Ebf3	Smad7	Esco2
Ttll7	Atf3	Meis1	Man1c1	Nipal2	Areg	Dkk3	Pdlim5	Lama4	Psmd1	Kirrel	Enah

Supplementary Table 4 Driver genes for different clusters of BMSCs from Runx2+/-;Mir338-/- mouse femurs
("C_ " stands for "Cluster_")

C_0	C_1	C_2	C_3	C_4	C_5	C_6	C_7	C_8	C_9	C_10	C_11
Lcp1	Ctss	Csf1	Sirpa	Creg1	Csf1	Ctss	Fn1	Lhfp	Tnnt1	Ctss	B3galt2
Wwp1	Creg1	Col1a2	Creg1	Ctss	Lhfp	Il10rb	Col1a2	Col1a2	Wwp1	BC005537	Tmem25
Fli1	Il10rb	Col1a1	Pid1	Ext1	Col5a2	Lcp1	Csf1	Csf1	Osbpl8	Apobec1	Skap1
Slc43a2	Ext1	Fat1	Tpp1	Ctsc	Fat1	Creg1	Col1a1	Fn1	Lgmn	Ctsc	Cecr2
Creg1	Lcp1	Fn1	Wwp1	Pid1	Col6a3	BC005537	Sec61b	Fgf7	Lcp1	Creg1	Tek
Ctsc	Osbpl8	Ncam1	Cybb	Igf1	Bicc1	Sirpa	Lhfp	Ebf1	Ccl9	Osbpl8	Ncam1
Igf1	Pid1	Fstl1	Fyb	Slc9a9	Utrn	Pid1	Bicc1	Col5a2	Fyb	Slc43a2	Fstl1
Sirpa	Selenop	Loxl3	Gm2a	Mbnl1	Sdc2	Prdx1	Ncam1	Ptprd	Atp6v1a	Pid1	Csf1
Pdlim4	Ctsc	Tead1	BC005537	Tpd52	Fam124a	Osbpl8	Myh11	Utrn	Cd36	Hexa	Grhl1
Osbpl8	Apobec1	Lhfp	Slc43a2	Apobec1	Bmpr1a	Selenop	Bcl9l	Igf1	Ifi207	Cd36	Arl3
BC005537	Lgmn	Apbb2	Mbnl1	Fli1	Tnc	Atp6v1a	Arl3	Fat1	Apobec1	Lcp1	Fn1
Lgmn	Atp6v1a	Myo10	Rab8b	Selenop	Socs5	Mbnl1	Ptk7	Nfix	Ctsc	Ext1	Ltbp1
Atp6v1a	Mbnl1	Ebf1	Cd36	Osbpl8	Loxl3	Slc43a2	Dcbld2	Igfbp7	Cfp	Tmem50a	Sec61b
Ucp2	BC005537	Ltbp1	Dab2	Wwp1	Smad7	Dock10	Frmd6	Cdh2	Slc9a9	Rab8b	Ptk7
Apobec1	Prdx1	Cald1	Igf1	Lcp1	Dcbld2	Dab2	Bgn	Sdc2	Fli1	Mitf	Rpl36
Nrros	Cybb	Prrx1	Atp2b1	Fyb	Actn4	Fam49b	Errfi1	Ammecr1	Rab8b	Cd84	Cdc42bpa
Mbnl1	Hexa	Igf1r	Inpp5d	Atp6v1a	Ext1	Ctsc	Rpl36	Runx2	Lipa	Dock10	Lhfp
Cybb	Psap	Pard3	Mrc1	Psap	Svep1	Fyb	Plin1	Fam49b	Sash1	Unc93b1	Gimap6
Ext1	Atp6v0d2	Cdc42bpa	Ctsc	Mrc1	Igfbp7	Prkcb	Ammecr1	Cdc42bpa	Vav3	Grk2	Bicc1
Rab8b	Dab2	Sec61b	Fli1	Cybb	Cobll1	Ext1	Antxr1	Slit2	Fgd4	Rbm47	1110002J07Rik
Csf1r	Gpnmb	Prkca	Apoe	Grk2	AC154478.1	Apobec1	Slit2	Setbp1	Fnip2	Lamp1	Dpep1
Taldo1	Ggh	Pcdh7	Ctss	Pgd	Prrx1	Slc9a9	Ptprg	Apbb2	Tpd52	Slc9a9	Ptprg
Slc9a9	Lamp1	Bicc1	Dock10	Dock2	Ttc39b	Lgmn	Col5a2	Frmd6	Dock2	Entpd1	Wipi1

Ctss	Fli1	Ptprd	Cd84	Dock10	Ripk2	Cd36	Nfib	Errfi1	Zdhhc14	Fli1	Il18r1
Arpc5	Mrc1	Frmd6	Myh9	Mitf	Phldb1	Psap	Nfix	Efemp2	Ptpn7	Mrc1	Dynlt1f
Dock10	Sirpa	Nfix	Tpd52	Atp6v0d2	Wwc2	Unc93b1	Kirrel	Cemip	Mbnl1	Dab2	Loxl1
Vav3	Slc43a2	Nedd4	Mitf	Abhd12	Cxcl1	Hexa	Adamts2	Hmga2	Eps8	H2-D1	Alpl
Rbm47	Cmtm6	Cdh2	Lcp1	Csf1r	Vcl	Cd84	Nedd4	Vcan	Prkcb	Nrros	Apbb2
Inpp5d	Dock10	Bcl9l	Lipa	Tns3	Nfia	Fgd4	Ece1	Nrros	Iqgap2	Lgmn	Igfbp7
Frmd4b	Prkcb	Bgn	Sash1	Slc43a2	Ccdc80	Gns	Cdh11	Actn1	Lars	Fnip2	Antxr1
Hpcal1	Grk2	Bsg	Cdkn1c	Ptbp3	Nfib	Atp6v1b2	Tnn	Ext1	Sirpa	Abhd12	Plin1
Zdhhc14	Zdhhc14	Smad7	Cd300a	Cd36	Epas1	Fnip2	Ltbp1	Pcdh7	Atp6v1b2	Atp6v1b2	Gm42715
Atp2b1	Unc93b1	Col5a2	Ifi207	Gpnmb	Igf1r	Cybb	Cdh2	Fam32a	Arhgap17	Cyba	Flt1
Gngt2	Cd36	Igfbp7	Selenop	Mertk	Kirrel	Wwp1	Vcan	Loxl3	Igf1	Fyb	Gm35248
H2-D1	Wwp1	Ammecr1	Grk2	Prkcb	Sec61b	Mitf	Socs5	Bgn	Pip4k2a	Akr1a1	Igf1r
Atp6ap2	Atp6v1b2	Vcl	Dapk1	Cltc	Ldha	Plek	Cdc42ep3	Fstl1	Tmem106a	Apoe	Bgn
Unc93b1	Cd84	Ddr2	Slc9a9	Por	Hmga2	Abhd12	Cdc42bpa	Ncam1	Gnas	Prdx1	Nfix
Apoe	Fnip2	Efemp2	Fam49b	Fnip2	Dlc1	Rnf128	Ube2e2	Timp3	Il10rb	Atp6v1a	Vcl
Entpd1	Slc9a9	Errfi1	Prkcb	Map3k1	Cpe	Grk2	Cemip	Col1a1	Slc43a2	Wwp1	Adamts2
Cd36	H2-D1	Glis3	Anxa1	Rock2	Ptprg	Atp2b1	Tead1	Fyn	Plekho2	Ptk2b	Errfi1
Tpd52	Fyb	Nfib	Apobec1	Myh9	Selenoh	Cd300a	Utrn	Adamts2	BC005537	Rreb1	Cdh2
Sash1	Sat1	Mrc2	Rai14	Stk17b	Tmem2	Mef2a	Sparc	Dcbld2	Unc93b1	Tmem65	Kirrel
Wipf1	Igf1	Tm4sf1	Rbm47	Rab8b	Mbnl2	Rassf4	Tbx15	Tead1	Abhd12	Ms4a6c	Vcan
Fyb	Nrros	Nbea	Zdhhc14	Sash1	Peak1	Csf1r	Trabd2b	Inpp5d	Mgat5	Gpnmb	Kank4
Hexa	Abhd12	Socs5	Dock2	Iqgap2	Mef2a	Fam105a	Hdlbp	Peak1	Dock10	Prkcb	Dcbld2
Colec12	Mitf	Col6a3	Fam105a	Gna12	Cald1	Cltc	Cp	Mrc1	Fam105a	Selenop	Nbea
F13a1	Cyba	Rbfox2	Cerk	Vav3	Prelid1	Il6ra	Tanc1	Igf1r	Rbm47	Cotl1	Irs1
Selenop	Rab8b	Ptprg	Celf2	Gnai2	Mprip	Stk17b	Vegfa	Cald1	Fam49b	Rhog	Ogn
Tmem106a	Cd300a	Hdlbp	Unc93b1	Atp6v1e1	Plxdc2	Myo5a	Pcdh7	Col5a1	Rnf130	Fgd4	Sorbs2

Dock2	Taldo1	Grb10	Frmd4b	Clec7a	Snx7	M6pr	Bsg	Myo10	Mef2a	Capza2	Pde3a
Pid1	Vav3	Gpc6	Plekhm1	Fam105a	Tnrc18	Vav3	Irs1	Bicc1	Cerk	Man1c1	Cd226
Fnip2	Mertk	Fgfr1	Ucp2	Lamp1	S100b	Rbm47	Ugcg	Lurap1l	Slc40a1	Ctso	Myh10
Lipa	Ehd4	Ece1	Csf1r	Lgmn	Tns3	Itgb1	Bcl11b	Ptk7	5430437J10Rik	Wipf1	Socs5
Dab2	Atp2b1	Setbp1	Plek	Ktn1	Raph1	Dapk1	Mrpl33	Dock2	Dapk1	Sirpa	Gm26799
Prdx1	Apoe	Adamts2	Arpc2	Fam49b	Myo10	Dock8	Fgfr1	Ddr2	Serinc3	Fam49b	Bcl9l
Cltc	C1qb	Dcbld2	Camk1d	Pycard	Ngf	Lamp1	Rbms3	Socs5	Cd300a	Dapk1	Eml1
Man1c1	Stk17b	Lamc1	Ptbp3	Dock8	Tanc1	Rab8b	Wipi1	Vcl	Fam96a	Fndc3b	Eogt
Ptk2b	Fndc3b	Amotl2	Myo5a	Sirpa	Rbfox2	Sash1	Inhba	Dst	Plek	Vav3	Plekhg6
Mrc1	Akr1a1	Lurap1l	Abhd12	Usp25	Ktn1	Mgat5	Plekhg6	Cerk	Rnf150	Zdhhc14	Erc1
Celf2	Dock2	Utrn	Taldo1	Taldo1	Ppp1r14a	H2-D1	Grb10	Sorbs2	Ppp1r9b	Syk	Frmd6
Mef2a	Plekhb2	Svep1	Lgmn	Sat1	Ppme1	F13a1	Prkca	Cobll1	Por	Rilpl2	Ext1
Fam49b	Atp6v1e1	Ptk7	Cd53	Ifi207	Rere	Ptbp3	Brinp1	Gng2	Itgb1	Arpc2	Ahcyl1
Cerk	Pip4k2a	Rbms3	Vav3	Clec4a1	Smad6	Mrc1	Smad7	Arhgap18	Tspan14	Ifi207	Fat1
Abhd12	Rnf128	Prrx2	Dock8	Itgb1	Gnb4	Tmem50a	Dkk3	Prkca	Cpeb2	Psap	Ugcg
Wdfy4	Fxyd5	Tnc	Enox2	Zdhhc14	Cblb	Fli1	Bmp1	H2-D1	Wipf1	M6pr	Msrb3
Nrp1	Plek	Ube2e2	Mef2a	Csf3r	Arsj	Ifi207	Neo1	Rras2	Akap10	Cd300a	Slc5a3
Atp6v1b2	Tmem50a	Col11a1	Wipf1	Apbb1ip	Nfix	Apoe	Serpine2	Gpc6	Rhog	Selplg	Cdh11
Rreb1	Alcam	Sdc2	Il10ra	C1qb	Sptbn1	Tbc1d9	Prelid1	Antxr1	Rnf128	Mbnl2	Satb1
Mgat5	Rhog	Sptbn1	Fxyd5	Mef2a	Sptan1	Pip4k2a	E330013P04Rik	Tnc	Rreb1	Gns	Umps
Zeb2	Rbm47	Lamb1	Gnaq	Hexa	Cd109	Adgre1	Fyn	Csmd1	Cat	Sash1	Unc5c
Prkcb	Zeb2	Msrb3	Mbnl2	Rnf128	Fam198b	Cotl1	Mrc2	Hspg2	F13a1	Zeb2	Satb2
Grb2	Iqgap2	Ahcyl1	Apbb1ip	Inpp5d	Hspe1	Fndc3b	Dpep1	Arhgef17	Cotl1	Tnfrsf21	Selenoh
Gns	Tns3	Unc5c	Hpcal1	Alcam	Ltbp1	Gpnmb	Ext1	Ctdspl	Mylip	Mertk	Tsc22d1
Por	Cd63	Irs1	Scarb2	Arhgap25	Gpc6	B4galt6	Cald1	Prrx1	Mctp1	Cat	Ift43
Capg	Myo1f	Fgf7	Hexa	Unc93b1	Frmd6	Cmtm6	Col6a3	Kirrel	Wdfy2	Dhrs3	Ece1

Iqgap2	C3ar1	Bmpr1a	Fcho2	Itm2b	Zfhx4	Ms4a6c	Tm4sf1	Nrp1	Atp8a1	Mef2a	Dip2c
Mertk	Atp6ap2	Tpm1	Tns3	Nfia	Otud7b	Il10ra	Loxl1	Ahcyl1	Itgb2	Atp2b1	Mical3
Dapk1	Entpd1	Sfrp4	Adgre1	Rassf4	Tln2	Dram2	Aebp1	Cybb	Cltc	Acer3	Greb1
Atp8a1	Fam49b	Col8a1	Tbc1d9	Man1a	Slc19a2	Zdhhc14	Afap1	Rassf4	Aftph	Bst2	Cox18
Cyba	Mgat5	Wipi1	Rassf4	Plek	Sorbs1	Glmp	Col11a1	Snx7	Cd84	Mcl1	Tanc1
Runx1	Cltc	Cobll1	Rnf150	Atp6v1b2	Crim1	Plekho2	Bmpr1a	Ttc3	Mbnl2	Grb2	Aebp1
Gab2	Fgd4	2900026A02Rik	Iqgap2	Grb2	Magi3	Klhl24	Cox18	Rbfox2	Cd300lb	Irs2	4930509G22Rik
Rassf4	Por	Postn	Nrros	Dapk1	Kdm5b	Usp25	Zeb1	Fndc3b	Hpcal1	Il6ra	Cxcl14
Maf	Fam105a	Hmga2	Atp6v1a	Mbnl2	Tax1bp1	Itga4	Runx2	Fign	Mtss1	Sat1	Nedd4
Pou2f2	B4galt6	Tbx15	Usp25	Msr1	Fndc3b	Dock2	Cxcl14	Ltbp2	Arhgap25	Itm2b	Chsy1
Myh9	Dapk1	Actn1	Rreb1	Nrros	Gcnt2	Npc1	Ptprd	Tln2	Lrp12	Lyst	Ctdspl
Camk1d	Mbnl2	Parva	Psap	Fxyd5	Tead1	Gab2	Fat1	Sec16b	Il10ra	Plekho1	Afap1
Itm2b	Ptbp3	Cp	Pycard	Cmtm6	Pmf1	Gnaq	Adamts6	Ece1	Fcho2	Myo5a	Gm16225
Atp6v0d2	Itgb1	Kirrel	Gab2	Atp2b1	Enah	Grb2	Kalrn	Fap	Pif1	Frmd4b	Tbx15
Fgd4	Grb2	Inhba	Tbxas1	Hpse	Nav3	Rreb1	Hif1a	Cdh11	Fen1	Lrp12	Lmo7
Mitf	Gnai2	Hspg2	Lamp1	Atp6v0a1	Thbs2	Alcam	Glis3	Tmem2	Canx	Cln3	Col5a1
Cd84	Il6ra	Arl3	Nrp1	Fndc3b	Itpr1	Ppm1h	Kdm5b	BC005537	Myo1f	Por	Ube2e2
Adgre1	Csf1r	Dst	Il7r	Cd300a	Actn1	Clec7a	Gria3	Phldb1	Ptk2b	Irf8	Ly6c2
Snx8	Inpp5d	Col12a1	Rab31	Adgre1	Ammecr1	Capza2	Tmem176a	Kdm5b	Ptbp3	Mbnl1	Adgra2
Gng2	Skap2	Sparc	H2-D1	Prdx1	Gm16225	Plekhb2	Fndc3b	Ltbp1	Dock8	Mgat5	Thbs1
Ly86	Itm2b	Kalrn	Prkcd	Plin2	Efemp2	Tnfrsf21	Pard3	Abhd12	Tcf7l2	Slc37a2	Hspg2
Cd53	Map3k1	Cox18	Itgb1	Camk1d	Pdlim5	Dnase1l1	Mical3	Smurf2	Magi3	Igf1	Arhgef18
Slc40a1	Dhrs3	Set	Atp8a1	B4galt6	Myh9	Zfp710	Zfp462	Fbn1	1-Mar	Stk17b	Rhobtb3
Hpgds	Frmd4b	Nav3	Atp6v0d2	Myo1f	Zbtb38	Atp6v0a1	Nbea	Cmtm4	Mitf	Rap1a	Bmp1
Dock8	Adgre1	Mprip	Tle4	Il10ra	Kif2a	Serinc3	Adgra2	Tnn	Raf1	Dock8	Tgfb2

Supplementary Table 5 Significantly changed genes in BMSCs from wildtype, Runx2+/- and Runx2+/-;Mir338-/- mouse femur before osteoblastic induction

ext_gene	wt_D0_1	wt_D0_2	Runx2_het_D0_1	Runx2_het_D0_2	DoubleMutant_D0_1	DoubleMutant_D0_2	cluster
Abat	10.1986	8.37399	6.66787	4.7724	11.0632	10.0678	D0C2
Abcc3	32.5618	27.8088	60.7158	56.9287	37.1222	33.8792	D0C4
Hif1a	573.73	556.15	174.565	219.622	682.74	447.94	D0C2
Acad10	2.82427	2.4272	5.27783	3.56366	2.87694	2.90194	D0C4
Adamtsl3	6.52161	4.12229	3.02989	1.98838	6.28873	5.67514	D0C2
Adm	29.1588	32.7122	47.3964	48.508	40.7967	31.5689	D0C4
Aebp1	680.356	511.664	309.842	251.177	424.708	469.656	D0C2
Ahnak2	30.4119	26.9557	21.0413	15.5537	33.6351	33.973	D0C2
Akap6	1.19458	0.907174	0.503062	0.323059	0.803052	0.865157	D0C2
Angpt4	59.5091	53.3807	20.2528	24.9489	40.5788	41.844	D0C2
Angptl7	6.35828	5.87456	0.76437	0.537849	6.67559	5.61834	D0C2
Aoc3	2.27864	1.85224	0.956033	0.999786	3.4737	2.44536	D0C2
Apoe	4822.03	3990.54	11018.5	10838.9	8360.25	8022.29	D0C4
Aqp1	37.4393	46.4725	66.9308	62.0683	54.521	46.3978	D0C4
Arl5c	2.19828	3.07595	6.85396	7.57206	4.16415	5.01432	D0C4
Aspn	4.51777	2.79808	13.3503	15.6101	24.369	34.5148	D0C1
Atp10d	2.83494	1.30827	6.07171	3.33655	2.60119	2.37282	D0C4
Bmp8a	9.7553	4.64473	1.7442	1.24228	3.18562	4.27272	D0C2
Cadm1	39.7295	34.4662	55.907	51.641	48.1842	49.0296	D0C4

Car8	5.5959	5.85543	12.7165	13.3914	8.96797	8.66679	D0C4
Cat	557.73	508.517	754.957	621.05	594.852	569.607	D0C4
Cbr2	17.9901	19.6859	6.91172	7.27926	16.3838	18.4814	D0C2
Cd36	245.326	151.091	325.834	265.723	255.575	190.7	D0C4
Cd37	26.6027	31.3473	50.7655	48.3573	32.9415	42.2801	D0C4
Cd5l	56.2252	55.6162	117.556	144.295	80.0291	75.5039	D0C4
Cd68	731.168	657.776	900.314	867.828	817.733	785.129	D0C4
Cd74	18.6268	16.8616	5.03662	4.48425	1.47562	2.76115	D0C3
Cemip	62.8718	42.0149	23.0628	14.2193	42.6633	44.6161	D0C2
Chn2	6.57246	4.88566	2.46876	1.87651	4.71139	3.86015	D0C2
Chst15	4.6283	4.4264	8.02811	6.47964	5.17568	5.06959	D0C4
Cnbd2	9.54435	12.9016	3.60534	2.88631	8.10762	6.81545	D0C2
Col18a1	8.11853	8.20846	15.305	15.174	10.8812	12.1311	D0C4
Col6a1	169.695	126.442	85.7199	89.8603	129.644	136.818	D0C2
Colec12	122.504	105.634	157.985	122.205	267.886	245.638	D0C1
Csf2rb2	17.479	16.9269	38.8736	32.2129	29.8874	33.0122	D0C4
Cxcr4	17.0476	17.0102	37.3418	35.909	24.6063	31.8108	D0C4
Dhrs7	26.0675	29.5365	45.1567	45.8469	35.0738	38.932	D0C4
Dhx40	19.5946	17.7062	12.6714	10.3591	21.4806	22.8007	D0C2
Dusp1	89.6123	108.298	139.94	126.181	117.073	96.1042	D0C4
Dysf	21.925	18.589	10.4409	10.0582	17.8396	21.3416	D0C2

Egr1	48.2262	45.4705	73.0407	99.1725	61.2744	55.8591	D0C4
Egr2	4.85732	5.16252	8.11049	9.01663	6.46871	6.36194	D0C4
Entpd1	16.4817	13.9756	9.89617	9.30362	24.3357	28.2648	D0C2
Entpd4	23.646	14.2423	10.1259	6.93345	15.9518	16.7375	D0C2
Erdr1	2.23966	2.32261	102.619	81.2081	76.2439	63.374	D0C4
Ereg	3.13001	3.18134	6.74867	5.32073	3.03487	3.24816	D0C4
F2r	11.1693	9.91457	24.1315	18.9935	43.5651	39.4575	D0C1
Fam46b	0.735085	0.818457	1.93251	1.44808	0.787106	0.92394	D0C4
Fcgr4	12.1239	12.7175	22.8357	22.528	17.0031	16.9465	D0C4
Fmod	144.485	95.5326	62.0042	53.5015	113.822	114.586	D0C2
Fos	71.8175	68.073	123.175	117.754	95.3501	89.9047	D0C4
Fzd5	20.1057	16.1015	12.8718	10.2401	19.7473	18.751	D0C2
Galnt18	38.3768	42.1735	71.3387	75.5693	40.4437	41.2201	D0C4
Gle1	12.4419	10.5824	6.94465	6.7028	11.1885	11.4891	D0C2
Gm10184	11.4489	12.5185	41.6933	41.321	11.653	9.83957	D0C4
Gm13835	43.3934	46.1635	97.6781	110.636	59.7527	57.6098	D0C4
Gm14548	2.61936	1.88623	0.797191	0.925781	2.62812	2.74705	D0C2
Gm15280	273.586	307.356	95.5544	76.1778	153.395	223.09	D0C2
Gm15920	9.52454	12.4459	25.0541	26.2404	10.8161	16.3808	D0C4
Gm16867	8.96196	8.6973	0.776399	0.3965	5.07911	6.18062	D0C2
Gm21887	6.91363	6.35991	148.499	137.01	51.664	40.2264	D0C4

Gm35315	1.04599	0.80792	0	0	0.803514	0.68523	D0C2
Gm46223	534.192	443.437	267.194	200.877	378.675	409.97	D0C2
Gm5905	13.1232	14.4781	0	0.381379	5.70782	7.78296	D0C2
Gm6768	20.8106	15.686	0	0	24.1809	24.0151	D0C2
Gm6969	80.5909	58.8248	0.875707	0	82.9901	57.4509	D0C2
Gm9625	10.1543	16.4987	26.3253	36.7546	5.02761	5.46072	D0C4
Gna12	75.3328	80.1083	104.302	118.398	75.9039	83.3909	D0C4
Gpr84	30.823	30.4486	19.6998	18.7949	11.5803	14.3524	D0C3
Grb14	52.8162	54.3312	35.2526	37.3925	66.4127	68.033	D0C2
Gxylt2	11.1973	7.36101	5.77618	4.91895	7.97529	10.082	D0C2
Hgsnat	190.634	186.641	354.168	271.83	126.548	126.072	D0C4
Hist1h4n	83.0563	39.0947	17.0009	10.751	50.1917	58.6516	D0C2
Hmga2	27.0844	25.4608	50.0143	33.6935	33.5863	31.6472	D0C4
Htr1b	4.48017	4.80229	7.30941	5.90613	5.31774	4.59272	D0C4
Idua	17.3124	15.3037	10.9233	10.8493	21.7639	22.0286	D0C2
Igf2	7.17652	8.12196	17.0173	9.7871	35.2518	21.7051	D0C1
Igfbp2	7.50507	15.5251	4.20474	2.07998	7.65144	7.80674	D0C2
Irs1	28.3658	18.9291	18.2978	11.4276	28.0479	27.5989	D0C2
Itga11	107.951	84.4679	57.6005	49.038	89.4107	100.857	D0C2
Itgbl1	6.99364	5.99785	10.8657	8.99488	8.78173	7.00141	D0C4
Kpna2	85.0889	73.8415	35.1353	29.1391	57.7453	54.8105	D0C2

Krt19	2.28526	3.18724	7.04168	9.48376	3.55878	3.53051	D0C4
Lce1g	9.61433	11.896	18.3728	21.6468	10.278	14.6473	D0C4
Lce1h	5.75192	9.8583	19.6049	20.2604	7.35046	10.429	D0C4
Lgi2	1.00377	0.478793	0.218888	0.24686	0.504488	0.743171	D0C2
Lifr	18.947	18.2322	9.52294	12.2724	20.0675	28.6473	D0C2
Lipo3	17.0196	13.5876	9.41812	5.74294	18.693	15.6507	D0C2
Lrp4	24.909	18.42	10.9635	8.6466	18.6941	19.1675	D0C2
Lrrc3	0.657953	0.473361	1.48167	1.1775	0.779018	0.861204	D0C4
Ltbp1	24.6979	23.2926	11.0233	14.6698	40.2237	39.5003	D0C2
Mcam	6.23142	7.7515	11.4391	11.8361	8.18643	8.19415	D0C4
Mcm2	27.9336	26.6	19.5215	17.8752	25.122	24.8415	D0C2
Mfng	4.69448	4.14864	8.57764	9.78369	5.65286	6.83444	D0C4
Mfsd13a	7.9559	8.34516	3.37414	4.67736	7.84566	8.96454	D0C2
Mgll	37.6663	28.5308	8.61263	8.49369	13.5388	19.1144	D0C2
Miga1	17.7655	16.1495	8.6949	7.69529	15.2312	15.9262	D0C2
Morn4	7.05835	6.07844	10.2993	9.92819	6.90641	7.17469	D0C4
Mrc1	130.93	103.521	96.4847	65.2229	127.113	112.084	D0C2
Ms4a7	245.687	224.179	292.147	309.163	237.524	245.836	D0C4
Mthfd2	41.7347	37.9772	26.068	25.806	20.9612	21.4004	D0C3
Nes	19.3166	20.4923	55.5245	39.9958	48.0802	36.2788	D0C4
Neurl1a	2.78252	2.67138	5.00409	5.1656	2.42666	2.48524	D0C4

Npy	21.1901	21.9346	51.7795	55.456	38.8335	31.3228	D0C4
Nr4a1	10.9767	12.885	48.3694	46.4084	30.502	26.9072	D0C4
Ogn	68.3523	51.3586	152.953	124.262	279.613	216.429	D0C1
Olfr1033	4339.71	3563.71	1996.9	1046.28	2521.18	2765.4	D0C2
Olfr1309	0	0.140844	1.12697	0.612859	0.118089	0.179172	D0C4
Parvg	2.64057	3.23414	15.8583	15.1658	2.36271	3.07077	D0C4
Pcnx	6.5952	5.1154	3.88618	3.07872	6.66593	6.46977	D0C2
Peak1	37.6322	35.9202	50.4502	40.1656	41.7447	38.4109	D0C4
Peg3	0.827064	0.709559	2.77808	1.56927	4.46173	3.79859	D0C1
Pgm5	4.57222	1.74302	0.350634	0.557991	1.13073	1.25215	D0C2
Phlda1	46.029	55.2153	71.9597	70.557	57.6974	57.2193	D0C4
Pi15	3.36108	1.24695	6.55371	4.63692	13.8906	16.5313	D0C1
Pira2	1.83371	2.11705	3.94845	4.52603	7.19949	8.91171	D0C1
Plagl1	5.21108	3.69469	11.1714	9.0804	16.8642	16.3034	D0C1
Pld4	53.0794	52.4198	84.6035	88.7098	76.5408	73.5192	D0C4
Plin2	175.202	186.146	246.304	252.285	206.568	221.333	D0C4
Polg	10.1912	10.5936	8.14481	5.14127	11.5093	10.8237	D0C2
Postn	362.49	203.786	50.925	60.6627	159.879	281.85	D0C2
Prg4	1.05472	0.268924	2.27E-07	0	0.557983	0.654003	D0C2
Prl2c2	59.3366	62.5904	124.728	124.333	92.0865	102.458	D0C4
Prnp	102.355	91.4338	70.3371	66.157	99.7187	101.761	D0C2

Ptn	13.7042	11.7805	5.7801	4.89448	20.907	19.4469	D0C2
Pyroxd2	2.93887	2.74592	6.59995	4.84647	3.61327	2.69196	D0C4
Qsox1	178.531	147.301	113.453	104.259	160.249	159.684	D0C2
Rab6b	3.21248	2.50123	6.55671	5.43365	4.36951	3.63465	D0C4
Rbms1	62.2758	53.879	71.5368	70.5587	69.0673	61.4658	D0C4
Rgs16	30.3616	36.1256	51.7943	49.7475	35.2894	32.8133	D0C4
Rhoc	219.634	186.087	280.369	242.137	235.895	182.098	D0C4
Rnaset2b	121.186	119.381	158.832	149.769	29.3971	25.8436	D0C4
Rpl3-ps1	208.395	227.896	0	0.354726	177.795	195.979	D0C2
Rpl34-ps1	122.649	179.675	425.189	650.162	258.331	281.195	D0C4
Rplp0	1191.02	1297.46	14.5346	22.6419	1224.01	1319.36	D0C2
Rsl1	1.69742	1.71934	4.10268	3.2713	2.16433	2.20573	D0C4
Rspo2	15.3582	14.7092	39.6928	34.8081	76.8048	62.035	D0C1
Satb2	17.306	7.71878	6.64014	3.92085	10.7307	12.9452	D0C2
Sema7a	80.3479	67.4795	59.3305	41.4366	41.3272	36.6956	D0C3
Sfxn2	5.21874	4.98784	2.20178	1.99234	4.66411	4.104	D0C2
Slc20a1	67.6293	58.5973	38.6475	41.4441	32.4197	33.1734	D0C3
Slc4a4	1.9366	1.38359	3.47837	2.35634	2.54957	1.89323	D0C4
Slfn8	2.53426	2.0524	0.541082	0.02971	6.68808	5.8652	D0C2
Slit3	32.6871	20.3792	18.022	10.9309	25.7955	21.5926	D0C2
Snapc1	3.21126	2.59799	6.26538	5.72201	4.70594	4.22275	D0C4

Socs3	36.4881	37.4276	25.0713	23.4806	37.9639	35.1842	D0C2
St5	13.5185	11.6076	8.63011	6.27435	11.8243	14.1637	D0C2
St8sia2	1.31829	0.982743	0.284849	0.269249	0.76171	0.812825	D0C2
Stac2	1.51888	1.60827	4.94273	3.83926	2.04167	3.0518	D0C4
Stxbp2	27.9447	28.0503	44.7668	40.7118	33.8125	34.2437	D0C4
Susd5	3.09352	2.37226	0.21794	0.389103	0.777616	0.836879	D0C2
Sycp1	0.799129	0.315561	0	0	1.64509	0.97178	D0C2
Thbs2	243.67	142.956	97.9467	102.054	190.589	231.695	D0C2
Tmem100	7.67195	6.16107	20.3959	17.2284	13.1178	11.9764	D0C4
Tmem200c	1.09946	0.982485	0.565689	0.262916	1.21057	1.04615	D0C2
Tnc	285.921	120.703	91.8305	44.5156	147.645	214.569	D0C2
Tnn	136.266	85.5618	13.5711	14.0145	25.5273	42.7646	D0C2
Tpm3-rs7	147.245	120.424	79.0945	88.9145	0.457167	0.23837	D0C3
Trib2	6.99459	7.3339	12.9495	12.9376	10.8605	11.168	D0C4
Trim12a	7.70685	7.23855	14.7867	13.8356	11.1068	9.28211	D0C4
Trim30d	2.50985	2.30718	7.61936	7.47028	5.43628	5.37377	D0C4
Trim34b	1.8252	1.35629	0.141316	0	2.02638	2.11456	D0C2
Tubb2b	7.19761	7.97204	13.7386	14.8991	6.86337	6.71579	D0C4
Unc5d	0.341983	0.286995	1.41604	0.779414	0.510954	0.352355	D0C4
Vps13d	4.68348	6.94406	10.4522	13.2672	6.69863	6.16117	D0C4
Wfdc17	146.847	183.489	48.4466	58.8858	401.857	439.397	D0C2

Zfp365	13.2592	10.8833	10.0735	5.63078	14.2345	12.5283	D0C2
Zfp729a	29.4243	28.6564	13.4687	12.4637	24.1481	25.5423	D0C2
Zfp991	10.4764	6.42484	17.5586	13.5246	12.5507	11.0698	D0C4
Zfp992	5.78496	3.78395	10.2929	6.98457	7.40576	5.95797	D0C4
Znf41-ps	5.88692	3.89364	11.1833	7.49251	8.00504	6.10714	D0C4

Supplementary Table 6 Significantly changed genes in BMSCs from wildtype, Runx2+/- and Runx2+/-;Mir338-/- mouse femur after osteoblastic induction

ext_gene	Runx2_wt_D9_1_tpm	Runx2_wt_D9_2_tpm	Runx2_het_D9_1_tpm	Runx2_het_D9_2_tpm	double_mutant_D9_1_tpm	double_mutant_D9_2_tpm	cluster
Igf2	8.64322	8.93574	35.6739	29.2287	90.1863	62.3296	D9C3
Gna12	72.5851	88.4858	105.51	108.333	65.9915	69.1681	D9C4
Rpa1	29.2087	32.743	46.4749	41.8126	34.8884	34.6355	D9C4
Mmp11	8.48782	8.9373	14.0916	14.225	21.0378	20.9671	D9C3
Cfp	153.654	185.537	227.164	243.379	193.474	196.009	D9C4
Uhrf1	9.56396	13.8592	18.5833	15.6568	12.3557	9.0132	D9C4
Cyp51	31.1447	34.6501	24.3011	23.5498	19.2589	19.4427	D9C1
Tubb6	84.6417	99.2169	118.491	128.323	89.4631	87.8054	D9C4
Trmt1	0.131807	0	0.774966	0.885497	13.0152	8.69337	D9C3
Ltbp2	31.5886	39.0098	77.1441	59.0273	98.3817	91.0826	D9C3
Sfrp4	36.8601	32.0017	94.0557	94.8822	87.0293	68.8815	D9C4
Chaf1a	7.04941	7.70671	12.7113	9.7259	7.43507	6.9288	D9C4
Apoc2	37.2014	42.1064	70.0905	73.1772	49.0222	42.3382	D9C4
Phyhip	1.35995	1.24162	0.051767	0	1.27086	1.53469	D9C2
Tpm4	602.785	615.109	811.378	702.912	718.556	650.901	D9C4
Comp	13.3653	11.1501	8.71043	7.5623	17.5572	17.0238	D9C2
Nqo1	248.573	256.115	488.298	482.898	289.173	285.243	D9C4
Mmd	54.6802	60.5519	86.4386	77.3938	63.6163	56.9695	D9C4
Dzip1	10.2379	9.09152	13.9031	10.354	18.4544	15.8375	D9C3

Col5a3	15.0279	12.8213	8.14912	7.42065	4.74735	5.38649	D9 C1
Angptl 2	182.805	156.454	130.704	122.3	111.057	111.055	D9 C1
Adh1	2.15023	2.15506	5.65466	6.49042	16.6136	13.525	D9 C3
Ptpn6	5.62464	6.92177	9.82727	10.695	8.10436	5.47469	D9 C4
Aqp1	29.051	19.9657	40.9056	49.339	25.6472	29.7232	D9 C4
Crabp2	12.6352	22.545	35.0939	45.1101	16.1357	16.7018	D9 C4
Asf1b	10.4637	14.1413	19.404	16.4137	13.5136	11.7741	D9 C4
Mmp1 2	0.076102	0	0.269084	0.436758	99.7811	86.0825	D9 C3
Cdt1	15.3225	17.4475	23.4458	22.9931	18.0919	17.996	D9 C4
Chrd	1.54949	1.05659	2.55844	2.17512	0.334739	0.437425	D9 C4
Hapln4	8.7429	5.03644	1.86749	1.70441	0.611782	0.791741	D9 C1
Rpl30	72.0417	87.1165	144.666	170.676	51.4474	56.4446	D9 C4
Serpin b2	339.368	152.899	48.7774	29.5135	13.782	13.8384	D9 C1
Ubp1	9.71E-06	0	0.537194	1.32298	0	0	D9 C4
Panx3	55.1636	33.686	8.25459	10.8334	2.96645	3.89539	D9 C1
Gltp	173.518	178.256	125.598	117.632	205.554	201.025	D9 C3
Zfp729 a	31.0317	22.5701	12.936	10.4831	19.9095	18.3527	D9 C2
Fgfrl1	16.5324	19.4078	30.4986	26.0582	55.008	48.4035	D9 C3
Clec4f	3.69094	2.90234	4.83348	4.78001	1.42524	1.71105	D9 C4
Pcolce 2	12.0149	8.87499	5.46662	6.30203	3.98392	4.08524	D9 C1
Gzmc	21.042	16.2234	4.74689	6.35075	2.9436	3.50533	D9 C1

Lbp	2.67772	3.47357	8.40187	6.74451	15.4298	13.0342	D9 C3
Ttll12	18.6649	18.0103	21.887	22.927	16.6081	17.8794	D9 C4
Stac2	1.10474	0.830044	2.05559	1.9654	1.22418	1.36051	D9 C4
Igfbp4	196.51	283.264	383.507	428.383	14.4999	9.62192	D9 C4
Mfng	6.28598	7.80496	10.8383	10.692	7.50821	6.59613	D9 C4
Kpna2	72.9034	77.8373	42.6491	36.8554	66.5042	64.4267	D9 C2
Dhx40	27.8967	23.9489	17.031	13.1529	27.8163	26.5772	D9 C2
Ypel2	7.70114	8.01238	5.65363	5.37808	10.7508	11.1296	D9 C3
Sparc	3667.65	2477.83	1452.29	1775.67	757.473	649.269	D9 C1
Ccl6	207.888	239.539	321.084	279.194	54.3294	38.4866	D9 C4
Isyna1	40.5938	51.0232	61.7193	66.5055	55.024	51.9541	D9 C4
Dpep1	21.8764	25.0218	42.3686	33.5809	90.049	77.2276	D9 C3
Gdpd2	0.365839	1.84666	6.17676	5.18276	19.0053	10.0752	D9 C3
Gle1	14.6291	13.4843	8.13805	7.84508	13.2913	11.0556	D9 C2
Calr3	1.90345	1.36597	3.27017	2.84255	1.29543	1.65018	D9 C4
Ntn4	5.97078	5.97562	10.9794	9.20547	8.04268	8.34512	D9 C4
Timp3	865.575	869.025	1197.49	1002.06	673.462	610.891	D9 C4
Hif1a	868.3975	969.8875	214.1296	204.4767	862.1766	867.3106	D9 C2
Prmt2	13.0327	12.3132	19.8281	22.5097	2.97343	4.55545	D9 C4
Btg2	44.8605	44.1556	67.1241	56.6982	103.633	92.5933	D9 C3
Igfbp3	6.78309	9.64611	24.3609	25.2616	65.3522	46.0036	D9 C3

Efemp 1	2.48349	5.39973	22.4075	16.8779	47.9112	39.9448	D9 C3
Pctp	20.5445	21.3951	14.3994	13.9142	21.5901	20.475	D9 C2
Klhl29	0.176625	0.172312	0.750011	0.814171	1.62291	1.47188	D9 C3
Rsad2	1.36132	1.1361	1.88397	1.96712	1.40442	0.92695	D9 C4
Id2	123.052	122.049	201.139	179.64	179.803	167.556	D9 C4
Rrm2	43.0602	49.1914	71.2085	61.6982	46.8247	44.9219	D9 C4
Adcy3	20.0285	17.1596	28.7749	26.2176	21.789	19.7828	D9 C4
Slc13a 5	61.8861	45.4468	7.15123	12.2344	2.24831	3.14689	D9 C1
Rflnb	20.9129	18.6677	10.9024	11.9017	22.8447	23.9475	D9 C3
Pole2	6.79707	6.94425	10.8317	8.95523	6.1539	6.41382	D9 C4
Smoc1	2.67537	3.46187	7.54157	7.01327	15.6967	13.6356	D9 C3
Akr1c1 8	17.1288	17.2072	93.3741	83.6588	60.4033	53.2268	D9 C4
Fos	52.741	49.0962	92.6655	75.8172	129.434	111.964	D9 C3
Id4	5.29283	6.3543	15.1187	11.8063	27.2996	28.894	D9 C3
Aspn	22.3477	22.1955	33.1834	30.7005	65.7415	65.0956	D9 C3
Ogn	142.744	125.929	290.824	209.997	501.61	405.729	D9 C3
Rsl1	1.80734	1.81948	3.52884	3.06599	1.98595	1.9749	D9 C4
Nkd2	35.3308	38.2948	74.1694	72.6488	55.7729	55.9871	D9 C4
Hmgcr	55.9663	48.8572	35.2912	32.0643	22.659	24.1555	D9 C1
Fgf10	0.339315	0.389106	2.52736	1.76997	3.69696	4.53746	D9 C3
Plau	22.873	25.7299	36.4066	36.3926	26.2384	27.7425	D9 C4

Tmem 254b	41.6161	47.4117	28.9431	31.1956	52.8267	50.5398	D9 C3
Gm676 8	25.6755	19.641	0	0	23.3417	20.5599	D9 C2
Epsti1	16.725	17.5297	22.5442	19.973	15.8297	13.8401	D9 C4
Rgcc	32.6576	33.9555	20.2455	19.8187	41.3254	42.2984	D9 C3
Clu	1.77284	2.31909	8.47775	7.63798	16.3715	16.8946	D9 C3
Loxl2	195.017	176.034	134.939	116.327	109.038	108.073	D9 C1
Bmp1	215.298	168.96	152.922	136.624	132.144	123.388	D9 C1
Gfra2	1.94807	2.28708	0.437367	0.39249	1.44686	1.13341	D9 C2
Haus4	11.2571	13.8924	20.0633	18.0119	16.1101	13.7216	D9 C4
Psmb5	237.147	244.515	300.276	342.665	191.139	219.859	D9 C4
Mtdh	101.631	92.8457	68.2932	47.657	117.855	99.9265	D9 C2
Tbc1d3 1	6.11348	7.58253	9.9419	8.21334	7.15845	7.07005	D9 C4
Parvg	5.01374	4.04462	12.2782	12.2574	3.32708	2.71715	D9 C4
Cldn1	0.051565	0.12566	0.691763	0.279232	2.33032	2.59882	D9 C3
Mcm4	17.7517	21.5755	28.4518	22.9099	22.5782	20.5321	D9 C4
Klhl24	27.3383	23.6237	20.3366	13.0412	31.6227	27.6415	D9 C2
Mylk	26.6848	31.8267	40.1273	33.2508	54.2938	55.0697	D9 C3
Adipoq	3.71377	6.12559	21.7259	16.6537	76.9951	74.0832	D9 C3
Rfc4	7.82859	6.37687	10.9174	10.0423	7.81393	7.36303	D9 C4
Adamt s5	10.3525	9.98043	17.3581	10.7909	40.3581	30.7412	D9 C3
Cxcl13	0.176441	0.429119	4.49137	1.56307	6.75363	11.152	D9 C3

Serping1	99.5846	99.4014	130.428	130.95	195.81	196.709	D9C3
Treml1	14.2764	9.59732	6.51135	5.36676	22.9692	20.6678	D9C2
Ehd3	54.1812	47.469	34.954	28.7831	49.9909	48.6336	D9C2
Epas1	28.2978	37.4024	54.1974	46.753	99.1369	88.9174	D9C3
Mcfd2	156.922	147.177	193.006	186.247	124.778	128.735	D9C4
C3	1.67333	1.24097	2.92987	2.35605	7.18255	8.27394	D9C3
Rps10	414.634	457.322	312.253	249.237	2.60089	4.34659	D9C1
Dynap	34.2698	22.2047	1.74832	1.93565	0.634235	0.30667	D9C1
Me2	45.0058	38.5195	29.9854	27.4491	25.3864	23.9974	D9C1
Lmnb1	11.5829	13.9176	17.1272	14.3786	12.4801	11.6057	D9C4
Psat1	13.2439	15.11	21.5058	22.8324	14.664	14.4424	D9C4
Ms4a6d	293.875	330.913	244.16	212.377	11.9563	6.9402	D9C1
Fam111a	44.2687	47.0331	58.4827	47.7437	48.7064	42.3547	D9C4
Aldh1a7	0.25722	0.313244	1.48296	1.12628	4.28577	3.96252	D9C3
Lipo3	15.3851	20.0491	12.7151	10.5825	19.9682	19.5644	D9C2
Jak2	7.82E-09	1.77E-08	3.71723	2.13845	0.124975	0.038854	D9C4
Hells	3.742	4.06406	6.73299	4.48538	4.29994	3.32579	D9C4
Sfxn2	6.02388	4.96974	2.53607	2.31268	5.04187	5.05771	D9C2
Got1	31.729	33.8281	42.7921	43.8372	28.2042	34.432	D9C4
Scd2	109.984	120.825	87.9872	79.4123	69.9712	69.4144	D9C1
Prim1	6.05143	5.28897	9.5167	9.96276	6.19369	7.1979	D9C4

Irf7	7.10784	7.7268	18.4675	18.7412	10.3968	10.3446	D9 C4
Itih5	0.193579	0.188841	1.24252	0.833757	3.39745	2.73704	D9 C3
St8sia2	2.81006	1.9658	0.31925	0.290813	1.05419	1.38157	D9 C2
Ccr1	17.8321	16.8688	5.31649	3.9171	3.2039	3.15695	D9 C1
Casp4	4.65576	4.94804	8.00782	8.35974	6.46486	5.31429	D9 C4
15000 15O10 Rik	4.69787	5.70508	10.756	11.6836	20.3379	26.7453	D9 C3
Igfbp5	10.1998	10.6213	68.2508	51.2904	144.639	131.477	D9 C3
Ngef	18.7732	13.0308	2.47301	3.2046	0.997851	1.66949	D9 C1
Gpr39	0.453873	1.12316	2.2557	2.68923	0.851233	1.07517	D9 C4
Mcm6	30.372	41.2318	52.5819	47.4348	36.7154	33.9886	D9 C4
Cd55	4.24717	4.3456	8.90488	5.25888	14.6845	11.8634	D9 C3
Rgs16	14.6096	18.1723	25.4365	25.2016	23.0719	19.464	D9 C4
Mcm1 0	2.7344	3.20765	5.06612	3.94514	3.13627	2.92678	D9 C4
Ccdc3	0.512278	0.520059	2.16085	2.30437	7.48588	9.02842	D9 C3
Nuf2	7.06543	9.31094	11.9179	10.6842	8.09554	8.19796	D9 C4
Mrc1	184.676	176.462	143.119	104.685	126.421	99.727	D9 C1
Nebl	3.61694	2.66875	0.942171	0.819768	0	0	D9 C1
Itga8	1.52009	1.78923	3.57055	2.04777	9.39982	6.56638	D9 C3
Fcna	20.2043	24.6062	32.6548	34.5439	22.995	21.4782	D9 C4
Galnt3	15.0838	8.75284	4.29408	4.08884	2.9674	3.09763	D9 C1
Hat1	24.1748	29.4774	34.545	32.175	27.6706	27.0863	D9 C4

Syt13	9.93497	8.97964	15.116	14.0212	9.86901	10.2365	D9 C4
Pkia	2.01697	2.06682	4.06728	3.12125	5.97556	5.76279	D9 C3
Stmn2	2.37958	2.42065	4.61221	5.04849	1.76832	1.1647	D9 C4
Zbp1	0.781493	0.706982	3.86136	1.70157	5.27565	2.96941	D9 C4
Procr	20.922	20.9498	29.0079	30.5205	23.3332	21.3278	D9 C4
Tm4sf 1	51.3595	56.6571	71.9023	83.4733	44.1987	43.5115	D9 C4
Sycp1	0.484571	0.354872	0	0	1.24349	0.918153	D9 C2
Vcam1	15.1	16.4266	30.4882	24.212	86.7388	70.2174	D9 C3
Sfrp2	4.66951	5.37574	9.48744	9.30515	21.2648	31.4493	D9 C3
Pdgfc	42.5784	43.4125	28.2274	24.8524	15.7349	11.2554	D9 C1
Enpep	0	0.041283	1.08618	0.384216	2.1765	1.78302	D9 C3
Ptgfr	2.92076	3.36415	4.57623	4.09563	13.7829	11.8231	D9 C3
Gatb	2.28867	2.6107	4.70433	4.02046	8.03125	7.77232	D9 C3
Dapp1	9.78968	10.1748	6.96245	5.88962	10.1328	10.0832	D9 C2
Trp53i np1	36.096	31.4002	27.1029	22.8354	51.7031	41.2013	D9 C2
Gbp2b	0.511495	0.513187	3.72316	2.74577	1.9613	1.18567	D9 C4
Epb41l 4b	1.16318	1.61014	3.58939	2.89617	9.3108	6.78915	D9 C3
Pdpn	27.3451	22.7414	15.811	17.2866	11.7419	11.8159	D9 C1
Capzb	2.54329	2.94401	7.58526	5.38357	3.05104	2.93949	D9 C4
Hgf	0	0.151987	0.579107	1.66714	5.38265	3.58729	D9 C3
Angptl 7	8.80357	10.5608	0.888804	1.01424	8.63972	8.80254	D9 C2

Nsg1	38.2098	35.9765	51.7298	52.0394	1.60219	0.885306	D9 C4
Tgfbr3	14.5896	16.4978	21.3194	16.0736	44.9002	40.7976	D9 C3
Ibsp	6490.87	3006.25	670.361	1254.93	404.086	477.046	D9 C1
Ereg	2.6901	4.16573	7.4215	5.01387	4.95671	3.97672	D9 C4
Anxa3	202.819	228.62	325.55	304.27	239.616	240.454	D9 C4
Col1a2	5012.6	3255.02	1513.23	1758.9	51.7521	33.2683	D9 C1
Gpnm b	415.974	385.723	666.368	548.605	62.7112	37.7363	D9 C4
Frmd4 b	20.6519	20.2423	16.6883	9.96345	8.87828	7.82831	D9 C1
Bhlhe4 0	46.4413	33.6793	26.8378	27.1364	18.2031	20.4043	D9 C1
Usp18	2.50089	2.62703	5.00945	4.35567	2.25799	1.94017	D9 C4
Clec2d	12.0889	15.937	22.0046	21.6747	34.2648	33.3074	D9 C3
Dmpk	126.253	151.806	217.661	211.922	14.3944	12.4215	D9 C4
Zscan2 6	2.76166	2.34996	4.79838	4.20568	21.0972	16.9907	D9 C3
Il4ra	34.3921	31.0496	24.8967	21.5585	39.4132	41.5468	D9 C2
Nsmce 1	32.026	33.4436	41.8302	43.1539	31.6101	36.2296	D9 C4
Ebf3	3.28318	1.94828	6.02605	5.44154	12.4731	10.2309	D9 C3
Vegfc	2.54058	3.49584	7.93683	6.27428	23.2232	19.1013	D9 C3
Dlc1	16.7232	12.9071	12.5538	9.47665	18.0063	16.0246	D9 C2
Dusp4	16.9521	13.2123	22.2046	18.4494	16.4102	15.6043	D9 C4
Hpgd	385.8	404.662	524.031	451.511	401.013	349.596	D9 C4
Orc6	13.9209	12.3909	17.6808	17.5753	13.4799	12.1958	D9 C4

Clmp	9.08436	6.97646	14.0457	11.3411	21.2278	19.0125	D9 C3
Aldh1a 2	12.7709	21.0841	108.83	91.5827	62.1467	67.1213	D9 C4
Itga11	154.202	139.644	82.9412	69.306	109.772	113.386	D9 C2
Gsta4	122.949	116.199	189.415	196.592	141.31	151.935	D9 C4
Tbx18	6.79998	4.54707	3.68441	2.59588	5.10577	4.53201	D9 C2
Rab6b	1.48805	1.23542	3.35931	3.05084	1.87749	1.66524	D9 C4
Lama1	0.413641	0.524463	1.23261	0.909856	2.14915	1.85025	D9 C3
Ppp1r9 a	2.00805	1.59447	1.28316	0.970504	2.42712	1.77731	D9 C2
Rassf4	38.0404	37.01	30.7946	22.705	37.2361	34.0882	D9 C2
Mamd c2	17.4348	7.41669	2.74965	3.27046	2.13042	1.72518	D9 C1
Prss35	12.96	11.4656	4.54564	5.58959	3.41782	1.24302	D9 C1
Wipf2	7.28109	8.26612	6.14252	5.182	4.19151	4.07067	D9 C1
F13a1	544.944	567.383	242.096	180.273	177.395	170.702	D9 C1
Hgsnat	262.499	288.908	477.412	463.614	136.776	129.964	D9 C4
Cbl	0.393691	0.211655	0	0.04493	0.710276	0.181085	D9 C2
Cd207	99.8459	98.1871	224.039	208.158	144.401	145.955	D9 C4
Slfn8	2.82379	2.7265	0.522968	0.244379	6.88019	4.56612	D9 C2
Esm1	4.5508	8.62107	36.66	28.8817	120.257	88.623	D9 C3
Lum	28.9124	19.7807	48.04	54.3385	30.3782	37.4766	D9 C4
Id1	173.117	175.639	230.669	245.602	191.069	202.966	D9 C4
Spry1	16.346	17.0151	25.8331	20.2083	41.5668	36.9509	D9 C3

Fam124a	3.80886	3.23462	1.38785	1.08697	4.77609	4.08112	D9 C2
Thyn1	15.6226	15.8214	29.622	31.9652	32.9256	28.8874	D9 C4
1810011H11Rik	22.0387	24.8657	32.1946	32.9685	28.6078	26.7139	D9 C4
Trim13	6.27583	4.66999	8.18621	7.77776	5.45613	5.43397	D9 C4
Gabrb3	6.2675	4.36537	1.94239	1.79828	1.18877	0.865156	D9 C1
Nup43	7.35257	7.50442	11.6782	9.76532	6.37074	7.20205	D9 C4
Cd59a	40.9122	38.5033	19.2278	17.389	27.4508	25.9655	D9 C2
Irs2	8.24761	7.68932	5.97075	4.77972	8.44138	8.2374	D9 C2
Maob	2.62188	3.03324	5.43241	4.72017	12.7374	12.3828	D9 C3
Fam110c	0.19071	0.177039	1.15535	0.88511	2.22574	1.94086	D9 C3
Srxn1	135.003	126.725	161.007	161.331	141.487	138.259	D9 C4
Scara3	15.8411	16.4282	22.6946	23.8181	34.7072	33.1706	D9 C3
Inhba	101.277	81.4714	70.3044	47.0628	51.8018	42.1266	D9 C1
Arl5c	4.10938	3.4605	7.9385	6.08861	4.27133	2.55911	D9 C4
Six4	2.97693	2.13886	1.46838	1.44136	2.5901	2.3088	D9 C2
Asb4	21.1209	20.8691	15.0789	14.2795	11.945	10.6174	D9 C1
Serpina3g	0.07851	0.204109	1.17636	0.766127	5.86554	5.93762	D9 C3
Lrrn3	0.511985	0.814571	1.79137	1.56825	4.72734	3.6219	D9 C3
Abca6	0.954962	0.879786	0.547691	0.24086	1.84835	0.850919	D9 C2
Bag2	17.8982	16.4197	24.5183	23.314	21.4166	17.4672	D9 C4
Lgr6	7.37279	6.42784	10.4278	10.3854	4.72634	5.99227	D9 C4

Oas3	7.33399	9.39954	14.4178	12.8003	7.91324	7.37077	D9 C4
Hist1h 1d	1.45323	0.979018	0.727765	0.482426	1.13434	1.09079	D9 C2
Adk	17.0393	12.7053	28.4254	19.1052	17.9873	15.9418	D9 C4
Fmo1	1.65444	1.87248	4.13686	4.78293	29.8602	20.4994	D9 C3
Spon2	1.04115	0.80608	2.97734	2.64634	6.1094	5.19293	D9 C3
Kcnk1	17.508	10.6777	2.58406	3.18785	1.32745	0.938305	D9 C1
Olfml2 b	59.761	49.9553	36.8794	34.4335	56.922	56.1243	D9 C2
Adam2 2	1.56821	1.05902	0	0.174113	2.63243	3.50189	D9 C3
Adgrl4	0.599625	0.832441	0.207652	0.185706	0.868279	0.490409	D9 C2
Plekhh 2	5.59313	5.75495	8.77923	5.91354	16.0804	13.807	D9 C3
Igfbp2	11.1045	12.4772	2.83726	6.04607	23.0582	16.2847	D9 C2
Arrdc4	35.8606	37.0215	62.9807	49.9012	108.946	95.5293	D9 C3
Egr2	2.57075	3.37754	5.47154	3.78846	3.12004	3.05604	D9 C4
Tsc22d 1	13.1859	10.9391	9.02755	7.77979	4.47439	3.74905	D9 C1
Clspn	3.22375	3.83226	6.1226	4.5549	4.09776	2.61818	D9 C4
Abi3bp	0.216169	0.309401	2.24133	0.960998	9.13079	5.97723	D9 C3
Pold1	8.11645	11.4931	15.1626	13.1656	11.3227	10.3528	D9 C4
Relb	3.14611	0.832972	0.000981	0.151584	1.00451	2.67106	D9 C2
Nov	1.21219	2.26312	5.23065	6.19923	24.1809	15.2163	D9 C3
Nudt1	4.84849	5.72142	8.78407	9.67734	1.54799	2.43064	D9 C4
Vsig4	93.8951	106.46	159.746	151.215	114.628	100.075	D9 C4

C5ar1	66.4243	54.1665	93.8089	143.923	62.0559	44.8684	D9 C4
Zc2hc1 a	20.9866	20.8363	32.1403	22.6142	50.8254	39.6969	D9 C3
Peg3	1.08013	0.913992	3.63425	2.39859	7.43947	4.71565	D9 C3
Adgrg3	0.533351	0.837038	0.164973	0	1.31977	0.993565	D9 C2
Synpo 2	2.21873	1.61923	1.10565	0.685547	1.81671	1.61398	D9 C2
Glp2r	0.233055	0.162316	0.518919	0.476778	0.173584	0.222382	D9 C4
Morn4	3.6901	3.84521	7.80282	7.77976	5.67941	5.55292	D9 C4
Pram1	7.14149	9.03766	20.1048	13.4329	12.2448	9.71648	D9 C4
Fam69 c	0.131152	0.099561	0.731694	0.675808	1.90804	1.89128	D9 C3
Rbpms	3.91999	4.50316	8.22915	6.63776	11.5808	10.8276	D9 C3
Mcm3	24.389	27.7117	38.8741	35.7593	23.3064	23.9781	D9 C4
Zfp748	0.578182	0.885423	3.77876	2.78614	0.244229	0.563579	D9 C4
Lingo3	1.67071	0.944146	0.398211	0.393399	0.063128	0.091757	D9 C1
Pcsk6	15.0649	8.81395	1.99616	4.16921	2.41896	1.04093	D9 C1
Tsc22d 3	128.339	142.574	91.9876	83.0775	127.415	139.027	D9 C2
Fads6	5.3198	3.68136	3.36801	2.4708	4.9963	4.51644	D9 C2
Adgrd1	10.3563	5.90851	3.40825	3.59987	2.05541	1.67417	D9 C1
Npas2	1.33377	1.09937	0.451863	0.299204	0.907075	0.776546	D9 C2
Kcne4	0.57947	0.261453	1.00277	1.12021	2.29444	2.56151	D9 C3
Mcm2	16.1843	18.5205	25.0574	23.223	15.5251	14.0234	D9 C4
Cd24a	56.7061	33.4911	23.1141	20.5373	18.9826	13.1079	D9 C1

E2f8	2.03281	1.50188	3.94436	3.29593	1.85804	2.15241	D9 C4
Fzd4	0.439168	0.733093	1.3571	1.13966	4.24942	3.95018	D9 C3
Klhl6	29.1648	35.8745	19.3999	16.1055	30.3134	28.1332	D9 C2
Basp1	77.9102	82.1655	97.2744	95.3623	79.8456	76.5706	D9 C4
Abi3	18.0133	15.2993	11.3429	9.89271	6.09138	7.81875	D9 C1
F2r	9.53658	11.1425	28.0995	19.5968	50.946	47.0657	D9 C3
Treml4	76.7808	75.2663	56.8957	50.8636	5.45384	6.10885	D9 C1
1700012B09Rik	3.43919	3.19408	10.7895	11.528	3.62376	4.99617	D9 C4
Nod1	9.17237	9.23895	13.0976	10.1898	10.7272	8.9203	D9 C4
Ccbe1	2.22367	1.87047	3.33596	2.45399	5.2262	5.87433	D9 C3
Hacd2	36.7117	32.8906	13.0104	13.4993	19.2625	18.3696	D9 C2
Itga1	3.7479	4.22745	5.88644	4.414	9.34258	8.42842	D9 C3
Cd14	45.7902	45.5307	56.84	55.8529	81.6441	89.8464	D9 C3
Mab21l3	0.746659	0.651441	0	0.068996	0.386729	1.08018	D9 C2
Pomk	36.1422	34.9357	44.8919	42.7375	19.1023	18.6623	D9 C4
Ptgs1	26.3929	29.55	39.8149	37.1643	33.6658	34.2512	D9 C4
Emx2	2.97328	1.8729	1.40657	0.974831	3.67116	3.20172	D9 C2
Foxc1	10.0711	9.6578	17.2174	16.2861	27.1004	27.4031	D9 C3
Phf11a	0.671246	0.347485	1.73651	1.37661	0.434673	0.540637	D9 C4
Chil3	10.8964	12.7928	5.68049	3.71142	9.47503	8.11415	D9 C2
Agt	13.1683	8.14241	23.1405	22.3636	53.3974	48.006	D9 C3

Rspo2	20.9829	22.2912	38.7374	32.9327	93.5588	93.7982	D9 C3
Gria4	0.18088	0.240857	0	0	0.243895	0.285658	D9 C2
Tagap1	9.59694	9.31265	13.6118	11.2646	6.13232	5.15919	D9 C4
Klf13	36.5941	39.5104	26.4866	22.6378	34.4662	36.2024	D9 C2
Fzd5	25.5219	18.59	16.1798	11.5756	24.5227	24.5514	D9 C2
Plpp3	40.0963	40.7502	113.307	85.7535	207.422	174.595	D9 C3
Cemip	61.7374	45.4166	17.1285	13.8256	38.2614	31.4821	D9 C2
Aatk	0	0	0.183967	0.320494	0	0	D9 C4
Adamts15	2.81461	1.94682	1.53555	1.23657	7.79741	5.05842	D9 C3
Gpr141	1.45556	0.984762	0.419967	0.16892	0.857306	0.690605	D9 C2
Chst1	2.64635	3.12185	7.91766	8.3334	6.26297	6.71078	D9 C4
Nap1l1	35.8748	30.2103	44.9185	41.5577	31.2904	27.7687	D9 C4
Tll1	5.90367	5.17798	11.2791	9.10262	7.5669	7.70133	D9 C4
Car8	4.75556	4.71145	15.8765	13.0053	8.22312	5.89208	D9 C4
Tmem179	0.931932	0.947093	1.91477	1.60268	0.792028	1.3476	D9 C4
Cthrc1	171.865	122.197	48.1756	52.8495	30.7592	38.4907	D9 C1
Ywhae	293.465	289.02	348.265	328.693	295.049	296.611	D9 C4
Adam12	16.7178	11.4718	6.71439	6.23031	10.3707	8.78809	D9 C2
Pcdh7	12.4463	8.23338	14.7866	12.542	1.25663	0.303959	D9 C4
C1ra	19.8997	19.2437	27.1359	22.9266	39.9007	37.2224	D9 C3
Ghr	14.6604	12.8031	18.2104	14.755	75.9814	62.2279	D9 C3

C4b	1.02068	0.658468	2.02238	1.42972	5.71174	6.22909	D9 C3
Irs1	31.2352	24.323	21.4349	14.2987	28.1742	27.7438	D9 C2
St3gal 5	163.165	166.813	224.115	195.318	185.814	164.561	D9 C4
Gm145 48	7.01854	7.49252	0.87504	1.15983	2.54226	2.7617	D9 C2
Trim30 d	2.71734	4.18599	7.04696	6.11606	3.52488	2.83499	D9 C4
Tpst2	2.26657	2.28094	3.79812	6.09982	75.2537	81.3265	D9 C3
Col12a 1	147.094	124.067	114.916	72.9012	148.489	128.661	D9 C2
Tmpo	11.3371	15.4719	20.9072	16.5605	15.0256	15.574	D9 C4
Tpm3-rs7	113.255	109.972	85.0226	72.6597	0.368991	0.278257	D9 C1
Cgnl1	14.8359	12.2636	25.912	19.422	0	0	D9 C4
Abcb8	10.4353	10.6157	13.4014	13.7335	10.2838	10.6841	D9 C4
Cnbd2	10.5016	12.8528	4.11747	4.07657	12.4763	9.84951	D9 C2
Negr1	0.227599	0.290186	1.65826	1.25526	8.00275	5.70043	D9 C3
Bmp4	11.3831	12.4613	20.8221	17.4032	37.1793	34.5695	D9 C3
Meis2	1.4621	1.09839	2.98983	2.25503	0.431811	0.461396	D9 C4
Eya4	2.03116	2.3336	2.72E-05	0.018552	11.6883	8.80156	D9 C3
Gulp1	4.9624	4.95842	8.28605	5.57789	19.1601	16.1539	D9 C3
Chrdl1	1.41486	0.950859	3.72158	2.51572	21.4784	13.3728	D9 C3
11-Sep	46.1273	50.2852	69.2013	64.4132	62.6021	59.5637	D9 C4
Hp	52.8379	73.7063	141.926	121.745	315.125	327.047	D9 C3
Cdh11	140.097	121.916	207.854	161.765	296.606	254.306	D9 C3

Acot7	63.2382	63.3035	86.956	98.4336	65.3947	65.6528	D9 C4
Lrmda	8.06533	7.19786	2.79212	3.4096	7.57246	7.99786	D9 C2
Megf10	3.43391	1.34816	0.768743	0.662757	0.220331	0.312396	D9 C1
Tubb2b	4.99453	5.84798	9.49866	9.08676	7.36974	4.36921	D9 C4
Dock7	0.752829	0.199785	0	0	0.634087	0.574257	D9 C2
Adgre5	13.9941	17.8233	21.3174	20.4713	38.9178	40.4051	D9 C3
Eno1b	58.2822	67.177	36.2133	42.1881	0	0	D9 C1
Tspan7	3.04153	3.738	7.17109	6.18785	11.3963	11.7939	D9 C3
Gm8730	13.0151	14.576	36.087	50.7681	4.27356	4.66458	D9 C4
Atrnl1	6.27194	5.38291	3.19409	2.84624	5.11236	5.15016	D9 C2
Pdia4	129.595	128.734	149.484	145.812	137.453	135.913	D9 C4
Rnpep	99.3498	110.626	127.007	120.748	110.987	111.474	D9 C4
Pop1	3.02766	2.31341	4.58844	3.5472	2.77789	1.67939	D9 C4
St5	15.283	15.1943	9.41037	10.8632	2.80416	3.12845	D9 C1
Fkbp5	120.846	132.912	86.832	63.4506	118.495	113.991	D9 C2
Mid1	6.72948	8.42293	28.2684	28.9136	14.939	13.9915	D9 C4
Ybx1	490.441	476.5	582.272	543.986	507.033	504.635	D9 C4
Tnfrsf11b	25.2634	19.7599	14.8698	13.2196	30.2112	24.7653	D9 C2
Notch2	28.5386	27.5113	23.676	16.2363	27.3877	25.0579	D9 C2
H2-T22	13.4192	13.2901	8.82207	7.79732	1.31205	1.73475	D9 C1
Fip1l1	2.71572	2.91085	5.1046	4.75628	9.89848	9.37084	D9 C3

Dbp	14.7223	13.9659	23.4837	36.5932	5.09866	3.35039	D9 C4
Fcgr2b	20.7387	23.7665	13.5677	15.3037	10.5276	9.65732	D9 C1
Fxn	5.85894	5.40717	8.12468	8.96099	4.87626	5.16342	D9 C4
Rpl3	441.107	525.962	747.806	827.489	509.403	489.306	D9 C4
Dio2	0.276231	0.224472	0.78943	0.791302	1.65616	2.56516	D9 C3
Zfp991	10.4226	8.27938	16.6868	12.6245	13.1956	11.2432	D9 C4
Pacs2	36.781	36.8491	27.3144	24.4082	3.45626	3.31391	D9 C1
Slc25a 36	15.9431	16.1415	12.3524	9.23124	16.5443	15.3778	D9 C2
F5	0.758996	0.590777	2.20369	1.12585	1.13318	0.761803	D9 C4
Scarb1	111.294	123.742	142.689	138.683	110.922	103.486	D9 C4
Oas1g	1.55511	2.47166	3.57327	4.70136	1.43573	1.80266	D9 C4
Gm101 84	11.7065	14.7833	57.4741	55.246	14.2266	7.91273	D9 C4
Rplp0	1021.16	1081.9	14.8582	24.2835	1141.72	1290.47	D9 C2
Tcf7	9.88257	5.53335	2.88183	3.5862	1.52745	2.06531	D9 C1
Mfsd1 3a	7.72287	8.95649	2.27196	3.31684	8.22392	7.68726	D9 C2
H2-M9	5.86068	5.64903	1.80213	1.73256	2.35124	1.04768	D9 C1
Gda	26.3358	25.2219	39.584	27.0426	27.6062	26.4261	D9 C4
Prune2	8.2308	9.03507	4.64808	3.41347	6.42452	5.69171	D9 C2
Notch3	7.14692	6.53656	10.1641	7.53986	13.0096	12.2023	D9 C3
Adnp	9.68942	10.4393	6.18468	6.0327	9.98095	10.8926	D9 C2
Ptgis	23.3943	21.8748	10.6854	13.23	8.47089	7.92215	D9 C1

Sulf2	53.1113	52.2203	72.2672	58.6655	89.8856	85.6505	D9 C3
Pi15	3.31207	2.8086	10.8011	5.94173	19.3039	19.3757	D9 C3
Blcap	3.9939	3.82741	2.64361	1.25408	4.77296	3.36033	D9 C2
Sulf1	10.5419	7.25395	16.0184	11.0836	23.7783	20.1011	D9 C3
Ncaph 2	3.86646	4.52261	6.91167	6.43962	2.4843	2.7785	D9 C4
Zdhhc1 4	19.5097	22.2258	28.6321	29.3898	25.5394	23.0888	D9 C4
Gzme	126.791	119.219	73.2174	75.2501	33.1441	33.9273	D9 C1
Chd8	0.00407	0.003568	0.341241	3.25242	0.08519	2.75E-05	D9 C4
Adh7	12.2791	11.8675	33.7761	27.8619	22.4182	19.4957	D9 C4
Gpr88	0.023985	0.019485	0.290658	0.296914	3.29343	2.40523	D9 C3
Bcan	8.88769	6.45743	1.47914	1.99189	0.790744	0.775226	D9 C1
Fcrls	133.394	133.96	28.1057	33.8191	14.5067	14.7499	D9 C1
Ddx3y	24.9314	23.8209	20.1175	13.5231	0.130293	0	D9 C1
Eif2s3y	35.6279	33.3935	24.0859	20.7145	0.093842	0.061374	D9 C1
Cp	3.57891	2.60425	6.70701	4.34015	10.7128	7.57608	D9 C3
Col11a 1	114.061	57.8908	40.8647	33.1824	52.7265	45.9712	D9 C2
Adamt s14	13.0073	8.88231	6.60645	6.29482	5.21134	4.24511	D9 C1
Rspo3	4.22739	4.96239	10.8432	8.0854	22.4946	21.8422	D9 C3
Cdc6	1.1126	0.861263	2.31161	1.70184	0.693171	0.696153	D9 C4
Tmem 100	11.2301	10.2855	36.9603	30.7514	19.7067	17.6769	D9 C4
Wfdc1 7	343.437	379.538	98.5994	114.396	459.637	459.142	D9 C3

P4ha2	51.2748	40.7427	31.4582	31.7602	2.00608	0.781535	D9 C1
Zbtb16	4.26378	4.00969	1.83617	0.993747	3.83969	4.03276	D9 C2
Abhd15	29.146	27.5609	19.7986	14.7743	25.8563	25.1687	D9 C2
Trabd2b	3.78761	1.83312	1.34769	0.840616	5.17762	4.0327	D9 C2
Akap6	1.36083	1.08027	0.624592	0.379773	0.748922	0.940787	D9 C2
Csf2rb2	16.5264	16.8739	28.978	25.4541	19.3652	17.6125	D9 C4
Apcdd1	32.1976	30.9861	18.1887	16.0912	11.3257	13.512	D9 C1
Gm10557	11.4327	6.13047	3.74111	2.82865	11.2843	8.44042	D9 C2
Cd72	25.4864	26.0293	39.5805	45.8207	36.1188	32.7716	D9 C4
Trim5	1.72828	1.81429	5.38763	2.79898	2.82774	2.41295	D9 C4
Afg3l1	0.480711	0.888492	0.139229	0	0.71334	0.657799	D9 C2
Lig1	12.3693	17.0374	25.0274	24.9853	15.1499	15.9456	D9 C4
Tmem267	9.39681	8.02555	16.4286	13.8725	5.07038	6.07025	D9 C4
Foxs1	34.2189	30.5287	19.9765	22.7139	17.4041	14.6443	D9 C1
Prl2c3	27.8414	28.245	63.2139	42.2827	94.0957	96.5833	D9 C3
Gm5662	0.883748	0.766597	0.042446	0	0.525799	0.938959	D9 C2
Aldoc	3.15249	2.57087	6.81036	6.46247	1.74967	2.60419	D9 C4
Rtn4rl1	3.34704	4.11685	5.38638	4.72338	8.05028	7.41384	D9 C3
Hmgn2	61.9347	70.774	125.388	122.155	1.31809	11.1912	D9 C4
Tom1l2	15.8955	15.7105	11.6555	11.1366	3.41E-05	0.078265	D9 C1
Akap2	0.002891	0.013344	0.362818	0.435763	42.4284	38.2786	D9 C3

Col15a1	4.1869	4.27968	6.66455	5.20036	3.0995	2.92949	D9 C4
Hist1h4n	68.104	42.7313	15.9084	11.7124	36.8617	21.2249	D9 C2
Aoc3	2.47025	3.08654	4.67577	3.1254	11.5401	8.99225	D9 C3
Epb41l1	0	0	0.190692	0.347729	0	0	D9 C4
E2f1	3.73197	5.36122	6.74994	6.86063	3.69733	4.25627	D9 C4
Kitl	11.3418	13.0954	28.3454	18.1452	73.4891	57.7014	D9 C3
Foxo3	30.058	25.6942	22.9861	19.1555	30.953	27.2216	D9 C2
Prdm16	0.31968	0.432191	3.92E-09	3.82E-07	0.14869	0.955048	D9 C3
Zfp992	5.53095	4.8362	9.99782	6.48056	7.22125	5.35862	D9 C4
Zfp984	5.39983	5.59937	8.49268	8.37288	4.63589	4.94032	D9 C4
Tspan32	0.33588	0	1.62084	0.854321	2.26013	2.19837	D9 C3
Zfp995	1.0962	1.23209	0	0	0.618103	1.02433	D9 C2
Aspscr1	3.26383	1.15499	0	0	2.50782	1.18693	D9 C2
Notum	13.271	6.61853	3.09594	3.24852	1.43968	0.919005	D9 C1
Sult1a1	21.476	25.2078	14.5747	9.626	31.9528	26.5737	D9 C2
Ccdc163	0	0	1.31876	3.20896	0	0	D9 C4
Prdx1	323.598	262.792	429.867	308.288	961.635	631.104	D9 C3
Galnt18	26.5983	30.3567	52.5274	48.993	42.8383	38.1196	D9 C4
Ddx5	3.26804	84.6986	0	0	220.036	187.181	D9 C3
Trim12a	8.54131	7.52863	16.3339	15.7639	10.7438	9.53438	D9 C4
Trim34b	1.98404	1.83253	0.18926	0	1.40588	1.81779	D9 C2

Map6	12.4709	12.8404	18.9111	18.5123	15.3042	14.9229	D9 C4
Nars2	2.42272	1.21313	0.096496	0.137694	1.22136	2.54874	D9 C2
Tenm4	0.143363	0.212448	0.388305	0.362538	1.64972	1.48386	D9 C3
Cgn	0.81807	0.778523	1.2298	1.60353	0.694064	0.721583	D9 C4
Tnc	286.688	258.495	111.249	64.2982	165.219	164.065	D9 C2
Cacna1 g	0.155755	0.276862	0	0	0.103261	0.258442	D9 C2
Msi2	7.33377	6.1	4.70479	4.12861	7.26197	7.71568	D9 C2
Bcas3	0.522473	0.594946	0.114288	0	0.630768	0.397487	D9 C2
Abr	13.165	10.7791	8.13119	4.60232	2.56288	2.32918	D9 C1
Slc1a5	9.16257	14.2536	21.9508	18.5235	30.4857	33.3774	D9 C3
Aldh3a 1	42.6223	44.7962	113.066	108.254	49.8604	51.4031	D9 C4
Fbln1	4.21333	2.91756	6.34225	7.34977	3.63222	3.02081	D9 C4
Zfp429	1.31066	0.568686	4.28269	4.34808	1.47773	1.4045	D9 C4
Fgd3	35.2929	20.915	16.6264	12.7932	27.4629	20.0334	D9 C2
Ext1	47.2989	28.6449	22.2281	15.8739	29.8375	21.9898	D9 C2
Ripor2	1.10614	0.423379	0	0	1.86533	1.23452	D9 C2
Map4k 5	25.2283	21.5309	32.8031	24.0785	4.39115	3.25469	D9 C4
Prl2c2	49.2081	64.4336	204.813	183.37	178.634	155.428	D9 C4
Nek3	2.07115	0.31309	0	0	1.09521	0.989106	D9 C2
Bnip3l	0.663602	0.665044	0	0	0.347627	0.296707	D9 C2
Lmo2	24.8245	28.6504	34.6085	34.4957	21.8298	22.4333	D9 C4

Ccr5	37.0464	39.6361	27.2662	19.8831	22.3725	18.9454	D9 C1
Tcf7l2	0.668406	0.824421	0	0	0	0	D9 C1
Ctnnd1	93.3185	84.395	79.5984	56.529	94.5405	87.9276	D9 C2
Acad1 0	1.42114	1.81245	3.93522	3.51219	2.10252	2.97589	D9 C4
Colq	1.14705	1.09366	0.337761	0.277699	0.943697	0.79367	D9 C2
Oasl1	0	0	4.00082	3.81681	0	0.457682	D9 C4
Entpd1	21.5819	19.524	10.6975	9.52191	19.0654	16.8422	D9 C2
Adgrg2	0.444987	0.204893	0	0	0.21551	0.534749	D9 C2
Crim1	16.5598	17.6311	26.6671	18.3828	32.6135	27.7536	D9 C3
Ltbp1	35.891	30.3272	25.3535	19.5431	52.8178	50.0426	D9 C2
Idua	15.5962	18.2052	12.2403	10.2448	20.3884	19.8733	D9 C2
Maged 2	10.1927	12.759	22.9307	18.8652	36.3087	30.0475	D9 C3
Cxcl12	182.645	275.82	776.706	640.1	2002	1697.77	D9 C3
Col4a5	7.67148	8.2068	6.58112	4.43302	1.49537	1.24719	D9 C1
Srgap3	0	2.33E-09	0.706961	0.686397	0	0	D9 C4
Nrxn2	0.332245	0.371556	0.904889	0.870223	0.498914	0.504884	D9 C4
Nr2c2	7.96057	8.99703	6.65278	4.8887	10.3524	9.42094	D9 C2
Obsl1	0.879092	1.1731	2.27468	2.22155	7.17827	6.68851	D9 C3
Tpm1	610.681	600.342	716.007	691.002	407.215	401.03	D9 C4
H2-T24	6.09412	6.65323	12.6534	9.11037	9.36738	7.77517	D9 C4
Dysf	25.8864	21.5947	10.1231	10.7755	15.9166	16.6738	D9 C2

Heph	8.82816	7.79069	22.3449	15.4459	16.5955	15.3315	D9 C4
Smad2	3.94494	1.97199	0.023137	0	3.433	1.47796	D9 C2
Kmt5b	5.0993	3.95755	0.573335	0.000346	4.7049	5.97714	D9 C2
Slc5a3	3.34197	4.1778	7.81439	6.85385	14.287	13.9766	D9 C3
Ikbkg	30.0633	24.4842	20.784	11.8215	14.478	12.7881	D9 C1
Gpsm1	0	0	1.10762	1.93499	14.2373	13.5781	D9 C3
Nrp2	120.178	95.6471	79.997	67.3856	64.1535	63.2657	D9 C1
Col11a 2	13.2231	5.97664	0.804475	2.61839	1.07767	1.08598	D9 C1
Il1rn	49.876	54.8267	114.515	101.553	217.281	201.094	D9 C3
Plxdc2	24.5579	27.6488	39.2964	34.8966	57.7761	56.6783	D9 C3
Hs6st2	5.66003	7.33775	12.6404	10.2961	19.9079	15.5274	D9 C3
Tnk2	8.49059	5.80471	11.6476	10.1932	8.08918	9.67313	D9 C4
Timp1	1598.71	1234.69	823.715	934.27	494.329	508.112	D9 C1
Kng2	4.55872	9.09374	34.6537	30.0708	53.0158	49.9674	D9 C3
Cped1	8.35747	7.72793	13.768	8.63392	20.4579	18.4299	D9 C3
Gm155 83	0	0.107456	0.724102	0.433832	0	0.036019	D9 C4
Hdac7	0.947278	0.874381	0.010669	6.40E-09	2.23399	0.626256	D9 C2
Prr16	1.53576	1.91842	4.08721	2.83046	7.18838	4.92299	D9 C3
Entpd5	2.29762	2.00696	4.53E-05	2.25E-07	8.64142	9.0793	D9 C3
Postn	924.411	577.188	154.608	156.653	347.472	480.029	D9 C2
Hmga1	33.5271	43.9636	12.6222	20.7053	42.9439	54.9588	D9 C3

Rab3il1	39.9519	34.5012	26.9465	24.688	35.4034	32.8025	D9C2
Rapgef2	0	0.002806	0.411105	1.42618	0	0.002611	D9C4
Gm12191	257.568	268.516	113.17	132.279	393.571	353.625	D9C3
Gm13835	63.4382	51.6494	93.2903	115.837	70.4752	63.5901	D9C4
Mtus1	1.50091	0.392688	0	0.000157	34.2404	28.0623	D9C3
Fgfr1	145.296	121.595	73.8353	81.3989	27.1472	23.265	D9C1
Prss46	10.8787	8.42001	4.62378	5.08097	2.79585	3.4315	D9C1
Mlkl	15.2847	16.4826	24.1359	22.5546	18.1052	20.2323	D9C4
Rpl3-ps1	196.218	227.907	0	0	172.06	227.932	D9C2
Arsk	7.71473	6.63126	10.6954	8.49314	1.16967	0.459842	D9C4
Timm22	6.43549	10.8455	16.4816	14.1823	7.2032	8.15679	D9C4
Fam167a	7.7418	4.62319	1.79298	2.0072	1.19415	1.20706	D9C1
Palld	7.37881	6.27385	14.8106	8.24706	27.1045	19.1184	D9C3
Plagl1	7.57932	8.46619	18.4832	12.9104	39.291	36.5086	D9C3
Lmo4	8.97999	8.16075	12.4278	13.1028	51.1498	44.3563	D9C3
Rpl34-ps1	120.883	165.218	447.286	630.474	265.886	252.805	D9C4
Mlh1	0.383623	1.05434	0	0	0.417858	0.186446	D9C2
Slc25a22	3.24588	1.83171	0	0	1.76568	2.90087	D9C2
Tpr	0	0	2.83519	5.59716	0	0	D9C4
B4galnt3	2.91244	1.02103	0.341291	0.479834	0	0	D9C1
Serpinf1	617.162	489.568	279.977	278.321	230.745	210.277	D9C1

Pafah1b1	3.1069	2.39843	1.76223	0.765988	4.4298	2.70162	D9 C2
Trafd1	15.4078	9.53023	2.64873	0	20.532	13.4116	D9 C2
Sgms2	13.8755	8.39491	4.22682	2.19646	16.8954	17.5652	D9 C2
Atp10d	3.18798	2.62257	8.4234	6.10571	3.19865	2.49563	D9 C4
Gm15280	247.364	226.197	67.4506	56.4758	100.724	115.159	D9 C2
Msl1	5.57145	5.30524	0	0.647897	7.6885	5.53929	D9 C2
Hsd17b12	1.22108	0.667336	0	0	100.978	101.809	D9 C3
Arfrp1	7.49825	8.7678	4.56167	5.08874	8.66611	10.2251	D9 C3
Rgs12	0.71802	0.286607	0.115913	0	4.24466	5.73948	D9 C3
Sh3kbp1	0.204362	0.247081	0	0	13.5414	12.1922	D9 C3
Fam45a	12.6552	8.40254	28.3162	22.8163	17.5934	18.2148	D9 C4
Phf19	0	0.120327	0.72851	0.976955	0.200808	0	D9 C4
Ctsc	31.1884	44.197	21.176	10.1849	169.133	153.826	D9 C3
Tsc22d4	6.4116	5.16434	3.27003	2.66413	6.72796	5.00429	D9 C2
Hal	0.985552	1.20275	3.41208	3.61976	2.09814	1.50411	D9 C4
Sorbs2	8.00742	6.51487	3.54217	2.62526	14.3186	12.4947	D9 C2
Smu1	2.40215	2.92573	2.88E-08	0.260625	1.66105	3.2645	D9 C2
Pif1	0.577405	0.895042	2.36E-07	6.00E-08	1.32406	0.560952	D9 C2
St18	0.349	0.283965	0	1.11E-10	0.4995	0.323991	D9 C2
Dnajc7	20.63	19.1064	13.1877	7.54627	20.672	17.5645	D9 C2
Limch1	1.7374	2.23795	0.510491	0	6.74066	7.06588	D9 C3

Penk	1.2124	3.47856	8.55059	7.18054	8.13693	8.55432	D9 C4
Fbxo6	11.8861	12.0543	4.74003	4.94478	16.1688	18.2131	D9 C3
Mtm1	1.0364	0.567684	0.296113	0.079658	0.02254	0	D9 C1
Slx4ip	0.936503	1.07629	0	0.001109	1.74325	1.09054	D9 C2
Ubc	369.766	344.04	129.202	123.902	357.997	372.304	D9 C2
Vill	19.6605	20.6926	36.9391	40.8073	1.53328	1.09734	D9 C4
Acap3	2.51869	2.80603	1.2655	1.20625	12.8532	12.3395	D9 C3
Gnao1	3.82195	3.59255	5.83289	4.52575	10.1576	8.84372	D9 C3
Slfn9	0.688886	0.478995	0.085477	0	1.39984	0.946133	D9 C2
Gm6969	61.4557	64.4277	0	0	68.5957	47.8499	D9 C2
Slc38a6	28.8146	25.5107	15.1982	13.3411	24.2238	22.0366	D9 C2
Tnrc6c	1.18269	0.920896	0.707841	0.373198	6.6815	7.67842	D9 C3
Cep85	181.866	186.088	155.691	98.8368	347.528	303.229	D9 C3
Usp36	0.377248	0.441766	1.38898	1.29213	0.533687	0.477878	D9 C4
Arhgap18	0	2.24E-07	1.0227	1.12895	5.44E-08	0.001285	D9 C4
Hps5	0	0	0.852947	0.41995	9.98781	6.73709	D9 C3
Haus5	0	0	3.56346	3.22222	0	0	D9 C4
Eif4enif1	7.84256	5.03038	0	0.540719	0	0	D9 C1
Oasl2	5.6555	5.22115	8.6992	8.42714	6.33419	5.22655	D9 C4
Usp16	0.598409	0.778824	0	5.51E-10	0.411355	2.14405	D9 C3
Actn4	18.537	15.2566	24.8227	32.0074	11.782	19.3383	D9 C4

Mcf2l	0.106486	0.595972	0	0	0.324422	0.414511	D9 C2
Cul7	0	0	0.372127	0.382417	0	0	D9 C4
Tle2	3.9163	2.25858	0	0	7.33206	2.3173	D9 C2
Slc43a 2	4.70201	3.54633	2.64817	1.09671	5.24803	3.5722	D9 C2
Capn3	0.555916	0.474541	0	0	0	0	D9 C1
Vegfa	23.0656	29.2597	3.3161	6.79572	22.5434	28.6533	D9 C2
Lipo2	0.206853	0.39017	1.03213	0.996177	0.264148	0.370793	D9 C4
Retreg 3	1.06072	2.69849	9.53E-07	0	1.237	1.9305	D9 C2
Psmd6	14.0462	13.2198	8.10509	4.04338	15.8123	14.2161	D9 C2
Abi1	13.8947	17.8128	6.45088	4.4407	13.4577	16.7703	D9 C2
Clk1	29.0135	25.8881	16.4891	14.0474	24.7088	23.9859	D9 C2
Slc4a4	3.0782	2.89605	7.49192	4.49268	4.92526	4.23017	D9 C4
Adgb	5.74598	5.08827	10.1944	9.83856	3.53636	1.77681	D9 C4
Tspyl2	2.68973	0.655939	2.92E-08	0	0.458586	1.76806	D9 C2
15000 09L16 Rik	6.1463	8.3519	10.9955	13.1421	24.5984	22.7654	D9 C3
Tor3a	0	0	0.400261	0.297343	0	0	D9 C4
Ece1	15.3859	18.812	28.4235	21.2252	52.7087	50.576	D9 C3
Shf	4.90764	5.52851	2.30357	2.24134	0	0	D9 C1
Uba1	10.4803	6.09501	2.50846	0.716267	14.0922	10.8094	D9 C2
Rps2	2.81566	1.7791	0	0	2.16324	2.56054	D9 C2
Pole	0	0.000379	1.14882	1.79675	3.0425	2.21679	D9 C3

Odf2	0.471763	0.477256	2.12295	2.8511	0.758146	0.743896	D9 C4
Tnfrsf2 3	35.2827	30.6188	22.0135	14.7733	16.4119	16.7346	D9 C1
Chid1	0.049279	6.18E-05	0.216159	5.0749	0.000225	0.004867	D9 C4
68204 31F20 Rik	79.3751	57.0322	53.371	31.2139	98.8588	74.939	D9 C2
Slc25a 51	11.2626	8.14817	4.54923	1.98171	7.37464	9.70947	D9 C2
Cc2d1a	3.66258	2.77764	1.13484	1.00244	2.97514	2.58476	D9 C2
Hp1bp 3	6.24362	5.6115	3.1245	1.44661	24.3972	20.4557	D9 C3
Gk	0.005168	0	0.672681	1.21369	0	0	D9 C4
Pappa 2	3.06132	2.50497	1.43766	1.528	5.95601	3.53128	D9 C2
Col22a 1	4.95764	2.21466	0.777671	1.08222	0.520536	0.414854	D9 C1
Plod2	91.0505	63.4846	48.1484	43.9121	87.1879	77.0219	D9 C2
Stxbp2	6.89405	4.86116	0	0.536072	4.82891	4.98177	D9 C2
Psma3	26.8879	19.0118	62.6639	61.7762	44.8608	32.4486	D9 C4
Pcdh1	0.773778	0.912011	1.33766	1.5304	7.57928	6.7283	D9 C3
Unkl	0.554762	0.404899	0	0	0.410198	0.520865	D9 C2
Tcf19	6.41144	7.23462	11.5982	12.0208	8.37597	7.02829	D9 C4
Rb1cc1	8.91855	7.24158	5.80568	4.69148	4.47024	1.71691	D9 C1
Adra1a	0.728012	0.626247	0	0	3.31225	2.68199	D9 C3
Aebp2	5.44579	6.67107	0	0	2.10441	4.11266	D9 C2
C1s1	5.73189	5.97006	17.2873	15.5293	32.2195	25.0093	D9 C3
Irf8	0.867844	0.921103	0.438481	0.261495	1.04728	0.67691	D9 C2

Ms4a7	13.5369	10.6329	7.68465	5.66524	75.246	68.2971	D9 C3
Clcn7	7.69619	6.00604	4.20982	1.75887	4.75231	4.62408	D9 C2
Spata1 3	16.9209	13.3819	8.76121	7.14467	11.7599	15.005	D9 C2
Gbe1	40.287	41.7942	66.4573	52.0139	46.6925	45.2775	D9 C4
Map4k 4	3.1684	3.11878	4.31892	6.41631	2.2856	3.03242	D9 C4
Eps8l1	0.181318	0.182702	1.08456	0.619638	0.218242	0.081237	D9 C4
Mest	22.4896	19.7628	14.2164	10.237	27.8281	24.0241	D9 C2
Hnrnpc	16.4719	17.4621	26.8301	25.5468	19.136	21.9081	D9 C4
Tgfb1i 1	1.68823	1.91227	3.57546	2.33631	1.81495	0.945744	D9 C4
Phlda1	30.7873	27.0042	46.2989	44.1664	41.638	38.0223	D9 C4
Trps1	7.73169	6.65679	4.98623	4.47781	6.55077	6.90417	D9 C2
Lrrc32	9.81019	10.5779	27.3566	28.9984	18.2827	20.3958	D9 C4
Tsku	0	0.302014	1.12511	2.24894	0	0	D9 C4
Rcbtb2	9.48987	6.80816	1.8345	0.042855	7.62569	9.89966	D9 C2
Galnt9	0.486213	0.782371	0	0	0.581337	0.828538	D9 C2
Phf11b	3.08303	3.99185	6.86027	5.55958	3.95453	3.85558	D9 C4
Agpat3	23.6338	27.9318	32.4052	29.9602	5.75418	5.89998	D9 C4
Cmip	69.7818	54.7969	52.9484	41.0341	0	0	D9 C1
Brinp3	19.6596	14.9783	25.2404	20.1757	15.399	16.3821	D9 C4
Vldlr	7.44771	3.83971	2.49788	2.57285	1.08088	2.65949	D9 C1
Vgll3	52.1497	49.5346	43.3335	32.0753	52.1862	48.9787	D9 C2

Tonsl	2.82239	2.66293	4.26146	3.68181	3.04679	2.67779	D9 C4
Il7	1.73719	1.54386	3.27111	2.35687	7.66782	4.48493	D9 C3
Gdf10	0.721667	1.78609	4.25155	4.27767	15.5922	15.8847	D9 C3
Islr	26.6948	31.7239	39.0979	39.7921	60.0467	61.2103	D9 C3
Ppard	0.99434	3.01354	0	0	0	0	D9 C2
Slit1	3.33144	2.88368	0.916101	0.720616	3.12542	1.98388	D9 C2
Ly6e	0.507787	1.94131	0	0	464.233	413.282	D9 C3
Rai14	60.1053	55.3268	99.5574	77.9532	67.8642	66.3337	D9 C4
Plec	0.196463	0.868118	1.93712	11.138	0.209474	0.582796	D9 C4
Shoc2	1.25267	2.74307	0	0.001007	0.919198	1.16283	D9 C2
Thbs2	404.057	337.676	219.265	170.24	273.412	294.245	D9 C2
Dhrs3	39.1714	50.5298	66.2388	73.4902	9.8724	13.1138	D9 C4
Prr5l	1.17894	1.65842	2.60455	2.85016	2.31916	1.64211	D9 C4
Lifr	36.1718	35.9925	19.004	17.8118	30.9792	42.3349	D9 C2
Ang	18.0126	21.4865	28.1675	26.1522	17.5243	14.974	D9 C4
Tbc1d25	0.835135	1.57013	3.56E-10	8.18E-06	0.811186	1.72165	D9 C2
Pgbd5	8.11162	8.07293	2.50391	3.10586	1.3596	2.92276	D9 C1
E2f7	0	0	5.96817	5.30525	1.92385	1.81696	D9 C4
Snx14	0	0	0.289662	0.663384	0	0	D9 C4
Il1rl1	7.29328	6.12639	3.61894	4.55795	2.5193	2.27166	D9 C1
Irx3	45.7433	33.6149	62.9828	64.7599	53.6149	54.1906	D9 C4

Esd	613.453	611.023	723.607	771.122	789.763	671.675	D9 C4
Mta3	5.04805	3.44783	2.73621	1.78884	5.24316	3.87743	D9 C2
Aste1	0	0	4.01662	1.7939	0	0	D9 C4
Gm20605	23.0672	23.8875	29.2816	29.314	28.0924	24.5248	D9 C4
Abcc3	35.7628	41.3972	71.2394	56.7303	38.6069	38.2056	D9 C4
Fignl2	1.10E-09	3.59E-06	1.07921	0.52186	0	0.000114	D9 C4
St6gal1	9.90089	12.7648	15.9927	14.785	13.2022	11.2289	D9 C4
Sirpa	174.056	173.743	239.542	222.273	28.6845	44.76	D9 C4
Erdr1	1.87901	2.45887	143.202	152.459	102.558	79.0787	D9 C4
Gm16867	5.86478	6.26035	1.0332	0.518183	4.12275	4.99395	D9 C2
Gm21887	6.35248	8.87881	178.541	194.791	47.9885	52.6317	D9 C4
Gm9625	8.23332	11.9315	30.3905	40.973	4.17554	4.59704	D9 C4
Ccnd3	7.91099	5.45941	0.818006	1.17653	16.5499	18.2423	D9 C3
Gm8116	1.90992	2.41128	8.59494	10.1875	2.61811	2.91871	D9 C4
Gapdh	0	0	3.28259	3.14205	1530.25	1398.61	D9 C3
Plac9b	5.35204	12.8822	22.837	35.1012	88.5378	97.232	D9 C3
Gm9780	15.8226	17.2322	41.7204	66.3162	211.921	241.724	D9 C3
Ism1	6.10005	6.92271	15.9356	11.9276	42.8697	42.3189	D9 C3
Atf7	18.9986	16.3456	15.3077	11.214	19.3327	16.6068	D9 C2
Srgap2	16.4349	16.7447	11.8826	8.67509	15.3665	14.599	D9 C2
Eif4a2	31.4642	22.7251	20.3196	10.5855	1.54508	3.82588	D9 C1

Ptprn	1.20449	0.555993	3.85801	2.77512	24.6175	23.8077	D9 C3
Pik3r4	3.57519	2.89329	2.56808	1.39178	4.245	2.9559	D9 C2
Fam135a	3.709	0.546573	0.044361	2.90E-05	1.86598	0.71847	D9 C2
Morf4l1	4.59E-05	0	14.754	6.82289	0.006457	0.001159	D9 C4
Nkain2	0.114357	0.267817	0.608801	0.459158	0.145114	0.303208	D9 C4
Thap6	3.5364	2.83672	2.24474	1.22747	3.8391	3.04022	D9 C2
Mbnl1	11.0557	10.2249	3.32898	3.46307	23.9883	24.5726	D9 C3
Ncam1	8.97632	10.7274	4.62878	5.89968	4.28685	2.78069	D9 C1
Igsf10	6.99186	5.81924	4.56966	3.13845	2.03231	1.6569	D9 C1
Txnip	10.0698	15.151	25.1004	22.1957	9.30149	11.6776	D9 C4
Chfr	6.35284	6.16106	0.959086	0.556154	1.93163	0.712703	D9 C1
Rabggtb	2.79726	4.89844	0	1.70E-06	1.00016	3.59879	D9 C2
Stap1	5.67145	4.66808	3.58371	2.39088	7.20516	5.37115	D9 C2
Pfkfb4	18.8156	15.3988	6.76356	10.4718	15.3138	11.8117	D9 C2
Tapt1	0	0.070191	1.47995	1.66972	0	0	D9 C4
Miga1	17.9267	15.7922	9.66906	10.0944	16.5292	15.0836	D9 C2
Anapc5	8.39362	9.30027	21.5394	21.3663	8.89914	10.4941	D9 C4
Ncor2	27.3593	21.3553	35.7041	34.8992	22.5261	26.5454	D9 C4
Rchy1	2.05924	1.88893	0.548004	0.463042	3.42963	1.28961	D9 C2
Brca2	31.4752	21.1657	4.84839	10.6374	1.711	3.00402	D9 C1
Uso1	0	0	0.397116	2.34046	0	0	D9 C4

Bend4	0	0	0.285057	0.158696	0.3318	0.125713	D9 C4
Slc30a 9	9.44961	9.25119	13.9366	13.5887	11.2735	9.54485	D9 C4
Pparg	4.19238	3.30091	9.89971	7.41995	20.2098	14.4304	D9 C3
Rarres 2	0.969963	0.613295	4.49422	3.70995	17.791	15.5219	D9 C3
Tmem 176a	15.4998	13.2518	21.1003	22.5227	99.5901	113.077	D9 C3
Creb5	3.61292	3.34708	1.4884	1.46947	0.709548	0.226306	D9 C1
Plin1	0.120721	0.045383	0.869213	0.485609	2.59976	2.73817	D9 C3
Lsr	0.197819	0	0.831689	1.30044	0.887896	1.14759	D9 C4
Ptcd3	0.984289	0.341983	0	0.095516	0.880411	1.09003	D9 C2
Nrg1	0.89069	0.601237	0.295907	0.218174	0.643689	0.727326	D9 C2
C4300 49E01 Rik	11.2902	6.29782	1.41892	3.18834	0.648619	0.753533	D9 C1
Akap1 3	4.49572	3.11203	1.92062	0.74286	4.28598	2.75655	D9 C2
Smarc a2	3.1656	4.38754	8.78386	13.08	11.9505	14.1703	D9 C4
Chd9	534.268	531.213	404.456	355.169	576.394	470.693	D9 C2
Hook2	0.466867	0.549788	0	0	4.76306	4.34153	D9 C3
Prmt9	2.35357	0.645529	0	0	0.540339	0.71522	D9 C2
Rtp4	21.2338	24.8783	55.1862	45.2029	27.5862	23.934	D9 C4
Nkd1	1.89759	1.59691	3.8298	3.73885	5.56968	4.90385	D9 C3
Dock1	2.16635	1.85516	0.762452	0.688268	1.68193	1.78511	D9 C2
Gm590 5	13.0854	12.9403	0	0	3.84821	3.05684	D9 C2
Mcm5	23.0794	22.8563	13.279	15.6093	25.7979	21.6067	D9 C4

Olfr1033	3992.72	2624.52	1343.52	950.441	1876.06	1738.97	D9 C2
Gm46223	603.895	626.9	434.823	319.445	780.606	666.252	D9 C2
Tagln	52.0103	55.3583	104.811	79.1006	631.043	640.812	D9 C3
Cacna1a	4.05E-05	0.000738	1.72803	0.771631	10.4071	9.07025	D9 C3
Metap2	35.5765	20.8864	57.9491	34.1283	36.9162	21.1853	D9 C4
AC109138.8	1406.86	936.439	349.629	388.345	815.442	708.901	D9 C2
Cryab	164.154	130.544	184.305	193.009	106.058	95.9471	D9 C4
7-Sep	3.43981	3.06185	2.39667	1.61057	3.78599	3.09798	D9 C2
Aplp2	46.9847	39.8342	72.9715	45.5499	115.585	81.6124	D9 C3
Cfd	0	0	1.14596	2.44184	8.28832	27.8196	D9 C3
Ppfia2	0	0.029732	0.117961	0.196467	1.9023	1.48222	D9 C3
Atp2b1	12.9104	10.8803	4.09167	3.8075	11.3288	9.42576	D9 C2
Lilr4b	16.2612	13.8027	31.5752	17.9997	23.1383	20.9048	D9 C4
Plekhj1	1.87195	3.25685	7.59095	7.17593	1.75929	2.63326	D9 C4
Cited2	2.69176	1.77814	3.93322	3.45743	155.409	145.351	D9 C3
Nol8	2.00956	2.62286	0	4.54E-10	1.67027	0.787167	D9 C2
E2f3	6.44419	8.55291	3.21476	4.02394	1.92446	1.80909	D9 C1
B3galnt2	13.1907	3.71985	0	0	4.33091	9.37176	D9 C2
Habp4	3.40198	3.28187	1.70251	1.82975	3.85153	3.15076	D9 C2
Aoah	1.71913	1.47649	0.244007	0	2.48315	2.85148	D9 C2
Fbln5	76.2478	81.6625	125.24	117.815	38.556	37.0998	D9 C4

Daam2	2.66016	2.31042	4.11962	3.83798	7.22977	7.05998	D9 C3
Ppp2r2 a	22.4141	23.2627	18.4657	12.9545	40.5206	35.0138	D9 C3
Trappc 8	25.6426	21.3341	20.0684	14.4829	0.775957	0.348956	D9 C1
Btrc	1.76249	1.63775	0.850331	0.53147	2.89897	2.17742	D9 C2
Dapk1	13.7503	9.12227	8.03048	6.43362	39.9342	34.294	D9 C3

Supplementary Table 7 Primer used for genotyping of all transgenic mice generated in this study

Primer Name	sequence(5'-3')	Product Size (bp)	Usage
miR338KO-F	TACCCCAGGAGGACTGTGTC	649 (300)	genotyping for Mir338 KO;WT(KO)
miR338KO-R	TAGCCACTCTGCAGGGAACT		
miR338CKO-F	CACTCTGCAGGGAACTTTATCATT	440 (564)	genotyping for Mir338-flox allele;WT(with flox)
miR338CKO-R	GCCGCTTGTCCGTTGCCACTTC		
OsxCre-F	GAGAATAGGAACTTCGGAATAGTAAC	198	genotyping for OsxCre transgene
OsxCre-R	CCCTGGAAGTGACTAGCATTG		
Runx2-F	CACGGAGCACAGGAAGTTGGG		
Runx2-R_KO	AAGATGGATTGCACGCAGGTTCTC	1000	genotyping for Runx2 KO; KO allele
Runx2_R_WT	TGAGCGACGTGAGCCCGGTGG	281	genotyping for Runx2 KO; WT allele
Fgfr2-q-F	ACCACACCTACCACCTCGAT		qRT-PCR primer
Fgfr2-q-R	TGGGCATCGCTGTAAACCTT		
Fgfr3-q-F	TCTAGGCAGTGACGTGGAGT		qRT-PCR primer
Fgfr3-q-R	TCTAGCTCCTTGTCGGTGGT		
Runx2-q-F	CCGCACGACAACCGCACCAT		qRT-PCR primer
Runx2-q-R	CGCTCCGGCCCACAAATCTC		
